# Global, regional, and national progress towards the 2030 global nutrition targets and forecasts to 2050: a systematic analysis for the Global Burden of Disease Study 2021

**DOI:** 10.1016/S0140-6736(24)01821-X

**Published:** 2024-12-21

**Authors:** Global Nutrition Target Collaborators, Michael Benjamin Arndt, Yohannes Habtegiorgis Abate, Mohsen Abbasi-Kangevari, Samar Abd ElHafeez, Michael Abdelmasseh, Sherief Abd-Elsalam, Deldar Morad Abdulah, Rizwan Suliankatchi Abdulkader, Hassan Abidi, Olumide Abiodun, Richard Gyan Aboagye, Hassan Abolhassani, Yonas Derso Abtew, Eman Abu-Gharbieh, Niveen ME Abu-Rmeileh, Juan Manuel Acuna, Kidist Adamu, Denberu Eshetie Adane, Isaac Yeboah Addo, Daniel Adedayo Adeyinka, Qorinah Estiningtyas Sakilah Adnani, Aanuoluwapo Adeyimika Afolabi, Fatemeh Afrashteh, Saira Afzal, Antonella Agodi, Bright Opoku Ahinkorah, Aqeel Ahmad, Sajjad Ahmad, Tauseef Ahmad, Ali Ahmadi, Ali Ahmed, Luai A A Ahmed, Marjan Ajami, Budi Aji, Hossein Akbarialiabad, Maxwell Akonde, Hanadi Al Hamad, Yazan Al Thaher, Ziyad Al-Aly, Khalid F Alhabib, Robert Kaba Alhassan, Beriwan Abdulqadir Ali, Syed Shujait Ali, Yousef Alimohamadi, Syed Mohamed Aljunid, Hesham M Al-Mekhlafi, Sami Almustanyir, Mahmoud A Alomari, Alaa B Al-Tammemi, Khalid A Altirkawi, Nelson Alvis-Guzman, Nelson J Alvis-Zakzuk, Edward Kwabena Ameyaw, Tarek Tawfik Amin, Sohrab Amiri, Hubert Amu, Dickson A Amugsi, Tadele Fentabel Fentabil Anagaw, Robert Ancuceanu, Dhanalakshmi Angappan, Alireza Ansari-Moghaddam, Ernoiz Antriyandarti, Davood Anvari, Anayochukwu Edward Anyasodor, Jalal Arabloo, Aleksandr Y Aravkin, Hany Ariffin, Timur Aripov, Mesay Arkew, Benedetta Armocida, Ashokan Arumugam, Ni Ketut Aryastami, Malke Asaad, Zatollah Asemi, Mulu Tiruneh Asemu, Mohammad Asghari-Jafarabadi, Thomas Astell-Burt, Seyyed Shamsadin Athari, Gamechu Hunde Atomsa, Prince Atorkey, Maha Moh'd Wahbi Atout, Avinash Aujayeb, Mamaru Ayenew Awoke, Sina Azadnajafabad, Rui M S Azevedo, Darshan B B, Ashish D Badiye, Nayereh Baghcheghi, Nasser Bagheri, Sara Bagherieh, Atif Amin Baig, Jennifer L Baker, Madhan Balasubramanian, Ovidiu Constantin Baltatu, Maciej Banach, Palash Chandra Banik, Martina Barchitta, Till Winfried Bärnighausen, Ronald D Barr, Amadou Barrow, Lingkan Barua, Azadeh Bashiri, Pritish Baskaran, Saurav Basu, Alehegn Bekele, Sefealem Assefa Belay, Uzma Iqbal Belgaumi, Shelly L Bell, Luis Belo, Derrick A Bennett, Isabela M Bensenor, Girma Beressa, Amiel Nazer C Bermudez, Habtamu B Beyene, Akshaya Srikanth Bhagavathula, Nikha Bhardwaj, Pankaj Bhardwaj, Sonu Bhaskar, Natalia V Bhattacharjee, Zulfiqar A Bhutta, Saeid Bitaraf, Virginia Bodolica, Milad Bonakdar Hashemi, Dejana Braithwaite, Muhammad Hammad Butt, Zahid A Butt, Daniela Calina, Luis Alberto Cámera, Luciana Aparecida Campos, Chao Cao, Rosario Cárdenas, Márcia Carvalho, Carlos A Castañeda-Orjuela, Alberico L Catapano, Maria Sofia Cattaruzza, Francieli Cembranel, Ester Cerin, Joshua Chadwick, Julian Chalek, Eeshwar K Chandrasekar, Jaykaran Charan, Vijay Kumar Chattu, Kirti Chauhan, Ju-Huei Chien, Abdulaal Chitheer, Sonali Gajanan Choudhari, Enayet Karim Chowdhury, Dinh-Toi Chu, Isaac Sunday Chukwu, Sheng-Chia Chung, Rafael M Claro, Alyssa Columbus, Samuele Cortese, Natalia Cruz-Martins, Bashir Dabo, Omid Dadras, Xiaochen Dai, Emanuele D'Amico, Lalit Dandona, Rakhi Dandona, Isaac Darban, Gary L Darmstadt, Aso Mohammad Darwesh, Amira Hamed Darwish, Jai K Das, Saswati Das, Kairat Davletov, Fernando Pio De la Hoz, Aklilu Tamire Debele, Dessalegn Demeke, Solomon Demissie, Edgar Denova-Gutiérrez, Hardik Dineshbhai Desai, Abebaw Alemayehu Desta, Samath Dhamminda Dharmaratne, Meghnath Dhimal, Diana Dias da Silva, Daniel Diaz, Mengistie Diress, Shirin Djalalinia, Saeid Doaei, Deepa Dongarwar, Haneil Larson Dsouza, Sareh Edalati, Hisham Atan Edinur, Michael Ekholuenetale, Temitope Cyrus Ekundayo, Iffat Elbarazi, Islam Y Elgendy, Muhammed Elhadi, Omar Abdelsadek Abdou Elmeligy, Habitu Birhan Eshetu, Juan Espinosa-Montero, Habtamu Esubalew, Farshid Etaee, Werku Etafa, Adeniyi Francis Fagbamigbe, Ildar Ravisovich Fakhradiyev, Luca Falzone, Carla Sofia e Sá Farinha, Sam Farmer, Abidemi Omolara Fasanmi, Ali Fatehizadeh, Valery L Feigin, Alireza Feizkhah, Xiaoqi Feng, Pietro Ferrara, Getahun Fetensa, Florian Fischer, Ryan Fitzgerald, David Flood, Nataliya A Foigt, Morenike Oluwatoyin Folayan, Kayode Raphael Fowobaje, Richard Charles Franklin, Takeshi Fukumoto, Muktar A Gadanya, Abhay Motiramji Gaidhane, Santosh Gaihre, Emmanuela Gakidou, Yaseen Galali, Nasrin Galehdar, William M Gardner, Priyanka Garg, Teferi Gebru Gebremeskel, Urge Gerema, Lemma Getacher, Motuma Erena Getachew, Solomon Getawa, Kazem Ghaffari, Seyyed-Hadi Ghamari, Mohammad Ghasemi Nour, Fariba Ghassemi, Nermin Ghith, Maryam Gholamalizadeh, Ali Gholami, Ali Gholamrezanezhad, Sherief Ghozy, Paramjit Singh Gill, Tiffany K Gill, James C Glasbey, Mahaveer Golechha, Pouya Goleij, Davide Golinelli, Houman Goudarzi, Michal Grivna, Habtamu Alganeh Guadie, Mohammed Ibrahim Mohialdeen Gubari, Temesgen Worku Gudayu, Avirup Guha, Damitha Asanga Gunawardane, Anish Kumar Gupta, Bhawna Gupta, Rahul Gupta, Sapna Gupta, Veer Bala Gupta, Vivek Kumar Gupta, Hailey Hagins, Arvin Haj-Mirzaian, Alexis J Handal, Asif Hanif, Graeme J Hankey, Harapan Harapan, Arief Hargono, Josep Maria Haro, Ahmed I Hasaballah, Md Mehedi Hasan, Hamidreza Hasani, Abdiwahab Hashi, Soheil Hassanipour, Rasmus J Havmoeller, Simon I Hay, Khezar Hayat, Jiawei He, Mahsa Heidari-Foroozan, Claudiu Herteliu, Kamran Hessami, Demisu Zenbaba Heyi, Kamal Hezam, Yuta Hiraike, Ramesh Holla, Praveen Hoogar, Sheikh Jamal Hossain, Mehdi Hosseinzadeh, Mihaela Hostiuc, Sorin Hostiuc, Soodabeh Hoveidamanesh, Junjie Huang, Kyle Matthew Humphrey, Salman Hussain, Foziya Mohammed Hussien, Bing-Fang Hwang, Licia Iacoviello, Pulwasha Maria Iftikhar, Olayinka Stephen Ilesanmi, Irena M Ilic, Milena D Ilic, Mustapha Immurana, Leeberk Raja Inbaraj, Farideh Iravanpour, Sheikh Mohammed Shariful Islam, Farhad Islami, Nahlah Elkudssiah Ismail, Hiroyasu Iso, Gaetano Isola, Masao Iwagami, Chidozie Declan Iwu, Linda Merin J, Louis Jacob, Haitham Jahrami, Mihajlo Jakovljevic, Elham Jamshidi, Manthan Dilipkumar Janodia, Krishnamurthy Jayanna, Sathish Kumar Jayapal, Shubha Jayaram, Rime Jebai, Alelign Tasew Jema, Bijay Mukesh Jeswani, Jost B Jonas, Abel Joseph, Nitin Joseph, Charity Ehimwenma Joshua, Jacek Jerzy Jozwiak, Mikk Jürisson, Billingsley Kaambwa, Ali Kabir, Zubair Kabir, Vidya Kadashetti, Vineet Kumar Kamal, Bhushan Dattatray Kamble, Himal Kandel, Neeti Kapoor, Ibraheem M Karaye, Patrick DMC Katoto, Joonas H Kauppila, Harkiran Kaur, Gbenga A Kayode, Worku Misganaw Kebede, Jemal Yusuf Kebira, Tibebeselassie S Keflie, Jessica A Kerr, Mohammad Keykhaei, Yousef Saleh Khader, Himanshu Khajuria, Nauman Khalid, Mohammad Khammarnia, M Nuruzzaman Khan, Moien AB Khan, Taimoor Khan, Yusra H Khan, Javad Khanali, Shaghayegh Khanmohammadi, Khaled Khatab, Moawiah Mohammad Khatatbeh, Sorour Khateri, Mahalaqua Nazli Khatib, Hamid Reza Khayat Kashani, Jagdish Khubchandani, Zemene Demelash Kifle, Gyu Ri Kim, Ruth W Kimokoti, Adnan Kisa, Sezer Kisa, Farzad Kompani, Shivakumar KM Marulasiddaiah Kondlahalli, Hamid Reza Koohestani, Oleksii Korzh, Sindhura Lakshmi Koulmane Laxminarayana, Ai Koyanagi, Kewal Krishan, Vijay Krishnamoorthy, Barthelemy Kuate Defo, Burcu Kucuk Bicer, Mohammed Kuddus, G Anil Kumar, Manasi Kumar, Nithin Kumar, Almagul Kurmanova, Om P Kurmi, Dian Kusuma, Carlo La Vecchia, Ben Lacey, Dharmesh Kumar Lal, Anders O Larsson, Kamaluddin Latief, Caterina Ledda, Paul H Lee, Sang-woong Lee, Wei-Chen Lee, Yo Han Lee, Jacopo Lenzi, Ming-Chieh Li, Wei Li, Virendra S Ligade, Stephen S Lim, Paulina A Lindstedt, Chun-Han Lo, Justin Lo, Rakesh Lodha, Arianna Maever Loreche, László Lorenzovici, Stefan Lorkowski, Farzan Madadizadeh, Áurea M Madureira-Carvalho, Preetam Bhalchandra Mahajan, Konstantinos Christos Makris, Elaheh Malakan Rad, Ahmad Azam Malik, Tauqeer Hussain Mallhi, Deborah Carvalho Malta, Helena Manguerra, Abdoljalal Marjani, Santi Martini, Miquel Martorell, Awoke Masrie, Elezebeth Mathews, Andrea Maugeri, Maryam Mazaheri, Rishi P Mediratta, Man Mohan Mehndiratta, Yohannes Adama Melaku, Walter Mendoza, Ritesh G Menezes, George A Mensah, Alexios-Fotios A Mentis, Tuomo J Meretoja, Tomislav Mestrovic, Tomasz Miazgowski, Ted R Miller, GK Mini, Mojgan Mirghafourvand, Andreea Mirica, Erkin M Mirrakhimov, Moonis Mirza, Sanjeev Misra, Prasanna Mithra, Karzan Abdulmuhsin Mohammad, Abdollah Mohammadian-Hafshejani, Shafiu Mohammed, Mohammad Mohseni, Ali H Mokdad, Lorenzo Monasta, Mohammad Ali Moni, Maryam Moradi, Yousef Moradi, Shane Douglas Morrison, Vincent Mougin, Sumaira Mubarik, Ulrich Otto Mueller, Francesk Mulita, Daniel Munblit, Efren Murillo-Zamora, Christopher J L Murray, Ghulam Mustafa, Ahamarshan Jayaraman Nagarajan, Vinay Nangia, Sreenivas Narasimha Swamy, Zuhair S Natto, Muhammad Naveed, Biswa Prakash Nayak, Seyed Aria Nejadghaderi, Georges Nguefack-Tsague, Josephine W Ngunjiri, Phuong The Nguyen, QuynhAnh P Nguyen, Robina Khan Niazi, Chukwudi A Nnaji, Nurulamin M Noor, Jean Jacques Noubiap, Chisom Adaobi Nri-Ezedi, Dieta Nurrika, Vincent Ebuka Nwatah, Bogdan Oancea, Kehinde O Obamiro, Onome Bright Oghenetega, Ropo Ebenezer Ogunsakin, Hassan Okati-Aliabad, Akinkunmi Paul Okekunle, Daniel Micheal Okello, Osaretin Christabel Okonji, Andrew T Olagunju, Diriba Dereje Olana, Gláucia Maria Moraes Oliveira, Bolajoko Olubukunola Olusanya, Jacob Olusegun Olusanya, Sok King Ong, Doris V Ortega-Altamirano, Alberto Ortiz, Sergej M Ostojic, Adrian Otoiu, Abdu Oumer, Alicia Padron-Monedero, Jagadish Rao Padubidri, Adrian Pana, Songhomitra Panda-Jonas, Anamika Pandey, Seithikurippu R Pandi-Perumal, Paraskevi Papadopoulou, Shahina Pardhan, Maja Pasovic, Jay Patel, Aslam Ramjan Pathan, Deepak Paudel, Shrikant Pawar, Veincent Christian Filipino Pepito, Gavin Pereira, Marcos Pereira, Norberto Perico, Simone Perna, Ionela-Roxana Petcu, Fanny Emily Petermann-Rocha, Zahra Zahid Piracha, Nishad Plakkal, Naeimeh Pourtaheri, Amir Radfar, Venkatraman Radhakrishnan, Catalina Raggi, Pankaja Raghav, Fakher Rahim, Vafa Rahimi-Movaghar, Azizur Rahman, Md Mosfequr Rahman, Md Obaidur Rahman, Mosiur Rahman, Muhammad Aziz Rahman, Amir Masoud Rahmani, Vahid Rahmanian, Setyaningrum Rahmawaty, Rajesh Kumar Rai, Ivano Raimondo, Sathish Rajaa, Prashant Rajput, Pradhum Ram, Shakthi Kumaran Ramasamy, Sheena Ramazanu, Chythra R Rao, Indu Ramachandra Rao, Sowmya J Rao, Drona Prakash Rasali, Ahmed Mustafa Rashid, Mohammad-Mahdi Rashidi, Zubair Ahmed Ratan, Salman Rawaf, Lal Rawal, Elrashdy M Moustafa Mohamed Redwan, Giuseppe Remuzzi, Kannan RR Rengasamy, Andre M N Renzaho, Malihe Rezaee, Nazila Rezaei, Mohsen Rezaeian, Abanoub Riad, Jennifer Rickard, Alina Rodriguez, Jefferson Antonio Buendia Rodriguez, Leonardo Roever, Peter Rohloff, Bedanta Roy, Godfrey M Rwegerera, Chandan S N, Aly M A Saad, Maha Mohamed Saber-Ayad, Siamak Sabour, Mamta Sachdeva Dhingra, Basema Ahmad Saddik, Erfan Sadeghi, Malihe Sadeghi, Saeid Sadeghian, Umar Saeed, Sahar Saeedi Moghaddam, Sher Zaman Safi, Fatemeh Saheb Sharif-Askari, Amirhossein Sahebkar, Harihar Sahoo, Soumya Swaroop Sahoo, Mirza Rizwan Sajid, Marwa Rashad Salem, Abdallah M Samy, Juan Sanabria, Rama Krishna Sanjeev, Senthilkumar Sankararaman, Itamar S Santos, Milena M Santric-Milicevic, Sivan Yegnanarayana Iyer Saraswathy, Saman Sargazi, Yaser Sarikhani, Maheswar Satpathy, Monika Sawhney, Ganesh Kumar Saya, Abu Sayeed, Nikolaos Scarmeas, Markus P Schlaich, Rachel D Schneider, Aletta Elisabeth Schutte, Subramanian Senthilkumaran, Sadaf G Sepanlou, Dragos Serban, Allen Seylani, Mahan Shafie, Pritik A Shah, Ataollah Shahbandi, Masood Ali Shaikh, Adisu Tafari T Shama, Mehran Shams-Beyranvand, Mohd Shanawaz, Mequannent Melaku Sharew, Pavanchand H Shetty, Rahman Shiri, Velizar Shivarov, Seyed Afshin Shorofi, Kerem Shuval, Migbar Mekonnen Sibhat, Luís Manuel Lopes Rodrigues Silva, Jasvinder A Singh, Narinder Pal Singh, Paramdeep Singh, Surjit Singh, Anna Aleksandrovna Skryabina, Amanda E Smith, Yonatan Solomon, Yi Song, Reed J D Sorensen, Jeffrey D Stanaway, Mu'awiyyah Babale Sufiyan, Muhammad Suleman, Jing Sun, Dev Ram Sunuwar, Mindy D Szeto, Rafael Tabarés-Seisdedos, Seyed-Amir Tabatabaeizadeh, Shima Tabatabai, Moslem Taheri Soodejani, Jacques Lukenze JL Tamuzi, Ker-Kan Tan, Ingan Ukur Tarigan, Zerihun Tariku, Md Tariqujjaman, Elvis Enowbeyang Tarkang, Nathan Y Tat, Birhan Tsegaw Taye, Heather Jean Taylor, Yibekal Manaye Tefera, Arash Tehrani-Banihashemi, Mohamad-Hani Temsah, Masayuki Teramoto, Pugazhenthan Thangaraju, Rekha Thapar, Arulmani Thiyagarajan, Amanda G Thrift, Ales Tichopad, Jansje Henny Vera Ticoalu, Tala Tillawi, Tenaw Yimer Tiruye, Marcello Tonelli, Roman Topor-Madry, Mathilde Touvier, Marcos Roberto Tovani-Palone, Mai Thi Ngoc Tran, Sana Ullah, Eduardo A Undurraga, Bhaskaran Unnikrishnan, Tolassa Wakayo Ushula, Seyed Mohammad Vahabi, Alireza Vakilian, Sahel Valadan Tahbaz, Rohollah Valizadeh, Jef Van den Eynde, Shoban Babu Varthya, Tommi Juhani Vasankari, Narayanaswamy Venketasubramanian, Madhur Verma, Massimiliano Veroux, Dominique Vervoort, Vasily Vlassov, Stein Emil Vollset, Rade Vukovic, Yasir Waheed, Cong Wang, Fang Wang, Molla Mesele Wassie, Kosala Gayan Weerakoon, Melissa Y Wei, Andrea Werdecker, Nuwan Darshana Wickramasinghe, Asrat Arja Wolde, Gedif Ashebir Wubetie, Ratna Dwi Wulandari, Rongbin Xu, Suowen Xu, Xiaoyue Xu, Lalit Yadav, Kazumasa Yamagishi, Lin Yang, Yuichiro Yano, Sanni Yaya, Fereshteh Yazdanpanah, Sisay Shewasinad Yehualashet, Arzu Yiğit, Vahit Yiğit, Dong Keon Yon, Chuanhua Yu, Chun-Wei Yuan, Giulia Zamagni, Sojib Bin Zaman, Aurora Zanghì, Moein Zangiabadian, Iman Zare, Michael Zastrozhin, Bethany Zigler, Mohammad Zoladl, Zhiyong Zou, Nicholas J Kassebaum, Robert C Reiner

## Abstract

**Background:**

The six global nutrition targets (GNTs) related to low birthweight, exclusive breastfeeding, child growth (ie, wasting, stunting, and overweight), and anaemia among females of reproductive age were chosen by the World Health Assembly in 2012 as key indicators of maternal and child health, but there has yet to be a comprehensive report on progress for the period 2012 to 2021. We aimed to evaluate levels, trends, and observed-to-expected progress in prevalence and attributable burden from 2012 to 2021, with prevalence projections to 2050, in 204 countries and territories.

**Methods:**

The prevalence and attributable burden of each target indicator were estimated by age group, sex, and year in 204 countries and territories from 2012 to 2021 in the Global Burden of Diseases, Injuries, and Risk Factors Study (GBD) 2021, the most comprehensive assessment of causes of death, disability, and risk factors to date. Country-specific relative performance to date was evaluated with a Bayesian meta-regression model that compares prevalence to expected values based on Socio-demographic Index (SDI), a composite indicator of societal development status. Target progress was forecasted from 2021 up to 2050 by modelling past trends with meta-regression using a combination of key quantities and then extrapolating future projections of those quantities.

**Findings:**

In 2021, a few countries had already met some of the GNTs: five for exclusive breastfeeding, four for stunting, 96 for child wasting, and three for child overweight, and none met the target for low birthweight or anaemia in females of reproductive age. Since 2012, the annualised rates of change (ARC) in the prevalence of child overweight increased in 201 countries and territories and ARC in the prevalence of anaemia in females of reproductive age decreased considerably in 26 countries. Between 2012 and 2021, SDI was strongly associated with indicator prevalence, apart from exclusive breastfeeding (|*r*-|=0·46–0·86). Many countries in sub-Saharan Africa had a decrease in the prevalence of multiple indicators that was more rapid than expected on the basis of SDI (the differences between observed and expected ARCs for child stunting and wasting were –0·5% and –1·3%, respectively). The ARC in the attributable burden of low birthweight, child stunting, and child wasting decreased faster than the ARC of the prevalence for each in most low-income and middle-income countries. In 2030, we project that 94 countries will meet one of the six targets, 21 countries will meet two targets, and 89 countries will not meet any targets. We project that seven countries will meet the target for exclusive breastfeeding, 28 for child stunting, and 101 for child wasting, and no countries will meet the targets for low birthweight, child overweight, and anaemia. In 2050, we project that seven additional countries will meet the target for exclusive breastfeeding, five for low birthweight, 96 for child stunting, nine for child wasting, and one for child overweight, and no countries are projected to meet the anaemia target.

**Interpretation:**

Based on current levels and past trends, few GNTs will be met by 2030. Major reductions in attributable burden for exclusive breastfeeding and anthropometric indicators should be recognised as huge scientific and policy successes, but the comparative lack of progress in reducing the prevalence of each, along with stagnant anaemia in women of reproductive age and widespread increases in child overweight, suggests a tenuous status quo. Continued investment in preventive and treatment efforts for acute childhood illness is crucial to prevent backsliding. Parallel development of effective treatments, along with commitment to multisectoral, long-term policies to address the determinants and causes of suboptimal nutrition, are sorely needed to gain ground.

**Funding:**

Bill & Melinda Gates Foundation.

## Introduction

Maternal, neonatal, and child health has historically served as a barometer for health system performance. Suboptimal nutrition status is one of the leading contributors to death and disability in these populations.^1^ Growing recognition of the links between maternal health and child health has led to the promotion of policies and partnerships aimed at addressing nutrition as a global health priority. In 2012, the 65th World Health Assembly endorsed a comprehensive plan to improve maternal and child nutrition and established the global nutrition targets (GNTs).^2^ The GNTs were a call to action for governments and policy makers to prioritise change in six inter-related nutrition indicators—specifically, low birthweight, exclusive breastfeeding, child stunting, child wasting, child overweight, and anaemia in females of reproductive age—with a deadline of the year 2025. In 2017, a proposed extension of the GNTs to 2030 was disseminated by WHO;^3^ these extended targets are the focus of this Article (with the exception of child overweight, for which the 2012 target definition was used). Reaching the GNTs by 2030 is a key component of the UN's Sustainable Development Goal (SDG) to eliminate hunger by 2030. As the year 2030 approaches, measuring the progress that has been made towards these targets and where trends are moving in the wrong direction is vital.

**Research in context**

**Evidence before this study**

Several previous studies have estimated trends in the prevalence of one or more global nutrition target (GNT) indicators since 2012. We searched PubMed using the terms “global nutrition target” AND (“progress” OR “prevalence” OR “trends”) for publications between Jan 1, 2016, and June 1, 2022, with no language restrictions, and identified 17 publications, six of which estimated the trend in prevalence of GNT indicators over time in multiple countries and included at least two data sources per country after 2012. The Local Burden of Disease Study series estimated the subnational prevalence of exclusive breastfeeding in the first 6 months of life, anaemia in females of reproductive age, and stunting, wasting, and overweight in children younger than 5 years in low-income and middle-income countries (LMICs) using high-resolution Bayesian model-based geostatistics from 2000 to 2018. Many such studies also modelled the probability of attaining corresponding GNTs by 2025 and 2030. However, estimates and projections are not available for high-income countries where exclusive breastfeeding, child overweight, and anaemia are also concerns. Another study analysed data from Demographic and Health Surveys (DHS) with Bayesian linear regression models to estimate the national trends and projections of the prevalence of anaemia in females of reproductive age from 2000 to 2025 in 15 LMICs. Another study used Bayesian hierarchical mixture models to estimate the prevalence of anaemia by severity in females of reproductive age and children from 2000 to 2019 in 133 countries. Whether trends in the prevalence of each GNT indicator are uniformly related to trends in burden across different countries is unclear.

**Added value of this study**

To our knowledge, no systematic analyses of GNT indicator prevalence trends and change in societal development have been done to date. We estimated country-level prevalence for each of the GNT indicators, evaluated their historical patterns, and used a Bayesian cascading spline model to produce future projections of each to the extended target year of 2030 and annually up to 2050. This study used all accessible data sources up to 2021 and modelled indicators, covariates, and populations systematically using the standardised and rigorous Global Burden of Disease methods. We predicted the future prevalence, borrowing strength across locations, ensuring the data for the smallest locations are reflected in final assessments of levels and trends. Forecasting models featured Socio-demographic Index forecasts as a covariate, which accounted for the effects of the COVID-19 pandemic on income and education.

**Implications of all the available evidence**

Between 2012 and 2021, progress in meeting the global nutrition targets has been variable and largely too slow across indicators to meet the 2030 deadline. The policies of the recent past have not done enough to produce the desired changes and should be investigated to determine whether adaptation, substitution, or wider adoption would yield improved progress in changing indicator prevalence. Barring future reductions in the severity of low birthweight, stunting, and wasting, or declines in the linked causes of death and disability, the future attributable burden of these indicators will decline only gradually. If overweight in children commonly persists into adulthood, then the rising global prevalence of child overweight portends future increases in non-communicable disease burden in adults.

The GNT indicators influence death and disability before birth and throughout the life course. Several studies have previously measured progress towards the GNTs in low-income and middle-income countries (LMICs). The Local Burden of Disease study series estimated the subnational prevalence of exclusive breastfeeding in the first 6 months of life and stunting, wasting, and overweight in children younger than 5 years and modelled the probability of attaining the original GNTs by 2025.^4^, ^5^, ^6^, ^7^ To our knowledge, prevalence estimates and projections to 2030 have not been produced for high-income countries, where increasing prevalence of child overweight and low prevalence of exclusive breastfeeding are concerns. Furthermore, recent estimates of country-level progress in reducing the prevalence of low birthweight and anaemia in females of reproductive age are lacking.^8^, ^9^ One study reported GNT indicator prevalence from 2012 to 2017 for the states of India and made projections to 2030 using The Global Burden of Diseases, Injuries, and Risk Factors Study (GBD) 2017 estimates.^10^ To date, no study has produced GNT indicator forecasts that account for the effects of the COVID-19 pandemic. We expect that population-level nutrition status improves with development (eg, increasing educational attainment, income growth, and declining fertility); yet, to our knowledge, no systematic analyses of GNT indicator progress and development have been done to date. These literature gaps indicate the need for comprehensive analyses of country-level prevalence trends since 2012 for all six indicators and sound projections of their future prevalences.

In this study, completed as part of the GBD Study 2021, we describe global and country-level or territory-level progress on each target from 2012 to 2021, and forecast progress beyond the 2030 deadline to 2050. We describe the change in prevalence of each indicator from 2012 to 2021 in 204 countries and territories and compare these with the change in corresponding attributable burden. Then we compare the change in indicator prevalence with the change that would be expected on the basis of societal development changes in the period. And finally, we model the future prevalences of the indicators in these countries from 2022 to 2050 and identify where projected indicator prevalence will reach targets by 2030, after 2030, or by 2050.

## Methods

### Overview

Prevalence and attributable burden for each of the GNT indicators (table 1) were estimated for countries and territories at the most granular level of location, age, sex, and year for 1990 to 2021. Prevalence and burden were not estimated in disputed, non-sovereign, or low-population locations that are not modelled in the GBD framework, including western Sahara, French Guiana, and Svalbard. This study is compliant with GATHER.^46^ This manuscript was produced as part of the GBD Collaborator Network and in accordance with the GBD Protocol.

**Table 1:** Global nutrition target indicator 2017 extension definitions, demographics, metrics, health outcomes, and 2012 burden

	**Definition**	**Demographic group**	**Target metric**	**Health outcomes to be alleviated by reaching target, by age group**	2012 global burden in attributable DALYs (millions)*
Low birthweight	Birthweight <2500 g irrespective of gestational age^11^	Both sexes, birth	Prevalence: reduce by 30%	Neonatal (age <28 days): sepsis, jaundice, and mortality;^12^, ^13^, ^14^ post-neonatal (age 1–12 months): growth deficits, infectious morbidity, and mortality;^15^, ^16^, ^17^ and adult (age ≥18 years): chronic disease risk	177
Exclusive breastfeeding	Giving infants only breastmilk^18^, ^19^	Both sexes, <6 months	Prevalence: increase to ≥70%	Age <5 years: morbidity and mortality from diarrhoea, lower respiratory infection, and other infectious diseases; high BMI^20^, ^21^, ^22^, ^23^, ^24^, ^25^	17·7
Child stunting	Height-for-age Z score <–2^26^	Both sexes, <5 years	Number: reduce by 50%	Age <5 years: morbidity and mortality from diarrhoea, lower respiratory infection, and other infectious diseases;^27^, ^28^, ^29^ cognitive development and school performance^30^, ^31^, ^32^, ^33^	58·2
Child wasting	Weight-for-height Z score <–2^26^	Both sexes, <5 years	Prevalence: reduce to <3%	Age <5 years: morbidity and mortality from diarrhoea, lower respiratory infection, and other infectious diseases^27^, ^28^, ^29^	77·0
Child overweight†	BMI >IOTF age-specific and sex-specific value^34^, ^35^	Both sexes, 2–4 years	Prevalence: no increase	Age <5 years: asthma; adolescents and adults (age ≥18 years): high BMI,^36^ cardiometabolic diseases, and cancer^27^, ^36^, ^37^, ^38^, ^39^	0·100
Anaemia	Red blood cell count or haemoglobin <WHO thresholds‡^40^	Females, 15–49 years	Prevalence: reduce by 50%	Age 15–49 years: lost energy and physical capacity,^41^ maternal morbidity and mortality;^42^, ^43^ birth: stillbirth, low birthweight, and short gestation births^44^, ^45^	17·3

DALYs=disability-adjusted life-years. GBD=Global Burden of Diseases, Injuries, and Risk Factors study. IOTF=International Obesity Task Force.

* Global burden (with counts given to three significant figures) in millions of attributable DALYs in specified demographic, and in neonates for low birthweight.

† 2012 overweight target was used in this study because GBD uses the IOTF definition rather than weight-for-height Z score-based definition for child overweight, which precludes direct comparison with absolute prevalences published elsewhere; however, trends might be similar.

‡ Mild anaemia in non-pregnant women is haemoglobin concentration of 110–119 g/L, and in pregnant women is haemoglobin concentration of 100–109 g/L.

### Definitions

The definitions, GBD-modelled demographic, target metric, and associated health outcomes for the GNT indicators are listed in table 1 with additional details in appendix 1 (pp 5–7). For consistency with the other indicator definitions, we refer to the prevalence of low birthweight, which is equivalent to the cumulative incidence of low birthweight among all livebirths that year. Overweight is modelled in GBD for children aged 2–4 years on the basis of International Obesity Task Force (IOTF) standards, a range of age-specific and sex-specific BMI cutoff points from a pooled analysis of multi-country longitudinal cohorts whose centile curves pass through BMI of 25 kg/m^2^ at age 18 years.^35^ Hence, for the purpose of this analysis, we used the 2012 GNT extension definition of the child overweight indicator, rather than the 2017 GNT extension definition, because the use of IOTF standards rather than weight-for-height Z score definition for child overweight precludes direct comparison with absolute prevalences published elsewhere. However, trends might be similar.

The Socio-demographic Index (SDI) is a summary indicator created to reflect the background social and economic conditions that shape health outcomes in each location. SDI is calculated as the geometric mean of 0 to 100 indices of total fertility rate in individuals younger than 25 years, mean educational attainment for those age 15 years or older, and lag-distributed income per capita.^47^ An SDI of 0 indicates the point at which decreasing each component does not worsen health outcomes, and an SDI of 100 indicates the level at which increasing each component does not improve health outcomes.

### Summary of estimation approaches for GNT indicators

Prevalence data for each indicator were collected from population-representative surveys, administrative data sources, and published scientific literature. More details on the data-seeking approach for each indicator, including data source maps, are in appendix 1 (pp 7–14). Data availability and sources for each indicator are accessible from the GBD input data tool and have been summarised by GBD region (appendix 1 p 48). Data processing was required for each indicator (eg, standardising data to match reference definition, age-sex splitting, and haemoglobin altitude adjustment), details of which are in appendix 1 (pp 8–14).

### Prevalence

Modelling the prevalence of each GNT indicator involved fitting spatiotemporal Gaussian process regression (ST-GPR) models, with unique parameterisation, covariate selection, and modelling steps for each indicator.^48^, ^49^, ^50^, ^51^, ^52^ Several multistep modelling approaches were tested, and an inclusive set of model covariates (panel) and parameterisations were fit before model optimisation and selection based on in-sample and out-of-sample predictive validity. Birthweight, height-to-age Z score (ie, for stunting), weight-for-height Z score (ie, for wasting), and haemoglobin were modelled as continuous distributions using an ensemble of parametric distributions (eg, gamma, mirror gamma, and Weibull), and prevalence was estimated by integration of these modelled distributions in each location, year, and age group. Prevalence of exclusive breastfeeding in infants younger than 6 months and overweight in children aged 2–4 years was modelled completely in ST-GPR. We propagated all sources of uncertainty into subsequent modelling and aggregation steps by drawing 1000 samples from the posterior distribution of each estimation step for each age-sex-location-year. We report mean and 95% uncertainty intervals (UIs) for all prevalence estimates. Details for each indicator's estimation approach have been published previously and are in appendix 1 (pp 16–32).

Panel **Covariates used for indicator modelling**

**Low birthweight**

• Purpose: imputation for missing birthweights
• Covariates: urbanicity, sex, birthweight recorded on health card, birth order, maternal education, paternal education, child age, child weight, child height, mother's age at birth, mother's weight, shared toilet facility, and household water treated

**Exclusive breastfeeding**

• Purpose: prediction in ST-GPR
• Covariates: SDI, SEV unsafe water, total fertility rate, maternal education, antenatal care (four or more visits), HIV mortality in females of reproductive age, high BMI in females of reproductive age, and underweight in females of reproductive age

**Stunting and wasting**

• Purpose: prediction in ST-GPR
• Covariates: SDI and logit-transformed proportion of households with improved sanitation

**Overweight**

• Purpose: prediction in ST-GPR
• Covariates: 10-year lag-distributed energy per capita, proportion of the population living in urban areas, SDI, lag-distributed income per capita, educational attainment (years) per capita, proportion of the population working in agriculture, grams of sugar adjusted for energy per capita, grams of sugar not adjusted for energy per capita, and number of two-wheeled or four-wheeled vehicles per capita

**Anaemia**

• Purpose: prediction in ST-GPR
• Covariates: age-specific fertility rate, HIV prevalence, SEV for child underweight, SEV for child wasting, malaria incidence, haemoglobin C trait, haemoglobin S trait, SDI, SEV for impaired kidney function, HAQI, GDP per capita, modern contraception prevalence, and 50th percentile of haemoglobin (pooled across all microdata sources)

GDP=gross domestic product. HAQI=Healthcare Access and Quality Index. SDI=Socio-demographic Index. SEV=summary exposure value. ST-GPR=spatiotemporal Gaussian process regression.

### Attributable burden

The GBD 2021 estimation of attributable burden followed the general framework established for comparative risk assessment used in GBD since 2002. Detailed methods on the six key steps of comparative risk assessment are available in previous publications.^48^, ^51^ All GNT indicators except for anaemia are risk factors in GBD. Population attributable fractions (PAFs) were calculated separately for each risk factor (ie, indicator) and outcome pair at each age-sex-location-year. The attributable burden for each indicator, except for anaemia, was calculated as the sum of the cause-specific years of life lost (YLLs) and years lived with disability (YLDs) to produce DALYs, which were multiplied by the PAF for the indicator, and summed with the attributable DALYs across all causes related to that indicator (appendix 1 pp 32–34). Attributable burden was divided by the relevant population in each country to calculate the rate.

As in earlier GBD rounds, we summarised exposure distributions for indicators using the summary exposure value (SEV). SEV is the relative risk-weighted prevalence of indicator exposure, a univariate population measure of risk-weighted exposure that is 0 when no excess risk exists and 1 when the population is at the highest level of risk (more details are in appendix 1 [pp 33–34]).

### Anaemia burden

Anaemia is considered an impairment in GBD—ie, a condition or specific domain of functional health loss that is spread across many GBD causes as sequelae. Therefore, in this Article, we present total anaemia burden in the form of YLDs rather than YLDs attributed to distinct sequelae (which are estimated in GBD).

### Annualised rate of change and epidemiological transition

We performed several analyses to explore geographical, demographic, and temporal trends in the indicators. Initially, we calculated the annualised rate of change (ARC) between 2012 and 2021 for the prevalence of each indicator and its attributable burden (ie, rate) in each country. ARC is calculated as

$$\frac{\ln\left(\frac{{\text{value}}_{2}}{{\text{value}}_{1}}\right)}{{\text{year}}_{2}-{\text{year}}_{1}},$$

where value_1_ and value_2_ are the quantity of interest (ie, prevalence or attributable burden) in the first year (year_1_) and later year (year_2_). We calculated the mean ARC and uncertainty intervals (2·5th and 97·5th percentiles) from 1000 posterior draw-level ARCs for each country, age, and sex combination. We considered a country's ARC to be substantial if the mean and at least 80% of model posterior draw-level ARCs were consistent in direction.

Next, to understand how socioeconomic development relates to differential progress across indicators, we compared prevalence with expected values for each country based on the association between SDI and prevalence from 1990 to 2021 using a meta-regression—Bayesian, regularised, trimmed (MR-BRT) approach (described in appendix 1 [pp 36–37]). Briefly, such models synthesise results from different locations and years, incorporate the uncertainty in the dependent variable (ie, mean prevalence estimate), and include random effects that permit variation in the true effect (ie, the association between SDI and prevalence). Model-predicted expected prevalence values based on SDI do not include uncertainty. We subtracted expected ARC from observed ARC from 2012 to 2021 to identify countries with change in indicator prevalence larger or smaller than expected on the basis of each country's level of socioeconomic development.

### Predicting future prevalence from forecasted SEVs

To forecast future prevalence, we modelled the global associations between age-sex-indicator-matched SEVs and indicator prevalence in a MR-BRT model with SDI as a linear fixed effect. SEVs are not modelled for impairments (eg, anaemia) in GBD; however, iron deficiency is the primary risk factor for anaemia and therefore we modelled the global associations between age-sex-matched iron deficiency SEVs and anaemia prevalence in females. We then fit a cascading random spline model to optimise location-year-specific fit of the relational model^53^ (appendix 1 pp 42–44). The cascading splines approach uses prespecified information about group membership to create priors that borrow information at each level of the cascade and enable groups to deviate from the global trend. To optimise model configuration, we trained models on historical estimates from 1990 to 2014, used each model version to predict prevalence from SEVs for 2015 to 2021, and calculated the out-of-sample root-mean-square error. We then used the parameter values from the best model (ie, the lowest root-mean-square error) to fit the full set of SEV and prevalence estimates from 1990 to 2021, and input corresponding SEV forecasts and SDI projections,^54^ to generate indicator-specific prevalence projections from 2022 to 2050. SDI forecasts were calculated from forecasts of the three SDI component measures: educational attainment, total fertility rate in females younger than 25 years, and lag-distributed income. Income and education projections were revised to adjust for the effects of the COVID-19 pandemic (eg, school closures and short-term economic gross domestic product effects). Details on the methods used to predict future SEVs, SDI, and prevalence are in appendix 1 (pp 38–42). All analyses were done using R (versions 3.6.3 and 4.2.15).

### Role of the funding source

The funder of the study had no role in study design, data collection, data analysis, data interpretation, or writing of the report.

## Results

### GNT indicator trends from 2012 to 2021

The change in the prevalence of GNT indicators varied considerably by location between 2012 and 2021. Trends in the prevalence of each indicator were not uniformly related to trends in attributable burden across different countries. In 2021, we found that seven countries had already met two of six targets (Georgia, Mongolia, South Korea, Peru, Rwanda, American Samoa, and Puerto Rico), 94 had met one target, and 100 had met no targets.

**Figure 1:**
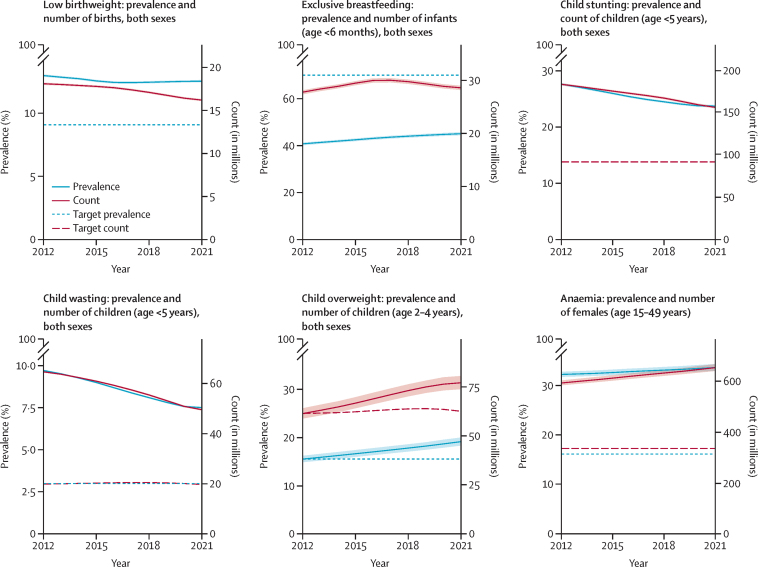
Global nutrition target indicator prevalence and count trajectories, 2012 to 2021 Shaded areas indicate 95% uncertainty intervals.

**Figure 2:**
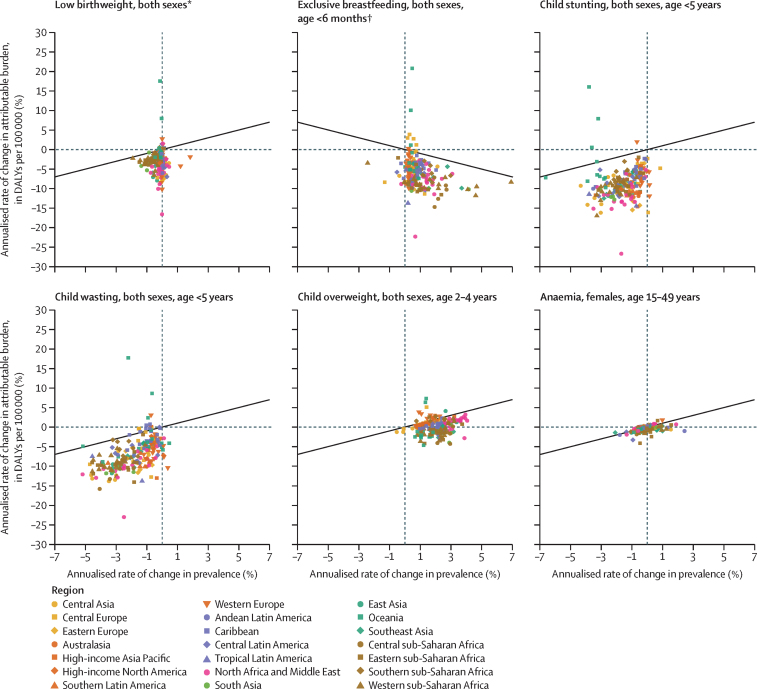
Annual rates of change in prevalence versus attributable burden of global nutrition target indicators, by region, from 2012 to 2021 The solid line in each plot indicates where rates of change are equal for both variables plotted. *Prevalence is measured at birth and the attributable burden is during the neonatal period (age 0–27 days). †Because breastfeeding is protective, the solid line has a negative relationship.

Between 2012 and 2021, the global prevalence of low birthweight in newborns declined from 12·94% (95% UI 12·94 to 13·02) to 12·49% (12·41 to 12·58), 3·4% more than the target (figure 1, table 2). In 2021, no country met the GNT for 30% reduction in the prevalence of low birthweight. From 2012 to 2021, the ARC in the prevalence of low birthweight was lowest in Mali (–1·9% [–2·9 to –1·1]) and highest in Ireland (1·8% [0·6 to 3·0]; appendix 2 pp 5–6, 105–133). In all but 14 countries, including most of those in east Asia and south Asia, the ARC in attributable burden (in DALYs per 100 000) due to low birthweight in neonates was less than the ARC in the prevalence of low birthweight in newborns (figure 2; appendix 2 pp 36–56, 102–130).

**Table 2:** Global, regional, and country prevalence of malnutrition indicators, 2012, 2021, and projected for 2030

		**Low birthweight both sexes, birth**	**Exclusive breastfeeding, both sexes, age <6 months**	**Child stunting, both sexes, age <5 years**	**Child wasting, both sexes, age <5 years**	**Child overweight, both sexes, age 2–4 years**	**Anaemia, females, age 15–49 years**
**Global**							
**2012**		**12·94% (12·85–13·02)**	**40·74% (40·15–41·24)**	**27·54% (27·34–27·72)**	**9·71% (9·67–9·76)**	**15·50% (14·87–16·18)**	**32·30% (31·82–32·84)**
**2021**		**12·49% (12·41–12·58)**	**45·05% (44·42–45·67)**	**23·66% (23·46–23·86)**	**7·51% (7·46–7·56)**	**19·11% (18·30–19·95)**	**33·69% (33·03–34·39)**
**2030**		**11·88% (11·20–12·60)**	**46·68% (27·38–63·30)**	**21·03% (14·22–24·31)**	**7·47% (6·18–8·69)**	**22·32% (19·27–25·83)**	**32·43% (30·22–34·99)**
**Central Europe, eastern Europe, and central Asia**							
**2012**		**6·33% (6·25–6·41)**	**25·18% (23·49–26·83)**	**14·38% (13·91–14·87)**	**4·29% (4·20–4·39)**	**26·11% (23·59–29·03)**	**26·41% (24·80–28·19)**
**2021**		**6·28% (6·18–6·37)**	**26·91% (25·14–28·66)**	**12·48% (11·99–13·00)**	**3·57% (3·48–3·66)**	**28·78% (25·87–31·82)**	**26·53% (24·93–28·56)**
**2030**		**6·26% (5·94–6·63)**	**28·65% (12·19–49·99)**	**10·63% (6·25–13·54)**	**3·49% (2·61–4·42)**	**31·58% (26·42–37·58)**	**25·65% (22·41–29·06)**
Central Asia							
2012		6·23% (6·04–6·42)	28·57% (26·97–30·21)	17·37% (16·81–17·92)	5·69% (5·56–5·84)	24·53% (22·28–26·91)	42·39% (39·37–46·39)
2021		6·02% (5·83–6·21)	30·08% (27·82–32·79)	13·32% (12·71–13·94)	4·36% (4·23–4·50)	26·60% (23·60–30·06)	41·94% (38·49–46·17)
2030		5·94% (5·70–6·31)	32·70% (11·64–56·11)	10·79% (6·15–14·08)	4·16% (3·19–5·09)	30·63% (23·96–38·63)	39·85% (36·38–43·23)
Armenia							
	2012	8·21% (7·78–8·66)	39·02% (35·97–41·99)	15·18% (14·02–16·29)	4·13% (3·87–4·40)	30·63% (26·29–35·59)	21·29% (18·94–24·18)
	2021	8·55% (8·06–9·05)	41·74% (37·51–45·86)	12·46% (11·28–13·80)	3·82% (3·55–4·09)	33·07% (26·11–40·31)	22·35% (18·96–27·53)
	2030	8·34% (6·80–9·75)	44·34% (16·71–63·42)	10·43%* (5·85–14·24)	3·70% (2·50–5·03)	37·71% (27·32–49·65)	22·06% (17·99–25·80)
	Azerbaijan						
	2012	8·06% (7·52–8·58)	12·18% (9·07–15·60)	17·92% (16·87–18·98)	6·24% (5·94–6·58)	25·30% (20·10–31·15)	36·26% (33·55–39·07)
	2021	7·83% (7·26–8·40)	13·37% (9·67–17·99)	15·98% (14·54–17·38)	5·27% (4·95–5·58)	26·49% (20·09–34·00)	35·40% (31·55–40·41)
	2030	7·71% (6·59–9·86)	14·11% (0·18–39·58)	13·15% (7·43–17·90)	5·33% (3·81–6·77)	29·31% (24·25–35·29)	33·29% (28·24–37·90)
	Georgia						
	2012	9·23% (8·77–9·69)	33·03% (29·33–36·61)	10·88% (9·85–11·96)	1·91% (1·80–2·04)	24·32% (19·19–30·26)	25·41% (23·21–27·71)
	2021	9·45% (8·88–10·02)	33·95% (28·76–39·14)	7·99% (7·08–9·09)	1·26% (1·18–1·35)	23·21% (17·77–29·35)	25·15% (22·25–28·65)
	2030	9·44% (9·03–10·15)	36·48% (18·10–56·96)	6·80%* (3·71–9·11)	1·24%* (0·89–1·62)	25·26% (18·36–35·61)	25·21% (21·71–28·97)
	Kazakhstan						
	2012	6·13% (5·65–6·67)	30·27% (28·12–32·42)	12·87% (11·98–13·73)	5·08% (4·82–5·32)	24·62% (20·57–29·34)	40·48% (34·58–49·19)
	2021	5·97% (5·52–6·46)	34·40% (30·08–38·61)	9·80% (8·77–10·82)	3·85% (3·64–4·06)	26·43% (21·05–32·75)	38·53% (32·29–48·54)
	2030	5·77% (5·05–6·83)	36·66% (11·17–61·17)	7·96% (4·03–11·39)	3·58% (2·53–4·65)	30·60% (23·47–40·77)	35·93% (30·84–41·44)
	Kyrgyzstan						
	2012	5·90% (5·44–6·34)	48·46% (46·56–50·39)	17·11% (16·21–18·01)	3·80% (3·59–4·01)	18·10% (15·25–21·11)	37·65 % (34·98–41·11)
	2021	5·78% (5·37–6·23)	49·46% (45·51–53·10)	14·36% (13·03–15·72)	2·91% (2·72–3·11)	19·72% (15·44–24·37)	36·33% (32·51–41·42)
	2030	5·75% (5·56–6·33)	52·34% (24·75–70·10)	11·73% (6·72–15·34)	2·90%* (1·97–3·91)	19·07% (13·84–30·06)	33·87% (30·19–37·46)
	Mongolia						
	2012	5·57% (5·16–6·00)	56·52% (54·58–58·26)	15·72% (14·62–16·75)	2·24% (2·11–2·36)	21·67% (18·48–25·21)	28·84% (26·44–31·10)
	2021	5·54% (5·09–6·02)	56·74% (54·03–59·53)	12·01% (10·73–13·25)	1·48% (1·39–1·58)	21·50% (17·07–26·51)	27·86% (25·07–30·53)
	2030	5·62% (5·11–6·52)	57·59% (36·45–71·17)	8·99% (4·57–12·65)	1·39%* (0·95–1·89)	25·28% (17·59–37·61)	25·12% (21·56–28·77)
	Tajikistan						
	2012	8·44% (7·87–9·02)	32·90% (31·18–34·76)	28·31% (27·00–29·63)	9·55% (9·19–9·93)	15·97% (13·21–19·06)	35·56% (33·61–37·57)
	2021	8·02% (7·47–8·59)	34·60% (31·04–38·33)	22·48% (20·82–24·14)	7·39% (6·99–7·78)	17·73% (13·56–22·54)	40·91% (36·82–46·40)
	2030	7·77% (7·59–8·23)	36·71% (11·80–60·13)	18·05% (10·79–23·59)	6·61% (4·92–8·16)	22·09% (16·84–28·97)	38·69% (35·00–42·15)
	Turkmenistan						
	2012	4·45% (4·09–4·81)	38·88% (35·78–41·96)	13·95% (13·23–14·71)	6·39% (6·12–6·67)	19·55% (14·21–26·04)	34·51% (29·97–41·03)
	2021	4·36% (4·00–4·75)	47·04% (42·88–50·65)	9·44% (8·65–10·33)	5·21% (4·95–5·47)	21·22% (15·46–27·95)	33·61% (28·84–40·17)
	2030	4·33% (3·90–5·36)	52·47% (22·23–70·42)	7·13% (3·71–10·23)	4·84% (3·56–6·11)	27·64% (18·10–39·37)	31·77% (26·92–36·67)
	Uzbekistan						
	2012	5·02% (4·63–5·41)	21·16% (17·55–25·11)	17·38% (15·98–18·69)	5·71% (5·39–6·06)	29·49% (23·96–35·39)	54·76% (47·51–64·83)
	2021	4·97% (4·59–5·35)	20·55% (15·61–26·28)	12·24% (10·86–13·67)	4·18% (3·89–4·49)	32·59% (25·32–40·20)	52·88% (45·19–63·99)
	2030	5·01% (4·85–5·27)	21·44% (1·00–48·08)	9·89% (5·88–12·98)	3·96% (3·15–4·70)	37·74% (25·73–53·11)	50·71% (45·49–55·56)
**Central Europe**							
2012		7·10% (6·98–7·22)	28·46% (26·82–30·12)	9·65% (9·24–10·11)	3·27% (3·17–3·36)	24·42% (21·90–27·14)	21·98% (20·29–24·09)
2021		7·13% (7·00–7·26)	29·66% (27·77–31·37)	9·01% (8·58–9·47)	2·86% (2·77–2·94)	27·14% (24·26–30·11)	22·17% (20·29–24·58)
2030		7·32% (6·77–7·97)	29·90% (15·27–46·48)	7·95% (4·33–10·57)	2·76%* (1·95–3·65)	29·48% (24·57–34·80)	21·19% (18·22–24·54)
	Albania						
	2012	4·64% (4·28–5·03)	24·18% (19·26–28·99)	24·59% (23·27–25·82)	8·26% (7·78–8·72)	33·62% (28·40–39·16)	27·43% (23·93–32·28)
	2021	4·57% (4·19–4·94)	21·54% (16·27–27·51)	19·53% (18·09–20·95)	5·40% (5·04–5·78)	33·40% (26·81–40·54)	28·83% (24·16–35·47)
	2030	4·50% (4·28–4·83)	22·73% (1·48–46·74)	16·49% (11·35–19·69)	5·08% (3·48–6·85)	35·33% (29·12–40·10)	28·66% (24·73–32·61)
	Bosnia and Herzegovina						
	2012	4·41% (4·03–4·80)	15·55% (12·11–19·40)	10·34% (9·50–11·28)	3·23% (3·03–3·44)	33·62% (28·46–38·63)	23·04% (19·18–28·34)
	2021	4·37% (4·01–4·77)	16·36% (12·31–20·54)	10·08% (9·13–11·17)	3·09% (2·88–3·30)	36·62% (29·88–44·29)	23·52% (19·51–29·63)
	2030	4·36% (3·57–5·16)	16·99% (2·03–43·33)	8·68% (5·23–11·45)	2·78%* (1·75–3·93)	39·80% (33·04–47·18)	22·74% (18·43–26·83)
	Bulgaria						
	2012	9·06% (8·61–9·57)	21·53% (17·00–26·28)	5·50% (4·78–6·29)	3·46% (3·19–3·77)	26·55% (20·07–34·50)	25·11% (21·13–31·10)
	2021	9·08% (8·57–9·61)	22·46% (18·13–26·87)	5·21% (4·52–5·92)	3·19% (2·91–3·48)	28·64% (21·42–36·30)	25·38% (21·13–31·66)
	2030	8·99% (7·77–10·57)	22·39% (3·91–40·13)	4·77% (2·73–6·34)	3·10% (2·13–3·96)	30·18% (23·30–39·66)	24·41% (20·01–29·08)
	Croatia						
	2012	5·28% (4·96–5·60)	17·28% (13·31–21·47)	9·54% (8·56–10·59)	3·37% (3·14–3·62)	26·12% (19·44–33·51)	19·43% (16·10–23·86)
	2021	5·39% (4·97–5·78)	18·47% (14·27–22·78)	9·25% (8·27–10·35)	3·05% (2·84–3·29)	29·50% (22·40–37·53)	19·27% (15·81–23·84)
	2030	5·23% (4·92–5·59)	18·52% (7·39–32·88)	8·11% (3·99–10·88)	2·97%* (2·05–4·01)	32·71% (25·07–41·08)	18·66% (14·50–22·95)
	Czechia						
	2012	7·99% (7·59–8·39)	34·23% (30·24–38·14)	2·69% (2·40–3·01)	2·95% (2·74–3·16)	25·12% (18·97–32·00)	18·81% (15·93–22·55)
	2021	7·95% (7·51–8·45)	34·76% (30·95–38·72)	2·70% (2·43–3·03)	2·77% (2·56–2·99)	27·50% (20·69–34·89)	19·27% (15·96–24·01)
	2030	7·80% (6·92–8·84)	35·04% (29·90–41·21)	2·59% (1·37–3·86)	2·73%* (1·86–3·80)	29·20% (22·51–36·87)	18·45% (14·65–22·45)
	Hungary						
	2012	8·53% (8·09–9·01)	13·41% (10·16–16·92)	9·71% (8·71–10·78)	3·42% (3·19–3·70)	25·63% (19·04–33·02)	20·67% (17·29–25·24)
	2021	8·50% (7·98–9·00)	14·10% (11·03–17·61)	9·17% (8·20–10·30)	2·99% (2·77–3·23)	28·56% (21·24–36·99)	20·50% (16·59–25·73)
	2030	8·39% (7·28–9·74)	14·02% (5·96–26·60)	8·03% (4·10–10·96)	2·93%* (2·03–3·99)	30·67% (23·50–40·24)	19·56% (15·20–23·80)
	Montenegro						
	2012	7·72% (7·18–8·23)	13·32% (10·32–16·73)	10·22% (9·45–11·15)	4·01% (3·75–4·25)	33·82% (28·91–38·38)	21·60% (17·99–26·28)
	2021	7·71% (7·20–8·20)	14·96% (11·25–19·09)	10·28% (9·28–11·45)	3·76% (3·54–3·98)	38·51% (31·75–45·63)	21·67% (18·05–26·54)
	2030	7·57% (6·75–8·70)	15·54% (1·20–46·39)	9·88% (5·45–13·32)	3·67% (2·69–4·59)	42·27% (35·27–50·03)	21·11% (17·16–25·60)
	North Macedonia						
	2012	7·24% (6·80–7·69)	17·52% (13·70–21·60)	8·01% (7·29–8·80)	3·77% (3·54–4·01)	27·52% (23·54–31·78)	20·37% (17·94–22·96)
	2021	7·26% (6·82–7·72)	18·82% (14·49–23·61)	7·26% (6·43–8·14)	3·28% (3·06–3·49)	30·34% (24·39–36·87)	20·35% (17·72–23·09)
	2030	7·12% (6·65–7·59)	19·16% (0·68–50·15)	6·29% (3·02–9·10)	3·20% (2·08–4·47)	32·27% (25·50–39·04)	19·88% (15·98–23·55)
	Poland						
	2012	6·17% (6·03–6·29)	37·58% (33·44–41·61)	9·04% (8·07–10·08)	3·06% (2·83–3·29)	23·02% (17·27–30·27)	22·34% (18·37–27·95)
	2021	6·21% (6·07–6·36)	38·58% (34·50–42·63)	8·60% (7·68–9·62)	2·67% (2·47–2·88)	26·11% (19·48–33·21)	22·72% (18·45–29·12)
	2030	6·45% (5·92–6·91)	39·28% (19·10–55·26)	7·41% (3·72–10·33)	2·60%* (1·79–3·50)	28·76% (21·91–35·79)	21·62% (17·45–26·29)
	Romania						
	2012	8·31% (7·85–8·78)	23·45% (18·23–29·03)	13·93% (12·58–15·30)	2·43% (2·25–2·63)	21·69% (15·88–28·43)	22·87% (18·95–28·22)
	2021	8·22% (7·77–8·68)	25·13% (19·61–31·15)	12·63% (11·19–14·01)	2·18% (2·00–2·34)	24·62% (18·74–31·80)	22·83% (18·76–28·60)
	2030	8·81% (7·50–10·46)	26·08% (11·60–47·16)	11·12% (6·02–14·71)	2·03%* (1·42–2·75)	27·54% (21·03–35·48)	21·63% (17·26–25·76)
	Serbia						
	2012	6·50% (6·09–6·90)	14·88% (12·35–17·70)	9·04% (8·31–9·80)	3·94% (3·69–4·19)	25·85% (22·57–29·36)	22·13% (18·73–26·67)
	2021	6·62% (6·18–7·07)	16·27% (12·63–20·69)	9·76% (8·84–10·59)	3·42% (3·22–3·63)	28·52% (22·73–34·33)	22·04% (18·26–27·11)
	2030	6·59% (5·57–7·97)	16·95% (2·58–43·13)	8·27% (4·51–11·17)	3·40% (2·38–4·45)	29·69% (23·82–34·82)	21·19% (17·14–25·58)
	Slovakia						
	2012	7·88% (7·41–8·37)	50·38% (47·34–53·28)	10·16% (9·15–11·29)	3·69% (3·44–3·94)	20·45% (14·87–27·02)	20·86% (17·48–26·03)
	2021	7·92% (7·41–8·45)	51·03% (48·12–53·85)	9·79% (8·78–10·86)	3·28% (3·05–3·52)	22·42% (16·99–29·13)	21·17% (17·50–26·34)
	2030	7·93% (6·07–10·43)	51·25% (32·89–62·41)	8·81% (4·59–12·28)	3·22% (2·20–4·27)	24·12% (17·77–30·83)	20·50% (15·66–24·73)
	Slovenia						
	2012	6·54% (6·18–6·92)	33·67% (27·38–39·63)	8·47% (7·57–9·37)	2·88% (2·67–3·09)	27·38% (20·57–34·59)	17·67% (14·71–21·36)
	2021	6·53% (6·04–6·97)	35·50% (29·49–41·71)	8·26% (7·38–9·21)	2·60% (2·41–2·81)	30·46% (23·37–38·57)	17·55% (14·50–21·69)
	2030	6·31% (5·08–7·92)	35·36% (8·95–55·46)	7·29% (3·74–9·90)	2·56%* (1·75–3·55)	33·21% (26·41–42·52)	16·39% (12·78–20·17)
**Eastern Europe**							
2012		6·07% (5·99–6·15)	21·07% (17·58–24·47)	14·52% (13·62–15·48)	3·79% (3·61–3·98)	28·07% (23·05–34·05)	21·67% (19·15–24·99)
2021		6·08% (6·01–6·16)	22·63% (19·09–26·28)	13·40% (12·48–14·41)	3·28% (3·11–3·44)	31·18% (25·17–37·51)	20·98% (18·61–24·13)
2030		6·03% (5·67–6·45)	23·30% (8·94–45·29)	11·96% (7·36–15·19)	3·14% (2·31–4·05)	33·83% (28·12–40·09)	19·92% (16·03–24·14)
	Belarus						
	2012	4·59% (4·22–4·97)	13·59% (10·64–16·77)	4·65% (4·19–5·16)	2·17% (2·01–2·33)	18·17% (14·15–23·07)	21·09% (17·79–24·94)
	2021	4·59% (4·21–4·99)	14·42% (10·83–18·55)	4·10% (3·63–4·62)	1·82% (1·69–1·97)	21·65% (16·24–28·16)	20·12% (17·12–24·25)
	2030	4·65% (4·13–5·33)	15·41% (3·33–37·44)	3·79% (1·90–5·65)	1·77%* (1·23–2·39)	24·08% (17·32–30·99)	19·22% (14·63–23·56)
	Estonia						
	2012	4·74% (4·41–5·07)	24·79% (19·76–29·57)	7·10% (6·23–8·04)	2·05% (1·89–2·23)	21·36% (15·42–27·87)	19·15% (16·41–22·53)
	2021	4·80% (4·41–5·18)	26·31% (21·51–31·32)	6·79% (6·05–7·63)	1·93% (1·77–2·10)	23·90% (17·32–31·02)	18·73% (15·79–22·27)
	2030	4·90% (4·35–5·71)	26·76% (8·47–46·54)	5·60% (2·70–8·19)	1·87%* (1·35–2·41)	26·24% (20·99–32·34)	17·63% (13·62–22·09)
	Latvia						
	2012	5·15% (4·76–5·55)	34·45% (27·92–40·50)	8·27% (7·34–9·38)	2·51% (2·32–2·72)	17·28% (12·36–22·91)	21·54% (18·31–25·55)
	2021	5·16% (4·74–5·56)	36·27% (30·12–42·41)	7·58% (6·67–8·58)	2·21% (2·04–2·41)	19·23% (13·82–25·25)	20·66% (17·58–24·72)
	2030	5·22% (4·78–5·82)	36·94% (8·45–58·43)	6·16%* (3·07–8·79)	2·15%* (1·51–2·80)	21·16% (16·72–26·41)	19·77% (15·98–24·06)
	Lithuania						
	2012	4·73% (4·40–5·07)	9·03% (6·97–11·18)	7·50% (6·62–8·46)	2·20% (2·03–2·39)	16·45% (11·71–22·05)	21·52% (18·09–25·70)
	2021	4·70% (4·36–5·08)	9·54% (7·48–11·79)	6·90% (5·99–7·82)	1·94% (1·78–2·12)	18·92% (13·89–24·92)	20·47% (17·22–24·38)
	2030	4·53% (3·87–5·35)	9·68% (2·39–17·73)	5·76%* (2·74–8·24)	1·89%* (1·34–2·49)	21·38% (16·18–26·83)	19·95% (15·72–23·89)
	Moldova						
	2012	5·73% (5·36–6·12)	24·84% (20·95–28·69)	7·75% (6·93–8·55)	3·29% (3·07–3·54)	9·06% (6·97–11·62)	25·92% (24·68–27·26)
	2021	5·68% (5·30–6·08)	26·16% (21·44–30·84)	6·56% (5·76–7·46)	2·66% (2·46–2·88)	11·08% (8·21–14·74)	24·79% (22·23–27·65)
	2030	5·69% (5·06–6·42)	26·67% (7·89–51·26)	5·48%* (2·52–7·88)	2·47%* (1·60–3·41)	13·20% (9·13–18·21)	24·20% (19·70–28·02)
	Russia						
	2012	6·58% (6·51–6·64)	23·09% (18·26–27·63)	13·23% (12·01–14·56)	2·66% (2·45–2·88)	32·56% (25·35–40·37)	22·75% (19·19–27·34)
	2021	6·53% (6·46–6·59)	24·42% (19·56–29·11)	12·25% (11·07–13·51)	2·31% (2·13–2·51)	35·07% (27·12–43·06)	21·70% (18·34–26·17)
	2030	6·43% (6·03–6·86)	24·99% (10·24–46·90)	10·85% (6·25–14·60)	2·30%* (1·60–3·07)	37·67% (31·07–45·14)	20·36% (15·86–25·50)
	Ukraine						
	2012	4·66% (4·33–5·00)	15·08% (12·24–18·11)	22·96% (21·52–24·42)	8·55% (8·01–9·11)	16·95% (12·10–22·67)	18·07% (15·91–20·36)
	2021	4·62% (4·29–4·96)	16·14% (12·16–20·30)	22·56% (20·99–24·22)	8·20% (7·66–8·78)	19·60% (14·49–25·90)	18·51% (16·12–20·97)
	2030	4·64% (4·16–5·22)	16·80% (2·35–40·81)	21·83%* (14·90–26·20)	8·17% (6·25–9·97)	20·62% (15·17–27·29)	18·20% (13·53–22·61)
**High income**							
**2012**		**7·61% (7·54–7·68)**	**38·05% (37·13–38·88)**	**3·00% (2·89–3·13)**	**1·22% (1·20–1·24)**	**22·12% (20·75–23·62)**	**11·12% (10·08–12·32)**
**2021**		**7·61% (7·53–7·68)**	**39·09% (37·81–40·34)**	**2·75% (2·63–2·88)**	**1·13% (1·11–1·15)**	**25·26% (23·34–27·17)**	**11·19% (9·97–12·78)**
**2030**		**7·43% (6·93–8·08)**	**39·88% (29·50–47·38)**	**2·52% (1·30–3·57)**	1·12%*(0·69–1·65)	**26·83% (23·45–31·04)**	**10·96% (9·35–12·80)**
Australasia							
2012		6·85% (6·42–7·30)	42·32% (39·13–45·59)	2·00% (1·80–2·23)	0·77% (0·71–0·83)	29·29% (23·98–35·20)	8·98% (7·66–10·60)
2021		6·75% (6·35–7·18)	43·86% (40·09–47·30)	1·88% (1·69–2·09)	0·71% (0·66–0·77)	33·42% (27·12–40·18)	8·83% (7·43–10·52)
2030		6·51% (5·96–7·45)	44·27% (26·49–57·76)	1·76% (0·81–2·72)	0·71%* (0·44–1·05)	35·73% (29·22–43·20)	8·65% (7·14–10·48)
	Australia						
	2012	6·98% (6·48–7·52)	41·91% (38·15–45·78)	1·88% (1·64–2·14)	0·76% (0·69–0·84)	28·39% (21·73–35·40)	8·94% (7·40–10·82)
	2021	6·87% (6·39–7·41)	43·68% (39·37–47·77)	1·79% (1·57–2·04)	0·71% (0·64–0·78)	32·84% (25·39–40·55)	8·85% (7·24–10·87)
	2030	6·64% (6·06–7·61)	44·15% (26·00–57·83)	1·68% (0·78–2·59)	0·70%* (0·44–1·05)	35·07% (28·23–43·09)	8·67% (6·99–10·74)
	New Zealand						
	2012	6·23% (5·91–6·59)	44·41% (40·81–47·88)	2·53% (2·24–2·86)	0·80% (0·72–0·87)	33·42% (26·16–41·27)	9·16% (7·59–10·99)
	2021	6·12% (5·74–6·49)	44·67% (40·68–48·25)	2·33% (2·04–2·61)	0·73% (0·66–0·80)	36·41% (28·39–45·04)	8·72% (7·22–10·41)
	2030	5·88% (4·85–7·30)	44·83% (19·30–59·20)	2·17% (0·96–3·33)	0·72%* (0·44–1·05)	38·99% (30·22–49·18)	8·53% (6·88–10·40)
**High-income Asia Pacific**							
2012		9·47% (9·25–9·67)	40·79% (36·49–44·78)	6·30% (6·20–6·39)	2·33% (2·29–2·37)	14·18% (11·06–17·39)	13·70% (10·82–17·61)
2021		9·38% (9·19–9·59)	42·46% (37·88–46·79)	5·77% (5·62–5·91)	2·13% (2·09–2·17)	17·30% (13·83–21·11)	13·83% (10·52–18·88)
2030		9·10% (8·29–9·97)	43·72% (22·37–56·78)	5·89% (3·47–7·37)	2·22%* (1·44–3·05)	18·36% (14·29–23·90)	13·02% (10·75–15·36)
	Brunei						
	2012	12·42% (11·85–12·99)	47·80% (42·82–52·76)	18·71% (17·56–19·85)	2·93% (2·71–3·15)	6·47% (4·51–8·93)	13·61% (10·98–17·64)
	2021	12·24% (11·60–12·92)	50·19% (44·84–55·07)	17·94% (16·58–19·41)	2·70% (2·49–2·93)	8·36% (5·79–11·51)	13·99% (11·13–19·55)
	2030	11·96% (9·39–15·57)	51·26% (31·64–64·64)	17·12% (12·26–20·95)	2·66%* (1·81–3·60)	9·59% (6·73–12·71)	13·89% (11·36–16·75)
	Japan						
	2012	9·39% (9·26–9·53)	43·89% (37·65–49·31)	7·91% (7·78–8·03)	2·73% (2·69–2·77)	13·59% (9·75–18·09)	15·42% (11·24–21·63)
	2021	9·28% (9·13–9·44)	45·45% (39·16–51·13)	7·59% (7·39–7·78)	2·54% (2·50–2·58)	16·21% (11·57–21·56)	15·80% (10·88–23·67)
	2030	9·11% (8·41–9·87)	46·36% (21·74–60·52)	7·31% (4·37–8·99)	2·54%* (1·66–3·48)	17·11% (12·79–22·79)	14·89% (11·78–18·01)
	Singapore						
	2012	9·32% (8·86–9·81)	29·00% (26·79–31·16)	3·68% (3·25–4·15)	2·67% (2·43–2·94)	11·04% (7·94–14·96)	19·19% (8·19–36·26)
	2021	9·21% (8·65–9·75)	31·08% (26·72–35·40)	3·48% (3·04–3·99)	2·59% (2·32–2·87)	14·74% (10·67–20·25)	18·21% (8·21–34·66)
	2030	8·61% (6·94–10·79)	33·96% (22·16–53·26)	3·32% (1·41–5·17)	2·54%* (1·75–3·46)	16·86% (12·61–21·64)	16·69% (13·18–20·04)
	South Korea						
	2012	9·64% (8·98–10·24)	35·11% (30·63–39·78)	2·62% (2·44–2·81)	1·34 % (1·25–1·45)	15·99% (11·42–21·23)	9·47% (7·72–11·34)
	2021	9·63% (8·98–10·28)	36·37% (31·96–40·91)	1·94% (1·72–2·18)	1·18% (1·09–1·28)	20·13% (14·79–26·32)	9·18% (7·50–11·01)
	2030	9·08% (7·21–11·45)	37·15% (24·60–48·38)	1·86%* (0·76–2·79)	1·18%* (0·70–1·75)	22·54% (17·03–30·39)	8·52% (6·84–10·18)
**High-income North America**							
2012		7·89% (7·80–7·98)	43·34% (42·02–44·64)	2·57% (2·31–2·86)	0·84% (0·79–0·88)	20·33% (17·49–23·64)	11·96% (10·98–13·26)
2021		8·06% (7·95–8·16)	44·26% (41·79–46·79)	2·31% (2·02–2·64)	0·81% (0·77–0·85)	22·60% (18·48–27·15)	11·96% (10·19–14·28)
2030		7·93% (7·41–8·71)	44·89% (37·06–52·72)	2·22% (1·01–3·45)	0·82%* (0·50–1·27)	23·53% (21·53–26·10)	12·13% (9·78–14·58)
	Canada						
	2012	6·00% (5·56–6·42)	28·34% (23·60–32·79)	0·83% (0·74–0·94)	2·21% (2·00–2·42)	30·30% (22·55–37·84)	12·31% (7·51–23·57)
	2021	6·00% (5·57–6·47)	29·31% (23·85–34·69)	0·75% (0·66–0·84)	2·22% (2·02–2·44)	32·59% (24·81–40·05)	12·35% (7·49–24·11)
	2030	5·76% (4·91–6·84)	29·68% (18·78–40·10)	0·72% (0·33–1·12)	2·21%* (1·31–3·38)	33·90% (28·32–39·64)	12·05% (8·70–15·45)
	Greenland						
	2012	7·66% (7·11–8·22)	50·42% (46·66–54·20)	3·05% (2·68–3·51)	1·06% (0·98–1·15)	28·23% (21·10–35·93)	11·95% (9·63–15·09)
	2021	7·57% (6·98–8·13)	51·45% (47·81–55·22)	2·62% (2·28–2·98)	0·97% (0·89–1·06)	30·59% (23·25–38·84)	11·16% (9·17–13·47)
	2030	7·26% (6·08–9·03)	51·74% (42·68–61·91)	2·36% (1·05–3·65)	0·96%* (0·56–1·43)	33·07% (26·89–40·42)	11·04% (8·95–13·20)
	USA						
	2012	8·07% (7·98–8·16)	44·80% (43·40–46·13)	2·74% (2·46–3·06)	0·70% (0·66–0·75)	19·36% (16·40–22·69)	11·92% (11·07–12·91)
	2021	8·26% (8·15–8·36)	45·69% (43·10–48·41)	2·47% (2·15–2·83)	0·66% (0·62–0·70)	21·56% (16·82–26·52)	11·92% (10·23–14·22)
	2030	8·16% (7·63–9·00)	46·53% (38·85–54·49)	2·38% (1·09–3·71)	0·67%* (0·41–1·02)	22·39% (20·30–24·93)	12·13% (9·80–14·59)
**Southern Latin America**							
2012		7·19% (6·89–7·50)	41·25% (39·42–42·95)	6·92% (6·30–7·58)	1·21% (1·12–1·30)	29·09% (23·79–34·89)	15·13% (8·45–26·63)
2021		7·27% (6·97–7·62)	43·13% (39·85–46·09)	6·46% (5·80–7·17)	1·07% (0·99–1·15)	33·59% (27·81–39·68)	14·84% (8·24–25·50)
2030		7·32% (5·79–8·97)	44·23% (19·38–60·65)	5·51% (2·78–7·74)	1·01%* (0·59–1·46)	37·37% (30·37–44·88)	14·30% (11·41–17·13)
	Argentina						
	2012	7·43% (7·03–7·84)	36·83% (34·57–38·74)	8·21% (7·33–9·15)	1·38% (1·26–1·50)	26·90% (20·29–34·55)	17·93% (8·06–35·15)
	2021	7·52% (7·12–7·95)	38·82% (34·52–42·78)	7·69% (6·78–8·70)	1·25% (1·13–1·37)	31·15% (23·82–39·03)	17·62% (7·92–33·25)
	2030	7·73% (5·79–9·75)	39·43% (9·67–59·02)	6·70% (3·29–9·48)	1·21%* (0·70–1·77)	34·54% (26·54–43·31)	17·07% (13·34–20·68)
	Chile						
	2012	6·23 % (5·85–6·61)	52·72 % (49·15–56·25)	2·30 % (2·06–2·56)	0·58 % (0·54–0·62)	36·21 % (28·36–44·72)	8·76 % (7·18–10·51)
	2021	6·26% (5·82–6·71)	54·66% (50·82–58·26)	2·04% (1·80–2·32)	0·42% (0·39–0·46)	41·81% (33·27–51·27)	8·05% (6·43–9·97)
	2030	6·09% (4·89–7·99)	55·43% (40·73–65·74)	1·83% (1·02–2·59)	0·40%* (0·24–0·61)	45·49% (36·17–55·73)	7·78% (5·69–9·93)
	Uruguay						
	2012	8·35% (7·92–8·78)	49·91% (45·93–53·53)	11·17% (10·27–12·22)	1·80% (1·66–1·96)	25·23% (18·23–32·84)	13·57% (7·94–25·32)
	2021	8·23% (7·71–8·75)	51·36% (47·05–55·12)	10·27% (9·20–11·39)	1·58% (1·44–1·74)	28·60% (21·19–36·46)	12·56% (7·59–23·86)
	2030	8·24% (6·78–10·14)	52·18% (32·34–65·04)	9·17% (4·71–12·29)	1·55%* (0·92–2·33)	32·70% (24·28–41·65)	12·28% (9·65–15·12)
**Western Europe**							
2012		6·85% (6·73–6·96)	30·95% (29·72–32·28)	1·49% (1·42–1·56)	1·24% (1·21–1·27)	24·54% (22·56–26·48)	8·79% (7·79–10·66)
2021		6·78% (6·67–6·91)	31·70% (30·30–33·10)	1·43% (1·36–1·50)	1·16% (1·14–1·19)	27·85% (25·76–30·14)	8·90% (7·72–11·17)
2030		6·55% (6·10–7·11)	32·47% (24·70–39·11)	1·38% (0·76–2·02)	1·17%* (0·71–1·71)	29·80% (24·97–35·67)	8·63% (7·47–9·93)
	Andorra						
	2012	6·56% (6·11–7·02)	47·62% (43·63–51·77)	1·27% (1·12–1·43)	1·02% (0·95–1·09)	25·55% (18·79–32·64)	8·03% (6·51–9·90)
	2021	6·56% (6·14–7·02)	48·68% (44·55–52·76)	1·28% (1·14–1·44)	1·05% (0·98–1·13)	29·06% (22·24–36·89)	7·92% (6·37–9·78)
	2030	6·37% (4·64–9·09)	49·42% (40·41–60·87)	1·27% (0·67–1·92)	1·07%* (0·67–1·56)	30·27% (23·01–39·14)	7·76% (6·40–9·24)
	Austria						
	2012	6·81% (6·39–7·23)	17·74% (14·48–21·28)	1·48% (1·33–1·64)	1·26% (1·17–1·35)	22·71% (16·80–29·84)	8·21% (6·59–10·42)
	2021	6·76% (6·32–7·23)	18·26% (14·73–21·83)	1·44% (1·30–1·59)	1·20% (1·11–1·28)	25·05% (18·90–32·40)	7·89% (6·41–9·79)
	2030	6·47% (5·70–7·66)	18·68% (12·48–26·67)	1·37% (0·74–2·05)	1·18%* (0·72–1·69)	27·15% (21·21–34·50)	7·81% (6·52–9·31)
	Belgium						
	2012	7·26% (6·86–7·70)	31·48% (26·84–36·02)	1·45% (1·29–1·61)	1·22% (1·14–1·30)	18·50% (13·17–23·98)	7·38% (6·17–8·78)
	2021	7·25% (6·80–7·69)	32·52% (28·07–37·21)	1·39% (1·24–1·56)	1·14% (1·06–1·22)	21·47% (15·64–28·30)	7·13% (5·89–8·60)
	2030	7·06% (6·69–7·70)	33·19% (24·29–44·27)	1·34% (0·71–2·02)	1·14%* (0·69–1·65)	23·07% (17·26–30·22)	6·96% (5·55–8·52)
	Cyprus						
	2012	11·82% (11·25–12·38)	45·83% (41·45–49·70)	1·81% (1·62–2·03)	1·67% (1·57–1·78)	24·69% (18·16–32·28)	8·60% (6·87–10·77)
	2021	11·66% (11·02–12·32)	47·16% (42·56–51·31)	1·74% (1·57–1·95)	1·58% (1·48–1·69)	29·21% (22·01–37·47)	8·45% (6·82–10·42)
	2030	11·06% (9·39–13·06)	47·99% (37·02–59·09)	1·64% (0·93–2·38)	1·53%* (0·94–2·20)	30·46% (23·11–39·75)	8·23% (6·67–10·22)
	Denmark						
	2012	6·56% (6·06–7·06)	49·17% (45·12–52·89)	1·57% (1·39–1·75)	1·37% (1·27–1·47)	28·39% (21·63–36·37)	8·52% (6·77–11·99)
	2021	6·51% (6·06–6·98)	49·83% (45·96–53·66)	1·53% (1·37–1·70)	1·31% (1·23–1·41)	31·31% (23·95–39·90)	8·21% (6·48–13·34)
	2030	6·65% (5·58–8·38)	50·22% (40·26–59·26)	1·46% (0·79–2·20)	1·29%* (0·79–1·83)	33·25% (26·30–43·05)	8·03% (6·94–9·45)
	Finland						
	2012	4·70% (4·37–5·02)	43·57% (39·42–47·88)	1·60% (1·44–1·80)	1·40% (1·30–1·49)	27·62% (20·87–35·69)	7·41% (6·11–8·90)
	2021	4·65% (4·31–5·01)	44·80% (40·28–49·10)	1·54% (1·38–1·72)	1·37% (1·28–1·47)	30·92% (23·49–38·96)	7·26% (5·84–8·78)
	2030	4·54% (4·42–4·89)	45·18% (37·00–55·17)	1·51% (0·81–2·27)	1·35%* (0·81–1·94)	32·92% (26·01–41·85)	7·05% (5·83–8·35)
	France						
	2012	6·57% (6·10–7·05)	20·87% (17·45–24·78)	1·50% (1·34–1·68)	1·27% (1·18–1·36)	21·73% (15·61–28·42)	6·53% (5·47–7·74)
	2021	6·55% (6·10–7·04)	21·59% (18·12–25·48)	1·43% (1·28–1·59)	1·19% (1·11–1·28)	25·19% (18·45–32·77)	6·71% (5·56–8·10)
	2030	6·51% (6·35–6·75)	22·19% (15·57–30·50)	1·38% (0·73–2·01)	1·19%* (0·72–1·74)	28·03% (21·86–36·56)	6·42% (4·89–8·22)
	Germany						
	2012	6·54% (6·12–6·95)	35·11% (31·16–39·39)	1·31% (1·19–1·45)	1·23% (1·15–1·31)	29·28% (22·59–36·56)	11·49% (7·36–21·41)
	2021	6·56% (6·13–6·96)	35·44% (30·77–39·92)	1·26% (1·14–1·39)	1·16% (1·08–1·24)	31·76% (24·64–39·64)	12·21% (7·24–24·05)
	2030	5·91% (4·96–7·30)	35·57% (28·06–42·29)	1·24% (0·67–1·86)	1·16%* (0·73–1·69)	34·14% (26·54–43·19)	11·96% (9·98–13·97)
	Greece						
	2012	8·95% (8·46–9·44)	36·35% (31·95–40·83)	2·08% (1·83–2·35)	0·76% (0·70–0·83)	27·05% (20·50–34·22)	9·86% (7·92–12·79)
	2021	8·98% (8·44–9·51)	37·20% (32·75–42·01)	2·10% (1·84–2·42)	0·77% (0·71–0·84)	31·86% (24·66–40·18)	10·08% (8·00–13·29)
	2030	8·84% (6·93–10·09)	37·86% (30·45–44·72)	2·02% (1·10–2·94)	0·76%* (0·47–1·13)	33·59% (26·00–43·65)	9·82% (7·95–11·88)
	Iceland						
	2012	6·64% (6·16–7·16)	41·74% (37·24–46·32)	1·51% (1·36–1·69)	1·30% (1·21–1·40)	31·15% (23·90–39·44)	6·91% (5·63–8·50)
	2021	6·65% (6·17–7·18)	42·30% (37·44–46·73)	1·46% (1·31–1·63)	1·26% (1·18–1·36)	33·26% (25·86–41·90)	6·69% (5·48–8·14)
	2030	6·28% (5·21–7·65)	42·53% (35·05–51·87)	1·42% (0·74–2·14)	1·24%* (0·77–1·80)	35·65% (27·86–46·01)	6·63% (5·35–8·37)
	Ireland						
	2012	4·51% (4·19–4·85)	17·91% (14·56–21·63)	1·44% (1·29–1·61)	1·20% (1·12–1·29)	28·28% (20·98–36·26)	7·97% (6·37–10·05)
	2021	5·32% (4·93–5·77)	18·72% (15·22–22·77)	1·33% (1·19–1·48)	1·10% (1·02–1·18)	32·74% (25·44–41·32)	7·61% (6·14–9·64)
	2030	5·31% (4·53–6·51)	19·20% (12·24–27·21)	1·29% (0·71–1·89)	1·10%* (0·68–1·57)	34·77% (27·26–44·34)	7·22% (6·02–8·57)
	Israel						
	2012	6·61% (6·17–7·06)	43·52% (39·02–47·58)	1·47% (1·31–1·64)	1·23% (1·15–1·31)	22·62% (16·68–29·03)	9·98% (7·86–14·89)
	2021	6·58% (6·08–7·07)	44·65% (40·30–49·01)	1·40% (1·25–1·56)	1·15% (1·07–1·24)	25·19% (18·59–32·82)	9·63% (7·58–14·05)
	2030	6·01% (5·51–6·77)	45·47% (33·98–57·09)	1·34% (0·71–1·99)	1·14%* (0·70–1·63)	27·19% (20·29–36·71)	9·28% (8·09–10·72)
	Italy						
	2012	7·48% (7·31–7·65)	39·92% (35·52–43·97)	1·43% (1·28–1·59)	1·19% (1·12–1·28)	20·47% (15·30–26·69)	8·66% (6·57–12·00)
	2021	7·30% (7·14–7·47)	40·74% (36·37–45·07)	1·39% (1·25–1·53)	1·14% (1·07–1·23)	23·95% (17·89–31·04)	8·47% (6·44–11·96)
	2030	6·72% (6·29–7·26)	41·25% (32·82–49·02)	1·33% (0·73–1·99)	1·15%* (0·72–1·65)	25·36% (19·19–33·40)	8·19% (6·83–9·82)
	Luxembourg						
	2012	6·17% (5·73–6·60)	43·65% (39·50–47·82)	1·63% (1·47–1·82)	1·46% (1·37–1·57)	24·24% (17·84–30·81)	7·92% (6·26–10·19)
	2021	6·18% (5·73–6·64)	44·85% (40·46–49·11)	1·54% (1·38–1·72)	1·37% (1·28–1·47)	27·30% (20·78–35·08)	7·60% (6·09–9·38)
	2030	6·11% (6·05–6·28)	45·51% (34·31–57·26)	1·49% (0·79–2·22)	1·34%* (0·85–1·92)	28·67% (22·68–36·68)	7·43% (6·21–9·02)
	Malta						
	2012	7·37% (6·92–7·81)	43·84% (39·59–48·30)	1·69% (1·51–1·88)	1·50% (1·40–1·60)	18·16% (13·19–24·10)	9·62% (7·73–12·23)
	2021	7·05% (6·59–7·51)	45·42% (41·17–49·79)	1·55% (1·39–1·73)	1·35% (1·25–1·45)	21·88% (16·25–28·49)	9·18% (7·44–11·64)
	2030	6·43% (4·81–8·15)	46·49% (35·01–58·88)	1·47% (0·81–2·15)	1·31%* (0·82–1·90)	23·69% (17·83–30·58)	8·96% (7·47–10·69)
	Monaco						
	2012	6·62% (6·15–7·09)	47·77% (43·29–51·82)	1·27% (1·14–1·41)	1·04% (0·97–1·12)	32·81% (25·08–41·49)	6·71% (5·37–8·59)
	2021	6·60% (6·17–7·05)	48·76% (44·36–52·64)	1·19% (1·06–1·33)	0·97% (0·91–1·04)	35·76% (27·59–44·85)	6·42% (5·15–7·93)
	2030	6·56% (6·40–7·04)	49·43% (39·92–61·04)	1·18% (0·62–1·78)	0·98%* (0·60–1·44)	37·77% (28·40–48·29)	6·24% (5·11–7·57)
	Netherlands						
	2012	5·60% (5·22–6·02)	32·51% (28·69–36·36)	1·45% (1·30–1·63)	1·24% (1·15–1·33)	21·52% (15·61–28·37)	8·27% (6·63–10·55)
	2021	5·38% (4·98–5·83)	34·28% (29·92–38·52)	1·45% (1·30–1·63)	1·23% (1·15–1·32)	24·49% (17·98–32·09)	7·93% (6·41–9·82)
	2030	5·31% (4·91–5·96)	34·48% (28·84–40·91)	1·40% (0·73–2·09)	1·21%* (0·74–1·75)	26·39% (20·12–33·82)	7·76% (6·45–9·21)
	Norway						
	2012	5·24% (5·11–5·37)	54·94% (51·81–58·44)	1·46% (1·31–1·63)	1·26% (1·17–1·35)	27·64% (20·85–35·46)	7·11% (5·74–9·11)
	2021	5·08% (4·95–5·23)	56·07% (52·74–59·11)	1·40% (1·25–1·57)	1·20% (1·11–1·28)	30·28% (23·49–38·53)	6·97% (5·50–9·24)
	2030	5·05% (5·03–5·11)	56·33% (43·04–68·95)	1·37% (0·72–2·05)	1·19%* (0·73–1·70)	31·88% (24·42–39·61)	6·90% (5·56–8·50)
	Portugal						
	2012	7·52% (7·11–7·95)	34·20% (30·19–38·06)	1·71% (1·53–1·92)	1·48% (1·38–1·59)	21·39% (15·66–28·18)	9·65% (7·74–12·81)
	2021	8·38% (7·91–8·90)	35·22% (30·89–39·53)	1·63% (1·47–1·82)	1·39% (1·29–1·49)	26·17% (19·75–33·60)	9·24% (7·40–11·93)
	2030	8·37% (7·26–10·27)	35·82% (28·18–44·20)	1·52% (0·84–2·23)	1·37%* (0·83–1·97)	27·67% (21·29–36·15)	8·68% (7·38–10·29)
	San Marino						
	2012	6·65% (6·17–7·14)	47·03% (42·61–51·04)	1·40% (1·25–1·57)	1·17% (1·09–1·26)	30·24% (22·79–38·26)	7·79% (6·20–9·62)
	2021	6·64% (6·14–7·16)	47·96% (43·45–51·99)	1·42% (1·27–1·60)	1·18% (1·10–1·27)	33·54% (25·22–42·09)	7·76% (6·22–9·45)
	2030	6·59% (6·24–7·45)	48·92% (38·66–60·74)	1·40% (0·69–2·11)	1·21%* (0·72–1·73)	35·68% (27·19–45·71)	7·46% (6·08–9·06)
	Spain						
	2012	8·33% (7·92–8·74)	38·66% (34·32–42·96)	1·19% (1·06–1·32)	1·90% (1·76–2·07)	24·16% (18·01–31·06)	8·40% (6·83–10·30)
	2021	8·21% (7·76–8·68)	39·82% (35·23–44·00)	1·15% (1·03–1·29)	1·82% (1·67–1·97)	28·49% (21·83–36·02)	8·28% (6·65–10·36)
	2030	7·79% (7·39–8·43)	40·36% (31·84–47·44)	1·11% (0·65–1·54)	1·82%* (1·09–2·63)	29·74% (23·02–38·50)	7·82% (6·55–9·29)
	Sweden						
	2012	4·16% (3·90–4·41)	50·51% (47·18–53·80)	1·45% (1·30–1·62)	1·24% (1·15–1·32)	25·59% (18·74–33·22)	7·90% (6·15–11·01)
	2021	4·19% (3·94–4·45)	50·79% (47·00–54·44)	1·41% (1·26–1·58)	1·18% (1·10–1·27)	28·13% (21·02–36·23)	7·66% (5·98–11·06)
	2030	4·12% (3·98–4·30)	51·40% (40·38–61·33)	1·37% (0·71–2·05)	1·17%* (0·72–1·72)	30·64% (24·33–38·97)	7·64% (6·28–9·15)
	Switzerland						
	2012	6·83% (6·41–7·23)	39·33% (34·86–44·02)	1·40% (1·26–1·56)	1·20% (1·12–1·28)	25·75% (18·85–33·28)	11·64% (7·09–21·76)
	2021	6·79% (6·32–7·25)	40·66% (36·33–45·19)	1·35% (1·22–1·51)	1·15% (1·07–1·23)	27·42% (20·51–34·52)	12·97% (7·22–25·25)
	2030	6·60% (6·03–7·54)	40·83% (27·88–49·38)	1·32% (0·69–1·99)	1·13%* (0·69–1·60)	28·64% (22·00–36·94)	12·73% (10·62–14·98)
	UK						
	2012	6·87% (6·82–6·93)	16·84% (13·44–20·48)	1·76% (1·49–2·08)	0·88% (0·80–0·95)	27·21% (25·61–28·74)	8·23% (7·09–9·56)
	2021	6·88% (6·82–6·94)	17·19% (13·81–21·05)	1·68% (1·43–1·96)	0·82% (0·75–0·90)	29·56% (27·48–31·57)	8·42% (6·78–10·80)
	2030	7·21% (6·02–8·69)	17·70% (7·42–25·31)	1·64% (0·97–2·33)	0·82%* (0·49–1·20)	30·90% (25·48–37·68)	8·22% (6·82–9·63)
**Latin America and Caribbean**							
**2012**		**8·81% (8·72–8·91)**	**35·59% (34·38–36·81)**	**14·55% (14·37–14·74)**	**2·42% (2·39–2·45)**	**22·58% (20·26–25·11)**	**25·10% (23·16–27·56)**
**2021**		**8·83% (8·73–8·93)**	**37·35% (35·73–39·05)**	**12·92% (12·70–13·14)**	**2·11% (2·08–2·14)**	**27·83% (25·07–30·80)**	**24·59% (22·46–27·16)**
**2030**		**8·92% (8·32–9·60)**	**38·91% (18·98–58·18)**	**11·07% (6·18–14·40)**	2·05%*(1·40–2·76)	**31·66% (26·35–38·03)**	**23·11% (20·55–25·92)**
Andean Latin America							
2012		7·61% (7·32–7·88)	59·64% (58·05–61·04)	20·85% (20·23–21·48)	1·67% (1·62–1·73)	29·59% (25·85–33·83)	19·63% (18·68–20·57)
2021		7·54% (7·23–7·87)	61·98% (59·83–63·97)	16·69% (15·85–17·53)	1·37% (1·32–1·42)	35·29% (30·74–40·20)	21·60% (19·48–24·71)
2030		7·63% (7·02–8·61)	64·45% (47·66–76·40)	13·36% (7·05–18·16)	1·28%* (0·88–1·76)	38·99% (30·55–49·78)	19·36% (17·06–21·89)
	Bolivia						
	2012	6·33% (5·84–6·82)	57·50% (54·92–60·10)	22·16% (20·87–23·50)	2·34% (2·20–2·48)	29·53% (24·28–35·56)	25·45% (23·41–27·57)
	2021	6·13% (5·65–6·61)	59·87% (55·91–63·45)	18·06% (16·27–19·79)	1·85% (1·73–1·97)	36·28% (29·08–44·18)	21·67% (19·34–24·11)
	2030	6·04% (5·86–6·57)	61·47% (41·27–74·90)	14·47% (7·45–20·65)	1·78%* (1·23–2·41)	37·51% (26·84–50·25)	19·86% (16·86–23·12)
	Ecuador						
	2012	8·42% (7·96–8·92)	45·01% (40·73–48·75)	22·44% (20·98–23·92)	2·55% (2·41–2·69)	18·25% (15·05–21·82)	12·47% (11·31–13·60)
	2021	8·54% (8·00–9·08)	46·94% (41·15–51·68)	20·29% (18·52–22·04)	2·20% (2·08–2·33)	23·88% (18·40–29·46)	11·97% (10·48–13·60)
	2030	8·91% (7·85–10·20)	48·90% (24·67–65·41)	17·41% (10·36–22·50)	2·15%* (1·43–3·00)	26·60% (18·47–39·29)	11·21% (9·41–13·05)
	Peru						
	2012	7·71% (7·24–8·19)	69·02% (67·71–70·29)	19·37% (18·59–20·13)	0·87% (0·82–0·92)	36·28% (29·49–44·29)	21·27% (19·77–22·82)
	2021	7·60% (7·07–8·12)	71·48% (68·92–73·94)	14·11% (12·83–15·38)	0·67% (0·62–0·72)	41·06% (33·28–49·52)	26·56% (22·75–31·93)
	2030	7·57% (6·75–9·53)	73·81%* (59·96–83·06)	10·83% (5·04–16·10)	0·65%* (0·45–0·87)	45·89% (34·06–60·13)	23·52% (20·38–27·07)
Caribbean							
2012		13·05% (12·59–13·50)	26·10% (25·02–27·14)	14·05% (13·58–14·54)	4·36% (4·22–4·50)	16·71% (15·37–18·24)	37·60% (35·46–40·14)
2021		13·18% (12·71–13·69)	26·39% (24·57–28·09)	13·36% (12·72–13·98)	3·99% (3·83–4·15)	19·01% (16·99–21·31)	40·39% (37·16–44·17)
2030		13·19% (11·10–16·18)	28·40% (8·91–48·46)	11·78% (7·19–15·19)	3·93% (2·80–5·10)	20·23% (15·95–25·24)	39·43% (35·92–43·37)
	Antigua and Barbuda						
	2012	9·77% (9·06–10·48)	27·37% (20·92–33·51)	7·63% (6·71–8·64)	2·97% (2·75–3·22)	23·48% (17·10–31·01)	34·07% (28·21–42·37)
	2021	9·82% (9·08–10·52)	30·00% (23·88–36·69)	7·15% (6·32–8·05)	2·77% (2·56–2·98)	28·08% (20·80–35·82)	33·32% (27·75–41·11)
	2030	9·67% (7·59–11·68)	30·95% (6·85–56·57)	6·60% (3·02–9·62)	2·67%* (1·77–3·64)	30·64% (21·75–39·34)	32·19% (26·54–38·31)
	The Bahamas						
	2012	10·17% (9·50–10·86)	27·93% (21·88–34·40)	7·65% (6·71–8·67)	2·90% (2·68–3·15)	29·11% (21·54–37·04)	35·73% (29·24–45·22)
	2021	10·17% (9·53–10·81)	30·51% (24·33–36·70)	7·35% (6·48–8·34)	2·82% (2·60–3·07)	34·15% (26·60–42·87)	35·62% (29·56–45·34)
	2030	10·00% (8·87–11·37)	31·36% (7·65–56·73)	7·04% (3·51–10·02)	2·81%* (1·85–3·84)	35·19% (26·22–45·24)	35·03% (28·84–40·90)
	Barbados						
	2012	9·73% (9·12–10·38)	19·19% (14·79–24·01)	11·78% (10·69–12·98)	7·64% (7·21–8·09)	20·59% (15·90–25·81)	30·65% (26·00–37·38)
	2021	9·60% (8·93–10·23)	20·70% (15·93–26·17)	10·92% (9·90–12·04)	7·14% (6·69–7·63)	25·26% (19·37–31·82)	30·31% (25·57–37·30)
	2030	9·26% (7·94–11·37)	21·40% (4·09–46·25)	10·10% (5·89–13·50)	7·04% (4·86–9·34)	25·48% (19·30–33·70)	29·56% (23·67–35·70)
	Belize						
	2012	10·36% (9·71–11·02)	21·50% (18·12–24·84)	16·98% (15·81–18·19)	3·35% (3·12–3·60)	20·78% (16·81–25·32)	40·75% (33·83–50·73)
	2021	10·29% (9·64–10·95)	25·85% (21·28–30·81)	14·66% (13·24–16·03)	2·77% (2·56–2·97)	24·47% (18·70–30·77)	40·45% (33·22–51·18)
	2030	10·54% (8·41–13·31)	28·04% (13·13–52·14)	12·96% (7·72–16·95)	2·73%* (1·83–3·78)	26·33% (19·96–33·71)	39·36% (32·59–45·17)
	Bermuda						
	2012	9·44% (8·74–10·18)	29·26% (22·76–36·01)	5·60% (4·88–6·31)	1·97% (1·83–2·14)	31·80% (23·87–40·41)	23·03% (18·63–28·93)
	2021	9·46% (8·77–10·18)	31·43% (24·80–37·97)	5·25% (4·62–5·93)	1·85% (1·72–2·01)	37·27% (28·99–46·09)	22·19% (17·93–27·56)
	2030	9·27% (8·74–10·16)	32·32% (6·10–57·70)	4·96% (1·99–7·47)	1·82%* (1·24–2·47)	39·40% (30·71–50·37)	21·23% (16·32–26·75)
	Cuba						
	2012	5·65% (5·32–5·99)	32·85% (29·59–36·18)	6·15% (5·55–6·77)	1·92% (1·77–2·07)	22·69% (20·28–25·16)	31·35% (26·33–39·31)
	2021	5·69% (5·27–6·13)	35·05% (30·17–39·67)	5·84% (5·24–6·53)	1·87% (1·74–2·01)	27·63% (23·06–32·26)	30·26% (25·34–37·47)
	2030	5·61% (5·54–5·75)	36·27% (16·75–57·52)	5·72% (2·96–8·49)	1·82%* (1·20–2·58)	30·66% (21·78–41·84)	29·31% (23·63–35·23)
	Dominica						
	2012	10·01% (9·35–10·67)	29·92% (23·94–36·26)	7·58% (6·70–8·56)	2·84% (2·63–3·07)	38·31% (30·26–47·22)	33·50% (30·21–36·76)
	2021	10·14% (9·49–10·85)	32·04% (25·42–37·92)	7·46% (6·56–8·45)	2·81% (2·59–3·04)	44·07% (35·38–53·23)	33·91% (29·97–38·93)
	2030	9·88% (9·05–11·47)	32·77% (5·95–58·10)	7·09% (3·47–10·19)	2·79%* (1·80–3·85)	46·25% (37·37–57·18)	33·88% (28·55–39·39)
	Dominican Republic						
	2012	12·46% (11·65–13·29)	6·50% (5·44–7·63)	9·07% (8·41–9·77)	2·51% (2·36–2·66)	16·32% (12·94–20·43)	33·67% (30·16–37·25)
	2021	12·32% (11·59–13·12)	6·49% (4·94–8·42)	7·53% (6·80–8·28)	2·13% (1·99–2·27)	19·95% (15·27–25·35)	33·50% (29·43–39·50)
	2030	13·24% (9·57–21·59)	6·16% (0·12–19·79)	6·29% (2·99–9·07)	2·15%* (1·39–3·05)	23·71% (16·36–33·11)	31·92% (26·49–37·84)
	Grenada						
	2012	9·86% (9·28–10·52)	22·79% (17·17–29·08)	8·92% (7·83–10·04)	3·60% (3·36–3·89)	16·55% (11·70–21·89)	33·66% (29·01–40·32)
	2021	9·90% (9·28–10·57)	25·59% (19·92–31·58)	8·10% (7·16–9·13)	3·17% (2·94–3·43)	20·54% (15·12–27·31)	32·66% (27·68–38·93)
	2030	9·83% (8·81–11·42)	26·54% (6·79–51·50)	7·63% (3·72–10·79)	3·11% (2·13–4·28)	22·99% (16·29–31·14)	31·66% (24·96–38·49)
	Guyana						
	2012	13·14% (12·40–13·93)	25·88% (23·04–28·85)	15·15% (14·19–16·11)	7·67% (7·25–8·11)	15·69% (12·53–19·17)	38·93% (35·52–43·65)
	2021	12·74% (12·01–13·48)	27·84% (23·14–32·50)	11·19% (10·12–12·33)	6·25% (5·87–6·70)	18·67% (14·17–23·93)	37·84% (32·91–45·55)
	2030	12·70% (10·44–14·90)	31·65% (5·52–58·23)	9·16%* (4·84–12·01)	5·53% (4·04–6·93)	22·69% (16·57–31·41)	35·02% (29·92–39·74)
	Haiti						
	2012	16·52% (15·55–17·58)	38·18% (36·03–40·27)	24·19% (23·06–25·30)	7·06% (6·74–7·42)	9·31% (7·43–11·87)	51·82 % (47·72–57·43)
	2021	16·05% (15·12–17·04)	37·33% (33·80–41·05)	22·35% (20·93–23·76)	6·19% (5·83–6·55)	11·81% (8·91–15·53)	58·58% (50·39–68·76)
	2030	15·49% (12·11–20·56)	40·32% (10·75–65·35)	18·44% (11·43–23·30)	5·76% (4·12–7·38)	12·28% (8·67–17·13)	55·70% (51·00–59·76)
	Jamaica						
	2012	12·81% (12·15–13·52)	28·24% (24·59–31·83)	7·27% (6·60–8·01)	3·33% (3·12–3·55)	23·82% (17·36–31·11)	35·44% (29·40–44·14)
	2021	12·53% (11·70–13·47)	31·13% (25·55–36·92)	6·39% (5·68–7·19)	3·04% (2·82–3·28)	28·29% (21·37–36·10)	35·52% (29·20–44·80)
	2030	12·26% (10·41–14·20)	32·93% (7·80–56·85)	5·76% (2·89–8·24)	2·87%* (1·90–4·00)	29·75% (23·13–38·20)	34·90% (28·87–40·58)
	Puerto Rico						
	2012	9·63% (8·98–10·31)	29·35% (23·31–35·59)	5·44% (4·77–6·20)	1·78% (1·64–1·95)	41·45% (32·71–50·47)	25·69% (21·23–32·14)
	2021	9·66% (9·04–10·24)	32·01% (25·50–38·63)	5·10% (4·44–5·83)	1·66% (1·51–1·80)	46·10% (37·32–54·90)	25·19% (20·59–31·81)
	2030	9·35% (7·84–11·00)	32·36% (6·64–55·91)	4·77%* (1·97–7·19)	1·65%* (1·09–2·24)	48·92% (38·16–60·56)	23·99% (19·02–29·99)
	Saint Kitts and Nevis						
	2012	9·70% (9·06–10·40)	28·26% (21·86–34·57)	7·62% (6·74–8·65)	2·93% (2·71–3·17)	25·14% (18·43–32·82)	36·28% (29·93–45·84)
	2021	9·68% (9·00–10·34)	30·83% (24·44–37·23)	7·10% (6·24–8·06)	2·67% (2·47–2·91)	30·28% (23·02–38·65)	36·14% (29·64–46·01)
	2030	9·99% (8·74–11·58)	31·82% (6·14–57·54)	6·56% (2·99–9·65)	2·61%* (1·73–3·50)	31·99% (24·22–42·53)	34·98% (28·17–41·92)
	Saint Lucia						
	2012	19·13% (18·04–20·26)	5·04% (3·37–7·22)	3·46% (3·07–3·90)	4·33% (3·93–4·71)	10·87% (7·85–14·82)	37·59% (31·56–46·56)
	2021	19·17% (17·89–20·40)	5·80% (3·87–8·41)	3·34% (2·96–3·76)	4·06% (3·69–4·46)	13·79% (9·88–18·70)	37·64% (31·60–46·84)
	2030	19·21% (16·23–22·67)	5·91% (0·01–21·35)	3·01% (1·72–4·32)	4·00% (2·68–5·36)	14·50% (10·14–20·19)	36·02% (29·71–42·03)
	Saint Vincent and the Grenadines						
	2012	9·94% (9·16–10·71)	22·38% (16·92–28·56)	8·38% (7·34–9·50)	3·27% (3·02–3·52)	14·14% (10·14–18·99)	39·11% (32·55–48·38)
	2021	10·01% (9·35–10·71)	24·96% (19·27–31·22)	7·78% (6·87–8·84)	2·97% (2·73–3·22)	17·76% (12·77–23·43)	38·98% (32·14–49·29)
	2030	9·89% (7·89–12·13)	25·85% (6·12–50·27)	7·33% (3·42–10·59)	2·96%* (2·03–4·10)	20·99% (14·83–29·09)	38·19% (31·77–43·61)
	Suriname						
	2012	11·23% (10·52–11·95)	4·38% (3·03–5·86)	9·45% (8·59–10·38)	5·65% (5·29–6·05)	7·54% (5·41–10·14)	41·14% (34·47–50·99)
	2021	11·04% (10·42–11·74)	4·86% (3·26–6·77)	8·30% (7·35–9·34)	5·16% (4·78–5·57)	9·23% (6·56–12·28)	40·79% (33·94–50·13)
	2030	10·68% (8·30–13·35)	4·97% (2·05–11·19)	7·28% (3·60–10·68)	5·01% (3·53–6·54)	10·32% (6·85–15·09)	39·55% (33·83–45·36)
	Trinidad and Tobago						
	2012	20·57% (19·44–21·97)	13·68% (10·70–16·95)	8·88% (8·05–9·78)	5·93% (5·54–6·38)	13·13% (9·68–17·49)	37·07% (30·50–46·41)
	2021	20·46% (19·31–21·68)	14·08% (10·67–18·22)	8·03% (7·13–9·03)	5·33% (4·91–5·77)	16·10% (11·88–21·83)	36·48% (29·77–45·77)
	2030	20·26% (17·95–22·15)	14·80% (0·71–37·04)	7·25% (3·63–11·02)	5·12% (3·57–6·84)	18·18% (10·88–28·30)	35·62% (29·63–41·98)
	Virgin Islands						
	2012	10·11% (9·40–10·78)	29·69% (23·86–35·90)	6·50% (5·66–7·38)	2·28% (2·10–2·45)	44·50% (35·96–53·39)	31·11% (25·09–39·34)
	2021	10·18% (9·49–10·92)	31·83% (25·95–37·60)	6·38% (5·60–7·27)	2·25% (2·07–2·43)	48·54% (39·65–57·55)	31·14% (25·29–39·39)
	2030	9·66% (7·66–12·10)	32·44% (6·52–56·67)	6·13%* (2·99–8·87)	2·23%* (1·51–3·05)	50·65% (39·98–61·16)	30·74% (24·79–36·80)
**Central Latin America**							
2012		8·36% (8·21–8·51)	29·48% (27·80–31·19)	16·43% (16·13–16·76)	2·30% (2·25–2·33)	19·91% (17·17–22·72)	14·77% (13·92–16·10)
2021		8·38% (8·22–8·53)	31·75% (29·48–33·80)	14·38% (14·04–14·76)	1·99% (1·95–2·03)	24·54% (21·48–27·90)	14·15% (13·37–15·25)
2030		8·47% (7·77–9·43)	32·90% (13·55–51·82)	12·53% (7·32–16·06)	1·92%* (1·29–2·62)	27·26% (20·81–35·96)	13·52% (11·92–15·36)
	Colombia						
	2012	8·54% (8·00–9·10)	42·75% (39·94–45·47)	11·44% (10·61–12·31)	1·50% (1·42–1·60)	13·07% (10·05–16·77)	12·85% (10·91–15·06)
	2021	8·79% (8·15–9·40)	40·83% (35·68–45·74)	8·61% (7·67–9·66)	1·26% (1·18–1·35)	17·47% (12·93–22·87)	11·96% (10·10–14·38)
	2030	9·36% (7·58–11·55)	41·95% (12·65–62·82)	7·41% (3·05–10·87)	1·19%* (0·77–1·69)	19·54% (14·02–26·41)	11·25% (9·20–13·84)
	Costa Rica						
	2012	6·92% (6·42–7·45)	39·55% (35·83–43·36)	4·70% (4·10–5·33)	1·18% (1·08–1·29)	29·98% (23·12–38·05)	18·97% (14·33–26·98)
	2021	6·86% (6·35–7·38)	41·71% (36·06–46·78)	3·56% (3·09–4·13)	1·16% (1·05–1·28)	35·23% (27·65–44·40)	18·29% (13·57–27·50)
	2030	6·96% (5·90–8·09)	42·80% (17·12–61·19)	3·10%* (1·54–4·46)	1·13%* (0·73–1·62)	40·07% (29·61–53·86)	17·65% (14·38–21·32)
	El Salvador						
	2012	8·51% (7·95–9·09)	50·00% (47·07–52·70)	16·87% (15·73–17·95)	1·81% (1·70–1·92)	16·62% (12·23–21·93)	9·95% (8·72–11·19)
	2021	8·46% (7·91–9·01)	52·20% (47·12–56·61)	14·25% (12·72–15·81)	1·55% (1·45–1·66)	20·85% (15·48–27·44)	9·28% (7·87–10·94)
	2030	8·31% (6·84–10·05)	54·62% (27·15–70·98)	11·08%* (5·64–15·41)	1·51%* (0·96–2·12)	24·59% (17·36–33·60)	8·70% (6·93–10·75)
	Guatemala						
	2012	10·40% (9·71–11·06)	47·42% (45·62–49·20)	43·18% (40·86–45·97)	1·34% (1·25–1·44)	18·87% (15·22–23·32)	36·42 % (33·50–40·08)
	2021	10·20% (9·53–10·95)	50·52% (47·09–53·91)	39·62% (37·61–42·19)	1·02% (0·94–1·10)	23·45% (17·76–29·63)	33·51% (29·38–39·13)
	2030	9·18% (7·90–10·28)	51·62% (28·00–67·70)	34·92% (24·71–40·87)	0·93%* (0·60–1·33)	26·54% (20·05–35·75)	30·99% (26·42–35·45)
	Honduras						
	2012	9·48% (8·77–10·12)	31·66% (29·39–33·81)	24·42% (23·08–25·85)	1·62% (1·53–1·73)	14·06% (10·51–18·54)	16·77% (15·46–18·07)
	2021	9·50% (8·90–10·11)	34·27% (29·56–38·71)	20·51% (18·95–22·23)	1·58% (1·48–1·69)	17·08% (12·62–22·55)	16·32% (14·30–18·46)
	2030	8·86% (6·63–11·40)	34·95% (7·99–56·27)	15·87% (9·57–20·45)	1·46%* (0·91–2·11)	20·28% (14·94–27·98)	15·59% (13·28–18·21)
	Mexico						
	2012	8·30% (8·15–8·45)	25·68% (22·48–28·85)	14·17% (13·89–14·44)	2·58% (2·54–2·63)	21·23% (16·53–26·37)	11·36% (11·04–11·70)
	2021	8·35% (8·20–8·50)	28·91% (24·88–32·74)	12·36% (12·07–12·66)	2·26% (2·22–2·30)	26·34% (20·45–32·30)	10·80% (10·40–11·17)
	2030	8·30% (7·49–9·60)	29·51% (12·27–47·77)	10·29% (5·30–13·95)	2·23%* (1·50–3·06)	28·55% (18·61–41·64)	10·57% (9·38–11·86)
	Nicaragua						
	2012	8·25% (7·66–8·82)	32·85% (29·28–36·43)	17·34% (16·46–18·24)	1·87% (1·76–1·99)	18·08% (14·33–22·72)	16·29% (14·08–18·81)
	2021	8·07% (7·57–8·57)	34·49% (29·24–39·83)	14·72% (13·36–16·05)	1·51% (1·41–1·61)	22·37% (16·99–28·30)	15·86% (13·51–18·79)
	2030	7·58% (6·59–9·03)	35·87% (11·49–56·03)	10·83% (5·63–14·75)	1·42%* (0·87–2·01)	27·72% (19·04–40·71)	14·13% (11·81–16·99)
	Panama						
	2012	8·09% (7·56–8·68)	20·02% (16·46–23·82)	18·28% (17·14–19·61)	1·30% (1·21–1·39)	27·67% (20·67–35·46)	26·17% (19·37–35·86)
	2021	7·80% (7·28–8·36)	22·43% (17·97–26·91)	14·44% (13·04–15·91)	0·97% (0·90–1·04)	33·44% (25·83–42·34)	23·23% (16·84–34·40)
	2030	7·63% (6·57–8·93)	23·01% (5·12–44·98)	12·82% (6·57–17·49)	0·93%* (0·59–1·33)	39·59% (29·38–50·54)	22·18% (18·54–25·84)
	Venezuela						
	2012	7·03% (6·55–7·54)	10·08% (6·77–13·81)	14·31% (13·19–15·48)	3·60% (3·39–3·82)	26·08% (19·80–33·66)	19·89% (14·49–30·30)
	2021	7·03% (6·59–7·52)	11·16% (7·79–15·50)	14·18% (12·89–15·64)	3·55% (3·30–3·81)	31·52% (23·81–40·36)	20·20% (15·00–29·78)
	2030	7·67% (6·50–8·84)	11·06% (0·58–30·63)	12·62% (7·62–16·19)	3·39% (2·39–4·49)	35·14% (27·15–43·81)	19·45% (15·97–23·50)
**Tropical Latin America**							
2012		8·85% (8·73–8·98)	37·73% (35·00–40·78)	10·01% (9·75–10·25)	2·39% (2·34–2·43)	25·13% (19·52–31·36)	35·34% (30·59–41·83)
2021		8·81% (8·68–8·96)	38·39% (34·13–42·75)	9·29% (9·04–9·55)	2·10% (2·06–2·14)	31·41% (24·66–38·97)	33·99% (28·90–40·51)
2030		8·98% (7·80–9·94)	38·81% (15·38–61·36)	8·39% (4·24–11·43)	2·07%* (1·40–2·79)	36·52% (30·20–44·96)	32·41% (27·80–36·97)
	Brazil						
	2012	8·91% (8·79–9·04)	37·86% (34·99–41·01)	10·00% (9·75–10·26)	2·40% (2·35–2·44)	25·15% (19·45–31·66)	35·48% (30·60–42·20)
	2021	8·88% (8·74–9·03)	38·55% (34·11–43·17)	9·38% (9·13–9·64)	2·13% (2·09–2·17)	31·45% (24·34–39·29)	34·14% (28·88–40·84)
	2030	9·01% (7·81–10·01)	38·84% (15·66–61·44)	8·50% (4·30–11·54)	2·09%* (1·41–2·81)	36·55% (30·02–45·15)	32·58% (27·85–37·20)
	Paraguay						
	2012	7·56% (7·07–8·05)	34·78% (31·85–37·95)	10·05% (9·17–11·03)	2·18% (2·08–2·30)	24·60% (18·11–31·85)	30·77% (25·98–37·03)
	2021	7·45% (6·99–7·97)	35·03% (30·33–39·63)	7·15% (6·34–8·03)	1·45% (1·36–1·54)	30·36% (23·28–38·29)	29·27% (24·67–35·71)
	2030	8·00% (6·39–9·35)	37·89% (6·57–60·54)	5·54%* (2·57–8·57)	1·58%* (1·04–2·28)	35·68% (26·15–46·79)	27·73% (22·62–32·47)
**North Africa and Middle East**							
**2012**		**12·06% (11·79–12·33)**	**36·01% (35·15–36·84)**	**23·24% (22·81–23·69)**	**7·43% (7·32–7·55)**	**24·49% (22·90–26·20)**	**33·96% (32·19–35·79)**
**2021**		**11·83% (11·57–12·11)**	**39·76% (38·49–40·97)**	**19·82% (19·33–20·35)**	**5·89% (5·77–6·01)**	**31·89% (29·64–34·15)**	**32·84% (31·04–34·74)**
**2030**		**11·69% (10·12–14·66)**	**41·98% (18·21–61·89)**	**17·29% (11·15–20·62)**	**5·86% (4·67–6·96)**	**36·65% (32·07–41·89)**	**30·62% (27·37–33·99)**
	Afghanistan						
	2012	17·38% (16·23–18·56)	53·09% (51·18–55·23)	42·50% (39·32–46·35)	8·72% (8·33–9·12)	8·55% (5·86–12·17)	24·96% (22·76–27·76)
	2021	15·96% (14·87–17·03)	51·95% (48·22–55·54)	35·81% (33·54–38·77)	6·74% (6·36–7·12)	11·07% (7·66–15·14)	23·89% (20·90–27·62)
	2030	15·72% (12·50–22·73)	52·71% (25·15–71·45)	31·67% (17·65–39·33)	6·57% (4·88–8·18)	13·74% (9·39–19·38)	22·62% (18·96–26·66)
	Algeria						
	2012	6·08% (5·63–6·59)	27·27% (25·26–29·31)	14·93% (13·57–16·19)	5·75% (5·47–6·05)	17·79% (12·80–23·51)	37·16% (29·75–47·11)
	2021	5·97% (5·53–6·47)	31·01% (26·55–35·13)	12·29% (10·99–13·54)	4·36% (4·13–4·61)	25·28% (18·57–32·34)	35·86% (28·97–45·91)
	2030	6·23% (4·22–9·58)	32·37% (12·22–54·00)	8·89% (4·57–12·31)	4·03% (3·05–4·95)	30·24% (22·95–39·05)	33·09% (27·98–38·88)
	Bahrain						
	2012	9·75% (9·28–10·26)	36·05% (30·32–41·62)	10·64% (9·53–11·85)	3·47% (3·22–3·73)	27·67% (20·56–35·79)	45·39% (37·01–56·46)
	2021	10·03% (9·46–10·67)	39·68% (34·08–45·21)	9·68% (8·68–10·87)	2·92% (2·69–3·13)	38·17% (29·64–47·25)	42·66% (33·34–55·19)
	2030	9·85% (7·74–13·65)	42·79% (17·59–64·48)	6·91% (3·46–10·31)	2·73%* (1·79–3·71)	42·07% (33·67–51·03)	39·93% (33·04–46·63)
	Egypt						
	2012	15·41% (14·48–16·37)	43·11% (41·46–44·62)	23·03% (21·91–24·14)	6·64% (6·36–6·94)	37·15% (31·13–43·44)	29·11% (26·63–32·19)
	2021	15·06% (14·22–15·90)	44·89% (40·89–48·65)	18·30% (16·86–19·71)	4·71% (4·44–4·97)	48·37% (39·79–57·11)	25·84% (22·53–29·82)
	2030	15·00% (12·96–16·49)	46·87% (18·60–68·12)	13·78% (7·82–18·07)	4·63% (3·51–5·88)	52·60% (43·93–62·08)	21·76% (17·64–26·57)
	Iran						
	2012	8·62% (8·44–8·80)	49·12% (44·61–53·51)	10·65% (9·63–11·71)	4·18% (3·94–4·45)	24·41% (17·97–32·15)	28·51% (24·45–33·54)
	2021	8·62% (8·45–8·79)	52·18% (46·83–56·75)	9·15% (8·11–10·30)	3·34% (3·08–3·60)	35·08% (26·98–43·93)	28·09% (23·80–33·56)
	2030	8·39% (7·13–9·19)	54·68% (31·83–71·19)	7·21%* (3·40–10·16)	3·26% (2·31–4·20)	39·93% (31·96–50·22)	25·55% (20·40–31·35)
	Iraq						
	2012	13·24% (12·37–14·08)	22·80% (21·40–24·18)	24·01% (22·89–25·06)	7·05% (6·72–7·40)	24·05% (20·06–28·24)	33·12% (27·26–42·76)
	2021	13·16% (12·25–14·04)	30·11% (27·50–32·54)	17·93% (16·57–19·30)	4·95% (4·67–5·25)	31·27% (24·98–38·26)	30·71% (25·30–38·73)
	2030	12·34% (10·11–16·53)	32·82% (3·93–58·11)	15·12% (8·80–20·30)	4·51% (3·35–5·62)	35·41% (28·20–43·16)	28·02% (22·18–33·33)
	Jordan						
	2012	11·59% (10·87–12·29)	23·90% (22·23–25·51)	9·31% (8·43–10·23)	2·59% (2·42–2·78)	17·31% (13·39–21·47)	34·40% (32·61–36·25)
	2021	12·09% (11·42–12·82)	27·40% (24·23–30·53)	8·49% (7·53–9·55)	2·28% (2·11–2·47)	23·82% (17·79–30·49)	40·84% (36·74–46·42)
	2030	12·46% (8·84–17·73)	28·14% (1·96–48·95)	7·20% (3·46–10·47)	2·18%* (1·50–2·79)	27·23% (19·24–37·81)	39·39% (34·71–44·04)
	Kuwait						
	2012	6·88% (6·46–7·32)	34·43% (28·55–40·61)	4·93% (4·43–5·44)	2·09% (1·95–2·25)	41·58% (32·92–50·28)	25·72% (21·50–31·89)
	2021	7·05% (6·62–7·53)	38·96% (32·64–44·94)	4·36% (3·87–4·87)	1·91% (1·78–2·06)	52·57% (43·30–61·88)	27·01% (22·37–34·33)
	2030	8·51% (4·22–16·88)	40·11% (28·74–56·61)	3·96% (1·80–6·11)	1·88%* (1·14–2·73)	55·64% (46·14–65·45)	24·63% (19·89–29·90)
	Lebanon						
	2012	7·86% (7·33–8·39)	25·15% (20·48–29·85)	16·02% (14·75–17·20)	4·42% (4·13–4·73)	23·02% (17·92–29·23)	23·80% (21·13–26·40)
	2021	7·80% (7·29–8·26)	28·98% (22·89–35·23)	14·19% (12·88–15·53)	3·83% (3·56–4·15)	32·42% (25·51–40·42)	23·96% (21·07–26·96)
	2030	7·81% (5·60–10·13)	30·30% (7·87–54·20)	12·00% (5·95–16·42)	3·67% (2·69–4·80)	35·71% (26·03–49·26)	22·15% (17·11–27·76)
	Libya						
	2012	5·95% (5·55–6·35)	41·11% (34·14–47·36)	19·73% (18·37–21·12)	5·19% (4·85–5·56)	27·56% (20·61–35·07)	31·65% (26·02–40·39)
	2021	5·98% (5·53–6·46)	44·62% (38·40–50·61)	19·57% (18·01–21·13)	5·25% (4·91–5·65)	38·06% (29·31–46·58)	32·86% (27·53–40·69)
	2030	5·73% (4·53–7·85)	47·90% (18·73–67·07)	17·80% (10·21–22·50)	5·15% (3·59–6·87)	39·80% (31·92–49·66)	31·74% (25·85–37·45)
	Morocco						
	2012	9·77% (9·17–10·41)	32·22% (27·46–37·12)	16·76% (15·60–17·85)	4·72% (4·45–5·01)	26·63% (21·07–33·12)	37·15% (30·57–46·36)
	2021	9·14% (8·57–9·71)	37·87% (32·01–43·19)	13·60% (12·31–14·85)	3·20% (3·00–3·41)	33·77% (26·33–42·25)	34·79% (28·35–44·24)
	2030	8·59% (6·14–15·09)	38·66% (9·70–61·74)	9·89%* (5·42–13·15)	2·95%* (2·18–3·68)	40·43% (30·07–52·30)	31·39% (25·82–36·80)
	Oman						
	2012	8·85% (8·27–9·46)	54·18% (50·05–57·96)	13·07% (12·50–13·66)	7·74% (7·43–8·06)	25·91% (19·14–33·51)	34·55% (26·56–45·89)
	2021	8·86% (8·29–9·47)	57·71% (54·05–61·08)	12·93% (11·94–14·02)	7·53% (7·16–7·92)	36·82% (28·60–45·69)	32·85% (25·36–43·02)
	2030	8·87% (6·56–14·78)	59·74% (41·25–73·38)	11·77% (6·64–15·25)	6·94% (5·19–8·70)	40·85% (30·89–54·60)	30·82% (25·15–37·34)
	Palestine						
	2012	8·76% (8·22–9·38)	24·77% (23·15–26·34)	11·01% (10·16–11·87)	3·15% (3·00–3·32)	18·75% (15·34–22·84)	25·52% (23·04–28·24)
	2021	8·57% (8·03–9·23)	28·19% (23·92–32·30)	8·54% (7·69–9·52)	1·98% (1·85–2·11)	26·14% (20·61–32·78)	24·57% (21·17–29·09)
	2030	8·12% (6·55–10·55)	29·56% (5·61–56·47)	7·04% (3·08–10·63)	1·77%* (1·24–2·43)	30·70% (21·82–40·17)	22·71% (18·45–27·20)
	Qatar						
	2012	12·33% (11·56–13·16)	32·37% (27·26–37·83)	9·70% (8·63–10·73)	2·11% (1·97–2·25)	35·89% (27·50–44·93)	32·08% (24·83–43·60)
	2021	12·07% (11·31–12·83)	36·81% (31·07–42·67)	8·35% (7·39–9·35)	1·69% (1·58–1·81)	46·45% (37·04–56·04)	30·50% (23·93–41·37)
	2030	11·41% (4·89–27·59)	40·38% (18·19–61·87)	7·45% (3·51–10·91)	1·62%* (1·11–2·18)	52·38% (42·34–61·71)	28·28% (22·52–34·37)
	Saudi Arabia						
	2012	6·13% (5·64–6·62)	42·43% (36·31–48·24)	9·48% (8·55–10·48)	6·86% (6·44–7·30)	37·18% (28·80–46·56)	34·22% (27·82–42·93)
	2021	6·04% (5·59–6·51)	47·40% (40·90–52·99)	8·30% (7·34–9·37)	5·33% (4·93–5·78)	49·67% (39·90–59·19)	33·12% (26·73–42·94)
	2030	5·81% (4·80–7·01)	50·67% (25·27–69·18)	6·91% (3·18–9·88)	5·05% (3·53–6·65)	56·46% (45·95–65·90)	31·67% (25·47–38·31)
	Sudan						
	2012	11·72% (11·03–12·48)	47·20% (45·78–48·68)	34·74% (33·23–36·40)	15·24% (14·51–15·98)	10·89% (8·08–14·15)	46·63% (37·93–59·14)
	2021	11·05% (10·39–11·76)	56·05% (52·73–59·21)	29·88% (28·33–31·48)	11·74% (11·08–12·41)	15·46% (11·07–20·28)	44·19% (35·97–56·36)
	2030	10·37% (7·83–14·97)	61·82% (33·85–77·45)	25·77% (17·59–29·72)	10·81% (8·98–12·46)	20·07% (15·36–26·39)	41·64% (35·46–48·20)
	Syria						
	2012	10·19% (9·48–10·85)	29·84% (26·53–33·69)	30·25% (28·92–31·54)	11·93% (11·28–12·57)	37·86% (33·20–42·86)	36·71% (30·02–46·86)
	2021	10·16% (9·54–10·88)	33·83% (28·18–39·14)	28·12% (26·55–29·73)	10·93% (10·28–11·56)	50·13% (42·59–58·07)	35·43% (29·18–44·85)
	2030	9·92% (7·36–13·11)	35·75% (8·19–57·73)	26·13%* (17·79–31·73)	10·85% (8·57–12·46)	52·59% (44·35–61·09)	34·29% (27·24–40·86)
	Tunisia						
	2012	7·18% (6·66–7·72)	10·29% (8·45–12·27)	10·56% (9·92–11·29)	3·98% (3·78–4·19)	21·40% (17·28–26·05)	27·56% (24·07–32·25)
	2021	7·26% (6·72–7·84)	12·17% (9·08–15·57)	9·34% (8·50–10·22)	3·15% (2·98–3·36)	28·97% (24·38–34·30)	26·70% (22·91–31·91)
	2030	7·10% (5·27–9·01)	12·52% (0·22–36·40)	7·41% (3·95–10·42)	3·05% (2·14–3·87)	32·70% (25·03–42·35)	24·41% (18·66–29·52)
	Türkiye						
	2012	10·71% (10·01–11·41)	24·72% (20·49–28·97)	10·58% (9·63–11·59)	1·52% (1·43–1·61)	23·18% (17·60–29·31)	31·86% (27·54–37·43)
	2021	10·65% (9·93–11·38)	27·48% (22·46–32·21)	7·72% (6·82–8·73)	1·41% (1·33–1·49)	32·09% (24·76–39·65)	30·75% (25·43–38·44)
	2030	10·04% (8·44–12·63)	30·41% (3·28–57·27)	5·76%* (2·69–8·36)	1·34%* (0·94–1·77)	37·68% (28·91–48·42)	27·56% (22·01–33·50)
	United Arab Emirates						
	2012	9·34% (8·68–9·94)	46·93% (42·54–51·29)	14·44% (13·00–15·87)	6·47% (6·02–6·92)	46·51% (37·48–55·90)	54·27% (39·65–71·69)
	2021	9·30% (8·67–9·98)	49·19% (44·75–53·26)	13·17% (11·77–14·56)	5·18% (4·79–5·62)	58·04% (49·12–66·70)	50·30% (37·38–67·43)
	2030	8·92% (6·70–12·16)	51·56% (31·94–65·71)	11·82% (5·89–15·82)	5·08% (3·71–6·28)	59·75% (51·87–68·67)	47·76% (40·45–54·44)
	Yemen						
	2012	14·20% (13·35–15·10)	10·12% (9·16–11·09)	47·82% (45·75–50·08)	16·05% (15·70–16·41)	21·62% (16·13–27·74)	62·25% (57·61–66·91)
	2021	13·48% (12·66–14·37)	12·62% (10·09–15·51)	43·73% (41·29–46·65)	15·46% (14·77–16·13)	28·50% (21·37–36·20)	62·15% (56·99–67·67)
	2030	12·77% (5·04–39·02)	12·85% (4·64–31·07)	39·90% (29·62–44·41)	15·17% (12·99–17·28)	32·12% (25·17–39·97)	60·77% (54·62–67·15)
**South Asia**							
**2012**		**23·44% (23·14–23·74)**	**48·19% (47·29–48·99)**	**42·84% (42·50–43·19)**	**19·17% (19·04–19·30)**	**8·89% (6·91–11·06)**	**55·21% (53·66–56·83)**
**2021**		**21·83% (21·54–22·15)**	**53·85% (52·12–55·45)**	**35·32% (34·92–35·75)**	**14·39% (14·23–14·54)**	**11·22% (8·81–14·06)**	**54·51% (52·55–56·51)**
**2030**		**20·39% (18·79–22·07)**	**55·25% (34·66–69·78)**	**29·11% (18·90–33·12)**	**13·82% (11·95–15·57)**	**13·66% (10·79–17·07)**	**50·72% (48·30–53·49)**
	Bangladesh						
	2012	26·54% (25·07–28·10)	53·69% (52·38–55·04)	37·51% (36·36–38·92)	14·47% (14·22–14·74)	4·37% (3·12–6·01)	44·72% (37·21–54·16)
	2021	24·29% (22·92–25·74)	61·71% (58·89–64·43)	30·34% (28·84–32·00)	10·43% (9·92–10·92)	5·72% (4·03–7·89)	44·39% (35·00–57·32)
	2030	20·82% (17·26–23·88)	64·19% (41·45–78·01)	23·40%* (14·42–29·66)	9·91% (7·61–11·84)	7·30% (5·16–10·32)	39·81% (34·29–45·22)
	Bhutan						
	2012	8·06% (7·43–8·66)	47·50% (44·20–50·85)	28·49% (26·65–30·51)	5·88% (5·55–6·24)	12·48% (9·22–16·76)	58·17% (48·94–69·80)
	2021	7·42% (6·90–8·00)	50·14% (45·08–54·84)	21·57% (19·53–23·36)	4·16% (3·91–4·43)	15·30% (10·96–20·17)	56·56% (45·47–69·89)
	2030	6·73% (5·28–8·03)	52·69% (25·23–70·67)	14·93%* (8·48–19·87)	3·81% (2·45–5·14)	21·42% (16·45–27·47)	51·17% (45·32–57·19)
	India						
	2012	23·80% (23·51–24·09)	52·14% (50·89–53·21)	43·74% (43·43–44·06)	20·80% (20·70–20·91)	9·52% (6·84–12·45)	56·78% (55·21–58·36)
	2021	22·46% (22·13–22·80)	56·73% (54·42–58·90)	35·75% (35·40–36·15)	15·58% (15·44–15·73)	12·33% (9·00–16·21)	55·57% (53·53–57·70)
	2030	21·20% (19·29–23·18)	58·37% (37·40–72·81)	29·40% (18·45–33·53)	14·99% (13·13–16·78)	15·44% (11·75–19·94)	51·94% (49·53–54·19)
	Nepal						
	2012	18·34% (17·36–19·40)	62·71% (60·93–64·42)	39·63% (37·08–42·53)	10·72% (10·22–11·23)	10·37% (8·13–12·95)	47·55% (40·82–55·99)
	2021	16·24% (15·27–17·23)	65·14% (62·22–67·64)	32·67% (30·62–35·10)	9·07% (8·61–9·55)	13·59% (10·65–17·06)	50·39% (40·98–62·66)
	2030	14·22% (12·26–16·11)	66·94% (46·25–79·59)	23·22% (14·16–30·46)	8·26% (5·59–11·05)	19·18% (14·12–25·53)	45·33% (40·35–50·38)
	Pakistan						
	2012	20·55% (19·68–21·41)	27·49% (26·35–28·66)	42·13% (40·76–43·46)	15·62% (15·03–16·20)	8·44% (6·53–10·67)	54·38% (52·14–57·73)
	2021	19·04% (18·28–19·85)	39·97% (37·00–42·95)	36·18% (34·70–37·80)	12·14% (11·58–12·73)	9·19% (6·66–12·14)	56·23% (49·58–63·92)
	2030	17·78% (15·72–20·06)	38·10% (13·91–57·35)	31·20% (19·64–36·56)	11·69% (9·52–13·69)	9·10% (6·36–12·53)	52·04% (46·08–58·34)
**Southeast Asia, east Asia, and Oceania**							
**2012**		**6·78% (6·71–6·84)**	**45·93% (43·44–48·14)**	**18·87% (18·22–19·54)**	**5·63% (5·54–5·74)**	**12·77% (12·04–13·53)**	**22·07% (21·59–22·58)**
**2021**		**6·81% (6·74–6·88)**	**50·13% (47·84–52·19)**	**16·13% (15·54–16·74)**	**4·51% (4·43–4·60)**	**16·76% (15·81–17·75)**	**22·02% (21·24–22·87)**
**2030**		**6·79% (6·20–7·42)**	**52·00% (31·69–66·11)**	**15·43% (9·93–18·80)**	**5·06% (3·96–6·16)**	**20·30% (16·64–24·45)**	**20·27% (17·53–23·12)**
East Asia							
2012		5·08% (5·00–5·17)	52·17% (47·87–56·06)	9·75% (8·64–10·91)	2·29% (2·14–2·45)	15·01% (13·98–16·04)	17·44% (16·93–17·97)
2021		4·94% (4·86–5·02)	55·01% (51·06–58·66)	8·23% (7·24–9·24)	1·72% (1·59–1·84)	20·21% (19·04–21·47)	15·97% (15·43–16·53)
2030		5·04% (3·84–6·10)	57·69% (40·37–68·28)	6·58%* (3·39–9·10)	1·68%* (1·13–2·29)	26·53% (20·73–32·23)	13·65% (10·56–16·53)
	China						
	2012	5·05% (4·96–5·13)	51·83% (47·40–55·83)	9·44% (8·29–10·64)	2·22% (2·06–2·38)	14·57% (13·55–15·66)	17·27% (16·76–17·80)
	2021	4·90% (4·82–4·99)	54·74% (50·66–58·51)	8·02% (7·00–9·06)	1·66% (1·53–1·79)	19·71% (18·50–21·07)	15·74% (15·19–16·30)
	2030	5·02% (3·77–6·12)	57·43% (39·87–68·15)	6·33%* (3·19–8·87)	1·61%* (1·08–2·21)	26·03% (20·27–31·84)	13·36% (10·20–16·28)
	North Korea						
	2012	5·59% (5·20–6·01)	61·27% (59·17–63·57)	26·12% (25·14–27·12)	4·80% (4·52–5·08)	25·63% (19·17–33·15)	27·23% (24·27–30·16)
	2021	5·30% (4·92–5·70)	62·77% (60·54–64·97)	20·77% (19·51–22·11)	3·57% (3·33–3·85)	33·10% (25·88–40·66)	26·74% (22·88–31·62)
	2030	5·36% (4·19–7·33)	63·53% (50·72–73·84)	17·67%* (10·82–21·85)	3·41% (2·43–4·13)	35·19% (27·70–43·30)	26·09% (22·40–29·88)
	Taiwan (province of China)						
	2012	6·65% (6·22–7·11)	61·71% (58·22–64·93)	2·64% (2·37–2·91)	3·08% (2·87–3·30)	30·95% (23·71–38·28)	16·76% (14·15–20·63)
	2021	6·57% (6·10–7·10)	63·22% (59·86–66·30)	2·47% (2·20–2·76)	2·82% (2·62–3·05)	39·22% (30·91–47·86)	16·59% (13·95–20·37)
	2030	5·83% (4·76–7·20)	64·49% (51·92–74·44)	2·34% (1·08–3·44)	2·83%* (1·69–4·13)	42·65% (34·25–52·84)	15·67% (12·17–19·26)
Oceania							
2012		12·25% (11·65–12·82)	63·76% (61·84–65·66)	38·77% (37·62–40·10)	12·60% (12·05–13·12)	17·74% (15·09–20·90)	50·24% (41·99–60·56)
2021		12·34% (11·72–12·96)	64·38% (61·95–66·53)	38·02% (36·47–39·94)	11·05% (10·52–11·58)	19·44% (16·25–23·03)	50·83% (41·33–61·76)
2030		12·01% (9·51–16·49)	65·95% (47·80–79·81)	36·88% (27·59–41·50)	10·95% (8·67–12·98)	19·91% (15·33–25·23)	48·48% (43·49–53·61)
	American Samoa						
	2012	9·77% (9·11–10·50)	58·19% (53·17–62·59)	11·24% (9·86–12·60)	2·68% (2·48–2·91)	55·91% (47·13–64·82)	35·97% (29·65–44·56)
	2021	9·62% (8·98–10·29)	60·60% (55·85–64·54)	8·36% (7·30–9·61)	2·51% (2·32–2·71)	61·32% (52·52–69·90)	35·65% (29·34–44·63)
	2030	9·92% (7·69–12·70)	61·96% (42·41–75·93)	7·71%* (3·92–10·51)	2·52%* (1·76–3·30)	63·36% (55·89–70·88)	35·00% (29·80–40·87)
	Cook Islands						
	2012	9·06% (8·46–9·65)	61·19% (56·60–65·34)	8·25% (7·20–9·43)	1·70% (1·56–1·84)	67·05% (58·52–74·88)	30·86% (25·10–38·98)
	2021	9·00% (8·43–9·59)	63·27% (59·04–66·90)	5·96% (5·12–6·89)	1·57% (1·45–1·69)	72·04% (64·37–79·32)	29·29% (24·00–36·77)
	2030	8·57% (6·95–11·16)	64·10% (48·12–75·65)	5·31%* (2·05–7·74)	1·52%* (1·05–2·05)	76·03% (68·10–83·39)	28·34% (23·41–33·52)
	Federated States of Micronesia						
	2012	10·17% (9·48–10·85)	53·25% (47·38–58·67)	16·09% (14·38–17·93)	4·62% (4·28–4·99)	41·83% (32·85–50·84)	41·35% (33·85–51·23)
	2021	9·95% (9·28–10·61)	56·21% (50·67–61·09)	12·18% (10·56–13·75)	4·28% (3·98–4·62)	48·39% (39·17–57·53)	40·37% (33·38–50·40)
	2030	10·01% (7·37–13·71)	57·96% (36·10–72·99)	10·95% (6·65–14·42)	4·23% (3·07–5·47)	52·56% (43·73–60·75)	38·07% (32·09–44·44)
	Fiji						
	2012	9·10% (8·55–9·69)	56·62% (51·69–60·97)	7·09% (6·21–8·15)	6·48% (6·13–6·88)	33·13% (24·98–42·88)	38·53% (34·61–43·77)
	2021	8·94% (8·39–9·47)	58·86% (53·89–62·73)	6·80% (6·12–7·55)	5·01% (4·76–5·28)	39·56% (30·81–49·31)	37·34% (32·51–44·26)
	2030	9·25% (7·71–11·55)	60·19% (41·69–73·68)	6·20% (3·20–8·89)	4·86% (3·64–5·97)	43·47% (35·33–53·25)	36·49% (31·65–41·41)
	Guam						
	2012	9·29% (8·71–9·94)	60·64% (55·87–64·56)	7·90% (6·88–8·99)	1·67% (1·54–1·80)	55·19% (46·60–64·36)	31·74% (25·64–40·17)
	2021	9·24% (8·66–9·87)	62·41% (57·85–66·11)	6·12% (5·32–7·04)	1·67% (1·54–1·80)	58·47% (50·04–66·92)	32·08% (25·95–40·51)
	2030	9·64% (7·45–11·67)	62·62% (43·06–75·63)	5·81% (2·40–8·49)	1·64%* (1·12–2·15)	61·31% (51·69–70·39)	32·03% (26·77–37·20)
	Kiribati						
	2012	11·17% (10·41–11·92)	52·18% (46·63–57·30)	14·54% (12·89–16·25)	4·62% (4·34–4·90)	43·50% (34·24–53·45)	47·37% (38·56–59·72)
	2021	10·93% (10·20–11·66)	54·78% (49·70–59·52)	13·81% (12·36–15·36)	4·19% (3·94–4·46)	50·90% (41·56–59·72)	46·51% (37·71–58·28)
	2030	10·68% (7·99–14·32)	56·29% (33·52–71·42)	12·06% (7·68–15·32)	4·05% (3·30–4·83)	54·64% (46·07–63·85)	44·19% (37·98–50·27)
	Marshall Islands						
	2012	12·78% (12·04–13·58)	51·24% (44·97–57·38)	13·41% (11·89–15·00)	0·35% (0·32–0·39)	42·17% (33·70–51·47)	36·43% (31·38–43·01)
	2021	12·39% (11·69–13·15)	53·37% (46·25–59·68)	11·67% (10·17–13·07)	0·35% (0·33–0·38)	48·55% (39·15–57·14)	35·45% (30·71–42·47)
	2030	12·36% (9·36–16·73)	55·32% (30·30–71·35)	9·93% (5·85–13·10)	0·34%* (0·24–0·45)	54·60% (45·35–62·87)	33·56% (27·99–39·85)
	Nauru						
	2012	9·94% (9·25–10·66)	51·92% (45·95–57·04)	19·31% (17·89–20·59)	0·97% (0·89–1·05)	57·47% (48·95–66·77)	45·97% (36·92–58·10)
	2021	9·54% (8·88–10·24)	56·24% (50·99–60·82)	15·14% (13·79–16·55)	0·68% (0·63–0·74)	64·05% (55·42–71·93)	42·48% (34·28–53·79)
	2030	10·03% (7·05–14·01)	57·79% (37·45–72·49)	13·23% (6·29–18·53)	0·67%* (0·44–0·93)	68·05% (60·65–76·08)	40·25% (34·09–46·27)
	Niue						
	2012	8·96% (8·36–9·55)	59·87% (55·29–63·86)	10·27% (9·11–11·68)	2·40% (2·21–2·60)	52·60% (42·87–61·91)	37·33% (30·32–48·00)
	2021	8·93% (8·36–9·49)	61·90% (57·64–65·73)	7·71% (6·70–8·84)	2·26% (2·09–2·45)	59·20% (49·89–68·23)	36·32% (29·45–46·41)
	2030	8·99% (6·66–12·81)	63·19% (45·87–75·93)	6·66% (2·79–9·71)	2·19%* (1·52–2·96)	64·09% (55·77–72·79)	34·63% (29·26–39·95)
	Northern Mariana Islands						
	2012	9·14% (8·47–9·81)	61·01% (56·58–64·84)	8·78% (7·63–9·96)	1·82% (1·67–1·99)	59·40% (50·19–68·02)	34·66% (27·63–46·03)
	2021	9·07% (8·42–9·69)	62·96% (58·58–66·78)	6·18% (5·34–7·09)	1·72% (1·58–1·85)	65·20% (56·62–73·15)	32·87% (26·28–42·45)
	2030	9·70% (8·07–11·58)	63·77% (45·58–76·56)	5·82%* (2·45–8·57)	1·72%* (1·19–2·29)	65·75% (56·57–75·30)	32·30% (27·28–37·48)
	Palau						
	2012	9·19% (8·55–9·84)	60·63% (56·00–64·68)	10·44% (9·19–11·83)	2·44% (2·24–2·65)	56·60% (47·94–65·54)	37·65% (30·56–47·45)
	2021	9·12% (8·49–9·75)	62·14% (57·89–65·78)	7·79% (6·72–8·95)	2·24% (2·06–2·42)	62·29% (54·05–70·39)	36·99% (30·22–46·84)
	2030	8·98% (5·97–14·58)	63·19% (43·98–75·60)	7·04%* (3·25–10·21)	2·19%* (1·55–2·93)	65·52% (56·89–74·18)	35·74% (30·16–41·16)
	Papua New Guinea						
	2012	12·99% (12·24–13·72)	65·07% (62·62–67·45)	44·82% (43·37–46·47)	14·47% (13·76–15·12)	13·36% (10·10–17·34)	53·33% (42·37–67·04)
	2021	12·98% (12·24–13·69)	65·08% (61·98–67·64)	42·81% (40·95–45·13)	12·29% (11·65–12·91)	15·50% (11·72–19·68)	53·74% (42·00–67·70)
	2030	12·48% (9·59–17·48)	66·44% (47·51–80·57)	40·75% (30·51–45·75)	11·96% (9·51–14·12)	16·35% (11·66–21·77)	50·73% (44·98–56·94)
	Samoa						
	2012	7·41% (6·87–7·99)	62·82% (58·66–66·78)	4·79% (4·24–5·38)	5·15% (4·83–5·48)	55·54% (46·75–63·67)	27·41% (24·39–30·61)
	2021	7·11% (6·61–7·64)	64·14% (59·42–68·04)	4·37% (3·80–4·98)	4·76% (4·45–5·09)	61·73% (53·15–69·45)	27·15% (23·93–30·86)
	2030	7·04% (4·80–9·31)	65·20% (49·72–76·08)	4·09% (1·92–5·85)	4·74% (2·98–6·66)	64·22% (56·49–71·70)	26·24% (21·07–31·71)
	Solomon Islands						
	2012	10·19% (9·51–10·85)	72·27% (69·71–74·72)	28·12% (27·24–29·02)	7·04% (6·69–7·42)	20·31% (14·71–26·96)	46·01% (37·25–57·12)
	2021	9·79% (9·16–10·50)	73·09% (70·32–75·57)	25·30% (24·05–26·55)	7·03% (6·60–7·46)	24·48% (18·26–31·81)	44·47% (36·41–55·32)
	2030	9·60% (7·21–13·01)	74·25%* (62·30–82·63)	22·55% (15·10–27·72)	6·78% (4·88–8·95)	27·98% (21·09–35·93)	41·94% (35·60–48·34)
	Tokelau						
	2012	9·15% (8·47–9·82)	57·64% (53·01–62·11)	11·71% (10·35–13·17)	3·05% (2·82–3·30)	51·16% (42·52–60·96)	39·56% (32·44–50·02)
	2021	9·05% (8·43–9·67)	60·08% (55·23–64·27)	8·32% (7·25–9·51)	2·50% (2·32–2·69)	57·86% (48·72–66·35)	37·74% (30·77–47·50)
	2030	8·91% (6·74–12·16)	61·49% (41·95–75·14)	6·94% (3·36–9·99)	2·48%* (1·77–3·31)	63·27% (55·97–71·84)	35·42% (30·14–41·63)
	Tonga						
	2012	4·57% (4·19–4·95)	55·44% (50·23–60·02)	5·81% (5·04–6·62)	3·54% (3·25–3·86)	65·33% (56·83–73·50)	36·81% (30·50–45·83)
	2021	4·49% (4·12–4·90)	58·20% (53·65–62·43)	3·20% (2·75–3·75)	2·23% (2·03–2·43)	71·77% (64·00–78·52)	35·77% (29·88–45·36)
	2030	4·54% (3·29–6·18)	59·79% (40·06–73·94)	2·85%* (1·41–4·02)	2·22%* (1·53–3·04)	74·25% (65·03–82·75)	33·96% (28·40–39·71)
	Tuvalu						
	2012	6·49% (6·02–6·98)	53·40% (47·86–58·28)	8·59% (7·60–9·70)	3·14% (2·88–3·40)	42·03% (33·70–51·06)	42·98% (34·83–54·78)
	2021	6·44% (5·99–6·94)	56·40% (50·79–60·78)	6·71% (5·83–7·68)	3·28% (3·05–3·53)	49·48% (40·58–58·88)	41·47% (33·22–53·94)
	2030	6·61% (4·97–8·80)	58·33% (36·52–73·18)	5·91% (3·37–7·72)	3·21% (2·28–4·19)	58·93% (50·59–67·11)	39·16% (33·61–44·91)
	Vanuatu						
	2012	9·57% (8·96–10·18)	40·20% (35·32–44·58)	24·61% (23·47–25·70)	5·01% (4·70–5·36)	13·97% (10·64–18·17)	52·77% (42·63–65·74)
	2021	9·21% (8·62–9·80)	43·44% (37·55–48·83)	22·02% (20·54–23·44)	4·38% (4·07–4·71)	17·49% (13·02–22·99)	51·47% (40·91–64·71)
	2030	8·73% (6·40–11·93)	45·32% (16·79–65·33)	20·68% (13·73–24·93)	4·20% (2·92–5·55)	20·49% (15·15–27·79)	48·90% (42·53–54·63)
**Southeast Asia**							
2012		8·79% (8·68–8·91)	37·48% (36·47–38·57)	30·58% (30·26–30·89)	9·95% (9·83–10·07)	9·53% (8·38–10·71)	32·16% (30·99–33·38)
2021		8·43% (8·32–8·54)	43·55% (41·89–45·04)	26·22% (25·79–26·68)	8·11% (7·98–8·23)	11·26% (9·79–12·86)	32·47% (30·44–34·63)
2030		8·11% (7·49–9·05)	46·25% (24·07–63·62)	22·62% (15·06–27·17)	7·89% (6·34–9·43)	14·54% (12·10–17·58)	30·65% (27·53–33·83)
	Cambodia						
	2012	9·71% (9·04–10·44)	63·87% (62·11–65·53)	37·86% (36·42–39·52)	10·57% (10·30–10·86)	4·90% (3·70–6·35)	42·93% (39·67–47·26)
	2021	9·35% (8·69–10·03)	65·58% (62·69–68·22)	31·34% (29·66–33·14)	8·35% (7·93–8·80)	5·99% (4·28–7·99)	41·70% (36·43–49·40)
	2030	8·06% (5·95–11·10)	69·96% (54·85–79·59)	23·97% (14·91–29·69)	7·51% (5·23–9·21)	8·14% (5·81–11·03)	37·11% (32·08–41·82)
	Indonesia						
	2012	7·31% (7·17–7·46)	42·44% (40·72–44·10)	36·17% (35·87–36·44)	12·58% (12·43–12·73)	10·17% (7·85–12·62)	26·03% (24·62–27·60)
	2021	7·12% (6·97–7·26)	50·16% (47·55–52·58)	31·89% (31·48–32·32)	10·27% (10·13–10·41)	11·87% (8·47–15·58)	28·09% (24·09–32·72)
	2030	6·88% (6·18–8·63)	53·15% (31·05–68·27)	27·98% (18·37–33·12)	10·03% (8·60–11·41)	15·45% (11·67–20·41)	26·82% (23·90–30·06)
	Laos						
	2012	8·70% (8·09–9·38)	40·75% (38·91–42·64)	41·74% (39·37–44·65)	7·90% (7·54–8·26)	5·68% (4·12–7·69)	43·79% (39·97–48·64)
	2021	7·98% (7·42–8·53)	46·87% (44·06–49·92)	32·86% (30·85–35·15)	6·70% (6·33–7·12)	6·93% (4·87–9·33)	47·97% (41·39–55·68)
	2030	7·01% (6·20–9·21)	50·25% (13·31–72·52)	27·85% (18·77–33·48)	6·00% (4·03–7·96)	9·94% (7·31–13·69)	44·04% (39·98–48·61)
	Malaysia						
	2012	10·53% (10·05–11·02)	44·86% (38·35–50·79)	19·21% (18·21–20·12)	9·45% (8·98–9·95)	17·31% (12·27–23·08)	77·65% (64·75–89·85)
	2021	10·45% (9·84–11·05)	47·78% (41·16–53·13)	18·03% (16·93–19·23)	8·46% (7·99–8·93)	21·41% (15·65–27·57)	64·60% (48·82–81·31)
	2030	10·21% (8·44–12·60)	49·96% (25·37–67·43)	15·34% (9·88–18·84)	8·16% (6·00–9·90)	24·40% (18·70–30·90)	61·76% (56·26–67·28)
	Maldives						
	2012	9·70% (9·17–10·27)	47·71% (44·34–50·85)	20·06% (18·72–21·53)	9·44% (8·82–10·06)	8·91% (6·58–11·62)	54·91% (48·87–63·04)
	2021	9·93% (9·28–10·58)	50·74% (45·87–55·14)	17·08% (15·45–18·65)	7·99% (7·40–8·62)	11·85% (8·54–15·70)	60·00% (51·21–70·66)
	2030	9·81% (8·35–12·43)	52·84% (23·56–70·03)	13·22% (7·83–17·48)	7·44% (5·93–9·19)	15·77% (11·08–21·57)	54·83% (48·27–60·92)
	Mauritius						
	2012	15·52% (14·80–16·27)	45·69% (39·73–51·34)	18·07% (16·55–19·67)	8·40% (7·79–9·08)	15·13% (10·72–20·33)	35·23% (28·77–44·63)
	2021	15·22% (14·39–16·09)	49·06% (43·34–54·59)	16·62% (15·19–18·14)	7·54% (6·99–8·12)	18·88% (13·75–24·93)	35·10% (28·54–44·68)
	2030	14·14% (11·77–16·93)	51·05% (27·09–67·60)	15·32% (8·32–20·23)	7·17% (5·65–8·63)	22·11% (16·21–29·81)	34·17% (28·32–40·22)
	Myanmar						
	2012	9·47% (8·83–10·14)	29·87% (27·76–32·00)	31·62% (30·35–33·21)	7·81% (7·39–8·28)	7·44% (5·20–10·18)	54·07% (48·26–61·11)
	2021	8·73% (8·11–9·35)	41·69% (37·47–45·70)	24·51% (23·12–25·98)	5·84% (5·46–6·24)	9·08% (6·26–12·70)	57·28% (48·94–67·73)
	2030	8·24% (7·05–10·78)	46·37% (16·93–67·10)	19·08% (11·79–23·68)	5·27% (3·51–7·00)	12·98% (9·12–17·39)	54·73% (50·64–59·34)
	Philippines						
	2012	11·79% (11·67–11·90)	36·64% (33·41–39·58)	30·17% (29·25–31·20)	8·41% (8·07–8·77)	6·84% (4·75–9·42)	31·66% (29·12–34·22)
	2021	11·01% (10·90–11·12)	40·35% (35·53–44·87)	25·67% (24·40–27·04)	6·97% (6·59–7·38)	8·57% (5·97–11·61)	30·07% (26·88–33·67)
	2030	10·85% (9·76–11·49)	41·89% (17·44–59·45)	22·92% (14·60–28·78)	6·71% (4·86–8·95)	10·52% (7·66–14·09)	27·79% (23·22–32·32)
	Seychelles						
	2012	9·79% (9·10–10·45)	49·09% (43·21–54·22)	10·11% (8·88–11·31)	4·68% (4·31–5·06)	22·01% (16·01–28·66)	35·46% (28·80–45·22)
	2021	9·75% (9·10–10·48)	51·50% (46·07–56·04)	9·33% (8·06–10·66)	4·20% (3·88–4·53)	27·43% (19·95–35·47)	35·05% (28·34–45·19)
	2030	9·39% (7·53–12·24)	53·49% (28·56–69·35)	8·42% (4·27–11·86)	4·10% (2·83–5·54)	30·80% (23·58–37·85)	33·26% (27·99–38·90)
	Sri Lanka						
	2012	15·23% (14·64–15·87)	79·87% (77·24–82·21)	16·53% (15·64–17·41)	14·73% (14·16–15·32)	13·55% (9·78–18·07)	36·19% (31·45–42·87)
	2021	14·42% (13·78–15·08)	80·48% (77·87–82·83)	15·13% (13·97–16·33)	13·01% (12·34–13·66)	17·14% (12·26–22·79)	36·29% (30·31–45·06)
	2030	14·60% (11·01–18·17)	81·68%* (73·43–86·71)	12·12% (6·00–17·51)	12·62% (10·69–14·72)	19·65% (14·35–25·98)	33·72% (27·30–39·46)
	Thailand						
	2012	8·76% (8·22–9·36)	13·29% (11·23–15·46)	14·31% (13·44–15·24)	6·40% (6·04–6·78)	15·32% (12·36–18·66)	23·85% (20·87–27·42)
	2021	8·81% (8·22–9·39)	17·09% (13·70–20·89)	12·62% (11·63–13·70)	6·06% (5·69–6·45)	18·47% (13·77–23·86)	23·14% (20·23–26·36)
	2030	7·96% (6·32–10·32)	19·13% (0·71–51·71)	10·85% (6·16–14·22)	5·82% (4·31–7·43)	22·66% (16·03–30·48)	21·08% (15·88–26·62)
	Timor-Leste						
	2012	12·44% (11·63–13·20)	53·04% (50·80–55·22)	48·11% (46·37–50·16)	19·20% (18·35–19·98)	12·14% (9·23–15·60)	30·15% (26·54–34·91)
	2021	11·78% (11·06–12·49)	55·74% (52·11–58·90)	44·99% (43·21–47·20)	19·15% (18·19–20·05)	14·82% (11·00–19·47)	32·01% (26·47–39·64)
	2030	10·19% (7·54–13·59)	58·81% (36·89–72·69)	40·51% (29·26–45·66)	18·11% (14·89–20·98)	19·48% (14·05–26·46)	28·85% (23·78–34·07)
	Viet Nam						
	2012	5·89% (5·45–6·36)	21·74% (19·17–24·15)	26·10% (25·23–27·00)	6·90% (6·68–7·12)	8·13% (6·62–9·93)	25·71% (23·33–28·44)
	2021	5·66% (5·25–6·14)	25·27% (20·55–29·89)	21·62% (20·28–23·02)	5·39% (5·11–5·69)	9·57% (7·01–12·50)	24·88% (21·90–27·94)
	2030	5·26% (4·37–8·29)	27·56% (2·29–53·22)	16·33% (9·97–21·45)	5·07% (3·55–6·58)	13·68% (9·93–18·44)	22·11% (17·14–26·62)
**Sub-Saharan Africa**							
**2012**		**11·75% (11·64–11·87)**	**35·88% (35·51–36·25)**	**36·03% (35·70–36·38)**	**10·28% (10·21–10·36)**	**15·26% (14·36–16·25)**	**45·19% (43·99–46·39)**
**2021**		**11·16% (11·05–11·27)**	**42·16% (41·41–42·87)**	**31·23% (30·85–31·61)**	**8·03% (7·94–8·14)**	**17·98% (16·66–19·34)**	**45·71% (44·32–47·15)**
**2030**		**10·77% (10·23–11·50)**	**44·62% (23·05–64·43)**	**27·35% (18·90–31·02)**	**7·84% (6·33–9·16)**	**21·24% (18·07–25·29)**	**43·33% (41·20–46·01)**
Central sub-Saharan Africa							
2012		9·11% (8·76–9·45)	39·59% (38·44–40·81)	38·53% (37·45–39·82)	9·54% (9·28–9·80)	17·11% (14·44–19·89)	50·40% (45·46–57·04)
2021		8·46% (8·09–8·85)	47·29% (44·60–49·79)	34·31% (33·18–35·45)	6·76% (6·48–7·04)	21·40% (17·93–25·45)	47·85% (41·73–55·34)
2030		8·01% (7·16–9·30)	52·95% (27·76–69·93)	30·27% (21·40–33·91)	6·33% (4·97–7·55)	24·98% (19·91–31·09)	42·65% (38·27–47·56)
	Angola						
	2012	10·39% (9·77–11·07)	35·30% (32·73–37·99)	38·60% (36·37–41·44)	6·63% (6·24–7·04)	20·72% (15·49–26·65)	54·76% (43·67–68·57)
	2021	9·74% (9·15–10·39)	42·91% (39·08–46·48)	32·82% (31·11–34·69)	4·88% (4·55–5·23)	27·00% (20·56–34·55)	52·00% (40·76–65·94)
	2030	9·05% (7·28–11·50)	47·80% (17·48–65·42)	26·19% (17·45–31·13)	4·28% (2·86–5·58)	35·34% (27·40–44·69)	48·60% (41·45–55·34)
	Central African Republic						
	2012	11·92% (11·20–12·68)	38·20% (36·56–40·03)	38·83% (37·30–40·76)	9·47% (9·01–9·98)	17·88% (13·80–22·41)	38·15% (34·30–43·54)
	2021	11·72% (10·95–12·46)	42·08% (37·54–46·43)	37·22% (35·58–39·25)	7·99% (7·49–8·50)	22·63% (16·67–28·44)	38·26% (33·11–46·04)
	2030	11·66% (9·14–14·87)	49·84% (12·73–73·69)	35·98% (27·40–39·06)	7·74% (5·65–9·66)	20·93% (15·43–27·05)	37·53% (32·50–44·05)
	Congo (Brazzaville)						
	2012	10·73% (10·03–11·42)	24·96% (22·70–27·28)	23·10% (22·09–24·19)	7·55% (7·19–7·94)	15·40% (11·73–19·85)	50·77% (46·70–56·14)
	2021	10·26% (9·55–10·97)	31·90% (27·62–36·51)	19·54% (18·05–20·99)	6·39% (6·02–6·79)	20·07% (14·90–26·19)	49·79% (43·47–58·20)
	2030	10·27% (8·08–12·97)	33·44% (4·41–60·23)	16·87% (10·63–21·16)	6·44% (4·67–8·13)	22·94% (17·14–30·23)	46·11% (39·03–52·96)
	DR Congo						
	2012	8·27% (7·78–8·73)	42·81% (41·37–44·29)	39·82% (38·52–41·45)	10·85% (10·49–11·24)	15·49% (11·94–19·44)	49·57% (43·26–58·64)
	2021	7·49% (6·95–8·07)	50·96% (47·17–54·56)	35·88% (34·44–37·50)	7·53% (7·12–7·94)	18·80% (14·05–24·38)	46·68% (38·90–57·62)
	2030	7·14% (6·29–8·92)	57·42% (34·95–73·64)	32·78% (23·71–36·49)	7·25% (5·86–8·47)	20·22% (14·76–27·65)	40·14% (35·27–46·36)
	Equatorial Guinea						
	2012	10·04% (9·37–10·72)	18·58% (14·05–23·42)	25·75% (24·92–26·64)	3·71% (3·47–3·96)	44·17% (36·61–52·27)	51·30% (41·45–65·91)
	2021	9·44% (8·83–10·09)	22·09% (16·41–28·80)	21·81% (20·20–23·33)	2·94% (2·75–3·15)	54·89% (45·98–63·59)	48·57% (39·65–61·41)
	2030	9·76% (7·58–12·27)	22·69% (1·82–49·27)	17·81% (11·21–23·94)	2·83%* (2·04–3·63)	62·31% (51·63–71·51)	45·91% (37·61–53·37)
	Gabon						
	2012	12·11% (11·44–12·78)	5·61% (4·11–7·40)	18·24% (16·96–19·56)	3·99% (3·73–4·27)	15·95% (13·86–18·23)	58·75% (53·68–65·31)
	2021	11·80% (11·15–12·48)	7·16% (5·05–9·78)	15·31% (13·92–16·81)	3·05% (2·84–3·28)	21·22% (16·73–25·87)	57·37% (50·27–66·63)
	2030	12·04% (9·84–14·58)	7·43% (1·23–21·01)	12·58% (7·24–16·76)	2·96%* (2·00–3·87)	25·31% (16·79–37·30)	55·09% (49·60–59·26)
**Eastern sub-Saharan Africa**							
2012		12·11% (11·90–12·31)	51·67% (50·94–52·34)	37·09% (36·60–37·59)	9·26% (9·16–9·37)	14·16% (13·11–15·32)	35·99% (34·54–37·70)
2021		11·43% (11·24–11·62)	55·84% (54·76–56·90)	31·97% (31·47–32·44)	7·26% (7·16–7·37)	17·23% (15·69–18·82)	36·32% (34·44–38·38)
2030		10·81% (10·36–11·46)	58·80% (40·31–71·87)	27·38% (19·02–31·25)	7·10% (5·61–8·37)	20·32% (16·90–24·09)	33·51% (31·56–35·85)
	Burundi						
	2012	9·88% (9·23–10·52)	74·82% (73·21–76·37)	48·38% (46·47–50·95)	6·63% (6·39–6·87)	8·90% (6·47–11·90)	32·92% (29·37–36·90)
	2021	9·82% (9·15–10·52)	80·38% (78·61–81·97)	47·33% (45·23–50·34)	5·44% (5·18–5·74)	11·14% (7·99–14·96)	37·96% (32·80–45·06)
	2030	9·49% (9·00–10·77)	84·81%* (76·96–89·66)	46·32% (40·48–48·46)	5·11% (3·76–6·52)	12·66% (8·84–16·95)	37·31% (34·21–39·67)
	Comoros						
	2012	15·12% (14·35–15·97)	12·67% (10·80–14·87)	33·86% (32·23–35·61)	12·26% (11·66–12·89)	35·02% (27·45–42·76)	39·59% (31·43–50·45)
	2021	14·38% (13·52–15·29)	14·21% (10·98–17·70)	30·10% (28·42–31·77)	10·42% (9·83–11·06)	41·43% (33·36–50·16)	39·29% (31·49–50·25)
	2030	13·88% (10·17–18·82)	14·14% (1·87–37·31)	24·72% (15·79–29·82)	10·04% (7·86–11·71)	44·43% (37·55–50·65)	36·21% (30·98–41·16)
	Djibouti						
	2012	10·05% (9·47–10·69)	15·43% (12·18–18·84)	32·39% (31·28–33·53)	24·20% (23·28–25·16)	21·99% (16·95–27·66)	38·44% (30·27–50·72)
	2021	9·80% (9·24–10·42)	16·98% (12·81–21·54)	28·59% (27·10–30·11)	19·79% (18·75–20·80)	25·51% (19·31–32·45)	36·73% (29·11–47·41)
	2030	9·56% (7·83–11·98)	16·88% (0·79–44·77)	25·10% (15·31–30·53)	19·18% (17·12–20·96)	31·95% (24·54–40·85)	33·41% (28·54–38·10)
	Eritrea						
	2012	13·17% (12·32–14·08)	57·38% (52·93–61·15)	42·67% (40·00–45·77)	15·13% (14·62–15·63)	7·87% (5·62–10·72)	42·21% (33·55–53·97)
	2021	11·93% (11·17–12·69)	61·11% (56·40–65·19)	37·72% (35·30–40·65)	12·21% (11·57–12·90)	11·01% (7·76–15·33)	41·31% (32·67–53·18)
	2030	11·14% (9·61–13·48)	63·79% (42·59–78·75)	35·27% (25·65–39·37)	11·46% (9·51–13·02)	13·70% (9·95–17·92)	37·78% (33·05–42·19)
	Ethiopia						
	2012	13·34% (12·93–13·78)	55·91% (53·67–57·98)	39·74% (38·70–40·89)	12·83% (12·53–13·15)	8·73% (6·67–11·48)	24·39% (23·47–25·34)
	2021	11·68% (11·31–12·06)	61·26% (58·22–63·79)	33·35% (32·42–34·24)	10·15% (9·87–10·47)	10·27% (7·33–14·01)	23·08% (21·89–24·35)
	2030	10·98% (10·49–11·80)	62·92% (45·90–75·01)	28·30% (18·48–32·45)	9·77% (7·67–11·52)	13·98% (10·16–18·82)	20·61% (18·53–22·80)
	Kenya						
	2012	9·35% (9·25–9·46)	39·89% (37·83–41·96)	30·65% (29·71–31·63)	6·46% (6·30–6·64)	13·29% (10·87–15·79)	24·34% (23·18–25·69)
	2021	8·94% (8·83–9·04)	49·36% (45·57–52·85)	27·45% (26·58–28·33)	5·57% (5·42–5·73)	15·95% (12·01–20·14)	24·79% (23·56–26·19)
	2030	8·77% (8·55–9·19)	54·38% (19·57–74·65)	23·65% (15·70–28·16)	5·46% (4·33–6·49)	17·74% (13·08–23·21)	23·95% (22·87–25·15)
	Madagascar						
	2012	14·81% (13·97–15·61)	48·23% (45·28–51·07)	47·02% (44·39–50·05)	14·22% (13·49–14·98)	10·34% (7·92–13·29)	35·85% (32·36–39·74)
	2021	13·66% (12·87–14·43)	51·87% (46·87–55·93)	42·16% (39·20–45·31)	9·44% (8·79–10·08)	13·28% (9·47–17·83)	35·94% (31·07–43·21)
	2030	12·97% (10·03–16·49)	54·04% (31·53–71·05)	40·03% (31·41–43·23)	8·99% (6·99–10·75)	16·60% (11·88–22·21)	33·75% (28·88–38·10)
	Malawi						
	2012	11·10% (10·38–11·82)	64·47% (63·28–65·68)	35·04% (33·69–36·69)	4·91% (4·67–5·14)	24·18% (19·94–29·05)	59·62% (46·18–74·17)
	2021	10·88% (10·21–11·53)	65·96% (63·65–68·28)	28·60% (27·06–30·00)	3·86% (3·61–4·10)	29·57% (23·44–36·86)	58·55% (44·58–74·89)
	2030	10·68% (9·06–13·37)	70·69%* (55·27–81·40)	23·16% (14·89–27·42)	3·46% (2·39–4·49)	30·97% (22·21–42·65)	53·28% (48·39–58·43)
	Mozambique						
	2012	12·49% (11·65–13·34)	35·30% (33·07–37·82)	38·38% (36·50–40·57)	5·50% (5·24–5·78)	17·66% (15·34–20·51)	53·47% (50·55–56·99)
	2021	11·86% (11·10–12·59)	40·47% (35·56–45·04)	32·68% (30·98–34·62)	4·18% (3·94–4·45)	21·58% (16·77–26·67)	56·84% (52·95–61·61)
	2030	11·17% (10·70–12·56)	44·60% (16·56–63·73)	25·66% (17·01–31·79)	3·72% (2·30–4·74)	27·95% (21·40–35·62)	54·20% (49·04–59·02)
	Rwanda						
	2012	6·73% (6·26–7·20)	84·93% (83·82–86·00)	38·21% (36·42–40·42)	3·61% (3·41–3·82)	19·77% (16·98–22·95)	20·01% (18·45–21·69)
	2021	6·58% (6·14–7·07)	86·50% (85·15–87·83)	33·13% (31·44–35·10)	2·47% (2·32–2·64)	23·80% (18·62–30·24)	19·09% (16·82–21·55)
	2030	6·36% (5·92–7·12)	87·75%* (84·12–90·01)	27·41% (18·00–32·92)	2·15%* (1·44–2·80)	28·67% (22·04–36·23)	16·17% (13·47–18·94)
	Somalia						
	2012	16·66% (15·64–17·59)	10·01% (8·70–11·59)	28·22% (26·79–29·71)	15·38% (14·99–15·79)	10·73% (7·67–14·78)	56·05% (45·54–68·43)
	2021	16·40% (15·48–17·35)	10·75% (8·02–14·02)	26·21% (24·32–28·17)	13·06% (12·61–13·51)	13·34% (9·44–17·77)	55·54% (43·10–69·78)
	2030	16·07% (14·93–17·91)	11·03% (0·17–29·60)	24·63% (16·82–28·91)	12·55% (10·45–14·48)	14·20% (10·78–19·09)	50·60% (43·55–59·51)
	South Sudan						
	2012	11·86% (11·08–12·62)	44·73% (42·00–47·48)	30·00% (28·93–31·03)	23·39% (22·72–24·07)	16·69% (12·04–22·23)	44·05% (34·24–58·63)
	2021	11·99% (11·20–12·72)	42·98% (37·68–47·85)	28·52% (26·92–30·04)	20·91% (20·00–21·79)	19·21% (14·11–25·19)	43·90% (34·53–58·18)
	2030	11·46% (9·99–13·91)	51·89% (15·89–74·90)	23·99% (13·74–31·06)	20·60% (18·49–22·58)	22·05% (16·16–30·32)	41·01% (33·33–51·98)
	Tanzania						
	2012	11·47% (10·82–12·14)	52·99% (51·07–54·78)	36·88% (35·46–38·71)	5·28% (5·07–5·50)	13·95% (10·75–17·67)	48·36% (41·77–57·94)
	2021	10·87% (10·27–11·50)	57·72% (54·66–60·65)	31·57% (30·08–33·06)	4·05% (3·83–4·30)	18·05% (13·22–23·53)	49·80% (40·87–62·53)
	2030	10·24% (9·59–11·90)	62·56% (40·91–76·45)	26·76% (17·37–32·46)	3·56% (2·45–4·58)	20·32% (15·30–25·19)	43·96% (38·48–49·57)
	Uganda						
	2012	10·85% (10·17–11·61)	62·63% (61·15–64·04)	31·93% (30·65–33·35)	5·62% (5·41–5·82)	17·80% (13·84–22·63)	33·59% (30·53–37·20)
	2021	10·22% (9·54–10·94)	67·23% (65·00–69·31)	26·84% (25·41–28·29)	4·36% (4·14–4·57)	22·99% (17·44–29·62)	34·52% (29·72–41·32)
	2030	9·61% (8·94–12·14)	69·24% (53·29–79·57)	21·34% (13·24–26·90)	4·14% (2·93–5·36)	25·95% (18·25–32·98)	31·93% (28·16–35·54)
	Zambia						
	2012	10·83% (10·13–11·58)	65·13% (63·58–66·61)	41·96% (40·56–43·65)	6·56% (6·32–6·81)	32·59% (27·11–38·65)	42·08% (39·60–44·91)
	2021	9·82% (9·19–10·50)	67·74% (65·10–70·15)	31·50% (29·93–32·93)	4·38% (4·15–4·63)	36·60% (29·31–44·93)	40·37% (36·16–45·96)
	2030	9·28% (8·44–11·30)	70·92%* (54·24–82·77)	25·89% (16·19–31·38)	4·24% (3·06–5·42)	39·96% (33·83–47·20)	37·65% (33·20–42·86)
**Southern sub-Saharan Africa**							
2012		11·29% (11·03–11·55)	26·67% (24·13–29·14)	26·42% (25·93–26·92)	5·40% (5·31–5·49)	24·48% (21·93–27·19)	34·61% (32·45–37·29)
2021		10·99% (10·73–11·25)	33·23% (30·24–36·47)	22·71% (22·14–23·26)	4·51% (4·42–4·61)	28·02% (24·00–32·31)	34·36% (31·77–37·60)
2030		11·26% (10·21–12·58)	37·91% (14·08–58·92)	19·70% (12·57–24·69)	4·34% (3·31–5·41)	29·16% (25·39–33·92)	32·86% (30·14–35·60)
	Botswana						
	2012	10·99% (10·25–11·71)	30·44% (25·72–35·37)	29·53% (28·17–30·99)	8·60% (8·17–9·03)	20·36% (14·98–26·21)	36·28% (29·84–45·62)
	2021	10·78% (10·11–11·54)	35·33% (29·55–40·99)	26·29% (24·52–28·04)	7·07% (6·65–7·51)	25·69% (19·28–33·67)	34·49% (27·84–43·43)
	2030	11·50% (9·20–13·48)	37·51% (8·11–62·49)	23·84% (15·43–30·08)	6·88% (5·20–8·44)	29·78% (22·51–37·25)	31·85% (26·14–37·13)
	Eswatini						
	2012	7·61% (7·07–8·14)	36·91% (34·13–39·71)	30·05% (28·59–31·74)	2·24% (2·10–2·37)	27·02% (22·27–32·38)	29·61% (26·70–32·89)
	2021	7·31% (6·82–7·82)	43·35% (38·66–47·56)	24·93% (23·21–26·70)	1·78% (1·67–1·90)	32·41% (25·53–39·94)	28·58% (25·02–33·08)
	2030	7·70% (6·23–9·37)	47·73% (25·05–67·76)	21·56% (13·66–26·60)	1·78%* (1·19–2·47)	33·70% (26·57–43·43)	27·34% (23·24–31·94)
	Lesotho						
	2012	10·88% (10·20–11·64)	54·81% (52·65–56·86)	37·47% (35·36–39·88)	4·80% (4·56–5·04)	14·93% (12·41–17·94)	29·53% (27·67–31·29)
	2021	10·51% (9·88–11·19)	61·39% (58·86–64·06)	32·77% (31·05–34·63)	3·60% (3·37–3·81)	16·65% (12·94–20·79)	28·47% (25·81–31·16)
	2030	10·45% (8·56–13·11)	67·01% (47·42–78·12)	27·88% (18·42–33·00)	3·16% (2·29–4·01)	19·00% (13·69–24·96)	27·48% (23·87–30·96)
	Namibia						
	2012	12·38% (11·55–13·19)	39·70% (37·79–41·73)	24·77% (23·58–25·99)	8·03% (7·61–8·44)	11·52% (8·98–14·35)	33·29% (27·38–41·33)
	2021	11·93% (11·17–12·68)	44·07% (40·52–47·47)	20·37% (18·89–21·87)	6·25% (5·86–6·67)	14·58% (10·87–19·18)	30·30% (24·62–39·11)
	2030	12·16% (10·11–14·33)	49·42% (20·54–68·05)	17·68% (10·83–23·18)	6·03% (4·50–7·64)	16·13% (11·82–21·19)	27·20% (23·76–30·90)
	South Africa						
	2012	11·59% (11·30–11·87)	20·13% (16·25–23·92)	24·18% (23·64–24·76)	5·49% (5·37–5·60)	29·93% (26·01–34·08)	34·58% (31·83–37·92)
	2021	11·42% (11·13–11·72)	26·32% (21·53–31·41)	21·08% (20·41–21·71)	4·76% (4·65–4·88)	34·80% (28·77–41·38)	34·81% (31·32–39·01)
	2030	12·38% (11·20–13·83)	29·71% (7·21–51·79)	18·30% (11·79–22·81)	4·72% (3·67–5·67)	37·27% (32·01–42·55)	33·53% (30·48–36·70)
	Zimbabwe						
	2012	10·79% (10·01–11·49)	36·67% (35·25–38·28)	30·43% (29·09–31·88)	4·74% (4·52–4·98)	13·67% (10·96–16·43)	35·87% (32·43–40·54)
	2021	10·26% (9·62–10·88)	42·88% (40·13–45·67)	25·37% (23·84–26·92)	3·65% (3·43–3·87)	14·66% (11·11–18·70)	34·67% (30·46–41·00)
	2030	9·32% (7·35–12·21)	48·68% (18·00–70·40)	21·44% (13·85–27·36)	3·42% (2·41–4·71)	15·38% (11·83–20·84)	32·69% (28·25–36·79)
**Western sub-Saharan Africa**							
2012		12·26% (12·12–12·40)	22·47% (22·09–22·84)	35·53% (35·03–36·08)	12·05% (11·94–12·16)	14·54% (12·87–16·50)	54·50% (52·64–56·57)
2021		11·70% (11·56–11·86)	30·64% (29·54–31·62)	30·63% (29·94–31·35)	9·45% (9·28–9·65)	16·56% (14·27–19·05)	55·62% (53·86–57·45)
2030		11·37% (10·50–12·58)	32·23% (7·20–58·42)	27·23% (18·76–30·84)	9·07% (7·37–10·58)	20·37% (16·99–25·00)	53·77% (50·30–57·45)
	Benin						
	2012	12·37% (11·57–13·17)	38·77% (37·59–39·94)	40·42% (39·13–41·80)	11·59% (11·12–12·10)	15·49% (11·72–20·23)	59·29% (52·38–68·11)
	2021	12·02% (11·31–12·77)	42·24% (39·80–44·68)	33·66% (32·10–35·32)	8·00% (7·56–8·47)	18·08% (13·18–24·00)	66·39% (56·47–77·00)
	2030	11·91% (9·43–14·85)	45·99% (17·00–62·93)	32·50% (22·78–36·30)	7·72% (5·80–9·37)	22·52% (16·57–29·03)	64·13% (58·14–69·90)
	Burkina Faso						
	2012	10·72% (10·23–11·25)	31·97% (30·84–33·10)	32·06% (31·15–33·07)	15·43% (14·96–15·90)	14·16% (11·25–17·39)	47·01% (42·96–52·56)
	2021	10·32% (9·79–10·87)	40·48% (37·86–42·92)	27·23% (25·84–28·61)	11·45% (10·91–12·02)	15·60% (11·35–20·08)	47·01% (40·99–55·74)
	2030	9·98% (8·11–12·57)	48·14% (9·30–70·38)	21·63% (13·76–26·15)	10·62% (9·21–12·15)	19·37% (14·05–25·05)	47·05% (39·94–52·09)
	Cabo Verde						
	2012	6·68% (6·24–7·12)	46·51% (42·61–50·16)	14·65% (13·25–16·16)	2·60% (2·41–2·78)	12·95% (9·35–17·64)	42·37% (34·29–55·36)
	2021	6·42% (6·00–6·83)	48·96% (44·30–52·65)	12·55% (11·12–13·94)	2·09% (1·94–2·25)	17·16% (12·63–22·99)	42·20% (33·87–54·20)
	2030	5·94% (4·80–7·77)	50·61% (22·17–67·76)	8·69%* (4·05–12·70)	1·98%* (1·25–2·74)	21·36% (16·43–27·74)	38·22% (32·38–44·18)
	Cameroon						
	2012	8·39% (7·82–8·96)	23·72% (21·96–25·29)	32·81% (31·42–34·33)	6·42% (6·09–6·77)	22·37% (18·04–27·23)	39·00% (36·32–41·80)
	2021	7·73% (7·17–8·27)	28·99% (24·87–32·92)	27·47% (25·83–28·95)	4·77% (4·48–5·13)	28·24% (21·95–34·81)	37·86% (33·69–43·40)
	2030	7·56% (6·71–9·19)	31·84% (2·61–57·61)	21·88% (14·24–27·13)	4·53% (3·15–5·74)	33·98% (24·37–48·18)	34·34% (29·11–40·23)
	Chad						
	2012	9·30% (8·69–9·95)	3·38% (2·82–3·99)	36·76% (35·06–38·93)	16·82% (16·12–17·56)	10·64% (7·52–14·36)	57·09% (46·56–69·71)
	2021	8·90% (8·22–9·56)	2·74% (1·98–3·85)	32·64% (30·53–35·43)	15·25% (14·55–15·91)	12·33% (8·90–16·55)	58·06% (47·02–71·09)
	2030	8·71% (7·91–10·72)	2·68% (0·00–10·87)	30·49% (22·35–33·09)	14·72% (12·52–16·70)	14·92% (10·33–19·69)	56·58% (49·72–63·03)
	Côte d'Ivoire						
	2012	14·49% (13·66–15·34)	14·05% (12·49–15·75)	29·08% (27·89–30·42)	8·70% (8·31–9·11)	9·92% (7·92–12·41)	48·57% (45·42–52·55)
	2021	13·28% (12·46–14·15)	21·44% (18·18–25·08)	22·16% (20·78–23·66)	6·11% (5·76–6·44)	11·74% (8·90–15·46)	48·02% (42·45–55·92)
	2030	12·17% (9·37–15·62)	25·94% (0·50–53·49)	18·50% (11·64–24·03)	5·57% (3·96–7·13)	13·92% (9·10–20·75)	43·31% (37·07–49·29)
	The Gambia						
	2012	17·38% (16·34–18·46)	45·66% (43·68–47·68)	22·79% (21·77–23·84)	9·77% (9·29–10·24)	5·59% (4·09–7·51)	56·84% (52·33–62·79)
	2021	15·75% (14·77–16·67)	52·82% (50·13–55·52)	17·80% (16·56–19·20)	6·76% (6·33–7·24)	6·17% (4·28–8·39)	57·11% (50·14–66·66)
	2030	15·30% (12·53–19·17)	56·55% (23·34–74·26)	14·62% (9·55–18·03)	6·54% (4·66–8·36)	7·74% (5·53–10·29)	55·16% (50·68–59·43)
	Ghana						
	2012	9·88% (9·27–10·49)	51·24% (49·32–53·20)	23·54% (22·48–24·59)	9·37% (8·99–9·79)	9·57% (7·52–12·25)	57·65% (48·62–68·59)
	2021	9·05% (8·46–9·66)	51·09% (46·93–54·87)	17·51% (16·11–19·01)	6·55% (6·18–6·93)	12·42% (9·14–16·33)	56·90% (46·75–69·22)
	2030	8·87% (8·61–9·27)	54·77% (27·53–70·62)	12·92% (7·07–16·54)	6·06% (4·41–7·82)	15·10% (10·60–20·40)	52·50% (45·68–60·51)
	Guinea						
	2012	11·51% (10·86–12·26)	22·91% (20·65–25·35)	34·16% (32·75–36·02)	9·95% (9·54–10·37)	10·51% (8·14–13·12)	47·46% (43·75–52·10)
	2021	11·26% (10·57–11·94)	22·94% (18·88–27·02)	29·66% (28·25–31·43)	8·43% (8·00–8·91)	11·78% (9·00–15·21)	45·87% (41·17–51·86)
	2030	10·91% (9·63–12·78)	24·26% (0·46–48·92)	26·02% (17·27–30·50)	8·14% (5·79–10·23)	13·43% (9·73–18·02)	43·68% (37·57–49·17)
	Guinea-Bissau						
	2012	14·15% (13·29–15·03)	40·76% (38·50–42·98)	32·02% (30·83–33·21)	7·51% (7·08–7·95)	10·64% (7·55–14·23)	56·31% (45·94–68·96)
	2021	13·58% (12·75–14·48)	49·58% (46·01–53·08)	27·31% (25·77–28·94)	5·86% (5·47–6·28)	13·02% (9·13–17·66)	56·50% (46·25–70·26)
	2030	13·25% (10·86–16·40)	57·11% (27·79–75·11)	22·36% (14·97–27·87)	5·62% (4·01–6·91)	15·22% (10·93–20·19)	53·83% (46·89–59·68)
	Liberia						
	2012	13·68% (12·86–14·50)	48·53% (46·64–50·25)	33·09% (31·25–35·26)	6·05% (5·74–6·37)	15·61% (12·30–19·42)	65·63% (58·10–75·52)
	2021	12·98% (12·24–13·79)	54·33% (51·10–57·45)	26·68% (24·97–28·70)	4·44% (4·18–4·71)	19·32% (14·36–25·16)	71·41% (61·73–82·38)
	2030	12·83% (10·80–15·69)	60·60% (38·21–74·40)	21·48% (13·71–25·99)	4·30% (3·15–5·45)	23·13% (17·01–30·22)	68·68% (62·53–73·97)
	Mali						
	2012	20·14% (19·14–21·16)	33·27% (31·92–34·57)	29·23% (28·27–30·25)	12·30% (11·84–12·76)	7·70% (5·56–10·30)	56·82% (51·03–65·41)
	2021	16·97% (16·03–17·98)	35·50% (32·22–38·40)	24·62% (23·35–26·00)	9·45% (8·97–9·94)	8·44% (6·06–11·56)	57·44% (49·91–68·60)
	2030	17·06% (14·81–20·46)	38·90% (4·70–61·96)	20·65% (13·89–25·04)	9·05% (7·18–10·41)	10·54% (7·29–14·64)	53·17% (46·58–59·74)
	Mauritania						
	2012	26·82% (25·38–28·15)	29·06% (27·38–30·97)	23·89% (23·19–24·62)	13·28% (12·93–13·64)	8·72% (6·70–11·11)	52·32% (42·49–64·95)
	2021	24·91% (23·59–26·26)	42·02% (38·11–45·69)	19·16% (17·81–20·49)	9·85% (9·37–10·38)	11·68% (8·64–15·66)	50·87% (40·86–63·57)
	2030	23·69% (20·66–28·04)	48·85% (14·27–67·16)	13·57% (7·89–17·22)	9·36% (7·04–10·86)	13·24% (10·11–17·60)	47·02% (39·76–54·72)
	Niger						
	2012	16·06% (15·08–17·03)	19·54% (18·34–20·79)	45·10% (42·62–47·82)	17·02% (16·45–17·60)	6·98% (5·13–8·97)	50·37% (43·20–60·17)
	2021	15·53% (14·52–16·55)	20·82% (16·99–24·46)	42·33% (39·90–44·78)	14·18% (13·55–14·79)	7·52% (5·39–10·19)	50·44% (42·22–61·74)
	2030	15·78% (12·45–20·41)	23·54% (0·03–54·93)	38·91% (27·61–44·00)	13·74% (12·00–15·58)	9·13% (6·12–12·67)	46·21% (38·93–53·61)
	Nigeria						
	2012	11·47% (11·33–11·60)	15·27% (14·61–15·94)	39·41% (38·45–40·42)	12·89% (12·71–13·07)	17·53% (14·11–21·46)	56·99% (53·77–60·55)
	2021	11·22% (11·07–11·38)	28·49% (26·43–30·47)	34·33% (32·92–35·72)	9·90% (9·56–10·26)	19·64% (14·87–24·81)	58·64% (56·16–61·22)
	2030	10·64% (9·27–12·18)	28·89% (1·69–60·93)	31·18% (21·46–34·95)	9·37% (7·29–11·25)	24·74% (19·98–31·77)	58·29% (52·68–63·65)
	São Tomé and Príncipe						
	2012	10·57% (9·91–11·26)	62·46% (59·97–65·03)	26·61% (25·31–27·82)	7·70% (7·29–8·14)	12·97% (9·62–16·68)	43·54% (38·64–50·25)
	2021	10·09% (9·44–10·71)	67·79% (64·72–70·73)	19·80% (18·46–21·19)	5·11% (4·79–5·51)	16·02% (11·67–20·98)	42·93% (36·33–53·72)
	2030	9·37% (7·37–12·25)	69·94% (55·75–79·72)	14·96%* (8·95–19·35)	4·68% (3·36–6·13)	18·35% (13·76–24·04)	38·74% (32·88–45·27)
	Senegal						
	2012	13·28% (12·48–14·13)	38·19% (37·02–39·43)	21·08% (20·14–22·00)	8·67% (8·29–9·07)	7·91% (5·84–10·51)	62·28% (56·61–69·77)
	2021	11·96% (11·28–12·73)	42·03% (38·88–45·06)	16·03% (14·87–17·22)	6·64% (6·28–7·03)	10·03% (7·31–13·41)	67·86% (58·68–78·84)
	2030	11·66% (9·25–14·51)	47·93% (11·04–67·45)	12·07% (7·13–16·03)	6·30% (4·30–8·38)	11·51% (8·28–15·71)	63·59% (58·57–68·14)
	Sierra Leone						
	2012	13·60% (12·66–14·45)	29·91% (28·61–31·28)	36·97% (35·69–38·50)	10·22% (9·80–10·60)	21·01% (18·49–23·74)	43·87% (40·94–47·69)
	2021	11·96% (11·12–12·75)	45·33% (42·60–47·79)	28·33% (26·85–29·73)	6·82% (6·48–7·18)	24·61% (20·67–28·69)	43·49% (38·61–50·51)
	2030	11·38% (10·06–13·85)	53·51% (29·06–70·50)	25·45% (16·60–29·17)	6·53% (4·85–7·86)	30·59% (23·81–37·76)	40·64% (35·49–45·82)
	Togo						
	2012	11·16% (10·38–11·90)	50·66% (48·41–52·77)	26·17% (25·27–27·08)	8·08% (7·68–8·44)	9·18% (7·06–11·81)	62·78% (54·27–72·43)
	2021	10·56% (9·85–11·27)	54·16% (50·09–57·93)	21·77% (20·27–23·16)	5·77% (5·44–6·11)	11·66% (8·44–15·53)	67·20% (58·15–77·13)
	2030	9·77% (8·25–11·75)	59·96% (31·95–76·96)	16·00% (8·99–21·39)	5·07% (3·34–6·81)	14·44% (10·26–20·39)	64·20% (59·34–68·80)

Data are prevalence with 95% uncertainty intervals in parentheses.

* Indicator metric projected to meet the target in 2030.

Global prevalence of exclusive breastfeeding of infants younger than 6 months was 40·74% (95% UI 40·15 to 41·24) in 2012 and 45·05% (44·42 to 45·67) in 2021 (figure 1, table 2). Five countries in 2021 met the GNT of at least 70% prevalence of exclusive breastfeeding, one more than in 2012. These five countries are Rwanda (86·50% [85·15 to 87·83]), Burundi (80·38% [78·61 to 81·97]), Sri Lanka (80·48% [77·87 to 82·83]), Solomon Islands (73·09% [70·32 to 75·57]), and Peru (71·48% [68·92 to 73·94]). The ARC in the prevalence of exclusive breastfeeding from 2012 to 2021 was highest in Nigeria (6·9% [5·9 to 7·9]) and lowest in Chad (–2·3% [–5·3 to 0·5]; appendix 2 pp 5–6, 102–130). In 2021, the prevalence of exclusive breastfeeding was less than 30% in three regions (the Caribbean, central Europe, and eastern Europe), and the ARC of the prevalence of exclusive breastfeeding was less than 2% in the Caribbean and Oceana. The decrease in the ARC of the attributable burden in DALYs of exclusive breastfeeding was greater than the increase in the ARC in prevalence for most countries, including many in sub-Saharan Africa; however, eight countries had a positive ARC in the attributable burden of exclusive breastfeeding despite a positive ARC in prevalence, mostly in central Europe and Oceana (figure 2; appendix 2 pp 37–57, 102–130).

Globally, 155·7 million (95% UI 154·4 to 157·0) children younger than 5 years had stunting in 2021, 64·3 million (63·0 to 65·6) more than the targeted 50% numerical reduction (figure 1; appendix 2 pp 131–141). In 2021, four countries met the stunting GNT: American Samoa (–55% [–60·7 to –48·2]), South Korea (–50·6% [–56·2 to –44·3]), Puerto Rico (–51·9% [–58·2 to –45·1]), and Syria (–63·1% [–65·2 to –61·0]). From 2012 to 2021, the ARC in the prevalence of stunting was lowest in Tonga (–6·6% [–8·2 to –5·0]) and highest in Serbia (0·9% [–0·2 to 1·9]; appendix 2 pp 5, 102–130). In all but five countries (American Samoa, Cook Islands, Monaco, Niue, and Tokelau), the ARC in the attributable burden of stunting was less than the ARC in prevalence (figure 2; appendix 2 pp 37–57, 102–130).

Between 2012 and 2021, the global prevalence of wasting in children younger than 5 years declined from 9·71% (95% UI 9·67 to 9·76) to 7·51% (7·46 to 7·56; figure 1, table 2). Although 83 countries met the wasting target in 2012, 13 additional countries met the target in 2021, with a prevalence of less than 3%, including four in central Europe, eastern Europe, and central Asia. Among the 13 additional countries that met the wasting target in 2021, the ARC in the prevalence from 2012 to 2021 was lowest in Palestine (–5·2% [–5·8 to –4·6]) and highest in Taiwan (–1·0% [–1·8 to –0·1]). The ARC of the prevalence of wasting did not exceed 0·5% in any country (appendix 2 pp 5–6, 102–130). In all but 16 countries, the ARC in the attributable burden of wasting was less than the ARC in the prevalence (figure 2; appendix 2 pp 37–57, 102–130).

The global prevalence of overweight in children aged 2–4 years increased from 15·50% (95% UI 14·87 to 16·18) in 2012 to 19·11% (18·30 to 19·95) in 2021 (figure 1, table 2). Three countries in 2021 met the original GNT of no increase in the prevalence of child overweight: Georgia, Mongolia, and Albania. From 2012 to 2021, the ARC in the prevalence of overweight was lowest in Georgia (–0·5% [–2·6 to 1·7]) and highest in Iran (4·1% [2·0 to 6·3]; appendix 2 pp 5–6, 102–130). Three countries had substantially increased ARC in prevalence (≥80% of model posteriors were positive) for overweight, wasting, and stunting, which were Andorra, Greece, and San Marino. The ARC for the attributable burden of child overweight was less than the ARC in the prevalence in 187 countries; however, the ARC of the attributable burden of child overweight was more than double the ARC of the prevalence in six countries (figure 2; appendix 2 pp 37–57, 102–130).

The global prevalence of anaemia in females of reproductive age rose slightly between 2012 and 2021 from 32·30% (95% UI 31·82 to 32·84) to 33·69% (33·03 to 34·39; figure 1, table 2). In 2021, no countries met the anaemia GNT of halving their 2012 prevalence. From 2012 to 2021, the ARC of the prevalence of anaemia in women of reproductive age was lowest in Malaysia (–2·1% [–4·1 to 0·0]) and highest in Peru (2·4% [0·6 to 4·5]); 26 countries had substantial decreases in anaemia prevalence, as indicated by negative ARCs and that at least 80% of model posteriors were negative, of which most were concentrated in central Latin America (Colombia, El Salvador, Guatemala, Mexico, and Panama; appendix 2 pp 5–6, 102–130). The ARC of the burden of anaemia (in YLDs per 100 000) was less than the ARC of the prevalence for 150 countries, 23 of which had ARCs in prevalence that were substantially less than zero (>80% of posteriors were negative). The ARC for the burden of anaemia was negative in 36 countries with positive ARCs for prevalence (figure 2; appendix 2 pp 37–57, 102–130).

### Epidemiological transition and GNT indicators

**Figure 3:**
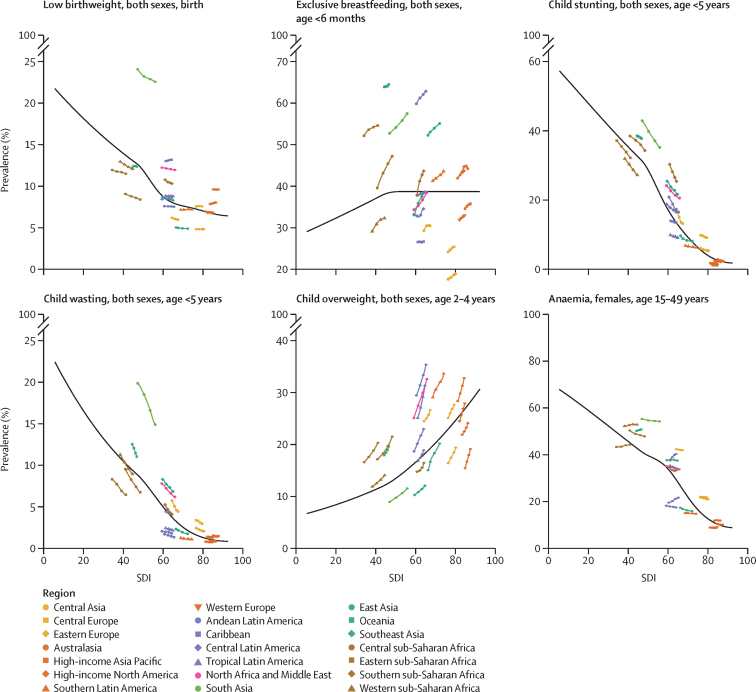
Co-evolution of global nutrition target indicator prevalence with SDI, by region, 2012 to 2021 The expected prevalence values of each indicator, based on the SDI, are represented by the solid black lines. Observed values of the indicators are shown for each region and coloured by super-region. Points are shown every 3 years from 2012 to 2021. SDI=Socio-demographic Index.

**Figure 4:**
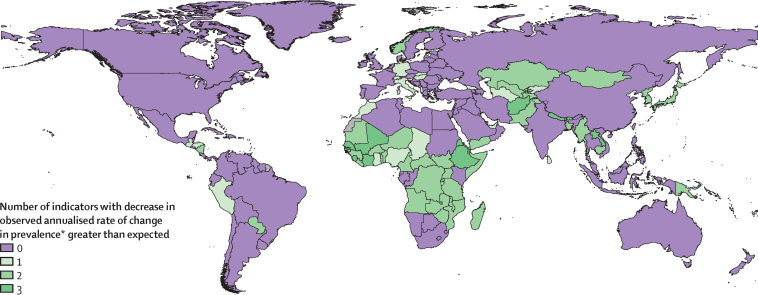
Decrease in global nutrition target indicator observed annualised rate of change in prevalence greater than expected based on Socio-demographic Index, 2012 to 2021 *Restricted to observed annualised rate of change uncertainty level of <20% (>80% of model posteriors were <0).

Socio-demographic Index was generally strongly associated with the prevalence for each indicator except for exclusive breastfeeding. We found a notable regional variation in indicator prevalences compared with values that would be expected on the basis of SDI. Estimated indicator prevalences from 2012 to 2021 by GBD region compared with what would be expected on the basis of SDI is shown in figure 3 (within region comparisons are in appendix 2 [pp 13–33]). In both sexes, SDI negatively correlated with the prevalence of low birthweight, child stunting, and child wasting, whereas SDI positively correlated with prevalence of child overweight (|*r*_s_|=0·46–0·86; appendix 2 p 100). Prevalence of anaemia in females of reproductive age was negatively correlated with SDI and prevalence of exclusive breastfeeding did not correlate with SDI. Individual countries’ prevalence of each indicator compared with expected values on the basis of SDI mostly clustered around the region's performance (appendix 2 pp 13–33), with notable exceptions in regions dominated by a single high-population country (eg, India in south Asia and the USA in high-income North America). The geographical patterns in the ratio between observed prevalence and expected values changed little between 2012 and 2021 (appendix 2 pp 34–35). However, we observed notable patterns where substantial decreases in observed prevalence ARC were greater than expected for multiple indicators (figure 4).

Regions such as central sub-Saharan Africa, east Asia, and eastern Europe exhibited much lower prevalences of low birthweight than would be expected on the basis of SDI (figure 3). By contrast, the prevalence of low birthweight in the regions of south Asia, the Caribbean, and high-income Asia Pacific greatly exceeded expected values. From 2012 to 2021, the ARC in prevalence of low birthweight decreased more than expected in 19 countries (appendix 2 p 36). Because the prevalence of exclusive breastfeeding was not associated with SDI (appendix 2 p 100), we have chosen to omit exclusive breastfeeding from subsequent epidemiological transition results because their interpretation differs.

The region of tropical Latin America tended to exhibit lower prevalence of child stunting than expected on the basis of SDI (figure 3). By contrast, the prevalence of child stunting in south Asia and southern sub-Saharan Africa were considerably higher than expected. From 2012 to 2021, the ARC in prevalence of stunting in Tonga, Bhutan, Côte d’Ivoire, The Gambia, Senegal, and Sierra Leone decreased by over 1·5% more than expected (appendix 2 pp 36, 102–130). The ARC in the prevalence of stunting increased by over 2% more than expected in 63 countries.

Much lower prevalences of child wasting than expected on the basis of SDI were observed in some regions, such as eastern sub-Saharan Africa and Andean Latin America (figure 3). By contrast, prevalences of wasting in south Asia and southeast Asia greatly exceed the expected values. From 2012 to 2021, the ARC in the prevalence of child wasting decreased by at least 1% more than expected in 45 countries, mostly concentrated in western sub-Saharan Africa and central sub-Saharan Africa, and in two countries in South Asia (Nepal and Bhutan; appendix 2 pp 36, 102–130). In aggregate, the differences between observed and expected ARCs for child wasting and stunting in the sub-Saharan Africa super-region were –1·3% and –0·5%, respectively. The ARC in the prevalence of wasting increased by over 2% more than expected in 39 countries, which were mostly in the super-regions of Latin America and the Caribbean and southeast Asia, east Asia, and Oceania.

Lower prevalence of child overweight than expected on the basis of SDI was observed in the high-income Asia Pacific and southeast Asia regions (figure 3). By contrast, the prevalences of child overweight in Andean Latin America and north Africa and the Middle East greatly exceeded expected values. From 2012 to 2021, the ARC in the prevalence of child overweight decreased by over 1% more than expected in one country, Georgia (appendix 2 pp 36, 102–130), and it increased by over 2% more than expected in 25 countries.

Lower prevalence of anaemia in females of reproductive age than expected on the basis of SDI were observed in some regions, such as central Latin America and east Asia (figure 3). By contrast, prevalence of anaemia greatly exceeded expected values in south Asia, central Asia, and central Europe. From 2012 to 2021, no country had a decrease in the ARC in the prevalence of anaemia of over 1% more than expected. An increase in the ARC in the prevalence of anaemia of over 2% more than expected was observed in 41 countries, most of which were in central Europe, southeast Asia, and north Africa and the Middle East (appendix 2 pp 36, 102–130).

### Projected GNT indicator prevalence in 2030 and beyond

**Figure 5:**
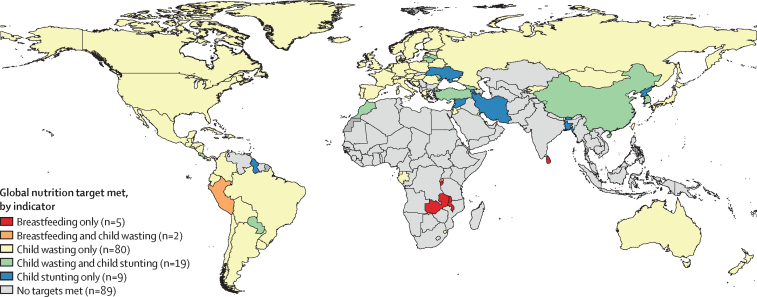
Predicted global nutrition target attainment, 2030

In 2030, we project that 94 countries will meet one of the six targets, 21 countries will meet two targets, and 89 countries will meet no targets (figure 5). Most countries are projected to either meet or be close to meeting the wasting target in 2030, with the exception of most countries in the super-region of sub-Saharan Africa, and no countries in the regions of south Asia and southeast Asia (appendix 2 pp 2–4). By contrast, almost all countries are projected to have a prevalence that is more than 50% higher than the anaemia target in 2030. Such patterns are apparent when examining the maps of required ARC from 2022 to 2030 to meet each target (appendix 2 pp 5–6).

We project that no country in 2030 will meet the low birthweight target of 30% decrease in prevalence compared with the year 2012. Nepal and Bangladesh are projected to be closest to meeting the target, with decreases of 22·5% (95% UI 12·2–33·1) and 21·5% (10·0–35·0), respectively (table 2, appendix 2 pp 2–4).

Seven countries (Sri Lanka, Solomon Islands, Peru, Burundi, Malawi, Rwanda, and Zambia) are projected to meet the target of 70% exclusive breastfeeding prevalence in 2030, and two of these countries (Peru and Rwanda) are also forecasted to meet the wasting GNT.

We project that in 2030, 28 countries, mostly LMICs, will attain the target of reducing the number of children with stunting compared with the year 2012 by 50% (figure 5; appendix 2 pp 134–144). The number of children with stunting relates to the size of the total child population, which is projected to decrease (by 12–54%) from 2012 in all 28 of these countries (appendix p 101). 19 of these countries are also projected to meet the wasting target. Tonga is the only country projected to decrease the prevalence of stunting by more than 50% by 2030, and the prevalence in 17 other countries is projected to be within 20% of the target (appendix 2 pp 2–4).

In 2030, we project 101 countries will meet the wasting target of less than 3% prevalence, 18 of which are LMICs where the prevalence in 2012 was more than 3% (table 2). 22 countries are projected to have a prevalence of 3–4% child wasting, and so will be close to the target (table 2; appendix 2 p 10).

In 2030, we project that no country will attain the child overweight target of no increase in prevalence, or the target of reducing the prevalence of anaemia in females of reproductive age by 50% (table 2; appendix 2 pp 2–4). The projected 2030 prevalence of anaemia in females of reproductive age is at least 50% higher than the target in all countries except for Egypt.

In the decade following 2030, many countries in south Asia and Latin America and the Caribbean are projected to reach at least one additional target (appendix 2 p 58). Looking forward to 2050, we project that 77 countries will meet two or more of the six targets, 94 countries will meet one target, and 33 countries will meet no targets, mostly in central and western sub-Saharan Africa (appendix 2 p 59). We project that five countries (Bangladesh, Cambodia, Nepal, Timor-Leste, and Zimbabwe) will meet the low birthweight target after 2030, but most will not meet it by 2050 (country and target level forecasts to 2050 not shown). Between 2030 and 2050, we project seven more countries will reach the exclusive breastfeeding target, but 190 countries will not reach 70% exclusive breastfeeding prevalence by 2050. We project that 96 more countries will meet the child stunting reduction target between 2030 and 2050, but 80 countries mostly in the high income and sub-Saharan African super-regions will not reach the target by 2050. Nine countries are projected to meet the child wasting target between 2030 and 2050, mostly in sub-Saharan Africa. For child overweight, Kyrgyzstan is the only country projected to have its prevalence in 2050 below its 2012 value. No country is projected to meet the anaemia reduction target by 2050.

## Discussion

Although substantial progress has been made since 2012, few countries are on track to attain multiple global nutrition targets by 2030. Although seven countries had already met two targets in 2021 (Georgia, Mongolia, South Korea, Peru, Rwanda, American Samoa, and Puerto Rico) and 94 had met one target, only 16 additional countries are projected to attain two targets by 2030, and none are projected to meet three targets. 89 countries are projected to miss all targets in 2030, and these are mostly concentrated in western sub-Saharan Africa, southern sub-Saharan Africa, and southeast Asia. The global prevalence of child overweight is projected to increase significantly from 2012 to 2030 and no country is projected to meet the anaemia target even by 2050. While most countries are projected to meet either the child stunting or wasting target by 2050, we project few will meet the low birthweight or child overweight targets by 2050.

LMICs have a disproportionately high disease burden of the GNT indicators, particularly low birthweight and child wasting, which had the greatest global attributable burden (130**·**7 million DALYs in neonates were due to low birthweight and 35**·**0 million DALYs in children younger than 5 years were due to child wasting in 2021).^51^ In 2021, south Asia and sub-Saharan Africa had the largest share of the attributable DALYs from low birthweight and wasting, with 60**·**4 million and 81**·**0 million DALYs, respectively.^51^ Unfortunately, in 2030 all countries in south Asia and sub-Saharan Africa are projected to miss the low birthweight targets, and only five countries in sub-Saharan Africa (Cabo Verde, Equatorial Guinea, Eswatini, Gabon, and Rwanda) are projected to meet the wasting target.

Declines in the attributable burden of low birthweight, child stunting, and child wasting in LMICs have outpaced reductions in prevalence since 2012. This phenomenon is due in part to concurrent declines in the prevalence of diarrhoea, respiratory disease, and other infectious illnesses from a combination of increased availability and uptake of preventive interventions (ie, vaccines), and improvements in indoor air quality and water, sanitation, and hygiene.^55^, ^56^ Additionally, reductions in the prevalence of severe forms of malnutrition resulted in a declining attributable burden from these nutrition indicators. A recent GBD publication noted that, from 1990 to 2019, reductions in the prevalence of moderate stunting and moderate wasting were outpaced by changes in the prevalence of severe (weight-for-height Z score or height-for-age Z score of less than –3) and extreme (less than –4) forms of child stunting and wasting.^49^ Similarly, across age groups and sexes, anaemia YLDs are reported to have decreased more than total anaemia prevalence since 1990 because of a shift towards reduced severity of anaemia among remaining prevalent cases.^57^ If the past trends continue and resources for preventive and treatment programmes are maintained at a level that keeps pace with projected population growth in many of the high-burden areas, then we anticipate the attributable burdens of these indicators will decrease at faster rates than their prevalence in many LMICs in the future.

We found that SDI is a strong correlate of progress for the GNT indicators, apart from for exclusive breastfeeding. Many of the components featured in SDI are probably surrogates for the causal factors driving progress (eg, infrastructure, health systems, and good governance). Therefore, evaluating levels and trends in relation to SDI can be quite powerful, since it can help identify settings where other system factors might be leading to better or worse performance than would be suggested solely by the level of socioeconomic development. Such associations can help identify locations that might have the most to gain from simple interventions or policies (negative exemplars) and those that can serve as a potential example for others to follow (positive exemplars).

The prevalence of both stunting and wasting decreased more than expected across much of sub-Saharan Africa between 2012 and 2021, suggesting that other factors have advanced progress in ways beyond socioeconomic development. The Exemplars in Global Health programme identifies and analyses countries that have made great progress in key health outcomes and highlights the policy and intervention drivers of success. The programme highlighted six countries (Ethiopia, Senegal, Uganda, Nepal, Kyrgyzstan, and Peru) where stunting prevalence has declined faster than in other countries with similar sociodemographic characteristics since the 1990s and identified several non-socioeconomic development drivers of progress.^58^ For example, in Senegal, drivers include enhanced maternal and newborn health services, such as increased antenatal care and skilled birth attendants; strengthened community health systems for better disease management and dietary intake; and government-coordinated, multisectoral nutrition efforts.^59^ And in Ethiopia, drivers include reduced food insecurity from agricultural advancements, a government-led programme that expanded the health-care workforce and improved access to care, and a community-led sanitation programme that substantially reduced rates of open defecation.^60^

Recent evidence suggests that nutritional interventions intended to address undernutrition might contribute to child overweight.^61^ By contrast with undernutrition, between 2012 and 2021 the prevalence of overweight increased more than expected in nearly all countries in sub-Saharan Africa. Policies targeting child overweight, such as parent nutrition education, physical activity promotion, and sugar-sweetened beverage limits and taxes might not be keeping up with socioeconomic development. Notably, the current evidence supporting the effectiveness of these policies is relatively weak,^62^, ^63^, ^64^ and because population growth in sub-Saharan Africa is expected to accelerate in the coming years,^65^ this finding is especially concerning.

Undernutrition and overnutrition are interconnected across the spectrum of growth and development from infancy through adolescence to adulthood. The concept of the double burden of malnutrition—the concurrence of both undernutrition and overweight and obesity—underscores the necessity of integrated strategies that foster a comprehensive approach to public health.^66^ This complex association suggests that interventions designed to address one aspect of malnutrition without considering the other might inadvertently exacerbate the problem, particularly in LMICs with rapidly changing food systems.^67^ Since 2000, in LMICs, the double burden of malnutrition most notably affected areas in central Nigeria, Cameroon, Botswana, southeastern China, Thailand, and Indonesia.^6^ Three high-income countries in the present analysis (Andorra, Greece, and San Marino) exhibited increasing prevalence of child stunting, wasting, and overweight from 2012 to 2021. Therefore, it is vital to adopt so-called double-duty actions, tailored to simultaneously mitigate the risks of undernutrition and overnutrition and ensure that solutions are both effective and sustainable.^68^ For instance, promoting the consumption of nutrient-dense, minimally processed foods can help prevent stunting due to nutrient deficiencies and reduce the risk of obesity from high-calorie, low-nutrient diets. Incorporating this perspective into public health discourse and clinical practice is essential for the health and wellbeing of current and future generations.

All countries in south Asia had considerable decreases in the prevalence of stunting and wasting between 2012 and 2021. Nepal in particular had a greater reduction in the ARC of stunting prevalence than expected on the basis of SDI. The Exemplars in Global Health reported that large education and literacy gains have driven much of Nepal's stunting progress, along with strengthening of health-care systems (eg, expansion of the female community health volunteer programme and primary health services to rural populations, as well as multisectoral collaborations that improved obstetric care and increased antenatal visits), substantive improvements to water, sanitation, and hygiene (eg, community-led total sanitation programmes that decreased open defecation).^69^ Many policy changes in Nepal increasingly empowered women in ways that had numerous benefits for poverty reduction and child health. Sustained decreases in the prevalence of stunting in south Asia and elsewhere will require policies that promote these and other identified drivers.

Many LMICs in central Europe, eastern Europe, central Asia, and southeast Asia underperformed considerably for anaemia and wasting, suggesting that existing treatment or prevention strategies, like iron supplementation and ready-to-use therapeutic food, respectively, might be insufficient or not adequately implemented. The weak association between locations’ SDI and the prevalence of exclusive breastfeeding suggests that the cultural factors and policies underlying breastfeeding practices are probably not correlated with socioeconomic development in the full 1990–2021 modelled period. Hence, improvements in breastfeeding rates are most likely largely due to successful implementation of policy efforts, regardless of the socioeconomic status of the country. The weak association might also be driven in part by the subnational locations included in the analysis (ie, for India, Brazil, and China), with median prevalence of exclusive breastfeeding being approximately 10% higher in those subnational locations than in the national locations included (subnational SDI estimates not shown; data can be accessed via Global Health Data Exchange).

In 2021, the two largest indicators by disease burden in countries in the high-income super-region were low birthweight (1·4 million DALYs in neonates) and anaemia in females of reproductive age (387 797 YLDs).^51^ Although all 36 countries in the high-income super-region are projected to meet the wasting target in 2030, they are not projected to meet any other target. Most countries in this super-region did not have a decrease in the prevalence of any indicators that was more rapid than expected on the basis of SDI from 2012 to 2021. Nine countries in the high-income super-region had an increase in the ARC of the prevalence of anaemia and low birthweight that was greater than expected on the basis of SDI, but this overperformance was very slight (<0·5%). Globally, the prevalence of low birthweight projected for 2030 (11·88% [95% UI 11·20 to 12·60]) exceeds the 30% reduction target from 2012 (9·06%) by 2·82%. South Asia will probably continue to have the greatest burden of low birthweight into 2030, although the attributable burden will probably decline more quickly than the prevalence of low birthweight, should past trends continue. Because newborns with low birthweight are at increased risk of morbidity and mortality and more likely to have stunting and wasting in childhood,^12^, ^13^, ^14^, ^15^, ^16^, ^17^ stagnant prevalences of low birthweight could impede future progress in reducing stunting and wasting. Increased adoption and implementation of policies that reduce low birthweight include those to improve maternal nutrition (eg, supplementation with folic acid, iron, and vitamin A), promote antenatal care visits, increase maternal health education (eg, time between pregnancies, age at first pregnancy, and substance use), and ensure access to contraceptives and family planning. Although global prevalence of exclusive breastfeeding is projected to increase to 46·68% by 2030, this estimate does not meet the desired target, leaving many young children more susceptible to infectious diseases and nutritional deficiencies.^24^, ^25^ Important approaches to increase exclusive breastfeeding are policies to promote the early initiation of breastfeeding (which improves neonatal outcomes) and discourage prelacteal feeding and breast milk substitutes, the establishment of milk banks to ensure breastmilk for premature or sick infants whose mothers might not be able to produce enough milk, and regulations that provide mothers with paid parental leave, breastfeeding breaks, and facilities for milk expression at the workplace.^70^, ^71^, ^72^, ^73^, ^74^ Public education campaigns can help to normalise breastfeeding in public, reducing stigma and barriers for breastfeeding mothers.^71^ We project that there will be approximately 38·9 million more children with stunting than the target in the year 2030, and that the decline in the global prevalence of wasting after 2021 will be modest. The projected 6**·**8% growth in the global prevalence of overweight children from 2012 to 2030 puts an increasing proportion of children at heightened immediate risk for asthma and for continued high BMI later in childhood.^36^, ^75^ Childhood obesity continues to increase, suggesting that in most countries there is an overabundance of industrially processed foods that contribute to increased body mass, along with factors such as decreases in physical activity and shifting cultural norms. These trends will probably result in future increases in the prevalence of high BMI in adults, thus contributing to its attributable burden of cardiometabolic disorders, high blood pressure, and other chronic health issues.^36^, ^37^, ^38^, ^39^ GBD estimates of the burden of anaemia in females of reproductive age only includes the estimation of YLDs related to prevalence of mild, moderate, and severe anaemia and does not include separate assessment of low haemoglobin concentrations in pregnancy as a risk factor for other conditions (eg, post-partum haemorrhage, peri-partum depression, and low birthweight and short-gestation births, among others). How the burden of these anaemia-related conditions has changed since 2012 is not clear. Anaemia is caused by many different and often comorbid conditions. These conditions include deficiency of micronutrients such as folic acid, vitamin B12, elemental iron, and vitamin A; infections such as malaria, HIV/AIDS, and parasitic infections; non-communicable diseases such as gastrointestinal disorders, autoimmune conditions, and kidney and liver disease; menstrual dysregulation; genetic blood and haemoglobin disorders; and any condition that leads to bone marrow suppression or clinically significant blood loss. Although most anaemia reduction policies have prioritised iron supplementation,^76^ individuals with anaemia might be less responsive to iron interventions if the specific cause of their condition remains untreated. Therefore, extensive and improved diagnostics for better aetiological and population specificity, targeted dietary iron supplementation, expanded programmes to control parasitic and infectious diseases, and effective education of females of reproductive age are needed to sustainably reduce anaemia in the long term.^77^, ^78^, ^79^ WHO recently published a framework to improve the prevention, diagnosis, and management of anaemia that acknowledges the condition's multifactorial origin.^80^ Concerningly, we project that 11 of 19 countries in western sub-Saharan Africa will not meet any targets, and only three countries in western Europe will meet new targets by 2050.

The GNTs are valuable but flawed benchmarks, and the variation in progress to date underscores the need to carefully choose appropriate indicators and metrics for future targets. No country had met the low birthweight target by 2021 or is projected to do so by 2030. The current low birthweight target does not differentiate between preterm and small-for-gestational-age births, which can lead to misdirected interventions that do not address the specific needs of different populations. Reliable and consistent data on birthweight are not available in all countries, especially in LMICs where home births are common. Importantly, the low birthweight target aims for a relative decrease in prevalence without considering the starting level; for instance, having a 30% reduction can be more challenging in countries where the prevalence is already low. Conversely, the exclusive breastfeeding target of 70% is nearly impossible to achieve for countries in which exclusive breastfeeding rates in 2012 were less than 30%, while others might need to sustain current rates to hit the target. Most of the GNTs are defined as rates, which are useful to compare case levels and trends between populations of different sizes or when population sizes are fluctuating. By contrast, investigating counts enables us to see the full scope of the health problem, who is affected, and where. If case counts are seen to be increasingly concentrated in a region or country, this information can have important global policy implications (eg, for health spending, implementation, and research). The attainment of the stunting count target (ie, total number of affected children) conflates demographic shifts with improvements in child health. Child populations are decreasing in many countries due to a combination of emigration (particularly of females of reproductive age) and decreasing fertility rates. These predicted fertility trends have been reported elsewhere.^65^, ^81^ Such demographic shifts might affect countries’ ability to meet the count-based stunting target. For example, Syria, a country that has been in civil war since 2011, is forecast to meet the stunting target in 2030, due to the combination of a projected 4% prevalence drop and halving of the total child population. Of the 28 countries projected to meet the stunting target in 2030, only Tonga is expected to have a decrease in the prevalence of stunting of 50%. Importantly, we acknowledge that the interpretation of counts or rates in isolation can be misleading. In 2017, the child overweight target was changed from no change from 2012 to 2025 to an absolute target of 3% prevalence in 2030.^3^ Notably, WHO defines overweight on the basis of the weight-to-height Z score of more than 2 SD in children younger than 5 years. There is no consensus about the optimal definition of overweight in children (IOTF BMI *vs* WHO weight-for-height Z score), so if an absolute target is used, ideally it should be articulated in both standards. In future nutrition targets, we suggest that prevalence of child overweight and obesity is better examined in school-aged and adolescent children (age 12–18 years), in whom it is more closely associated with high adult BMI.^39^ The initial target for a 50% reduction in the prevalence of anaemia in females of reproductive age appears overly ambitious because no country is projected to meet the target even by 2050. A more realistic goal, such as a 35% reduction, could encourage a broader range of strategies beyond just iron supplementation to target the specific causes of anaemia. Addressing the various causes of anaemia, including nutritional deficiencies, parasitic infections, and genetic conditions, requires diverse and context-specific policies.^57^, ^80^ Potential selection criteria for future nutrition indicators include sensitivity and specificity for morbidity and mortality risk, feasibility and scalability (specifically, ease of data collection, analysis, and interpretation across different contexts and at scale), relevance, and actionability. Nutrition indicators that ought to be considered for the post-2030 agenda might encompass measures that reflect dietary quality, such as fruit and vegetable intake, processed food consumption, or deficiencies of micronutrients such as vitamin A or zinc.

The findings of this analysis, as well as the associated areas of uncertainty, point to several near-term implications for global nutritional status. First and foremost, global disruptions such as the COVID-19 pandemic were major threats to progress towards attaining nutritional improvements. Although some of this disruption is captured within the SDI forecasts used in the 2030 prevalence projections, which feature downward adjustments for the effects of the pandemic, these SDI projections do not account for the regional and global effects of major conflicts such as those in Ukraine, Ethiopia, Israel, and Palestine, or the potential effects of ongoing and worsening climate factors. All these factors probably variably impede GNT progress in ways not captured fully by development indicator forecasts.

To our knowledge, this analysis is the most comprehensive assessment of GNT progress to date and is fully integrated with GBD 2021; however, several other studies have also reported on progress toward GNTs. In a joint effort, UNICEF, WHO, and World Bank annually produce the global and country-level joint child malnutrition estimates (JMEs), and estimated that globally in 2022, 22·3%, 6·8%, and 5·6% of children younger than 5 years had stunting, wasting, and overweight, respectively.^82^, ^83^ The first two global estimates are similar to those in the current Article, but comparing child overweight estimates is difficult due to age differences and the use of WHO rather than IOTF criteria. According to the JME, between 2012 and 2022, the prevalence of overweight in children younger than 5 years was either maintained or decreased in 80 of 160 countries, and prevalence increased by at least 40% in 22 countries. In comparison, we estimated that between 2012 and 2021, three (1%) of 204 countries had no discernible increase in prevalence of overweight in children aged 2–4 years. We estimated that 155·7 million children had stunting globally in 2021, compared with 149·7 million estimated by JME. We estimated that the prevalence of stunting increased in six (3%) of 204 countries compared with 24 (15%) of 160 countries as part of JME, and the ARC in the prevalence of child stunting produced by JME differs from our estimate by more than 20% for 136 (80%) of 158 countries included in estimations by both groups. The JME statistical approach is cruder than that used in GBD but with more stringent criteria for dataset inclusion, includes some more recent datasets, and estimates the prevalence of categorical growth status rather than continuous growth distributions with a penalised longitudinal mixed model.^82^ Future iteration and collaboration efforts could reconcile the differences in country-level estimates, some of which are fairly wide (eg, 5% difference in the prevalence of stunting in India). A recent JME report concluded that fewer than a third of countries are on track to meet the GNT stunting target (based on ARC), which is consistent with our 2030 projections, in which only 28 (14%) of 204 countries and territories are anticipated to meet the target by 2030.^83^ These joint UNICEF, WHO, and World Bank estimates are used in the GNT prevalence trends that are featured in the annual Global Nutrition Report and the State of Food Security, Nutrition in the World report, and a WHO discussion paper that proposed process indicators and operational targets for the GNTs.^84^, ^85^, ^86^

A 2019 study of low birthweight in 2015 estimated the prevalence of low birthweight from 2000 to 2015 (with a restricted maximum likelihood approach with country-level random effects) and estimated the global prevalence in 2015 to be 14·6%, which is slightly higher than our 2015 estimate (12·7% [95% UI 12·6–12·7]; data not shown; available on the Global Health Data Exchange).^9^ Another publication using spatial modelling in LMICs predicted that ten LMICs (*vs* 60 included in the present study) would meet the original (GNT 2025) exclusive breastfeeding target by 2025.^4^, ^7^ In most countries, our projected 2030 prevalences of stunting are less optimistic and our projected prevalences of wasting are more optimistic than in another spatial modelling study of LMICs that used 2010 as the reference year.^5^ Although we estimated that only Tonga is on track to reduce stunting prevalence by 50% by 2030, the prevalence of severe stunting (height-for-age Z score of –4 to –3) and extreme stunting (less than –4) are declining at a higher rate than stunting overall in most countries.^49^ A 2020 publication estimated the prevalence of overweight in children in LMICs based on BMI from 2000 to 2017, but raster (5 × 5 km grid) projections for 2025 were not aggregated to the national level.^6^ Similar spatial modelling methods were used to estimate subnational prevalence of anaemia in females of reproductive age in 82 LMICs from 2000 to 2018, with simple prevalence projections to the year 2030 created using recency-weighted ARCs applied to the final estimate year.^87^ In this study, they projected that only three countries will meet the target to reduce the prevalence of anaemia by 2030: China, Iran, and Thailand. One previous study comprehensively modelled the prevalence and burden of anaemia and reported all-age prevalence from 1990 to 2013 in 195 countries, but did not report separate estimates in females of reproductive age.^8^ A 2022 study estimated the national and subnational prevalence of anaemia in females of reproductive age in 15 LMICs from 2000 to 2018 and projected prevalence to 2025, and concluded that none were on track to hit the anaemia target by 2025.^88^ Another study estimated the prevalence of anaemia in females of reproductive age for 133 countries from 2000 to 2019 and reported a global prevalence in 2019 of 30%,^89^ similar to our study estimate of 33·69% in 2021. Our prevalence projections for 2030 in India were less optimistic than those in an India-specific analysis using GBD 2017 results, with projected prevalence differing between 0% and 3·6% for all GNT indicators.^10^ Major changes from previously published forecast studies from earlier GBD iterations and Local Burden of Disease studies include new data sources to improve retrospective estimates and methodological advances for improved retrospective estimates (eg, change to ensemble models that minimise absolute prediction error in highly relevant areas of the child growth curve and an optimisation algorithm that targets the set of ensemble weights that minimises the predictive error across all microdata sources). These forecasts are the first to use a cascading spline model that borrows strength across locations to predict future prevalence from summary exposure values and feature limited adjustments for the effects of the COVID-19 pandemic.

### Limitations

This study has limitations, many of which have been described elsewhere.^49^, ^50^, ^51^, ^57^ First, the raw data used to inform retrospective estimates of indicator prevalence are of variable quality and sparse in some countries or time periods. This is particularly true for birthweight and gestational age data in LMICs. However, GBD has developed a comprehensive methodology to address such problems, including annual searches with in-country collaborators for data followed by thorough cleaning and correction, and modelling approaches to maximise the use of available data. Although we endeavour to identify and include as much data as possible, there are inevitably useful new data sources identified by collaborators and GBD users that can be incorporated into models. Regular updates to GBD estimates help minimise the lag between data identification and incorporation into models. Input data sources beyond 2019 were relatively sparse and some countries, such as Syria, have reduced data availability due to major disruptions from prolonged conflict. The validity of breastfeeding data varies substantially by ascertainment method and the specific metric measured,^90^, ^91^ and because most input data for exclusive breastfeeding comes from maternal self-report, exclusive breastfeeding might be overestimated. Second, because GBD uses IOTF standards to model overweight in children aged 2–4 years, it is not possible to directly compare prevalence estimates of child overweight with the extended 2030 target of 3% prevalence in children younger than 5 years, which uses the WHO weight-for-height Z score-based definition. Instead, we applied the 2025 overweight target of no increase from 2012.^3^ Third, the sole use of iron deficiency SEVs to predict the future prevalence of anaemia in females of reproductive age means that other major causes of anaemia, such as malaria and uterine fibroids,^8^ are not considered. The inclusion of forecasts of these conditions as covariates would probably improve the prediction of the prevalence of anaemia and decrease uncertainty. Finally, we did not account for the sizeable effects that both the COVID-19 pandemic and regional conflicts continue to have on food insecurity in LMICs, as well as disruptions to health services and supply chains (eg, for nutrition commodities). The omission of these factors in our models and data sparsity after 2019 suggest our prevalence estimates after 2019 might be overly optimistic, and are likely to worsen the future prevalence of the GNT indicators compared with our forecasts. Between 2019 and 2020, and before the recent Russia–Ukraine conflict and the recent conflicts in the Middle East, the global prevalence of moderate or severe food insecurity increased from 26·6% to 30·4%, more than in the previous 5 years combined.^85^

### Future directions

As mentioned, research is needed to elucidate the effects of the COVID-19 pandemic on GNT indicator progress across locations. Future work is needed to improve data collection across all the GNT indicators, but especially low birthweight in LMICs. Researchers must work to quantify the intergenerational effects and potential burden of anaemia during pregnancy in neonates and young children. The evaluation of haemoglobin alone might not be sufficient to make adequate recommendations for policy, and therefore the inclusion of additional laboratory values could be used to improve the utility of recommendations.^78^, ^79^ Additionally, to our knowledge, no systematic efforts exist that model the future disease burden of overweight and obesity in adults that is anticipated given the growing prevalence of child overweight and obesity. Such attributable burden projections would be essential tools for health policy makers. Furthermore, repeating and focusing analyses and forecasting to the year 2030 (with new data through 2023) for each indicator separately could be useful because our findings differ considerably by indicator.

### Conclusions

Progress towards attaining the GNTs has been largely insufficient. Although the specific targets outlined for the GNTs might not have been optimally formulated to motivate in-country policy changes, improvement of these indicators has been slow. Additional efforts are needed to reduce the prevalence of both low birthweight and stunting to a meaningful degree not conflated with demographic shifts. Promisingly, most countries and territories have increased the prevalence of exclusive breastfeeding, but considerable progress needs to be made in the regions of eastern Europe, central Europe, and the Caribbean. In most countries in western and central sub-Saharan Africa, the prevalence of wasting decreased more rapidly than expected on the basis of SDI between 2012 and 2021, but greater decreases are needed. The prevalence of overweight in children rose in almost all countries, and particularly in south Asia and east Asia. Persistence of child overweight and obesity into adulthood has the potential to lead to a tremendous increase in future global disease burden. Policy makers, governments, and researchers must redouble current efforts to improve maternal and child nutrition globally to prevent future deaths and disability.

### Global Nutrition Target Collaborators

### Affiliations

### Contributors

### Data sharing

The code for the analysis is available from GitHub and to download GBD data used and estimates generated in these analyses see The Global Health Data Exchange.

For more on the **SDGs** see https://sdgs.un.org/

## Declaration of interests

S Afzal reports support from King Edward Medical University for study material, research articles, valid data sources and authentic real time information for the present manuscript. S Afzal reports payment or honoraria for lectures, presentations, speakers bureaus, manuscript writing or educational events from King Edward Medical University and collaborative partners including University of Johns Hopkins, University of California, University of Massachusetts, King Edward Medical College Alumni Association of North America (KEMCAANA), and King Edward Medical College Alumni Association UK (KEMCA UK) for international scientific conferences, webinars, and meetings; support for attending meetings and/or travel from King Edward Medical University; participation on a Data Safety Monitoring Board or Advisory Board with National Bioethics Committee Pakistan, King Edward Medical University Ethical Review Board, Ethical Review Board Fatima Jinnah Medical University and Sir Ganga Ram Hospital as a member of the Technical Working Group on Infectious Diseases; leadership or fiduciary roles in board, society, committee, or advocacy groups, paid or unpaid, with Pakistan Association of Medical Editors, Faculty of Public Health Royal Colleges UK (FFPH) as a fellow, Society of Prevention, Advocacy and Research, King Edward Medical University (SPARK), and Pakistan Society of Infectious Diseases as a member; other financial or non-financial interests as Dean of Public Health and Preventive Medicine at King Edward Medical University, Chief Editor of *Annals of King Edward Medical University* since 2014, Director of Quality Enhancement Cell at King Edward Medical University, Fellow of Faculty of Public Health United Kingdom, Advisory Board Member and Chair of Scientific Session KEMCA UK, Chairperson of International Scientific Conference KEMCAANA, Member of Research and Publications Higher Education Commission (HEC) Pakistan, Member of Research and Journals Committee Pakistan Medical and Dental Council, Pakistan Member of National Bioethics Committee at Punjab Level, Member of Corona Experts Advisory Group, Member of Technical Working Group on Infectious Diseases, Member of Dengue Experts Advisory Group, and Chair of Punjab Residency Program Research Committee; all outside the submitted work. R Ancuceanu reports consulting fees from AbbVie; payment or honoraria for lectures, presentations, speakers bureaus, manuscript writing or educational events from AbbVie, Laropharm, Reckitt, Merck Romania; supports for attending meetings and/or travel from Merck Romania; all outside the submitted work. T Bärnighausen reports grants or contracts paid to their institution from the US National Institutes of Health (NIH), Alexander von Humboldt Foundation, German National Research Foundation (DFG), European Union, German Ministry of Education and Research, German Ministry of the Environment, Wellcome, KfW; honoraria as Editor-in-Chief of *PLoS Medicine*; participation on a Data Safety Monitoring Board or Advisory Board, unpaid, on two Scientific Advisory Boards for NIH-funded research projects in Africa on Climate Change and Health; stock or stock options in CHEERS, a subject matter expert focusing on approaches to measure climate change and health-related variables in population cohorts; all outside the submitted work. J Baker reports grants or contracts paid to their institution from World Cancer Research Fund, Novo Nordisk Foundation, Independent Research Fund Denmark; consulting fees to them and their institution from Novo Nordisk A/S; payment or honoraria for lectures, presentations, speakers bureaus, manuscript writing, or educational events paid to their institution from Novo Nordisk A/S; leadership or fiduciary roles in board, society, committee, or advocacy groups, unpaid, with European Association for the Study of Obesity as Trustee; all outside the submitted work. O Baltatu reports grants or contracts from Alfaisal University; Anima Institute (AI) Research Professor Fellowship; and a National Council for Scientific and Technological Development Fellowship (CNPq, 304224/2022-7); leadership or fiduciary roles in board, society, committee, or advocacy groups, paid or unpaid with VividiWise Analytics as Managing Partner, São José dos Campos Tech Park – CITS as Health and Biotech Advisory Board Member; all outside the submitted work. S L Bell reports grants or contracts paid to their institution from US Environmental Protection Agency (EPA), NIH, High Tide Foundation, Health Effects Institute, Yale Women Faculty Forum, Environmental Defense Fund, Wellcome Trust Foundation, Yale Climate Change and Health Center, Robert Wood Johnson Foundation, Hutchinson Postdoctoral Fellowship; consulting fees from Clinique, ToxiMap, SciQuest; payment or honoraria for lectures, presentations, speakers bureaus, manuscript writing, or educational events from Colorado School of Public Health, Duke University, University of Texas, Data4Justice, Korea University, Organization of Teratology Information Specialists, University of Pennsylvania, Boston University, IOP Publishing, NIH, Health Canada, EHS, PAC-10, UK Research and Innovation (UKRI), AXA Research Fund Fellowship, Harvard University, University of Montana, and SciQuest; support for attending meetings and/or travel from Colorado School of Public Health, University of Texas, Duke University, Boston University, University of Pennsylvania, Harvard University, American Journal of Public Health, Columbia University, CMAS Conference, and Nature Conference; leadership or fiduciary roles in board, society, committee, or advocacy groups unpaid with Fifth National Climate Assessment, Lancet Countdown, Johns Hopkins EHE Advisory Board, Harvard external advisory committee for training grant, WHO Global Air Pollution and Health Technical Advisory group, National Academies Panels and Committees, and paid with US EPA Clean Air Scientific Advisory Committee (CASAC); all outside the submitted work. S Bhaskar reports grants or contracts from Japan Society for the Promotion of Science (JSPS) and Japanese Ministry of Education, Culture, Sports, Science and Technology (MEXT) for a grant-in-aid for Scientific Research (KAKENHI) (P23712), and from JSPS and the Australian Academy of Science for a JSPS International Fellowship (P23712); leadership or fiduciary roles in board, society, committee or advocacy groups, paid or unpaid with Rotary District 9675, Sydney, Australia (District Chair, Diversity, Equity & Inclusion), Global Health & Migration Hub Community, Global Health Hub Germany, Berlin, Germany (Chair, Founding Member and Manager), *PLoS One*, *BMC Neurology*, *Frontiers in Neurology*, *Frontiers in Stroke*, *Frontiers in Public Health*, *Journal of Aging Research,* and *BMC Medical Research Methodology* (Editorial Board Member), College of Reviewers, Canadian Institutes of Health Research (CIHR), Government of Canada (Member), World Headache Society, Bengaluru, India (Director of Research), Cariplo Foundation, Milan, Italy (Expert Adviser/Reviewer), National Cerebral and Cardiovascular Center, Department of Neurology, Suita, Osaka, Japan (Visiting Director), and Cardiff University Biobank, Cardiff, UK (Member, Scientific Review Committee); all outside the submitted work. Z A Bhutta reports grants or contracts from Gates Ventures and Bill & Melinda Gates Foundation, outside the submitted work. M Carvalho reports support from Laboratório Associado para a Química Verde (LAQV/REQUIMTE), and University of Porto, Porto, Portugal Foundation for Science and Technology (FCT) Portuguese Ministry of Science, Technology, and Higher Education (MCTES) under the scope of the project UIDP/50006/2020 (DOI 10.54499/UIDP/50006/2020), outside the submitted work. A L Catapano reports grants or contracts from Amryt Pharma, Menarini, Ultragenyx; consulting fees from Menarini - Menarini Ricerche, Sanofi; payment or honoraria for lectures, presentations, speakers bureaus, manuscript writing or educational events from Amarin Amgen Amryt Pharma, AstraZeneca, Daiichi Sankyo Esperion Ionis Pharmaceutical Medscaper, Menarini, Merck, Novartis, Novo Nordisk, Peervoice, Pfizer, Recordati, Regeneron, Sandoz, Sanofi, The Corpus, Ultragenyx, and Viatris; all outside the submitted work. S Cortese reports grants or contracts from National Institute for Health and Care Research (NIHR) UK, European Research Agency; payment or honoraria for lectures, presentations, speakers bureaus, manuscript writing, or educational events from Association for Child and Adolescent Mental Health (ACAMH), Medice, British Association of Psychopharmacology (BAP), and Canadian ADHD Resource Alliance (CADDRA); support for attending meetings and/or travel from Medice, BAP, and CADDRA; leadership or fiduciary roles in board, society, committee or advocacy groups, paid or unpaid with European ADHD Guidelines Group (EAGG) as Chair; all outside the submitted work. R C Franklin reports support for attending meetings and/or travel from ACTM – Tropical Medicine and Travel Medicine Conference 2022, 2023, ISTM – Travel Medicine Conference, Basel 2023; leadership or fiduciary roles in board, society, committee, or advocacy groups, paid or unpaid with Kidsafe as President/Director, Farmsafe as Director, Auschem as Director, ISASH Governance Committee as PHAA Injury Prevention SIG Convenor, and ACTM as President Elect; all outside the submitted work. N Ghith reports grants or contracts from Novo Nordisk Foundation (NNF16OC0021856) paid to their institute, Technical University of Denmark, between 2019 and 2022; payment or honoraria for lectures, presentations, speakers bureaus, manuscript writing, or educational events from Danish Data Science Institute at the Technical University of Denmark, travel grant in 2023; support for attending meetings and/or travel from Danish Data Science Institute at the Technical University of Denmark; all outside the submitted work. P Gill reports support for the present manuscript paid to their institution from NIHR Senior Investigator Award. C Herteliu reports grants or contracts from the Romanian Ministry of Research, Innovation and Digitalization through UEFISCDI (project “Analysis of the impact of COVID-19 on the main demographic indicators in Romania and the Republic of Moldova by using econometric modeling”; code PN-IV-P8-8.3-ROMD-2023-0208), the European Commission Horizon 4P-CAN (Personalised Cancer Primary Prevention Research through Citizen Participation and Digitally Enabled Social Innovation), European Union – NextgenerationEU and Romanian Government under National Recovery and Resilience Plan for Romania through the Romanian Ministry of Research, Innovation and Digitalization, within Component 9, Investment I8 (projects “Societal and Economic Resilience within multi-hazards environment in Romania” contract no.760050/ 23.05.2023, cod PNRR-C9-I8-CF 267/ 29.11.2022 and “A better understanding of socio-economic systems using quantitative methods from Physics” contract no.760034/ 23.05.2023, cod PNRR-C9-I8-CF 255/ 29.11.2022); all outside the submitted work. I Ilic reports support for the present manuscript from the Ministry of Education, Science and Technological development, Republic of Serbia (project No 175042, 2011-2023). M Ilic reports support for the present manuscript from the Ministry of Science, Technological Development and Innovation of the Republic of Serbia (no. 451-03-47/2023-01/200111). N E Ismail reports unpaid leadership or fiduciary roles in board, society, committee, or advocacy groups with the Malaysian Academy of Pharmacy as Bursar and Council Member and the Malaysian Pharmacists Society as Committee Member of the Education Chapter; all outside the submitted work. J Jozwiak reports payment or honoraria for lectures, presentations, speakers bureaus, manuscript writing, or educational events from Novartis, Adamed, and Amgen; all outside the submitted work. K Kewal reports non-financial support from the UGC Centre of Advanced Study, CAS II, awarded to the Department of Anthropology, Panjab University, Chandigarh, India, outside the submitted work. B Lacey reports support for the present manuscript from UK Biobank, funded largely by the UK Medical Research Council and Wellcome, paid to their institution, the University of Oxford. M-C Li reports support for the present manuscript from the National Science and Technology Council, Taiwan (NSTC 113-2314-B-003-002). M-C Li reports leadership or fiduciary roles in board, society, committee, or advocacy groups, paid or unpaid, with the *Journal of the American Heart Association* as Technical Editor, outside the submitted work. S Lorkowski reports grants or contracts paid to their institution from dsm-firmenich (formerly DSM Nutritional Products); consulting fees from Danone, Novartis Pharma, and Swedish Orphan Biovitrum (SOBI); payment or honoraria for lectures, presentations, speakers bureaus, manuscript writing, or educational events from AMARIN Germany, Amedes Holding, Amgen, Berlin-Chemie, Boehringer Ingelheim Pharma, Daiichi Sankyo Deutschland, Danone, Hubert Burda Media Holding, Janssen-Cilag, Lilly Deutschland, Novartis Pharma, Novo Nordisk Pharma, Roche Pharma, Sanofi-Aventis, Swedish Orphan Biovitrum (SOBI), and SYNLAB Holding Deutschland; support for attending meetings and/or travel from Amgen; participation on a Data Safety Monitoring Board or Advisory Board with Amgen, Daiichi Sankyo Deutschland, Novartis Pharma, and Sanofi-Aventis; all outside the submitted work. Y A Melaku reports grants or contracts from National Health and Medical Research Council of Australia (NHMRC) Investigator Grant (2009776) outside the submitted work. A-F Mentis reports grants or contracts from ‘MilkSafe: A novel pipeline to enrich formula milk using omics technologies’, a research co-financed by the European Regional Development Fund of the European Union and Greek national funds through the Operational Program Competitiveness, Entrepreneurship and Innovation, under the call RESEARCH - CREATE - INNOVATE (project code: T2EDK-02222), as well as from ELIDEK (Hellenic Foundation for Research and Innovation, MIMS-860); payment for expert testimony from Fondazione Cariplo, Italy as external peer-reviewer; leadership or fiduciary roles in board, society, committee or advocacy groups, paid or unpaid with *Systematic Reviews* and *Annals of Epidemiology* as Editorial Board Member, and with *Translational Psychiatry* as Associate Editor; stock or stock options in a family winery; other financial or non-financial support from BGI Group as Scientific Officer; all outside the submitted work. L Monasta reports support for the present manuscript from the Italian Ministry of Health (Ricerca Corrente 34/2017), payments made to the Institute for Maternal and Child Health IRCCS Burlo Garofolo. A P Okekunle reports support for the present manuscript from National Research Foundation of Korea funded by the Ministry of Science and ICT (2020H1D3A1A04081265). A P Okekunle reports supports for attending meetings and/or travel from National Research Foundation of Korea funded by the Ministry of Science and ICT (2020H1D3A1A04081265) outside the submitted work. A Ortiz reports grants or contracts from Sanofi (paid to their institution: IS-FJD Universidad Autonoma de Madrid (UAM)) and as Director of the Catedra Mundipharma-UAM of diabetic kidney disease and the Catedra Astrazeneca-UAM of chronic kidney disease and electrolytes (paid to their institution: UAM); consulting fees from Advicciene , Astellas, AstraZeneca, Amicus, Amgen, Fresenius Medical Care, GSK, Bayer, Sanofi-Genzyme, Menarini, Kyowa Kirin, Alexion, Idorsia, Chiesi, Otsuka, Novo Nordisk, and Vifor Fresenius Medical Care Renal Pharma; payment or honoraria for lectures, presentations, speakers bureaus, manuscript writing, or educational events from Advicciene, Astellas, AstraZeneca, Amicus, Amgen, Fresenius Medical Care, GSK, Bayer, Sanofi-Genzyme, Menarini, Kyowa Kirin, Alexion, Idorsia, Chiesi, Otsuka, Novo Nordisk, and Vifor Fresenius Medical Care Renal Pharma; support for attending meetings and/or travel from Advicciene, Astellas, AstraZeneca, Fresenius Medical Care, Boehringer-Ingelheim, Bayer, Sanofi-Genzyme, Menarini, Chiesi, Otsuka, and Sysmex; participation on a Data Safety Monitoring Board or Advisory Board with Astellas, AstraZeneca, Boehringer-Ingelheim, Fresenius Medical Care, Bayer, Sanofi-Genzyme, Idorsia, Chiesi, Otsuka, Novo Nordisk, and Sysmex; leadership or fiduciary roles in board, society, committee or advocacy groups, unpaid with Council European Renal Association (ERA) Madrid Society of Nephrology (SOMANE); all outside the submitted work. A Radfar reports other financial and logistical support from Avicenna Medical and Clinical Research Institute to provide critical feedback and comments on important intellectual content on GBD manuscripts before publication. J Sanabria reports support for attending meetings and/or travel via Continuous Medical Education (CME) grant to their institution/department from the School of Medicine; patents granted and pending with no royalties; participation on a Data Safety Monitoring Board or Advisory Board with their institution/department for quality assessment and assurance; all outside the submitted work. S Sankararaman reports consulting fees from Nestle (US$1400) outside the submitted work. N Scarmeas reports grants or contracts from Novo Nordisk paid to their institution as local principal investigator of recruiting site for multinational, multicentre, industry sponsored phase 3 treatment trial for Alzheimer's disease; participation on a Data Safety Monitoring Board or Advisory Board with Albert Einstein College of Medicine for NIH-funded study Multicultural Healthy Diet to Reduce Cognitive Decline & AD Risk as Chair, and with Primus AD for public private funded Phase 2 study in Germany as Member (unpaid); all outside the submitted work. A E Schutte reports grants or contracts from NHMRC of Australia and Medical Research Future Fund of Australia; consulting fees from Abbott, Skylabs, Medtronic, and Servier; payment or honoraria for lectures, presentations, speakers bureaus, manuscript writing, or educational events from Medtronic, Servier, Omron, Aktiia, Sanofi, and Novartis; support for attending meetings and/or travel from Medtronic and Servier; leadership or fiduciary roles in board, society, committee, or advocacy groups, paid or unpaid with National Hypertension Taskforce of Australia as Co-Chair and Hypertension Australia and Australian Cardiovascular Alliance as Board Member; all outside the submitted work. V Shivarov reports one patent and one utility model planned, issued, or pending filed with the Bulgarian Patent Office; stock or stock options in ICON plc; other financial or non-financial support via a salary from ICON plc; all outside the submitted work. J A Singh reports consulting fees from ROMTech, Atheneum, Clearview Healthcare Partners, American College of Rheumatology, Yale University, Hulio, Horizon Pharmaceuticals, DINORA, ANI/Exeltis, USA Inc, Frictionless Solutions, Schipher, Crealta/Horizon, Medisys, Fidia, PK Med, Two Labs, Adept Field Solutions, Clinical Care Options, Putnam Associates, Focus Forward, Navigant Consulting, Spherix, MedIQ, Jupiter Life Science, UBM LLC, Trio Health, Medscape, WebMD, Practice Point Communications, and the NIH; payment or honoraria for speakers bureaus from Simply Speaking; past support for attending meetings and/or travel from OMERACT as a steering committee member; participation on a Data Safety Monitoring Board or Advisory Board (unpaid) with the US Food and Drug Administration Arthritis Advisory Committee; leadership or fiduciary roles in other board, society, committee, or advocacy groups with OMERACT as past steering committee member (paid), the Veterans Affairs Rheumatology Field Advisory Committee as Chair (unpaid), and the UAB Cochrane Musculoskeletal Group Satellite Center on Network Meta-analysis as Editor and Director (unpaid); stock or stock options in Atai Life Sciences, Kintara Therapeutics, Intelligent Biosolutions, Acumen Pharmaceutical, TPT Global Tech, Vaxart Pharmaceuticals, Atyu Biopharma, Adaptimmune Therapeutics, GeoVax Labs, Pieris Pharmaceuticals, Enzolytics, Seres Therapeutics, Tonix Pharmaceuticals Holding, Aebona Pharmaceuticals, and Charlotte's Web Holdings, and previously owned stock options in Amarin, Viking, and Moderna Pharmaceuticals; all outside the submitted work. J D Stanaway reports support for the present manuscript from the Bill & Melinda Gates Foundation via grants paid to their institution. A G Thrift reports grants or contracts paid to their institution from NHMRC (Australia), Heart Foundation (Australia), and Stroke Foundation (Australia); all outside the submitted work. J H V Ticoalu reports leadership or fiduciary roles in board, society, committee, or advocacy groups, paid or unpaid with Benang Merah Research Center, Indonesia as Co-Founder outside the submitted work. M Y Wei reports grants or contracts paid to their institution with NIH National Institute on Aging and Veterans Health Administration; leadership or fiduciary roles in board, society, unpaid committee, or advocacy groups with the Society of General Internal Medicine; all outside the submitted work.
