# Supplementary material for: Global, regional, and national progress towards the 2030 global nutrition targets and forecasts to 2050: a systematic analysis for the Global Burden of Disease Study 2021
Source: Lancet. 2024 Dec 21;404(10471):2543–83. doi: 10.1016/S0140-6736(24)01821-X (PMC11703702; doi:10.1016/S0140-6736(24)01821-X)
Supplement: Supplementary appendix 1 [file mmc1.pdf]

THE LANCET

Supplementary appendix 1

This appendix formed part of the original submission and has been peer reviewed. We post it as supplied by the authors.

Supplement to: Global Nutrition Target Collaborators. Global, regional, and national progress towards the 2030 global nutrition targets and forecasts to 2050: a systematic analysis for the Global Burden of Disease Study 2021. *Lancet* 2024; published online Dec 9. https://doi.org/10.1016/S0140-6736(24)01821-X.

Appendix to "Global, regional, and national progress towards the 2030 global nutrition targets and forecasts to 2050: a systematic analysis for the Global Burden of Disease Study 2021"

Table of Contents

Table of Contents 1
GATHER checklist 4
Supplementary methods 5
Dimensions of GBD 5
Overview and definitions 5
Low birthweight 5
Exclusive breastfeeding 6
Stunting and wasting 6
Overweight 6
Anaemia 6
Data identification and processing 7
Low birthweight 7
Exposure 7
Data identification 7
Data processing 8
Relative risk 8
Exclusive breastfeeding 9
Exposure 9
Data identification 9
Relative risk 10
Stunting and wasting 10
Exposure 10
Data identification 10
Data processing 11
Relative risk 11
Overweight 12
Data identification 13
Data processing 14

Anaemia 14
Data identification 14
Data processing 14
Indicator modelling 16
Low birthweight 16
Exposure 17
Relative risks & TMREL 20
Exclusive breastfeeding 21
Exposure 21
TMREL 22
Relative risk 22
Stunting and wasting 23
Exposure 23
TMREL 24
Relative risk 24
Overweight 28
Exposure 28
TMREL 29
Relative risk 29
Anaemia (women of reproductive age) 29
Modelling strategy 29
Central computation for deaths, YLLs, YLDs, and DALYs 32
YLD calculation 32
Comorbidity simulation (COMO) 32
CoDCorrect 32
Years of life lost calculation 33
DALYnator 33
DALYs: the sum of YLLs and YLDs 33
Population attributable fraction 33
SEVs 33
Attributable burden 34
SDI analysis and forecasting 34
SDI overview 35

SDI calculation 35
Epidemiological transitions 36
MR-BRT models 36
Expected annualised rate of change based on SDI 37
SDI forecasting 38
Education 38
Lag-distributed Income 39
Fertility 40
Prevalence forecasting 41
SEV forecasting 41
Using SEV forecasts to predict future prevalence 42
Model training and performance refinement 42
MR-BRT methods[1] 43
Cascading spline methods 43
Prediction, intercept shifting, and aggregation 44
Forecasting population 45
Livebirth forecasts 45
Custom forecasts of new child age groups 45
Future population of ages 1–5 months 45
Future population of ages 2–4 years 46
Intercept shifting 46
Demographic shifts 46
Software packages 47
Figure A5 Prevalence data availability for GNT indicators by GBD region, 1990−2021 48
Supplemental Results: Figures 49
Supplemental Results: Tables 49
References 50

GATHER checklist

Table A1. GATHER checklist

Item #	Checklist item	Reported on page #
**Objectives and funding**		
**1**	Define the indicator(s), populations (including age, sex, and geographic entities), and time period(s) for which estimates were made.	Main manuscript introduction and methods and appendix methods
**2**	List the funding sources for the work.	Main manuscript funding statement
**Data Inputs**		
For all data inputs from multiple sources that are synthesized as part of the study:		
**3**	Describe how the data were identified and how the data were accessed.	Main manuscript methods and appendix (7-16)
**4**	Specify the inclusion and exclusion criteria. Identify all ad-hoc exclusions.	Main manuscript methods and appendix methods (7-16)
**5**	Provide information on all included data sources and their main characteristics. For each data source used, report reference information or contact name/institution, population represented, data collection method, year(s) of data collection, sex and age range, diagnostic criteria or measurement method, and sample size, as relevant.	Main manuscript methods and appendix methods. Detailed data sources for each component available online at Global Burden of Disease Study 2021 (GBD 2021) Sources Tool | GHDx (healthdata.org)
**6**	Identify and describe any categories of input data that have potentially important biases (e.g., based on characteristics listed in item 5).	Main manuscript methods and appendix methods
For data inputs that contribute to the analysis but were not synthesized as part of the study:		
**7**	Describe and give sources for any other data inputs.	Main manuscript methods and appendix methods (7-16)
For all data inputs:		
**8**	Provide all data inputs in a file format from which data can be efficiently extracted (e.g., a spreadsheet rather than a PDF), including all relevant meta-data listed in item 5. For any data inputs that cannot be shared because of ethical or legal reasons, such as third-party ownership, provide a contact name or the name of the institution that retains the right to the data.	Detailed data sources for each component available online Global Burden of Disease Study 2021 (GBD 2021) Sources Tool | GHDx (healthdata.org)
**Data analysis**		
**9**	Provide a conceptual overview of the data analysis method. A diagram may be helpful.	Main manuscript methods and appendix methods (16-46)
**10**	Provide a detailed description of all steps of the analysis, including mathematical formulae. This description should cover, as relevant, data cleaning, data pre-processing, data adjustments and weighting of data sources, and mathematical or statistical model(s).	Main manuscript methods and appendix methods (16-46)
**11**	Describe how candidate models were evaluated and how the final model(s) were selected.	Appendix methods (19-46)
**12**	Provide the results of an evaluation of model performance, if done, as well as the results of any relevant sensitivity analysis.	
**13**	Describe methods for calculating uncertainty of the estimates. State which sources of uncertainty were, and were not, accounted for in the uncertainty analysis.	Main manuscript methods and appendix methods (16-46)
**14**	State how analytic or statistical source code used to generate estimates can be accessed.	All statistical code for each component available online at GBD Code Repository (https://ghdx.healthdata.org/gbd/2021/code)
**Results and Discussion**		

**15**	Provide published estimates in a file format from which data can be efficiently extracted.	GBD 2021 results are publicly available from the GHDx online Results Tool (http://ghdx.healthdata.org/gbd-results-tool)
**16**	Report a quantitative measure of the uncertainty of the estimates (e.g. uncertainty intervals).	Main manuscript results text includes 95% uncertainty intervals. Uncertainty intervals are also present in the online Results Tool.
**17**	Interpret results in light of existing evidence. If updating a previous set of estimates, describe the reasons for changes in estimates.	Main manuscript discussion
**18**	Discuss limitations of the estimates. Include a discussion of any modelling assumptions or data limitations that affect interpretation of the estimates.	Main manuscript discussion

This checklist should be used in conjunction with the GATHER statement and Explanation and Elaboration document, found on gather-statement.org

Supplementary methods

Dimensions of GBD

This analysis uses estimates from the Global Burden of Diseases, Injuries, and Risk Factors (GBD) Study 2021, which evaluated age, geographical, and temporal patterns for 369 diseases and injuries and 87 risks.[1] The GBD methods and input data comply with the Guidelines for Accurate and Transparent Health Estimates Reporting (GATHER) and are reported in **Table A1**.

The GBD estimates health loss for 204 countries and territories grouped across 21 regions and seven super-regions, with additional subnational estimates produced for 21 countries (Italy, Nigeria, Pakistan, the Philippines, Poland, Brazil, China, Ethiopia, India, Indonesia, Iran, Japan, Kenya, Mexico, New Zealand, Norway, Russia, South Africa, Sweden, the UK, and the USA) at a more granular level. The GBD produces prevalence and disability estimates between the years 1990 and 2020, and cause-specific mortality estimates for years 1980 to 2021, for 23 age groups and two sexes. We note that sex and gender are not mutually exclusive and that while sex is a biological variable most often assigned at birth, gender is a social construct. Due to limitations in data collection, data presented here refer only to males and females.

Within the GBD, diseases and injuries are organised in a tiered cause hierarchy from Level 1, being most broad, to Level 4, being most specific. All input data for all data measures used to generate estimates within the GBD are publicly available in the Global Health Data Exchange (GHDx) interactive tool, and further visualisations outside the scope of this analysis using GBD 2019 results can be viewed in the GBD Compare tool at https://vizhub.healthdata.org/gbd-compare/.

Overview and definitions

Low birthweight

Short gestational age and low birthweight are highly correlated risk factors associated with poor child health outcomes. The “low birthweight and short gestation” (LBWSG) risk factor quantifies the burden of disease attributable to increased risk of death and disability due to 1) less than ideal birthweight (“low birthweight”) and 2) shorter than ideal length of gestation (“short gestation”). In GBD 2016,

LBWSG became the first (and, as of GBD 2019, only) group of GBD risk factors in which combined attributable burden is quantified by direct estimation of the joint exposure, relative risk, theoretical minimum risk exposure level (TMREL), and population attributable fraction (PAF) of multiple risk factors. Details about the rationale for this choice have been provided in a previous GBD publication.[1]

"Low birthweight" has historically referred to any birthweight less than 2500 grams, dichotomising birthweight into two categories: "normal" and "low". In the context of the GBD LBWSG risk factor, low birthweight refers to any birthweight less than the birthweight TMREL (the birthweight that minimises risk at the population level). Because LBWSG is estimated in a grid of 500-gram and two-week bins, any 500-gram birthweight unit less than the TMREL, which was determined as [38, 40) weeks and [3500, 4000) g for the LBWSG parent risk factor, is considered "low birthweight". This includes, for example, birthweight of [2500, 3000) grams, which the traditional, dichotomous definition of "low birthweight" would not include.

Like birthweight, gestational age is typically classified into broad categories. "Preterm" is used to describe any newborn baby born less than 37 completed weeks of gestation. In the GBD context, "short gestation" is used to refer to all gestational ages below the gestational age TMREL.

Exclusive breastfeeding

Non-exclusive breastfeeding is defined in GBD as the proportion of children who are not exclusively breastfed. We then parse those not exclusively breastfed into three categories – predominant, partial, and no breastfeeding. Exclusive breastfeeding is 1 minus the non-exclusive breastfeeding proportion, and is defined as the proportion of children under 6 months of age who receive no other food or drink except breastmilk (allowing for oral rehydration therapy, drops, or syrups containing vitamins, minerals, or medicines).[2,3]

Stunting and wasting

Stunting (height-for-age; HAZ) and wasting (weight-for-height; WHZ) are defined using the age- and sex-specific WHO growth standards for children aged 0–59 months.[4] These standards were developed in the 2006 WHO Multicenter Growth Reference Study, which followed an international cohort of ideally nourished children. HAZ and WHZ are measured in terms of Z-scores from these growth standards curve medians.

Overweight

High BMI for children (ages 1–19) is defined as being overweight or obese based on International Obesity Task Force standards.[1,5]

Girls: BMI of 18.09 (24 months) – 17.23 (59 months)

Boys: BMI of 18.36 (24 months) – 17.39 (59 months)

Anaemia

Anaemia is defined as decreased blood concentration of haemoglobin, irrespective of underlying cause, red blood cell morphology, or red blood cell function. Thresholds for defining individuals as being anaemic, as well as thresholds for anaemia severity, are based on WHO thresholds for haemoglobin in g/L.[6] Anaemia thresholds among women of reproductive age vary by pregnancy status as a result of physiological changes during pregnancy. The thresholds used in this analysis for women of reproductive age are detailed in **Table A2**.

Table A2. Definitions of mild, moderate, and severe anaemia based on blood haemoglobin concentration

Sex	Mild	Moderate	Severe
Female, non-pregnant	110–119 g/L	80–109 g/L	<80 g/L
Female, pregnant	100–109 g/L	70–99 g/L	<70 g/L

Data identification and processing

Low birthweight

Exposure

Data identification

Input data needed to model univariate gestational age and birthweight distributions at birth (Step A) are:

- Prevalence of preterm birth (<37 weeks), by l/y/s
- Prevalence of preterm birth (<28 weeks), by l/y/s
- Mean gestational age, by l/y/s
- Gestational age microdata
- Prevalence of low birthweight (<2500 grams), by l/y/s
- Mean birthweight, by l/y/s
- Birthweight microdata

Microdata are the ideal data source for modelling distributions; however, microdata are not widely available for birthweight and are scarcer for gestational age. Categorical prevalence data are more readily available from a wider range of locations and years for low birthweight (<2500g), extremely preterm (<28 weeks of gestation), and preterm birth (<37 weeks of gestation). Because categorical prevalence has wider availability than microdata, we use prevalence data to assist in modelling birthweight and gestational age ensemble distributions.

To model joint distributions of gestational age and birthweight (Step B), joint microdata of gestational age and birthweight are also required. Additional inputs to modelling joint distributions from birth to 28 days (Step C) are all-cause mortality by l/y/s and joint birthweight and gestational age microdata linked to mortality outcomes. Prevalence of extremely preterm birth (<28 weeks) and preterm birth (<37 weeks) were modelled using vital registration, survey, and clinical data. For the preterm models, only inpatient and insurance claims data were included from clinical informatics datasets; outpatient data were excluded because they were more likely to capture repeated visits by the same child rather than unique visits. Prevalence of low birthweight was modelled using only vital registration and survey data.

Before GBD 2016, available preterm birth data were sourced by a technical working group. In GBD 2016 and GBD 2017, we conducted systematic reviews to identify additional sources beyond the data already used in the models. Search terms used in the systematic review have been published previously.[1]

Table A3. LBWSG search hits, full-text review, extracted sources

Search	Hits	Full-text review	Extracted	Search date
GBD 2017	16,174	2200	154	6/6/2017

Table A4. Input data for exposure models

Input data	Exposure

Source count (total)	2233
Number of countries with data	176

Data processing

Starting in GBD 2019, as was the case with all other non-fatal analyses, we applied empirical age and sex ratios from previous GBD 2019 Decomposition 1 models to disaggregate observations that did not entirely fit in one GBD age category or sex. Ratios were determined by dividing the result for a specific age and sex by the result for the aggregate age and sex specified in a given observation.

Low birthweight data were extracted from literature, vital registration systems, and surveys. Survey data (most commonly from DHS and MICS) were observed to have high missingness birthweight responses. We evaluated the patterns of missingness and found a number of distinct patterns that suggested non-random omission of birthweight observations. We therefore imputed missing birthweight values using the Amelia II (Version 1.7.6) package in R. Birthweight was predicted using standard Amelia imputation methods from the following variables also in the DHS surveys: urbanicity, sex, birthweight recorded on card, birth order, maternal education, paternal education, child age, child weight, child height, mother's age at birth, mother's weight, shared toilet facility, and household water treated.

"Crosswalking", or the process of reducing non-random bias by adjusting non-standard data to the likely value had the data been "gold-standard", was used to process data in the extremely preterm (28 weeks) and preterm (<37 weeks) models. All preterm crosswalks were done using meta-regression—Bayesian, regularised, trimmed (MR-BRT) tool.[1] Additional details on the data processing can be found in a prior GBD publication.[1]

These data adjustments had the effect of dramatically increasing the size of each of the modelling datasets and are primarily responsible for most changes in preterm estimates between GBD 2019 and GBD 2021. After all crosswalks, we performed a deduplication step on GA models. Namely, if low birthweight data in countries that were 1) categorised as "data-rich" locations in cause-of-death modelling or had at least 10 consecutive years of vital registration data recording gestational age, and 2) had both preterm birth and low birthweight data, then crosswalked low birthweight data were outliered so that the model was informed only by the gestational age data.

Relative risk

LBWSG is paired with the outcomes listed in Table 15 and is only attributed to burden in the early and late neonatal period.

Table A5. Cause list of outcomes for low birthweight and short gestation

Cause name
Diarrhoeal diseases
Lower respiratory infections
Upper respiratory infections
Otitis media
Pneumococcal meningitis
H influenzae type B meningitis
Meningococcal meningitis

Other meningitis
Encephalitis
Neonatal preterm birth complications
Neonatal encephalopathy due to birth asphyxia and trauma
Neonatal sepsis and other neonatal infections
Haemolytic disease and other neonatal jaundice
Other neonatal disorders
Sudden infant death syndrome

The available data for deriving relative risk was only for all-cause mortality. The exception was the USA linked infant birth-death cohort data, which contained three-digit ICD causes of death, but also had nearly 30% of deaths coded to causes that are ill-defined, or intermediate, in the GBD cause classification system. We analysed the relative risk of all-cause mortality across all available sources and selected outcomes based on criteria of biological plausibility. Some causes, most notably congenital birth defects, haemoglobinopathies, malaria, and HIV/AIDS, were excluded based on the criteria that reverse causality could not be excluded.

In Norway, New Zealand, and USA Linked Birth/Death Cohort microdata datasets, livebirths are reported with gestational age, birthweight, and an indicator of death at 7 days and 28 days. For this analysis, gestational age was grouped into two-week categories, and birthweight was grouped into 500-gram categories. The Taiwan, Japan, and Singapore datasets were prepared in tabulations of joint 500-gram and two-week categories. A pooled country analysis of mortality risk in the early neonatal period and late neonatal period by "small for gestational age" category in developing countries in Asia and sub-Saharan Africa were also used to inform the relative risk analysis.

Table A6. Input data for low birthweight and short gestation relative risk models

Input data	Relative risk
Source count (total)	113
Number of countries with data	6

Exclusive breastfeeding

Exposure

Data identification

The data used in the analysis consisted mostly of processed individual-level microdata from surveys; in the cases where microdata were unavailable, we used reported tabulated data from survey reports and scientific literature. Data used to categorise type of non-exclusive breastfeeding (predominant, partial, and none) came from surveys with 24-hour dietary logs based on maternal recall.

We updated our systematic review in GBD 2021 by searching the Global Health Data Exchange (GHDx) using the keyword "breastfeeding." We prioritised extraction of surveys with microdata and new surveys from major survey series such as Demographic and Health Surveys (DHS) and Multiple Indicator Cluster Surveys (MICS).

Table A7. Input data counts – suboptimal breastfeeding exposure

	Countries with data	New sources	Total sources
Non-exclusive breastfeeding	169	49	737
Discontinued breastfeeding	162	50	679

Relative risk

We included outcomes based on the strength of available evidence supporting a causal relationship. Studies evaluating the causal evidence for our risk-outcome pairs came primarily from studies compiled in a published review by the World Health Organization.[7] Non-exclusive breastfeeding was paired with diarrhoea and lower respiratory infections as disease outcomes.

Table A8. Input data counts – suboptimal breastfeeding relative risk

	Countries with data	New sources	Total sources
Relative risk	26	0	43

Stunting and wasting

Exposure

Data identification

We included data from population-representative surveys, administrative data sources, and published scientific literature. These sources can be categorised into three main data types: 1) age- and sex-specific microdata from population surveys, 2) tabulated reports, and 3) the WHO Global Database on Child Growth and Malnutrition.[8] Here, microdata refers to a cross-sectional data source with individual-level observations of height, weight, and age. The primary data additions in GBD 2021 for child growth failure were from population surveys that include anthropometry. Population surveys include a variety of multi-country and country-specific survey series such as Multiple Indicator Cluster Surveys (MICS), Demographic and Health Surveys (DHS), Living Standards Measurement Surveys (LSMS), and the China Health and Nutrition Survey (CHNS), as well as other one-time country-specific surveys such as the Indonesia Family Life Survey and the Brazil National Demographic and Health Survey of Children and Women. Tabulated reports contain sample sizes and prevalences of categorical forms of CGF, which may be reported in an age- and sex-specific fashion, or collectively for children under 5 years of age. These are often Demographic and Health Surveys and Multiple Indicator Cluster Surveys. The WHO Global Database on Child Growth and Malnutrition contains a large collection of tabulation sources which also contain sample sizes and prevalences of categorical forms of CGF. Studies that were not representative of a geography's population were excluded. Any study with self-reported height and weight values (as opposed to measurements from examinations) was also excluded.

Table A9. Input data counts for stunting exposure models

Input data	Exposure
Source count (total)	1897
Number of countries with data	160

Table A10. Input data counts for wasting exposure models

Input data	Exposure
Source count (total)	1908
Number of countries with data	159

Data processing

Only data sources that were representative of a location's entire population were included in the analysis to avoid sampling bias. Clinical trials that recruited specific populations, studies that implemented interventions that could have affected measurements, or analyses that were in specific non-representative locations were excluded. Four data cleaning steps were performed to maximise internal consistency of the modelling dataset. First, microdata that reported height, weight, and age were converted into stunting, wasting, and underweight Z-scores using the 2006 Child Growth Standards and the LMS method.[9] Data that did not meet the following three criteria were dropped: 1) non-sex-specific data, 2) data with invalid Z-scores (HAZ, WAZ, WHZ, or BMI above 6 SD or below -6 SD), and 3) data with impossible values (negative height, weight, or age). Data sources that only include mid-upper arm circumference measurements but not weight-for-height were excluded. Second, any tabulation data that were reported using the National Center for Health Statistics (NCHS) 1978 growth standards were transformed to corresponding values on the WHO 2006 Growth Standards curves. This was done using an OLS linear regression model and informed by a study that evaluated concordance between the two growth standards. Only overall (Z-score < –2SD) CGF prevalences were transformed, as this is where concordance was highest. Third, for any study that lacked a measure of mean Z-score for stunting, wasting, or underweight, we predicted a mean value for that study. To accomplish this, an ordinary-least-squares regression of mean Z-score versus overall (Z-score < –2SD) CGF prevalence was conducted for all sources where both were reported. Lastly, any data that were reported as both sexes combined, or for age groups broader than the GBD 2021 age groups for children under 5 (0–6 days, 7–27 days, 1–5 months, 6–11 months, 12–23 months, 2–4 years), were split into these most detailed groups. This process was conducted by applying the age and sex pattern from an ST-GPR (spatiotemporal Gaussian process regression) model which only included sources with age- and sex-specific data. Any data with sample sizes less than 5 after age sex disaggregation were excluded.

Relative risk

The outcomes paired with child growth failure (CGF) risks included morbidity and mortality from lower respiratory infections (LRI), diarrhoea, measles, malaria, and protein-energy malnutrition (PEM). We attributed 100% of PEM to childhood wasting and underweight but not stunting. The analysis incorporated both a pooled analysis of ten prospective cohort studies by Olofin and colleagues[10] and relative risk estimates from 26 longitudinal studies in locations across sub-saharan Africa, Asia, and Latin America conducted between 1989 and 2017 (**Table A11**).

Table A11. Input data for Child growth failure relative risk models

Study name	Country	Sample Size	Years conducted
Zimbabwe Vitamin A for Mothers and Babies Trial	ZWE	14,110	1997-2001
CMC Vellore Birth Cohort Study	IND	373	2002-2006
International Lipid-Based Nutrient Supplements Project	MWI	1,206	2011-2014
Malnutrition and Enteric Disease Study	BGD	265	2009-2017
Malnutrition and Enteric Disease Study	IND	251	2009-2017
Malnutrition and Enteric Disease Study	NEP	240	2009-2017
Malnutrition and Enteric Disease Study	PER	303	2009-2017
Malnutrition and Enteric Disease Study	BRA	233	2009-2017
Malnutrition and Enteric Disease Study	ZAF	314	2009-2017
Malnutrition and Enteric Disease Study	TZA	262	2009-2017
Medical Research Council Keneba	GMB	2,867	-
Performance of Rotavirus and Oral Polio Vaccines In Developing Countries	BGD	700	2011-2014
Community-based Intervention Trial to Compare the Impact of Preventive and Therapeutic Zinc Supplementation Programs Among Young Children in Burkina Faso	BFA	7,634	2010-2012
WASH Benefits Bangladesh	BGD	4,423	2011-2014
WASH Benefits Kenya	KEN	5,649	2012-2016
Promotion of Breastfeeding Intervention Trial	BLR	16,897	1996-1998
Childhood Malnutrition and Infection Network	BGD	477	1993-1996
Childhood Malnutrition and Infection Network	BRA	119	1989-1998
Childhood Malnutrition and Infection Network	GNB	350	1987-1990
Childhood Malnutrition and Infection Network	GNB	885	1996-1997
Childhood Malnutrition and Infection Network	PER	210	1989-1991
Childhood Malnutrition and Infection Network	PER	224	1995-1998
Delhi Infant Vitamin D Study	IND	2,100	2007-2010
Characterization of Respiratory pathogens endemic to Pakistan in pregnant women and newborns in urban settings	PAK	380	2012-2013
Impact of Zinc Supplementation in Low Birth Weight Infants on Severe Morbidity, Mortality and Zinc Status: A Randomized Controlled Trial	IND	2,052	2005-2007
A Trial of Zinc and Micronutrients in Tanzanian Children	TZA	2,400	2007-2012

Overweight

We conducted a systematic review in GBD 2017 to identify studies providing nationally or subnationally representative estimates of overweight prevalence, obesity prevalence, or mean body-mass index (BMI). We included representative studies providing data on mean BMI or prevalence of overweight or obesity among children ages 2–19. Studies were included if they used International Obesity Task Force (IOTF) standards to define overweight and obesity thresholds. We only included studies reporting data collected after January 1, 1980. Studies were excluded if they used non-random samples (eg, case-control studies or convenience samples), conducted among specific subpopulations, used alternative methods to assess adiposity (eg, waist circumference, skin-fold thickness, or hydro densitometry), had

sample sizes of less than 20 per age-sex group, or provided inadequate information on any of the inclusion criteria. We also excluded review articles and non-English-language articles. In GBD 2021, new data were added from sources included in the annual GHDx update of known survey series.

Data identification

Where individual-level survey data were available, we computed mean BMI using weight and height. We then used BMI to determine the prevalence of overweight and obesity. For individuals aged 2–19 years, we used monthly IOTF cutoffs to determine overweight and obese status when age in months was available. When only age in years was available, we used the cutoff for the midpoint of that year. Obese individuals were also considered to be overweight. We excluded studies using the World Health Organization (WHO) standards or country-specific cutoffs to define childhood overweight and obesity. At the individual level, we considered BMI 70 kg/m^2 to be biologically implausible and excluded those observations. The rationale for choosing to use the IOTF cutoffs over the WHO standards has been described elsewhere.[1] Briefly, WHO defines overweight in children under 5 years as weight-for-height z-score greater than 2 standard deviations above the median from the child growth standards based on multinational longitudinal cohort, and uses the WHO growth reference for defining overweight with BMI in children ages 5–19. The WHO growth reference for children ages 5–19 uses the CDC standards that were derived from United States data, which are less representative than the multinational data used by IOTF. The IOTF used a different approach and modelled sex-specific BMI centile curves in each of six country-specific longitudinal cohorts from ages 2–18. The IOTF determined which BMI centile curve passed through BMI of 25 (overweight) and 30 (obesity) when reaching adulthood (age 18) and averaged these curves to create age-sex-specific curves for each cutoff. In GBD, a switch between references and metrics (WHZ versus BMI) at age 5 could produce artificial discontinuities. Given that GBD estimates global childhood overweight and obesity for ages 2–19 (with age 19 using standard adult cutoffs), the IOTF cutoffs were preferable.

From report and literature data, we extracted data on mean BMI, prevalence of overweight, and prevalence of obesity, measures of uncertainty for each, and sample size, by the most granular age and sex groups available. Additionally, we extracted the same study-level covariates as were extracted from microdata (measurement, urbanicity, and representativeness), as well as location and year.

In addition to the primary indicators described above, we extracted relevant survey-design variables, including primary sampling unit, strata, and survey weights, which were used to tabulate individual-level microdata and produce accurate measures of uncertainty. We extracted three study-level covariates: 1) whether height and weight data were measured or self-reported; 2) whether the study was predominantly conducted in an urban area, rural area, or both; and 3) the level of representativeness of the study (national or subnational).

Finally, we extracted relevant demographic indicators, including location, year, age, and sex. We estimated the standard error of the mean from individual-level data, where available, and used the reported standard error of the mean for published data. When multiple data sources were available for the same country, we included all of them in our analysis. If data from the same data source were available in multiple formats such as individual-level data and tabulated data, we used individual-level data. Additional details are available from a prior GBD publication.[1]

Table A12. Data inputs for exposure for high body-mass index (all ages).

Input data	Exposure
Source count (total)	2016
Number of countries with data	194

Data processing

Any report or literature data provided in age groups wider than the standard five-year age groups or as both sexes combined were split using the approach used by Ng and colleagues.[11] Briefly, age-sex patterns were identified using sources with data on multiple age-sex groups, and these patterns were applied to split aggregated report and literature data. Uncertainty in the age-sex split was propagated by multiplying the standard error of the data by the square root of the number of splits performed. We did not propagate the uncertainty in the age pattern and sex pattern used to split the data as they seemed to have small effect.

We included both measured and self-reported data. We tested for bias in self-report data compared to measured data, which is considered to be the gold-standard. There was no clear direction of bias for children ages 2–14, so for these age groups we only included measured data.

Anaemia

Data identification

To estimate total anaemia (called "the envelope"), we utilised data from a variety of sources. Inclusion criteria consisted of quantitative measurement of haemoglobin in either a population-based sample or group judged to adequately represent the sex, age groups, and location of the study.

Population-based surveys including the Demographic and Health Survey (DHS), Multiple Indicator Cluster Survey (MICS) series, national micronutrient surveys, and other national and subnational nutrition surveys comprised the bulk of input data. We extracted all sources with individual-level data into GBD age groups by sex and pregnancy status in six different formats: mean haemoglobin concentration, severe anaemia prevalence, moderate anaemia prevalence, moderate+severe anaemia prevalence, mild anaemia prevalence, and total anaemia prevalence. We supplemented these data with additional sources of mean haemoglobin concentration and anaemia prevalence from the WHO Vitamin and Mineral Nutrition Information System[4] and other literature sources, where available. The complete list of input data sources used to estimate anaemia prevalence can be found in the supplemental materials of a prior publication[12] .

Data processing

Method of blood sampling and method of testing

Most surveys used a HemoCue test, adjusted for altitude, and excluded those with terminal or acute medical conditions. Published scientific literature studies and those from higher-income locations typically measured haemoglobin with a Coulter counter. Both of these methods operate by reacting haemoglobin with a specific reagent (Drabkin's solution) and measuring absorbance wavelengths and were treated as equivalent for this analysis. We also did not make any formal distinction in data processing between studies that drew whole blood from participants and those that completed capillary venous sampling. Further investigation is needed to determine if formal data adjustment for HemoCue and capillary venous sampling is needed or if their additional variability, which leads to higher uncertainty in input data sources (and intrinsically lower influence on the model), is sufficient.

Altitude adjustment and smoking adjustment

Haemoglobin concentration increases with increasing elevation, a physiological response to lower ambient oxygen levels that aims to maintain oxygen delivery throughout the body. Under 1000 meters, there appears to be little effect on haemoglobin, but previous studies have suggested an exponentially increasing effect of elevation, as illustrated by **Figure A1**, which is the WHO-recommended formula for haemoglobin adjustment.

We used all survey-reported, altitude-adjusted, or altitude- and smoking-adjusted haemoglobin data directly without further adjustment. Using the equation above, we adjusted individual-level data that did not present altitude-adjusted haemoglobin values but did include altitude levels. Testing alternative approaches for altitude adjustment is an area for further investigation. No additional adjustments were made for smoking in the GBD 2021 analysis.

Figure A1. Haemoglobin adjustment for altitude

WHO haemoglobin adjustment factor (difference in haemoglobin) by altitude in meters. Adjustments are based on the formula: $\Delta Hb = -0.32 \times (\text{altitude in meters} \times 0.0033) + 0.22 \times (\text{altitude in meters} \times 0.0033)^2$

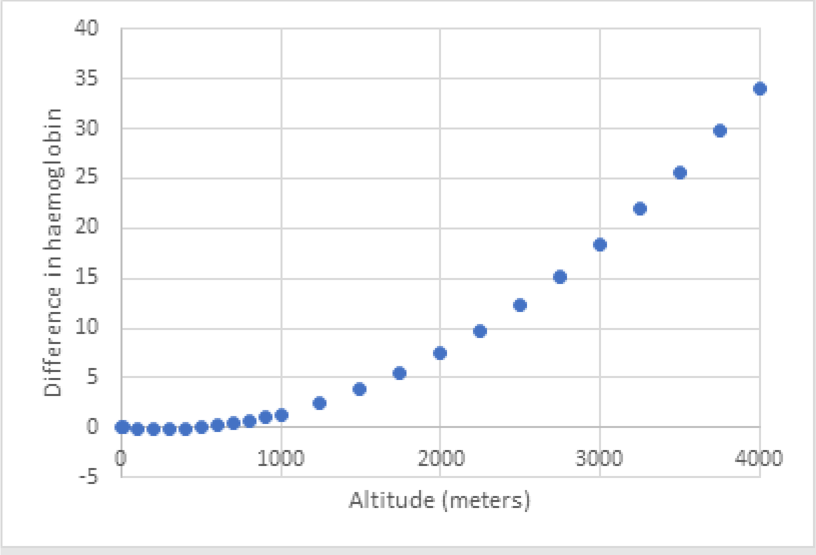

Age-sex splitting and crosswalking data by pregnancy status

We pooled all data that fit entirely within single GBD age-sex groups to calculate a global age and sex pattern of mean haemoglobin concentration and anaemia prevalence by severity. We then used these global age and sex patterns to proportionally split any datum that spanned more than a single GBD age-sex group into the age- and sex-specific GBD groups.

Haemoglobin concentration varies systematically based on pregnancy status. Our models of mean haemoglobin concentration and anaemia prevalence are based on these values among non-pregnant females, and these estimates are later adjusted to account for pregnancy prevalence for each location/year/age/sex as described in the "Fitting ensemble distributions with method of moments" methods section included below. Studies that only reported mean haemoglobin among pregnant women were crosswalked to fit our model case definition of non-pregnant women. To estimate this crosswalk, we matched data sources where we had age-specific estimates of mean haemoglobin separately by pregnancy status and calculated the ratio of these values among pregnant females versus

non-pregnant females within a single study. We log-transformed these ratios and calculated standard errors of the ratios using the delta method. We then meta-analysed these ratios using MR-BRT with a 10% trim setting. Although age was tested as a potential predictor in the model, we did not observe a significant age dependence of the ratios. The crosswalk effects are illustrated below.

Table A13. MR-BRT mean haemoglobin crosswalk values

Model	Data input	Reference or alternative case definition	Gamma	Beta coefficient, log (mean [Hb]) (95% UI)*	Adjustment factor**
**Mean haemoglobin**	Non-pregnant women	Ref	0.033	---	---
	Pregnant women	Alt		–0.09 (–0.15 to –0.02)	0.92 (0.86 to 0.98)

**MR-BRT crosswalk adjustments can be interpreted as the factor the alternative case definition is adjusted by to reflect what it would have been had it been measured using the reference case definition. If the log/logit beta coefficient is negative, then the alternative is adjusted up to the reference. If the log/logit beta coefficient is positive, then the alternative is adjusted down to the reference.*
***The adjustment factor column is the exponentiated beta coefficient. For log beta coefficients, this is the relative rate between the two case definitions. For logit beta coefficients, this is the relative odds between the two case definitions.*

Figure A2. MR-BRT mean haemoglobin crosswalk funnel plot

Funnel plot for MR-BRT meta-analysis of mean haemoglobin by pregnancy status, standard error versus effect size. Each datapoint represents a within-study, age-specific ratio of haemoglobin between pregnant and non-pregnant women (log-transformed).

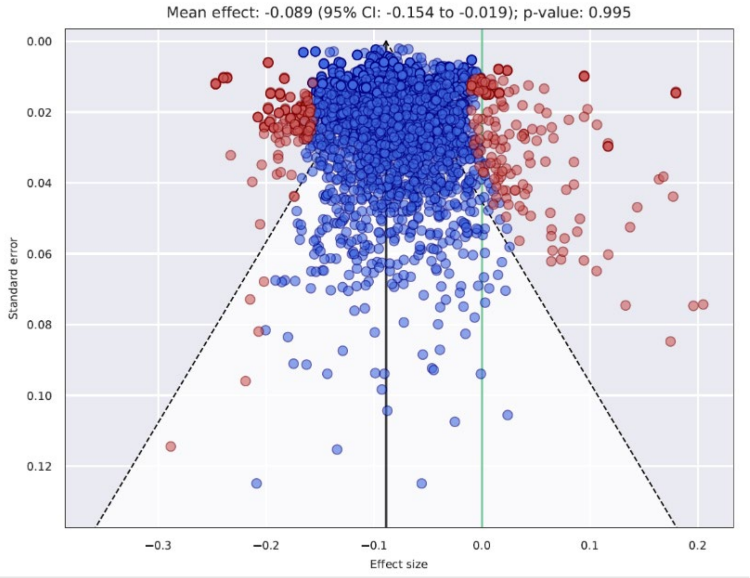

Indicator modelling

Low birthweight

The following section is copied from the appendix of GBD 2021 risk factors publication.[1]

Exposure

As mentioned in the Overview and definitions section above, low birthweight short gestation (LBWSG) is a group of GBD risk factors in which combined attributable burden is quantified by direct estimation of the joint exposure, relative risk, TMREL, and PAF of multiple risk factors.

In GBD 2016, LBWSG became the first (and, as of GBD 2021, only) group of GBD risk factors in which combined attributable burden is quantified by direct estimation of the joint exposure, relative risk, TMREL, and PAF of multiple risk factors. After first directly estimating the joint exposure, relative risk, TMREL, and PAF of birthweight and gestational age together, we then separate out the independent PAFs due to birthweight only or gestational age only. Because of this modelling strategy, the joint GBD risk factor quantifying the burden of disease due to both less than ideal birthweight ("low birthweight") and shorter than ideal gestational age ("short gestation") is grouped into a single "parent" risk factor termed "low birthweight and short gestation". LBWSG is disaggregated into two "child" risk factors: "low birthweight for gestation" and "short gestation for birthweight". Low birthweight for gestation quantifies the burden of disease attributable to less than ideal birthweight, after adjusting for the influence of gestational age. Likewise, short gestation for birthweight quantifies the burden of disease attributable to shortened gestational age, after adjusting for the influence of birthweight.

Ideally, the model for joint exposure and joint relative risk would be fully continuous. To simplify the computation for the analysis, a grid of 500-gram and 2-week units ("bins") is used as the LBWSG dimensions and to approximate a fully continuous joint distribution model (see **Figure A3**).

Figure A3. Fully continuous analysis of joint gestational age and birthweight (left) is approximated with a grid of birthweight and gestational age with 500-gram and 2-week "bins" (right)

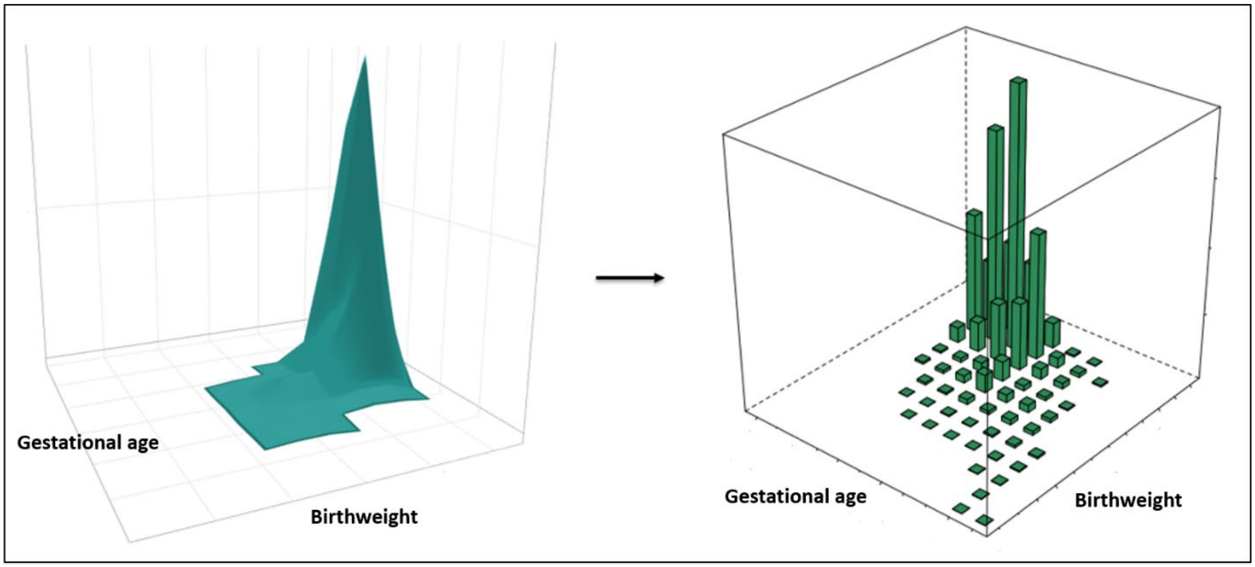

LBWSG is paired with the outcomes listed in Table A14 and is only attributed to burden in the early and late neonatal period.

Table A14. Cause list of outcomes for low birthweight and short gestation

Cause name
Diarrhoeal diseases

Lower respiratory infections
Upper respiratory infections
Otitis media
Pneumococcal meningitis
H influenzae type B meningitis
Meningococcal meningitis
Other meningitis
Encephalitis
Neonatal preterm birth complications
Neonatal encephalopathy due to birth asphyxia and trauma
Neonatal sepsis and other neonatal infections
Haemolytic disease and other neonatal jaundice
Other neonatal disorders
Sudden infant death syndrome

In LBWSG, exposure refers to the portion of the joint distribution of gestational age and birthweight less than the TMREL, by location/year/sex (l/y/s), from birth to the end of the neonatal period. Modelling LBWSG exposure can be summarised in three steps:

A. Model univariate gestational age and birthweight distributions at birth, by l/y/s
B. Model joint distributions of gestational age and birthweight at birth, by l/y/s
C. Model joint distributions from birth to the end of the neonatal period, by l/y/s

Table A15. Analytical steps in estimation of YLDs due to preterm birth

	Summary of exposure modelling strategy
**Step A**<br>Model univariate distributions at birth	1. Model mean gestational age, prevalence of gestational age <28 weeks, and prevalence of gestational age <37 weeks, by l/y/s<br>2. Model mean birthweight and prevalence of birthweight <2500 grams, by l/y/s<br>3. Model univariate gestational age and birthweight distributions separately at birth, by l/y/s
**Step B**<br>Model joint distributions at birth	1. Use copulae to model the correlation structure of the joint distribution of gestational age and birthweight, globally<br>2. Model the joint distribution of gestational age and birthweight, by location/year/sex at birth, by applying the globally modelled correlation structure to the location/year/sex-specific univariate models of gestational age and birthweight distributions
**Step C**<br>Model joint distributions from birth to 28 days	1. Model all-cause mortality rates by gestational age and birthweight<br>2. Model gestational age and birthweight distributions of surviving neonates for all l/y/s from birth to end of the neonatal period, using all-cause mortality rates by gestational age and birthweight

Modelling strategy

Step A: Model univariate birthweight and gestational age distributions at birth, by l/y/s

Ensemble distribution models can be constructed with three pieces of information: mean of the distribution, variance of the distribution, and the weights of the distributions being ensemble. To model mean and variance for all l/y/s for birthweight and gestational age, we first used spatiotemporal Gaussian process regression (ST-GPR) models to model prevalence of low birthweight, extremely preterm, and preterm birth for all l/y/s at birth. To model mean birthweight for all l/y/s, OLS linear regression was used to regress mean birthweight on log-transformed low birthweight prevalence. This model was then used to predict mean birthweight for all l/y/s, using the prevalence of low birthweight (<2500 grams) modelled for all l/y/s in ST-GPR. Similarly, to model gestational age mean for all l/y/s, OLS linear regression model was used to regress mean gestational age on log-transformed preterm prevalence. Mean gestational age for all l/y/s was predicted using the preterm birth (<37 weeks) estimated modelled in ST-GPR.

Global ensemble weights for gestational age were derived by using a 3 million sample of all available gestational age and birthweight microdata in Table 8 to select the ensemble weights. The two distribution families that received the highest weights were the Weibull (43%) and log-logistic (21%) distributions. Global ensemble weights for birthweight were derived using a 3 million sample of all available microdata in Table 8, in addition to birthweight microdata available primarily through the DHS and MICS surveys. The four distribution families that received the highest weights were the mirror gamma (31%), log-logistic (19%), normal (10%), and mirror Gumbel (10%) distributions.

For each l/y/s, given the mean and ensemble weights, the variance was optimised to minimise error on the prevalence of preterm birth (<37 weeks) for the gestational age distribution and prevalence of low birthweight (<2500 grams) for the birthweight distribution.

Step B: Model joint birthweight and gestational age distributions at birth, by l/y/s

In order to model the joint distribution of gestational age and birthweight from separate distributions, information was needed about the correlation between the two distributions. Distributions of gestational age and birthweight are not independent; the Spearman correlation for each country where 174 joint microdata were available (Table 8), pooling across all years of data available, ranged from 0.25 to 0.49. The overall Spearman correlation was 0.38, pooling across all countries in the dataset.

Joint distributions between the birthweight and gestational age marginal distributions were modelled with copulae. The Copula and VineCopula packages in R were used to select the optimal copula family and copula parameters to model the joint distribution, using joint microdata from the country-years in Table 8. The copula family selected from the microdata was "Survival BB8", with theta parameter set to 1.75 and delta parameter set to 1.

The joint distribution of birthweight and gestational age per location-year-sex was modelled using the global copula family and parameters selected and the location-year-sex gestational age and birthweight distributions. The joint distribution was simulated 100 times to capture uncertainty. Each simulation consisted of 10,000 simulated joint birthweight and gestational age datapoints. Each joint distribution was divided into 500 g by 2-week bins to match the categorical bins of the relative risk surface. Birth prevalence was then calculated for each 500 g by 2-week bin.

Step C: Model joint distributions from birth to the end of the neonatal period, by l/y/s

Early neonatal prevalence and late neonatal prevalence were estimated using life table approaches for each 500 g and 2-week bin. Using the all-cause early neonatal mortality rate for each location-year-sex, births per location-year-sex-bin, and the relative risks for each location-year-sex-bin in the early neonatal period, the all-cause early neonatal mortality rate was calculated for each location-year-sex-bin. The early neonatal mortality rate per bin was used to calculate the number of survivors at seven days and prevalence in the early neonatal period. Using the same process, the all-cause late neonatal mortality rate for each location-year-sex was paired with the number of survivors at seven days and late neonatal relative risks per bin to calculate late neonatal prevalence and survivors at 28 days.

Relative risks & TMREL

Modelling strategy

For each location, data were pooled across years, and the risk of all-cause mortality at the early neonatal period and late neonatal period at joint birthweight and gestational age combinations was calculated. In all datasets except for the USA, sex-specific data were combined to maximise sample size. The USA analyses were sex-specific. To calculate relative risk at each 500-gram and two-week combination, logistic regression was first used to calculate mortality odds for each joint two-week gestational age and 500-gram birthweight category. Mortality odds were smoothed with Gaussian process regression, with the independent distributions of mortality odds by birthweight and mortality odds by gestational age serving as priors in the regression.

A pooled country analysis of mortality risk in the early neonatal period and late neonatal period by SGA category in developing countries in Asia and sub-Saharan Africa were also converted into 500-gram and two-week bin mortality odds surfaces. The relative risk surfaces produced from microdata and the Asia and Africa surfaces produced from the pooled country analysis were meta-analysed, resulting in a meta-analysed mortality odds surface for each location. The meta-analysed mortality odds surface for each location was smoothed using Gaussian process regression and then converted into mortality risk. To calculate mortality relative risks, the risk of each joint two-week gestational age and 500-gram birthweight category were divided by the risk of mortality in the joint gestational age and birthweight category with the lowest mortality risk.

For each of the country-derived relative risk surfaces, the 500-gram and two-week gestational age joint bin with the lowest risk was identified. This bin differed within each country dataset. To identify the universal 500-gram and two-week gestational age category that would serve as the universal TMREL for our analysis, we chose the bins that was identified to be the TMREL in each country dataset to contribute to the universal TMREL. Therefore, the joint categories that served as our universal TMREL for the LBWSG risk factor were “38–40 weeks of gestation and 3500–4000 grams”, “38–40 weeks of gestation and 4000–4500 grams”, and “40–42 weeks of gestation and 4000–4500 grams”. As the joint TMREL, all three categories were assigned to a relative risk equal to 1.

The total population attributable fraction (PAF) for the low birthweight and short gestation joint risk factor was calculated by summing the PAF calculated from each 500 g x two-week category, with the lowest risk category among all the 500 g x two-week categories serving as the TMREL. The equation for calculating PAF for each 500 g x two-week category is:

$$PAF_{joasgt} = \frac{\sum_{x=1}^{u} RR_{joast}(x)P_{jasgt}(x) - RR_{joasg}(TMRE_{jas})}{\sum_{x=1}^{u} RR_{joas}(x)P_{jasgt}(x)}$$

To calculate the PAFs for the univariate risks (“short gestation for birthweight” and “low birthweight for gestation”), relative risks are first weighted by global exposure in 2019, summed across one of the dimensions (gestational age or birthweight), and then rescaled by the maximum RR in the TMREL block (38–42 weeks of gestation and 3500–4500 grams). Any RR less than 1 was set to 1. Exposure was also summed across the same dimension, and the univariate PAF equalled the sum of the product of the weighted RRs and exposures.

Exclusive breastfeeding

The following section is copied from the appendix of GBD 2021 risk factors publication.[1]

Flowchart

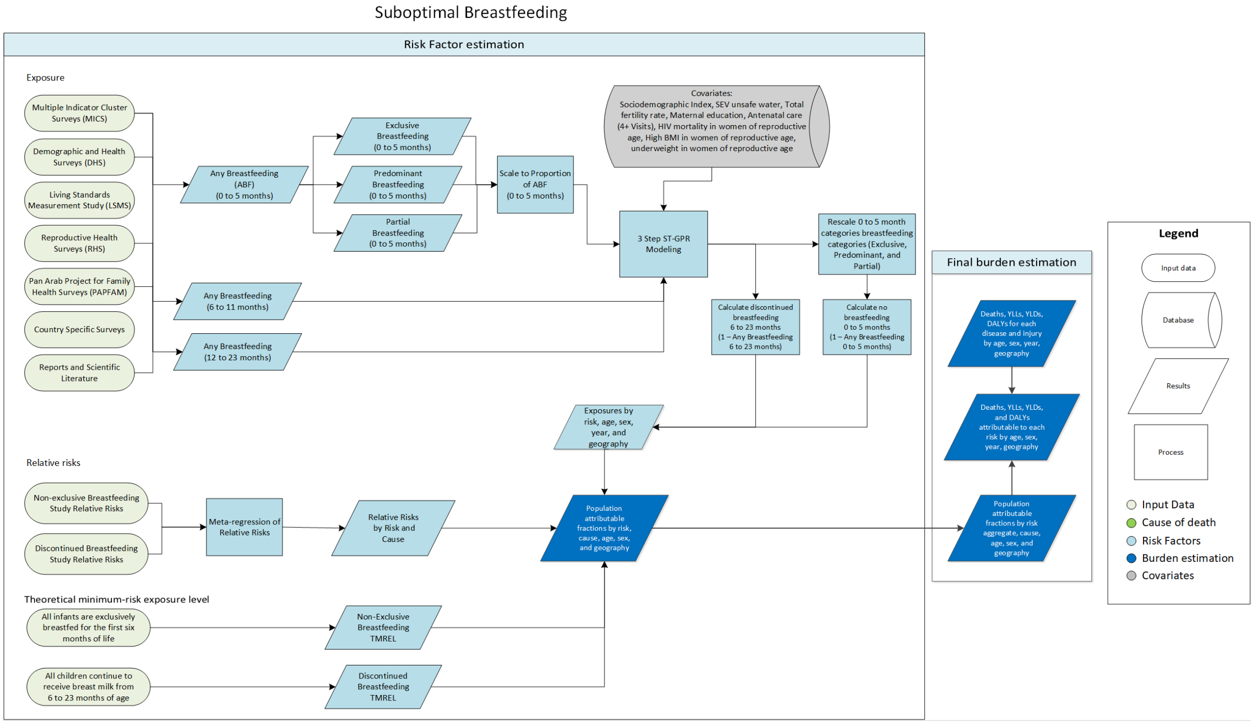

Exposure

Using the processed microdata and tabulated data from reports, we generated a complete time series from 1980 to 2021 for 1) any breastfeeding 0–5 months, 2) ratio of exclusive breastfeeding to any breastfeeding 0–5 months, 3) ratio of predominant breastfeeding to any breastfeeding 0–5 months, and 4) ratio of partial breastfeeding to any breastfeeding 0–5 months using a three-step spatiotemporal Gaussian process regression.

The first step of the ST-GPR process is an ensemble linear mixed-effects regression of our data on a set of potentially predictive covariates taken from the GBD study covariates database. We tested every

combination of these covariates in individual, sex-specific mixed-effects linear regressions with nested random effects at the super-region, region, and location levels. We then evaluated and ranked each of these sub-models by their out-of-sample root-mean-squared error (RMSE). Finally, to produce initial estimates for every location, year, age, and sex in the analysis, we averaged the 50 top-performing models where the estimated coefficients were 1) statistically significant at $p < 0.05$ and 2) in the expected direction. We tested the following covariates in the ensemble prior: Socio-demographic Index, SEV unsafe water, total fertility rate, maternal education, antenatal care (4+ visits), HIV mortality in women of reproductive age, high BMI in women of reproductive age, and underweight in women of reproductive age.

The second, spatiotemporal smoothing step of ST-GPR calculates the residual between our stage 1 regression estimate and each of our observed datapoints and then smooths this residual, drawing strength over space, age, and time and producing a revised stage 2 estimate of birth prevalence for every location, year, and sex. The third step of ST-GPR is a Gaussian process regression, using the stage 2 estimates as a prior and the observed datapoints and their variance to 1) further smooth the residual between the stage 2 predictions and observed data and produce a final mean estimate for each location, year, and sex and 2) estimate uncertainty around this mean estimate, quantified by taking 1000 draws from the posterior Gaussian process.

To generate exposure categories for non-exclusive breastfeeding, we converted the modelled ratios of exclusive, predominant, and partial breastfeeding to the total category prevalence by multiplying each ratio by the estimates of any breastfeeding among infants aged 0–5 months. This ensured that these categories sum correctly to the "any breastfeeding 0–5 months" envelope. We calculated the proportion of infants receiving no breastmilk 0–5 months of age by subtracting the estimates of current breastfeeding from 1.

TMREL

For non-exclusive breastfeeding, those children that received no source of nourishment other than breastmilk ("exclusively breastfed") were considered to be at the lowest risk of any of the disease outcomes.

Relative risk

We estimated relative risks for non-exclusive breastfeeding in a meta-analysis using the "metareg" package in Stata. For the 0–5-month age group, we included diarrhoea and lower respiratory infection as outcomes. We did not estimate separate relative risks for morbidity and mortality. The estimated relative risks are detailed in **Table A16**.

Table A16. Non-exclusive breastfeeding relative risk estimates

Exposure category (0–5 months)	Diarrhoea		Lower respiratory infection	
	Mortality	Morbidity	Mortality	Morbidity
Exclusive breastfeeding	1.00	1.00	1.00	1.00
Predominant breastfeeding	2.35 (1.67–3.23)	2.35 (1.67–3.23)	1.37 (1.06–1.80)	1.37 (1.06–1.80)
Partial breastfeeding	2.63 (1.94–3.48)	2.63 (1.94–3.48)	1.48 (1.21–1.79)	1.48 (1.21–1.79)
No breastfeeding	3.60	3.60	1.74	1.74

	(2.72–4.70)	(2.72–4.70)	(1.49–2.03)	(1.49–2.03)

We used the standard GBD population attributable fraction (PAF) equation to calculate PAFs for non-exclusive breastfeeding and discontinued breastfeeding and each of their paired outcomes using exposure estimates, the theoretical minimum-risk exposure level, and relative risks.

Stunting and wasting

The following exposure methods section is adapted from the appendix of a previous publication.[1]

Exposure

A four-step modelling strategy was employed and applied in parallel to stunting, wasting, and underweight. These four steps are described in detail below.

Ensemble weight fitting

All microdata sources were included in the process of fitting ensemble weights to parameterise characteristic HAZ, WHZ, and WAZ curve shapes. Ten distributions were fit simultaneously to microdata sources: normal, log-normal, log-logistic, exponential, gamma, mirrored gamma, inverse gamma, Gumbel, mirrored Gumbel, and Weibull. All component distributions were parameterised using "methods of moments," meaning that each could be described as a function of the mean and variance of the CGF Z-score distribution. Ensemble weights were assigned to each distribution, and the resulting ensemble distribution was the weighted sum of these individual distributions. Previous iterations of GBD have optimised ensemble weight sets to minimise predictive error across the entire distribution of CGF Z-scores. However, GBD 2021 methodology includes advancements that allow for ensemble weight sets to specifically minimise predictive error at the most relevant portions of the curves, the tails of the distribution associated with disease, namely Z-scores of –3SD, –2SD, and –1SD. The optimisation process weighted the fit evenly at these three portions of the curve. The predictive error in these three portions of the curve was minimised across all input microdata sources simultaneously, with each input source weighted evenly. One hundred sets of initial weights were entered into an optimisation algorithm that aimed to reduce this targeted predictive error across all input microdata sources.[13]

ST-GPR (spatiotemporal Gaussian process regression)

All microdata were collapsed to calculate mean Z-score, overall (CGF < –2SD) prevalence, and severe (CGF < –3SD) prevalence which was added to all tabulation data, including the WHO Global Database on Child Growth and Malnutrition, to be used in ST-GPR (spatiotemporal Gaussian process regression). ST-GPR is a common modelling framework used across GBD that leverages strength of evidence across space and time to produce estimates for each age group, sex, year, and location.[1]

The first step of the ST-GPR process is an ensemble mixed-effects linear regression. For each indicator, we identified potentially predictive covariates from the GBD covariates database and tested every combination of those covariates in a mixed-effects linear regression. In this analysis, indicator data points were regressed on the predictive covariates with nested random effects at the super-region, region, and location levels (See **Figure S12** for region and super-region designations). Models where estimated coefficients were not statistically significant ($p < 0.05$) or were not in the a priori expected direction were dropped. The remaining models were then ranked by their out-of-sample root-mean-square error (RMSE), with a weighted ensemble of the top-performing models ultimately used as the first-stage prior in ST-GPR. Details about the covariates that were tested in CGF ensemble regression have been published previously.[1] Selected covariates were consistent across all ST-GPR models for stunting, wasting, and underweight.

The second step of ST-GPR is a regression that incorporates evidence from neighbouring locations, proximate years, and similar age groups to smooth residuals in the estimate from the ensemble mixed-effects linear regression created in step 1. The spatiotemporal smoothing is controlled by three hyper-parameters (ζ = space, λ = time, Ω = age), which were adjusted to maximise out-of-sample predictive validity throughout the entire time series.

The third step of ST-GPR is a Gaussian process regression that further smooths the residuals between observed data and the stage two estimate and estimates uncertainty in the final modelled estimate. The Gaussian process regression incorporates uncertainty of input datapoints as well as difference between the stage two estimate and the ensemble mixed-effects linear regression created in step one to estimate model uncertainty. We obtained 1000 samples from the final Gaussian process distributions for every location, age, sex, and year, from which we calculated 95% uncertainty intervals as the 2.5th and 97.5th percentiles of these sample distributions. In countries that are modelled subnationally, national-level estimates may reflect either sums of estimates from subnational locations (a process called aggregation), or subnational estimates may reflect the proportionally split national-level estimate (a process called raking). This decision is based on availability of subnational data, concordance of subnational and national-level data, and levels of uncertainty in subnational and national-level data. In cases with more high-quality subnational-level data, aggregation is preferred. Raking allows for national-level estimates to have a larger influence on subnational estimates. Raking and aggregating decisions were consistent across stunting, wasting, and underweight.

Variance modelling

The third modelling step is an optimisation process that grounds the distribution at the mean Z-score estimate modelled from ST-GPR and calculates the variance value that minimises the predictive error on ST-GPR estimates of severe (Z-score < –3SD) and overall (Z-score < –2SD) CGF prevalence. The form of this distribution does not change in this optimisation process, as the distribution maintains the characteristic shape determined by the weight optimisation in modelling step 1. In this way, the spread of the characteristic curve shape for stunting, wasting, and underweight is optimised to best align with ST-GPR estimates of key portions of the distribution. Using the "method of moments" equation for each component of the ensemble distribution, a probability density function is calculated for each age, sex, year, and location.

Integration

The probability density functions produced from variance modelling are integrated to determine the prevalence of overall CGF (Z-score < –2SD), mild CGF (–2SD < Z-score < –1SD), moderate CGF (–3SD < Z-score < –2SD), severe CGF (Z-score < –3SD), and extreme CGF (Z-score < –4SD). The categorical exposures to mild, moderate, and severe CGF are used for subsequent risk analysis in GBD.

TMREL

Theoretical minimum risk exposure level for underweight, stunting, and wasting was assigned to be greater than or equal to –1 SD of the WHO 2006 standard weight-for-age, height-for-age, and weight-for-height curves, respectively. This has not changed since GBD 2010.

Relative risk

The final list of outcomes paired with child growth failure risks included mortality and morbidity for lower respiratory infections (LRI), diarrhoea, malaria, measles, and protein-energy malnutrition (PEM). These were derived from a Burden of Proof analysis[14] that incorporated both a pooled analysis of ten

prospective cohort studies by Olofin and colleagues[10] as well as relative risk estimates from Knowledge Integration (KI) studies (**Table A11**). For the KI studies, aggregated relative risks of disease or cause-specific mortality were calculated for 1-unit z-score bins for stunting, wasting, and underweight (e.g., relative risk of diarrhea-attributable death in children 1 to 2 years of age and with a HAZ score between -4 and -3). The burden of proof analysis uses all available relative risks with corresponding uncertainty to create continuous relative risk curves for each outcome/risk pair. These continuous risk curves are then combined with the global exposure curves for HAZ, WAZ, and WHZ, to calculate exposure-weighted relative risks for severe, moderate, and mild stunting, wasting, and underweight with uncertainty. Of historical note, upper respiratory infections and otitis media were included as outcomes in the GBD 2013 risk analysis, based on the "analogy" causal criterion, assuming there is similar pathway as LRI outcome. However, closer review for GBD 2015 did not find sufficient evidence to support their inclusion and they were excluded, a decision that was carried forward into GBD 2016. We also attributed 100% of PEM to childhood wasting and underweight but not stunting. To build on the existing literature base for GBD on risk-outcome pairs, a literature search was conducted for GBD 2017 searching for case-control studies published after January 1, 1985 did not return any sources that were usable.

There is a high degree of correlation between stunting, wasting, and underweight. Failing to account for their covariance and assuming independence would overestimate the total burden significantly and misrepresent the attributable burden of individual CGF indicators. Inability to address these correlations is the main reason that GBD 2010 only included childhood underweight.

In order to account for the high degree of correlation between CGF indicators, GBD uses a constrained optimisation method to adjust the observed univariate RRs that come out of the Burden of Proof analysis. First, we created a joint distribution of stunting, underweight, and wasting from a population of children. Second, we generated 1000 RR draws for each univariate indicator and severity based from the Burden of Proof analysis. Third, we altered these univariate RRs for the four causes (diarrhoea, LRI, measles, and measles) and the two outcomes (mortality and morbidity) based upon interactions among the CGF indicators. An interaction occurs when the effect of one CGF indicator variable (eg, stunting) has a different effect on the outcome depending on the value of another CGF indicator variable (eg, wasting). Interaction terms alter the risk of the outcome among children with more than one indicator of CGF. These interaction terms were extracted from a pooled cohort analysis of all-cause mortality published by McDonald, et al.[15] Lastly, we optimised the adjusted relative risks by minimising the error between the observed RRs (generated from Olofin, et al) and the altered RRs derived from the joint distribution and accounting for the interaction terms while ensuring that no alteration resulted in a previously identified increase in relative risk becoming protective.

For GBD 2021, we made several changes to improve the four main steps of RR adjustment. From GBD 2013 to GBD 2019, a simulated joint distribution of stunting, underweight, and wasting measures was created from the Olofin, et al. meta-analysis. Sources in this meta-analysis were cross-sectional DHS. We created age-specific joint distributions of stunting, underweight, and wasting measures from 15 longitudinal studies (from 26 locations) in the Bill and Melinda Gates Foundation's Knowledge Integration (Ki) database.[16] The RR adjustment method was strengthened by constraining optimisation in two ways. Optimisation was only permitted to alter the RR for an indicator/severity in draws where the observed RR was greater than 1, and constraints were placed on the error that penalise larger alterations to the RR. These changes enabled the estimation and utilisation of age-specific adjusted RRs for GBD 2021 burden estimation. The largest change for GBD 2021 was conducting Burden of Proof

Analyses for each cause/outcome/risk triplet using both data from Olofin et al as well as KI data. These changes result in identifying large differences in the relationship between CGF and mortality versus morbidity as well as identifying some impact of CGF on malaria. The estimated cause-specific relative risks are detailed in **Table A17**.

Table A17. Age-specific adjusted RRs for each risk–outcome pair for child growth failure

**1 to 5 months**		**Incidence**			**Mortality**		
**Cause**		**<-3**	**-3,-2**	**-2,-1**	**<-3**	**-3,-2**	**-2,-1**
**Diarrhea**	HAZ	1.2 (0.8, 1.7)	1.2 (0.8, 1.6)	1.1 (0.9, 1.5)	3.6 (2.1, 4.4)	2.1 (1.6, 2.6)	1.4 (1.2, 1.6)
	WAZ	1.6 (0.9, 2.7)	1.6 (0.9, 2.6)	1.5 (0.9, 2.4)	6.7 (4.4, 9.3)	3.4 (2.2, 4.7)	1.8 (1.3, 2.2)
	WHZ	1.3 (0.8, 1.8)	1.2 (0.9, 1.7)	1.2 (0.9, 1.6)	40.8 (0.8, 224.7)	12.8 (0.8, 51.5)	4.1 (0.9, 10.6)
**LRI**	HAZ	1.3 (0.6, 2.5)	1.1 (0.7, 1.8)	1.1 (0.8, 1.5)	5.1 (2.7, 7.6)	2.9 (1.7, 4.2)	1.8 (1.2, 2.4)
	WAZ	1.6 (0.6, 4.1)	1.3 (0.7, 2.2)	1.2 (0.8, 1.7)	31.1 (1.0, 191.4)	13.9 (1.0, 69.2)	4.9 (1.0, 16.8)
	WHZ	1.3 (0.9, 1.8)	1.1 (1.0, 1.2)	1.0 (1.0, 1.1)	6.5 (4.5, 8.8)	3.6 (2.6, 4.6)	1.8 (1.5, 2.1)
**Malaria**	HAZ	1.0 (1.0, 1.0)	1.0 (1.0, 1.0)	1.0 (1.0, 1.0)	3.0 (0.6, 12.4)	1.1 (0.9, 1.2)	1.0 (1.0, 1.0)
	WAZ	2.3 (0.6, 6.6)	2.3 (0.6, 6.6)	2.3 (0.6, 6.6)	2.7 (0.6, 8.4)	1.8 (0.7, 3.9)	1.5 (0.8, 2.5)
	WHZ	1.0 (1.0, 1.0)	1.0 (1.0, 1.0)	1.0 (1.0, 1.0)	1.0 (1.0, 1.0)	1.0 (1.0, 1.0)	1.0 (1.0, 1.0)
**Measles**	HAZ	1.3 (0.9, 2.3)	1.0 (1.0, 1.0)	1.0 (1.0, 1.0)	4.1 (1.8, 5.7)	2.2 (1.4, 2.9)	1.4 (1.1, 1.5)
	WAZ	1.0 (1.0, 1.0)	1.0 (1.0, 1.0)	1.0 (1.0, 1.0)	5.1 (2.2, 7.8)	2.7 (1.6, 3.5)	1.4 (1.2, 1.6)
	WHZ	1.7 (0.8, 3.3)	1.3 (0.9, 1.8)	1.3 (0.9, 1.8)	6.7 (2.5, 15.4)	2.6 (1.6, 4.2)	1.2 (1.1, 1.4)
**PEM**	HAZ		0% PAF			0% PAF	
	WAZ		100% PAF			100% PAF	
	WHZ		100% PAF			100% PAF	
**6 to 11 months**		**Incidence**			**Mortality**		
**Cause**		**<-3**	**-3,-2**	**-2,-1**	**<-3**	**-3,-2**	**-2,-1**
**Diarrhea**	HAZ	1.2 (0.8, 1.7)	1.2 (0.8, 1.6)	1.1 (0.9, 1.5)	3.1 (2.2, 3.9)	1.9 (1.5, 2.4)	1.3 (1.2, 1.6)
	WAZ	1.6 (0.9, 2.7)	1.6 (0.9, 2.6)	1.5 (0.9, 2.4)	5.9 (3.7, 8.9)	3.1 (2.0, 4.5)	1.7 (1.3, 2.2)
	WHZ	1.3 (0.8, 1.8)	1.2 (0.9, 1.7)	1.2 (0.9, 1.6)	40.7 (0.8, 224.7)	12.7 (0.8, 51.5)	4.1 (0.9, 10.6)
**LRI**	HAZ	1.3 (0.6, 2.5)	1.1 (0.7, 1.8)	1.1 (0.8, 1.5)	4.3 (2.2, 6.8)	2.6 (1.5, 3.9)	1.7 (1.2, 2.3)
	WAZ	1.6 (0.6, 4.1)	1.3 (0.7, 2.2)	1.2 (0.8, 1.7)	31.0 (1.0, 191.4)	13.8 (1.0, 69.2)	4.9 (1.0, 16.8)
	WHZ	1.3 (0.9, 1.8)	1.1 (1.0, 1.2)	1.0 (1.0, 1.1)	6.2 (4.3, 8.4)	3.5 (2.5, 4.5)	1.8 (1.5, 2.1)
**Malaria**	HAZ	1.0 (1.0, 1.0)	1.0 (1.0, 1.0)	1.0 (1.0, 1.0)	3.0 (0.6, 12.4)	1.1 (0.9, 1.2)	1.0 (1.0, 1.0)
	WAZ	2.3 (0.6, 6.6)	2.3 (0.6, 6.6)	2.3 (0.6, 6.6)	2.7 (0.6, 8.4)	1.8 (0.7, 3.9)	1.5 (0.8, 2.5)

	WHZ	1.0 (1.0, 1.0)	1.0 (1.0, 1.0)	1.0 (1.0, 1.0)	1.0 (1.0, 1.0)	1.0 (1.0, 1.0)	1.0 (1.0, 1.0)
**Measles**	HAZ	1.3 (0.9, 2.3)	1.0 (1.0, 1.0)	1.0 (1.0, 1.0)	3.6 (1.8, 4.9)	2.1 (1.4, 2.6)	1.3 (1.1, 1.5)
	WAZ	1.0 (1.0, 1.0)	1.0 (1.0, 1.0)	1.0 (1.0, 1.0)	5.2 (2.2, 8.1)	2.7 (1.6, 3.6)	1.4 (1.2, 1.6)
	WHZ	1.7 (0.8, 3.3)	1.3 (0.9, 1.8)	1.3 (0.9, 1.8)	6.7 (2.5, 15.4)	2.6 (1.6, 4.2)	1.2 (1.1, 1.4)
**PEM**	HAZ		0% PAF			0% PAF	
	WAZ		100% PAF			100% PAF	
	WHZ		100% PAF			100% PAF	

**12 to 23 months**		**Incidence**			**Mortality**		
**Cause**		**<-3**	**-3,-2**	**-2,-1**	**<-3**	**-3,-2**	**-2,-1**
**Diarrhea**	HAZ	1.2 (0.8, 1.7)	1.2 (0.8, 1.6)	1.1 (0.9, 1.5)	2.7 (2.2, 3.4)	1.7 (1.4, 2.2)	1.3 (1.1, 1.5)
	WAZ	1.6 (0.9, 2.7)	1.6 (0.9, 2.6)	1.5 (0.9, 2.4)	5.4 (3.7, 8.4)	2.9 (2.0, 4.4)	1.6 (1.3, 2.1)
	WHZ	1.3 (0.8, 1.8)	1.2 (0.9, 1.7)	1.2 (0.9, 1.6)	40.7 (0.8, 225.5)	12.7 (0.8, 51.7)	4.0 (0.9, 10.6)
**LRI**	HAZ	1.3 (0.6, 2.5)	1.1 (0.7, 1.8)	1.1 (0.8, 1.5)	4.1 (2.3, 5.9)	2.5 (1.5, 3.5)	1.6 (1.2, 2.2)
	WAZ	1.6 (0.6, 4.1)	1.3 (0.7, 2.2)	1.2 (0.8, 1.7)	29.3 (1.0, 186.4)	13.0 (1.0, 67.8)	4.6 (1.0, 16.5)
	WHZ	1.3 (0.9, 1.8)	1.1 (1.0, 1.2)	1.0 (1.0, 1.1)	5.4 (3.5, 7.6)	3.1 (2.1, 4.2)	1.7 (1.3, 2.0)
**Malaria**	HAZ	1.0 (1.0, 1.0)	1.0 (1.0, 1.0)	1.0 (1.0, 1.0)	3.0 (0.6, 12.4)	1.1 (0.9, 1.2)	1.0 (1.0, 1.0)
	WAZ	2.3 (0.6, 6.6)	2.3 (0.6, 6.6)	2.3 (0.6, 6.6)	2.7 (0.6, 8.4)	1.8 (0.7, 3.9)	1.5 (0.8, 2.5)
	WHZ	1.0 (1.0, 1.0)	1.0 (1.0, 1.0)	1.0 (1.0, 1.0)	1.0 (1.0, 1.0)	1.0 (1.0, 1.0)	1.0 (1.0, 1.0)
**Measles**	HAZ	1.3 (0.9, 2.3)	1.0 (1.0, 1.0)	1.0 (1.0, 1.0)	3.5 (1.8, 4.4)	2.0 (1.4, 2.5)	1.3 (1.1, 1.4)
	WAZ	1.0 (1.0, 1.0)	1.0 (1.0, 1.0)	1.0 (1.0, 1.0)	4.6 (2.2, 6.1)	2.5 (1.6, 3.0)	1.4 (1.2, 1.5)
	WHZ	1.7 (0.8, 3.3)	1.3 (0.9, 1.8)	1.3 (0.9, 1.8)	6.7 (2.5, 15.4)	2.6 (1.6, 4.2)	1.2 (1.1, 1.4)
**PEM**	HAZ		0% PAF			0% PAF	
	WAZ		100% PAF			100% PAF	
	WHZ		100% PAF			100% PAF	
**2 to 4 years**		**Incidence**			**Mortality**		
**Cause**		**<-3**	**-3,-2**	**-2,-1**	**<-3**	**-3,-2**	**-2,-1**
**Diarrhea**	HAZ	1.2 (0.8, 1.7)	1.2 (0.8, 1.6)	1.1 (0.9, 1.5)	2.8 (2.1, 3.4)	1.8 (1.5, 2.2)	1.3 (1.2, 1.5)
	WAZ	1.6 (0.9, 2.7)	1.6 (0.9, 2.6)	1.5 (0.9, 2.4)	5.6 (4.0, 8.3)	2.9 (2.1, 4.3)	1.6 (1.3, 2.1)
	WHZ	1.3 (0.8, 1.8)	1.2 (0.9, 1.7)	1.2 (0.9, 1.6)	41.3 (0.8, 229.9)	12.8 (0.8, 52.4)	4.0 (0.9, 10.7)
**LRI**	HAZ	1.3 (0.6, 2.5)	1.1 (0.7, 1.8)	1.1 (0.8, 1.5)	9.5 (4.7, 21.2)	4.7 (3.0, 8.6)	2.5 (2.0, 3.8)

	WAZ	1.6 (0.6, 4.1)	1.3 (0.7, 2.2)	1.2 (0.8, 1.7)	14.5 (1.0, 60.4)	7.1 (1.0, 21.7)	3.1 (1.0, 5.6)
	WHZ	1.3 (0.9, 1.8)	1.1 (1.0, 1.2)	1.0 (1.0, 1.1)	5.1 (2.3, 7.9)	3.0 (1.5, 4.3)	1.6 (1.2, 2.1)
**Malaria**	HAZ	1.0 (1.0, 1.0)	1.0 (1.0, 1.0)	1.0 (1.0, 1.0)	3.0 (0.6, 12.4)	1.1 (0.9, 1.2)	1.0 (1.0, 1.0)
	WAZ	2.3 (0.6, 6.6)	2.3 (0.6, 6.6)	2.3 (0.6, 6.6)	2.7 (0.6, 8.4)	1.8 (0.7, 3.9)	1.5 (0.8, 2.5)
	WHZ	1.0 (1.0, 1.0)	1.0 (1.0, 1.0)	1.0 (1.0, 1.0)	1.0 (1.0, 1.0)	1.0 (1.0, 1.0)	1.0 (1.0, 1.0)
**Measles**	HAZ	1.3 (0.9, 2.3)	1.0 (1.0, 1.0)	1.0 (1.0, 1.0)	3.5 (1.8, 4.4)	2.0 (1.4, 2.5)	1.3 (1.1, 1.4)
	WAZ	1.0 (1.0, 1.0)	1.0 (1.0, 1.0)	1.0 (1.0, 1.0)	4.6 (2.2, 6.1)	2.5 (1.6, 3.0)	1.4 (1.2, 1.5)
	WHZ	1.7 (0.8, 3.3)	1.3 (0.9, 1.8)	1.3 (0.9, 1.8)	6.7 (2.5, 15.4)	2.6 (1.6, 4.2)	1.2 (1.1, 1.4)
**PEM**	HAZ		0% PAF			0% PAF	
	WAZ		100% PAF			100% PAF	
	WHZ		100% PAF			100% PAF	

Overweight

Exposure

After adjusting for self-report bias and splitting aggregated data into five-year age-sex groups, we used spatiotemporal Gaussian process regression (ST-GPR) to estimate the prevalence of overweight and obesity. This modelling approach has been described in detail elsewhere. The linear model, which when added to the smoothed residuals forms the mean prior for GPR is as follows:

$$\text{logit}(\text{overweight})_{c,a,t} = \beta_0 + \beta_1\text{energy}_{c,t} + \beta_2\text{SDI}_{c,t} + \beta_3\text{vehicles}_{c,t} + \beta_4\text{agriculture}_{c,t} + \sum_{k=5}^{21}\beta_k I_{A[a]} + \alpha_s + \alpha_r + \alpha_c$$

$$\text{logit}(\text{obesity/overweight})_{c,a,t} = \beta_0 + \beta_1\text{energy}_{c,t} + \beta_2\text{SDI}_{c,t} + \beta_3\text{vehicles}_{c,t} + \sum_{k=4}^{21}\beta_k I_{A[a]} + \alpha_s + \alpha_r + \alpha_c$$

where energy is ten-year lag-distributed energy consumption per capita, SDI is a composite index of development including lag-distributed income per capita, education, and fertility, vehicles is the number of two- or four-wheeled vehicles per capita, and agriculture is the proportion of the population working in agriculture. $I_{A[a]}$ is a dummy variable indicating specific age group A that the prevalence point captures, and α_s, α_r, and α_c are super-region, region, and country random intercepts, respectively. Random effects were used in model fitting but were not used in prediction.

We tested all combinations of the following covariates to see which performed best in terms of in-sample AIC for the overweight linear model and the obesity as a proportion of overweight linear model: ten-year lag-distributed energy per capita, proportion of the population living in urban areas, SDI, lag-distributed income per capita, educational attainment (years) per capita, proportion of the population working in agriculture, grams of sugar adjusted for energy per capita, grams of sugar not adjusted for energy per capita, and the number of two- or four-wheeled vehicles per capita. We selected these candidate covariates based on theory as well as reviewing covariates used in other publications. The

final linear model was selected based on 1) if the direction of covariates matched what is expected from theory, 2) all the included covariates were significant, and 3) minimising in-sample AIC. The covariate selection process was performed using the dredge package in R.

TMREL
For children (ages 2–19), the TMREL is "normal weight," that is, not overweight or obese, based on IOTF cutoffs.

Relative risk
Risk-outcome pairs were defined based on strength of available evidence supporting a causal effect. We performed a systematic review of published meta-analyses, pooled analyses, and systematic reviews available through PubMed using the following search string: ("Body Mass Index"[Mesh] OR "Overweight"[Mesh] OR "Obesity"[Mesh]) AND (Meta-Analysis[ptyp] OR "systematic review"[tiab] OR "pooled analysis"[tiab]). Additional details have been published previously.[1]

For childhood outcomes (ages 2–19), we computed categorical relative risks for overweight and obesity using a random effects meta-analysis.

Anaemia (women of reproductive age)
The following section is from the appendix of GBD 2021 anaemia impairment capstone.[12]

Modelling strategy
Estimation of overall anaemia prevalence occurred in four steps: 1) ST-GPR models of mean haemoglobin and prevalence of total, moderate+severe, and severe anaemia, 2) Calculation of ensemble weights, 3) Generation of ensemble distributions, and 4) Calculation of anaemia prevalence based on the ensemble distributions.

ST-GPR models of mean haemoglobin and anaemia prevalence
We modelled 1) mean haemoglobin, 2) total anaemia prevalence, 3) moderate+severe anaemia prevalence, and 4) severe anaemia prevalence using spatiotemporal Gaussian process regression (ST-GPR), a three-step modelling process for generating estimates for every location, year, age, and sex in the GBD study. The first step of the ST-GPR process is an ensemble linear mixed-effects regression of our data on a set of potentially predictive covariates taken from the GBD study covariates database. We tested every combination of these covariates in individual mixed-effects linear regressions with nested random effects at the super-region, region, and location levels. We then evaluated and ranked each of these sub-models by their out-of-sample root-mean-squared error (RMSE). Finally, to produce initial estimates for every location, year, age, and sex in the analysis, we averaged the 50 top-performing models where the estimated coefficients were 1) statistically significant at $p < 0.05$ and 2) in the expected direction. The following covariates were tested in this ensemble regression: Age-specific fertility rate, HIV prevalence, SEV for child underweight, SEV for child wasting, malaria incidence, haemoglobin C (sickle type C) trait (all ages), haemoglobin S (sickle type S) trait (all ages), Socio-demographic Index, SEV for impaired kidney function, Healthcare Access and Quality Index, GDP per capita, modern contraception prevalence, and 50th percentile of haemoglobin (pooled across all microdata sources).

The second, spatiotemporal smoothing step of ST-GPR calculates the residual between our stage 1 regression estimate and each of our observed datapoints and then smooths this residual, drawing

strength over space, age, and time and producing a revised stage 2 estimate for every location, year, age, and sex. The third step of ST-GPR is a Gaussian process regression, using the stage 2 estimates as a prior and the observed datapoints and their variance to 1) further smooth the residual between the stage 2 predictions and observed data and produce a final mean estimate for each location, year, age, and sex and 2) estimate uncertainty around this mean estimate, quantified by taking 1,000 draws from the posterior Gaussian process.

Ensemble distribution modelling

We modelled the full distribution of haemoglobin for each population (location/age/year/sex), from which we applied the WHO thresholds to calculate prevalence of anaemia by severity. We combined multiple two-parameter distributions to create an ensemble distribution as described below.

Generation of ensemble weights

First, we created a training and testing set of individual-level haemoglobin measurements. The training set consisted of 90 DHS surveys, providing 290 group-specific samples of microdata from children <5, males 15–45, pregnant females 15–45, and non-pregnant females 15–45 (not all groups were sampled in each DHS). A set of two-parameter distributions (gamma, mirror gamma, Weibull, mirror lognormal, and mirror Gumbel) were fit to the sample's haemoglobin mean and variance. These distributions were combined using weights optimised by a loss function of severity-specific prediction error weighted by the ratio of the severity's disability weight (DW) to mild anaemia DW. Weights were constrained to be positive and sum to 1, so that the resultant ensemble distribution is a proper probability density function. All permutations of the five distributions were tested (ie, we optimised weights for both a mix of all five distributions as well as a gamma-Weibull two-way combination).

The loss function is

$$\sum_{i=1}^{n_i}\sum_{j=1}^{n_j}\sum_{k=1}^{n_k} r_j|p_{ijk} - \hat{p}_{ijk}|$$

Where

$$\hat{p}_{ijk} = \sum_{z=1}^{n_z} w_z \int_{t1_{jk}}^{t2_{jk}} PDF_{ijz}$$

n_i is a list of surveys (in either the training or testing set)

n_j is the list of groups: children <5, males 15–45, pregnant females 15–45, non-pregnant females 15–45, males >45, and females >45

n_k is the list of severities (mild, moderate, severe)

n_z is the list of distributions (gamma, mirrored gamma, Weibull, mirrored lognormal, and mirrored Gumbel)

r is the ratio of the severity j disability weight to that of mild anaemia

r_k = 13 for moderate and r_k = 40 for severe

PDF is a probability density function fit to the sample mean and variance

t1 and t2 are the lower and upper bounds to the WHO anaemia definition for the group

w is the set of distribution weights (each constrained to be positive) such that

$\sum_{z=1}^{n_z} w_z = 1$ and all $w_z > 0$

Therefore $\sum_{z=1}^{n_z} w_z * PDF_z$ describes the ensemble probability density function that can be integrated to calculate prevalence for any severity.

The testing set consisted of nine NHANES and nine DHS surveys not included in the training data. Inclusion of NHANES as half the testing set ensured out of sample predictive validity by challenging the global weights, as it provided the ensemble distribution with high-income data (DHS is from LMIC countries) and data from adults >45 (DHS did not take blood tests from older populations). We selected the combination of distributions (including all individual component distributions) that minimised the loss function. For GBD 2021 this resulted in an ensemble distribution that was 40% gamma and 60% mirrored Gumbel.

Haemoglobin variance optimisation

In order to generate an ensemble distribution of haemoglobin concentration, we first need an estimate of the variance of the haemoglobin distribution. To generate an estimate of distribution variance, we used our ST-GPR estimates of mean haemoglobin and prevalence of total, moderate+severe, and severe anaemia prevalence in a variance-optimisation algorithm. For every location, year, age, and sex we anchored the distributions at the estimated mean [Hb] value and found the variance value that minimised the error between our estimates of severe, moderate+severe, and total anaemia and the corresponding values implied by a given mean and variance [Hb] combination. We weighted the errors in the variance optimisation algorithm based on the severity-specific disability weights, such that the severe anaemia error was weighted the most and total anaemia was weighted the least.

Fitting ensemble distributions with method of moments

Using our global model weights and our estimates of mean haemoglobin and standard deviation of haemoglobin, we modelled the full distribution of haemoglobin for each location, year, age, and sex by fitting each component distribution on the modelled mean and standard deviation and weighting each individual distribution to create the ensemble distribution.

Because anaemia thresholds depend on pregnancy, we separately modelled the distribution of haemoglobin among pregnant and non-pregnant women. Our modelling process directly provided estimates of mean haemoglobin and variance of haemoglobin concentration among non-pregnant women; to estimate the mean haemoglobin among pregnant women, we shifted the non-pregnant mean haemoglobin estimate for each location, year, age, and sex by the crosswalk adjustment factor calculated as described in the data processing section. We assumed that the distribution variance was the same among pregnant and non-pregnant women.

We used these estimates of mean haemoglobin and SD of haemoglobin concentration by pregnancy status to produce separate weighted ensemble distributions. To combine these distributions to represent the entire population of females in a given location, year, and age, we estimated the proportion of pregnant women for each location, year, and age using the formula:

$$pregnancy = (ASFR + SB) * 46/52$$

Where *ASFR* is the location- and age-specific fertility rate and *SB* is the location-specific stillbirth rate. We then weighted each of the haemoglobin distributions among pregnant and non-pregnant women accordingly to produce a final general population distribution.

Finding the area under the curve to calculate anaemia prevalence

Using the WHO anaemia thresholds shown in **Table A2**, we calculated the prevalence of mild, moderate, and severe anaemia for each location, year, age group, and sex as the area under the [Hb] concentration curve between the thresholds for each severity.

Central computation for deaths, YLLs, YLDs, and DALYs

YLD calculation

The number of years lived with disability (YLD) was calculated as the number of people living with the disease multiplied by the disability weight (DW) that was estimated to represent the level of health loss attributable to the disease between zero and one, where zero is no health loss and one is death.

Comorbidity simulation (COMO)

To account for co-occurrence of different diseases, we adjusted YLDs in a micro-simulation process termed "COMO," using the prevalence of the disease sequelae estimated via DisMod-MR 2.1 and their associated disability weights. A simulated dataset of 40,000 individuals for every location-age-sex-year estimated in the GBD was run through a four-step process:

1) Each individual was estimated to have zero to multiple disease sequelae.

2) Each individual's assigned disease sequelae disability weights (DW) were estimated (one minus the multiplicative sum of one minus each DW present).

3) The DW attributable to each sequela for the individual was calculated (DW of the disease sequela divided by the sum of the disease sequelae across all individuals, multiplied by the individual's total attributed DW).

4) YLDs per capita for every location-age-sex-year were calculated by summing the attributable DWs for a disease across individuals, and then multiplying this rate by the location-age-sex-year population.

Uncertainty was then propagated by repeating this process 1000 times for each location-age-sex-year and taking the 25th and 97.5th percentile from these 1000 iterations.

CoDCorrect

To ensure internal consistency across mortality estimation of all GBD causes, that is, to ensure that summed cause-specific modelled estimates did not exceed all-cause mortality estimates, a core algorithm was used in the process known as CoDCorrect. Level 1 causes were rescaled to match all-cause mortality estimates; Level 2 causes were rescaled to match the rescaled Level 1 causes, and so on.

$$CD_{lyasjd} = D_{lyasjd}\left(\frac{PD_{lyasjd}}{\sum_{j=1}^{j=k} D_{lyasjd}}\right)$$

Where:

CD_{lyasjd} is the corrected number of deaths for a location *l*, year *y*, age *a*, sex *s*, cause *j*, and draw *d*

PD_{lyasjd} is the parent CoD for a location *l*, year *y*, age *a*, sex *s*, cause *j*, and draw *d*

D_{lyasjd} is the uncorrected number of deaths estimated from a cause-specific model for a location *l*, year *y*, age *a*, sex *s*, cause *j*, and draw *d*

Years of life lost calculation

Years of life lost (YLLs) were calculated as a measure of premature death in reference to a theoretical minimum risk life table. The life table was compiled in 2016 by taking the lowest observed age-specific mortality rates by location and sex across all GBD locations with populations greater than 5 million. YLLs were calculated by multiplying deaths produced from final CoDCorrect estimates by the life expectancy at the age of death, thereby attributing a heavier weight to death at younger ages.

$$YLL = \sum_{c=1,a=0,s=1}^{\infty} d_{cas}e_a$$

DALYnator

Disability-adjusted life-years (DALYs) were estimated by summing YLLs and YLDs for every location-age-sex-year for which YLD estimates exist. Uncertainty was calculated by simulating 1000 draws each of YLLs and YLDs, summing these draws, and then taking the 25th and 97.5th percentiles of the distribution.

DALYs: the sum of YLLs and YLDs

One DALY represents the loss of the equivalent of one year of full health.[1] DALYs for a disease or health condition are the sum of the years of life lost to due to premature mortality (YLLs) and the years lived with a disability (YLDs) due to prevalent cases of the disease or health condition in a population.[17]

Population attributable fraction

Population attributable fraction (PAF) represents the relative reduction in the relevant outcome quantity if there was no exposure to a given indicator. Attributable deaths, YLLs, YLDs, and DALYs were computed for all GNT indicators (except for anaemia) by multiplying PAFs by the relevant outcome quantity for each age-sex-location-year. PAFs and attributable burden for combinations of risk factors were estimated taking into account mediation of different risk factors through other risk factors. Uncertainty in each step of the analysis was propagated into the final estimates of attributable burden.

SEVs

Summary exposure value (SEV) is the RR-weighted prevalence of exposure, a univariate measure of risk-weighted exposure, taking the value zero when no excess risk for a population exists and the value one when the population is at the highest level of risk.[1] We report SEVs on a scale from 0% to 100% on which a decline in SEV indicates reduced exposure to a given risk factor and an increase in SEV indicates increased exposure.

We first calculate risk, r, and cause, c, for specific SEVs by using the following equation,

$$SEV_{rc} = \frac{\frac{PAF_{rc}}{1 - PAF_{rc}}}{RR_{max} - 1}$$

for each most-detailed age, sex, location, year, and outcome. PAF is the YLL (except for any outcomes which are YLD only and thus use the YLD) PAF. RR_{max} for categorical risks is the RR at the highest category of exposure. For continuous risks, this is

$$RR_{max} = RR^{\frac{TMREL - 5^{th} \exp osure}{RR_{scalar}}} \text{ if protective, or}$$

$$= RR^{\frac{95^{th} \exp o\, sure - TMREL}{RR_{scalar}}}$$

If the PAF is negative, which signifies a protective effect for that outcome, the PAF is set to 0 and the SEV is then also 0 because the SEV is univariate and constrained to be a value between 0 and 1.

In most cases, risk – cause PAFs of 1 were not included in SEV calculations as the SEV function is undefined when the PAF value is 1. However, an alternate definition of SEV was used for iron deficiency. For iron deficiency, SEVs were set to the prevalence of moderate or severe anaemia.

Once we obtained a set of risk-cause-specific SEVs at the most-detailed risk, cause, age, sex, and location for all years, we averaged across causes to produce the final risk-specific SEV_r,

$$SEV_r = \frac{1}{N(c)} \sum_c SEV_{rc}$$

where $N(c)$is the total number of outcomes for a risk.

Attributable burden

Four key components are included in the estimation of the burden attributable to a given risk factor: the metric of burden being assessed (the number of deaths, YLLs, YLDs, or DALYs [the sum of YLLs and YLDs]); the exposure levels for a risk factor; the RR of a given outcome due to exposure; and the counterfactual level of risk factor exposure.[18] Estimates of attributable burden as DALYs for risk–outcome pairs were generated by using the following model:

$$AB_{jasgt} = \sum_{o=1}^{w} DALY_{joasgt} PAF_{joasgt}$$

where AB_{jasgt} is the attributable burden for risk factor j for age group a, sex s, location g, and year t; $DALY_{joasgt}$ is total DALYs for cause o (of w relevant outcomes for risk factor j) for age group a, sex s, location g, and year t; and PAF_{joasgt} is the PAF for cause o due to risk factor j for age group a, sex s, location g, and year t. The proportions of deaths, YLLs, or YLDs attributable to a given risk factor or risk factor cluster were analogously computed by sequentially substituting each metric in place of DALYs in the equation provided.

SDI analysis and forecasting

Socio-demographic Index (SDI) definition

The methods used to calculate SDI are described in detail in the 2019 Demographics capstone;[19,20] this section is largely reproduced from the appendix of that publication with updates for GBD 2021.

SDI overview

The SDI is a composite indicator of socio-demographic development status strongly correlated with health outcomes. In short, it is the geometric mean of 0 to 1 indices of total fertility rate in those under 25 years old (TFU25), mean education for those aged 15 years or older (EDU15+), and lag-distributed income per capita (LDI). For GBD 2021, after calculating SDI, values were multiplied by 100 for a scale of 0 to 100.

SDI calculation

GBD originally used the Human Development Index (HDI) methodology in developing SDI for the 2015 cycle. At the core of this method was the use of three covariate inputs: TFR in ages 15 to 49, EDU15+, and LDI per capita. The scale of the index was from 0 to 1. The observed minimum for each covariate over the estimation period determined the lower boundary of this range, whereas the observed maximum for each covariate over the estimation period determined the upper end of this range.

Further refinements to the method for calculating SDI have been implemented since that time. Beginning in GBD 2017, we decided to use TFU25 instead of the TFR component. The rationale for this was to attempt to better capture women's social status, given that it covers ages when women tend to enter the workforce and pursue further educational opportunities. It is also important that there has been a consistent decline in TFU25 over time in highly developed countries. In contrast, there have been rebounds in TFR driven by increasing fertility in older ages. The concordance correlation coefficient was 0·981 between SDI using the GBD 2016 method and the updated GBD 2017 method.

In order to improve the stability of the interpretation of SDI over time, we switched from relative index scales to absolute index scales during GBD 2016 when we noticed the introduction of subnational units led to stretched empirical minima and maxima.7 The minima and maxima of the scales were selected by looking at the relationships between each of the inputs and life expectancy at birth and under-5 mortality then identifying points of limiting returns at both high and low values, if they occurred prior to theoretical limits (eg, a EDU15+ of 0).

SDI was originally constructed for GBD 2015 by using the Human Development Index (HDI) methodology, wherein a 0 to 1 index value was determined for each of the original three covariate inputs (total fertility rate in those aged 15–49, EDU15+, and LDI per capita) by using the observed minima and maxima during the estimation period to set the scales.

During GBD 2016, we moved from using relative index scales to using absolute scales to enhance the stability of SDI interpretation over time because we noticed that the measure was highly sensitive to the addition of subnational units that tended to stretch the empirical minima and maxima. We selected the minima and maxima of the scales by examining the relationships each of the inputs had with life expectancy at birth and under-5 mortality and by identifying points of limiting returns at both high and low values if they occurred before theoretical limits (eg, a TFU25 of 0).

An index score of 0 therefore represents the point at which decreasing each covariate does not worsen selected health outcomes, and an index score of 1 represents the level at which increasing the level of

each covariate does not improve selected health outcomes. The means that a location with an SDI of 1 would have the theoretical maximum level of development relevant to these selected health outcomes. A location with an SDI approaching 0, on the other hand, would have the level of development relevant to these selected health outcomes approaching the theoretical minimum.

We computed the index scores underlying SDI as follows:

$$I_{Cly} = {(C_{ly} - C_{low})}\Big/{(C_{high} - C_{low})}$$

Where I_{Cly} – the index for covariate C, location l, and year y – is equal to the quotient of the difference between the value of that covariate in that location-year and the lower bound of the covariate and the difference between the upper and lower bounds for that covariate. We also note that the index value for TFU25 was computed as $1 - I_{TFU25ly}$ because lower TFU25s correspond to higher levels of development and thus higher index scores. For GBD 2021, we computed SDI for 983 national and subnational locations spanning the time period 1950–2021.

The composite SDI was calculated as the geometric mean of these three indices for a given location-year. The cutoff values used to determine quintiles for analysis were then computed using country-level estimates of SDI for the year 2021, excluding countries with populations less than 1 million. For reporting, final SDI values were multiplied by 100 to facilitate interpretation and engagement.

Epidemiological transitions

MR-BRT models

To investigate relationships between the prevalence of each indicator and social and economic conditions, MR-BRT splines were fit on SDI over the period from 1990 to 2021. Splines fit on SDI for indicator prevalence, *prev*, reflect the expected values (prevalence) of those indicators based on *SDI*. We used the following equation to estimate the relationship:

$$logit(prev_{l,y,a,s}) \sim \beta_{0,l} + spline(SDI_{l,y,a,s}) * \beta_1$$

Where *l* is the country or territory, *y* is the year, *a* is the age group, and *s* is the sex. Cubic splines were fit to prevalence in logit space to standard GBD locations, weighting each location evenly by setting the standard errors equal to 1 for all locations in logit space. Standard GBD locations refer to all 204 countries and territories plus subnational locations for India (30 states and 1 territory, divided into urban and rural), China (33 provinces), the USA (50 states, 1 territory), and Brazil (27 states). Data for China, India, the USA, and Brazil are also included at the country level. Subnational locations belong to countries where data quality is high and with populations over 200 million. Due to the absence of population-weighting, countries with subnational estimates included in the model play a predominant role in global association between SDI and indicator prevalence. Six knots were set in the model on the basis of SDI frequency, with one knot at the lowest SDI value observed from 1990 to 2021, one knot at the highest SDI value observed from 1990 to 2021, and four knots spaced at every 20 percentile of observed SDI between these knots. Models for most indicators were fit with decreasing monotonicity priors, meaning that the models were forced to show decreasing indicator prevalence as SDI increased. Models for EBF and overweight were exceptions (see table A18). The lowest and highest 2.5% of input

estimates relative to the spline were trimmed from the models. Models were fit in an age- and sex-specific manner and supplied with the mean SDI estimates for 204 countries and territories from 2012 to 2021, to produce location-, year-, age-, and sex-specific expected prevalence values. We then calculated weighted aggregates for both sexes (except for anaemia) and the GNT-specified age groups for each indicator using GBD population estimates.

Table A18. Specified parameters in epidemiological transition MR-BRT models, by indicator

Indicator	Prior spline monotonicity	Prior spline convexity	Age groups
LBW	decreasing	none	birth
EBF	increasing	concave	0-6 days<br>7-27 days<br>1-5 months
Stunting	decreasing	none	0-6 days<br>7-27 days<br>1-5 months<br>6-11 months<br>12-23 months<br>2-4 years
Wasting	decreasing	none	
Overweight	increasing	convex	2-4 years
Anaemia	decreasing	none	15 to 19 years<br>20 to 24 years<br>25 to 29 years<br>30 to 34 years<br>35 to 39 years<br>40 to 44 years<br>45 to 49 years

Expected values of indicator prevalence for GNT-specified age groups are shown at the region level in **Figure 2**. Results for individual countries and territories can be seen in **Figure S5**.

Expected annualised rate of change based on SDI

We calculated the expected annualised rate of change (ARC) for 204 countries and territories based on SDI between 2012 and 2021 for the prevalence of each indicator in every location. The expected ARC is calculated in the same manner as the observed (GBD estimate) ARC:

$$\frac{\ln\left(\frac{value_1}{value_2}\right)}{year_2 - year_1}$$

Where $value_1$ is the expected prevalence in $year_1$ (2012) and $value_2$ is the expected prevalence in $year_2$ (2021). We then calculated the difference in observed ARC and expected ARC for each location and GNT-specified age combination. Differences in observed ARC and expected ARC, by indicator, can be seen in **Figure S8**. EBF prevalence and SDI were not found to be associated, and therefore we chose to omit EBF from subsequent epidemiological transition results as their interpretation differs. The count of indicators (out of 5) with negative differences between observed ARC and expected ARC can be seen in **Figure 3**.

SDI forecasting

Forecasts of SDI were computed from forecasts of the three component measures: educational attainment, total fertility rate under age 25, and lag-distributed income. Forecasts of the components were rescaled and the geometric mean was computed using the same methods as above for the calculation of retrospective SDI. To adjust for the impacts of the COVID-19 pandemic, we revised income and education projections (described below) before incorporating into the SDI forecasts.

Education

We produced forecasts of education using the methods from Foreman et al, 2018.[21] Educational attainment (up to a maximum of 18 years) was assumed not to change after age 25. Forecasts were held constant after age 25 to prevent implausible changes in education within cohorts during older age, and conformed better with a cohort-specific fertility model, for which educational attainment was a covariate.

For age group intervals with a start year of less than or equal to 25, we first scaled mean years of education such that the maximum education level was 18 years. We then calculated age-, sex- and location-specific annualised rates of change (ARC) by a weighted average of annual differences in logit space (with recent years weighted more). The recency-weighting parameter was chosen through cross-validation, where the weight selected produced the smallest root-mean-square error (RMSE) at least 5% greater than the minimum RMSE of the cross-validation forecasts. Final ARCs were added to GBD 2019 draws year over year to produce forecast draws, denoted EDU_{lastd} of mean years of education for location l, age $a \leq 25$, sex s, future years t = 2021, ... , 2100, and draw d. For age groups with a start year over 25, the forecasted value was set to the previous value of the cohort, which was lagged by the width of the age group interval (5 years) due to the relationship

$$cohort\ birth = time - age.$$

For age groups indexed by the interval start $a = 30, 35, \dots, 95$ this is given by

$$\widehat{EDU}_{lastd} = \begin{cases} EDU_{l(a-5)s(t-5)d}, & t \leq 2019 + 5 \\ \widehat{EDU}_{l(a-5)s(t-5)d}, & t > 2019 + 5 \end{cases}$$

where $EDU_{l(a-5)s(t-5)d}$ and $\widehat{EDU}_{l(a-5)s(t-5)d}$ denote GBD past and future draws, respectively.

We estimated the effect of COVID-19 pandemic disruptions in schooling on educational attainment. IHME collected daily school closure data from the Oxford COVID-19 Government Response Tracker, government mandate primary documents, and local and international news sources(#REF). Closure data was split into primary and secondary education. The definition of a closure was “no in-person classroom activities for over 66% of students”, though in most cases a closure meant that all schools were closed. Daily closures were summed to annual counts and divided by 365 days to make proportions.

IHME extracted UNESCO data to map primary and secondary closure data to specific ages in each country. GBD education data was split from five-year age groups to single-year age groups by linear interpolation prior to the application of the closure data.

Shocks were applied over the course of the cohort since school closures may affect a cohort over more than one year. School closure proportions were scaled to the amount of education students would have gained without shocks by multiplying by the one-year absolute change in education before the shocks

were applied.To account for online education, IHME extracted country-specific fixed broadband subscriptions per 100 people from the International Telecommunication Union (ITU),12 and converted the statistic to per capita broadband subscriptions. IHME assumed that students can recover a maximum of half of their lost schooling through online education. Thus, the broadband data were scaled to be between zero and 0.5. The broadband data were then scaled again to the country-specific school closure proportions themselves. These values were added back to the closure proportions before they were applied to the education data as shocks.

To create long-term effects, a cumulative sum was applied to the scaled proportions over the full time series of the cohort. The cumulative values were then subtracted over the course of the whole cohort to generate education data with prolonged education disruptions. Data were subsequently converted back to period space, and the mean was taken over the single-year age groups to recreate the standard five-year age groups used for modelling.

Mean educational attainment over a cohort was defined as,

$$\bar{A} = \begin{pmatrix} a_1 \\ . \\ . \\ . \\ a_N \end{pmatrix}$$

Where α is the level of education attained by the cohort by a specific year.

Defining ρ_k as the fraction of education lost from group k, we define the cumulative shock value as,

$$c_i = \sum_{k=1}^{i} p_k \, , for\ i = 1, 2, \ldots, N$$

Thus,

$$\bar{C} = \begin{pmatrix} c_1 \\ . \\ . \\ . \\ c_N \end{pmatrix}$$

By subtraction, COVID-adjusted cohort educational attainment, $\bar{E}$, is

$$\bar{E} = \bar{A} - \bar{C}$$

Lag-distributed Income

Lag distributed income (LDI) per capita, which is a moving average transformation of gross domestic product (GDP) per capita and is one of three components of the SDI, was used in forecasting cause-specific mortality. LDI was computed by conducting a natural log transformation of the weighted average GDP with a 10-year lag.[22]

Data on retrospective gross domestic product (GDP) per capita were extracted from five leading sources of these estimates (World Bank, International Monetary Fund, United Nations, Penn World Tables, and The Maddison Project) and used methods described by James and colleagues[23] to generate a single series of GDP per capita using Gaussian processes. The resulting series spans 204 countries from 1950 to 2023 and includes uncertainty bounds based on concordance or missingness of the input data.

GDP estimates in the short term drew on predictions from several data sources which estimated the economic impacts of COVID-19 from 2021 to 2026, as well as the economic effects of the emerging conflict between Russia and Ukraine from 2022 to 2023.

GDP per working-age adult was then calculated as follows:

$$GDP_{working\ population} = GDP_{total\ population} \times \frac{Working\ population(20-64\ years\ old)}{Total\ population}.$$

Long-term GDP forecasts were created using GDP per worker, as compared to GDP per capita, because this improves out-of-sample predictive validity. Ensemble modelling techniques were implemented to generate 1000 projections from a broad set of models, and uncertainty was propagated across estimates. Models included indicators associated with ARIMA modelling, data recency weights, and a term for convergence to global growth rates. Model selection was determined by country-year-specific out-of-sample validation, as well as exclusion criteria such as statistical significance testing of estimated parameters, parameter estimates aligning with pre-determined causal priors, and observed growth rate from retrospective estimates. Projections from 2024 to 2100 were generated based on retrospective data through 2019.

Fertility

We produced forecasts of fertility using the updated modelling fertility framework 4 which differs from the published methods in Vollset et al, 2020.[24] Forecasts for age-specific fertility rates (ASFR) were used as a direct input to the population forecasting model and in forecasting future SDI.

The crux of the model is forecasting a cumulative cohort fertility quantity, CCF50, out to the 2100 cohort, followed by unfolding it into ASFR. CCF50 is defined as the average number of children born to an individual female from an observed birth cohort (indexed by year of birth) if she lived to the end of her reproductive lifespan (from age 15 through 49).

The past CCF50 for birth cohorts from 1945 to 1972 were used to forecast CCF50 for birth cohorts from 1973 to 2100. In the updated methods, we utilized not only female education and proportion of met need for contraception estimates, but also under-5 mortality and urbanicity estimates as covariates in the CCF50 sub-models. We forecasted CCF50 using three sub-models (with 2, 3 and 4 covariates) to generate an ensemble model forecast where all three sub-models were equally weighted.

From forecasted CCF50, we then derived future age-specific fertility rates (ASFR) forecasts for years 2022 to 2100 using a combination of linear mixed effect model, spline interpolation, and $ARIMA_{(1,0,0)}$ on residuals to estimate the age pattern of fertility for each cohort. Once the 15-49 ASFR values are obtained, we infer the 10-14 and 50-54 values based on their ratios to the rest of the age pattern during the last observed year (2021).

Forecasts for ASFR were used as a direct input to the populations forecasting model (section 2.4) and in forecasting future SDI, which is an input to cause-specific mortality (section 2.2) and migration models.

The detailed description of fertility forecasting methods can be found in GBD 2021 Fertility and Forecasting Collaborators.[25]

Prevalence forecasting

To forecast future prevalence, we first modelled the average relationships between sex-, age-, and indicator-matched SEVs and indicator prevalence in a constrained MR-BRT model with SDI as a linear fixed effect. The method for forecasting future SEVs is described below, followed by a description of the method for using future SEVs and SDI to predict the future prevalence.

SEV forecasting

To forecast summary exposure values (SEVs), we are using the ensemble modelling approach which uses 12 different submodels (or child models). For the child models, we use two main modelling approaches: annualised rates of change (ARCs) and a two-stage spline model based on MR-BRT. Each of these models have six different recency-weighting parameters ranging from 0 to 2.5 (the higher the weight, the more weight is given to recent years).

For ARC child models, we calculate the age-standardised sex-specific and location-specific annual change in logit of the SEV. To account for the effect of noisy data, we replaced annual changes outside the 2.5th and 97.5th percentiles with those corresponding percentile-values. The two-stage MR-BRT child models use the first stage to fit age-standardised sex-specific logit of the SEV on SDI and location-specific random intercept based on location; and then it uses the second stage to account for the residuals from the model by linearly modelling them on time (in our case, year variable was used as a covariate).

$$logit(SEV_{c,s,t}) = \beta_0 + \beta_1 spline(SDI_{c,t}) + \alpha_{c,s} + \varepsilon_{c,s,t} \; , \qquad (1)$$

where *logit($SEV_{c,s,t}$)* is the logit of the age-standardised summary exposure value in country *c*, sex *s* and year *t*, β_0 is an intercept, β_1 is a coefficient matrix, *spline* is the spline with five knots placed evenly across the distribution of SDI data and it assumes both right and left linear tails, is a location-specific random intercept, and is the residual.

$$logit(\varepsilon_{c,s,t}) = year_t + \lambda + \Psi_{c,s,t} \; , \qquad (2)$$

where is a fixed intercept value, and is an error term.

The weight of each submodel is defined by running out-of-sample predictive validity experiments. We train each submodel based on GBD 2019 estimates from 1990–2009 and validate each submodel based on 2010–2019 years estimates. We measure each child model's performance using root-mean-squared-error (RMSE) based on which we determine sampling weights of each child model.

We then produce each submodel forecasts based on 1990–2019 training dataset (1000 draws in each submodel). For ARC child models, we use the calculated annual change with corresponding recency-weighting parameter to produce 2021–2050 SEV forecasts. For MR-BRT child models, we use SDI values (1000 draws) forecasted by Vollset et al. to obtain forecasting values of SEVs based on the model fit.

We then obtain the final ensemble forecasts (1000 draws) based on draws from the child models using the sampling weights from the out-of-sample experiments (**Figure A4**).

Figure A4. Ensemble modelling framework

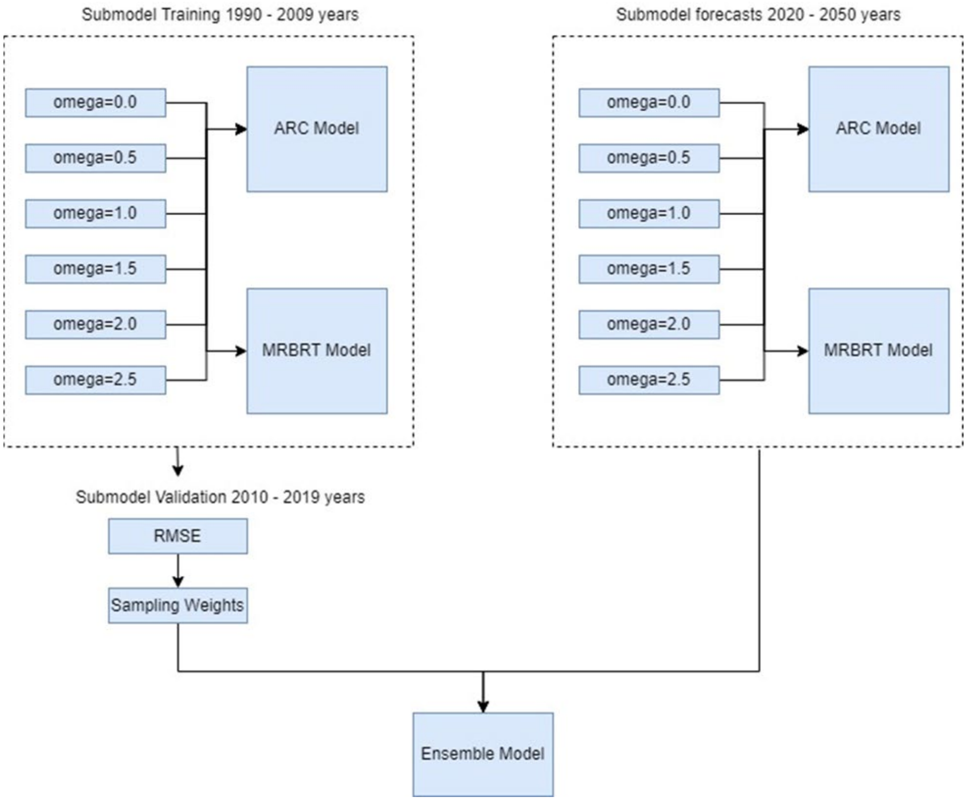

Using SEV forecasts to predict future prevalence

To forecast future prevalence, we first modelled the average relationships between sex-, age-, and indicator-specific SEVs and indicator prevalence. We used MR-BRT[1] to fit a cascading spline model with random intercepts.[26] This approach borrows strength across geographies, while allowing individual countries to differ from global or super-regional trends where supported by data. We fit one global spline on SEV including data from 1990–2021, 21 splines by GBD region, and 204 country-specific splines, with each further disaggregated model borrowing strength from the previous one in the hierarchy. That is, in each disaggregated model, the estimated coefficients from one level higher in the hierarchy are used as Gaussian priors (on the coefficients). SDI was included as a linear covariate in all models. Because GBD does not estimate SEVs for anaemia impairment, female iron-deficiency SEVs were the independent variable for anaemia models. Details on MR-BRT and cascading spline specifications are provided in the MR-BRT methods and Cascading splines methods section, below.

Model training and performance refinement

To test and refine model performance for each GNT indicator, we trained sex- and age-specific models on mean GBD estimates in each country from 1990 to 2014. The model was used to predict prevalence for 1990 to 2014 (in-sample) and 2015 to 2021 (out-of-sample). We then calculated the root-mean-

square error (RMSE) comparing predicted values to GBD prevalence estimates from the two periods to gauge the model's in-sample and out-of-sample predictive validity. Parameter adjustments were made to optimise each indicator model's predictive performance (ie, minimise RMSEs and temporal trends in residuals).

MR-BRT methods[1]

The following is description of the final model forms fit and passed to cascading splines that were used to predict future prevalence.

The global (stage 1) sex-, age-, and indicator-specific MR-BRT models were fit over the period from 1990 to 2021 with indicator specific SEVs fit as splines, SDI fit as a linear fixed effect, and a random intercept fit for each country. The global model equation is provided in the Cascading spline methods section, below. High BMI SEV in children 5 to 9 years was used instead of high BMI SEV in ages 2 to 4 year in child overweight models because forecasts of child overweight SEVs in children 2 to 4 years are not available due to a change in the age groupings between GBD rounds 2019 and 2021. Models trained with retrospective estimates of high BMI SEV for children 5 to 9 years predicted the prevalence of overweight in children 2 to 4 years with sufficient accuracy. Splines fit on SEV for indicator prevalence reflect the expected values (prevalence) based on SEV. Cubic splines were fit to prevalence in logit space to all 204 countries and territories, weighting each country by setting the standard errors equal to values calculated from the uncertainty intervals of GBD prevalence estimates in logit space. These logit-scale standard errors were calculated using the delta method:

$$logit(standard\ error) = \frac{logit(prevalence_{0.975}) - logit(prevalence_{0.025})}{3.92}$$

Five knots were set in the model on the basis of SEV frequency, with one knot at the lowest SEV observed from 1990 to 2021, one knot at the highest SEV observed from 1990 to 2021, and three knots spaced at every quartile of observed SEV between these knots. Models were fit without monotonicity priors, meaning that the models were free to show increasing or decreasing indicator prevalence as SEV increased. Trimming was not specified for any model. The anaemia model was fit with a concave convexity prior, meaning that the models were forced to be a concave function between SEV and logit prevalence. That is, the second derivative of f(SEV) is ≤0 everywhere.

For the GBD 2021 cycle, the post neonatal and 1–4 years age groups were broken apart into four age groups: 1–5 months, 6–11 months, 12–23 months, and 2–4 years. Stunting and wasting SEV and prevalence estimates from GBD 2021 were aggregated to the post neonatal and 1–4 years age groups used in GBD 2019 prior to fitting MR-BRT models, in order to align with the available SEV forecast age groups. Age group aggregation used GBD 2021 population estimates. Stunting and wasting SEVs are not estimated by GBD 2021 for early neonatal and late neonatal ages. Therefore, SEV estimates for the aggregate post neonatal age group were used to model prevalence for the two youngest age groups.

Cascading spline methods

Cascading spline models with stage variables of region and country were used to improve model predictions by borrowing information across countries within the same region. The cascade concept and notation has been described in detail in a prior publication.[26]

We modelled the relationship between indicator-specific SEV and the prevalence with a cascading random spline model with MR-BRT. We used the following set of equations to estimate the average relationship between the prevalence, *prev*, and the summary exposure value, *SEV*:

Stage 1

Global model $$logit(prevalence_{l,y,a,s}) \sim \beta_{0,l} + SDI_{l,y} * \beta_1 + spline(SEV_{l,y,a,s}) * \boldsymbol{\beta}_2$$

Stage 2

Region model $$logit(prevalence_{l,y,a,s}) \sim \beta_{0,l} + SDI_{l,y} * \hat{\beta}_1 + spline(SEV_{l,y,a,s}) * \boldsymbol{\beta}_{2\,r}$$

$$\boldsymbol{\beta}_{2\,r} \sim N(\boldsymbol{\beta}_2, \sigma_{2\,r}^2 * \boldsymbol{I})$$

Stage 3

Country model $$logit(prevalence_{l,y,a,s}) \sim \beta_{0,l} + SDI_{l,y} * \hat{\beta}_1 + spline(SEV_{l,y,a,s}) * \boldsymbol{\beta}_{2\,l}$$

$$\boldsymbol{\beta}_{2\,l} \sim N(\boldsymbol{\beta}_{2\,r}, \sigma_{2\,l}^2 * \boldsymbol{I})$$

Where *y* is the year, *a* is the age group, *s* is the sex, *r* is the region, and *l* is the country or territory. In stage 1 we fit a global model to all available prevalences. We fit a cubic spline with a linear tail on the right side to the logit of the prevalence. Each stage fit the same model to a subset of data, leveraging the estimates from the previous stage as a prior on the subsequent stage. In this way, the global model informed the region models; the region model informed the country (location, *l)*-specific model. In the equations above, items in bold ($\boldsymbol{\beta}$) represent matrices, and $\mathbf{I}$ is the identity matrix. Other values including (*prevalence, SDI,* and σ) are vectors, and $N()$ represents the Gaussian distribution. The notation $spline$ () refers to the basis spline design matrix described in the appendix of a prior publication.[26]

We manually selected several parameters to inform the spline models. Theta is a parameter that informs the strength of the prior when moving from one stage to the next in each step of the cascade. For most indicators, we selected thetas of 3 and 3 when moving between stages 1 and 2, and stages 2 and 3, respectively. For child overweight, we selected thetas of 1 and 4 when moving between stages 1 and 2, and stages 2 and 3, respectively. A smaller theta leads to a larger emphasis on the $\boldsymbol{\beta}$ from the earlier stage of the model in the estimation of $\boldsymbol{\beta}$ for the next stage.

We fit indicator-specific cascade models using the β_2 estimates from each stage as a prior for the subsequent step, such that the estimates for the super-region drew strength from the global model, and the country-specific estimates drew strength from the regional and global models. The value of β_1, on SDI was first estimated in the global model, and then set as fixed in the region and country models.

Prediction, intercept shifting, and aggregation

We fit the final cascading spline model for each indicator to the full set of estimates from 1990 to 2021. The full model was then applied to the pertinent country-year-age-sex-specific SEV forecast draws and mean country-, year-specific SDI forecasts to predict country-year-age-sex-specific prevalence draws from 2021 to 2050.

It was necessary to shift all future prevalence values to be continuous with GBD 2021 estimates. The prevalence forecasts for each indicator included the year 2021, for which GBD 2021 prevalence estimates were available. To avoid potential negative values that can occur with intercept shifting in natural space, we shifted all forecast trajectory draws in logit space by the same shift constant such that

the country-specific mean prevalence of the shifted trajectory draws matched the mean prevalence value for 2021 from GBD. This shift constant was calculated through an optimization algorithm that minimized this difference between the mean of the logit-space draws and the logit-tranformed GBD 2021 estimate. The result of this shift was that the mean of the future trajectories matched continuously with the GBD 2021 estimations.

For each indicator except anaemia, these intercept-shifted country-year-age-sex-specific prevalence forecast draws were then aggregated up to country-year-specific prevalence forecast draws for children of both sex, in the desired (GNT-specified) age groups using country-year-age-sex-specific population forecasts (described below). Intercept-shifted anaemia country-year-age-sex-specific, prevalence forecast draws were then aggregated up to country-year-specific prevalence forecast draws for women of reproductive age (15 to 49 years) using country-year-age-specific female population forecasts. Country-year-specific prevalence forecast draws were also aggregated to the region, super-region, and global level.

We calculated the mean prevalence and uncertainty intervals (2.5th and 97.5th percentiles) from the shifted and aggregated draws for each desired location, year, and age combination.

Forecasting population

Our methods for forecasting population, as well as the pipelines generating its requisite upstream inputs (life expectancy, ASFR, migration, and sex ratio at birth) are directly inherited from Vollset et al.[24]

Livebirth forecasts

Livebirth forecasts were calculated by taking the product of forecasted ASFRs and forecasted maternal age-specific populations and summing across all age groups. The country-specific sex ratios were calculated from GBD 2021 livebirth estimates for the year 2021. These sex ratios were applied to the livebirth forecasts to produce sex-specific livebirth forecasts for each country from 2021 to 2050. Livebirth forecasts were intercept-shifted to align with GBD 2021 livebirth estimates for the year 2021.

Custom forecasts of new child age groups

Several age group categories were further divided between the 2019 and 2021 GBD cycles; specifically, the post neonatal (ages 28–364 days) and ages 1–4 years age groups were revised to 1–5 months, 6–11 months, 12–23 months, and 2–4 years. As all forecasts were trained using GBD 2019 estimates and intercept-shifted to GBD 2021 estimates, this required a method to extrapolate the child populations of the new age groups from the estimates of the GBD 2019 age group populations. Therefore, additional modelling was necessary to enable count estimation and appropriate sex and regional aggregations of both EBF prevalence in infants <6 months and for overweight/obesity prevalence in ages 2–4 years.

Future population of ages 1–5 months

We predicted the future population of ages 1–5 months from 2021 to 2050 using a cascading splines approach informed by a MR-BRT model with SDI and the population for ages 7–27 days as linear fixed effects, and the post neonatal population as a linear spline covariate with four equally spaced knots.

This final model form had considerably lower RMSE in comparison with preliminary models with:

a. Just one age group as a linear fixed effect OR as a linear spline

b. Both age groups, with the population for ages 7–27 days as a linear spline and the post neonatal population as a linear fixed effect

Predicted values from the final model were then intercept shifted (described below). We summed the shifted population forecasts for ages 1–5 months with the population forecasts for early neonatal and late neonatal age groups to create the population forecast for ages <6 months.

Future population of ages 2–4 years

We predicted the future population of ages 2–4 years from 2021 to 2050 using cascading splines informed by a MR-BRT model with SDI and the post neonatal population as linear covariates, and the population of ages 1–4 years as a linear spline covariate with four equally spaced knots. Population forecasts were intercept shifted.

Intercept shifting

The livebirth forecasts and custom child age group population forecasts described above contained one year (2021) that overlapped with the GBD 2021 livebirth and population estimates, respectively. We calculated the mean difference between forecasted values and GBD estimated population for each age, sex, and country. These differences were then applied to the forecast population at the most detailed level to shift all future values. Shifted age group population forecasts were then used to aggregate each respective indicator forecasts to the appropriate locations and age groups.

Demographic shifts

To explore projected demographic shifts from 2012 to 2030 in the 28 countries that we project will attain the stunting target in 2030, we calculated the location-specific general fertility rate (GFR) in 2012 and 2030 in these locations. GFR was calculated as the number of livebirths born to women of reproductive age (WRA; age 15 to 49 years) in one year divided by the population of women in the same year.[27] This simplified GFR calculation includes person-time contributions of women who cannot give birth due to death in the period or recent pregnancy, which vary across locations; however, it is suitable for comparisons within the same location over time. We then calculated the ratio of the 2025 to the 2021 value for livebirths, child population under 5 years, WRA population, and GFR to determine whether fertility was projected to increase or decrease over the period, and to illustrate the degree to which change in child population may be due to shifts in livebirths and/or declines in the population of WRA (a proxy for emigration). Results of this analysis can be seen in **Figure S3.**

Software packages

Table A19. R software packages used in modeling pipelines, by indicator

LBW	Child overweight	Child growth failure	Breastfeeding	Anaemia
actuar	actuar	Cairo	argparse	actuar
Amelia	argparse	cowplot	binom	binom
argparse	arm	data.table	boot	boot
copula	assertable	dfoptim	crosswalk	cowplot
cowplot	boot	dplyr	data.table	crosswalk002
crosswalk	caret	fitdistrplus	dbplyr	data.table
data.table	chron	ggplot2	dplyr	DBI
dfoptim	classInt	ggpubr	ggplot2	dfoptim
dplyr	compiler	GoFKernel	ini	dplyr
fitdistrplus	cowplot	ini	magrittr	fitdistrplus
ggplot2	crosswalk	lubridate	MASS	gganimate
ggpubr	data.table	magrittr	msm	ggforce
GoFKernel	DBI	nloptr	openxlsx	ggplot2
haven	dfoptim	openxlsx	parallel	ggpubr
Hmisc	doParallel	plyr	readxl	ggrepel
ini	dplyr	RColorBrewer	RMySQL	ggridges
lme4	dpylr	readxl	scales	ggthemes
lubridate	fitdistrplus	scales	stats	GoFKernel
magrittr	foreach	stats	stringr	googlesheets4
metafor	foreign	stringr		grid
mrbrt001	ggplot2	tidyr		gridExtra
msm	ggpmisc			gtable
nloptr	glmnet			gtools
openxlsx	googlesheets			haven
parallel	grid			Hmisc
readstata13	gridExtra			ini
readxl	gtable			kableExtra
stats	gtools			lubridate
stringr	haven			magrittr
survey	Hmisc			maptools
VineCopula	ini			matrixStats
	iterators			mrbrt001
	knitr			mrbrt002
	lattice			mrbrt003
	lazyeval			msm
	leaps			nloptr
	lme4			odbc
	magrittr			openxlsx
	maptools			p
	MASS			pacman
	modi			parallel
	mortcore			patchwork
	mortdb			plotly
	mrbrt001			plyr
	msm			raster
	MuMIn			RColorBrewer
	mvtnorm			readstata13
	ncdf4			readxl
	onehot			reticulate
	openxlsx			rgdal
	pacman			rhdf5
	parallel			RMySQL
	plyr			scales
	RColorBrewer			sf
	Rcpp			shiny
	readstata13			sp
	readxl			stats
	RefManageR			stringdist
	reshape2			stringr
	reticulate			survey
	rhdf5			terra
	rio			tidyverse
	RMySQL			tinytex
	scales			vctrs
	stringi			viridis
	stringr			xlsx
	zipfR			zipfR
	zoo			

Figure A5. Prevalence data availability for GNT indicators by GBD region, 1990−2021

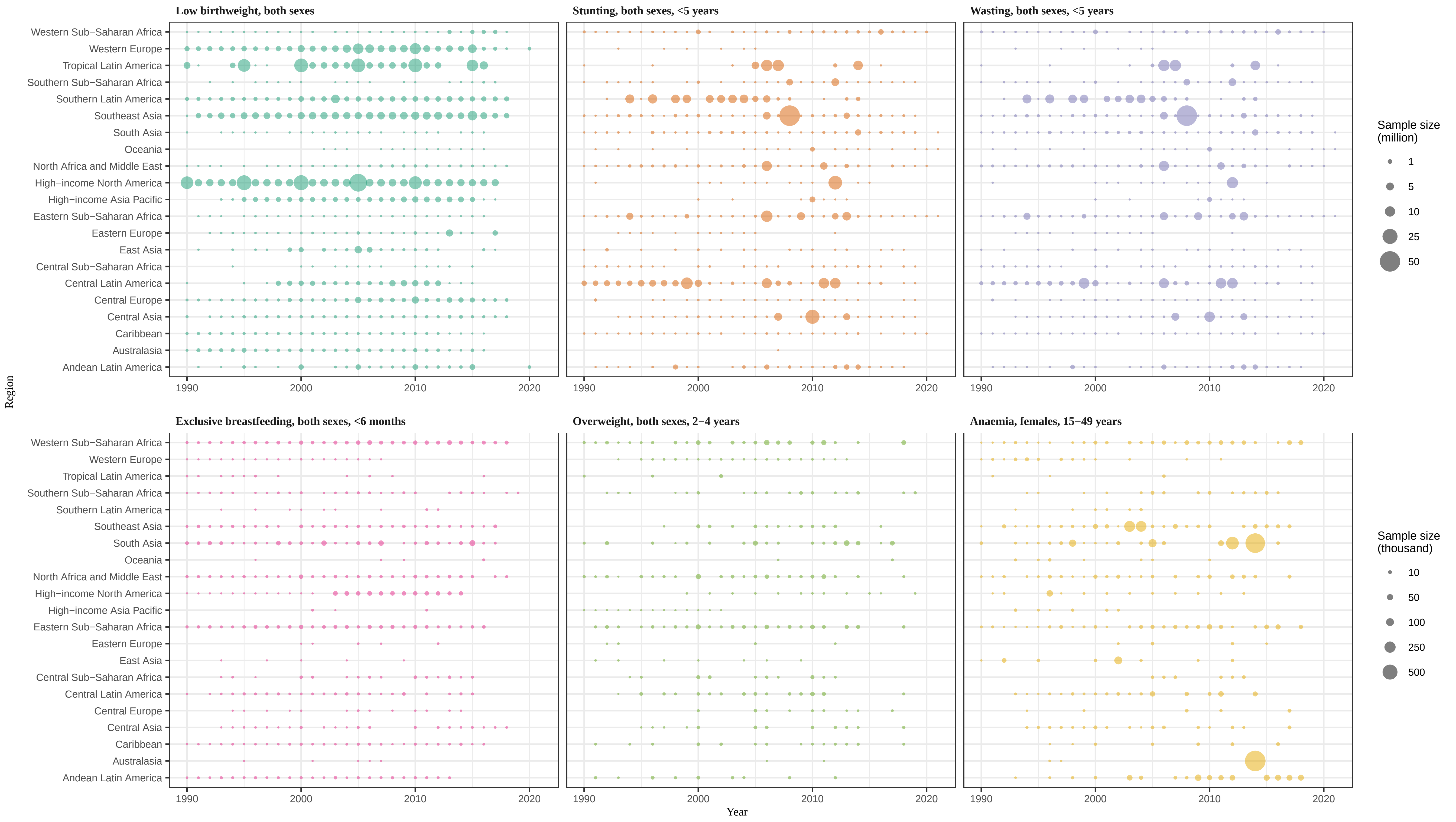

References

1 Murray CJL, Aravkin AY, Zheng P, *et al.* Global burden of 87 risk factors in 204 countries and territories, 1990–2019: a systematic analysis for the Global Burden of Disease Study 2019. *The Lancet* 2020; **396**: 1223–49.

2 CDC. CDC's Infant and Toddler Nutrition website. Cent. Dis. Control Prev. 2022; published online April 11. https://www.cdc.gov/nutrition/infantandtoddlernutrition/definitions.html (accessed June 13, 2022).

3 WHO | Breastfeeding. WHO. http://www.who.int/nutrition/topics/exclusive_breastfeeding/en/ (accessed June 13, 2022).

4 de Onis M, Garza C, Onyango AW, Rolland-Cachera M-F, le Comité de nutrition de la Société française de pédiatrie. [WHO growth standards for infants and young children]. *Arch Pediatr Organe Off Soc Francaise Pediatr* 2009; **16**: 47–53.

5 Obesity Classification. World Obes. Fed. https://www.worldobesity.org/about/about-obesity/obesity-classification (accessed July 1, 2022).

6 World Health Organization. Haemoglobin concentrations for the diagnosis of anaemia and assessment of severity. World Health Organization, 2011 https://apps.who.int/iris/handle/10665/85839 (accessed July 1, 2022).

7 Short-term effects of breastfeeding: a systematic review on the benefits of breastfeeding on diarrhoea and pneumonia mortality. https://apps.who.int/iris/handle/10665/95585 (accessed July 1, 2022).

8 de Onis M, Garza C, Onyango AW, Rolland-Cachera M-F, le Comité de nutrition de la Société française de pédiatrie. [WHO growth standards for infants and young children]. *Arch Pediatr Organe Off Soc Francaise Pediatr* 2009; **16**: 47–53.

9 Wang Y, Chen HJ. Use of Percentiles and Z -Scores in Anthropometry. *Handb Anthr Phys Meas Hum Form Health Dis* 2012; : 29–48.

10 Olofin I, McDonald CM, Ezzati M, *et al.* Associations of Suboptimal Growth with All-Cause and Cause-Specific Mortality in Children under Five Years: A Pooled Analysis of Ten Prospective Studies. *PLOS ONE* 2013; **8**: e64636.

11 Ng M, Freeman MK, Fleming TD, *et al.* Smoking Prevalence and Cigarette Consumption in 187 Countries, 1980-2012. *JAMA* 2014; **311**: 183–92.

12 GBD 2021 Anaemia Collaborators. Prevalence, years lived with disability, and trends in anaemia burden by severity and cause, 1990-2021: findings from the Global Burden of Disease Study 2021. *Lancet Haematol* 2023; **10**: e713–34.

13 Fitzgerald R, Manguerra H, Arndt MB, *et al.* Current dichotomous metrics obscure trends in severe and extreme child growth failure. *Sci Adv* 2022; **8**: eabm8954.

14 Zheng P, Afshin A, Biryukov S, *et al.* The Burden of Proof studies: assessing the evidence of risk. *Nat Med* 2022; **28**: 2038–44.

15 McDonald CM, Olofin I, Flaxman S, *et al.* The effect of multiple anthropometric deficits on child mortality: meta-analysis of individual data in 10 prospective studies from developing countries. *Am J Clin Nutr* 2013; **97**: 896–901.

16 Data Sharing and Inductive Learning — Toward Healthy Birth, Growth, and Development | NEJM. https://www.nejm.org/doi/10.1056/NEJMp1605441?url_ver=Z39.88-2003&rfr_id=ori:rid:crossref.org&rfr_dat=cr_pub%20%200www.ncbi.nlm.nih.gov (accessed July 1, 2022).

17 Swaminathan S, Hemalatha R, Pandey A, *et al.* The burden of child and maternal malnutrition and trends in its indicators in the states of India: the Global Burden of Disease Study 1990–2017. *Lancet Child Adolesc Health* 2019; **3**: 855–70.

18 GBD 2017 Risk Factor Collaborators. Global, regional, and national comparative risk assessment of 84 behavioural, environmental and occupational, and metabolic risks or clusters of risks for 195 countries and territories, 1990-2017: a systematic analysis for the Global Burden of Disease Study 2017. *Lancet Lond Engl* 2018; **392**: 1923–94.

19 Kyu HH, Abate D, Abate KH, *et al.* Global, regional, and national disability-adjusted life-years (DALYs) for 359 diseases and injuries and healthy life expectancy (HALE) for 195 countries and territories, 1990–2017: a systematic analysis for the Global Burden of Disease Study 2017. *The Lancet* 2018; **392**: 1859–922.

20 Wang H, Abbas KM, Abbasifard M, *et al.* Global age-sex-specific fertility, mortality, healthy life expectancy (HALE), and population estimates in 204 countries and territories, 1950–2019: a comprehensive demographic analysis for the Global Burden of Disease Study 2019. *The Lancet* 2020; **396**: 1160–203.

21 Forecasting life expectancy, years of life lost, and all-cause and cause-specific mortality for 250 causes of death: reference and alternative scenarios for 2016–40 for 195 countries and territories - The Lancet. https://www.thelancet.com/journals/lancet/article/PIIS0140-6736(18)31694-5/fulltext (accessed July 1, 2022).

22 Financing Global Health 2018: Countries and Programs in Transition. Inst. Health Metr. Eval. 2019; published online April 10. https://www.healthdata.org/policy-report/financing-global-health-2018-countries-and-programs-transition (accessed July 1, 2022).

23 James SL, Gubbins P, Murray CJ, Gakidou E. Developing a comprehensive time series of GDP per capita for 210 countries from 1950 to 2015. *Popul Health Metr* 2012; **10**: 12.

24 Vollset SE, Goren E, Yuan C-W, *et al.* Fertility, mortality, migration, and population scenarios for 195 countries and territories from 2017 to 2100: a forecasting analysis for the Global Burden of Disease Study. *The Lancet* 2020; **396**: 1285–306.

25 Bhattacharjee NV, Schumacher AE, Aali A, *et al.* Global fertility in 204 countries and territories, 1950–2021, with forecasts to 2100: a comprehensive demographic analysis for the Global Burden of Disease Study 2021. *The Lancet* 2024; published online March 20. DOI:10.1016/S0140-6736(24)00550-6.

26 Causey K, Fullman N, Sorensen RJD, *et al.* Estimating global and regional disruptions to routine childhood vaccine coverage during the COVID-19 pandemic in 2020: a modelling study. *The Lancet* 2021; **398**: 522–34.

27 Schoumaker B. A Stata module for computing fertility rates and TFRs from birth histories: tfr2. *Demogr Res* 2013; **28**: 1093–144.
