# Supplementary material for: Global, regional, and national progress towards the 2030 global nutrition targets and forecasts to 2050: a systematic analysis for the Global Burden of Disease Study 2021
Source: Lancet. 2024 Dec 21;404(10471):2543–83. doi: 10.1016/S0140-6736(24)01821-X (PMC11703702; doi:10.1016/S0140-6736(24)01821-X)
Supplement: Supplementary appendix 2 [file mmc2.pdf]

Appendix 2 results, to “Global, regional, and national progress towards the 2030 global nutrition targets and forecasts to 2050: a systematic analysis for the Global Burden of Disease Study 2021”

Figure S1. Relative distance between projected indicator prevalence and target, 2030……………….........2

Figure S2. Maps of relative gaps between projected indicator prevalence and target, 2030………….…....4

Figure S3. Annualised rate of change (ARC) 2012 to 2021 vs. required ARC 2022 to 2030 to attain 2030 target……..………………….5

Figure S4. Global maps of indicator metrics, 2012, 2021, 2030 ………………………………………………….…….…7

Figure S5. Co−evolution of GNT indicator prevalence by region…………………………………………………………13

Figure S6. Ratios of observed to expected prevalence based on SDI, 2012……………………………………..……34

Figure S7. Ratios of observed to expected prevalence based on SDI, 2021………………………………………..….35

Figure S8. Differences in observed ARC and expected ARC based on SDI, 2012 to 2021…………………..……..36

Figure S9. Comparison of annual rates of change from 2012 to 2021: prevalence vs. attributable burden by region………..……….…..…37

Figure S10. Projected year of next target attainment (2031 to 2050)…………………………………………….……58

Figure S11. Projected number of targets not met by 2050……………………………………………………….….……59

Figure S12. Ratio of projected 2050 population to estimated 2021 population, <5 years, both sexes.……60

Table S1. Global, super−regional, regional, and country/territory nutrition indicator attributable disability−adjusted life years, 2012 and 2021…………………………………………………………………………….……….61

Table S2. Spearman's rank correlation coefficients, by sex, standard GBD locations, 1990 – 2021……….100

Table S3. Ratio of projected 2030 to estimated 2012: livebirths, population of children under 5 years, population of females 15 to 49 years, and general fertility rate in 28 countries projected to meet the stunting Global Nutrition Target in 2030…………………………………………………………………………………….…101

Table S4. Annualised rates of change (ARC) 2012 to 2021: observed prevalence, expected prevalence, difference, attributable burden……………………………………………………………………………………..……………102

Table S5. Global, regional, and country number of children with stunting, 2012, 2021, and projected for 2030 and 2050..………….131

Figure S1. Relative distance between projected indicator prevalence and target, 2030

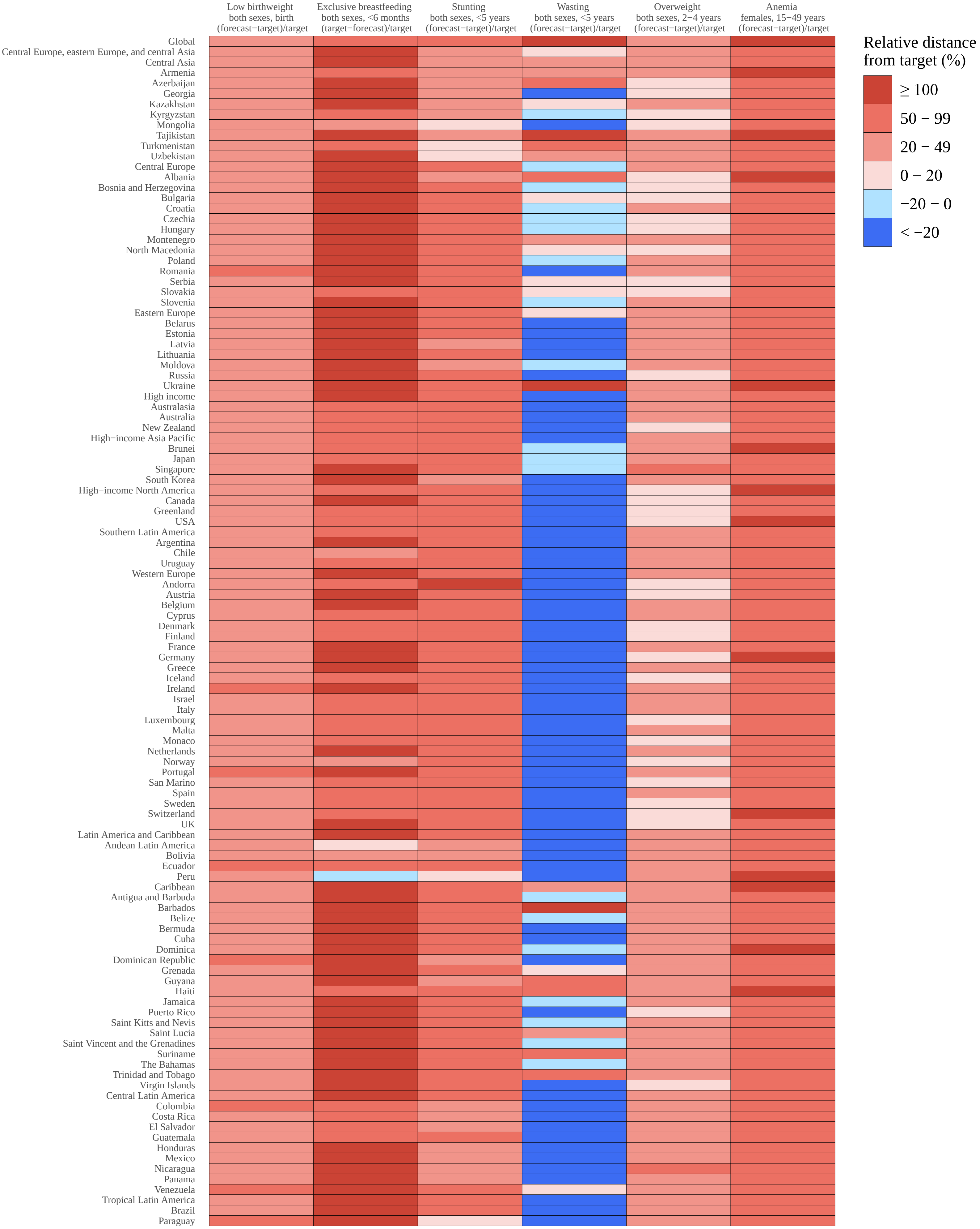

Figure S1. Relative distance between projected indicator prevalence and target, 2030

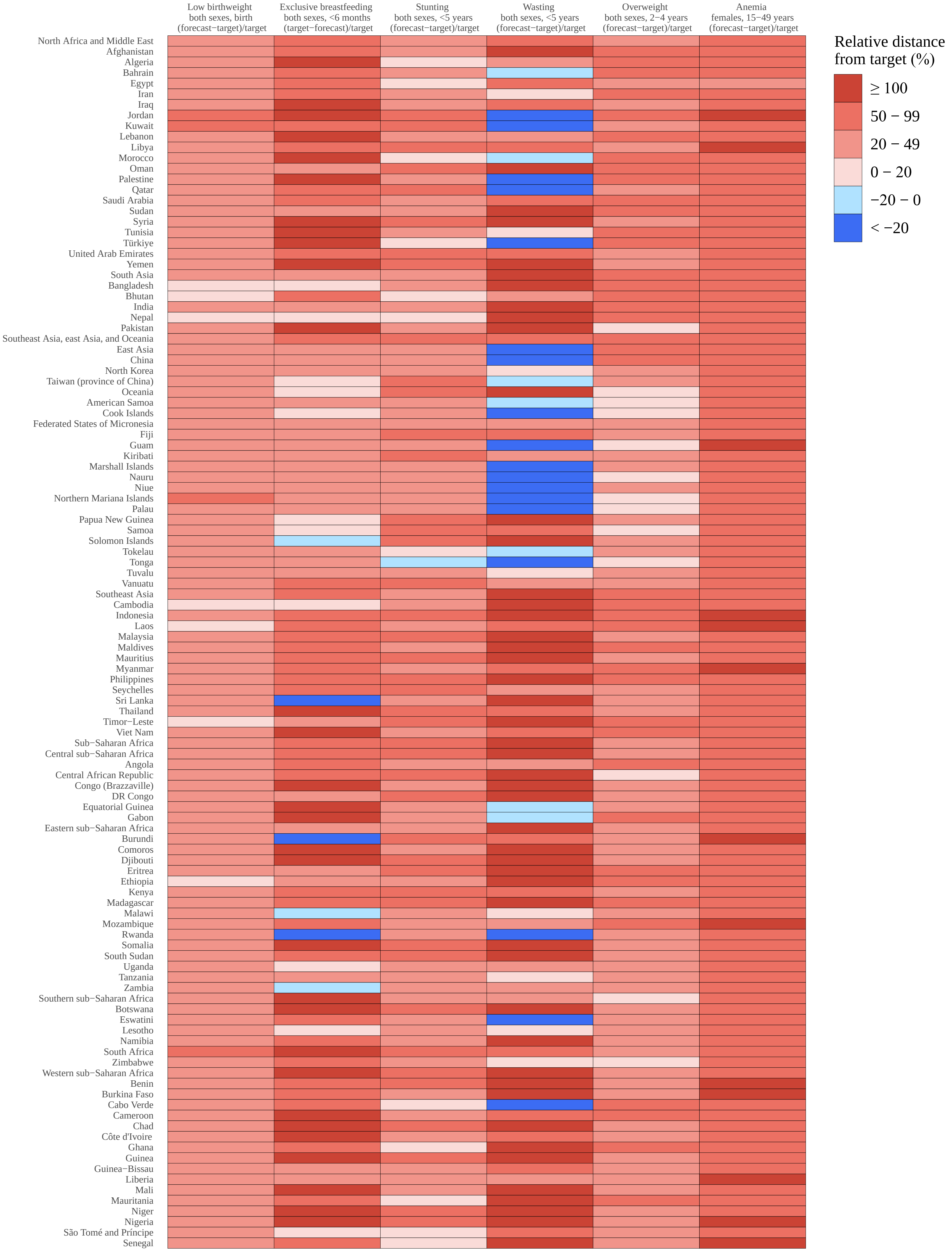

Figure S2. Maps of relative gaps between projected indicator prevalence and target, 2030

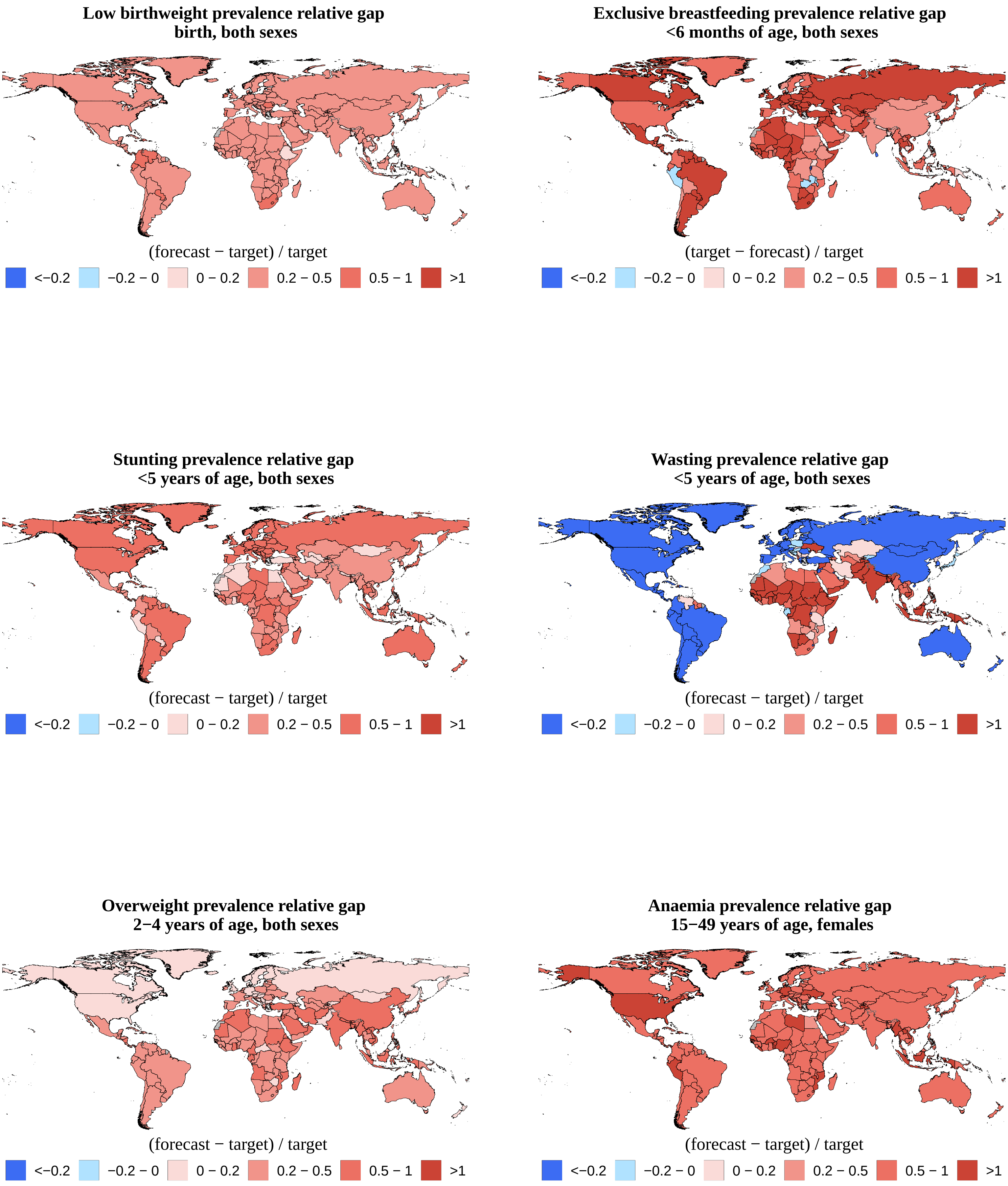

Figure S3. Annualised rate of change (ARC) 2012 to 2021 vs. required ARC 2022 to 2030 to attain 2030 target

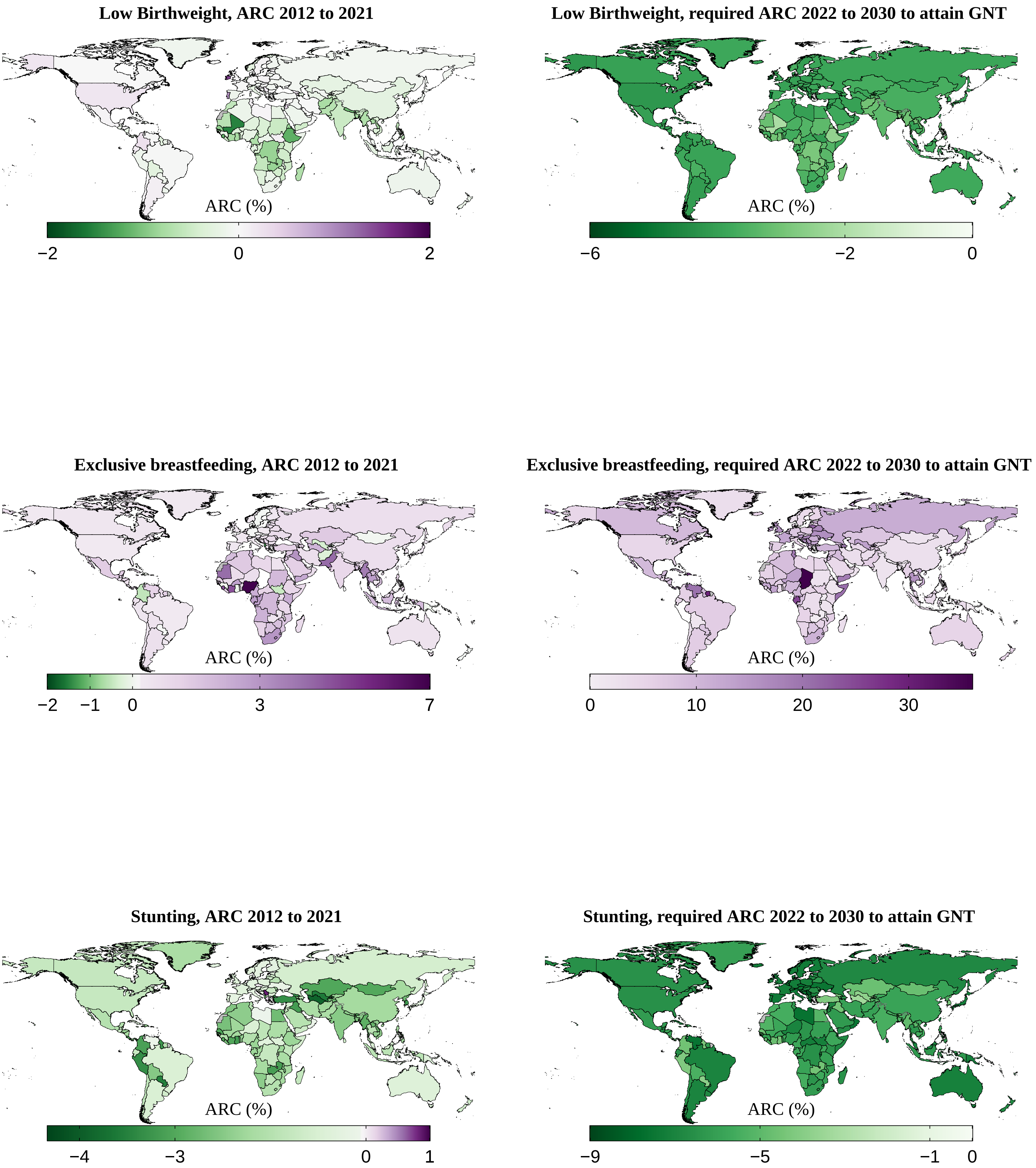

Figure S3. Annualised rate of change (ARC) 2012 to 2021 vs. required ARC 2022 to 2030 to attain 2030 target

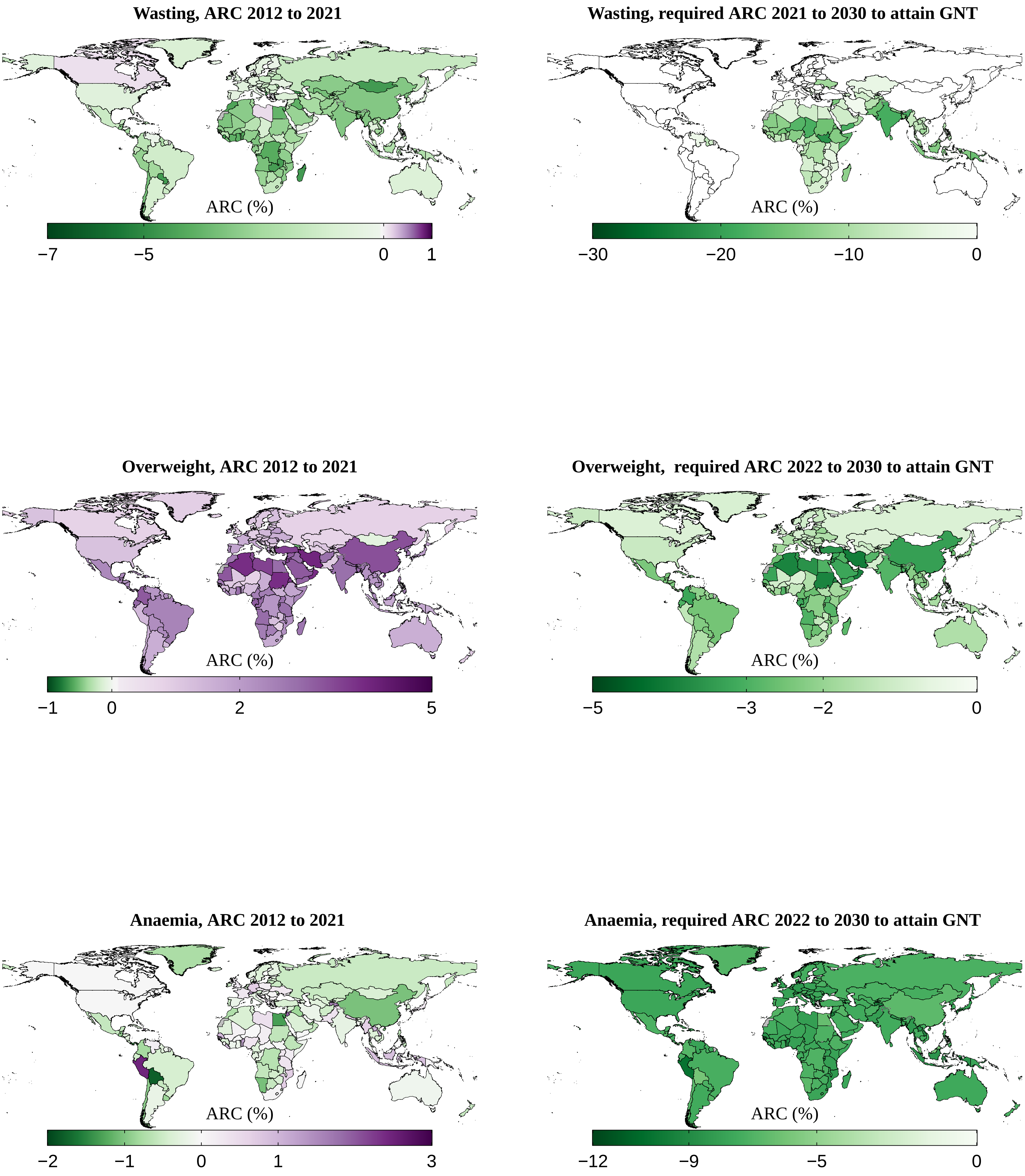

Figure S4. Global maps of indicator metrics, 2012, 2021, 2030

Low birthweight incidence (%), both sexes, birth, 2012

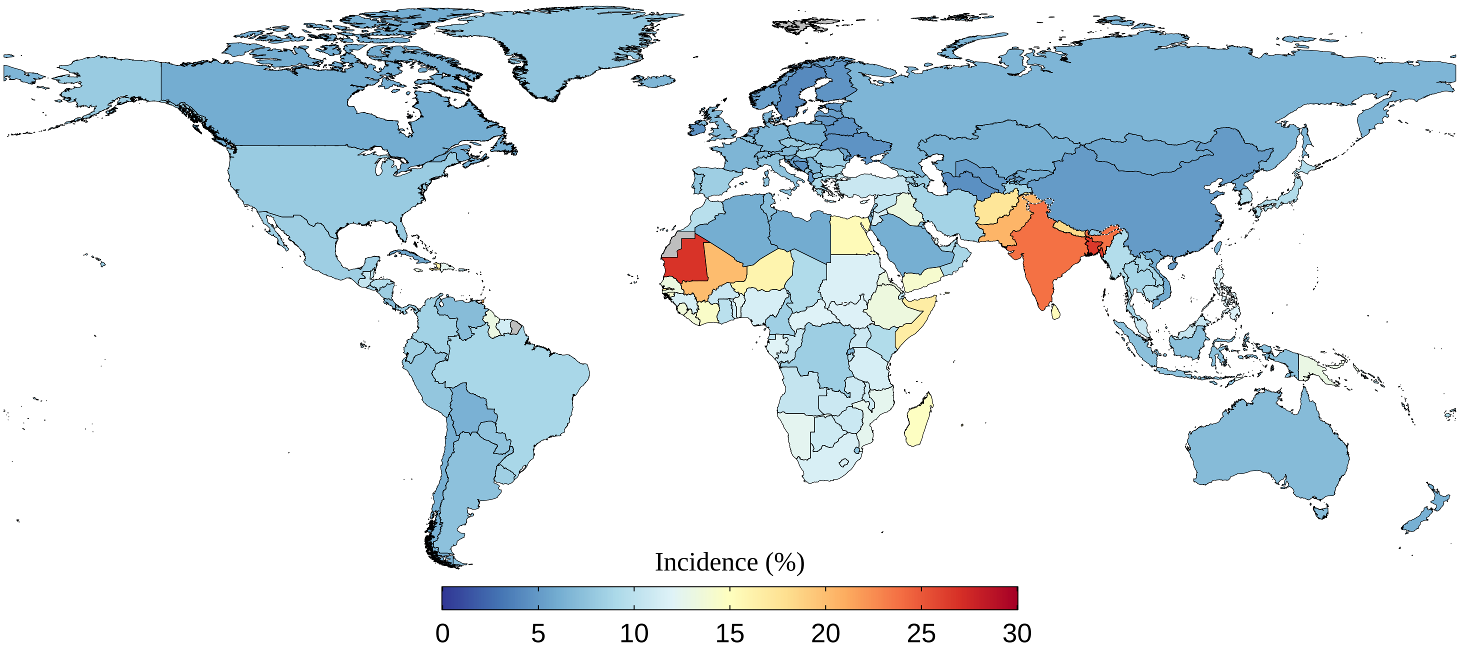

Low birthweight incidence (%), both sexes, birth, 2021

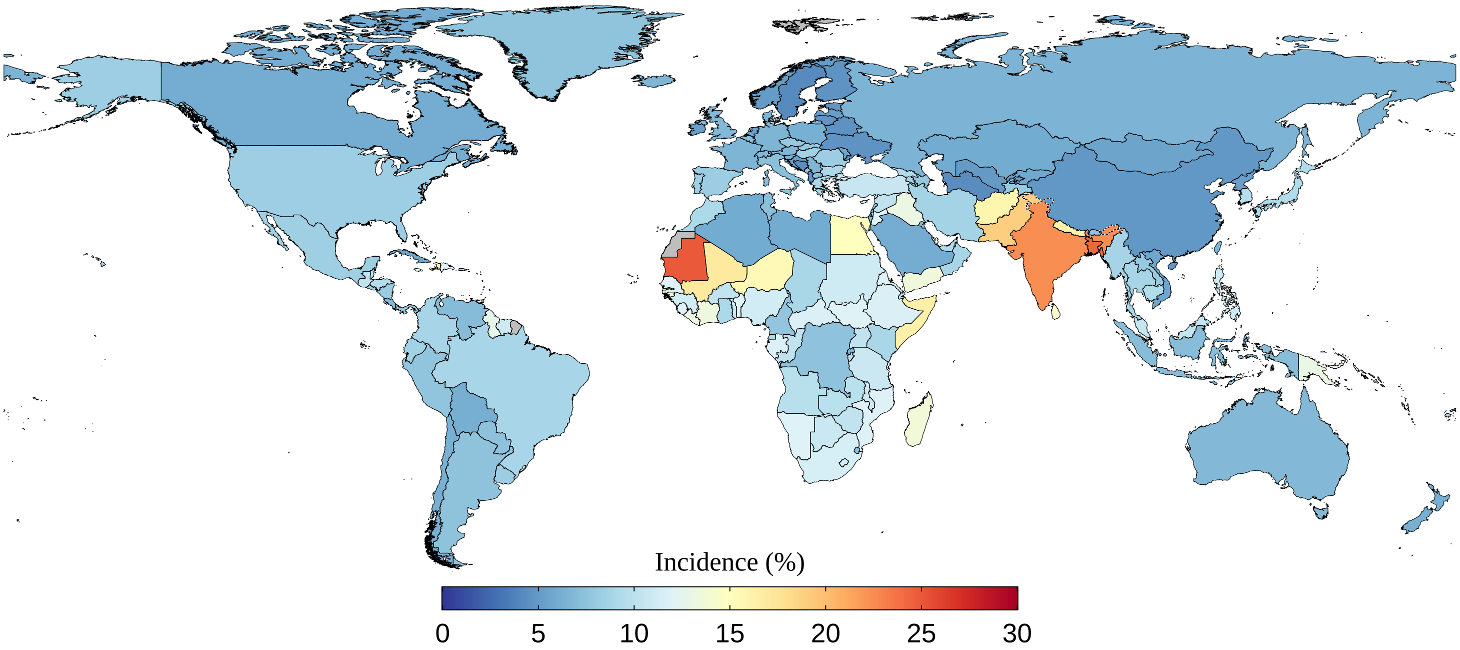

Low birthweight incidence (%), both sexes, birth, 2030

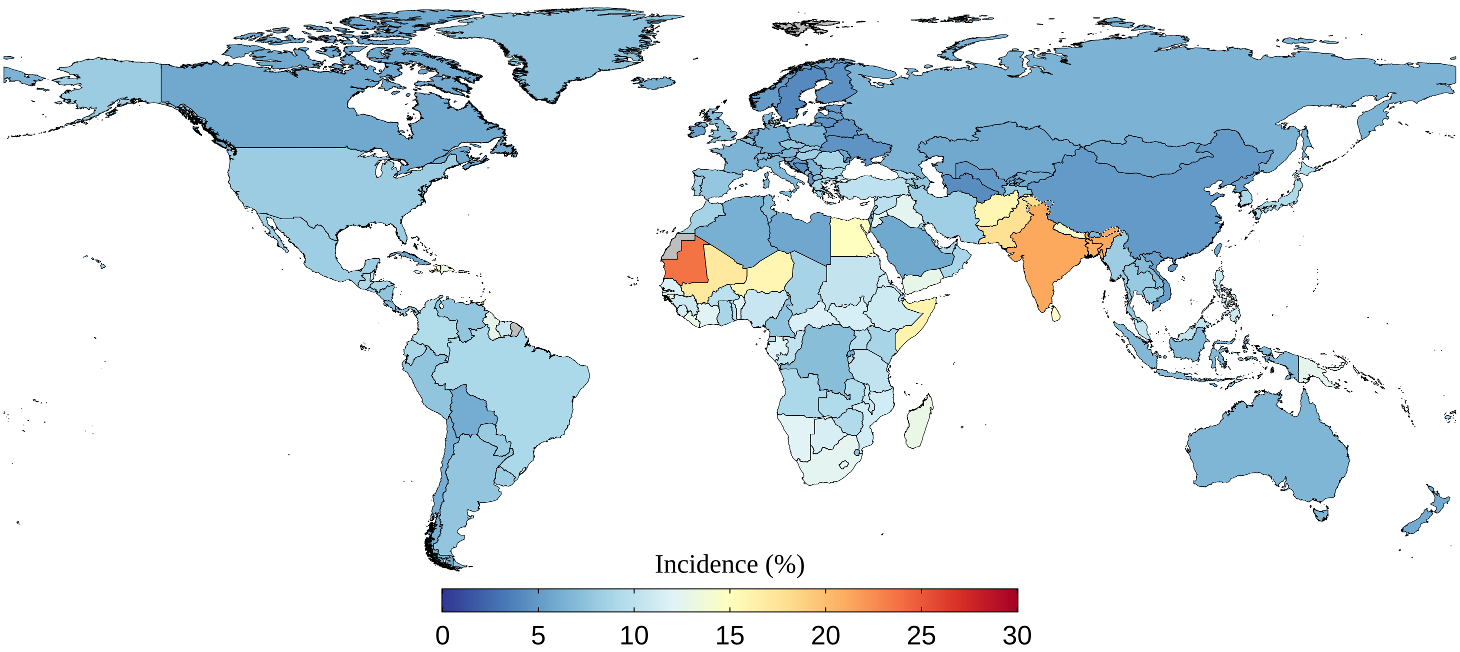

Figure S4. Global maps of indicator metrics, 2012, 2021, 2030

Exclusive breastfeeding prevalence (%), both sexes, <6 months, 2012

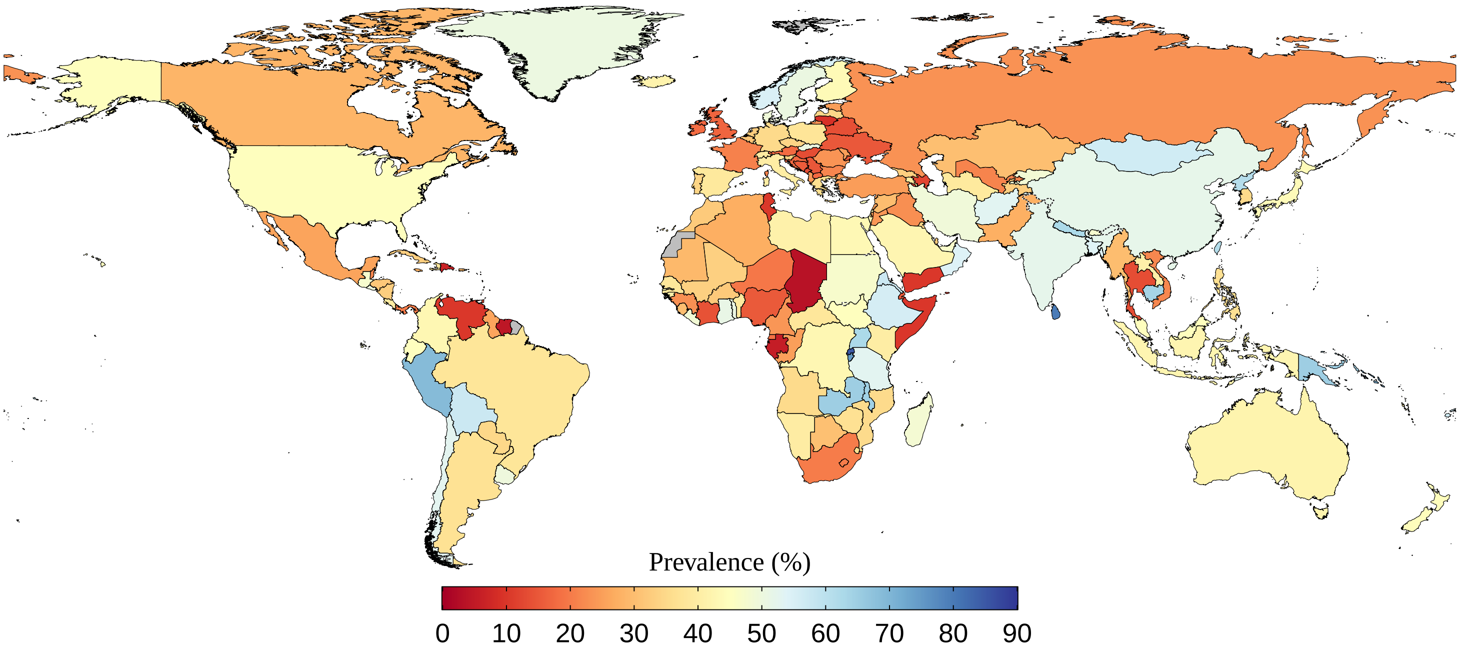

Exclusive breastfeeding prevalence (%), both sexes, <6 months, 2021

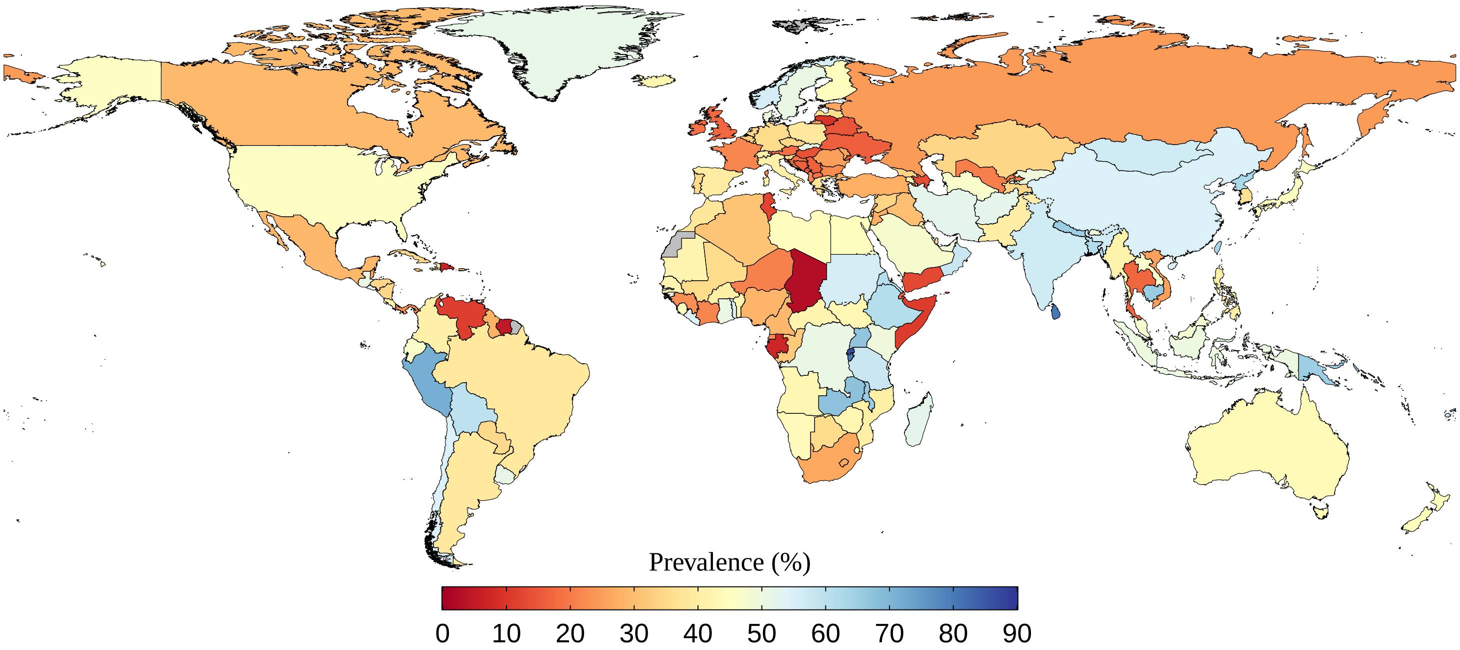

Exclusive breastfeeding prevalence (%), both sexes, <6 months, 2030

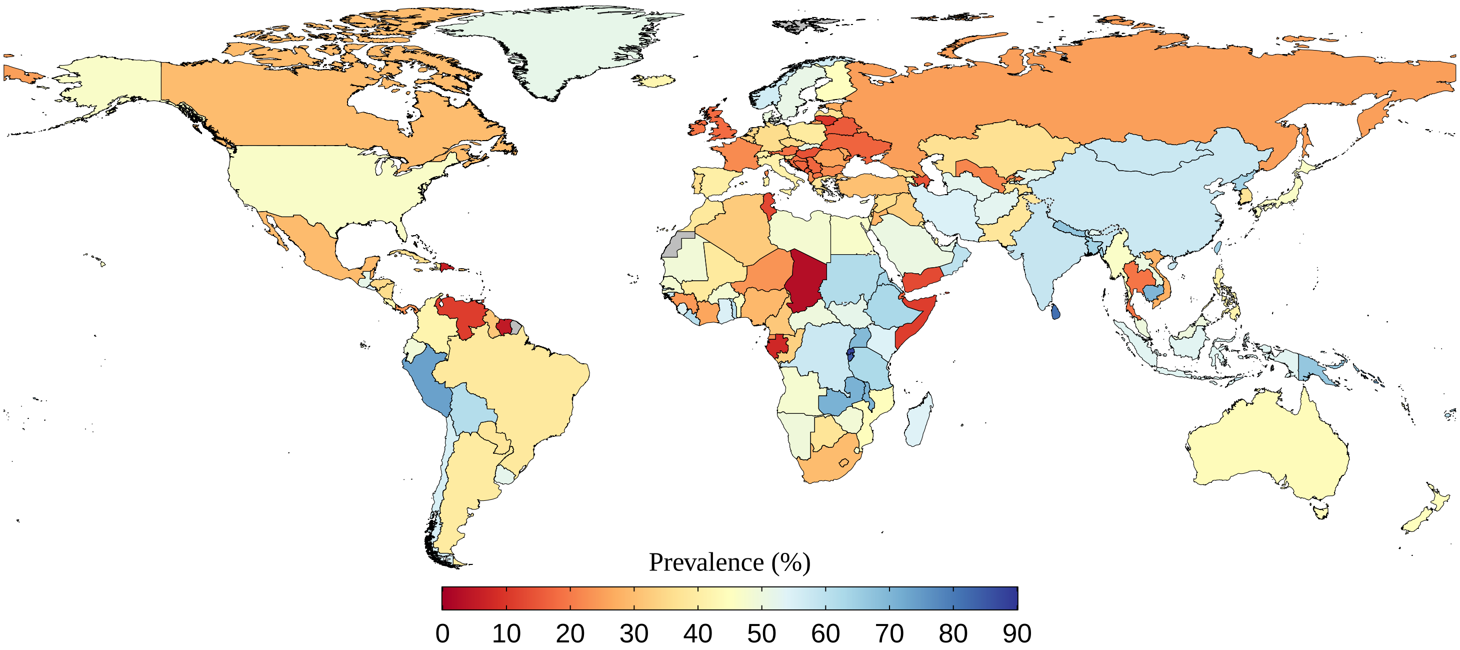

Figure S4. Global maps of indicator metrics, 2012, 2021, 2030

Stunting prevalence (%), both sexes, <5 years, 2012

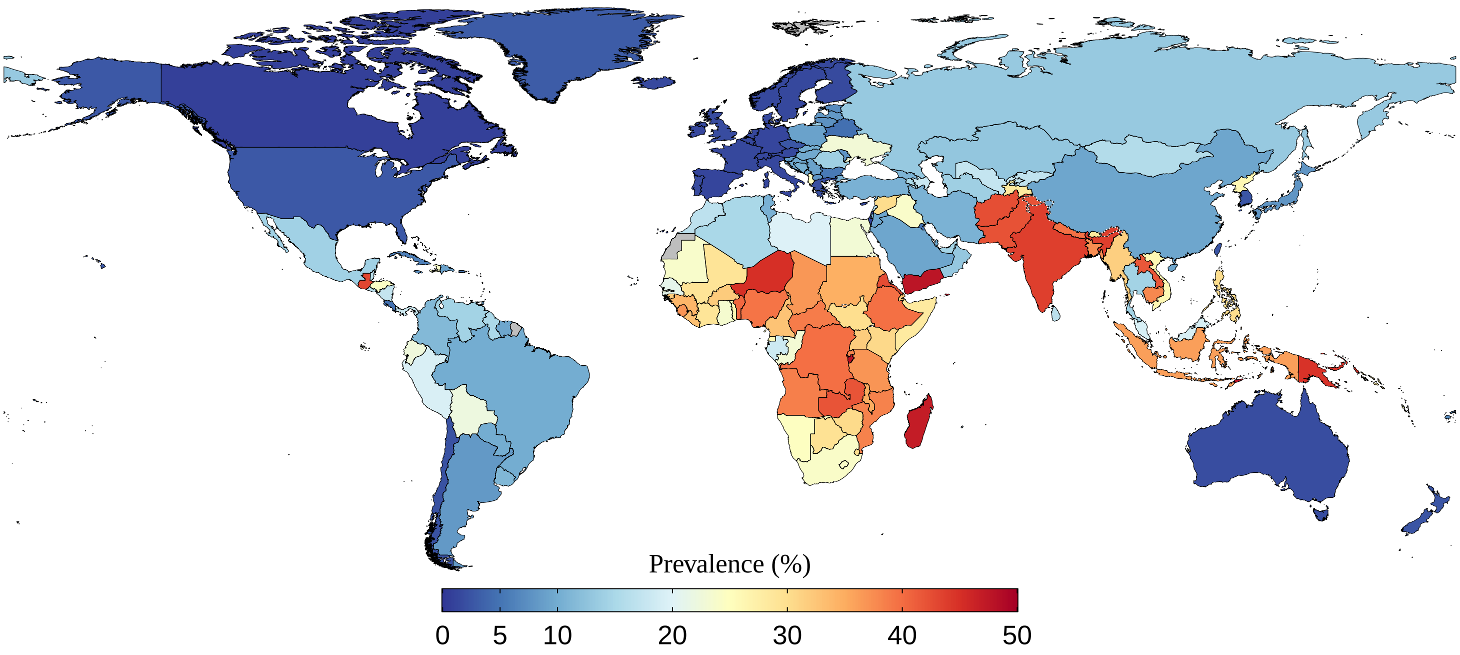

Stunting prevalence (%), both sexes, <5 years, 2021

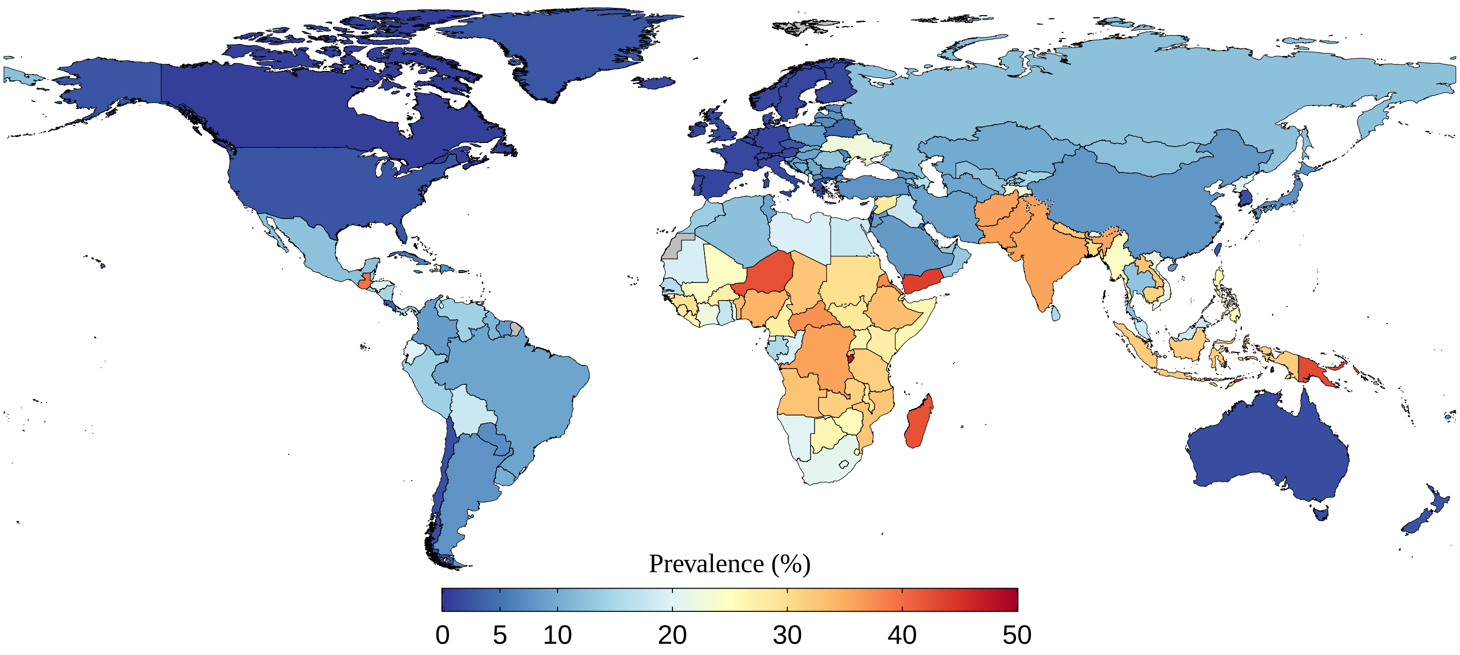

Stunting prevalence (%), both sexes, <5 years, 2030

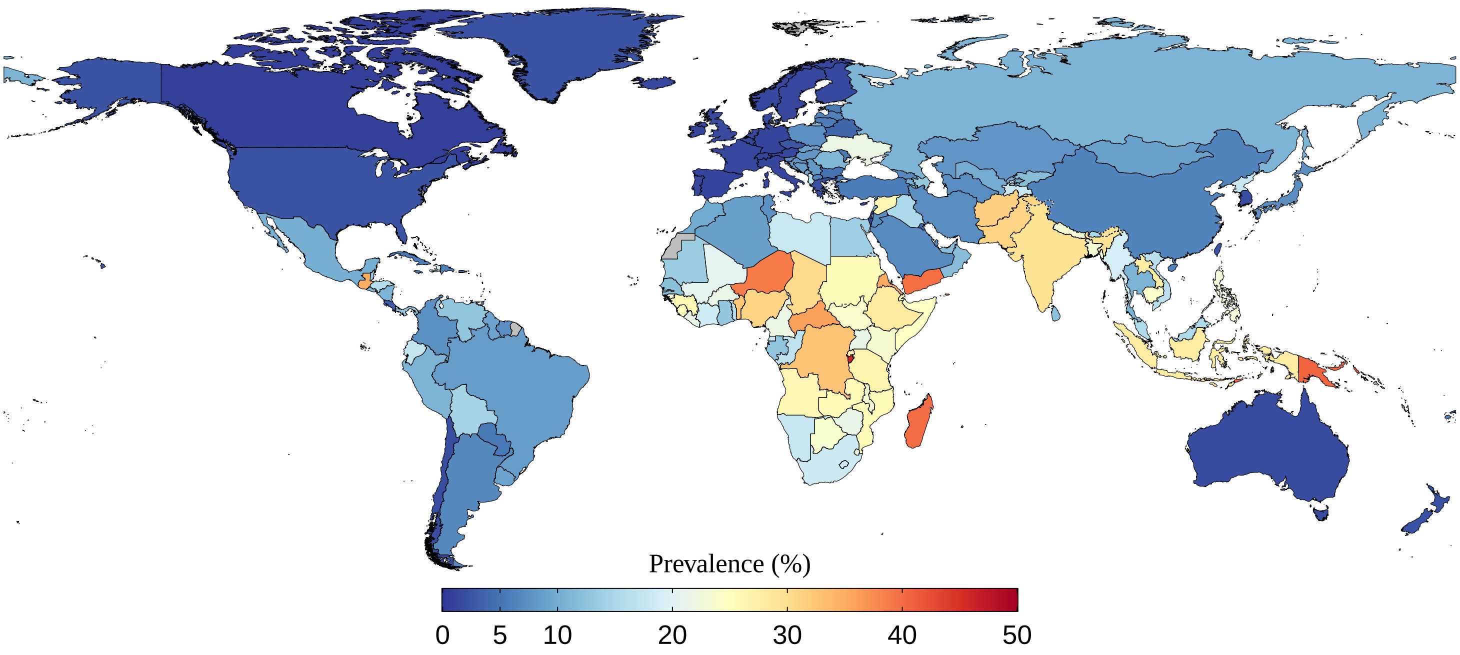

Figure S4. Global maps of indicator metrics, 2012, 2021, 2030

Wasting prevalence (%), both sexes, < 5 years, 2012

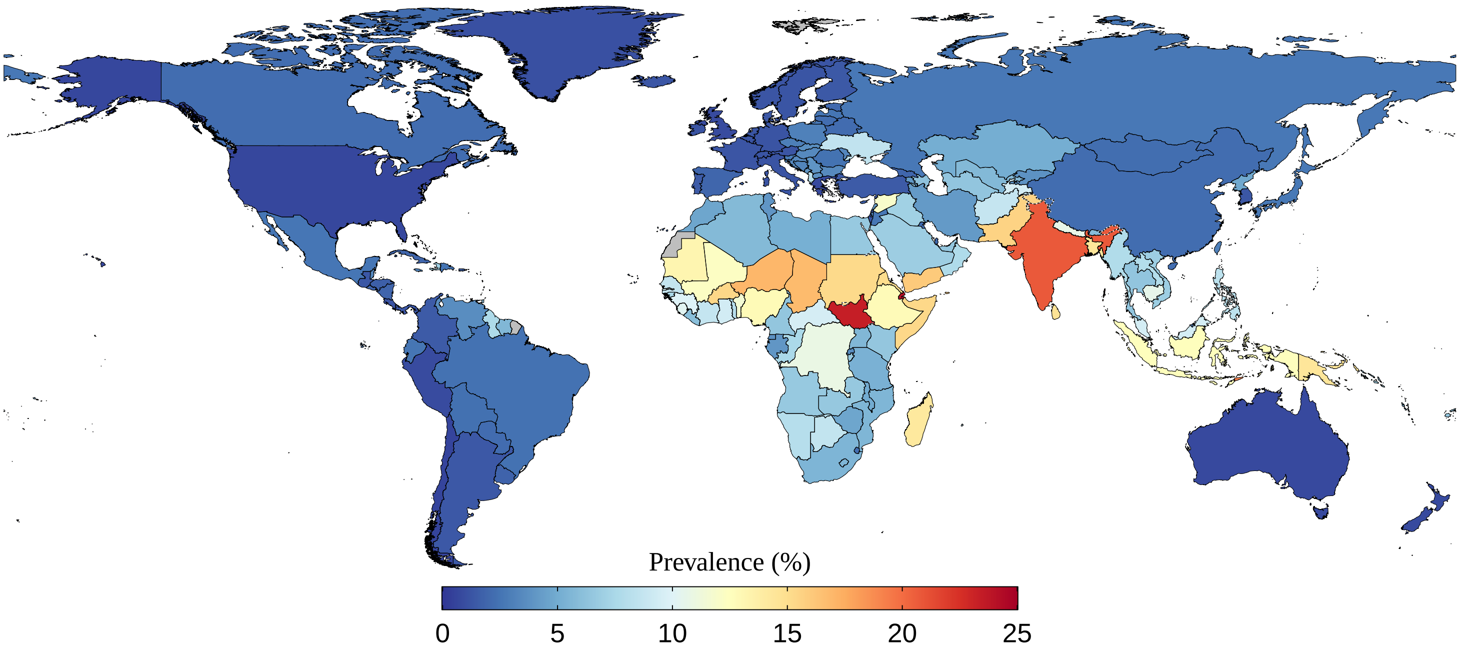

Wasting prevalence (%), both sexes, < 5 years, 2021

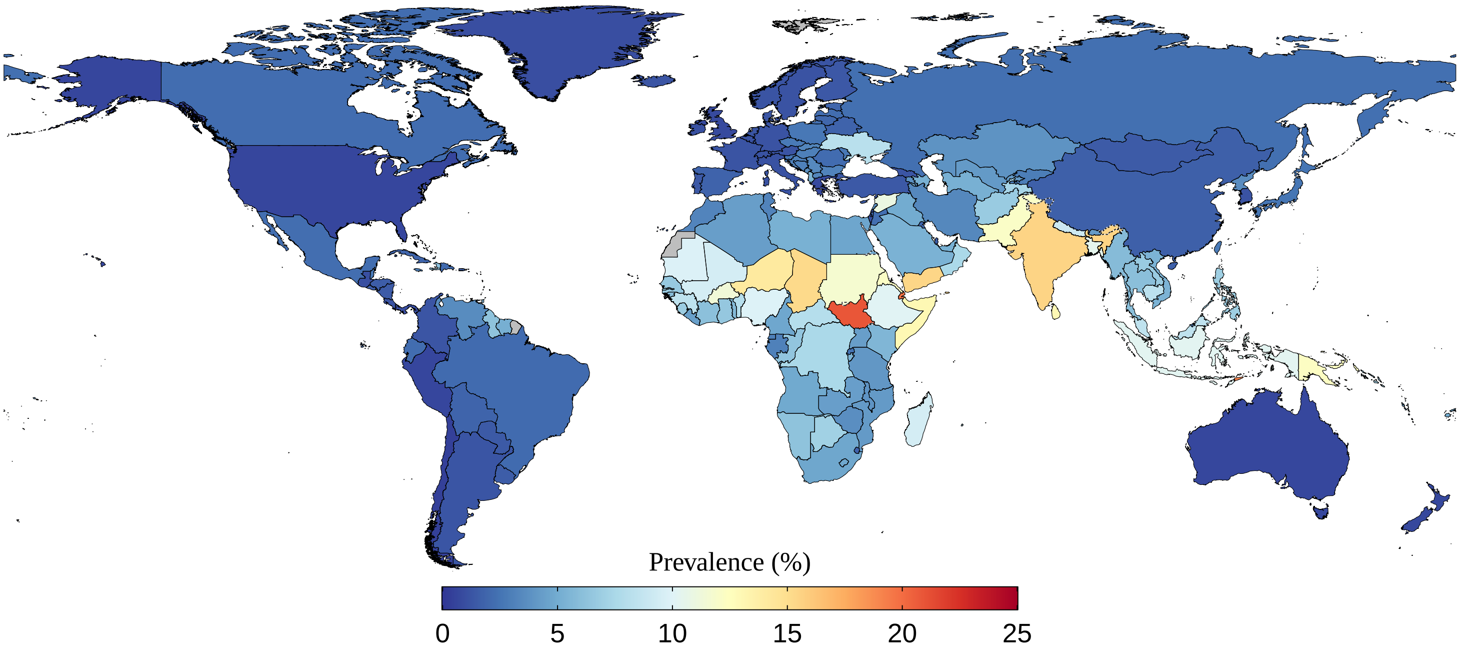

Wasting prevalence (%), both sexes, < 5 years, 2030

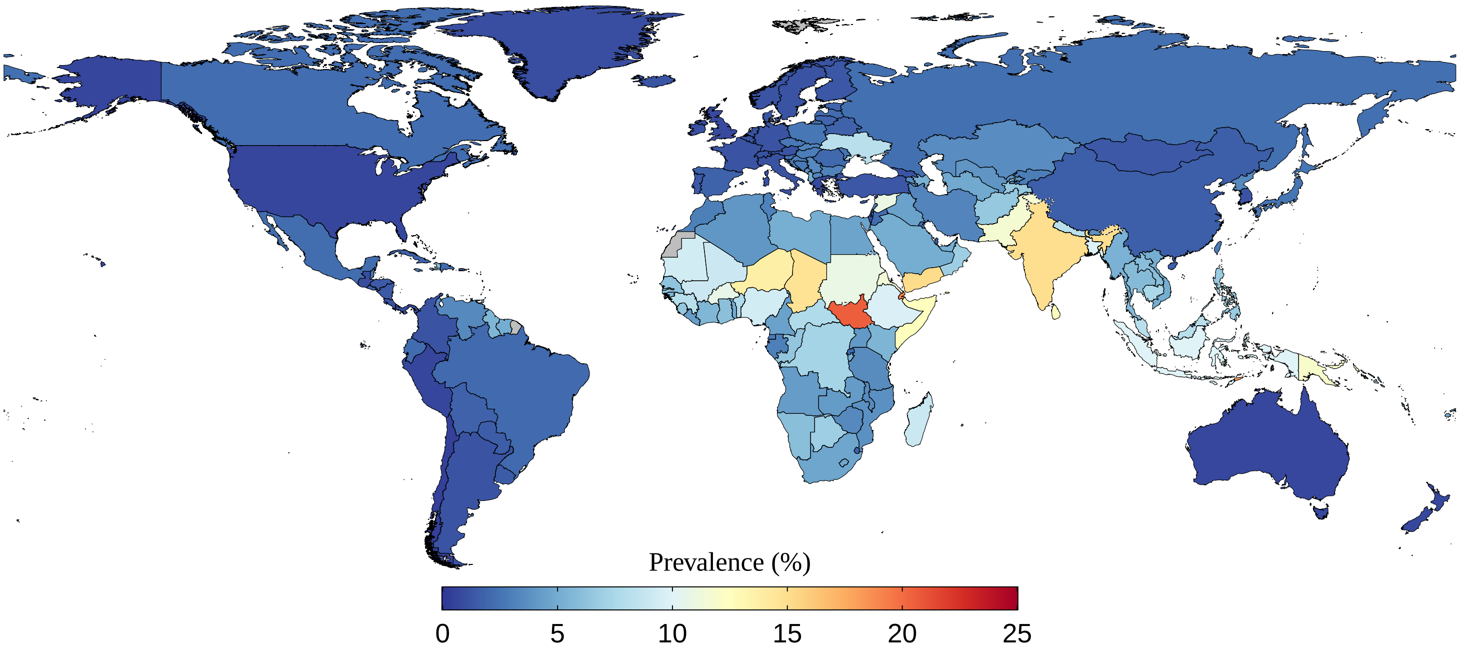

Figure S4. Global maps of indicator metrics, 2012, 2021, 2030

Overweight prevalence (%), both sexes, 2− 4 years, 2012

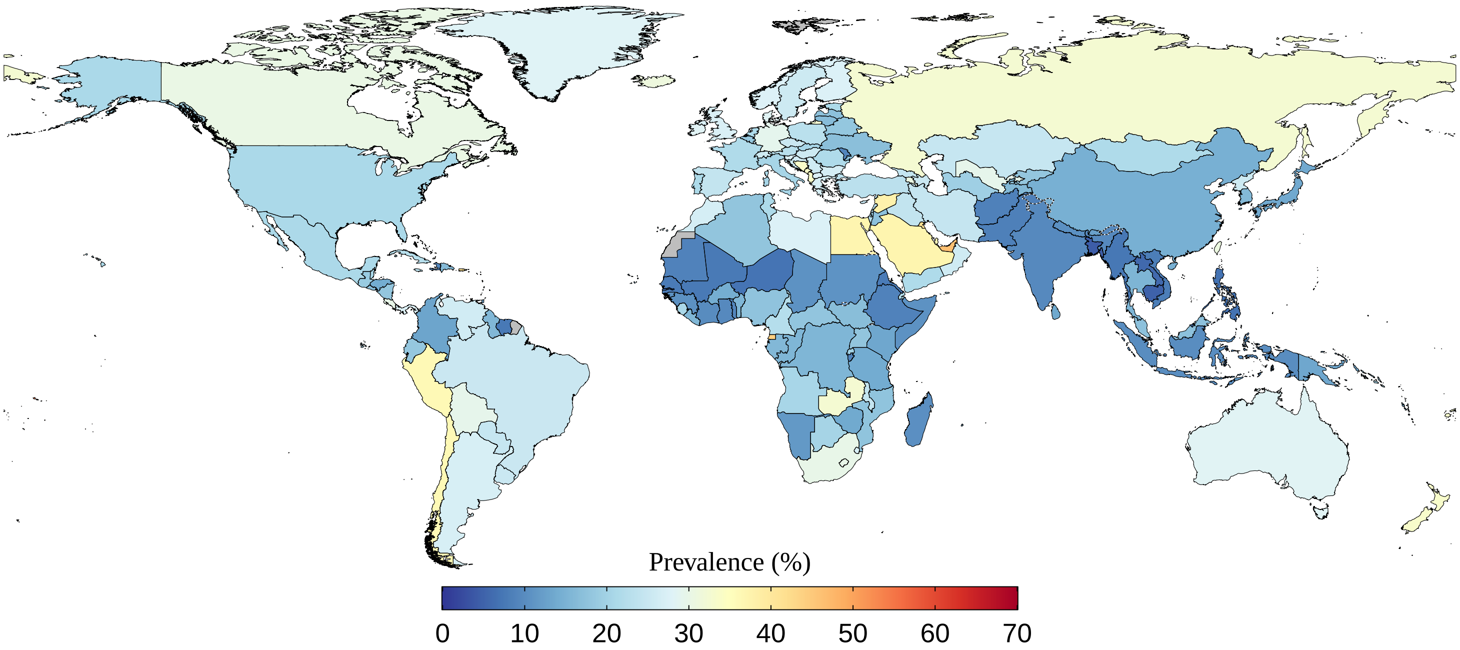

Overweight prevalence (%), both sexes, 2− 4 years, 2021

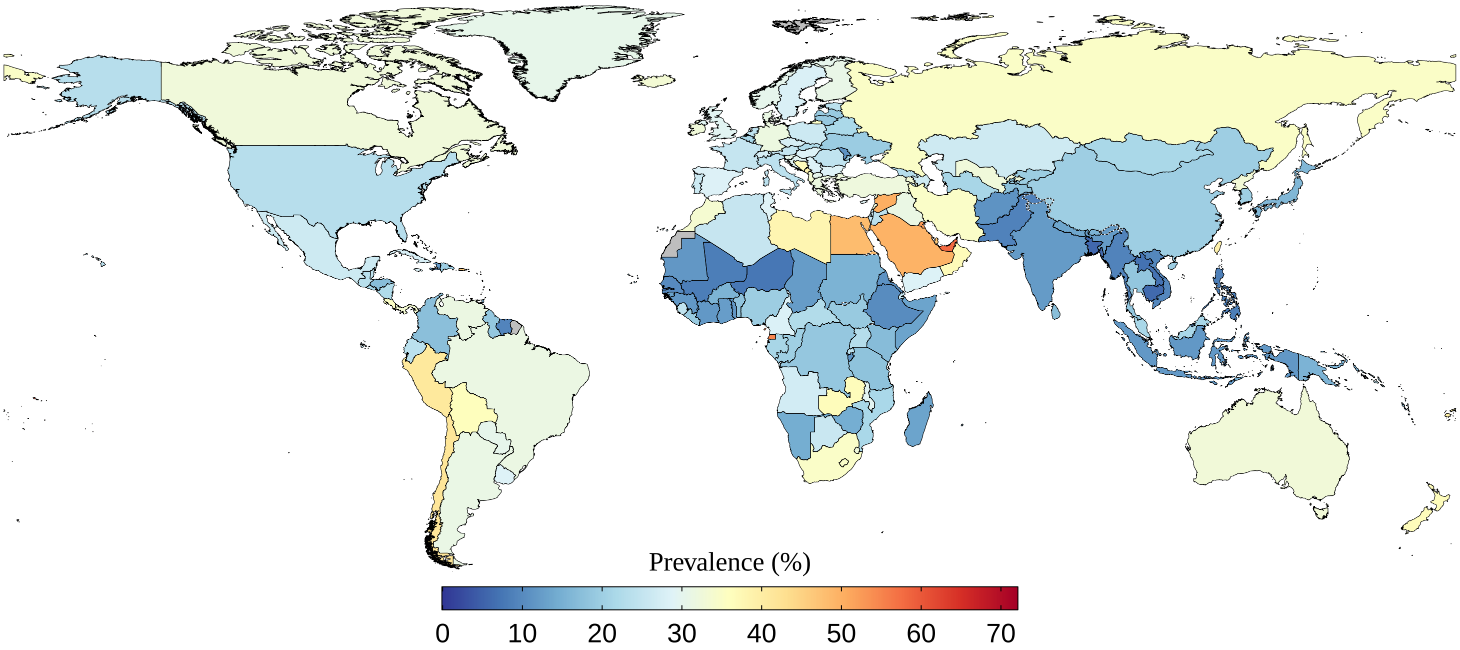

Overweight prevalence (%), both sexes, 2− 4 years, 2030

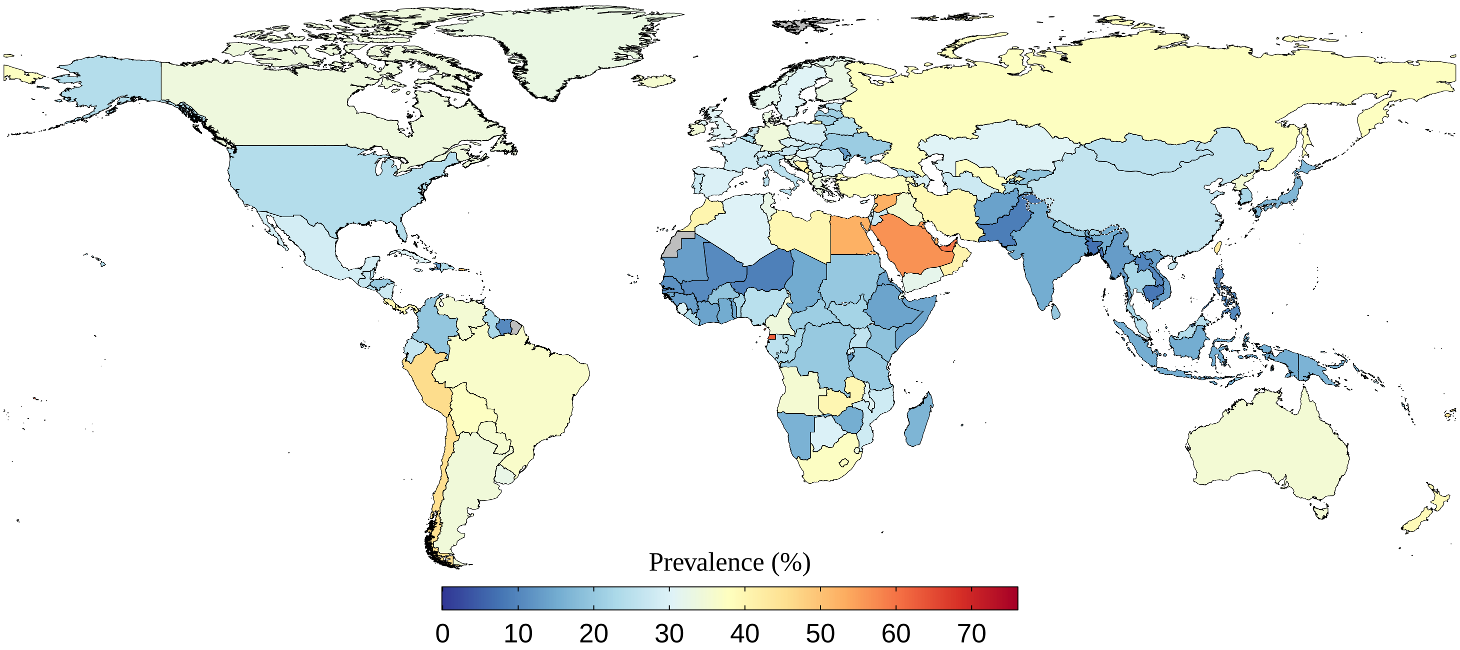

Figure S4. Global maps of indicator metrics, 2012, 2021, 2030

Anemia prevalence (%), females, 15−49 years, 2012

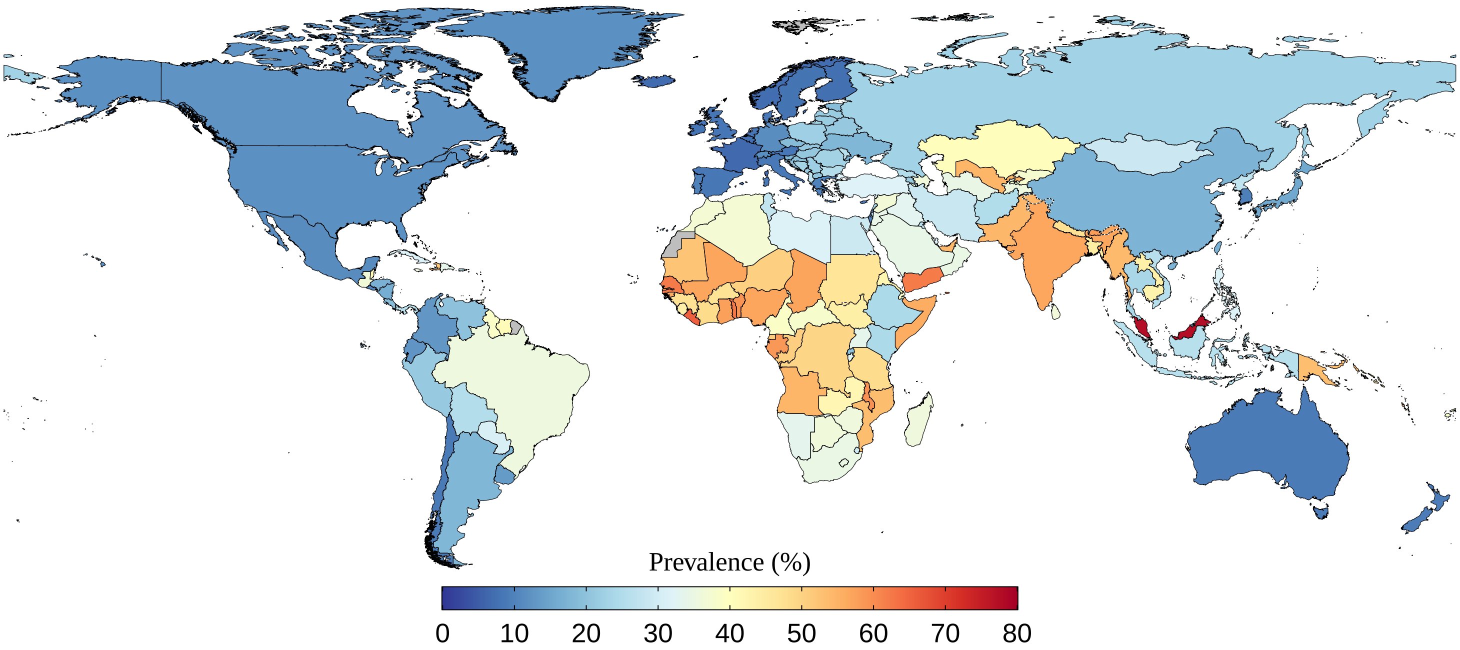

Anemia prevalence (%), females, 15−49 years, 2021

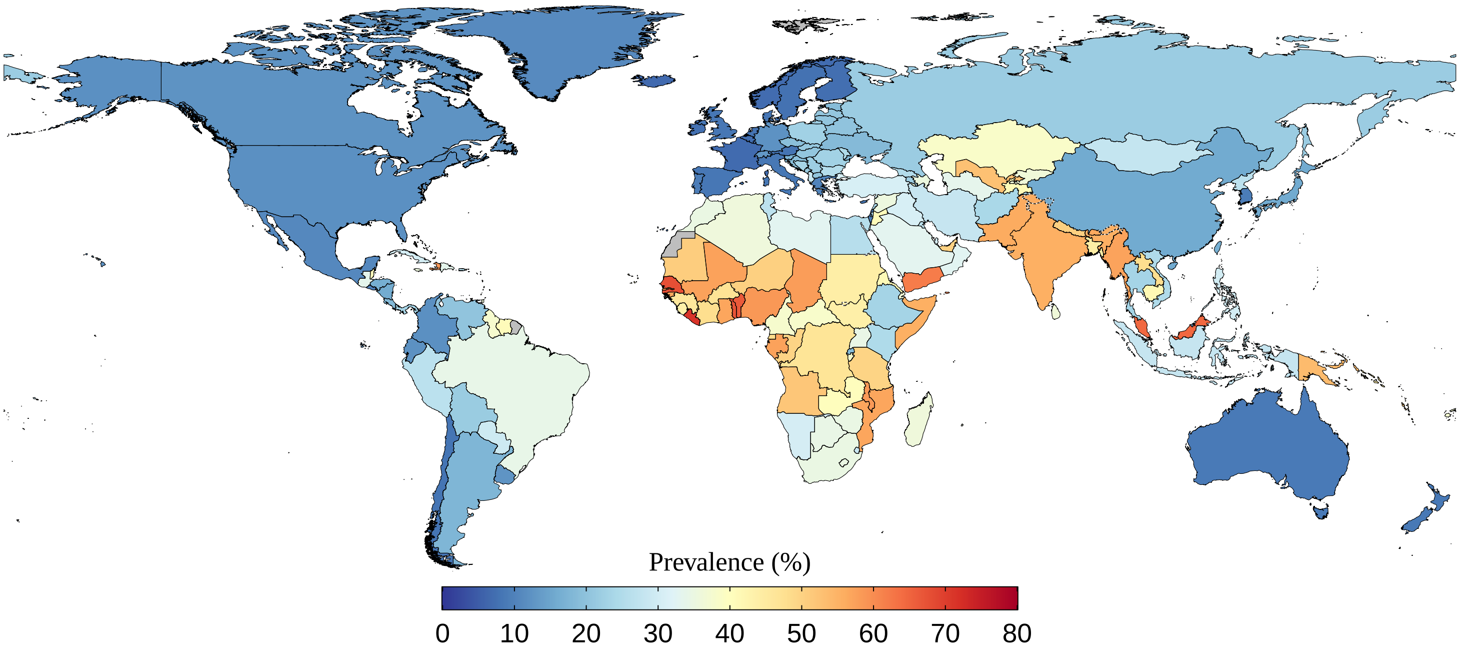

Anemia prevalence (%), females, 15−49 years, 2030

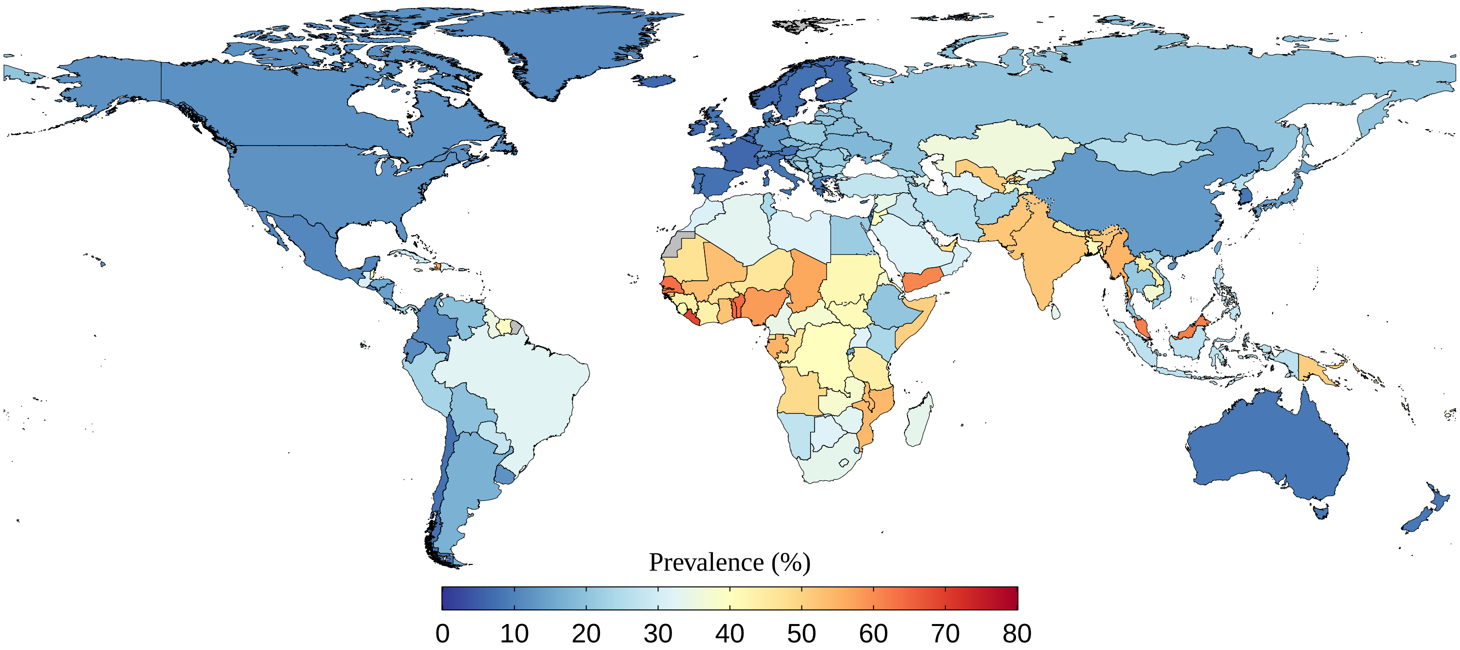

Figure S5. Co−evolution of GNT indicator prevalence in Central Asia, 2012 to 2021

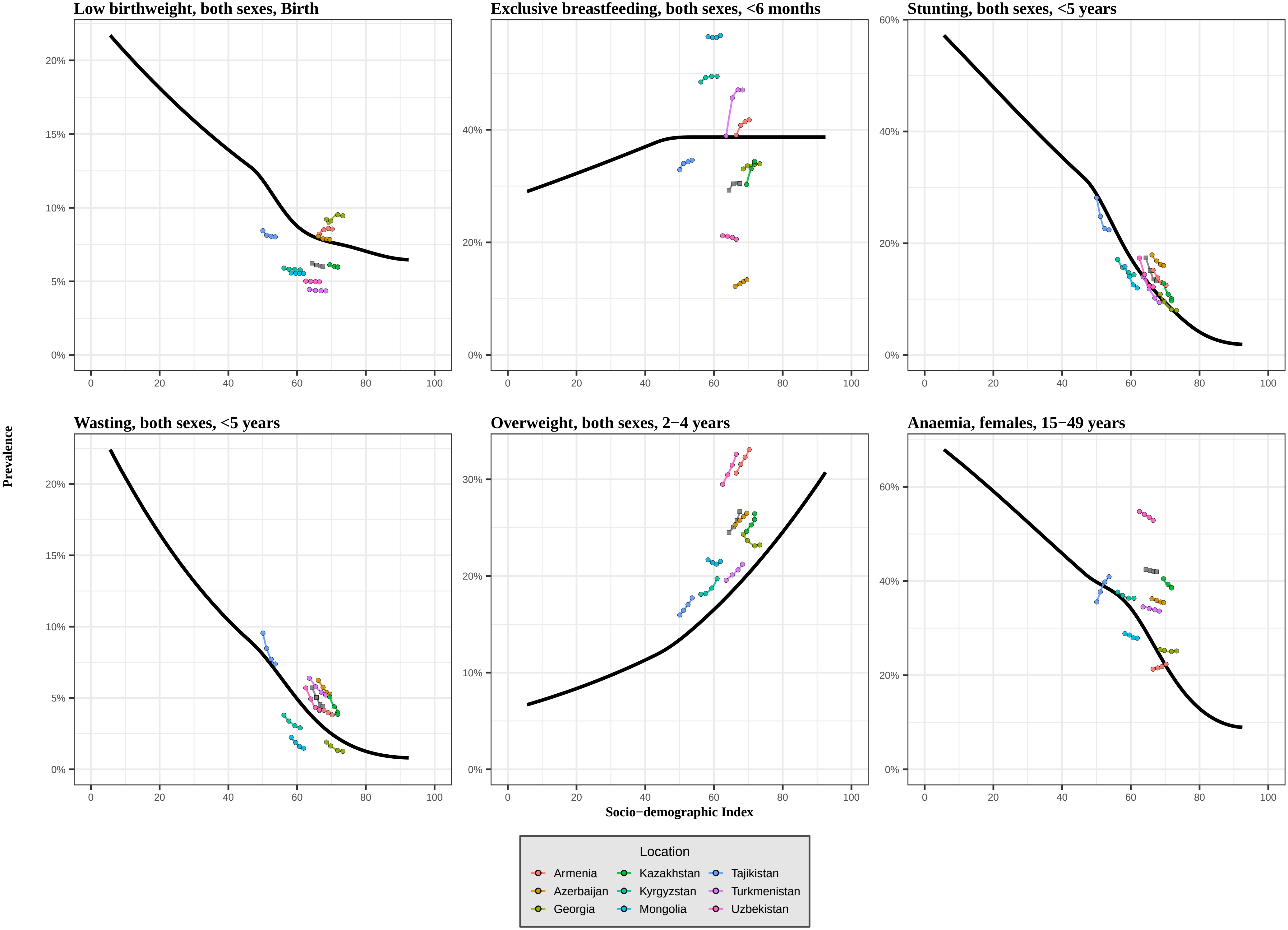

The expected prevalence values of each indicator based on the Socio−demographic Index (SDI) are represented by the solid black lines. Observed values of the indicator are shown for each country in Central Asia, with the region's values shown in grey. Points are shown every 3 years from 2012 to 2021.

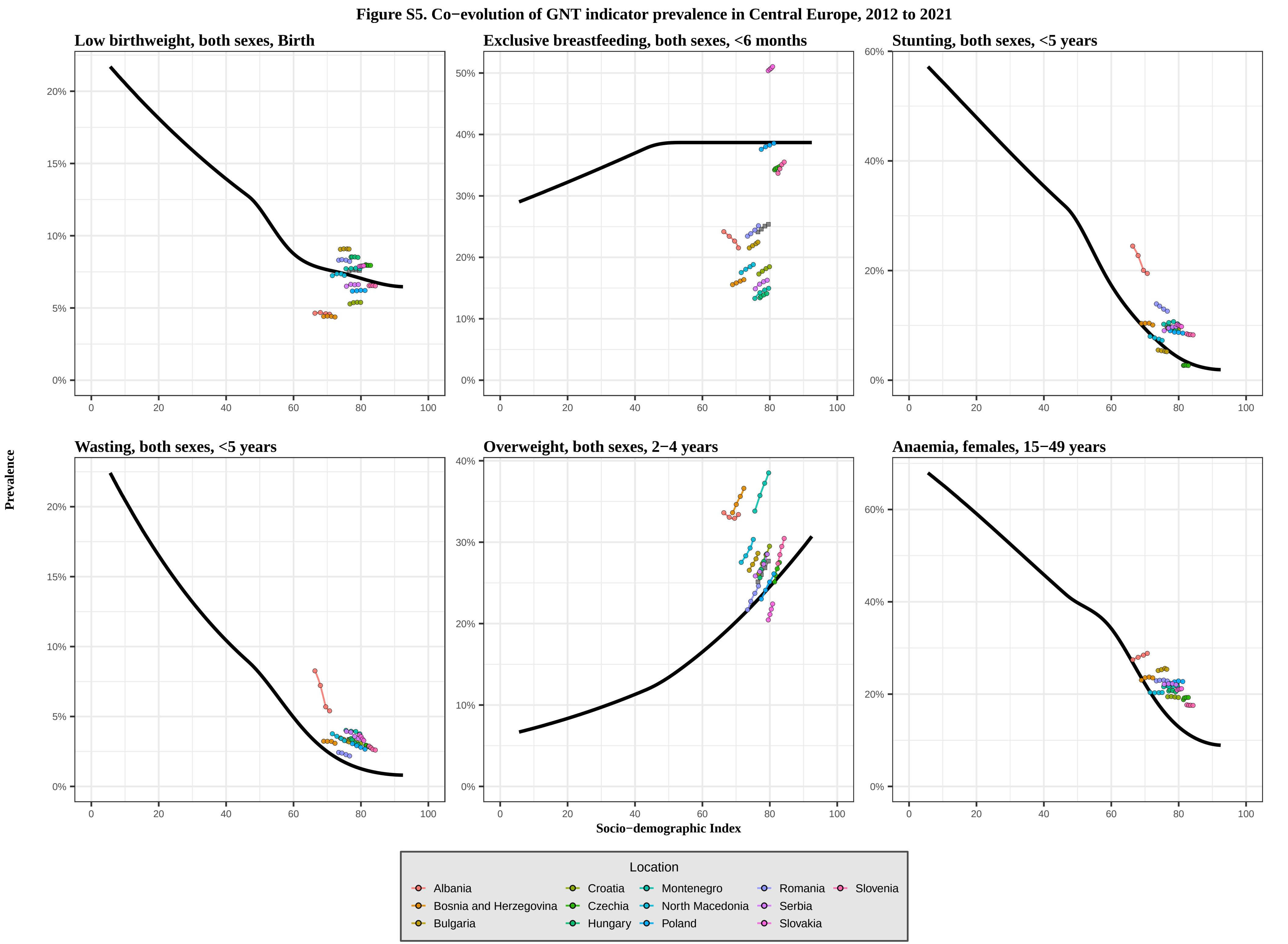

The expected prevalence values of each indicator based on the Socio−demographic Index (SDI) are represented by the solid black lines. Observed values of the indicator are shown for each country in Central Europe, with the region's values shown in grey. Points are shown every 3 years from 2012 to 2021.

Figure S5. Co−evolution of GNT indicator prevalence in Eastern Europe, 2012 to 2021

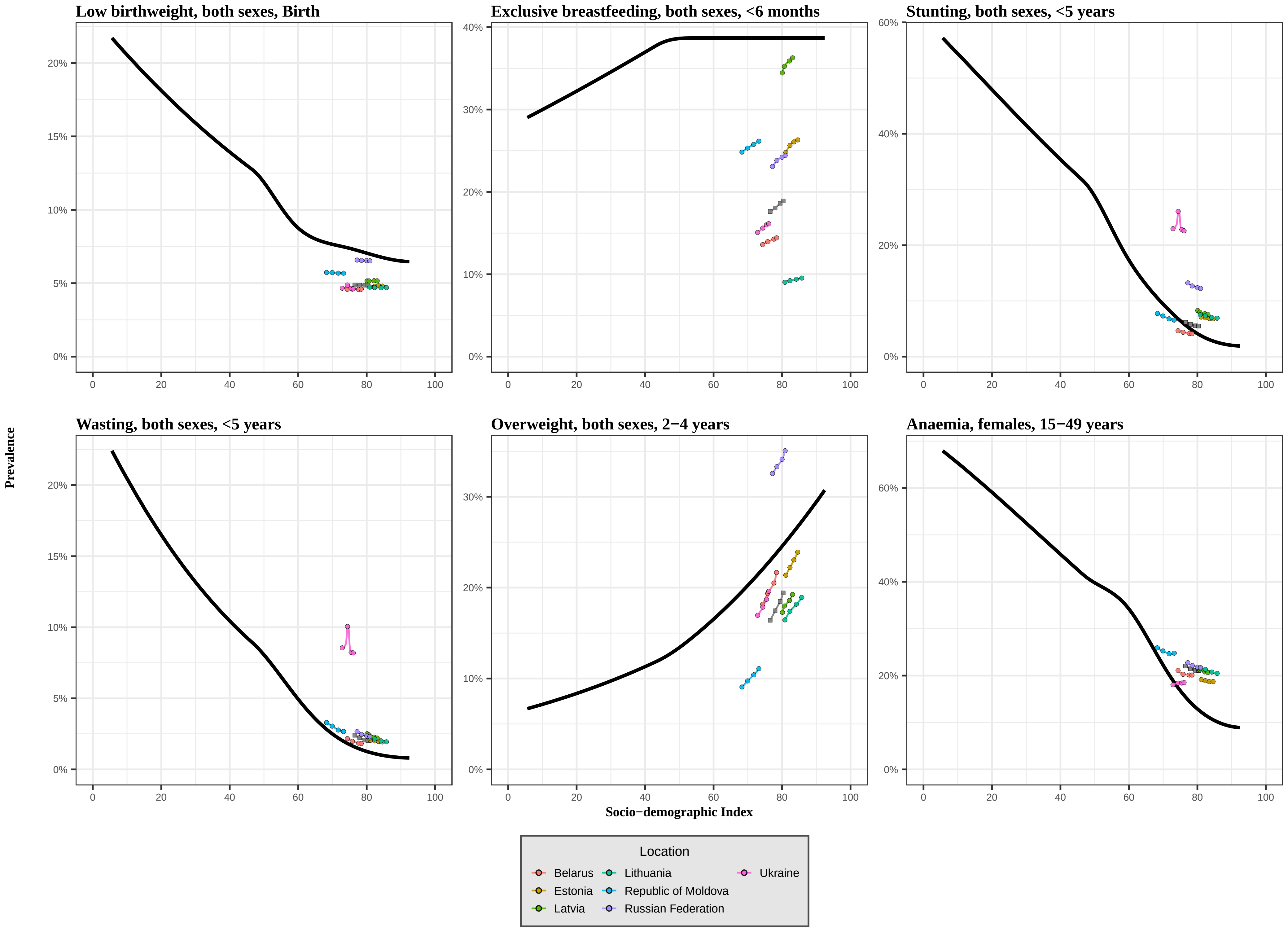

The expected prevalence values of each indicator based on the Socio−demographic Index (SDI) are represented by the solid black lines. Observed values of the indicator are shown for each country in Eastern Europe, with the region's values shown in grey. Points are shown every 3 years from 2012 to 2021.

Figure S5. Co−evolution of GNT indicator prevalence in Australasia, 2012 to 2021

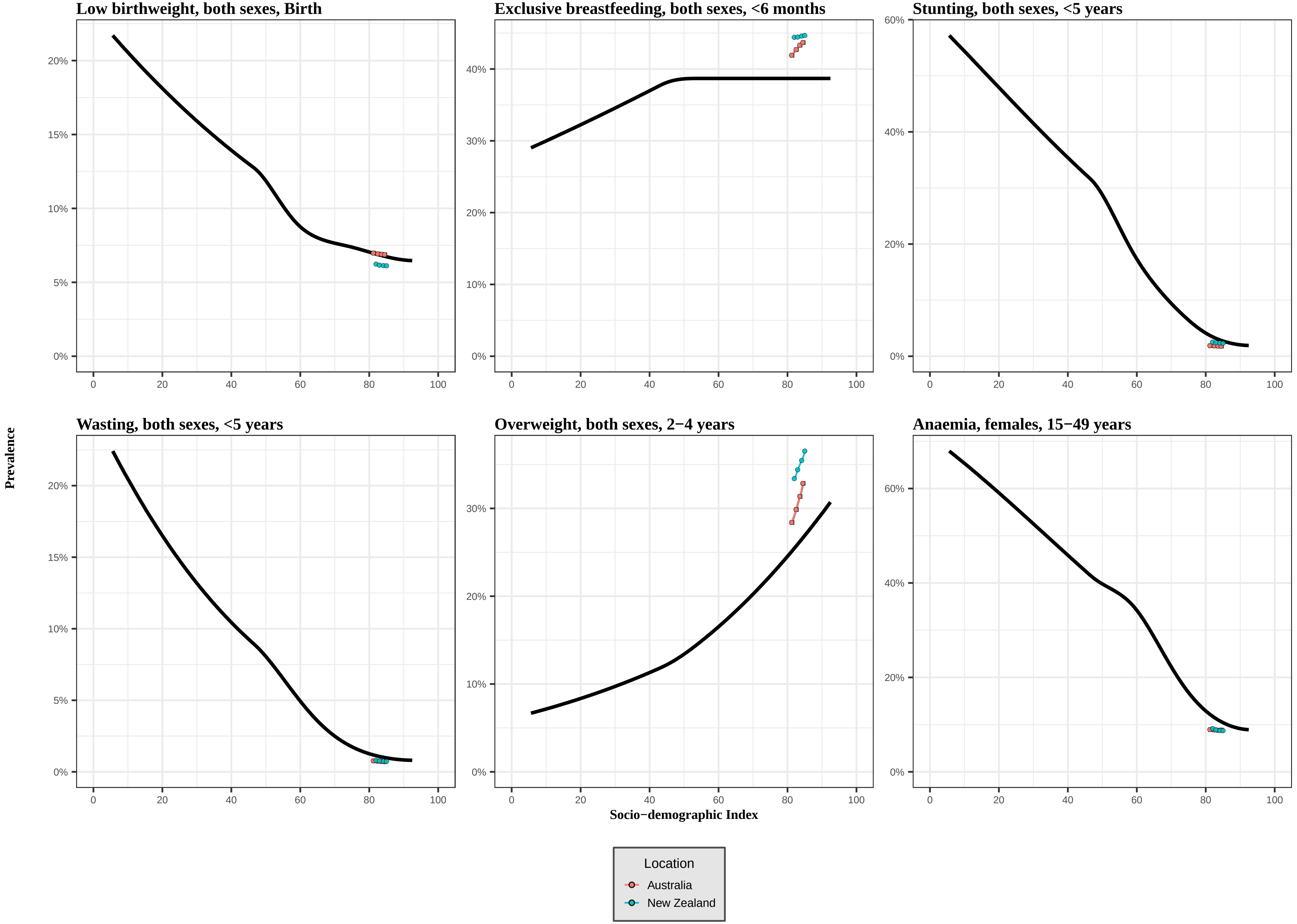

The expected prevalence values of each indicator based on the Socio−demographic Index (SDI) are represented by the solid black lines. Observed values of the indicator are shown for each country in Australasia, with the region's values shown in grey. Points are shown every 3 years from 2012 to 2021.

Figure S5. Co−evolution of GNT indicator prevalence in High−income Asia Pacific, 2012 to 2021

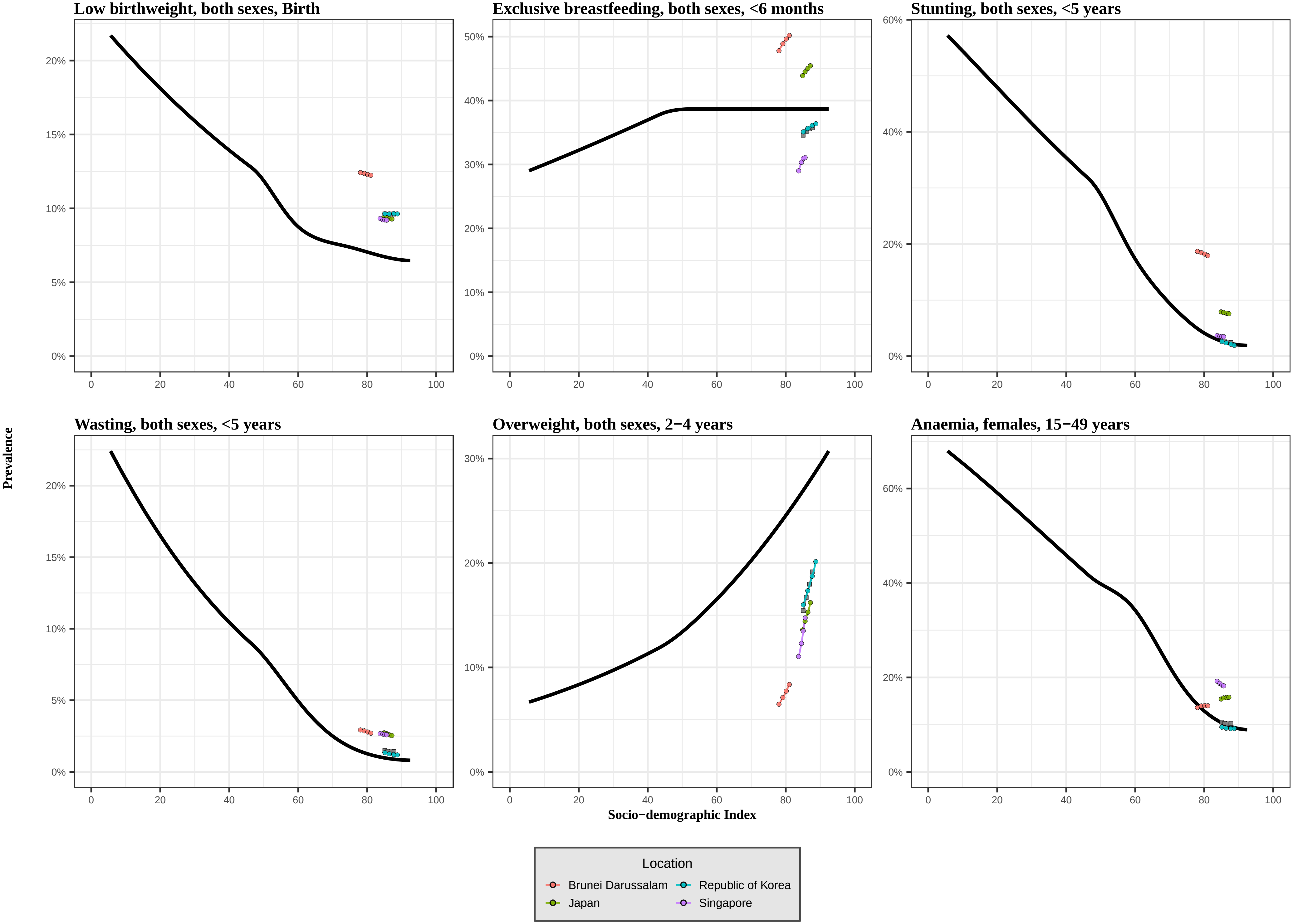

The expected prevalence values of each indicator based on the Socio−demographic Index (SDI) are represented by the solid black lines. Observed values of the indicator are shown for each country in High−income Asia Pacific, with the region's values shown in grey. Points are shown every 3 years from 2012 to 2021.

Figure S5. Co−evolution of GNT indicator prevalence in High−income North America, 2012 to 2021

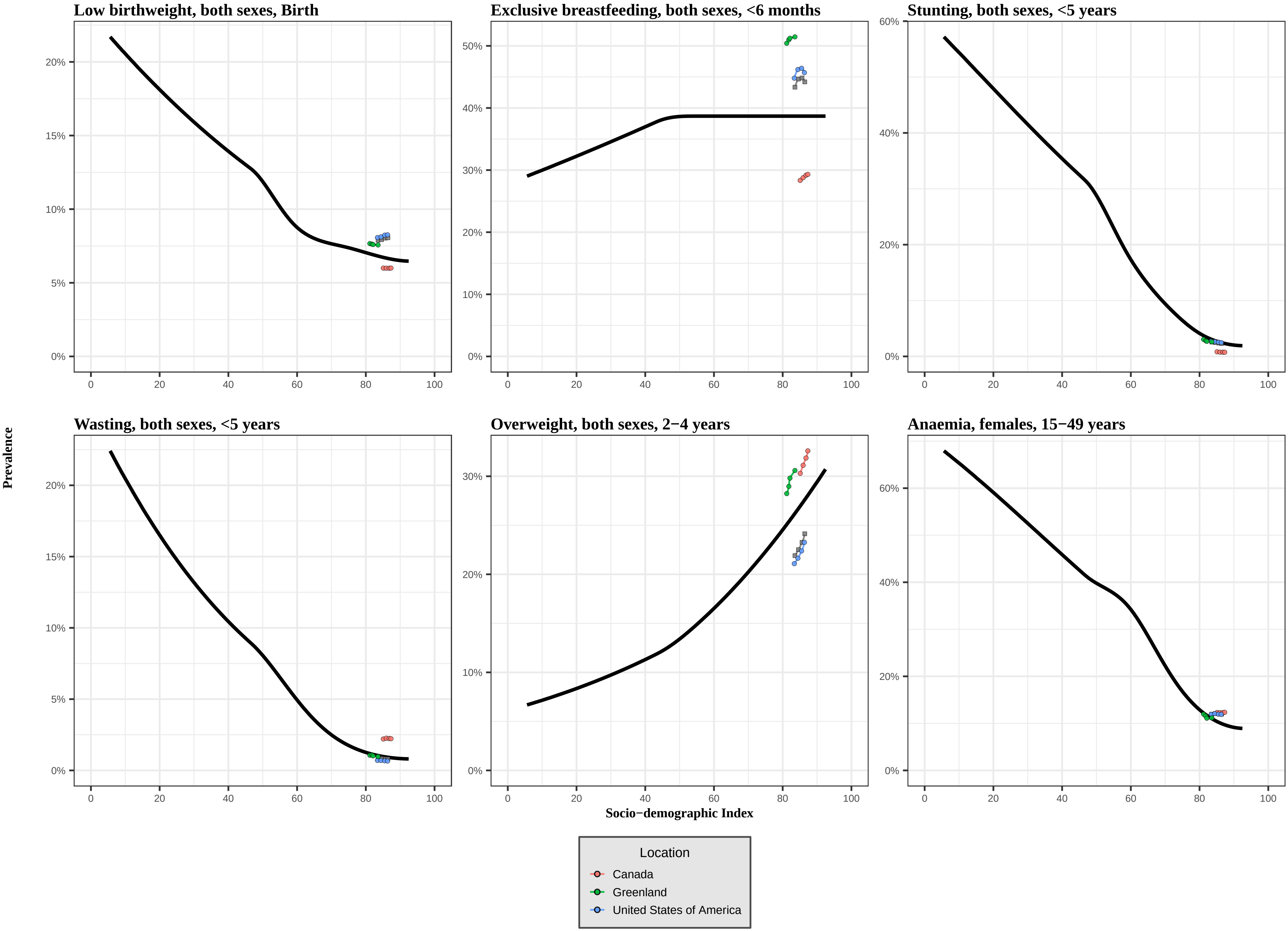

The expected prevalence values of each indicator based on the Socio−demographic Index (SDI) are represented by the solid black lines. Observed values of the indicator are shown for each country in High−income North America, with the region's values shown in grey. Points are shown every 3 years from 2012 to 2021.

Figure S5. Co−evolution of GNT indicator prevalence in Southern Latin America, 2012 to 2021

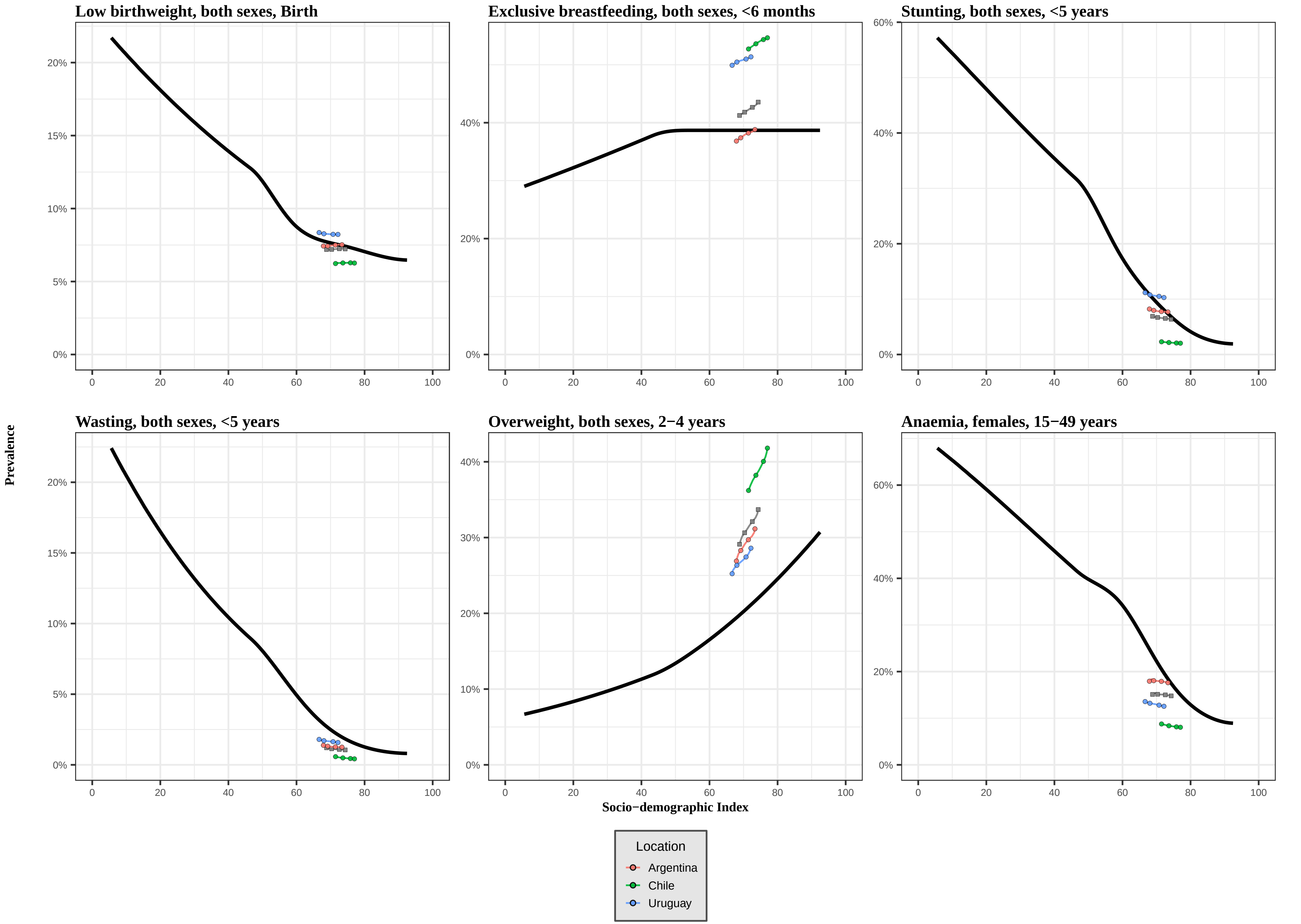

The expected prevalence values of each indicator based on the Socio−demographic Index (SDI) are represented by the solid black lines. Observed values of the indicator are shown for each country in Southern Latin America, with the region's values shown in grey. Points are shown every 3 years from 2012 to 2021.

Figure S5. Co−evolution of GNT indicator prevalence in Western Europe, 2012 to 2021

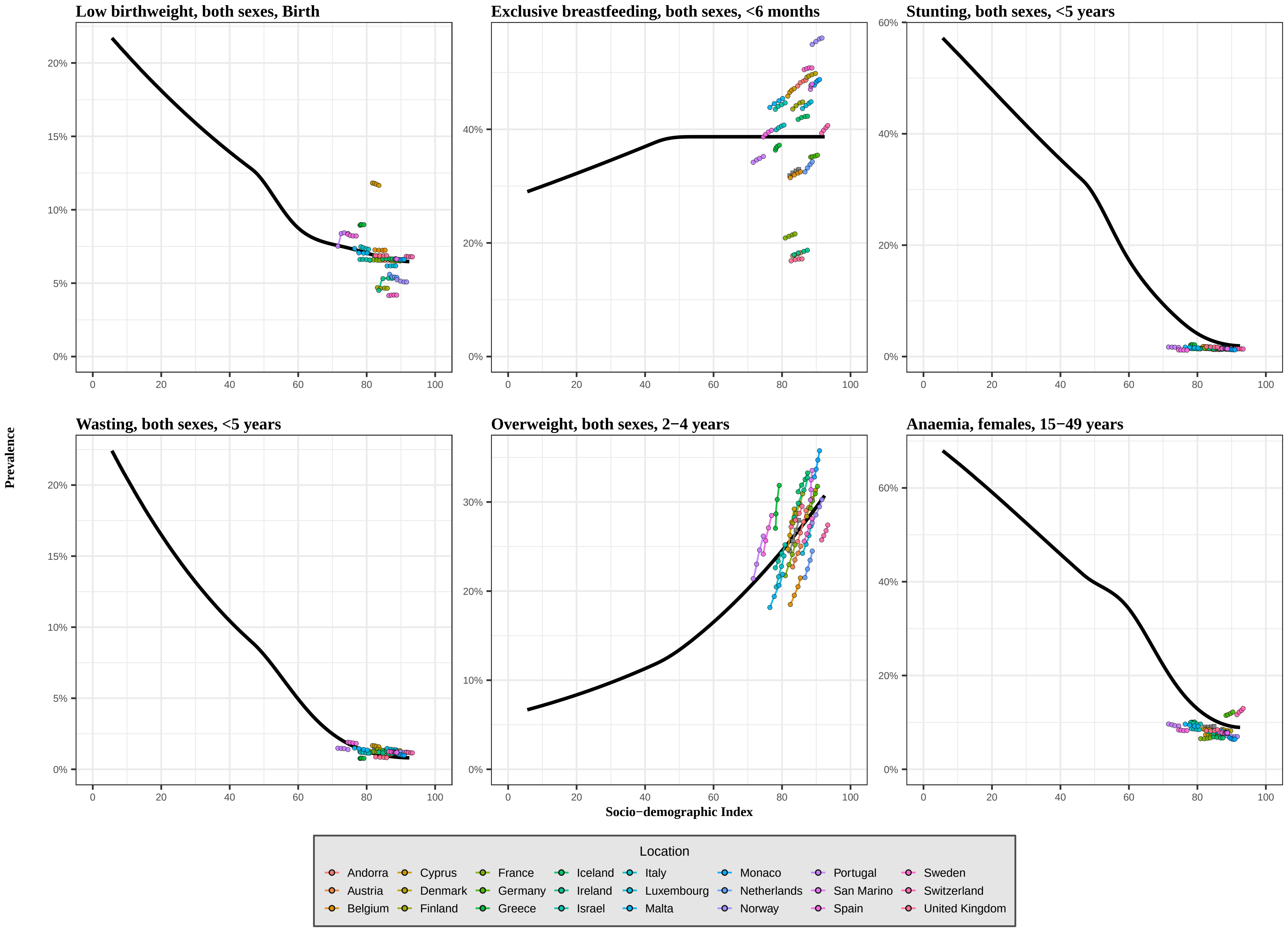

The expected prevalence values of each indicator based on the Socio−demographic Index (SDI) are represented by the solid black lines. Observed values of the indicator are shown for each country in Western Europe, with the region's values shown in grey. Points are shown every 3 years from 2012 to 2021.

Figure S5. Co−evolution of GNT indicator prevalence in Andean Latin America, 2012 to 2021

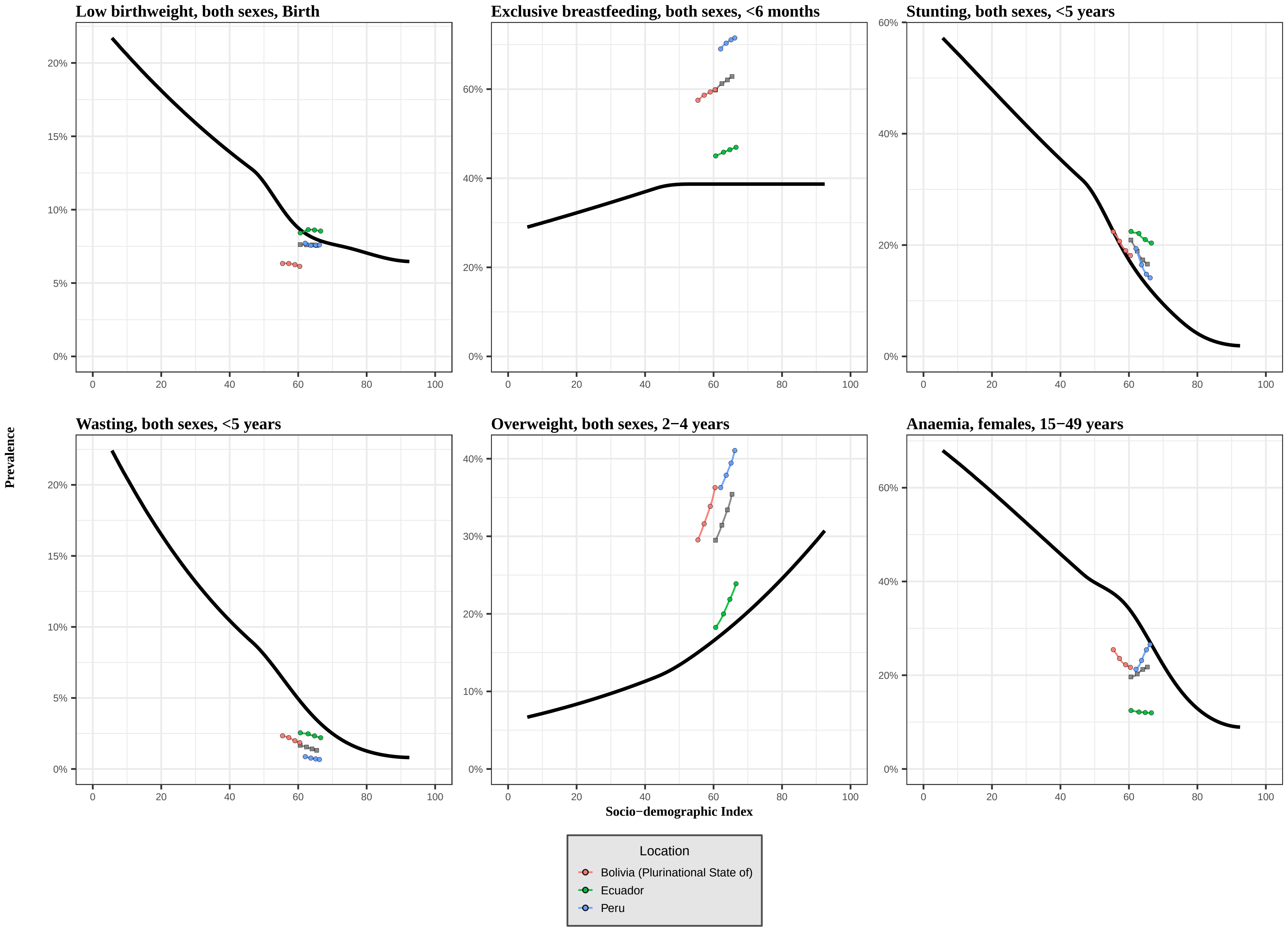

The expected prevalence values of each indicator based on the Socio−demographic Index (SDI) are represented by the solid black lines. Observed values of the indicator are shown for each country in Andean Latin America, with the region's values shown in grey. Points are shown every 3 years from 2012 to 2021.

Figure S5. Co−evolution of GNT indicator prevalence in Caribbean, 2012 to 2021

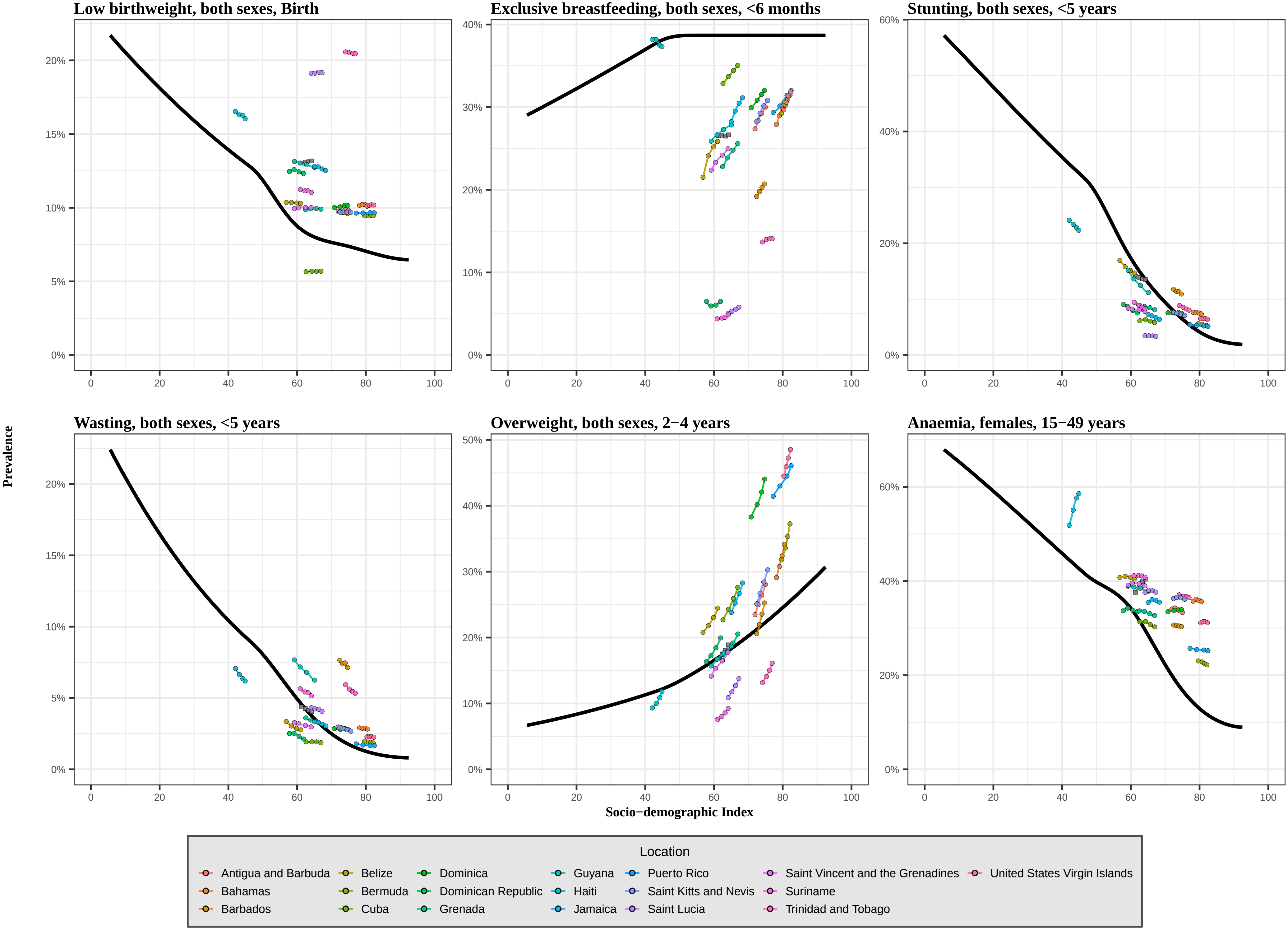

The expected prevalence values of each indicator based on the Socio−demographic Index (SDI) are represented by the solid black lines. Observed values of the indicator are shown for each country in Caribbean, with the region's values shown in grey. Points are shown every 3 years from 2012 to 2021.

Figure S5. Co−evolution of GNT indicator prevalence in Central Latin America, 2012 to 2021

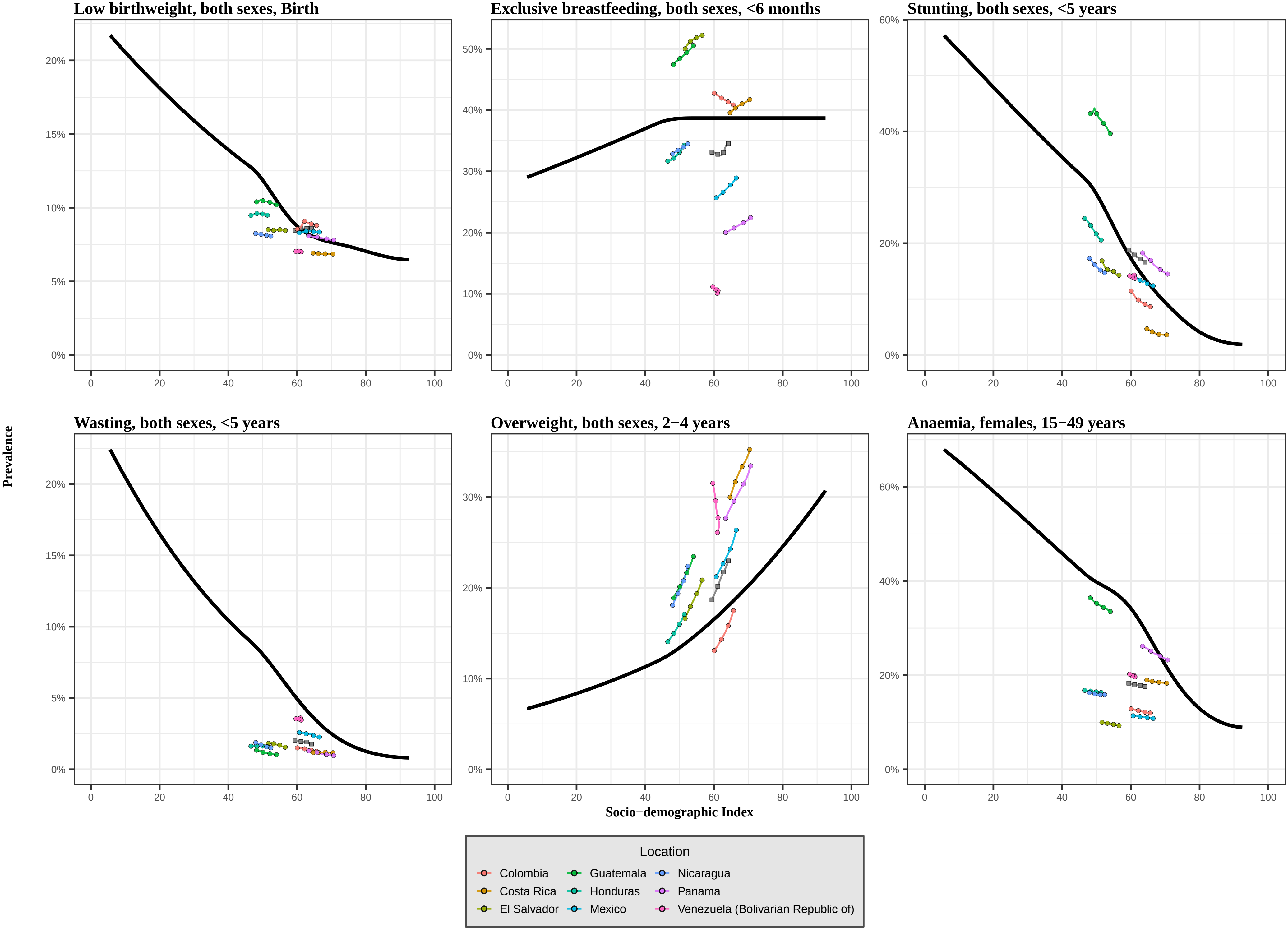

The expected prevalence values of each indicator based on the Socio−demographic Index (SDI) are represented by the solid black lines. Observed values of the indicator are shown for each country in Central Latin America, with the region's values shown in grey. Points are shown every 3 years from 2012 to 2021.

Figure S5. Co−evolution of GNT indicator prevalence in Tropical Latin America, 2012 to 2021

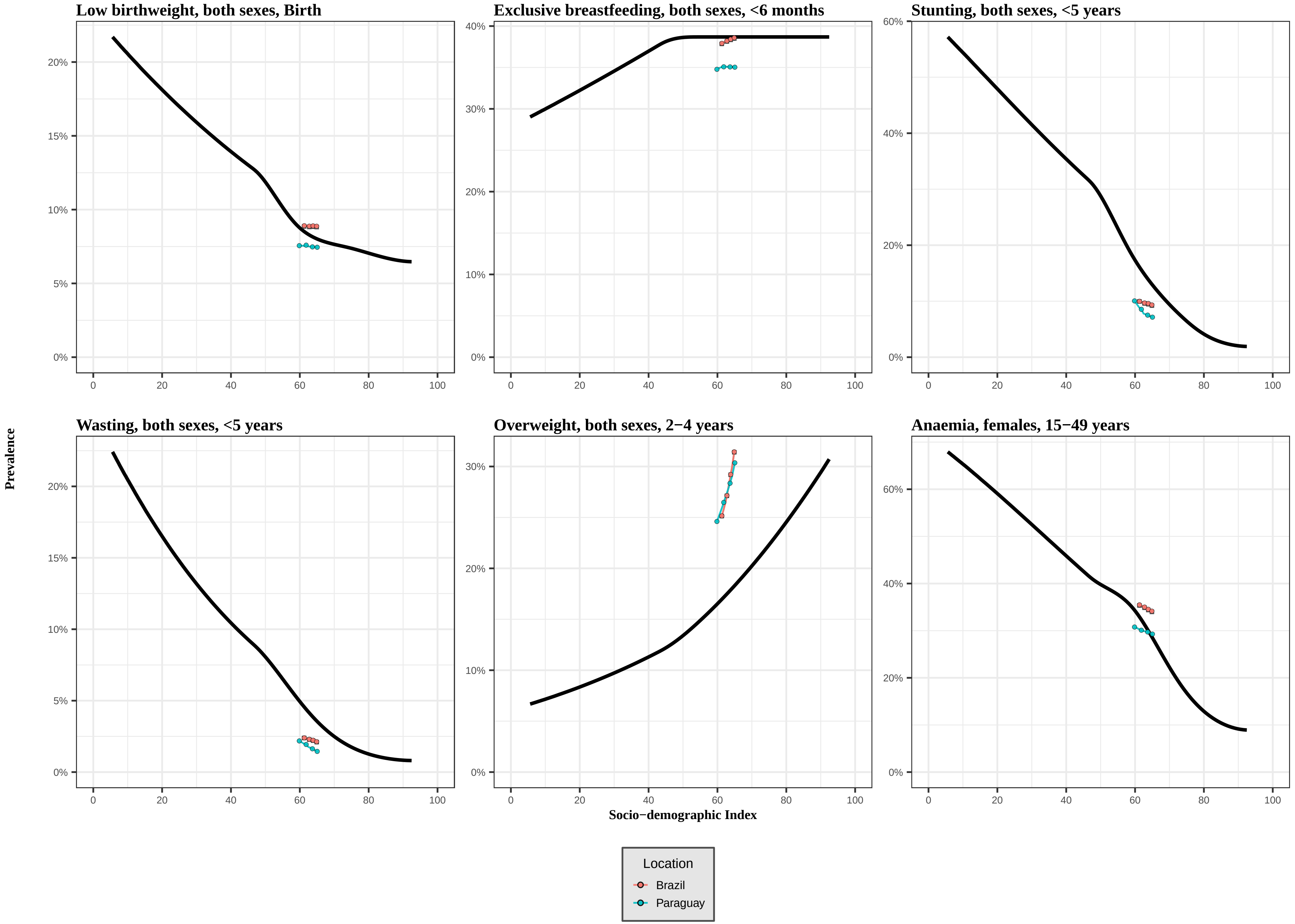

The expected prevalence values of each indicator based on the Socio−demographic Index (SDI) are represented by the solid black lines. Observed values of the indicator are shown for each country in Tropical Latin America, with the region's values shown in grey. Points are shown every 3 years from 2012 to 2021.

Figure S5. Co−evolution of GNT indicator prevalence in North Africa and Middle East, 2012 to 2021

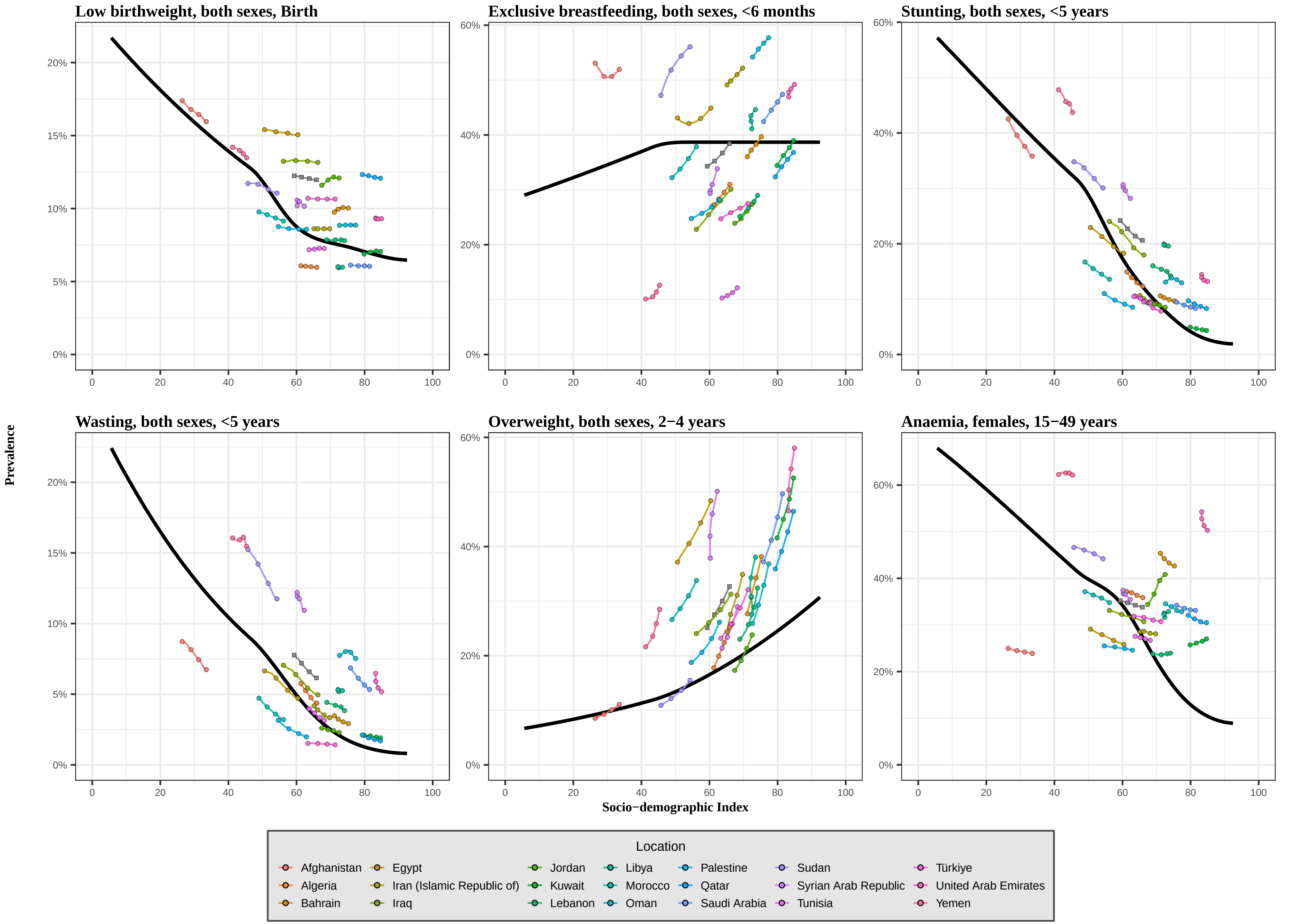

The expected prevalence values of each indicator based on the Socio−demographic Index (SDI) are represented by the solid black lines. Observed values of the indicator are shown for each country in North Africa and Middle East, with the region's values shown in grey. Points are shown every 3 years from 2012 to 2021.

Figure S5. Co−evolution of GNT indicator prevalence in South Asia, 2012 to 2021

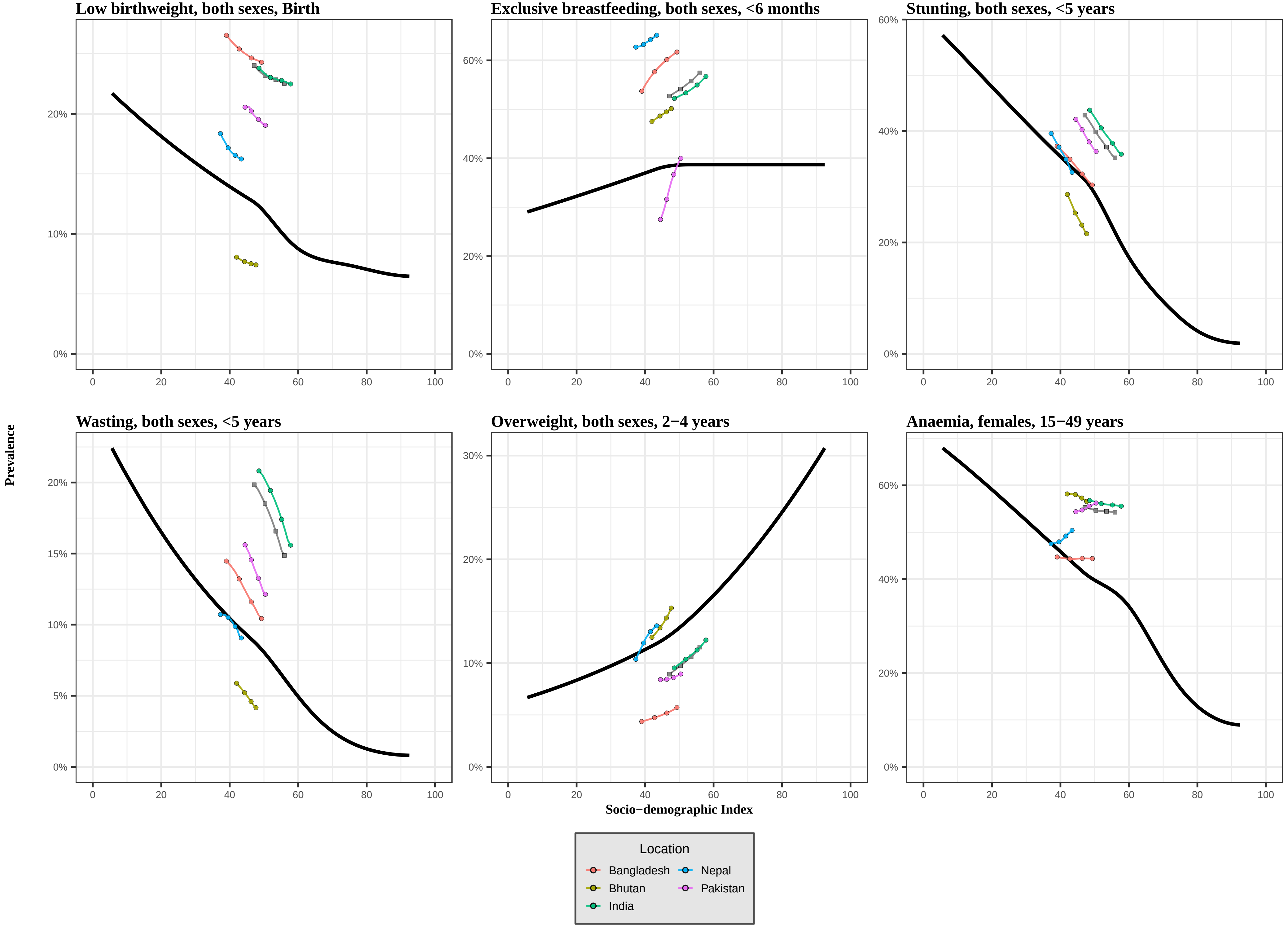

The expected prevalence values of each indicator based on the Socio−demographic Index (SDI) are represented by the solid black lines. Observed values of the indicator are shown for each country in South Asia, with the region's values shown in grey. Points are shown every 3 years from 2012 to 2021.

Figure S5. Co−evolution of GNT indicator prevalence in East Asia, 2012 to 2021

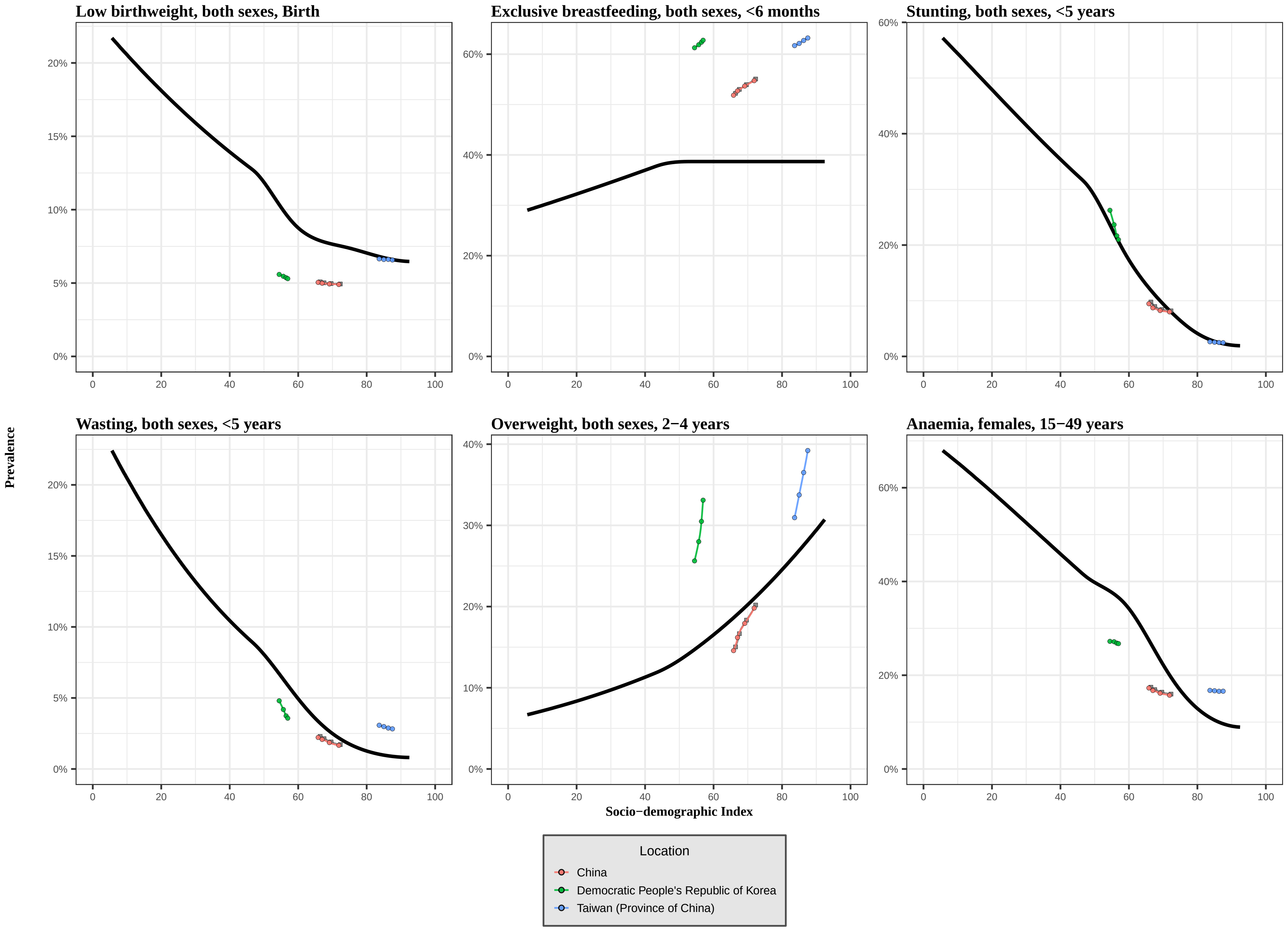

The expected prevalence values of each indicator based on the Socio−demographic Index (SDI) are represented by the solid black lines. Observed values of the indicator are shown for each country in East Asia, with the region's values shown in grey. Points are shown every 3 years from 2012 to 2021.

Figure S5. Co−evolution of GNT indicator prevalence in Oceania, 2012 to 2021

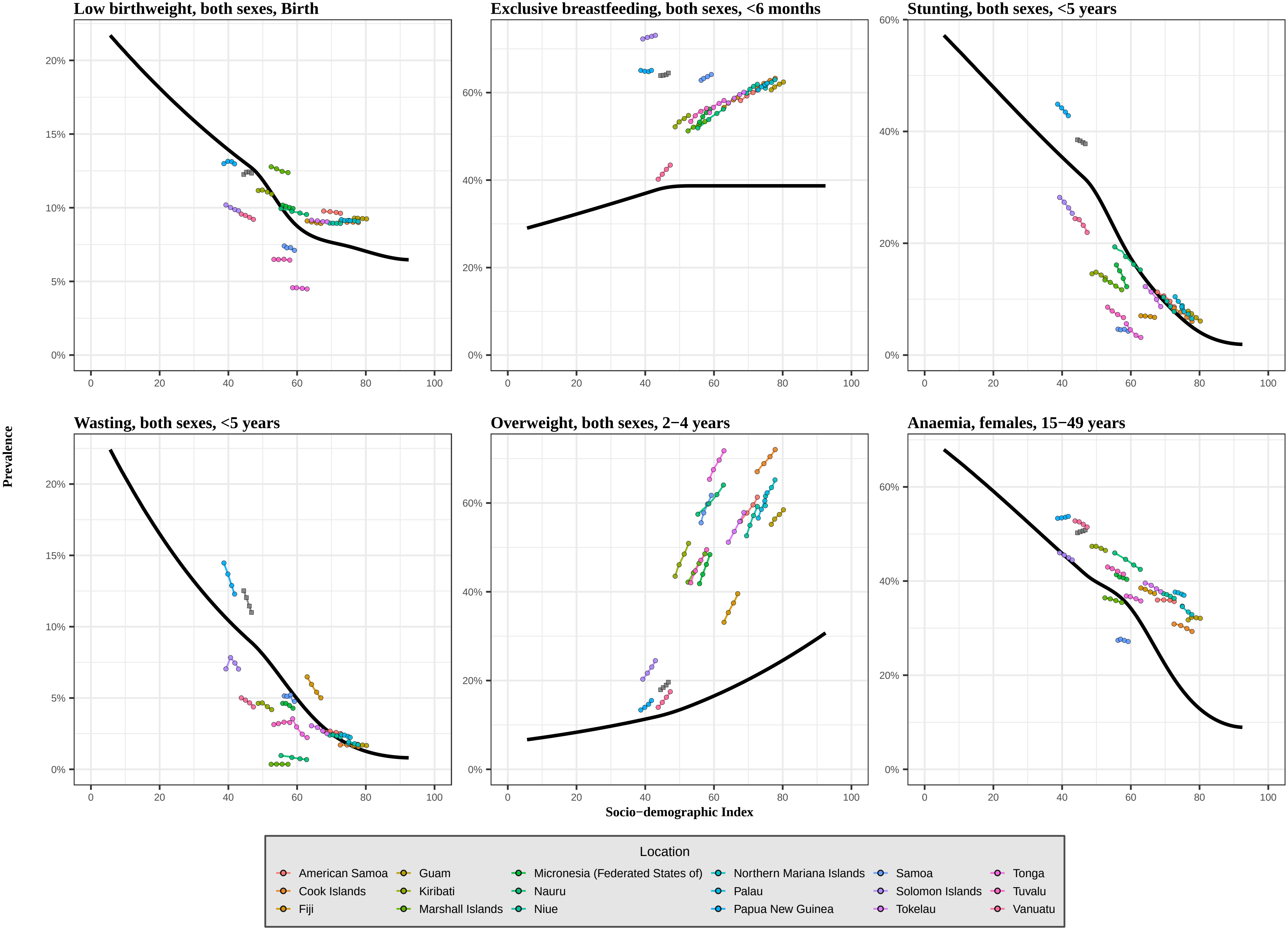

The expected prevalence values of each indicator based on the Socio−demographic Index (SDI) are represented by the solid black lines. Observed values of the indicator are shown for each country in Oceania, with the region's values shown in grey. Points are shown every 3 years from 2012 to 2021.

Figure S5. Co−evolution of GNT indicator prevalence in Southeast Asia, 2012 to 2021

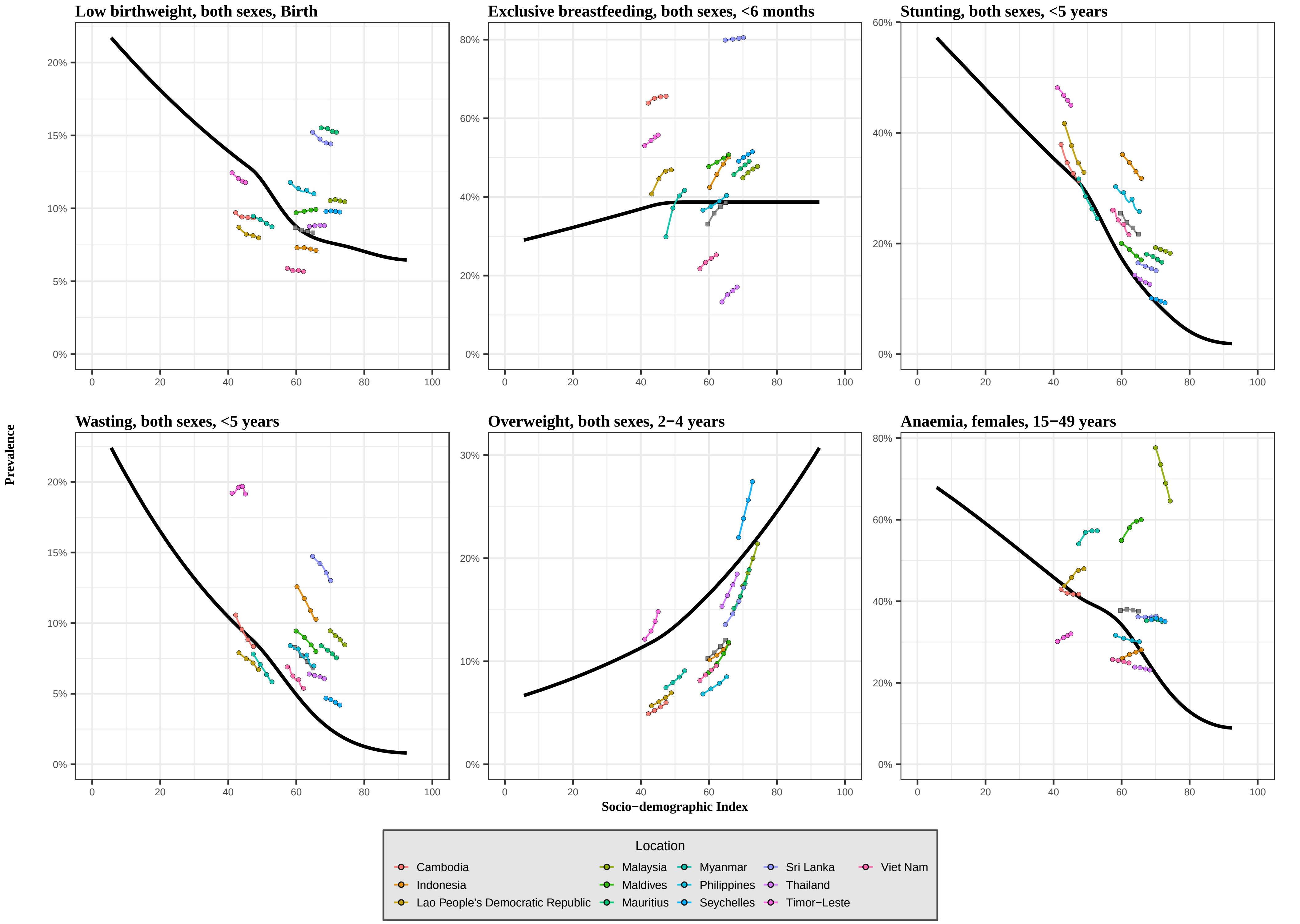

The expected prevalence values of each indicator based on the Socio−demographic Index (SDI) are represented by the solid black lines. Observed values of the indicator are shown for each country in Southeast Asia, with the region's values shown in grey. Points are shown every 3 years from 2012 to 2021.

Figure S5. Co−evolution of GNT indicator prevalence in Central Sub−Saharan Africa, 2012 to 2021

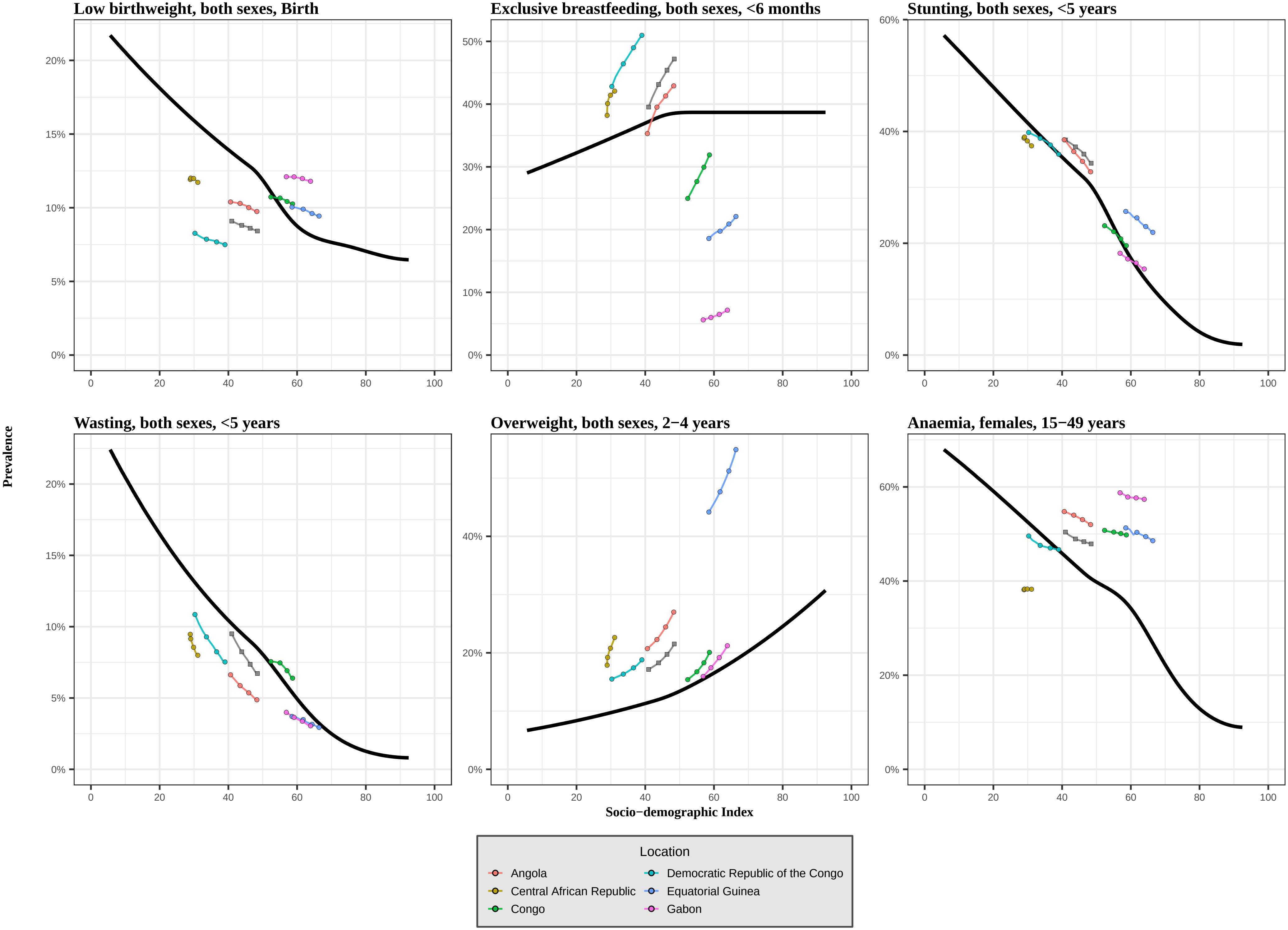

The expected prevalence values of each indicator based on the Socio−demographic Index (SDI) are represented by the solid black lines. Observed values of the indicator are shown for each country in Central Sub−Saharan Africa, with the region's values shown in grey. Points are shown every 3 years from 2012 to 2021.

Figure S5. Co−evolution of GNT indicator prevalence in Eastern Sub−Saharan Africa, 2012 to 2021

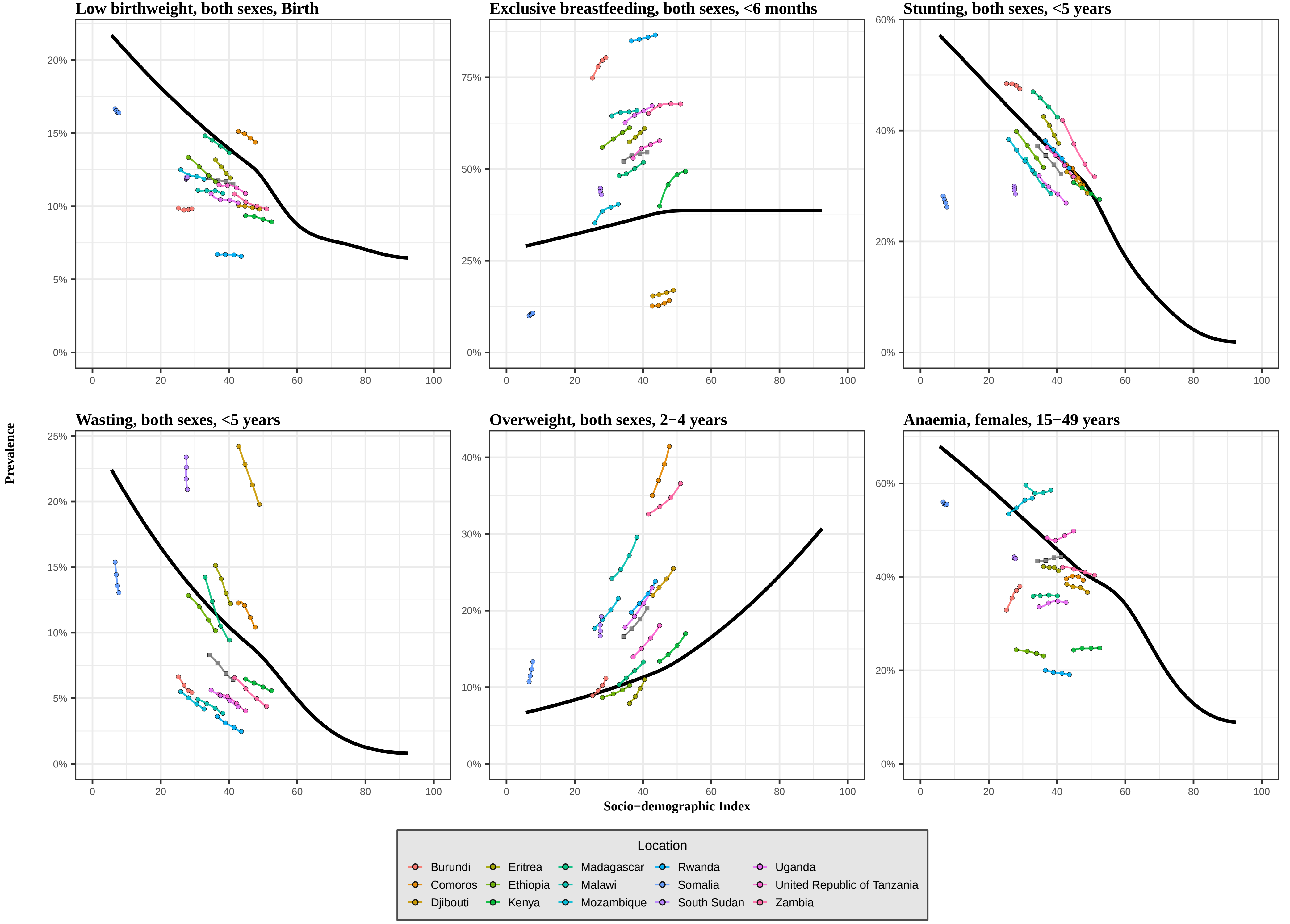

The expected prevalence values of each indicator based on the Socio−demographic Index (SDI) are represented by the solid black lines. Observed values of the indicator are shown for each country in Eastern Sub−Saharan Africa, with the region's values shown in grey. Points are shown every 3 years from 2012 to 2021.

Figure S5. Co−evolution of GNT indicator prevalence in Southern Sub−Saharan Africa, 2012 to 2021

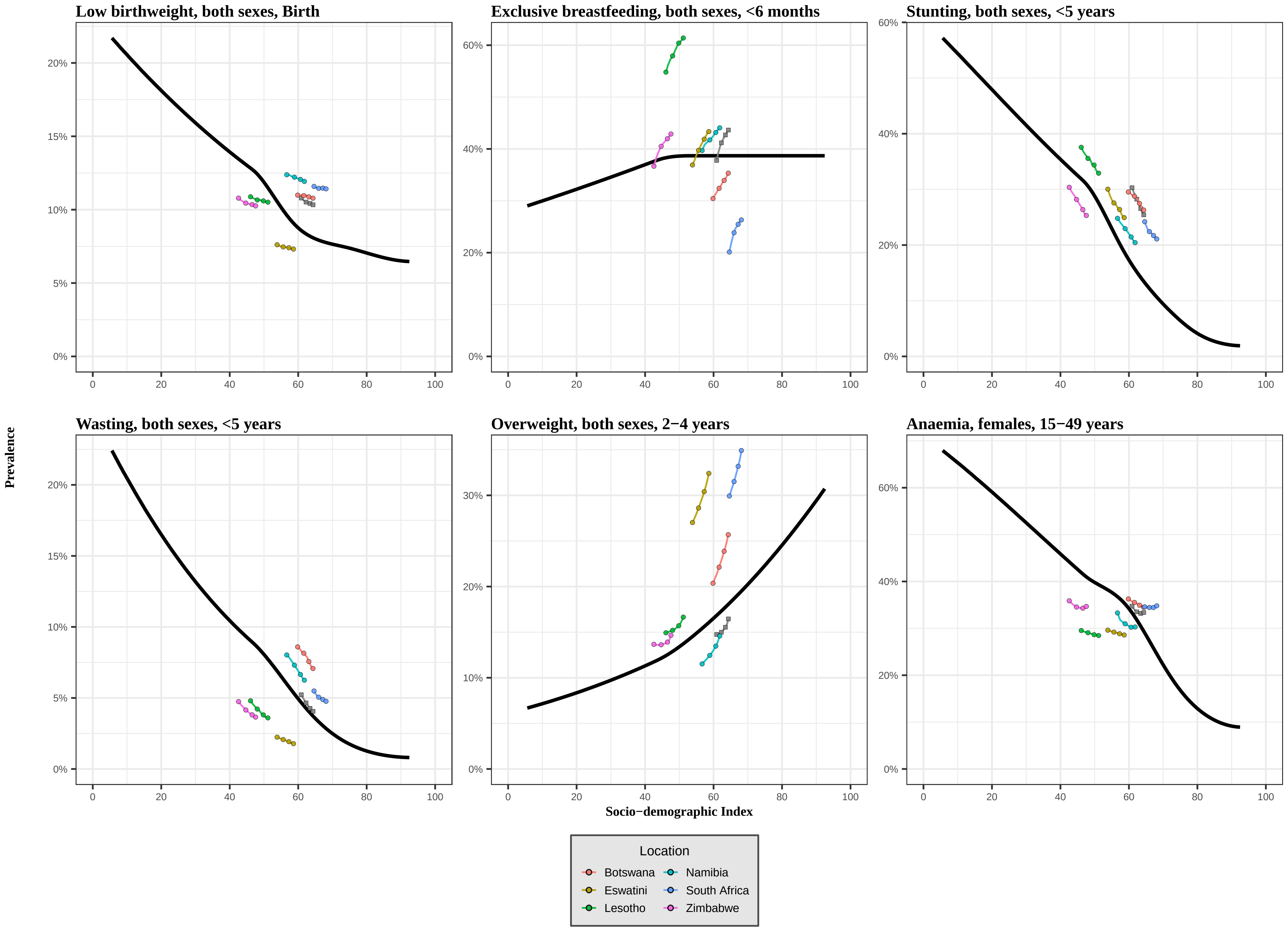

The expected prevalence values of each indicator based on the Socio−demographic Index (SDI) are represented by the solid black lines. Observed values of the indicator are shown for each country in Southern Sub−Saharan Africa, with the region's values shown in grey. Points are shown every 3 years from 2012 to 2021.

Figure S5. Co−evolution of GNT indicator prevalence in Western Sub−Saharan Africa, 2012 to 2021

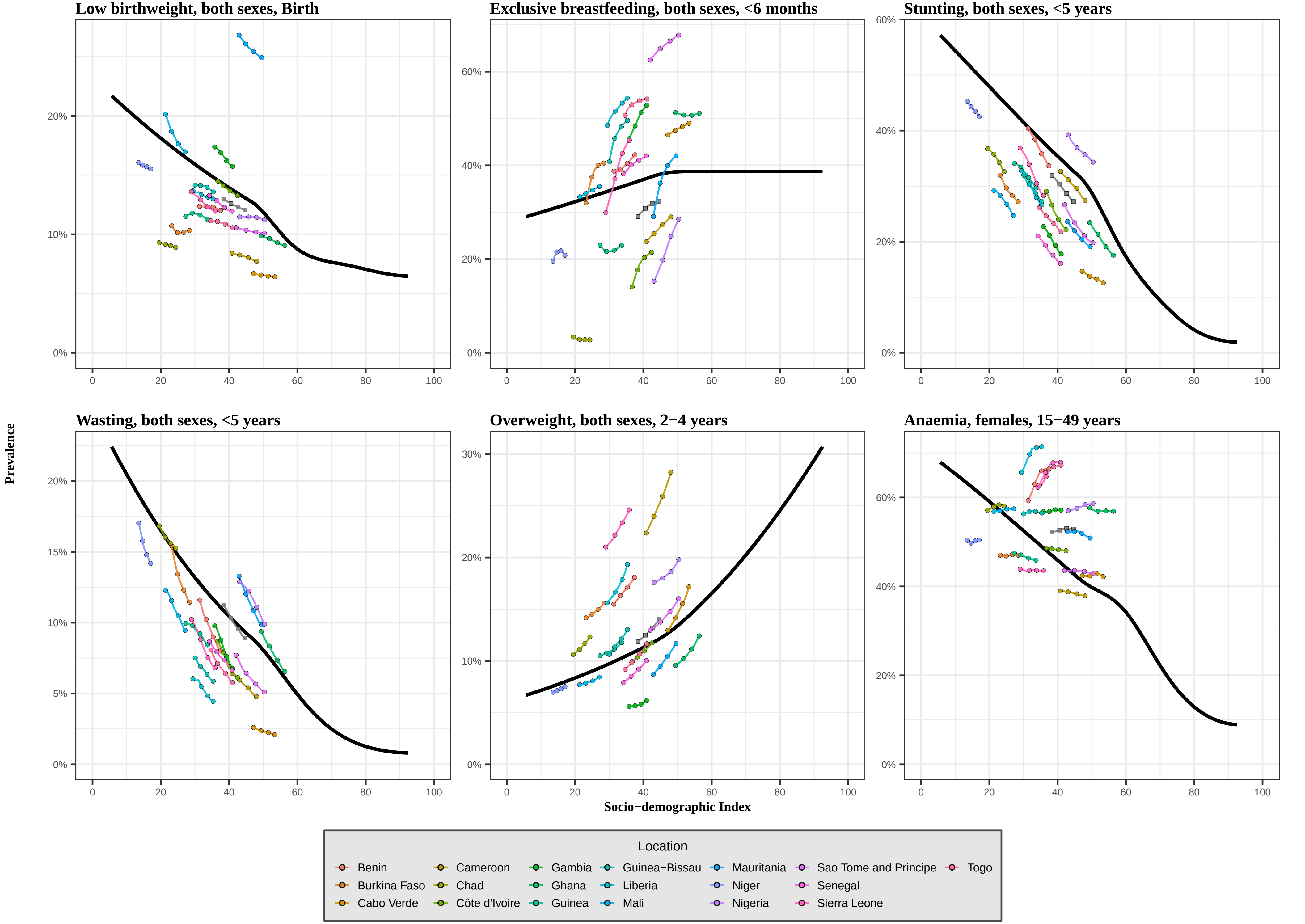

The expected prevalence values of each indicator based on the Socio−demographic Index (SDI) are represented by the solid black lines. Observed values of the indicator are shown for each country in Western Sub−Saharan Africa, with the region's values shown in grey. Points are shown every 3 years from 2012 to 2021.

Figure S6. Ratios of observed to expected prevalence based on SDI, 2012

Low birthweight, both sexes, birth

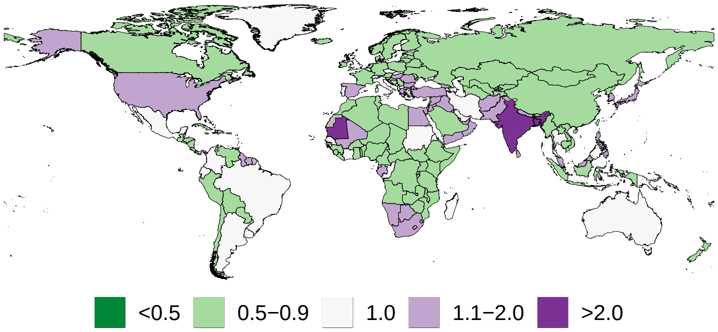

Stunting, both sexes, <5 years

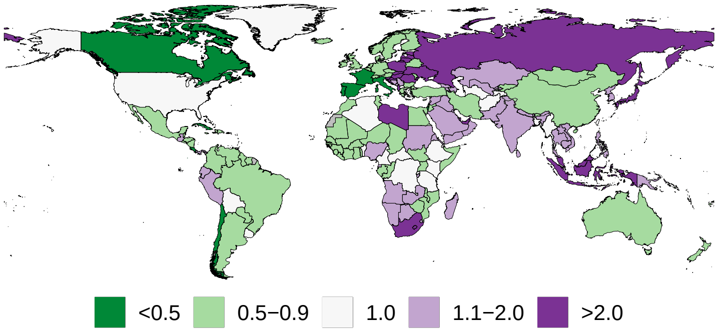

Wasting, both sexes, <5 years

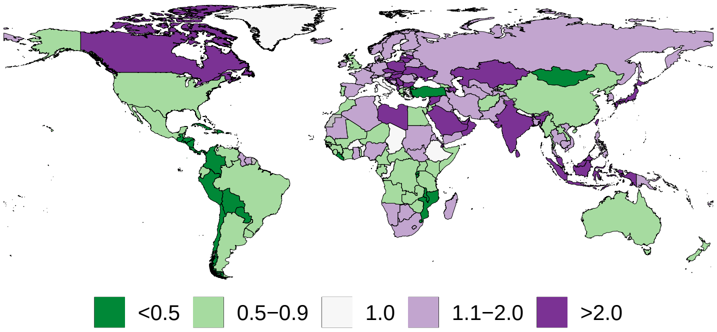

Overweight, both sexes, 2−4 years

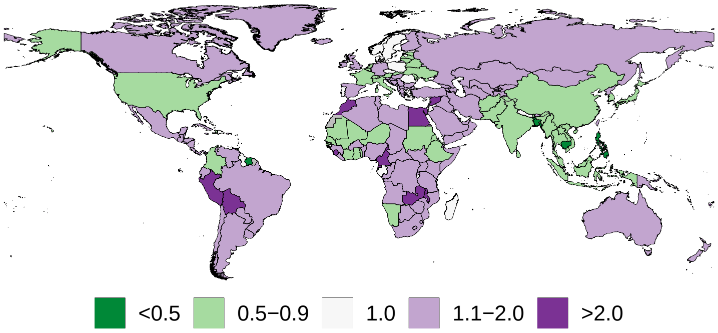

Anaemia, females, 15−49 years

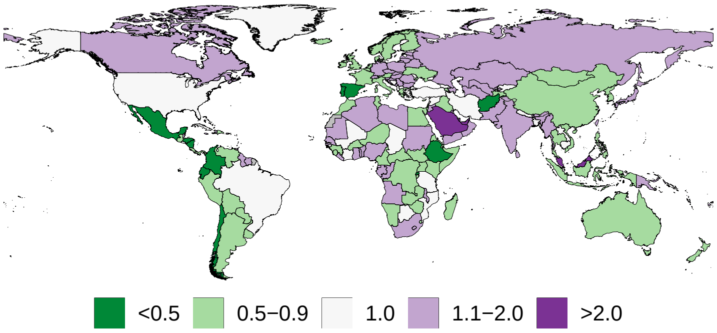

Figure S7. Ratios of observed to expected prevalence based on SDI, 2021

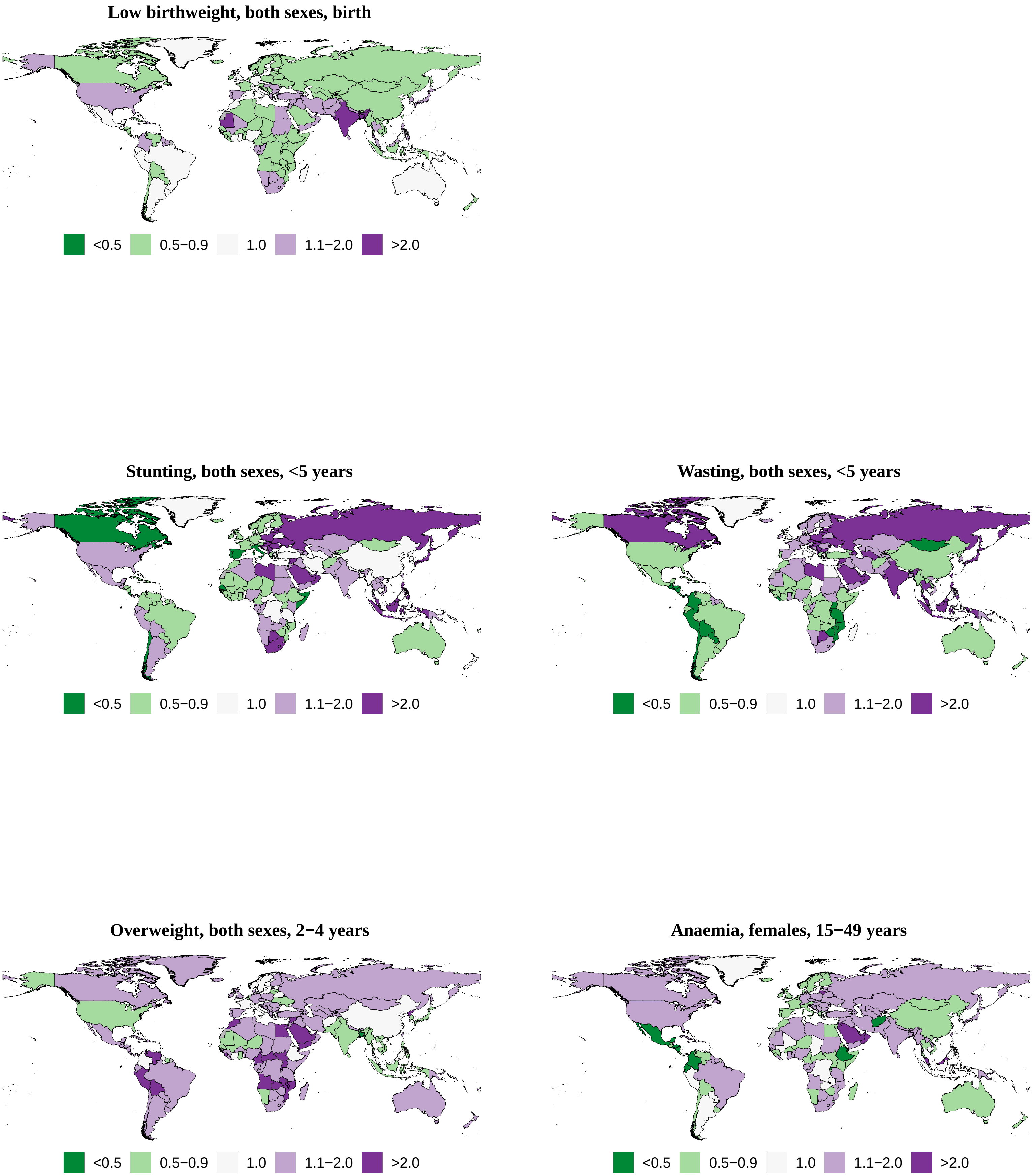

Figure S8. Differences in observed ARC and expected ARC based on SDI, 2012 to 2021

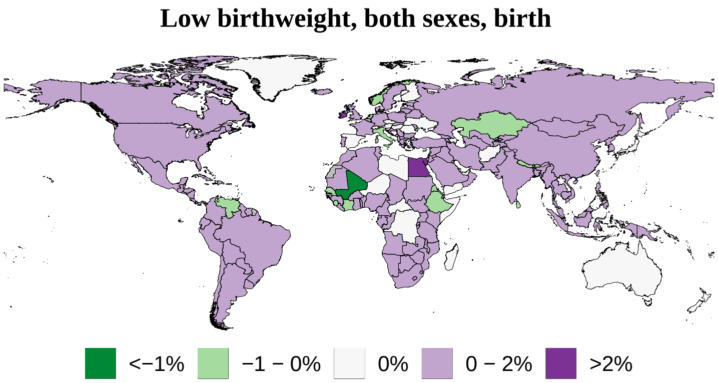

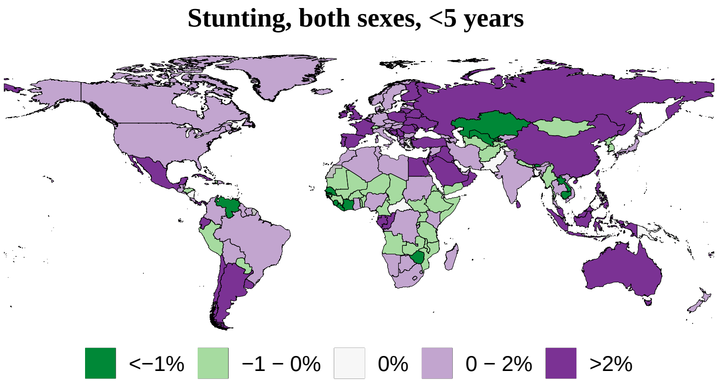

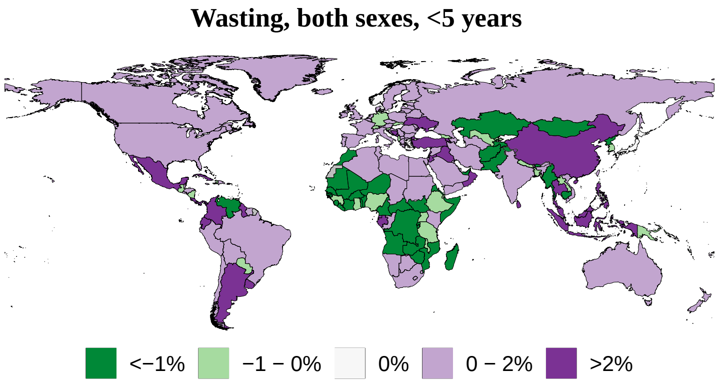

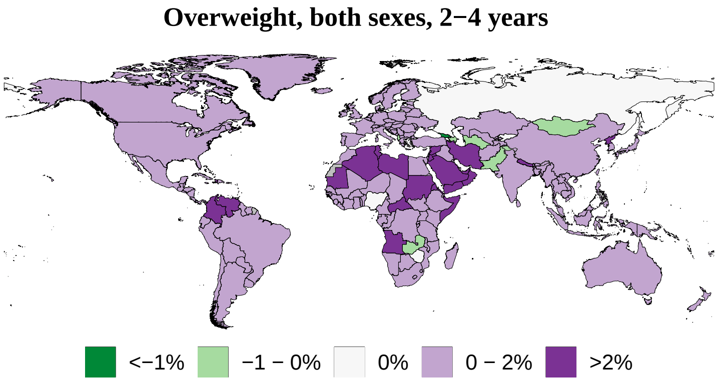

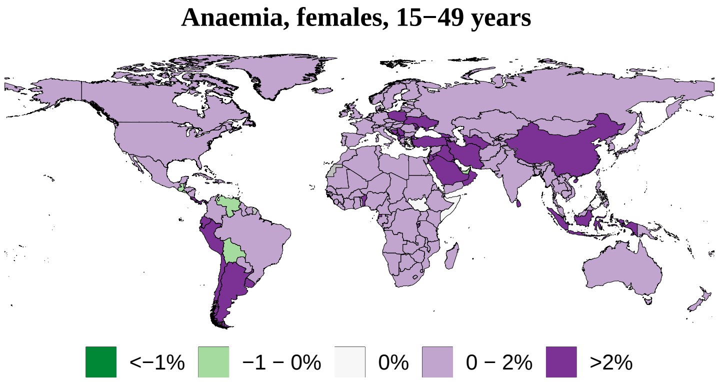

Figure S9. Comparison of annual rates of change from 2012 to 2021: prevalence vs. attributable burden, Central Asia

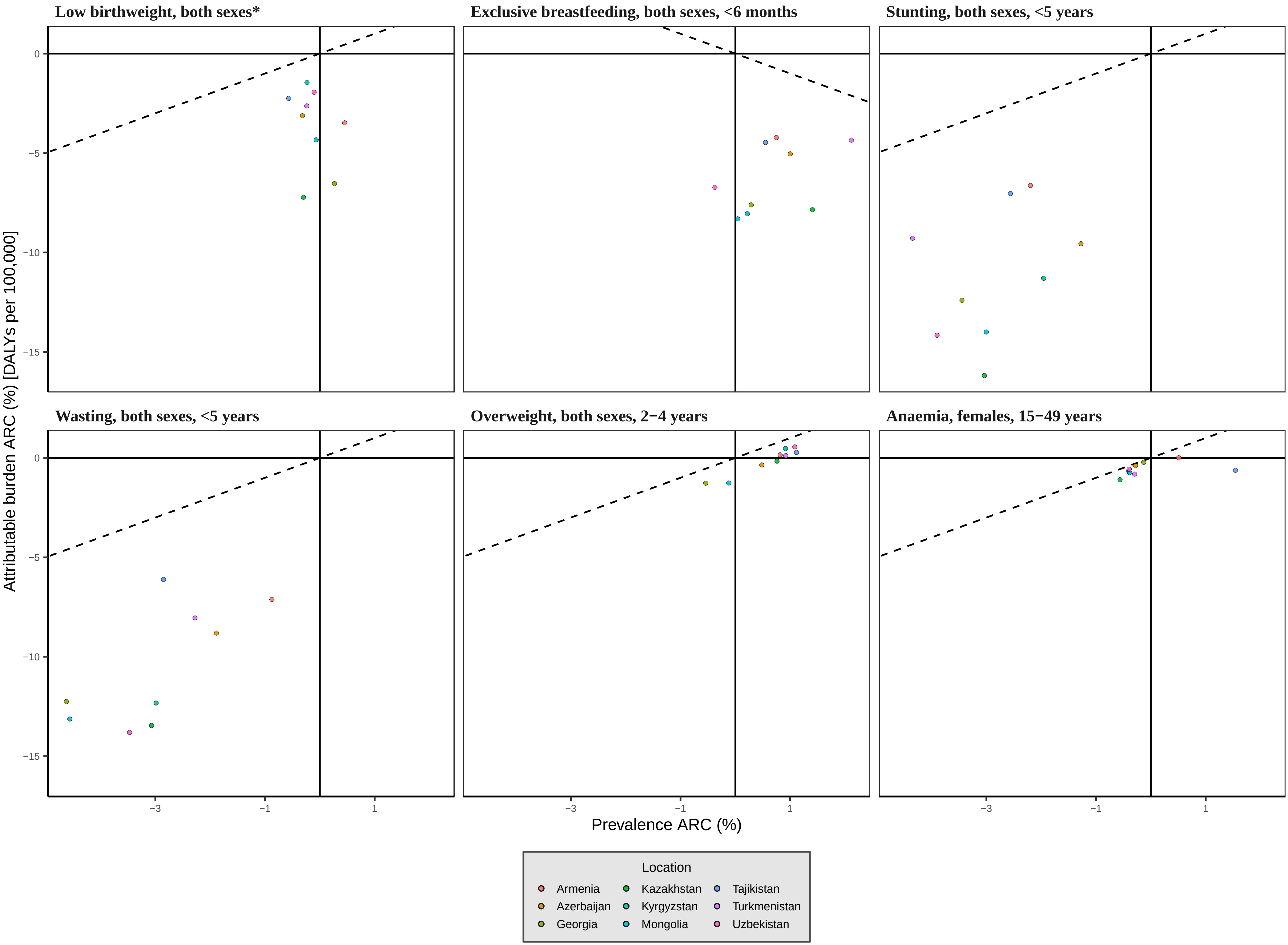

*Low birthweight prevalence ARC is at birth and attributable burden ARC is during neonatal age group (0 − 27 days). Exclusive breastfeeding line of unity is −1 due to the nature of the indicator.

Figure S9. Comparison of annual rates of change from 2012 to 2021: prevalence vs. attributable burden, Central Europe

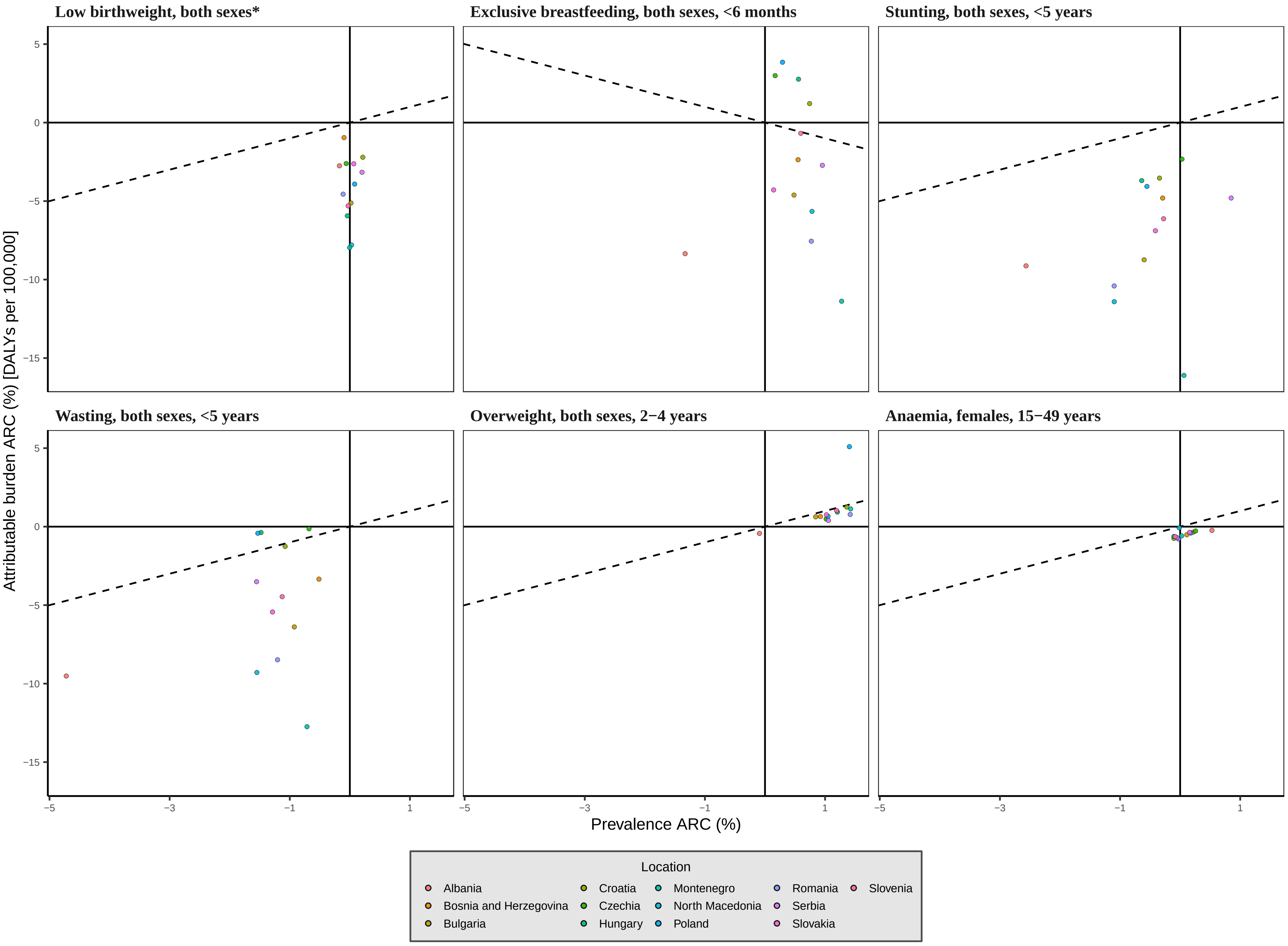

*Low birthweight prevalence ARC is at birth and attributable burden ARC is during neonatal age group (0 − 27 days). Exclusive breastfeeding line of unity is −1 due to the nature of the indicator.

Figure S9. Comparison of annual rates of change from 2012 to 2021: prevalence vs. attributable burden, Eastern Europe

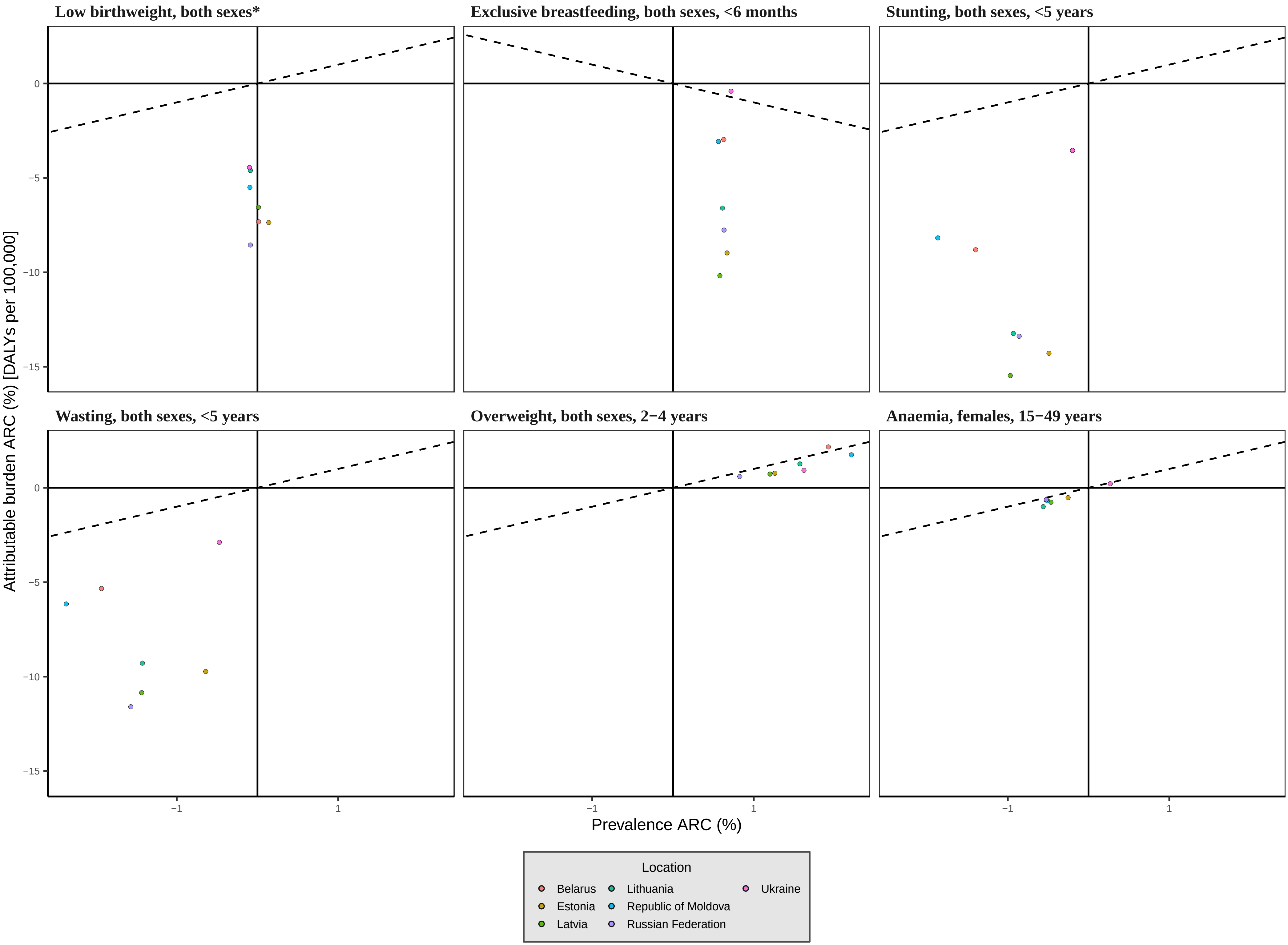

*Low birthweight prevalence ARC is at birth and attributable burden ARC is during neonatal age group (0 − 27 days). Exclusive breastfeeding line of unity is −1 due to the nature of the indicator.

Figure S9. Comparison of annual rates of change from 2012 to 2021: prevalence vs. attributable burden, Australasia

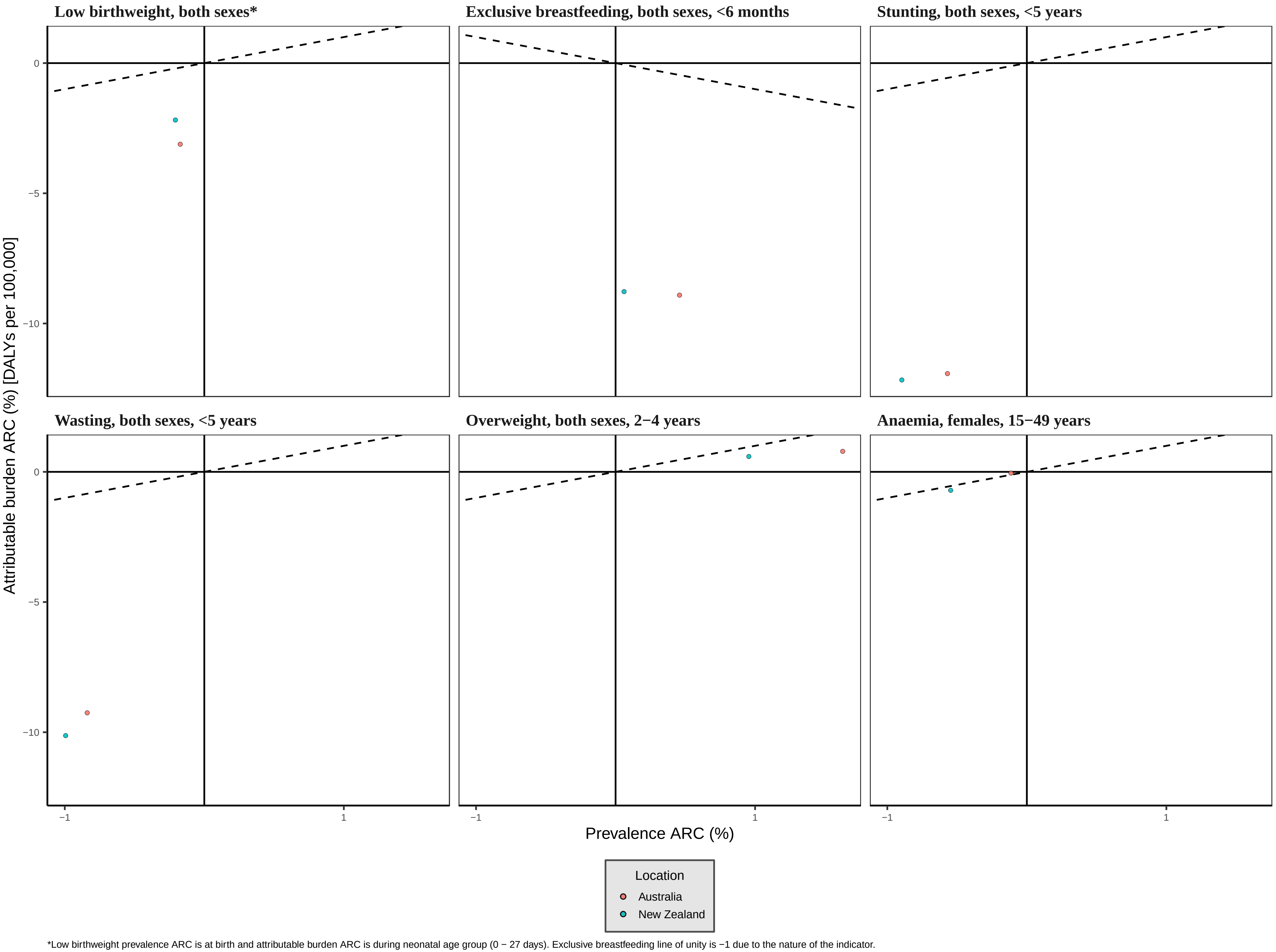

*Low birthweight prevalence ARC is at birth and attributable burden ARC is during neonatal age group (0 − 27 days). Exclusive breastfeeding line of unity is −1 due to the nature of the indicator.

Figure S9. Comparison of annual rates of change from 2012 to 2021: prevalence vs. attributable burden, High−income Asia Pacific

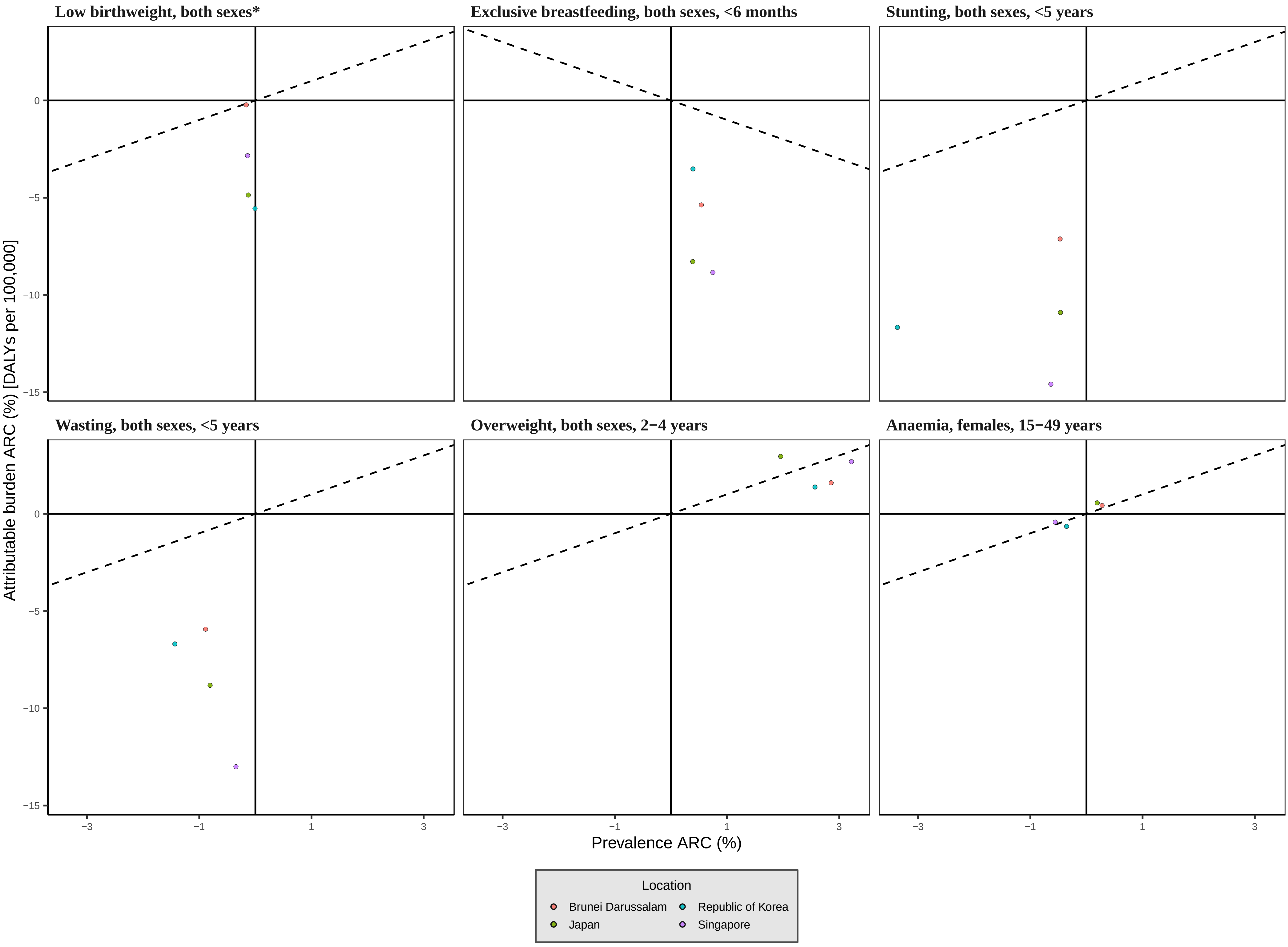

*Low birthweight prevalence ARC is at birth and attributable burden ARC is during neonatal age group (0 − 27 days). Exclusive breastfeeding line of unity is −1 due to the nature of the indicator.

Figure S9. Comparison of annual rates of change from 2012 to 2021: prevalence vs. attributable burden, High−income North America

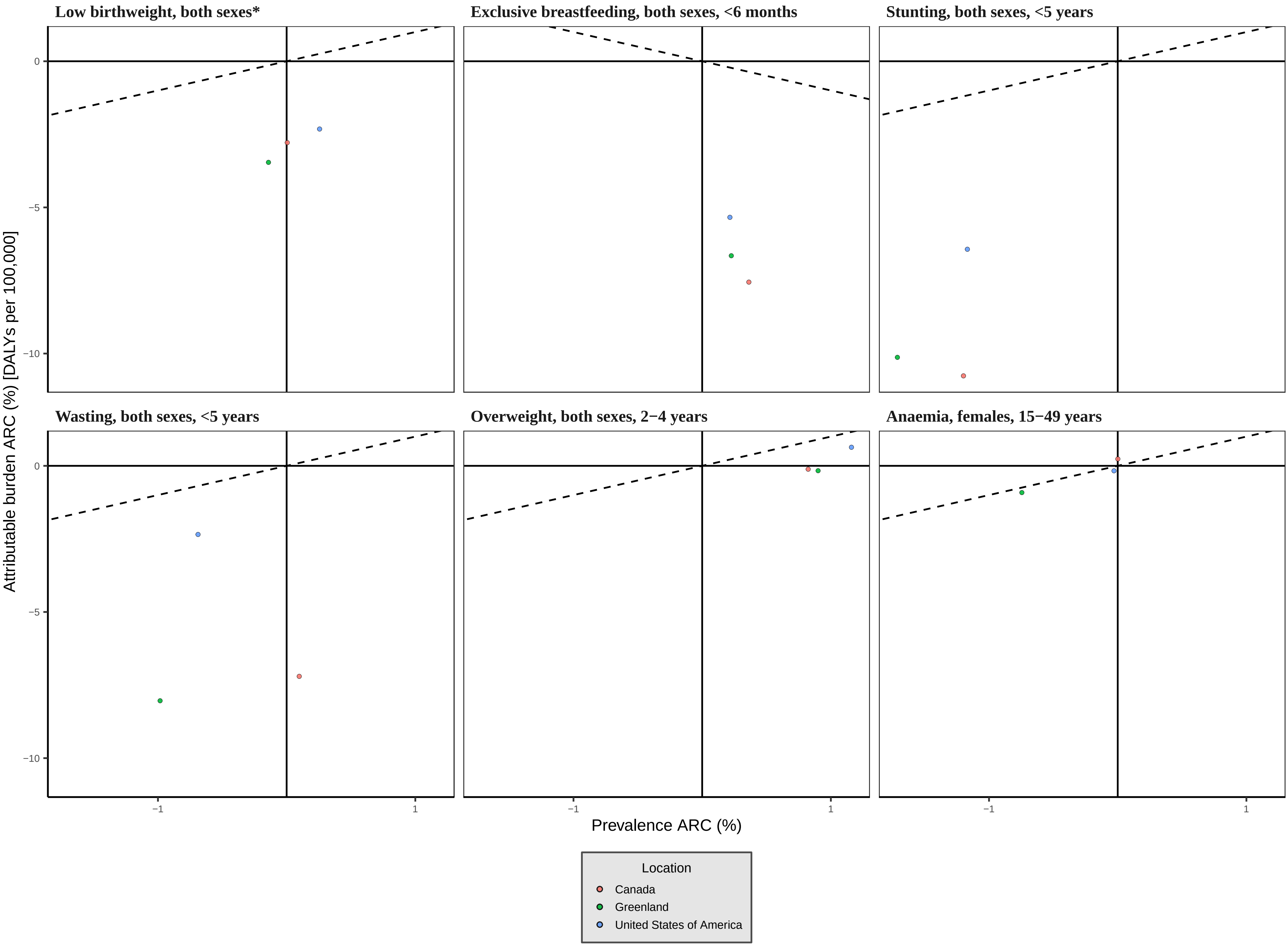

*Low birthweight prevalence ARC is at birth and attributable burden ARC is during neonatal age group (0 − 27 days). Exclusive breastfeeding line of unity is −1 due to the nature of the indicator.

Figure S9. Comparison of annual rates of change from 2012 to 2021: prevalence vs. attributable burden, Southern Latin America

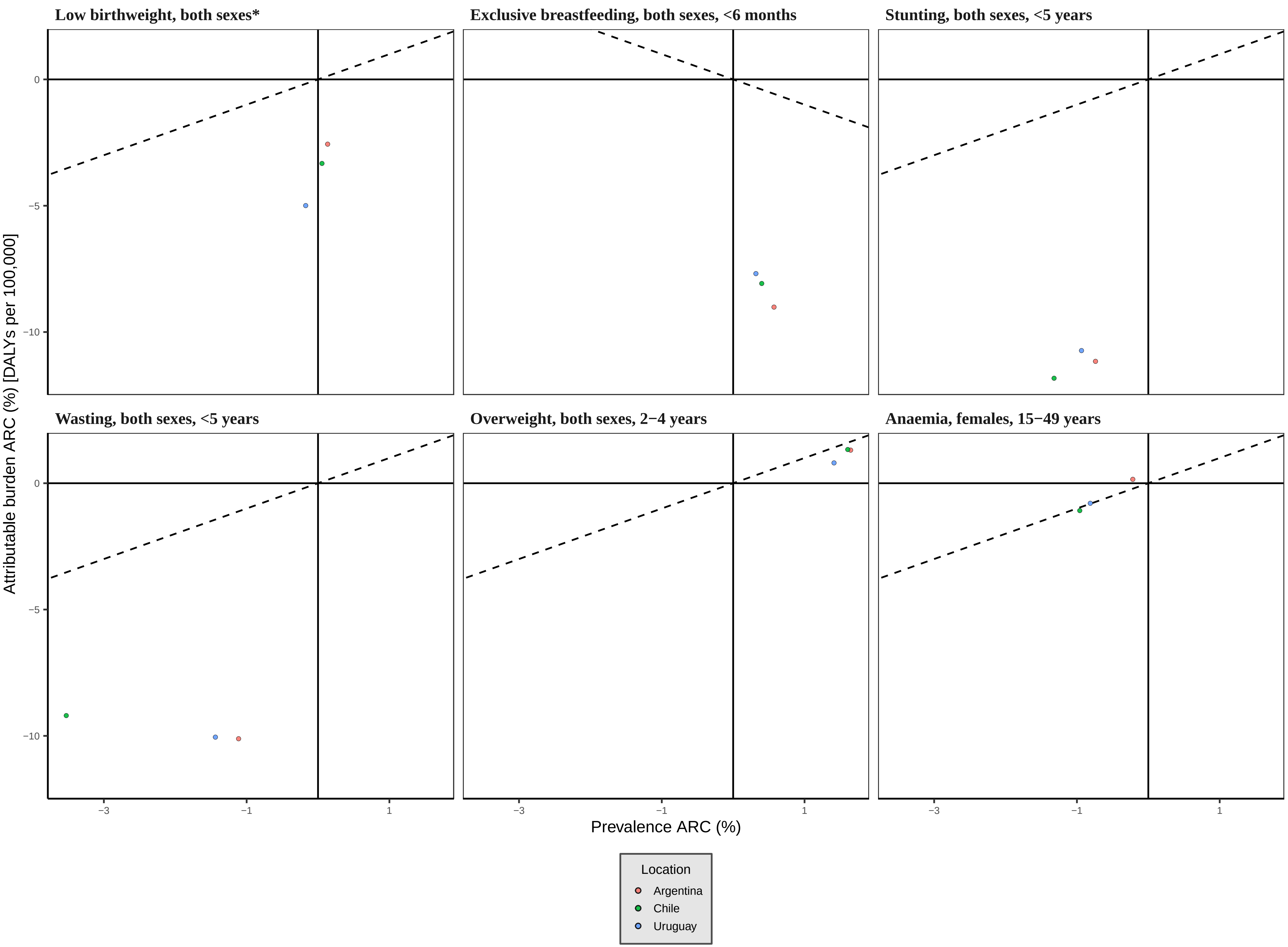

*Low birthweight prevalence ARC is at birth and attributable burden ARC is during neonatal age group (0 − 27 days). Exclusive breastfeeding line of unity is −1 due to the nature of the indicator.

Figure S9. Comparison of annual rates of change from 2012 to 2021: prevalence vs. attributable burden, Western Europe

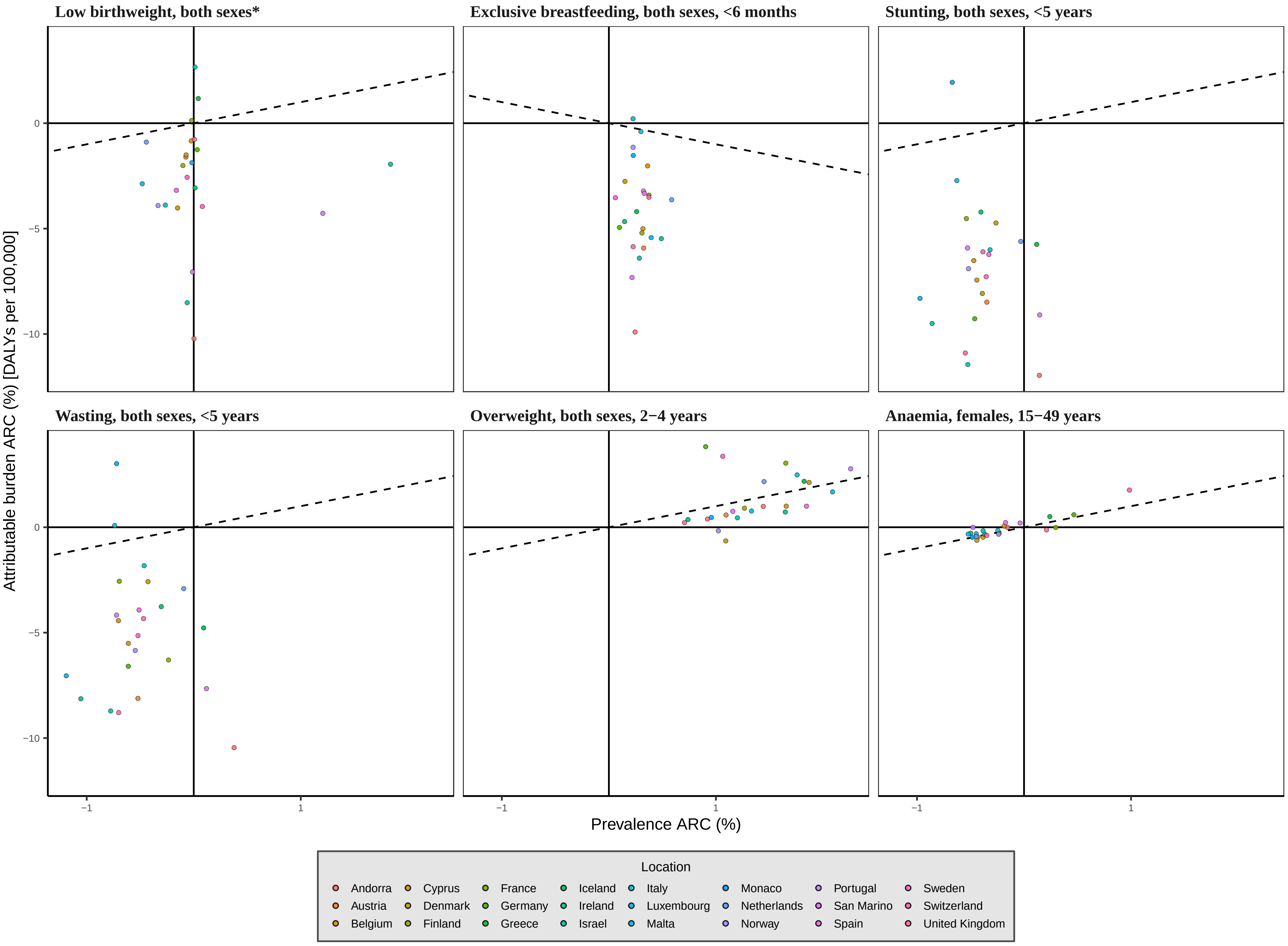

*Low birthweight prevalence ARC is at birth and attributable burden ARC is during neonatal age group (0 − 27 days). Exclusive breastfeeding line of unity is −1 due to the nature of the indicator.

Figure S9. Comparison of annual rates of change from 2012 to 2021: prevalence vs. attributable burden, Andean Latin America

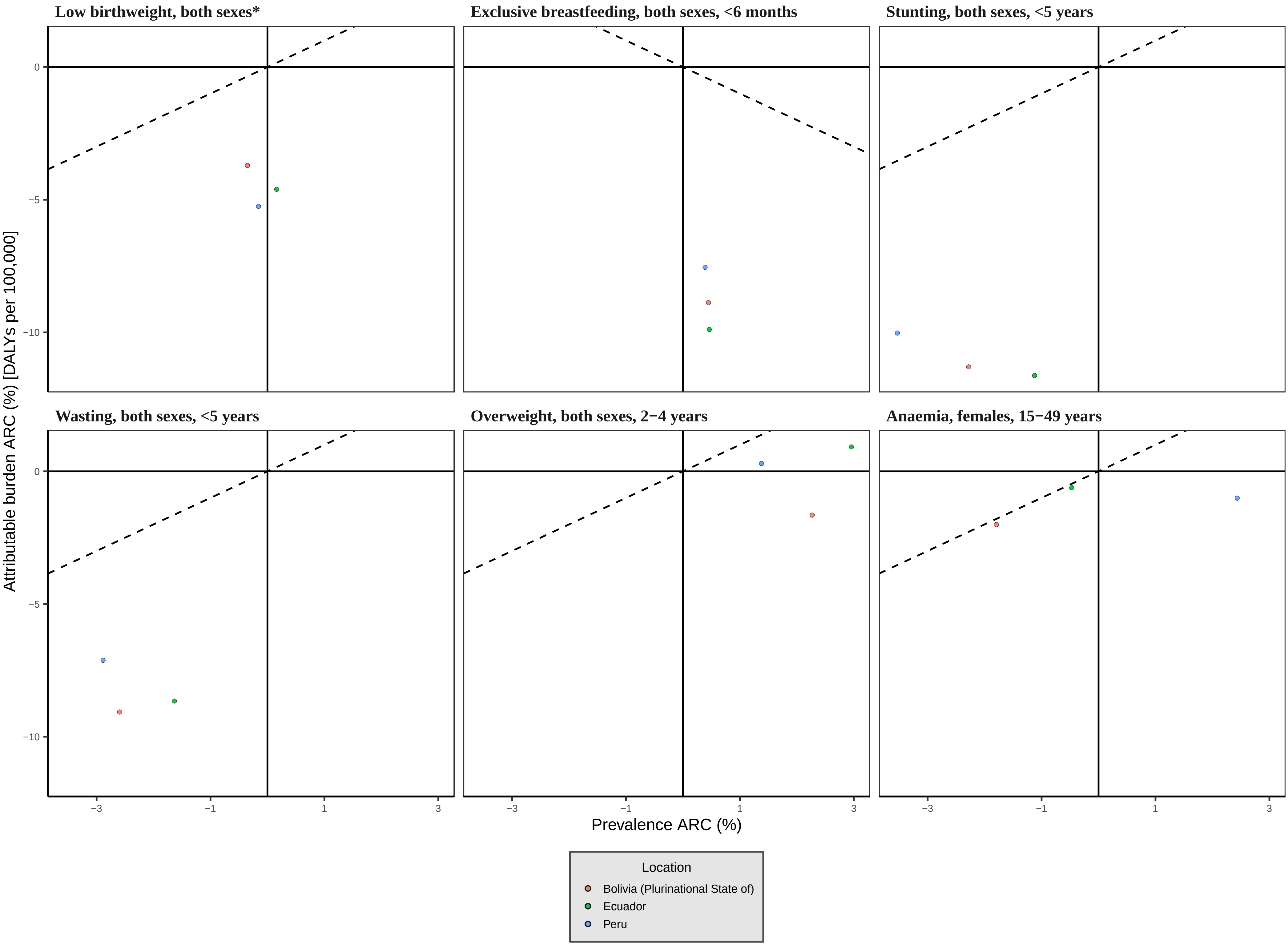

*Low birthweight prevalence ARC is at birth and attributable burden ARC is during neonatal age group (0 − 27 days). Exclusive breastfeeding line of unity is −1 due to the nature of the indicator.

Figure S9. Comparison of annual rates of change from 2012 to 2021: prevalence vs. attributable burden, Caribbean

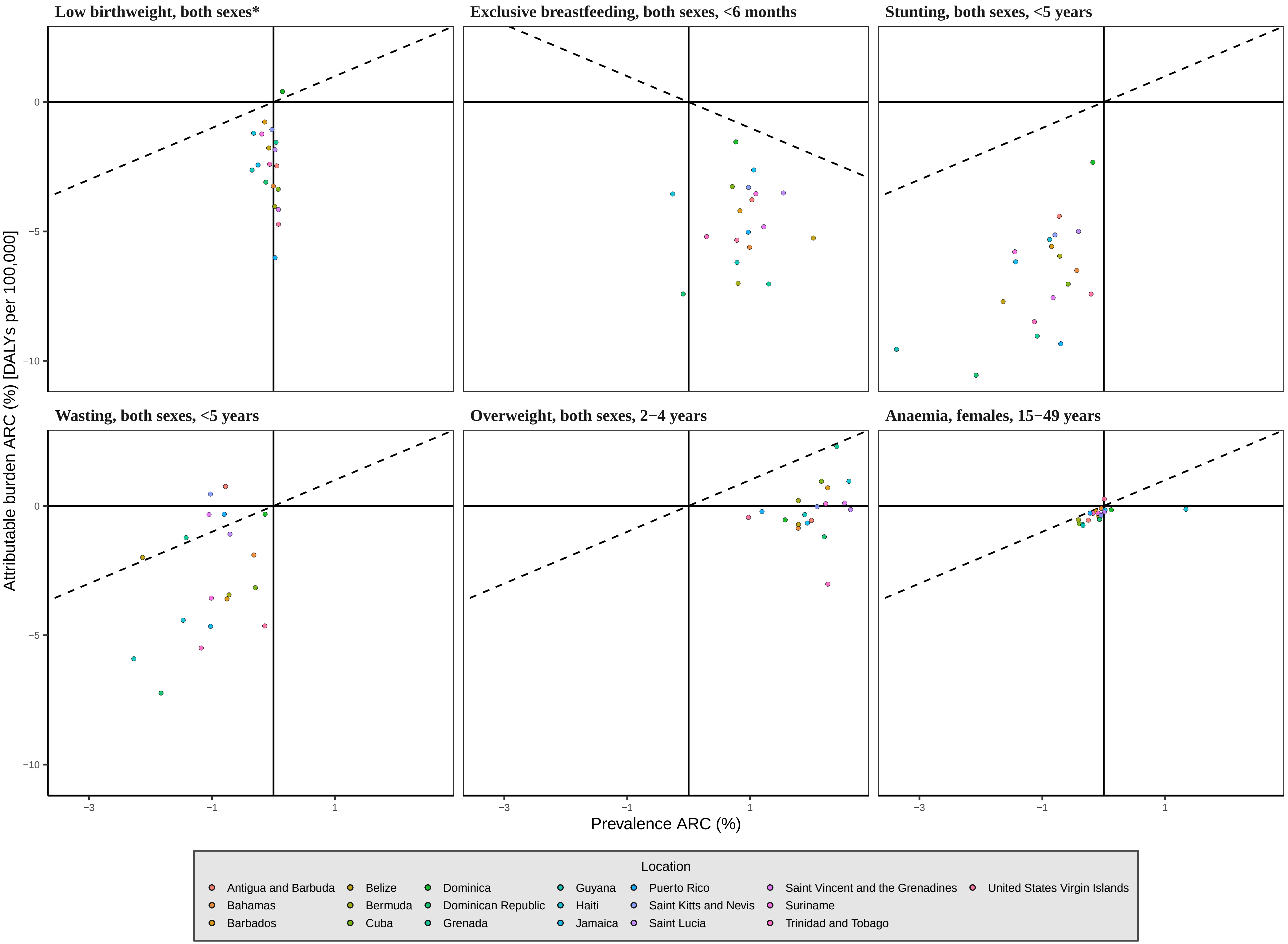

*Low birthweight prevalence ARC is at birth and attributable burden ARC is during neonatal age group (0 − 27 days). Exclusive breastfeeding line of unity is −1 due to the nature of the indicator.

Figure S9. Comparison of annual rates of change from 2012 to 2021: prevalence vs. attributable burden, Central Latin America

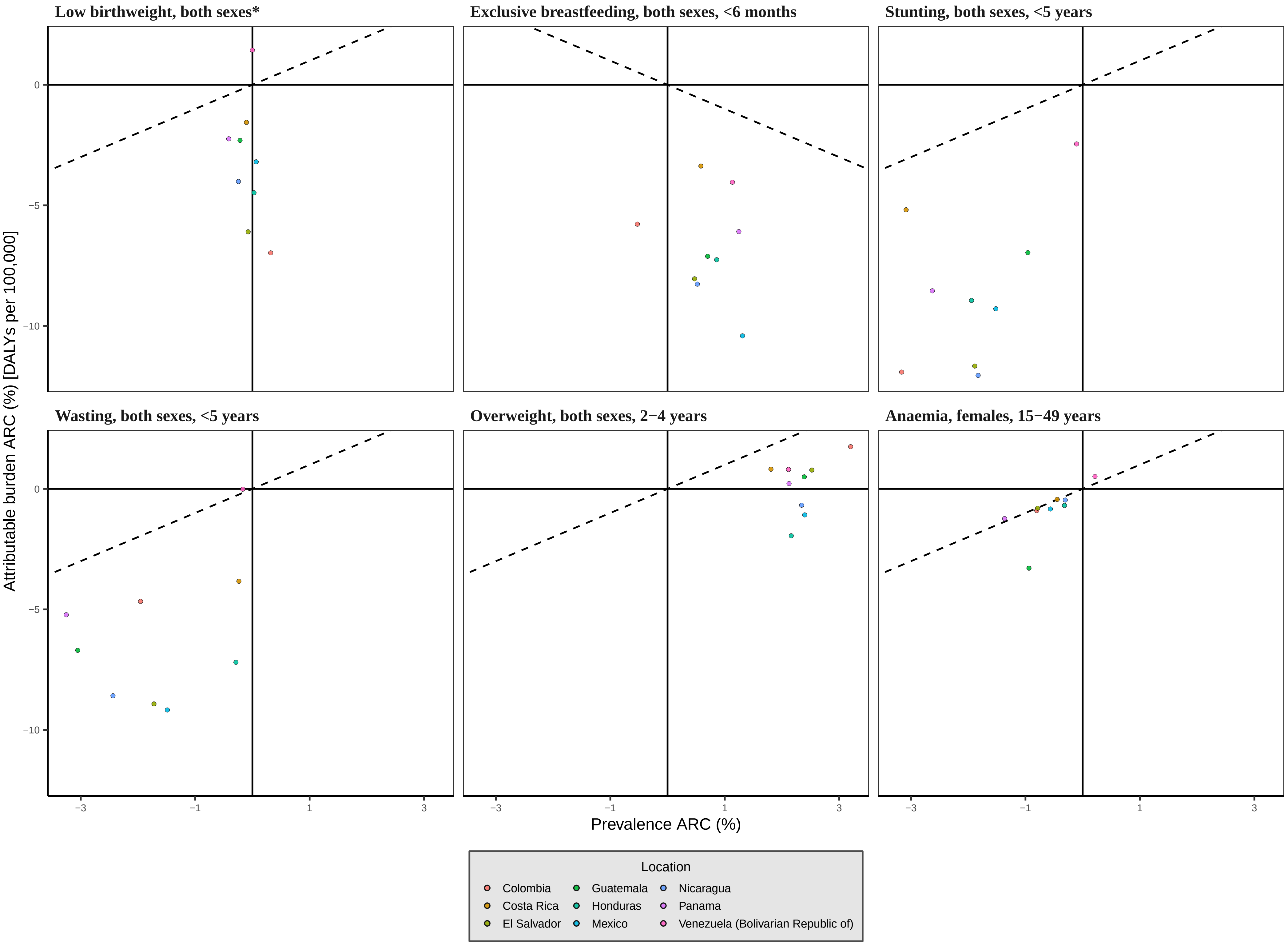

*Low birthweight prevalence ARC is at birth and attributable burden ARC is during neonatal age group (0 − 27 days). Exclusive breastfeeding line of unity is −1 due to the nature of the indicator.

Figure S9. Comparison of annual rates of change from 2012 to 2021: prevalence vs. attributable burden, Tropical Latin America

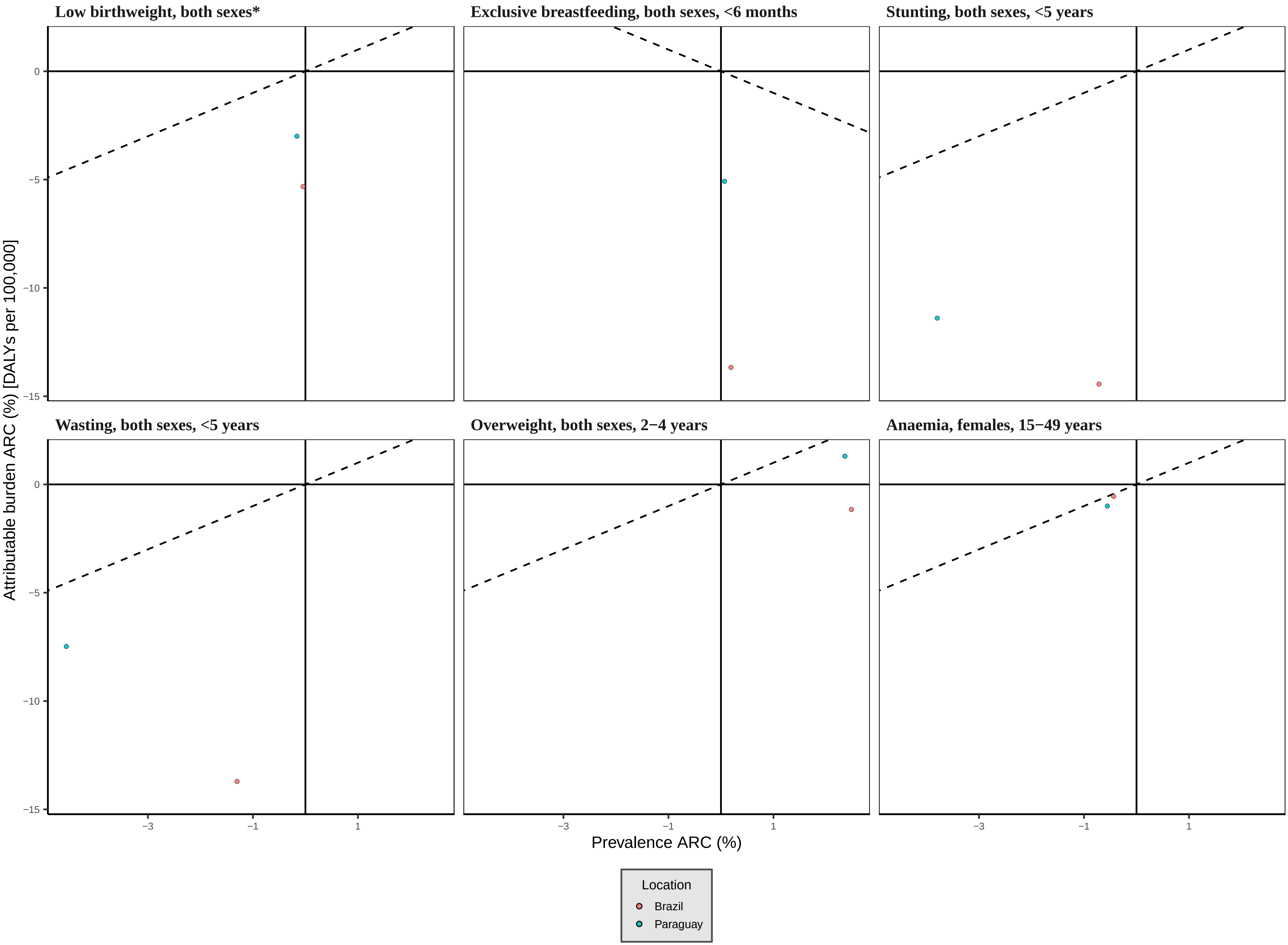

*Low birthweight prevalence ARC is at birth and attributable burden ARC is during neonatal age group (0 − 27 days). Exclusive breastfeeding line of unity is −1 due to the nature of the indicator.

Figure S9. Comparison of annual rates of change from 2012 to 2021: prevalence vs. attributable burden, North Africa and Middle East

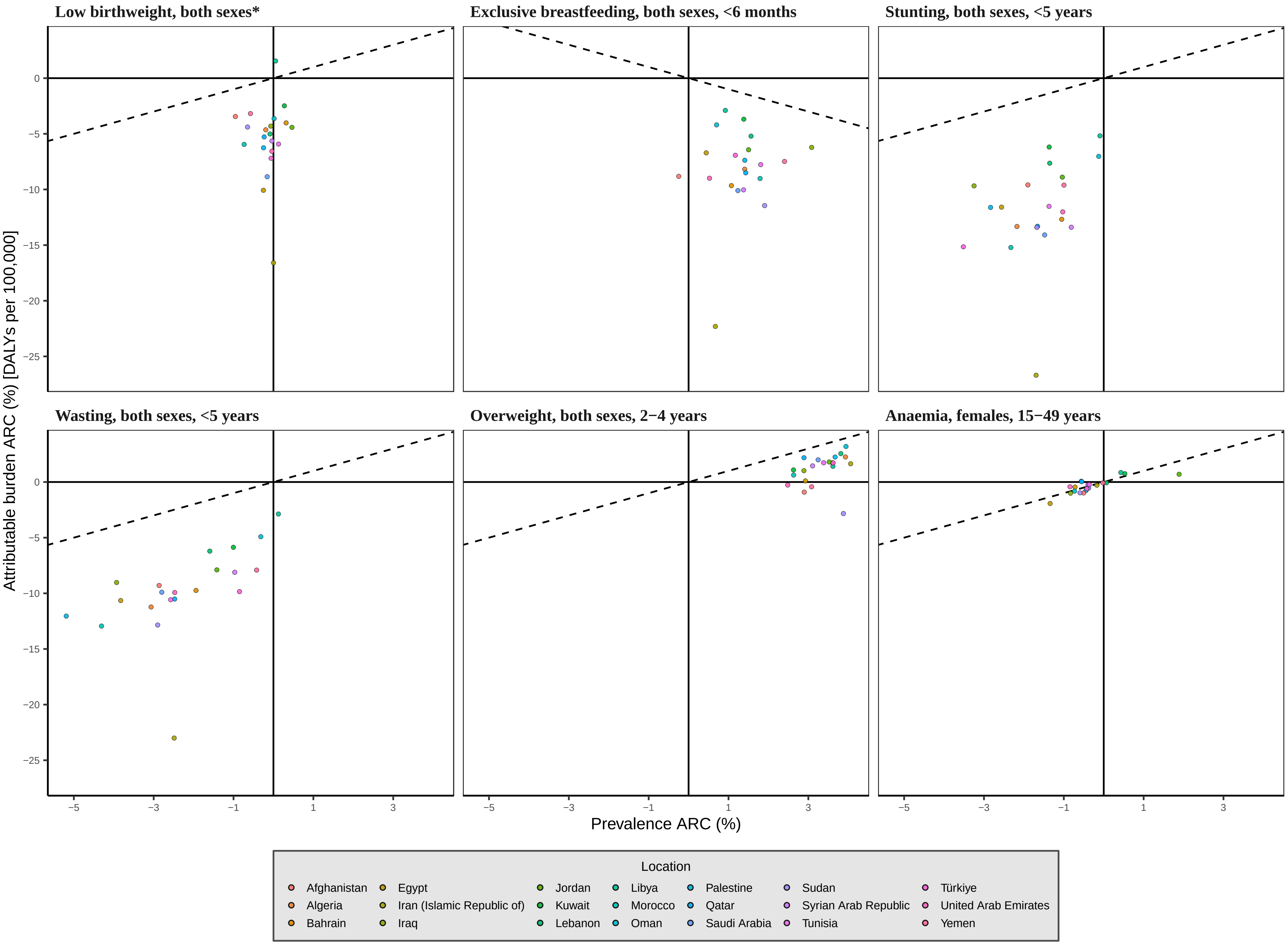

*Low birthweight prevalence ARC is at birth and attributable burden ARC is during neonatal age group (0 − 27 days). Exclusive breastfeeding line of unity is −1 due to the nature of the indicator.

Figure S9. Comparison of annual rates of change from 2012 to 2021: prevalence vs. attributable burden, South Asia

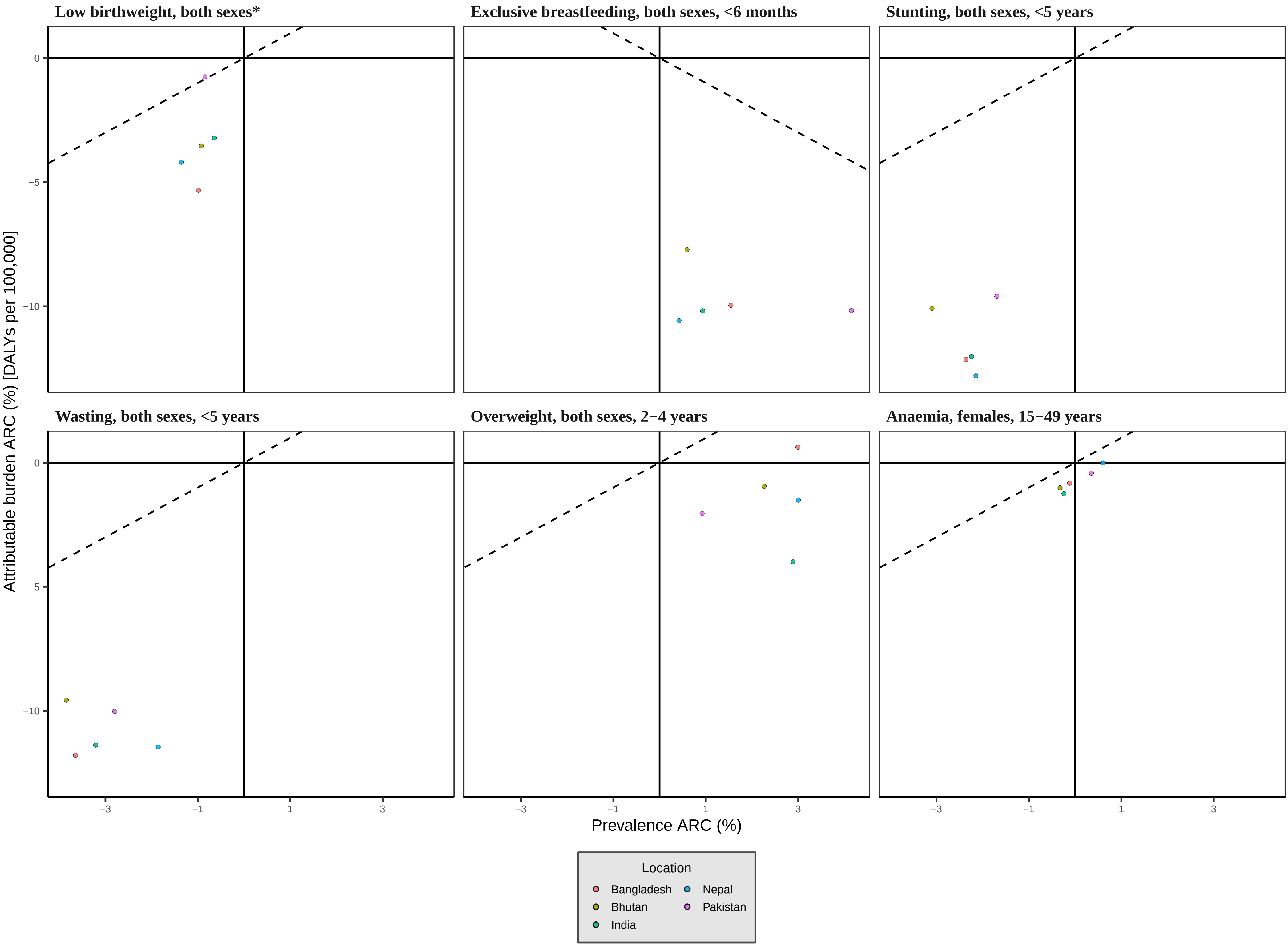

*Low birthweight prevalence ARC is at birth and attributable burden ARC is during neonatal age group (0 − 27 days). Exclusive breastfeeding line of unity is −1 due to the nature of the indicator.

Figure S9. Comparison of annual rates of change from 2012 to 2021: prevalence vs. attributable burden, East Asia

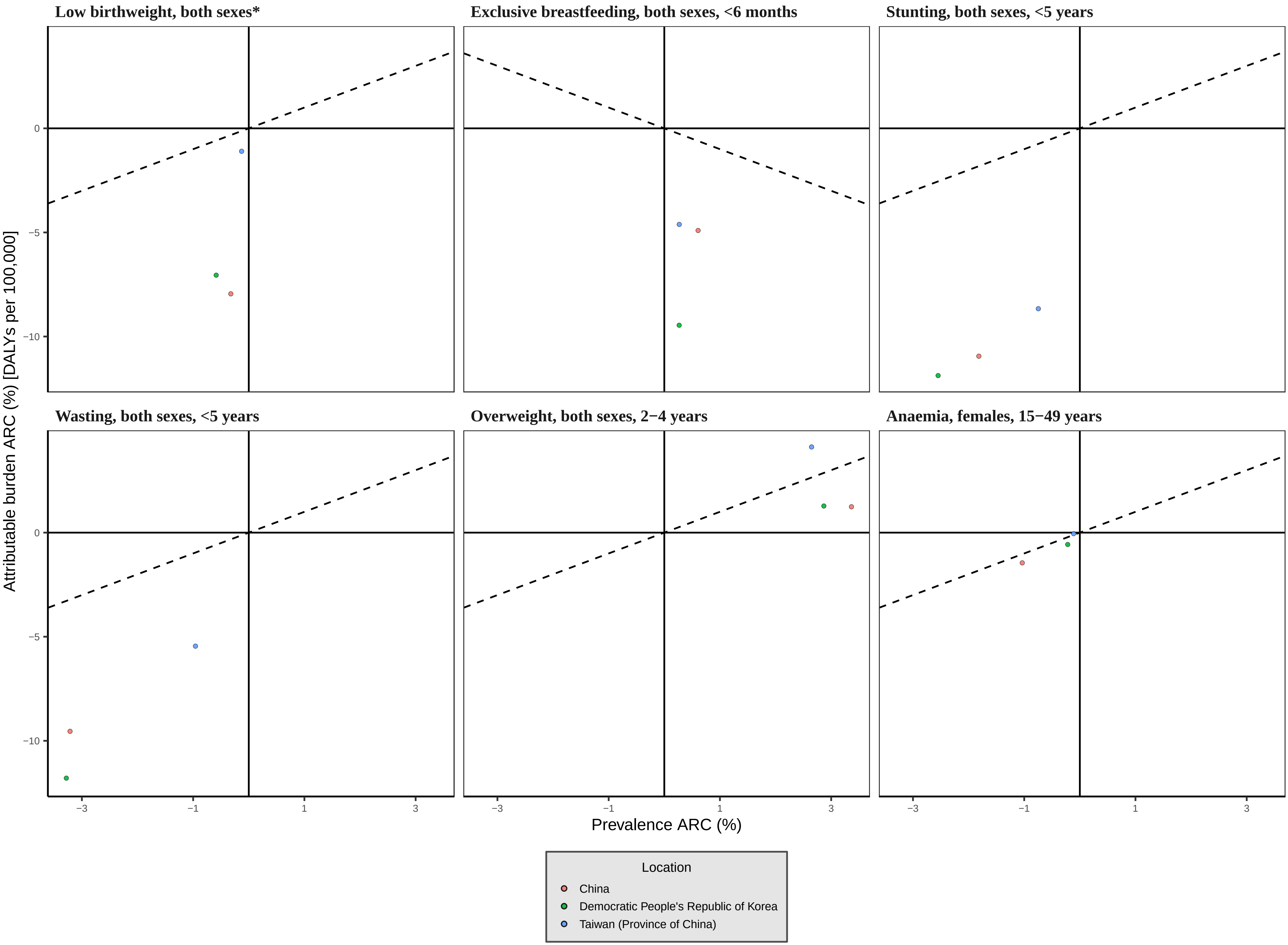

*Low birthweight prevalence ARC is at birth and attributable burden ARC is during neonatal age group (0 − 27 days). Exclusive breastfeeding line of unity is −1 due to the nature of the indicator.

Figure S9. Comparison of annual rates of change from 2012 to 2021: prevalence vs. attributable burden, Oceania

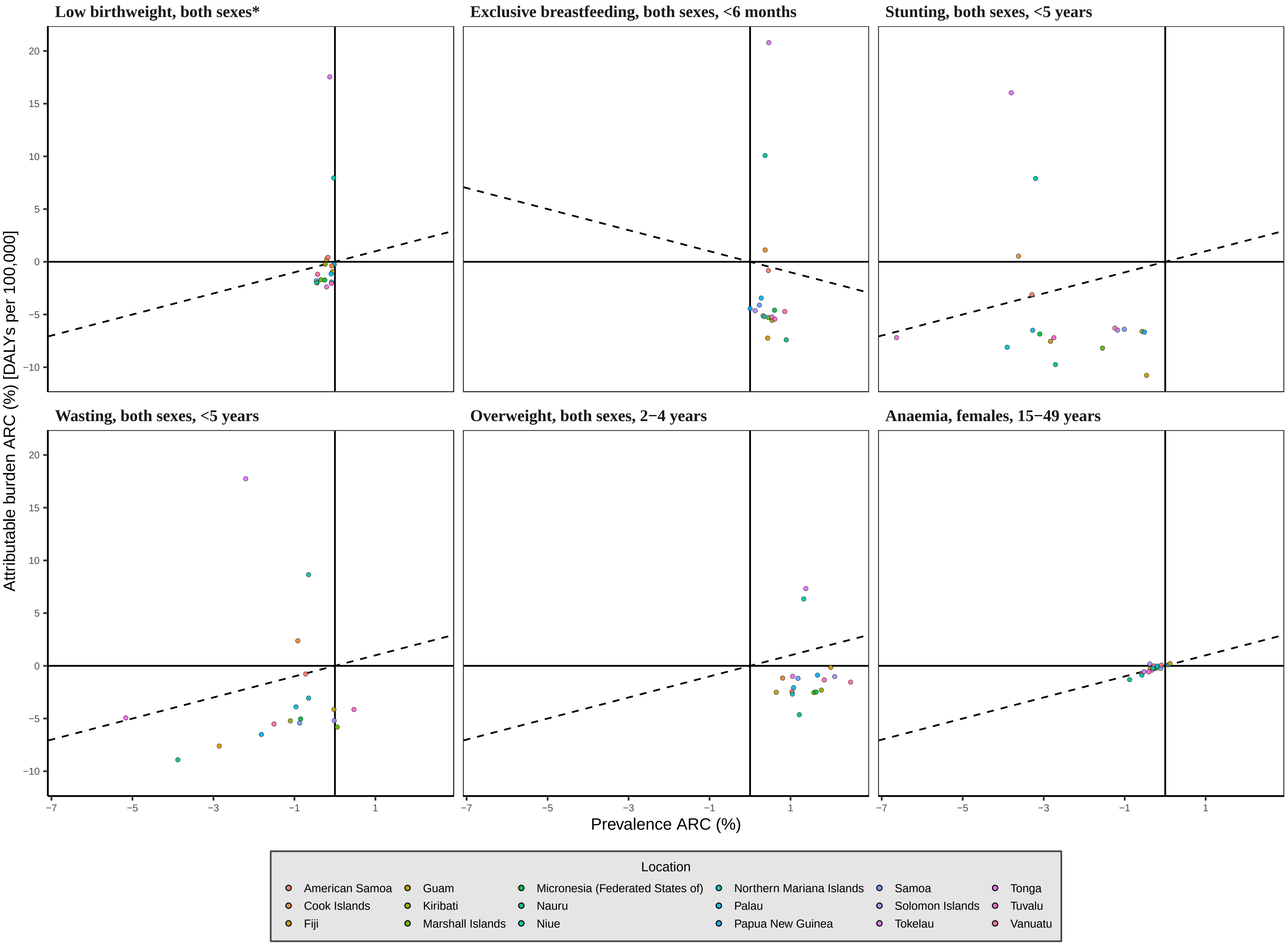

*Low birthweight prevalence ARC is at birth and attributable burden ARC is during neonatal age group (0 − 27 days). Exclusive breastfeeding line of unity is −1 due to the nature of the indicator.

Figure S9. Comparison of annual rates of change from 2012 to 2021: prevalence vs. attributable burden, Southeast Asia

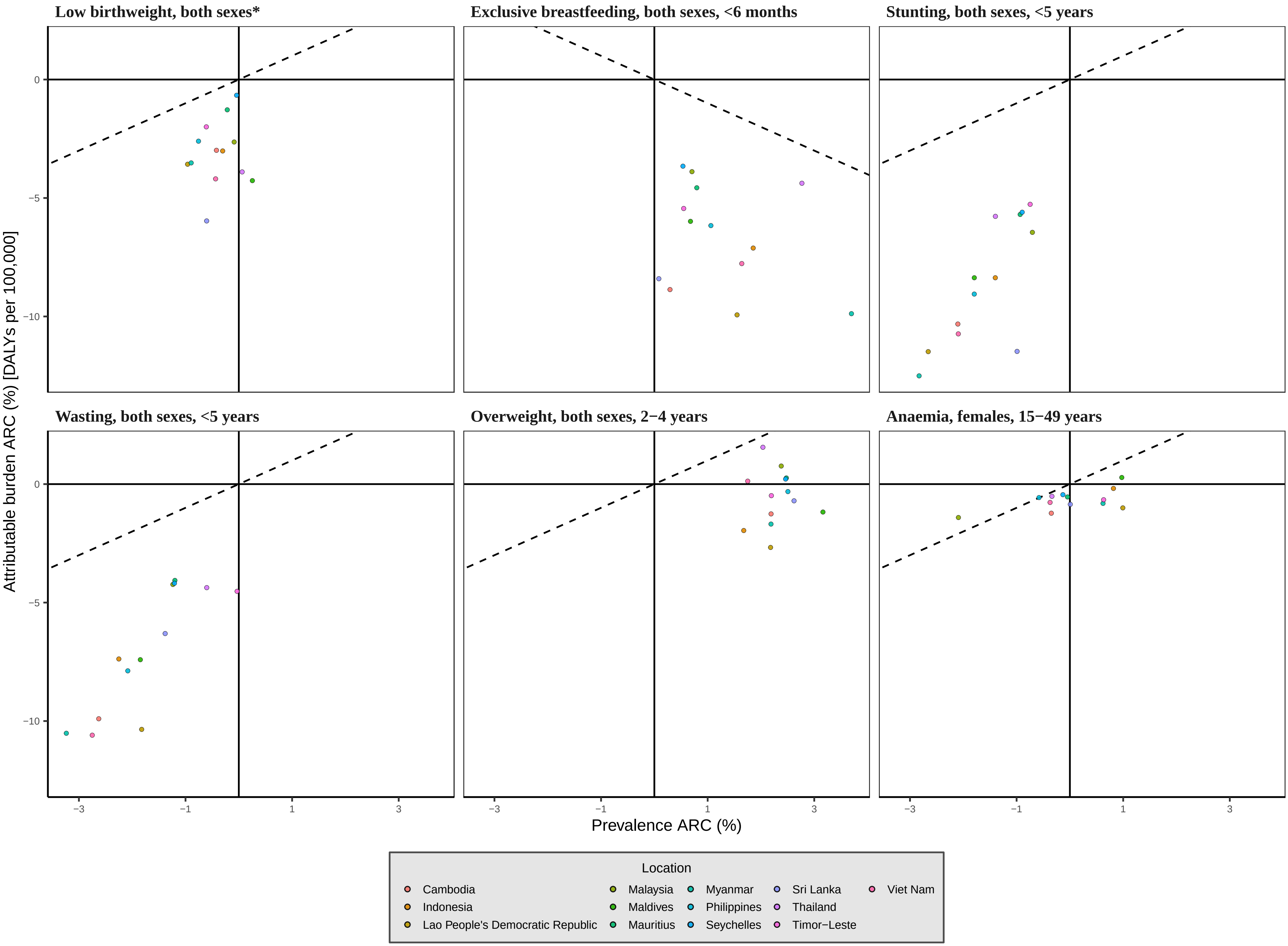

*Low birthweight prevalence ARC is at birth and attributable burden ARC is during neonatal age group (0 − 27 days). Exclusive breastfeeding line of unity is −1 due to the nature of the indicator.

Figure S9. Comparison of annual rates of change from 2012 to 2021: prevalence vs. attributable burden, Central Sub−Saharan Africa

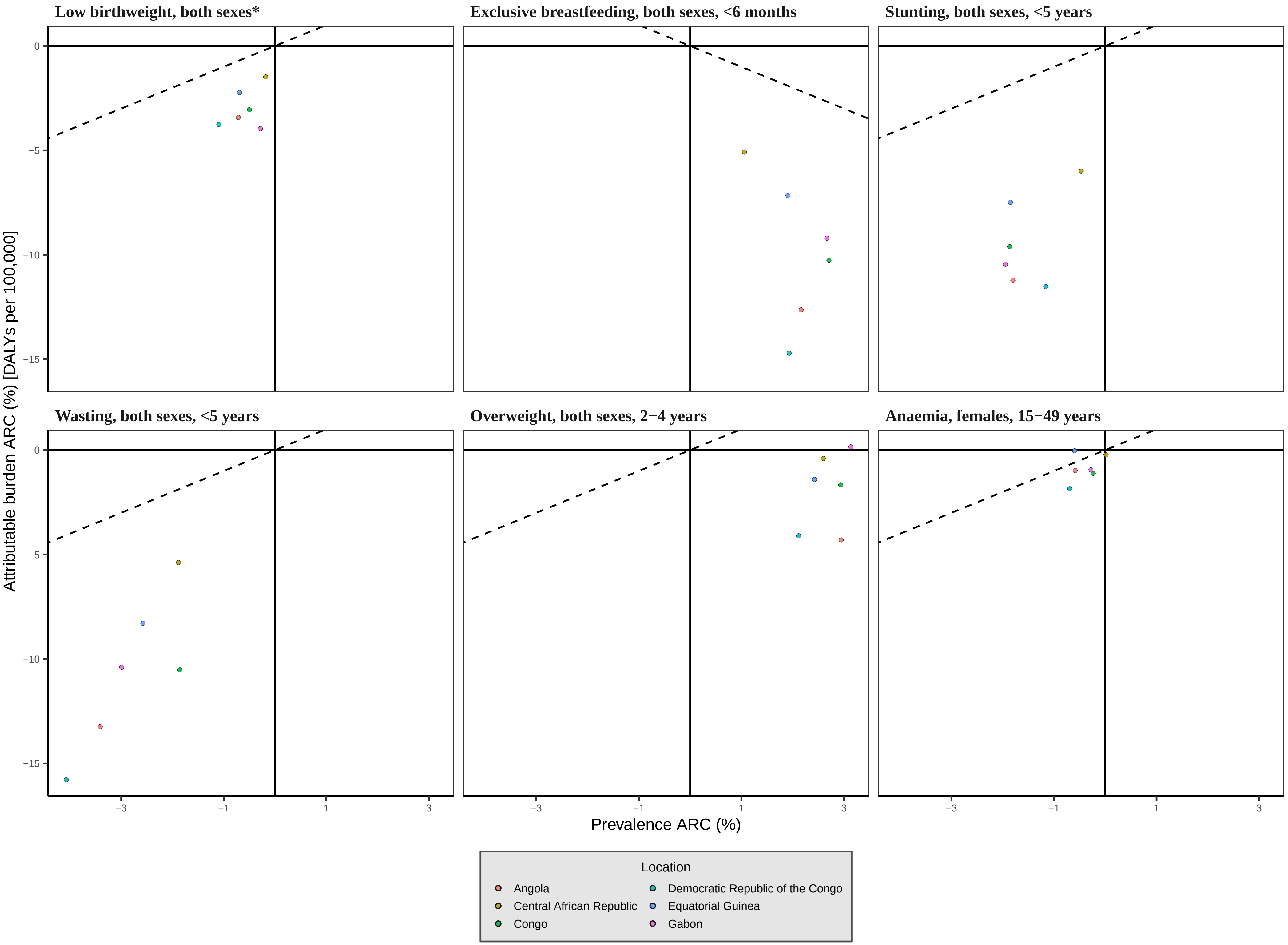

*Low birthweight prevalence ARC is at birth and attributable burden ARC is during neonatal age group (0 − 27 days). Exclusive breastfeeding line of unity is −1 due to the nature of the indicator.

Figure S9. Comparison of annual rates of change from 2012 to 2021: prevalence vs. attributable burden, Eastern Sub−Saharan Africa

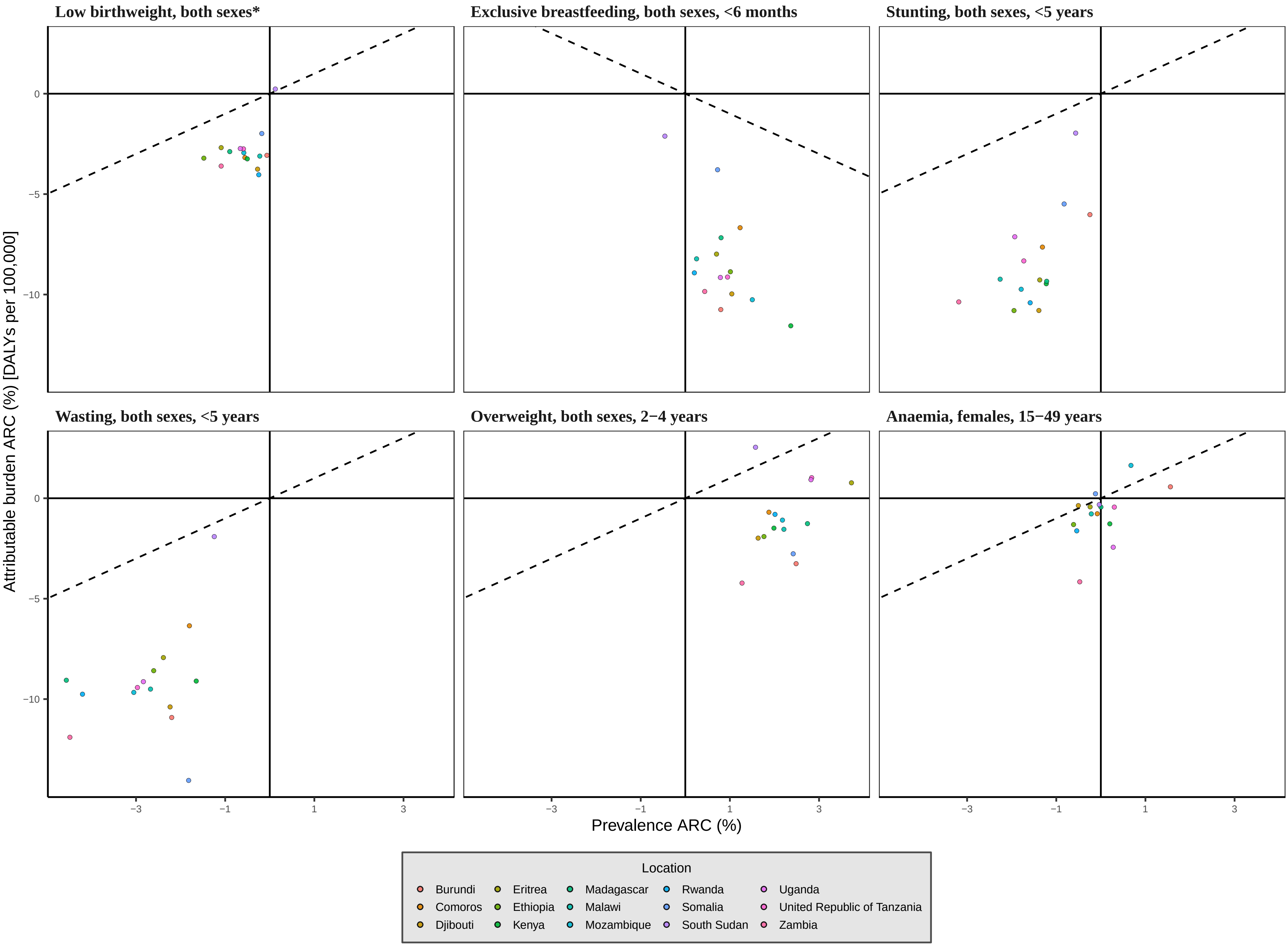

*Low birthweight prevalence ARC is at birth and attributable burden ARC is during neonatal age group (0 − 27 days). Exclusive breastfeeding line of unity is −1 due to the nature of the indicator.

Figure S9. Comparison of annual rates of change from 2012 to 2021: prevalence vs. attributable burden, Southern Sub−Saharan Africa

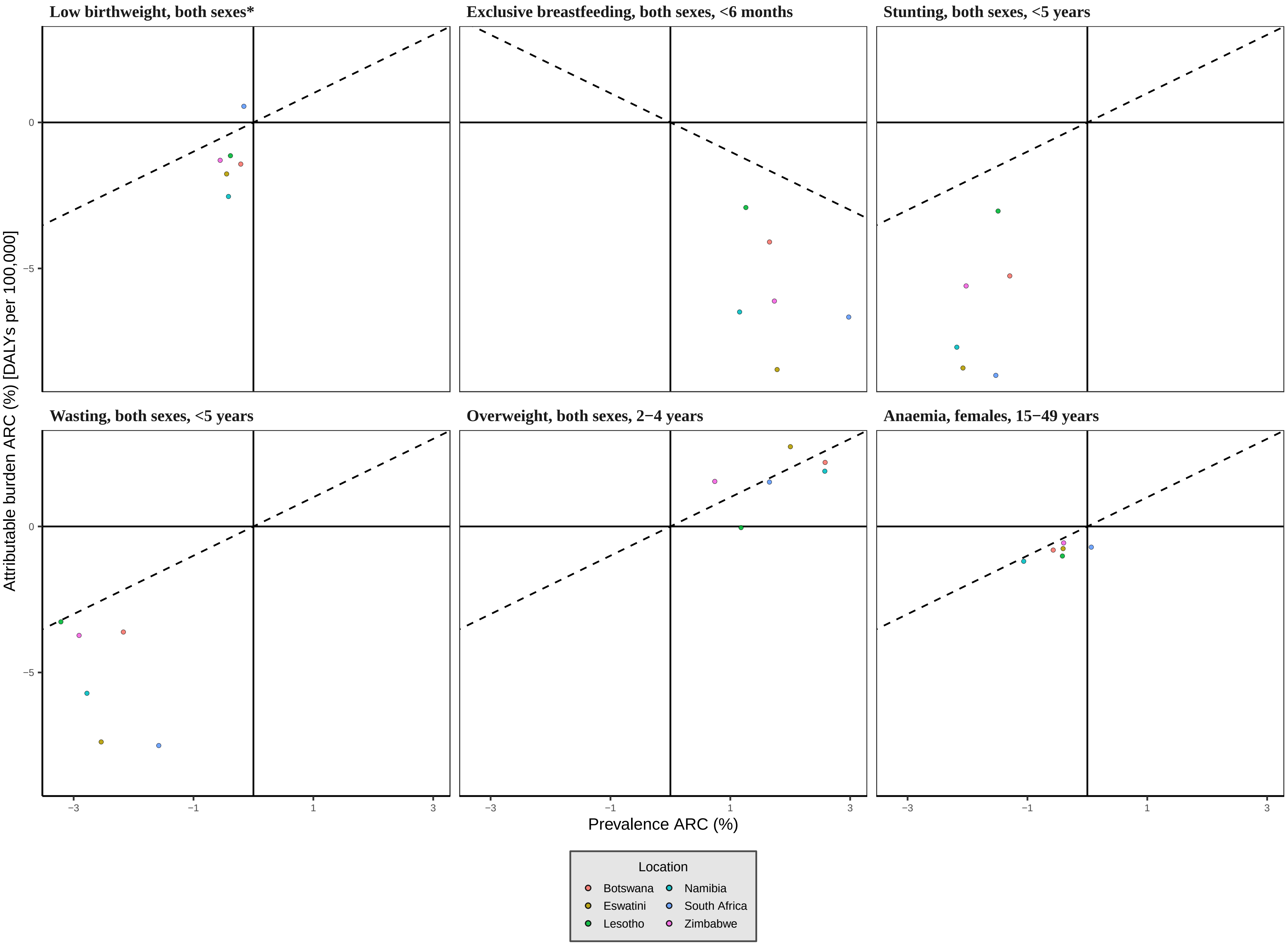

*Low birthweight prevalence ARC is at birth and attributable burden ARC is during neonatal age group (0 − 27 days). Exclusive breastfeeding line of unity is −1 due to the nature of the indicator.

Figure S9. Comparison of annual rates of change from 2012 to 2021: prevalence vs. attributable burden, Western Sub−Saharan Africa

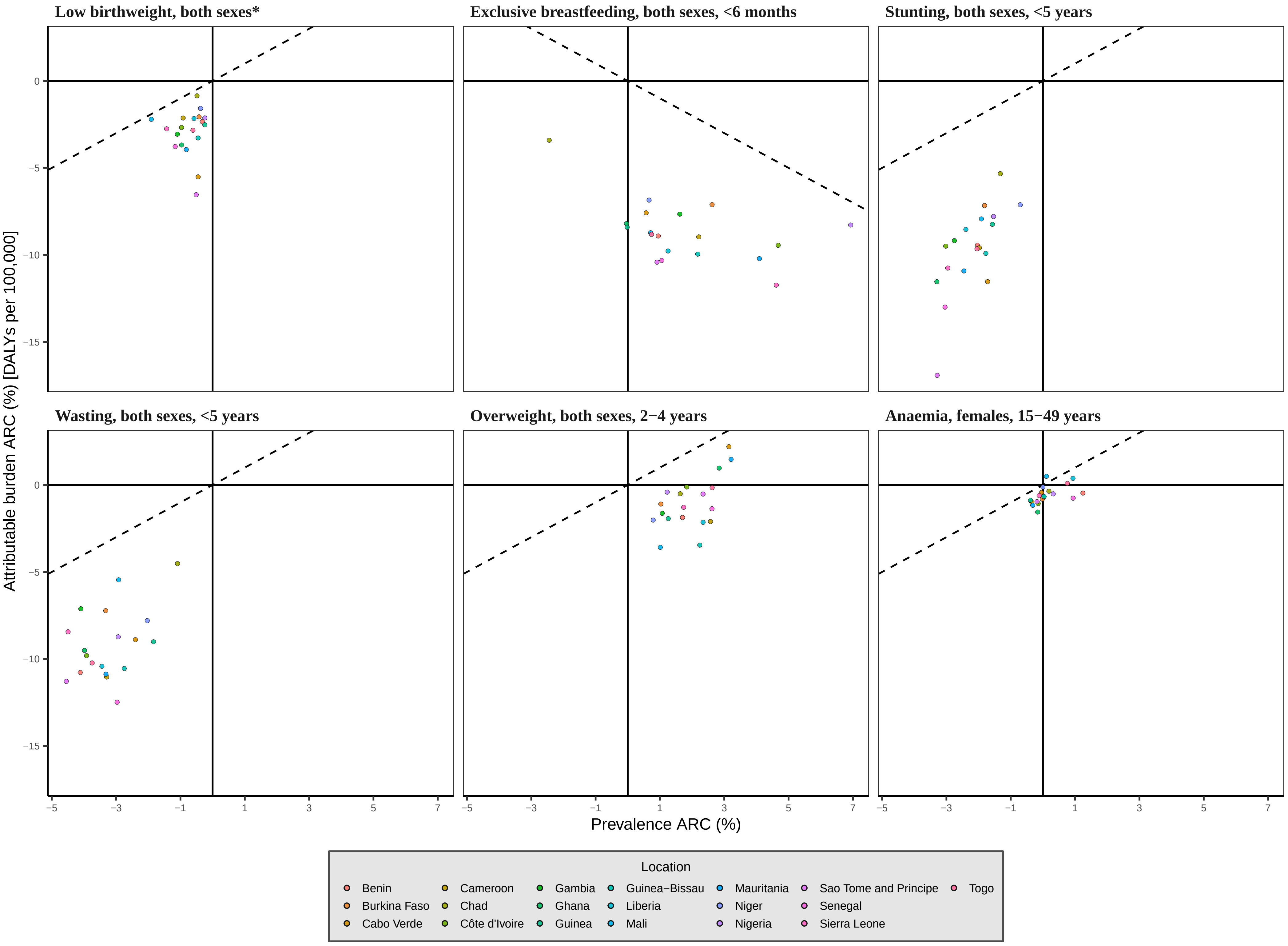

*Low birthweight prevalence ARC is at birth and attributable burden ARC is during neonatal age group (0 − 27 days). Exclusive breastfeeding line of unity is −1 due to the nature of the indicator.

Figure S10. Projected year of next target attainment (2031 to 2050)

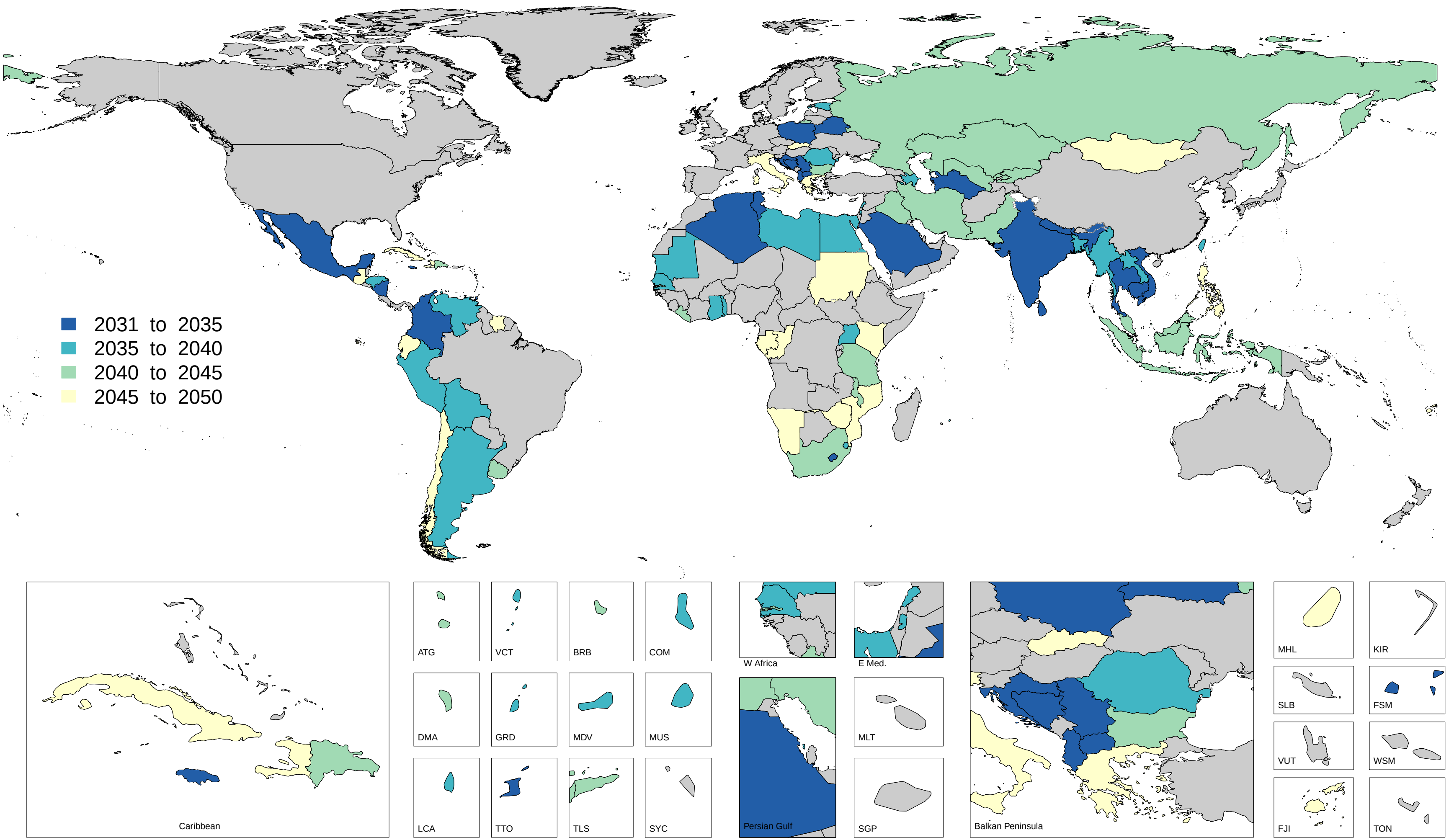

Figure S11. Projected number of targets not met by 2050

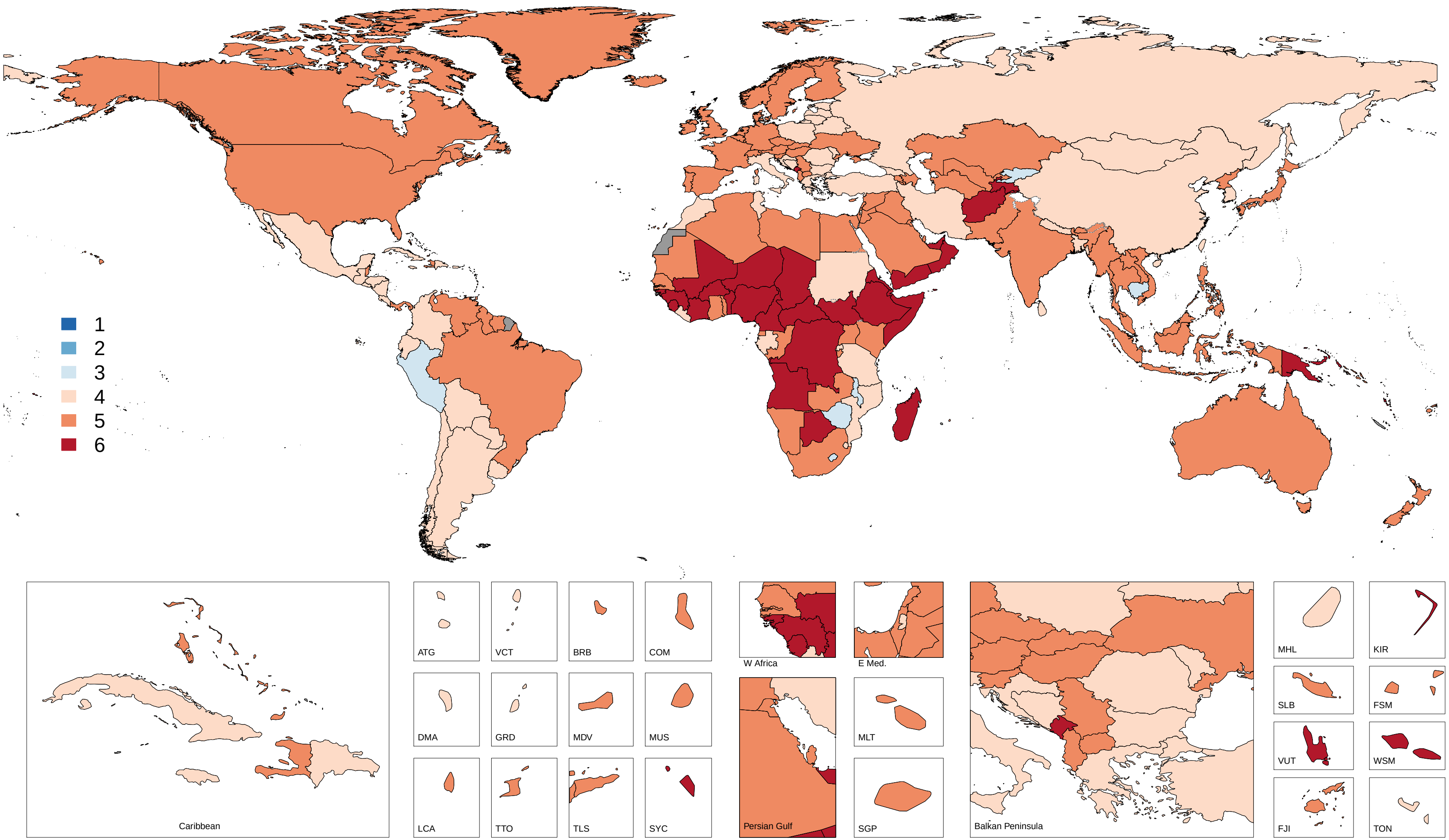

Figure S12. Ratio of projected 2050 population to estimated 2021 population, <5 years, both sexes

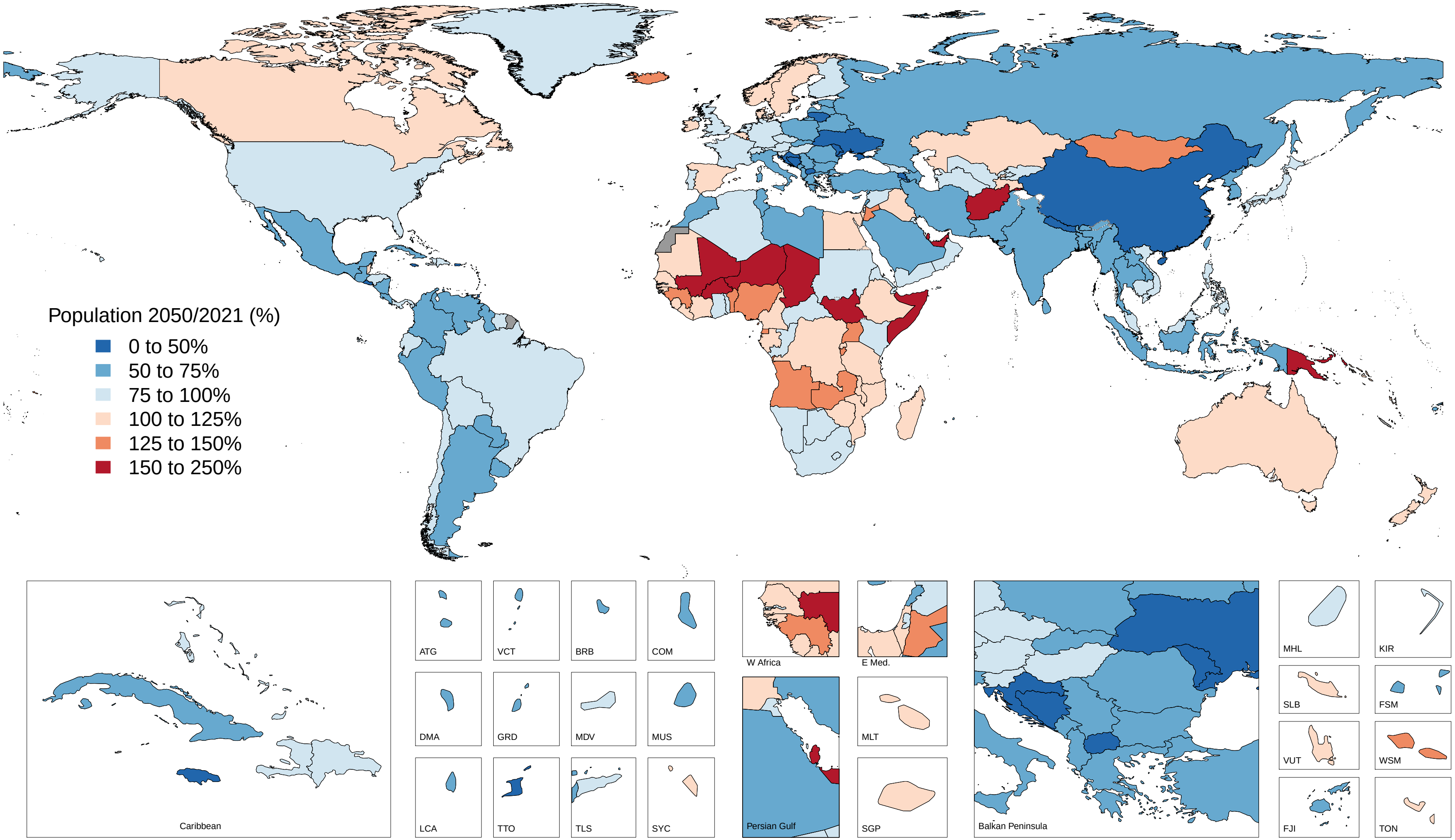

Table S1: Global, super−regional, regional, and country/territory nutrition indicator attributable disability−adjusted life years, 2012 and 2021

location_id	Year	Low birthweight, both sexes, <28 days [DALYs per 100,000]	Exclusive breastfeeding, both sexes, <6 months [DALYs per 100,000]	Stunting, both sexes, <5 years [DALYs per 100,000]	Wasting, both sexes, <5 years [DALYs per 100,000]	Overweight, both sexes, 2−4 years [DALYs per 100,000]	Anaemia, females, 15−49 years [DALYs per 100,000]
Global	2012	1674110 (1519714 − 1826438)	26926 (20246 − 34097)	8768 (6575 − 10523)	11605 (6370 − 14528)	25 (11 − 46)	937 (626 − 1357)
Global	2021	1355479 (1172075 − 1569179)	13726 (10067 − 17979)	4181 (2723 − 5773)	5326 (2845 − 7289)	25 (11 − 46)	927 (615 − 1343)
Central Europe, eastern Europe, and central Asia	2012	531096 (492495 − 568466)	7731 (5263 − 10355)	1757 (1099 − 2459)	1902 (1474 − 2320)	24 (9 − 47)	719 (475 − 1055)
Central Europe, eastern Europe, and central Asia	2021	402805 (349896 − 464245)	5413 (3551 − 7393)	788 (478 − 1123)	990 (715 − 1239)	27 (10 − 52)	707 (473 − 1032)
Central Asia	2012	932361 (827438 − 1041577)	17861 (11417 − 24840)	4637 (2897 − 6545)	5236 (3964 − 6468)	16 (6 − 31)	1338 (890 − 1984)
Central Asia	2021	714113 (603146 − 845265)	10633 (6510 − 15055)	1771 (1086 − 2552)	2259 (1605 − 2865)	16 (6 − 32)	1276 (839 − 1870)
Armenia	2012	544586 (461647 − 628447)	7131 (4483 − 10039)	1699 (957 − 2441)	1274 (878 − 1714)	19 (7 − 38)	507 (318 − 761)
Armenia	2021	398125 (325738 − 491541)	4876 (3166 − 6847)	935 (537 − 1397)	671 (444 − 957)	19 (7 − 39)	508 (307 − 756)
Azerbaijan	2012	1622192 (1321660 − 1938831)	31993 (21199 − 44235)	5449 (3326 − 8011)	6346 (4490 − 8351)	16 (7 − 33)	986 (586 − 1487)
Azerbaijan	2021	1224581 (980495 − 1473591)	20322 (13713 − 28150)	2305 (1390 − 3383)	2872 (1922 − 3815)	16 (6 − 33)	951 (569 − 1434)
Georgia	2012	707091 (579857 − 841286)	3130 (2156 − 4255)	613 (374 − 928)	422 (294 − 587)	20 (9 − 41)	713 (455 − 1058)
Georgia	2021	392522 (307591 − 498387)	1579 (1097 − 2161)	201 (111 − 316)	140 (92 − 197)	18 (7 − 37)	699 (453 − 1045)

Table S1: Global, super−regional, regional, and country/territory nutrition indicator attributable disability−adjusted life years, 2012 and 2021

location_id	Year	Low birthweight, both sexes, <28 days [DALYs per 100,000]	Exclusive breastfeeding, both sexes, <6 months [DALYs per 100,000]	Stunting, both sexes, <5 years [DALYs per 100,000]	Wasting, both sexes, <5 years [DALYs per 100,000]	Overweight, both sexes, 2−4 years [DALYs per 100,000]	Anaemia, females, 15−49 years [DALYs per 100,000]
Kazakhstan	2012	568431 (479962 − 672199)	4415 (2510 − 6513)	1075 (586 − 1567)	1248 (909 − 1622)	11 (4 − 23)	1089 (683 − 1636)
Kazakhstan	2021	296777 (247057 − 365238)	2177 (1266 − 3225)	250 (122 − 387)	372 (273 − 483)	11 (4 − 23)	986 (609 − 1479)
Kyrgyzstan	2012	688127 (610520 − 760170)	6806 (4674 − 9121)	2494 (1565 − 3404)	2373 (1781 − 2819)	13 (5 − 28)	1101 (700 − 1597)
Kyrgyzstan	2021	603907 (506702 − 708593)	3297 (2180 − 4773)	902 (585 − 1275)	783 (556 − 998)	14 (5 − 30)	1038 (667 − 1535)
Mongolia	2012	762725 (630884 − 894873)	8905 (4976 − 13380)	3316 (2135 − 4822)	2147 (1417 − 2945)	14 (5 − 28)	1233 (797 − 1825)
Mongolia	2021	516353 (420577 − 643919)	4215 (2165 − 6760)	941 (540 − 1493)	659 (428 − 1001)	12 (5 − 26)	1154 (691 − 1728)
Tajikistan	2012	1376654 (1184083 − 1602582)	38190 (25244 − 53923)	9898 (6387 − 14087)	14580 (7022 − 19639)	10 (4 − 21)	1233 (805 − 1791)
Tajikistan	2021	1124165 (915710 − 1357444)	25545 (15657 − 36806)	5254 (3202 − 7762)	8411 (4251 − 11809)	11 (4 − 22)	1165 (730 − 1689)
Turkmenistan	2012	935657 (812428 − 1069756)	11942 (6563 − 18428)	5416 (2872 − 8057)	6449 (4572 − 8548)	12 (4 − 26)	1082 (674 − 1644)
Turkmenistan	2021	738391 (598947 − 914064)	8073 (4283 − 12873)	2349 (1161 − 3566)	3125 (2135 − 4182)	13 (4 − 26)	1005 (607 − 1544)
Uzbekistan	2012	913169 (776374 − 1073141)	20669 (11076 − 30748)	5548 (3195 − 8076)	5246 (3466 − 7219)	20 (8 − 42)	1866 (1208 − 2754)
Uzbekistan	2021	766636 (614887 − 943549)	11280 (6138 − 17127)	1551 (926 − 2256)	1515 (964 − 2189)	22 (8 − 42)	1773 (1159 − 2635)

Table S1: Global, super−regional, regional, and country/territory nutrition indicator attributable disability−adjusted life years, 2012 and 2021

location_id	Year	Low birthweight, both sexes, <28 days [DALYs per 100,000]	Exclusive breastfeeding, both sexes, <6 months [DALYs per 100,000]	Stunting, both sexes, <5 years [DALYs per 100,000]	Wasting, both sexes, <5 years [DALYs per 100,000]	Overweight, both sexes, 2−4 years [DALYs per 100,000]	Anaemia, females, 15−49 years [DALYs per 100,000]
Central Europe	2012	263290 (253724 − 273815)	2911 (2274 − 3605)	373 (217 − 512)	361 (292 − 424)	41 (16 − 82)	486 (312 − 716)
Central Europe	2021	181031 (153730 − 208839)	2163 (1685 − 2644)	174 (100 − 250)	221 (126 − 304)	54 (21 − 106)	463 (298 − 684)
Albania	2012	657155 (521144 − 811924)	3769 (2132 − 6040)	1462 (885 − 2165)	1418 (1024 − 1905)	33 (13 − 66)	483 (306 − 750)
Albania	2021	512975 (390052 − 647938)	1778 (901 − 2943)	643 (379 − 1000)	602 (407 − 803)	32 (13 − 60)	473 (294 − 747)
Bosnia and Herzegovina	2012	278508 (239100 − 317448)	1546 (1040 − 2256)	116 (76 − 171)	152 (79 − 235)	52 (21 − 104)	528 (312 − 839)
Bosnia and Herzegovina	2021	255554 (201666 − 315325)	1250 (748 − 2266)	75 (45 − 115)	112 (51 − 189)	55 (23 − 108)	512 (310 − 789)
Bulgaria	2012	346113 (321453 − 372829)	5251 (4193 − 6408)	442 (242 − 653)	583 (468 − 700)	34 (13 − 69)	591 (345 − 892)
Bulgaria	2021	218296 (179948 − 258047)	3466 (2608 − 4426)	201 (111 − 319)	328 (211 − 439)	35 (14 − 73)	564 (335 − 891)
Croatia	2012	177857 (160878 − 196256)	1570 (1264 − 1937)	143 (96 − 199)	161 (106 − 203)	34 (13 − 73)	429 (262 − 660)
Croatia	2021	145808 (112130 − 184091)	1751 (1248 − 2339)	104 (64 − 157)	144 (66 − 226)	39 (15 − 81)	401 (250 − 635)
Czechia	2012	114985 (105959 − 124514)	2042 (1690 − 2432)	100 (60 − 144)	262 (143 − 368)	29 (12 − 61)	408 (246 − 639)
Czechia	2021	90925 (72565 − 111462)	2673 (2029 − 3471)	81 (46 − 122)	258 (94 − 438)	31 (12 − 63)	398 (237 − 648)

Table S1: Global, super−regional, regional, and country/territory nutrition indicator attributable disability−adjusted life years, 2012 and 2021

location_id	Year	Low birthweight, both sexes, <28 days [DALYs per 100,000]	Exclusive breastfeeding, both sexes, <6 months [DALYs per 100,000]	Stunting, both sexes, <5 years [DALYs per 100,000]	Wasting, both sexes, <5 years [DALYs per 100,000]	Overweight, both sexes, 2−4 years [DALYs per 100,000]	Anaemia, females, 15−49 years [DALYs per 100,000]
Hungary	2012	229594 (213679 − 245978)	2358 (1933 − 2813)	172 (109 − 240)	279 (98 − 444)	30 (12 − 60)	465 (264 − 718)
Hungary	2021	134537 (104091 − 167102)	3024 (2216 − 3900)	123 (75 − 184)	270 (45 − 519)	32 (14 − 70)	440 (248 − 692)
Montenegro	2012	254126 (223532 − 286112)	1010 (632 − 1479)	147 (91 − 222)	154 (108 − 210)	43 (18 − 87)	489 (295 − 737)
Montenegro	2021	124103 (98058 − 148831)	363 (212 − 566)	35 (18 − 59)	49 (32 − 68)	48 (19 − 97)	463 (282 − 717)
North Macedonia	2012	553169 (471142 − 627775)	4742 (3384 − 6441)	319 (192 − 462)	652 (207 − 1054)	41 (16 − 85)	574 (353 − 899)
North Macedonia	2021	274220 (231489 − 320628)	2850 (1762 − 4354)	114 (63 − 175)	283 (69 − 510)	44 (18 − 90)	570 (350 − 900)
Poland	2012	215951 (204000 − 228305)	739 (577 − 900)	102 (65 − 142)	105 (83 − 126)	56 (21 − 114)	477 (290 − 724)
Poland	2021	151782 (120528 − 190966)	1044 (784 − 1352)	71 (43 − 107)	101 (44 − 157)	89 (33 − 184)	460 (278 − 714)
Romania	2012	357363 (335070 − 380637)	8423 (5996 − 11222)	1159 (613 − 1625)	850 (651 − 1079)	36 (14 − 73)	524 (327 − 842)
Romania	2021	237083 (211191 − 264203)	4267 (3021 − 5563)	454 (239 − 664)	396 (284 − 514)	39 (16 − 77)	489 (294 − 750)
Serbia	2012	295757 (253200 − 347664)	1071 (741 − 1463)	87 (52 − 133)	141 (94 − 188)	29 (11 − 57)	501 (300 − 771)
Serbia	2021	222457 (190211 − 254926)	838 (555 − 1204)	57 (32 − 87)	103 (58 − 152)	30 (12 − 60)	471 (273 − 734)

Table S1: Global, super−regional, regional, and country/territory nutrition indicator attributable disability−adjusted life years, 2012 and 2021

location_id	Year	Low birthweight, both sexes, <28 days [DALYs per 100,000]	Exclusive breastfeeding, both sexes, <6 months [DALYs per 100,000]	Stunting, both sexes, <5 years [DALYs per 100,000]	Wasting, both sexes, <5 years [DALYs per 100,000]	Overweight, both sexes, 2−4 years [DALYs per 100,000]	Anaemia, females, 15−49 years [DALYs per 100,000]
Slovakia	2012	220792 (198426 − 244857)	2261 (1608 − 3056)	493 (272 − 712)	459 (339 − 588)	24 (9 − 48)	456 (270 − 738)
Slovakia	2021	174360 (143013 − 208123)	1537 (1000 − 2158)	265 (132 − 412)	281 (202 − 371)	25 (9 − 52)	441 (247 − 710)
Slovenia	2012	97627 (88340 − 108297)	901 (664 − 1171)	121 (81 − 165)	105 (79 − 132)	46 (17 − 94)	384 (234 − 640)
Slovenia	2021	60560 (51559 − 70713)	847 (602 − 1133)	70 (45 − 99)	71 (51 − 92)	51 (20 − 101)	363 (219 − 585)
Eastern Europe	2012	358538 (340518 − 375865)	2584 (2054 − 3090)	446 (275 − 599)	354 (290 − 426)	20 (8 − 39)	572 (376 − 850)
Eastern Europe	2021	179008 (161135 − 199299)	1444 (1174 − 1737)	156 (94 − 210)	160 (132 − 190)	21 (8 − 43)	550 (370 − 826)
Belarus	2012	203161 (168953 − 242619)	1265 (924 − 1677)	132 (77 − 201)	139 (102 − 185)	17 (7 − 35)	539 (324 − 840)
Belarus	2021	105076 (80773 − 133581)	969 (702 − 1288)	60 (33 − 96)	86 (60 − 115)	21 (8 − 42)	509 (298 − 789)
Estonia	2012	91967 (82442 − 101560)	1598 (1227 − 2020)	166 (110 − 228)	129 (93 − 167)	16 (6 − 35)	475 (287 − 708)
Estonia	2021	47410 (40676 − 55226)	713 (553 − 891)	46 (32 − 63)	54 (42 − 66)	17 (6 − 35)	454 (281 − 705)
Latvia	2012	213291 (193415 − 233512)	1473 (1006 − 2026)	209 (124 − 296)	172 (122 − 223)	16 (5 − 33)	549 (346 − 845)
Latvia	2021	118268 (100278 − 137095)	589 (422 − 792)	52 (32 − 75)	65 (50 − 81)	17 (6 − 34)	512 (311 − 794)

Table S1: Global, super−regional, regional, and country/territory nutrition indicator attributable disability−adjusted life years, 2012 and 2021

location_id	Year	Low birthweight, both sexes, <28 days [DALYs per 100,000]	Exclusive breastfeeding, both sexes, <6 months [DALYs per 100,000]	Stunting, both sexes, <5 years [DALYs per 100,000]	Wasting, both sexes, <5 years [DALYs per 100,000]	Overweight, both sexes, 2−4 years [DALYs per 100,000]	Anaemia, females, 15−49 years [DALYs per 100,000]
Lithuania	2012	130858 (119392 − 144501)	2298 (1800 − 2806)	181 (111 − 257)	153 (116 − 192)	13 (5 − 30)	550 (332 − 857)
Lithuania	2021	86472 (75143 − 98302)	1269 (987 − 1582)	55 (35 − 81)	66 (52 − 83)	15 (6 − 33)	503 (307 − 796)
Moldova	2012	549466 (468017 − 635637)	5190 (3663 − 6976)	543 (332 − 795)	519 (370 − 681)	8 (3 − 16)	603 (393 − 920)
Moldova	2021	334831 (246281 − 445483)	3936 (2709 − 5511)	260 (144 − 407)	298 (205 − 423)	9 (3 − 20)	567 (355 − 879)
Russia	2012	378349 (360248 − 397698)	2969 (2359 − 3557)	527 (323 − 715)	320 (256 − 389)	21 (8 − 43)	557 (350 − 857)
Russia	2021	175237 (155991 − 194015)	1476 (1187 − 1768)	158 (94 − 218)	113 (90 − 134)	23 (9 − 45)	527 (338 − 812)
Ukraine	2012	331282 (307855 − 355516)	1324 (963 − 1697)	250 (165 − 326)	534 (421 − 681)	17 (6 − 35)	629 (402 − 935)
Ukraine	2021	221908 (180633 − 267718)	1277 (898 − 1723)	182 (109 − 257)	412 (311 − 526)	19 (7 − 40)	642 (411 − 961)
High income	2012	211460 (206512 − 216474)	780 (660 − 893)	59 (42 − 77)	78 (53 − 104)	38 (16 − 71)	149 (93 − 233)
High income	2021	171220 (152363 − 189894)	434 (357 − 512)	25 (18 − 33)	41 (25 − 61)	42 (16 − 81)	150 (91 − 235)
Australasia	2012	160397 (152178 − 168739)	413 (334 − 499)	28 (17 − 41)	31 (19 − 47)	64 (28 − 119)	143 (85 − 227)
Australasia	2021	123936 (104627 − 146509)	188 (143 − 239)	9 (6 − 14)	13 (7 − 22)	68 (29 − 135)	141 (84 − 221)

Table S1: Global, super−regional, regional, and country/territory nutrition indicator attributable disability−adjusted life years, 2012 and 2021

location_id	Year	Low birthweight, both sexes, <28 days [DALYs per 100,000]	Exclusive breastfeeding, both sexes, <6 months [DALYs per 100,000]	Stunting, both sexes, <5 years [DALYs per 100,000]	Wasting, both sexes, <5 years [DALYs per 100,000]	Overweight, both sexes, 2−4 years [DALYs per 100,000]	Anaemia, females, 15−49 years [DALYs per 100,000]
Australia	2012	150746 (142018 − 160347)	341 (271 − 419)	20 (12 − 29)	25 (17 − 35)	62 (26 − 116)	139 (81 − 227)
Australia	2021	113881 (94714 − 136057)	153 (112 − 205)	7 (4 − 11)	11 (7 − 17)	67 (27 − 132)	139 (79 − 220)
New Zealand	2012	209830 (193746 − 225197)	783 (619 − 948)	66 (39 − 96)	62 (31 − 103)	73 (29 − 145)	162 (92 − 254)
New Zealand	2021	172359 (147514 − 195819)	355 (275 − 456)	22 (14 − 33)	25 (9 − 49)	77 (31 − 152)	152 (92 − 238)
High−income Asia Pacific	2012	76210 (71810 − 80512)	598 (494 − 700)	77 (62 − 91)	91 (55 − 121)	16 (6 − 31)	145 (82 − 242)
High−income Asia Pacific	2021	46680 (42041 − 51770)	318 (260 − 389)	30 (16 − 39)	43 (20 − 66)	19 (8 − 38)	148 (84 − 259)
Brunei	2012	351800 (305572 − 405401)	1262 (844 − 1805)	297 (189 − 416)	179 (132 − 232)	8 (3 − 17)	195 (110 − 321)
Brunei	2021	344763 (275452 − 437602)	778 (474 − 1185)	156 (93 − 235)	105 (69 − 146)	9 (4 − 20)	203 (111 − 346)
Japan	2012	56590 (53442 − 59959)	606 (498 − 717)	96 (77 − 112)	113 (68 − 148)	15 (5 − 30)	148 (79 − 254)
Japan	2021	36530 (31051 − 42060)	287 (228 − 357)	36 (17 − 51)	51 (24 − 77)	19 (7 − 39)	156 (82 − 281)
Singapore	2012	61816 (54672 − 69554)	1028 (796 − 1276)	99 (66 − 140)	87 (64 − 110)	13 (5 − 27)	140 (39 − 325)
Singapore	2021	47868 (38433 − 59106)	464 (344 − 606)	27 (18 − 39)	27 (20 − 35)	17 (6 − 35)	134 (36 − 340)

Table S1: Global, super−regional, regional, and country/territory nutrition indicator attributable disability−adjusted life years, 2012 and 2021

location_id	Year	Low birthweight, both sexes, <28 days [DALYs per 100,000]	Exclusive breastfeeding, both sexes, <6 months [DALYs per 100,000]	Stunting, both sexes, <5 years [DALYs per 100,000]	Wasting, both sexes, <5 years [DALYs per 100,000]	Overweight, both sexes, 2−4 years [DALYs per 100,000]	Anaemia, females, 15−49 years [DALYs per 100,000]
South Korea	2012	119165 (105676 − 130853)	516 (389 − 673)	26 (18 − 36)	40 (21 − 62)	18 (7 − 38)	139 (75 − 236)
South Korea	2021	72271 (53104 − 92029)	376 (242 − 597)	9 (6 − 14)	22 (7 − 45)	20 (8 − 40)	132 (76 − 214)
High−income North America	2012	275652 (268038 − 283092)	663 (561 − 755)	39 (23 − 56)	44 (30 − 60)	56 (23 − 108)	204 (128 − 317)
High−income North America	2021	222935 (197996 − 247366)	400 (327 − 477)	22 (12 − 32)	34 (24 − 46)	59 (23 − 112)	201 (121 − 322)
Canada	2012	214723 (202353 − 227635)	1074 (903 − 1272)	23 (14 − 34)	64 (21 − 115)	65 (26 − 140)	105 (42 − 238)
Canada	2021	167135 (138515 − 201905)	544 (415 − 696)	9 (5 − 13)	33 (12 − 61)	64 (25 − 129)	108 (39 − 240)
Greenland	2012	446640 (376570 − 529631)	1332 (935 − 1843)	206 (118 − 301)	152 (106 − 201)	60 (24 − 122)	190 (105 − 328)
Greenland	2021	327155 (259072 − 390871)	732 (470 − 1053)	83 (48 − 125)	74 (47 − 115)	59 (23 − 117)	175 (101 − 299)
USA	2012	281515 (273719 − 289245)	623 (522 − 715)	41 (23 − 59)	42 (30 − 55)	55 (23 − 106)	215 (135 − 326)
USA	2021	228500 (203632 − 253971)	385 (308 − 468)	23 (13 − 34)	34 (25 − 44)	58 (22 − 113)	212 (127 − 334)
Southern Latin America	2012	437921 (417645 − 458875)	2726 (2121 − 3364)	311 (195 − 421)	387 (324 − 451)	42 (17 − 84)	143 (63 − 275)
Southern Latin America	2021	337764 (265738 − 424622)	1208 (847 − 1617)	113 (70 − 162)	157 (116 − 210)	47 (19 − 96)	141 (56 − 282)

Table S1: Global, super−regional, regional, and country/territory nutrition indicator attributable disability−adjusted life years, 2012 and 2021

location_id	Year	Low birthweight, both sexes, <28 days [DALYs per 100,000]	Exclusive breastfeeding, both sexes, <6 months [DALYs per 100,000]	Stunting, both sexes, <5 years [DALYs per 100,000]	Wasting, both sexes, <5 years [DALYs per 100,000]	Overweight, both sexes, 2−4 years [DALYs per 100,000]	Anaemia, females, 15−49 years [DALYs per 100,000]
Argentina	2012	488597 (463274 − 516835)	3278 (2538 − 4080)	387 (241 − 524)	422 (351 − 493)	39 (15 − 77)	150 (48 − 327)
Argentina	2021	387960 (299096 − 498825)	1457 (999 − 1995)	142 (88 − 205)	170 (124 − 227)	44 (18 − 91)	152 (42 − 345)
Chile	2012	302873 (286268 − 321092)	1055 (815 − 1296)	76 (47 − 109)	261 (229 − 307)	51 (21 − 104)	129 (72 − 226)
Chile	2021	224520 (191701 − 260790)	510 (371 − 656)	26 (17 − 38)	114 (87 − 152)	58 (24 − 116)	117 (61 − 208)
Uruguay	2012	357458 (326146 − 387304)	2914 (2217 − 3679)	395 (238 − 547)	507 (374 − 654)	37 (14 − 77)	133 (48 − 283)
Uruguay	2021	228045 (178214 − 286560)	1459 (1014 − 1993)	150 (90 − 225)	205 (125 − 310)	39 (16 − 79)	124 (47 − 258)
Western Europe	2012	149121 (145217 − 152883)	541 (467 − 615)	18 (13 − 22)	41 (17 − 67)	25 (10 − 48)	106 (64 − 166)
Western Europe	2021	128956 (113353 − 144516)	378 (312 − 447)	9 (6 − 12)	27 (9 − 48)	29 (12 − 58)	107 (62 − 166)
Andorra	2012	66795 (56615 − 78793)	179 (123 − 256)	12 (7 − 19)	14 (8 − 23)	29 (11 − 63)	100 (56 − 175)
Andorra	2021	26627 (14592 − 38346)	73 (46 − 108)	4 (0 − 9)	6 (1 − 12)	32 (13 − 68)	100 (55 − 166)
Austria	2012	139900 (128570 − 151146)	323 (265 − 395)	12 (6 − 18)	23 (6 − 40)	23 (9 − 47)	103 (58 − 168)
Austria	2021	121060 (102446 − 138846)	190 (145 − 237)	6 (2 − 9)	11 (2 − 20)	24 (9 − 50)	98 (55 − 161)

Table S1: Global, super−regional, regional, and country/territory nutrition indicator attributable disability−adjusted life years, 2012 and 2021

location_id	Year	Low birthweight, both sexes, <28 days [DALYs per 100,000]	Exclusive breastfeeding, both sexes, <6 months [DALYs per 100,000]	Stunting, both sexes, <5 years [DALYs per 100,000]	Wasting, both sexes, <5 years [DALYs per 100,000]	Overweight, both sexes, 2−4 years [DALYs per 100,000]	Anaemia, females, 15−49 years [DALYs per 100,000]
Belgium	2012	143701 (133070 − 154699)	958 (776 − 1157)	30 (20 − 42)	81 (25 − 144)	18 (7 − 37)	122 (73 − 195)
Belgium	2021	133240 (106921 − 162367)	798 (603 − 1041)	17 (11 − 25)	55 (13 − 112)	19 (8 − 41)	117 (68 − 186)
Cyprus	2012	115379 (100819 − 130368)	510 (353 − 702)	19 (10 − 27)	56 (9 − 109)	25 (10 − 51)	105 (57 − 187)
Cyprus	2021	80343 (65436 − 98118)	325 (198 − 538)	10 (4 − 16)	34 (3 − 78)	30 (12 − 63)	106 (56 − 187)
Denmark	2012	170969 (155088 − 186077)	617 (494 − 762)	21 (14 − 27)	53 (12 − 96)	31 (12 − 62)	103 (52 − 177)
Denmark	2021	149316 (122480 − 178075)	481 (364 − 608)	14 (7 − 19)	42 (7 − 85)	29 (11 − 59)	97 (52 − 163)
Finland	2012	79946 (71932 − 88920)	361 (285 − 454)	18 (13 − 24)	32 (15 − 48)	30 (12 − 61)	106 (58 − 168)
Finland	2021	66729 (56149 − 78031)	226 (171 − 288)	9 (6 − 12)	18 (6 − 31)	32 (13 − 66)	103 (56 − 167)
France	2012	143455 (136003 − 151189)	642 (529 − 766)	18 (10 − 24)	57 (26 − 88)	21 (8 − 43)	124 (73 − 190)
France	2021	145077 (116937 − 175220)	472 (358 − 605)	12 (6 − 16)	45 (20 − 75)	27 (10 − 55)	123 (75 − 191)
Germany	2012	139140 (131345 − 146126)	440 (357 − 533)	16 (9 − 22)	40 (8 − 76)	19 (8 − 35)	83 (33 − 176)
Germany	2021	124248 (106487 − 140916)	282 (213 − 361)	7 (5 − 9)	22 (4 − 45)	26 (11 − 52)	87 (31 − 198)

Table S1: Global, super−regional, regional, and country/territory nutrition indicator attributable disability−adjusted life years, 2012 and 2021

location_id	Year	Low birthweight, both sexes, <28 days [DALYs per 100,000]	Exclusive breastfeeding, both sexes, <6 months [DALYs per 100,000]	Stunting, both sexes, <5 years [DALYs per 100,000]	Wasting, both sexes, <5 years [DALYs per 100,000]	Overweight, both sexes, 2−4 years [DALYs per 100,000]	Anaemia, females, 15−49 years [DALYs per 100,000]
Greece	2012	137127 (126435 − 147596)	455 (357 − 561)	24 (17 − 31)	17 (11 − 23)	23 (9 − 48)	128 (70 − 213)
Greece	2021	152347 (126023 − 180078)	312 (221 − 408)	14 (10 − 20)	11 (6 − 16)	28 (12 − 59)	134 (73 − 227)
Iceland	2012	69926 (59892 − 80265)	369 (291 − 460)	19 (14 − 26)	32 (16 − 48)	43 (18 − 86)	97 (56 − 154)
Iceland	2021	53057 (43959 − 63958)	242 (189 − 311)	13 (9 − 18)	23 (11 − 36)	45 (18 − 91)	94 (54 − 153)
Ireland	2012	128977 (118102 − 139821)	643 (512 − 777)	25 (19 − 32)	38 (23 − 51)	39 (16 − 76)	95 (50 − 159)
Ireland	2021	108242 (89050 − 128959)	393 (299 − 503)	11 (8 − 15)	18 (9 − 27)	42 (17 − 82)	93 (49 − 152)
Israel	2012	123393 (113623 − 134003)	769 (622 − 935)	26 (18 − 35)	74 (15 − 141)	20 (8 − 43)	123 (68 − 211)
Israel	2021	57347 (47032 − 69467)	432 (323 − 565)	9 (6 − 13)	34 (4 − 74)	21 (8 − 44)	121 (66 − 212)
Italy	2012	145461 (138882 − 151810)	374 (312 − 436)	15 (11 − 20)	31 (16 − 46)	14 (5 − 31)	106 (58 − 176)
Italy	2021	102553 (82164 − 122708)	382 (290 − 494)	9 (6 − 12)	26 (7 − 51)	18 (7 − 39)	105 (56 − 178)
Luxembourg	2012	71912 (62038 − 81877)	368 (292 − 472)	13 (8 − 16)	33 (10 − 59)	32 (12 − 63)	94 (51 − 155)
Luxembourg	2021	91375 (74900 − 111434)	355 (270 − 466)	10 (6 − 14)	33 (7 − 63)	34 (13 − 65)	91 (50 − 154)

Table S1: Global, super−regional, regional, and country/territory nutrition indicator attributable disability−adjusted life years, 2012 and 2021

location_id	Year	Low birthweight, both sexes, <28 days [DALYs per 100,000]	Exclusive breastfeeding, both sexes, <6 months [DALYs per 100,000]	Stunting, both sexes, <5 years [DALYs per 100,000]	Wasting, both sexes, <5 years [DALYs per 100,000]	Overweight, both sexes, 2−4 years [DALYs per 100,000]	Anaemia, females, 15−49 years [DALYs per 100,000]
Malta	2012	282606 (251021 − 316649)	506 (380 − 643)	34 (23 − 48)	49 (37 − 62)	21 (8 − 45)	120 (65 − 199)
Malta	2021	218223 (175128 − 271832)	311 (219 − 403)	16 (11 − 24)	26 (14 − 37)	24 (9 − 51)	117 (66 − 204)
Monaco	2012	86917 (64549 − 113587)	207 (136 − 307)	16 (10 − 23)	18 (11 − 27)	35 (14 − 70)	75 (40 − 119)
Monaco	2021	73427 (62207 − 83926)	181 (117 − 272)	19 (13 − 28)	24 (16 − 34)	37 (15 − 74)	72 (37 − 115)
Netherlands	2012	160956 (149642 − 173713)	598 (484 − 720)	22 (14 − 29)	47 (21 − 76)	20 (8 − 40)	105 (59 − 178)
Netherlands	2021	148498 (128749 − 166810)	432 (337 − 531)	13 (7 − 19)	36 (13 − 63)	24 (9 − 48)	101 (54 − 164)
Norway	2012	92794 (84630 − 101920)	105 (82 − 133)	8 (6 − 10)	18 (7 − 30)	40 (16 − 83)	87 (48 − 142)
Norway	2021	65297 (56572 − 75308)	95 (70 − 123)	4 (2 − 6)	11 (4 − 19)	40 (15 − 82)	85 (47 − 136)
Portugal	2012	152077 (140217 − 164823)	486 (394 − 581)	24 (18 − 32)	42 (26 − 57)	29 (11 − 55)	123 (68 − 212)
Portugal	2021	103480 (87332 − 119434)	364 (281 − 460)	14 (10 − 20)	29 (17 − 42)	37 (15 − 73)	123 (65 − 214)
San Marino	2012	85051 (69685 − 102794)	172 (119 − 237)	13 (7 − 17)	18 (8 − 28)	33 (14 − 66)	98 (53 − 165)
San Marino	2021	45068 (28587 − 64972)	89 (57 − 128)	6 (2 − 9)	9 (3 − 17)	35 (15 − 71)	99 (56 − 169)

Table S1: Global, super−regional, regional, and country/territory nutrition indicator attributable disability−adjusted life years, 2012 and 2021

location_id	Year	Low birthweight, both sexes, <28 days [DALYs per 100,000]	Exclusive breastfeeding, both sexes, <6 months [DALYs per 100,000]	Stunting, both sexes, <5 years [DALYs per 100,000]	Wasting, both sexes, <5 years [DALYs per 100,000]	Overweight, both sexes, 2−4 years [DALYs per 100,000]	Anaemia, females, 15−49 years [DALYs per 100,000]
Spain	2012	143469 (135533 − 152340)	457 (370 − 552)	12 (8 − 16)	33 (11 − 54)	23 (9 − 43)	101 (54 − 168)
Spain	2021	107724 (92745 − 120277)	339 (264 − 417)	7 (4 − 10)	23 (5 − 44)	25 (10 − 50)	103 (55 − 175)
Sweden	2012	86750 (79893 − 93911)	236 (190 − 289)	11 (8 − 15)	25 (11 − 41)	28 (10 − 58)	98 (52 − 166)
Sweden	2021	60788 (53425 − 68745)	172 (131 − 213)	6 (4 − 8)	16 (4 − 30)	38 (14 − 76)	95 (51 − 164)
Switzerland	2012	159509 (148090 − 173084)	606 (473 − 754)	15 (7 − 22)	35 (9 − 63)	30 (11 − 59)	76 (29 − 179)
Switzerland	2021	126613 (105821 − 149901)	441 (338 − 560)	9 (3 − 14)	24 (4 − 47)	30 (12 − 62)	89 (28 − 210)
UK	2012	193475 (184702 − 201840)	669 (562 − 777)	22 (18 − 27)	34 (22 − 47)	43 (18 − 86)	120 (70 − 189)
UK	2021	180557 (151143 − 206200)	395 (301 − 497)	8 (6 − 11)	15 (9 − 23)	45 (19 − 90)	118 (67 − 190)
Latin America and Caribbean	2012	785906 (701302 − 873443)	10215 (7790 − 12728)	1818 (1232 − 2420)	2526 (1587 − 3280)	57 (26 − 106)	636 (416 − 926)
Latin America and Caribbean	2021	573519 (460376 − 701450)	5384 (3863 − 7257)	855 (542 − 1257)	1391 (760 − 2061)	57 (25 − 105)	596 (379 − 865)
Andean Latin America	2012	723628 (610484 − 844564)	7138 (4702 − 10098)	2578 (1632 − 3571)	2311 (1797 − 2855)	84 (37 − 153)	465 (302 − 693)
Andean Latin America	2021	472633 (368781 − 579720)	3219 (2036 − 4644)	972 (568 − 1480)	1061 (738 − 1447)	85 (38 − 158)	415 (257 − 630)

Table S1: Global, super−regional, regional, and country/territory nutrition indicator attributable disability−adjusted life years, 2012 and 2021

location_id	Year	Low birthweight, both sexes, <28 days [DALYs per 100,000]	Exclusive breastfeeding, both sexes, <6 months [DALYs per 100,000]	Stunting, both sexes, <5 years [DALYs per 100,000]	Wasting, both sexes, <5 years [DALYs per 100,000]	Overweight, both sexes, 2−4 years [DALYs per 100,000]	Anaemia, females, 15−49 years [DALYs per 100,000]
Bolivia	2012	951381 (801162 − 1109345)	15279 (9525 − 22132)	4909 (3044 − 6908)	5631 (4134 − 7148)	93 (44 − 160)	810 (520 − 1232)
Bolivia	2021	681409 (539723 − 834314)	6869 (4339 − 10160)	1775 (1100 − 2626)	2489 (1707 − 3497)	80 (37 − 144)	676 (427 − 1017)
Ecuador	2012	674139 (542334 − 818381)	5879 (3827 − 8393)	1663 (1092 − 2288)	1714 (1337 − 2210)	44 (19 − 85)	254 (162 − 375)
Ecuador	2021	445326 (352039 − 560001)	2414 (1428 − 3595)	584 (324 − 930)	786 (536 − 1107)	48 (21 − 92)	240 (152 − 376)
Peru	2012	657913 (531610 − 798726)	4517 (2593 − 7131)	2160 (1345 − 3115)	1297 (974 − 1692)	104 (44 − 197)	455 (289 − 672)
Peru	2021	410235 (279176 − 539040)	2289 (1130 − 3846)	876 (443 − 1453)	683 (400 − 1022)	106 (44 − 202)	416 (249 − 656)
Caribbean	2012	1543403 (1361152 − 1746507)	36299 (25790 − 48312)	4727 (3231 − 6451)	9771 (3839 − 14139)	90 (40 − 160)	1054 (688 − 1536)
Caribbean	2021	1400511 (1128253 − 1686418)	28147 (18129 − 40028)	3186 (2001 − 4628)	7217 (2992 − 11570)	91 (40 − 161)	1073 (693 − 1536)
Antigua and Barbuda	2012	479411 (407092 − 563801)	3348 (2300 − 4491)	356 (211 − 527)	669 (522 − 859)	80 (31 − 166)	804 (489 − 1248)
Antigua and Barbuda	2021	384007 (326953 − 438719)	2382 (1667 − 3247)	239 (136 − 346)	715 (575 − 861)	76 (31 − 149)	766 (448 − 1227)
Barbados	2012	608476 (512861 − 715105)	2814 (1948 − 3830)	263 (153 − 386)	528 (410 − 661)	76 (30 − 149)	834 (519 − 1288)
Barbados	2021	567763 (406415 − 794344)	1928 (1204 − 2855)	159 (85 − 267)	382 (256 − 546)	81 (32 − 156)	818 (510 − 1269)

Table S1: Global, super−regional, regional, and country/territory nutrition indicator attributable disability−adjusted life years, 2012 and 2021

location_id	Year	Low birthweight, both sexes, <28 days [DALYs per 100,000]	Exclusive breastfeeding, both sexes, <6 months [DALYs per 100,000]	Stunting, both sexes, <5 years [DALYs per 100,000]	Wasting, both sexes, <5 years [DALYs per 100,000]	Overweight, both sexes, 2−4 years [DALYs per 100,000]	Anaemia, females, 15−49 years [DALYs per 100,000]
Belize	2012	814903 (695365 − 933503)	8133 (5671 − 10716)	1044 (617 − 1469)	1518 (1124 − 1854)	57 (25 − 108)	1124 (689 − 1696)
Belize	2021	694479 (569517 − 834907)	5067 (3587 − 6652)	522 (292 − 784)	1269 (933 − 1695)	54 (23 − 100)	1087 (670 − 1631)
Bermuda	2012	155799 (115536 − 195102)	644 (391 − 925)	82 (55 − 118)	101 (73 − 132)	125 (50 − 250)	440 (267 − 695)
Bermuda	2021	108371 (73602 − 140975)	343 (194 − 503)	48 (32 − 70)	74 (51 − 100)	128 (53 − 250)	419 (255 − 636)
Cuba	2012	164981 (148507 − 181283)	2268 (1759 − 2793)	224 (138 − 314)	233 (170 − 293)	96 (40 − 183)	764 (475 − 1183)
Cuba	2021	121811 (104010 − 139138)	1690 (1241 − 2251)	119 (63 − 183)	176 (119 − 236)	105 (43 − 200)	718 (438 − 1100)
Dominica	2012	1147577 (942587 − 1410299)	7507 (5038 − 10558)	733 (406 − 1149)	1363 (927 − 1757)	97 (43 − 174)	996 (597 − 1439)
Dominica	2021	1190464 (842547 − 1652915)	6536 (3876 − 10369)	595 (312 − 1023)	1324 (835 − 2021)	93 (43 − 165)	983 (602 − 1447)
Dominican Republic	2012	1793882 (1474124 − 2159812)	16799 (11778 − 22744)	1248 (751 − 1852)	3270 (1525 − 4994)	37 (17 − 68)	972 (603 − 1540)
Dominican Republic	2021	1357642 (1095693 − 1682870)	8615 (4415 − 13881)	483 (227 − 793)	1705 (822 − 2814)	33 (15 − 61)	927 (585 − 1421)
Grenada	2012	697927 (550816 − 863534)	4775 (3098 − 6716)	603 (318 − 931)	813 (594 − 1066)	73 (31 − 144)	1030 (619 − 1514)
Grenada	2021	606655 (488587 − 744866)	2536 (1654 − 3592)	267 (134 − 416)	728 (547 − 947)	89 (36 − 181)	965 (607 − 1427)

Table S1: Global, super−regional, regional, and country/territory nutrition indicator attributable disability−adjusted life years, 2012 and 2021

location_id	Year	Low birthweight, both sexes, <28 days [DALYs per 100,000]	Exclusive breastfeeding, both sexes, <6 months [DALYs per 100,000]	Stunting, both sexes, <5 years [DALYs per 100,000]	Wasting, both sexes, <5 years [DALYs per 100,000]	Overweight, both sexes, 2−4 years [DALYs per 100,000]	Anaemia, females, 15−49 years [DALYs per 100,000]
Guyana	2012	1550041 (1322384 − 1802730)	12875 (9660 − 16684)	1849 (1361 − 2454)	4162 (2590 − 5620)	40 (18 − 75)	1096 (709 − 1679)
Guyana	2021	1223300 (892185 − 1627736)	7370 (4719 − 10751)	782 (501 − 1197)	2446 (1551 − 3529)	39 (17 − 76)	1023 (644 − 1582)
Haiti	2012	2238817 (1876953 − 2658549)	78085 (53098 − 109268)	11494 (7820 − 15797)	23357 (8803 − 34311)	118 (49 − 219)	1669 (1086 − 2437)
Haiti	2021	2009039 (1601715 − 2456938)	56723 (35295 − 82943)	7124 (4478 − 10296)	15690 (6253 − 25632)	128 (54 − 230)	1650 (1041 − 2417)
Jamaica	2012	1190082 (986547 − 1415880)	2739 (1896 − 3616)	194 (117 − 284)	538 (349 − 763)	73 (30 − 142)	890 (534 − 1331)
Jamaica	2021	956121 (706923 − 1282539)	2163 (1350 − 3235)	111 (60 − 179)	354 (201 − 544)	69 (29 − 132)	878 (538 − 1323)
Puerto Rico	2012	477070 (435916 − 519165)	2382 (1739 − 3146)	126 (75 − 183)	200 (156 − 238)	210 (93 − 401)	526 (307 − 813)
Puerto Rico	2021	277569 (231901 − 334116)	1515 (1127 − 1984)	54 (31 − 85)	194 (131 − 264)	206 (91 − 402)	513 (307 − 812)
Saint Kitts and Nevis	2012	849761 (727204 − 982546)	6772 (5029 − 8634)	560 (323 − 831)	1564 (1058 − 2153)	84 (34 − 167)	867 (511 − 1364)
Saint Kitts and Nevis	2021	772335 (610621 − 988759)	5033 (3448 − 6817)	353 (183 − 576)	1629 (1099 − 2306)	84 (34 − 162)	838 (497 − 1291)
Saint Lucia	2012	1182447 (947486 − 1469072)	3963 (2727 − 5402)	231 (128 − 353)	608 (451 − 799)	32 (13 − 61)	983 (598 − 1476)
Saint Lucia	2021	1001567 (718589 − 1377029)	2889 (1866 − 4239)	148 (68 − 259)	551 (358 − 815)	32 (13 − 60)	962 (601 − 1442)

Table S1: Global, super−regional, regional, and country/territory nutrition indicator attributable disability−adjusted life years, 2012 and 2021

location_id	Year	Low birthweight, both sexes, <28 days [DALYs per 100,000]	Exclusive breastfeeding, both sexes, <6 months [DALYs per 100,000]	Stunting, both sexes, <5 years [DALYs per 100,000]	Wasting, both sexes, <5 years [DALYs per 100,000]	Overweight, both sexes, 2−4 years [DALYs per 100,000]	Anaemia, females, 15−49 years [DALYs per 100,000]
Saint Vincent and the Grenadines	2012	909056 (769116 − 1067094)	5367 (3803 − 7023)	438 (258 − 653)	1179 (836 − 1535)	41 (16 − 83)	1035 (615 − 1629)
Saint Vincent and the Grenadines	2021	625309 (465881 − 830699)	3478 (2341 − 4918)	222 (120 − 361)	1144 (797 − 1632)	41 (17 − 81)	1006 (619 − 1549)
Suriname	2012	1303727 (1082408 − 1556226)	15843 (10366 − 22250)	1127 (644 − 1738)	2644 (1255 − 3995)	21 (8 − 44)	1085 (650 − 1617)
Suriname	2021	1166801 (885948 − 1544028)	11518 (6749 − 18170)	670 (362 − 1083)	1918 (812 − 3188)	22 (8 − 43)	1057 (647 − 1605)
The Bahamas	2012	515228 (433070 − 610062)	4140 (2807 − 5695)	359 (188 − 541)	523 (393 − 657)	96 (40 − 192)	839 (525 − 1296)
The Bahamas	2021	384572 (296565 − 491876)	2498 (1609 − 3608)	200 (89 − 332)	441 (309 − 613)	89 (38 − 171)	831 (506 − 1262)
Trinidad and Tobago	2012	824871 (681071 − 1008037)	4723 (3231 − 6439)	310 (173 − 473)	810 (588 − 1035)	35 (15 − 70)	873 (516 − 1357)
Trinidad and Tobago	2021	664477 (504909 − 874708)	2957 (1935 − 4184)	144 (72 − 240)	494 (327 − 691)	27 (11 − 56)	851 (509 − 1334)
Virgin Islands	2012	327702 (270140 − 396015)	1250 (789 − 1857)	113 (65 − 184)	343 (222 − 495)	162 (69 − 313)	627 (390 − 974)
Virgin Islands	2021	214290 (163654 − 264821)	773 (422 − 1274)	58 (31 − 92)	226 (128 − 344)	156 (65 − 290)	642 (392 − 1017)
Central Latin America	2012	684651 (595397 − 778573)	8738 (6531 − 11172)	1741 (1123 − 2344)	2101 (1453 − 2732)	33 (14 − 65)	342 (226 − 492)
Central Latin America	2021	520583 (415584 − 654796)	4295 (2909 − 6059)	869 (508 − 1339)	1171 (703 − 1749)	33 (14 − 65)	312 (202 − 461)

Table S1: Global, super−regional, regional, and country/territory nutrition indicator attributable disability−adjusted life years, 2012 and 2021

location_id	Year	Low birthweight, both sexes, <28 days [DALYs per 100,000]	Exclusive breastfeeding, both sexes, <6 months [DALYs per 100,000]	Stunting, both sexes, <5 years [DALYs per 100,000]	Wasting, both sexes, <5 years [DALYs per 100,000]	Overweight, both sexes, 2−4 years [DALYs per 100,000]	Anaemia, females, 15−49 years [DALYs per 100,000]
Colombia	2012	685115 (533211 − 865316)	3269 (1873 − 5037)	760 (477 − 1135)	1576 (1108 − 2189)	26 (11 − 51)	270 (161 − 426)
Colombia	2021	365722 (255570 − 529077)	1943 (1135 − 2931)	260 (131 − 465)	1036 (673 − 1547)	30 (12 − 63)	249 (140 − 385)
Costa Rica	2012	378805 (351686 − 406321)	1644 (1188 − 2125)	130 (83 − 185)	167 (116 − 238)	73 (29 − 147)	229 (124 − 383)
Costa Rica	2021	329226 (268605 − 401916)	1214 (819 − 1669)	82 (51 − 126)	118 (71 − 188)	79 (33 − 151)	220 (117 − 373)
El Salvador	2012	516876 (436071 − 616431)	6849 (4560 − 9636)	1264 (752 − 1853)	1529 (973 − 2136)	45 (17 − 92)	196 (120 − 297)
El Salvador	2021	298551 (214023 − 412444)	3319 (1917 − 5173)	442 (248 − 724)	685 (376 − 1112)	49 (19 − 97)	182 (109 − 276)
Guatemala	2012	777189 (656591 − 916494)	24468 (16413 − 32735)	10261 (6679 − 13573)	6582 (4162 − 9392)	28 (12 − 56)	1372 (903 − 1992)
Guatemala	2021	631631 (479658 − 826038)	12898 (8243 − 18222)	5483 (3311 − 8125)	3602 (2120 − 5727)	29 (13 − 59)	1021 (656 − 1512)
Honduras	2012	835891 (684801 − 1023453)	13963 (8575 − 20534)	1845 (1092 − 2736)	1876 (622 − 3720)	41 (18 − 77)	380 (244 − 577)
Honduras	2021	558619 (419054 − 713896)	7265 (4180 − 10969)	825 (446 − 1332)	982 (306 − 2022)	35 (15 − 64)	357 (224 − 546)
Mexico	2012	658918 (567737 − 753129)	8037 (5977 − 10142)	1125 (691 − 1560)	1789 (1315 − 2219)	28 (12 − 56)	267 (173 − 387)
Mexico	2021	494197 (399366 − 599283)	3147 (2024 − 4624)	487 (260 − 798)	783 (490 − 1150)	25 (10 − 52)	248 (160 − 363)

Table S1: Global, super−regional, regional, and country/territory nutrition indicator attributable disability−adjusted life years, 2012 and 2021

location_id	Year	Low birthweight, both sexes, <28 days [DALYs per 100,000]	Exclusive breastfeeding, both sexes, <6 months [DALYs per 100,000]	Stunting, both sexes, <5 years [DALYs per 100,000]	Wasting, both sexes, <5 years [DALYs per 100,000]	Overweight, both sexes, 2−4 years [DALYs per 100,000]	Anaemia, females, 15−49 years [DALYs per 100,000]
Nicaragua	2012	800000 (648309 − 966796)	12056 (8453 − 16202)	1521 (927 − 2174)	2389 (1551 − 3406)	50 (22 − 96)	400 (246 − 619)
Nicaragua	2021	557499 (405215 − 732148)	5727 (3572 − 8386)	514 (266 − 856)	1103 (640 − 1760)	47 (20 − 94)	384 (233 − 586)
Panama	2012	502827 (421573 − 593904)	8886 (6602 − 11511)	1525 (903 − 2208)	1962 (1383 − 2676)	76 (32 − 147)	325 (179 − 547)
Panama	2021	410977 (315604 − 531775)	5136 (3504 − 6870)	707 (408 − 1094)	1226 (771 − 1829)	77 (33 − 149)	291 (153 − 504)
Venezuela	2012	754090 (709556 − 802164)	8083 (6264 − 10161)	802 (515 − 1100)	1790 (1122 − 2360)	50 (21 − 96)	310 (173 − 506)
Venezuela	2021	858247 (650060 − 1111648)	5619 (3633 − 7869)	643 (364 − 1034)	1788 (912 − 2827)	54 (23 − 106)	324 (178 − 560)
Tropical Latin America	2012	768557 (636978 − 914370)	7265 (5437 − 9384)	992 (600 − 1421)	1513 (1063 − 1960)	73 (30 − 135)	918 (596 − 1343)
Tropical Latin America	2021	479824 (385422 − 600034)	2205 (1558 − 2888)	274 (167 − 407)	456 (310 − 607)	67 (27 − 128)	872 (539 − 1279)
Brazil	2012	771857 (639442 − 922019)	7297 (5402 − 9454)	991 (595 − 1426)	1498 (1043 − 1955)	73 (30 − 136)	922 (599 − 1346)
Brazil	2021	478118 (381772 − 597399)	2132 (1510 − 2807)	270 (164 − 400)	436 (296 − 583)	66 (27 − 127)	877 (539 − 1290)
Paraguay	2012	685840 (551562 − 835472)	6454 (4235 − 9213)	1018 (674 − 1491)	1882 (1332 − 2500)	72 (28 − 143)	787 (482 − 1207)
Paraguay	2021	523645 (374532 − 711575)	4086 (2294 − 6481)	365 (211 − 604)	960 (579 − 1431)	81 (32 − 159)	719 (440 − 1131)

Table S1: Global, super−regional, regional, and country/territory nutrition indicator attributable disability−adjusted life years, 2012 and 2021

location_id	Year	Low birthweight, both sexes, <28 days [DALYs per 100,000]	Exclusive breastfeeding, both sexes, <6 months [DALYs per 100,000]	Stunting, both sexes, <5 years [DALYs per 100,000]	Wasting, both sexes, <5 years [DALYs per 100,000]	Overweight, both sexes, 2−4 years [DALYs per 100,000]	Anaemia, females, 15−49 years [DALYs per 100,000]
North Africa and Middle East	2012	1095047 (972697 − 1201961)	12030 (8471 − 16663)	3430 (2443 − 4435)	4360 (2658 − 5592)	32 (14 − 58)	875 (571 − 1264)
North Africa and Middle East	2021	696345 (600781 − 811063)	6275 (4245 − 8970)	1397 (1019 − 1853)	1876 (1119 − 2516)	32 (14 − 61)	849 (556 − 1233)
North Africa and Middle East	2012	1095047 (972697 − 1201961)	12030 (8471 − 16663)	3430 (2443 − 4435)	4360 (2658 − 5592)	32 (14 − 58)	875 (571 − 1264)
North Africa and Middle East	2021	696345 (600781 − 811063)	6275 (4245 − 8970)	1397 (1019 − 1853)	1876 (1119 − 2516)	32 (14 − 61)	849 (556 − 1233)
Afghanistan	2012	1869017 (1547557 − 2203785)	38012 (23226 − 58122)	15095 (9963 − 21065)	15858 (8332 − 22944)	12 (5 − 25)	712 (466 − 1039)
Afghanistan	2021	1370930 (1066031 − 1650510)	17183 (10246 − 27103)	6368 (4092 − 8820)	6868 (3670 − 10172)	11 (4 − 22)	651 (411 − 981)
Algeria	2012	1036110 (878942 − 1233946)	4357 (2789 − 6510)	864 (550 − 1223)	1169 (864 − 1471)	20 (8 − 42)	818 (501 − 1252)
Algeria	2021	682539 (489930 − 900322)	2087 (1309 − 3102)	260 (163 − 369)	426 (278 − 587)	25 (10 − 53)	799 (475 − 1236)
Bahrain	2012	206071 (181478 − 232334)	2339 (1725 − 3105)	298 (183 − 420)	402 (291 − 497)	35 (14 − 71)	778 (466 − 1188)
Bahrain	2021	143560 (116437 − 170737)	981 (671 − 1399)	95 (56 − 145)	167 (102 − 246)	41 (18 − 79)	747 (438 − 1188)
Egypt	2012	763270 (623689 − 926119)	16151 (10194 − 23894)	3824 (2528 − 5331)	4347 (2724 − 5630)	39 (18 − 76)	612 (385 − 913)
Egypt	2021	308224 (225329 − 400672)	8832 (5375 − 12903)	1348 (841 − 1989)	1667 (870 − 2393)	39 (18 − 77)	515 (325 − 781)

Table S1: Global, super−regional, regional, and country/territory nutrition indicator attributable disability−adjusted life years, 2012 and 2021

location_id	Year	Low birthweight, both sexes, <28 days [DALYs per 100,000]	Exclusive breastfeeding, both sexes, <6 months [DALYs per 100,000]	Stunting, both sexes, <5 years [DALYs per 100,000]	Wasting, both sexes, <5 years [DALYs per 100,000]	Overweight, both sexes, 2−4 years [DALYs per 100,000]	Anaemia, females, 15−49 years [DALYs per 100,000]
Iran	2012	947077 (828602 − 1074396)	3522 (2349 − 4780)	645 (385 − 938)	936 (720 − 1121)	24 (9 − 47)	779 (475 − 1197)
Iran	2021	212793 (169739 − 260750)	473 (302 − 693)	58 (37 − 85)	118 (80 − 157)	28 (10 − 56)	759 (482 − 1155)
Iraq	2012	1254669 (1014123 − 1497526)	8604 (5804 − 12275)	1845 (1334 − 2536)	2127 (1405 − 2787)	30 (13 − 58)	783 (461 − 1193)
Iraq	2021	851458 (659243 − 1085748)	4914 (2971 − 7482)	772 (514 − 1109)	944 (551 − 1376)	32 (13 − 62)	716 (428 − 1109)
Jordan	2012	693499 (589935 − 809306)	4579 (3109 − 6254)	560 (349 − 785)	524 (390 − 660)	27 (11 − 55)	988 (639 − 1419)
Jordan	2021	465891 (375228 − 562205)	2567 (1738 − 3657)	252 (156 − 380)	257 (174 − 357)	32 (13 − 61)	1051 (652 − 1605)
Kuwait	2012	322447 (294691 − 352457)	2841 (2033 − 3739)	328 (206 − 457)	253 (188 − 322)	57 (24 − 114)	542 (320 − 835)
Kuwait	2021	257818 (210052 − 309821)	2039 (1392 − 2786)	188 (115 − 277)	149 (104 − 205)	63 (28 − 122)	580 (347 − 913)
Lebanon	2012	457999 (364805 − 573150)	5660 (3620 − 8339)	811 (526 − 1126)	730 (448 − 1026)	36 (14 − 72)	778 (485 − 1178)
Lebanon	2021	291708 (204159 − 413241)	3541 (2151 − 5376)	408 (232 − 656)	417 (214 − 664)	45 (18 − 93)	774 (486 − 1150)
Libya	2012	538469 (420560 − 661894)	2038 (1160 − 3223)	859 (604 − 1166)	739 (500 − 981)	38 (16 − 70)	726 (444 − 1145)
Libya	2021	618866 (477356 − 782849)	1570 (863 − 2495)	539 (363 − 771)	570 (361 − 824)	44 (19 − 80)	784 (457 − 1230)

Table S1: Global, super−regional, regional, and country/territory nutrition indicator attributable disability−adjusted life years, 2012 and 2021

location_id	Year	Low birthweight, both sexes, <28 days [DALYs per 100,000]	Exclusive breastfeeding, both sexes, <6 months [DALYs per 100,000]	Stunting, both sexes, <5 years [DALYs per 100,000]	Wasting, both sexes, <5 years [DALYs per 100,000]	Overweight, both sexes, 2−4 years [DALYs per 100,000]	Anaemia, females, 15−49 years [DALYs per 100,000]
Morocco	2012	1166479 (907589 − 1475315)	14642 (8708 − 21866)	1995 (1245 − 2906)	2867 (1242 − 4724)	24 (10 − 43)	877 (549 − 1385)
Morocco	2021	682827 (541464 − 831430)	6506 (3664 − 10624)	507 (294 − 810)	894 (269 − 1777)	25 (10 − 48)	815 (501 − 1301)
Oman	2012	409242 (353046 − 454526)	2252 (1420 − 3321)	470 (301 − 656)	814 (615 − 986)	36 (15 − 74)	560 (333 − 906)
Oman	2021	295188 (245017 − 345252)	1543 (910 − 2406)	250 (146 − 371)	523 (360 − 679)	48 (19 − 99)	564 (341 − 914)
Palestine	2012	691803 (577045 − 822576)	3659 (2394 − 5157)	679 (487 − 899)	627 (468 − 793)	26 (11 − 54)	512 (300 − 789)
Palestine	2021	394043 (308008 − 511605)	1884 (1103 − 2904)	239 (154 − 361)	212 (139 − 306)	32 (13 − 63)	478 (282 − 719)
Qatar	2012	191324 (153561 − 231323)	1544 (1060 − 2138)	197 (123 − 288)	199 (143 − 268)	44 (18 − 86)	563 (337 − 902)
Qatar	2021	118982 (91468 − 148944)	718 (467 − 1061)	60 (36 − 92)	77 (45 − 119)	54 (23 − 103)	565 (326 − 927)
Saudi Arabia	2012	272737 (233369 − 311342)	1935 (1329 − 2680)	244 (164 − 346)	632 (430 − 815)	34 (15 − 66)	714 (409 − 1102)
Saudi Arabia	2021	122926 (89990 − 157630)	780 (452 − 1334)	69 (39 − 104)	259 (157 − 389)	41 (17 − 78)	679 (404 − 1035)
Sudan	2012	2009416 (1655302 − 2465458)	15507 (7102 − 34159)	6503 (4555 − 9296)	10603 (6242 − 17292)	24 (9 − 45)	1220 (759 − 1838)
Sudan	2021	1353713 (1101784 − 1698572)	5533 (2688 − 13221)	1946 (1205 − 3088)	3336 (1990 − 5884)	18 (8 − 34)	1118 (687 − 1697)

Table S1: Global, super−regional, regional, and country/territory nutrition indicator attributable disability−adjusted life years, 2012 and 2021

location_id	Year	Low birthweight, both sexes, <28 days [DALYs per 100,000]	Exclusive breastfeeding, both sexes, <6 months [DALYs per 100,000]	Stunting, both sexes, <5 years [DALYs per 100,000]	Wasting, both sexes, <5 years [DALYs per 100,000]	Overweight, both sexes, 2−4 years [DALYs per 100,000]	Anaemia, females, 15−49 years [DALYs per 100,000]
Syria	2012	515139 (438177 − 594144)	1892 (1159 − 3006)	1874 (1356 − 2509)	1907 (1452 − 2448)	65 (32 − 116)	868 (530 − 1339)
Syria	2021	310087 (241806 − 382451)	767 (421 − 1250)	561 (348 − 817)	919 (695 − 1213)	74 (37 − 135)	850 (509 − 1302)
Tunisia	2012	726577 (597420 − 872818)	4940 (2647 − 8166)	607 (360 − 944)	677 (439 − 960)	25 (10 − 49)	774 (501 − 1154)
Tunisia	2021	426415 (335423 − 525841)	2456 (1251 − 4034)	215 (119 − 356)	261 (146 − 373)	29 (12 − 61)	759 (488 − 1177)
Türkiye	2012	724597 (591162 − 878636)	3379 (2154 − 4927)	527 (351 − 744)	643 (440 − 872)	42 (17 − 81)	703 (422 − 1116)
Türkiye	2021	379049 (307949 − 467091)	1811 (1090 − 2872)	135 (84 − 208)	265 (150 − 426)	49 (20 − 95)	666 (390 − 1069)
United Arab Emirates	2012	284179 (241053 − 342998)	2293 (1516 − 3295)	352 (237 − 493)	647 (415 − 854)	82 (35 − 156)	822 (465 − 1450)
United Arab Emirates	2021	157425 (126196 − 199890)	1021 (656 − 1501)	119 (79 − 173)	265 (163 − 369)	80 (33 − 153)	791 (450 − 1278)
Yemen	2012	2018926 (1668215 − 2402479)	20943 (12315 − 34328)	6376 (4357 − 8724)	9637 (6265 − 13032)	30 (13 − 56)	3079 (2096 − 4363)
Yemen	2021	1516447 (1230492 − 1856114)	10680 (5742 − 17711)	2685 (1754 − 3866)	4727 (3213 − 6467)	29 (13 − 52)	3058 (2055 − 4242)
South Asia	2012	2775016 (2443007 − 3120143)	33436 (23523 − 44785)	9666 (7391 − 12201)	14192 (8947 − 16937)	7 (3 − 13)	1826 (1224 − 2615)
South Asia	2021	2186444 (1841818 − 2596005)	13662 (8964 − 18969)	3482 (2385 − 4848)	5299 (3584 − 6752)	5 (2 − 10)	1654 (1092 − 2368)

Table S1: Global, super−regional, regional, and country/territory nutrition indicator attributable disability−adjusted life years, 2012 and 2021

location_id	Year	Low birthweight, both sexes, <28 days [DALYs per 100,000]	Exclusive breastfeeding, both sexes, <6 months [DALYs per 100,000]	Stunting, both sexes, <5 years [DALYs per 100,000]	Wasting, both sexes, <5 years [DALYs per 100,000]	Overweight, both sexes, 2−4 years [DALYs per 100,000]	Anaemia, females, 15−49 years [DALYs per 100,000]
South Asia	2012	2775016 (2443007 − 3120143)	33436 (23523 − 44785)	9666 (7391 − 12201)	14192 (8947 − 16937)	7 (3 − 13)	1826 (1224 − 2615)
South Asia	2021	2186444 (1841818 − 2596005)	13662 (8964 − 18969)	3482 (2385 − 4848)	5299 (3584 − 6752)	5 (2 − 10)	1654 (1092 − 2368)
Bangladesh	2012	2840642 (2468514 − 3263153)	14674 (8592 − 22335)	5241 (3608 − 7199)	7369 (5838 − 9135)	2 (1 − 5)	1022 (657 − 1513)
Bangladesh	2021	1760481 (1321689 − 2262452)	5984 (3265 − 9704)	1757 (1097 − 2560)	2549 (1784 − 3379)	3 (1 − 5)	949 (578 − 1468)
Bhutan	2012	1805290 (1505507 − 2132065)	19633 (10796 − 32103)	4097 (2604 − 5825)	5652 (2552 − 9324)	8 (3 − 14)	1459 (896 − 2219)
Bhutan	2021	1313006 (997030 − 1654157)	9803 (5112 − 16539)	1654 (1040 − 2381)	2389 (1019 − 4077)	7 (3 − 13)	1332 (791 − 2071)
India	2012	2611186 (2229448 − 3038694)	33037 (22490 − 45030)	9994 (7393 − 12757)	14619 (9082 − 17891)	8 (3 − 16)	1935 (1300 − 2746)
India	2021	1953988 (1577518 − 2404596)	13210 (8538 − 19117)	3386 (2223 − 4823)	5250 (3572 − 6897)	5 (2 − 11)	1730 (1151 − 2460)
Nepal	2012	2475916 (2047236 − 2942860)	18783 (11934 − 27413)	5970 (4003 − 8084)	8802 (6278 − 11117)	8 (3 − 16)	1024 (648 − 1532)
Nepal	2021	1697266 (1293347 − 2219059)	7254 (4000 − 11725)	1886 (1164 − 2881)	3139 (2108 − 4362)	7 (3 − 14)	1023 (638 − 1542)
Pakistan	2012	3482004 (2974555 − 4081557)	48661 (33261 − 67453)	11503 (7975 − 15236)	17356 (9695 − 22129)	5 (2 − 10)	1888 (1197 − 2803)
Pakistan	2021	3253796 (2713521 − 3923334)	19471 (10948 − 29579)	4847 (3253 − 6996)	7041 (4361 − 9816)	4 (2 − 8)	1817 (1159 − 2671)

Table S1: Global, super−regional, regional, and country/territory nutrition indicator attributable disability−adjusted life years, 2012 and 2021

location_id	Year	Low birthweight, both sexes, <28 days [DALYs per 100,000]	Exclusive breastfeeding, both sexes, <6 months [DALYs per 100,000]	Stunting, both sexes, <5 years [DALYs per 100,000]	Wasting, both sexes, <5 years [DALYs per 100,000]	Overweight, both sexes, 2−4 years [DALYs per 100,000]	Anaemia, females, 15−49 years [DALYs per 100,000]
Southeast Asia, east Asia, and Oceania	2012	666234 (594505 − 741598)	10008 (7348 − 12744)	2241 (1656 − 2841)	2759 (1773 − 3353)	16 (7 − 30)	560 (374 − 820)
Southeast Asia, east Asia, and Oceania	2021	489470 (421056 − 561449)	5752 (4191 − 7507)	966 (671 − 1335)	1315 (791 − 1736)	17 (7 − 31)	528 (343 − 778)
East Asia	2012	376677 (332820 − 423237)	3030 (2176 − 4082)	549 (365 − 753)	474 (371 − 588)	14 (5 − 29)	456 (304 − 670)
East Asia	2021	185931 (158660 − 218315)	1929 (1315 − 2619)	203 (111 − 308)	198 (146 − 255)	16 (6 − 32)	402 (262 − 591)
China	2012	374067 (325680 − 422858)	3041 (2189 − 4111)	532 (351 − 731)	462 (362 − 573)	14 (5 − 28)	455 (304 − 668)
China	2021	182920 (155702 − 216052)	1955 (1332 − 2647)	199 (106 − 306)	196 (145 − 253)	15 (5 − 31)	399 (260 − 584)
North Korea	2012	602034 (459383 − 763588)	3816 (2040 − 6251)	1505 (901 − 2199)	1146 (739 − 1671)	30 (12 − 60)	623 (374 − 975)
North Korea	2021	319130 (225131 − 429385)	1629 (794 − 2750)	517 (290 − 784)	396 (228 − 579)	34 (14 − 68)	592 (356 − 903)
Taiwan (province of China)	2012	164479 (148339 − 181106)	884 (620 − 1194)	88 (64 − 123)	156 (124 − 186)	41 (16 − 79)	344 (204 − 538)
Taiwan (province of China)	2021	148953 (125816 − 171379)	583 (396 − 791)	40 (29 − 55)	95 (74 − 117)	59 (22 − 118)	343 (204 − 560)
Oceania	2012	1210537 (996548 − 1443061)	26037 (16768 − 37233)	17493 (12026 − 24056)	19433 (12498 − 26529)	23 (10 − 41)	1152 (723 − 1786)
Oceania	2021	1202856 (979905 − 1433285)	18034 (11292 − 26478)	10023 (6476 − 14331)	11293 (6464 − 16157)	21 (9 − 39)	1165 (720 − 1840)

Table S1: Global, super−regional, regional, and country/territory nutrition indicator attributable disability−adjusted life years, 2012 and 2021

location_id	Year	Low birthweight, both sexes, <28 days [DALYs per 100,000]	Exclusive breastfeeding, both sexes, <6 months [DALYs per 100,000]	Stunting, both sexes, <5 years [DALYs per 100,000]	Wasting, both sexes, <5 years [DALYs per 100,000]	Overweight, both sexes, 2−4 years [DALYs per 100,000]	Anaemia, females, 15−49 years [DALYs per 100,000]
American Samoa	2012	309511 (255862 − 363477)	2719 (1822 − 3889)	759 (428 − 1161)	1426 (1110 − 1766)	36 (15 − 70)	884 (532 − 1327)
American Samoa	2021	321558 (242604 − 416755)	2523 (1496 − 3748)	573 (315 − 925)	1330 (921 − 1875)	29 (13 − 55)	889 (540 − 1341)
Cook Islands	2012	126388 (88269 − 164056)	1252 (710 − 1931)	295 (169 − 460)	227 (140 − 332)	52 (21 − 104)	657 (389 − 1011)
Cook Islands	2021	122075 (97628 − 142144)	1385 (797 − 2133)	309 (194 − 441)	281 (181 − 387)	47 (21 − 88)	616 (371 − 1005)
Federated States of Micronesia	2012	626268 (495788 − 785225)	6064 (3907 − 9026)	1839 (939 − 2901)	2540 (1845 − 3288)	24 (11 − 46)	1095 (671 − 1685)
Federated States of Micronesia	2021	536007 (419043 − 659739)	4010 (2350 − 6143)	992 (497 − 1564)	1615 (1082 − 2185)	19 (9 − 36)	1069 (675 − 1622)
Fiji	2012	618612 (479497 − 760149)	6213 (4198 − 8758)	804 (517 − 1217)	4063 (2872 − 5336)	27 (12 − 50)	1004 (595 − 1530)
Fiji	2021	630905 (475442 − 846852)	3238 (1960 − 5032)	305 (180 − 486)	2048 (1291 − 3073)	27 (12 − 49)	980 (582 − 1520)
Guam	2012	519941 (464365 − 579500)	3146 (2009 − 4398)	586 (356 − 863)	926 (752 − 1115)	46 (20 − 97)	637 (387 − 1004)
Guam	2021	477990 (374753 − 601410)	1984 (1253 − 2886)	297 (158 − 478)	638 (465 − 865)	37 (16 − 72)	650 (408 − 997)
Kiribati	2012	1177124 (973942 − 1401419)	11969 (7172 − 18527)	3427 (2001 − 5270)	12256 (7513 − 17127)	41 (19 − 74)	1301 (832 − 2020)
Kiribati	2021	1152411 (933856 − 1424302)	7265 (4119 − 11714)	1891 (1070 − 2997)	7663 (4673 − 11157)	33 (15 − 57)	1276 (816 − 1977)

Table S1: Global, super−regional, regional, and country/territory nutrition indicator attributable disability−adjusted life years, 2012 and 2021

location_id	Year	Low birthweight, both sexes, <28 days [DALYs per 100,000]	Exclusive breastfeeding, both sexes, <6 months [DALYs per 100,000]	Stunting, both sexes, <5 years [DALYs per 100,000]	Wasting, both sexes, <5 years [DALYs per 100,000]	Overweight, both sexes, 2−4 years [DALYs per 100,000]	Anaemia, females, 15−49 years [DALYs per 100,000]
Marshall Islands	2012	842701 (690452 − 998120)	9639 (5812 − 14393)	3007 (1564 − 4616)	1807 (1287 − 2487)	24 (11 − 41)	1138 (700 − 1737)
Marshall Islands	2021	723107 (563929 − 923080)	5991 (3534 − 9100)	1439 (697 − 2415)	1071 (672 − 1602)	19 (9 − 34)	1113 (664 − 1717)
Nauru	2012	884191 (691900 − 1083343)	10961 (6464 − 16326)	6048 (3331 − 9077)	4055 (3043 − 5370)	53 (26 − 90)	1142 (714 − 1714)
Nauru	2021	740185 (535375 − 1024344)	5631 (3311 − 8507)	2515 (1384 − 4002)	1818 (1236 − 2582)	35 (17 − 57)	1015 (630 − 1604)
Niue	2012	648995 (515294 − 788146)	4848 (2964 − 7491)	1690 (942 − 2690)	2279 (1583 − 3098)	42 (19 − 77)	865 (523 − 1354)
Niue	2021	1327616 (1236356 − 1426153)	12009 (7446 − 18162)	3442 (2576 − 4708)	4964 (3981 − 6020)	75 (36 − 147)	848 (516 − 1310)
Northern Mariana Islands	2012	301961 (246405 − 367392)	1545 (950 − 2236)	310 (195 − 459)	448 (333 − 591)	46 (19 − 92)	769 (458 − 1227)
Northern Mariana Islands	2021	254037 (203882 − 307999)	968 (587 − 1434)	149 (89 − 223)	340 (246 − 455)	36 (15 − 72)	710 (426 − 1137)
Palau	2012	697415 (567813 − 831894)	5442 (3295 − 8175)	1197 (605 − 1913)	1699 (1179 − 2341)	42 (18 − 79)	878 (528 − 1356)
Palau	2021	628964 (516437 − 772281)	3992 (2584 − 5821)	667 (387 − 1044)	1197 (817 − 1693)	35 (16 − 64)	876 (532 − 1379)
Papua New Guinea	2012	1353337 (1104278 − 1621647)	30912 (19759 − 44999)	21637 (14846 − 29743)	23672 (15092 − 32518)	22 (9 − 43)	1186 (728 − 1869)
Papua New Guinea	2021	1322783 (1075201 − 1576653)	20732 (12867 − 30761)	11863 (7692 − 17002)	13174 (7476 − 18905)	21 (8 − 41)	1196 (723 − 1904)

Table S1: Global, super−regional, regional, and country/territory nutrition indicator attributable disability−adjusted life years, 2012 and 2021

location_id	Year	Low birthweight, both sexes, <28 days [DALYs per 100,000]	Exclusive breastfeeding, both sexes, <6 months [DALYs per 100,000]	Stunting, both sexes, <5 years [DALYs per 100,000]	Wasting, both sexes, <5 years [DALYs per 100,000]	Overweight, both sexes, 2−4 years [DALYs per 100,000]	Anaemia, females, 15−49 years [DALYs per 100,000]
Samoa	2012	501774 (400687 − 623500)	3668 (2039 − 5776)	843 (400 − 1364)	2119 (1327 − 3096)	31 (14 − 62)	953 (611 − 1416)
Samoa	2021	426899 (326938 − 563003)	2533 (1434 − 4140)	474 (211 − 885)	1300 (802 − 2013)	28 (12 − 54)	932 (573 − 1378)
Solomon Islands	2012	681374 (521352 − 866440)	8615 (5244 − 12685)	5219 (3194 − 7505)	5396 (3939 − 6813)	10 (4 − 20)	1213 (738 − 1818)
Solomon Islands	2021	569424 (431845 − 739714)	5672 (3362 − 8530)	2912 (1717 − 4351)	3379 (2478 − 4431)	9 (4 − 18)	1233 (769 − 1867)
Tokelau	2012	347784 (262676 − 440196)	3577 (2122 − 5450)	1047 (588 − 1634)	1541 (1064 − 2172)	32 (13 − 59)	941 (612 − 1368)
Tokelau	2021	1685944 (1491727 − 1841133)	23227 (13447 − 35989)	4431 (3141 − 6160)	7610 (5297 − 10429)	61 (26 − 114)	895 (570 − 1332)
Tonga	2012	419640 (333082 − 526390)	3811 (2179 − 5891)	871 (481 − 1384)	1745 (1287 − 2291)	57 (26 − 111)	918 (550 − 1412)
Tonga	2021	338556 (252441 − 445682)	2379 (1310 − 3830)	456 (258 − 765)	1119 (809 − 1513)	52 (22 − 98)	885 (526 − 1363)
Tuvalu	2012	702033 (551510 − 865725)	8025 (5137 − 12150)	2258 (1207 − 3540)	2637 (1957 − 3436)	25 (11 − 45)	1069 (656 − 1664)
Tuvalu	2021	583648 (431905 − 759058)	4919 (2827 − 7539)	1181 (643 − 1833)	1817 (1276 − 2441)	22 (10 − 41)	1015 (623 − 1533)
Vanuatu	2012	768140 (628969 − 936377)	10625 (6560 − 15878)	3717 (2121 − 5470)	4236 (2884 − 5466)	9 (4 − 16)	1175 (668 − 1852)
Vanuatu	2021	690234 (538728 − 869537)	6950 (4031 − 10626)	2110 (1131 − 3414)	2578 (1715 − 3714)	8 (3 − 14)	1174 (697 − 1773)

Table S1: Global, super−regional, regional, and country/territory nutrition indicator attributable disability−adjusted life years, 2012 and 2021

location_id	Year	Low birthweight, both sexes, <28 days [DALYs per 100,000]	Exclusive breastfeeding, both sexes, <6 months [DALYs per 100,000]	Stunting, both sexes, <5 years [DALYs per 100,000]	Wasting, both sexes, <5 years [DALYs per 100,000]	Overweight, both sexes, 2−4 years [DALYs per 100,000]	Anaemia, females, 15−49 years [DALYs per 100,000]
Southeast Asia	2012	1012413 (874745 − 1164463)	18135 (13186 − 23412)	4024 (2970 − 5115)	5271 (3222 − 6587)	19 (9 − 34)	788 (518 − 1136)
Southeast Asia	2021	774767 (654340 − 905907)	9319 (6676 − 12380)	1739 (1228 − 2381)	2561 (1458 − 3469)	18 (8 − 32)	744 (486 − 1098)
Cambodia	2012	1498738 (1236804 − 1782743)	25095 (15893 − 37486)	8289 (5803 − 11085)	8865 (6643 − 11775)	7 (3 − 13)	1215 (792 − 1773)
Cambodia	2021	1145409 (936749 − 1392354)	11300 (6666 − 16943)	3275 (2142 − 4530)	3636 (2728 − 4788)	7 (3 − 13)	1088 (689 − 1664)
Indonesia	2012	1132470 (927232 − 1370341)	20439 (14781 − 27038)	4292 (3041 − 5719)	6676 (3392 − 8785)	20 (9 − 36)	498 (304 − 738)
Indonesia	2021	863365 (691784 − 1055371)	10776 (7342 − 14761)	2021 (1380 − 2868)	3436 (1602 − 4909)	17 (8 − 30)	490 (296 − 759)
Laos	2012	1837892 (1538268 − 2155462)	62694 (42085 − 87494)	12194 (8037 − 17631)	13138 (7561 − 18727)	18 (7 − 36)	1073 (687 − 1586)
Laos	2021	1332256 (1024492 − 1651869)	25643 (16762 − 36262)	4338 (2735 − 6572)	5175 (3069 − 7275)	15 (6 − 30)	981 (612 − 1438)
Malaysia	2012	302965 (269353 − 347620)	2443 (1706 − 3309)	444 (321 − 580)	853 (658 − 1037)	17 (7 − 34)	1024 (588 − 1728)
Malaysia	2021	238969 (196126 − 285808)	1722 (1053 − 2575)	248 (163 − 350)	582 (421 − 761)	18 (7 − 35)	902 (498 − 1467)
Maldives	2012	789864 (670599 − 915037)	5858 (3939 − 8102)	1098 (776 − 1460)	1917 (1113 − 2554)	13 (5 − 24)	1854 (1239 − 2732)
Maldives	2021	537971 (428781 − 659208)	3418 (2136 − 5086)	517 (359 − 733)	984 (515 − 1450)	12 (5 − 22)	1901 (1217 − 2789)

Table S1: Global, super−regional, regional, and country/territory nutrition indicator attributable disability−adjusted life years, 2012 and 2021

location_id	Year	Low birthweight, both sexes, <28 days [DALYs per 100,000]	Exclusive breastfeeding, both sexes, <6 months [DALYs per 100,000]	Stunting, both sexes, <5 years [DALYs per 100,000]	Wasting, both sexes, <5 years [DALYs per 100,000]	Overweight, both sexes, 2−4 years [DALYs per 100,000]	Anaemia, females, 15−49 years [DALYs per 100,000]
Mauritius	2012	689976 (625454 − 753419)	5311 (3909 − 6869)	725 (505 − 951)	1289 (899 − 1533)	21 (8 − 41)	813 (493 − 1268)
Mauritius	2021	615002 (507894 − 714104)	3521 (2461 − 4631)	434 (284 − 599)	894 (631 − 1112)	21 (9 − 39)	774 (480 − 1150)
Myanmar	2012	1733328 (1479345 − 2009533)	44041 (27393 − 66074)	8865 (6191 − 12613)	9355 (5973 − 13182)	23 (9 − 43)	1337 (869 − 1952)
Myanmar	2021	1262854 (1015765 − 1555997)	18097 (11501 − 26456)	2876 (1788 − 4235)	3631 (2284 − 5004)	20 (9 − 39)	1242 (798 − 1882)
Philippines	2012	1035824 (877925 − 1228195)	14674 (10473 − 19674)	4311 (3119 − 5651)	5230 (3566 − 6491)	27 (12 − 50)	1021 (632 − 1513)
Philippines	2021	819559 (651363 − 1025056)	8426 (5811 − 11650)	1909 (1360 − 2690)	2573 (1643 − 3431)	26 (11 − 48)	970 (592 − 1446)
Seychelles	2012	593706 (511853 − 674409)	6274 (4246 − 8749)	745 (520 − 1026)	1130 (822 − 1374)	20 (8 − 37)	812 (507 − 1278)
Seychelles	2021	559253 (447152 − 703412)	4514 (2885 − 6500)	450 (283 − 652)	775 (549 − 1029)	20 (9 − 37)	780 (497 − 1206)
Sri Lanka	2012	485714 (440648 − 531637)	1381 (947 − 1827)	396 (275 − 528)	1160 (899 − 1403)	13 (5 − 25)	976 (621 − 1452)
Sri Lanka	2021	283863 (218369 − 375705)	648 (382 − 1019)	141 (86 − 218)	658 (471 − 884)	12 (5 − 26)	903 (557 − 1387)
Thailand	2012	273648 (242882 − 305154)	6795 (4992 − 8904)	794 (538 − 1090)	1181 (776 − 1467)	17 (7 − 32)	747 (472 − 1168)
Thailand	2021	192665 (164473 − 224466)	4582 (3147 − 6125)	472 (308 − 663)	797 (486 − 1039)	20 (8 − 39)	713 (450 − 1095)

Table S1: Global, super−regional, regional, and country/territory nutrition indicator attributable disability−adjusted life years, 2012 and 2021

location_id	Year	Low birthweight, both sexes, <28 days [DALYs per 100,000]	Exclusive breastfeeding, both sexes, <6 months [DALYs per 100,000]	Stunting, both sexes, <5 years [DALYs per 100,000]	Wasting, both sexes, <5 years [DALYs per 100,000]	Overweight, both sexes, 2−4 years [DALYs per 100,000]	Anaemia, females, 15−49 years [DALYs per 100,000]
Timor−Leste	2012	1369509 (1123079 − 1626068)	26105 (16673 − 38204)	9708 (6843 − 13265)	13310 (9369 − 18111)	44 (18 − 82)	611 (382 − 930)
Timor−Leste	2021	1144231 (953753 − 1356980)	15995 (10412 − 22638)	6044 (4071 − 8417)	8857 (6064 − 11807)	42 (18 − 79)	576 (357 − 921)
Viet Nam	2012	628181 (521462 − 745679)	7748 (4206 − 12372)	1419 (958 − 1947)	1186 (845 − 1576)	10 (4 − 20)	847 (528 − 1282)
Viet Nam	2021	430752 (336341 − 547274)	3851 (1994 − 6257)	540 (337 − 794)	457 (305 − 659)	10 (5 − 21)	790 (501 − 1187)
Sub−Saharan Africa	2012	2542897 (2291350 − 2778178)	57211 (42658 − 72504)	22630 (14767 − 31648)	28672 (14419 − 38701)	38 (18 − 66)	1427 (951 − 2057)
Sub−Saharan Africa	2021	2063366 (1735669 − 2429724)	28335 (20105 − 37752)	11099 (5940 − 17568)	13163 (6069 − 19924)	34 (16 − 59)	1353 (891 − 1943)
Central sub−Saharan Africa	2012	2032013 (1733211 − 2314834)	45330 (29498 − 69869)	18914 (8954 − 33139)	22201 (10835 − 34991)	42 (17 − 79)	1365 (893 − 1974)
Central sub−Saharan Africa	2021	1485049 (1206324 − 1761311)	14335 (9139 − 22153)	7039 (1641 − 16871)	6402 (3390 − 9833)	30 (13 − 57)	1189 (741 − 1770)
Angola	2012	2013255 (1682759 − 2361050)	43754 (29446 − 61974)	15764 (11252 − 20497)	20713 (10364 − 30619)	54 (22 − 103)	1238 (748 − 1837)
Angola	2021	1479380 (1229281 − 1755011)	14035 (8757 − 20738)	5736 (2553 − 11977)	6290 (3559 − 9227)	37 (16 − 67)	1134 (695 − 1722)
Central African Republic	2012	3210573 (2617352 − 3920448)	93856 (59047 − 145036)	31219 (19059 − 42734)	42348 (18918 − 65801)	67 (26 − 124)	1149 (711 − 1688)
Central African Republic	2021	2809618 (2287792 − 3439144)	59395 (36231 − 93403)	18209 (10030 − 27438)	26087 (11274 − 43346)	65 (26 − 115)	1127 (698 − 1687)

Table S1: Global, super−regional, regional, and country/territory nutrition indicator attributable disability−adjusted life years, 2012 and 2021

location_id	Year	Low birthweight, both sexes, <28 days [DALYs per 100,000]	Exclusive breastfeeding, both sexes, <6 months [DALYs per 100,000]	Stunting, both sexes, <5 years [DALYs per 100,000]	Wasting, both sexes, <5 years [DALYs per 100,000]	Overweight, both sexes, 2−4 years [DALYs per 100,000]	Anaemia, females, 15−49 years [DALYs per 100,000]
Congo (Brazzaville)	2012	1604499 (1354965 − 1900840)	35487 (20273 − 65483)	6461 (3339 − 11060)	10784 (3914 − 20138)	28 (12 − 55)	1642 (1073 − 2429)
Congo (Brazzaville)	2021	1218407 (954433 − 1504510)	14075 (6829 − 27300)	2721 (1031 − 6496)	4182 (1553 − 7848)	25 (11 − 46)	1486 (977 − 2256)
DR Congo	2012	1991394 (1644165 − 2327930)	43932 (25727 − 73008)	20403 (7656 − 40104)	22617 (10865 − 37766)	37 (15 − 74)	1388 (907 − 1998)
DR Congo	2021	1418914 (1100411 − 1767424)	11690 (6356 − 21914)	7234 (612 − 19210)	5467 (2766 − 9187)	26 (11 − 50)	1175 (706 − 1771)
Equatorial Guinea	2012	1544664 (1192212 − 1951479)	16340 (9389 − 27153)	5574 (1643 − 12984)	4187 (2277 − 6775)	49 (23 − 86)	1214 (720 − 1846)
Equatorial Guinea	2021	1263843 (909074 − 1706306)	8580 (4847 − 14449)	2842 (413 − 8303)	1985 (981 − 3426)	43 (19 − 79)	1211 (726 − 1837)
Gabon	2012	1730042 (1368453 − 2106097)	19781 (9892 − 37186)	4075 (2053 − 7082)	4649 (2299 − 7905)	20 (9 − 38)	2149 (1411 − 3100)
Gabon	2021	1211237 (905904 − 1611590)	8639 (3909 − 17111)	1590 (615 − 3488)	1824 (770 − 3591)	20 (9 − 39)	1974 (1264 − 2896)
Eastern sub−Saharan Africa	2012	2294592 (2007831 − 2617652)	37406 (27602 − 47891)	15101 (11185 − 18895)	24783 (15528 − 32223)	48 (22 − 84)	1115 (748 − 1612)
Eastern sub−Saharan Africa	2021	1799331 (1457256 − 2202313)	19322 (13661 − 25635)	7300 (4546 − 10548)	10742 (5866 − 15867)	45 (21 − 78)	1070 (721 − 1533)
Burundi	2012	2279254 (1973894 − 2623802)	22335 (12273 − 37001)	19947 (9449 − 30916)	21249 (11352 − 35223)	40 (16 − 78)	833 (507 − 1267)
Burundi	2021	1729031 (1372300 − 2148366)	8489 (3719 − 15634)	11602 (350 − 28025)	7956 (3224 − 16186)	30 (12 − 57)	877 (521 − 1363)

Table S1: Global, super−regional, regional, and country/territory nutrition indicator attributable disability−adjusted life years, 2012 and 2021

location_id	Year	Low birthweight, both sexes, <28 days [DALYs per 100,000]	Exclusive breastfeeding, both sexes, <6 months [DALYs per 100,000]	Stunting, both sexes, <5 years [DALYs per 100,000]	Wasting, both sexes, <5 years [DALYs per 100,000]	Overweight, both sexes, 2−4 years [DALYs per 100,000]	Anaemia, females, 15−49 years [DALYs per 100,000]
Comoros	2012	2909049 (2331779 − 3590446)	34445 (19531 − 55929)	9849 (6833 − 13544)	13681 (9007 − 19227)	95 (43 − 166)	854 (508 − 1348)
Comoros	2021	2184899 (1767408 − 2642511)	18892 (11106 − 29419)	4953 (3466 − 6866)	7725 (5138 − 10361)	89 (41 − 164)	796 (437 − 1278)
Djibouti	2012	1610914 (1287822 − 1959969)	32229 (18961 − 53097)	9565 (6046 − 13177)	21334 (13638 − 29611)	54 (24 − 105)	790 (470 − 1211)
Djibouti	2021	1148695 (927560 − 1404477)	13140 (7136 − 21508)	3621 (2225 − 5215)	8375 (5180 − 11688)	45 (21 − 86)	764 (436 − 1172)
Eritrea	2012	1826687 (1493363 − 2255406)	24263 (14190 − 39040)	13980 (9557 − 19270)	25331 (15147 − 34953)	29 (12 − 51)	906 (545 − 1421)
Eritrea	2021	1434293 (1125181 − 1825284)	11825 (6113 − 19444)	6068 (3999 − 8880)	12404 (7441 − 17822)	31 (13 − 56)	872 (528 − 1361)
Ethiopia	2012	2686596 (2317939 − 3081515)	25824 (16937 − 36162)	13478 (10149 − 17027)	20128 (12515 − 26313)	20 (9 − 37)	758 (505 − 1099)
Ethiopia	2021	2012377 (1608311 − 2488652)	11628 (7317 − 17263)	5100 (3517 − 6942)	9295 (5215 − 13299)	17 (7 − 31)	674 (448 − 994)
Kenya	2012	1687724 (1410317 − 1959482)	49003 (34819 − 63190)	8054 (5897 − 10383)	14936 (7420 − 21256)	20 (8 − 38)	694 (470 − 987)
Kenya	2021	1260216 (1031235 − 1543152)	17318 (12265 − 22725)	3437 (2398 − 4771)	6584 (3405 − 9821)	18 (7 − 36)	619 (421 − 889)
Madagascar	2012	2294952 (1989499 − 2614580)	65346 (43078 − 93716)	22083 (16095 − 28562)	39635 (18956 − 58210)	66 (30 − 123)	1094 (688 − 1670)
Madagascar	2021	1770361 (1409256 − 2181768)	34266 (20119 − 51961)	9527 (6188 − 13516)	17535 (7594 − 28016)	59 (23 − 113)	1052 (625 − 1645)

Table S1: Global, super−regional, regional, and country/territory nutrition indicator attributable disability−adjusted life years, 2012 and 2021

location_id	Year	Low birthweight, both sexes, <28 days [DALYs per 100,000]	Exclusive breastfeeding, both sexes, <6 months [DALYs per 100,000]	Stunting, both sexes, <5 years [DALYs per 100,000]	Wasting, both sexes, <5 years [DALYs per 100,000]	Overweight, both sexes, 2−4 years [DALYs per 100,000]	Anaemia, females, 15−49 years [DALYs per 100,000]
Malawi	2012	2120186 (1830168 − 2459355)	28289 (19195 − 38899)	14494 (9926 − 19432)	17517 (10006 − 24899)	78 (35 − 135)	1137 (693 − 1755)
Malawi	2021	1602904 (1317563 − 1918076)	13497 (8141 − 21147)	6312 (3419 − 9790)	7448 (3816 − 12081)	67 (31 − 118)	1060 (637 − 1691)
Mozambique	2012	2486737 (2064818 − 2960304)	43617 (27428 − 63140)	15089 (9191 − 21612)	16703 (8716 − 24708)	49 (22 − 94)	3194 (2253 − 4510)
Mozambique	2021	1908229 (1456052 − 2523219)	17326 (10217 − 27435)	6281 (2727 − 12215)	6998 (4002 − 11233)	45 (20 − 91)	3700 (2620 − 5126)
Rwanda	2012	1678653 (1374733 − 2017225)	10584 (6376 − 16594)	12999 (9147 − 17384)	12082 (6812 − 17954)	118 (51 − 224)	531 (340 − 786)
Rwanda	2021	1167305 (905129 − 1475683)	4742 (2530 − 8172)	5093 (3187 − 7563)	5022 (2917 − 8101)	110 (47 − 206)	459 (280 − 699)
Somalia	2012	2972305 (2301243 − 3654326)	101906 (62034 − 147885)	29688 (20390 − 39518)	110660 (88233 − 129687)	56 (23 − 116)	1024 (607 − 1617)
Somalia	2021	2486381 (1981036 − 3053171)	72472 (42875 − 105995)	18113 (9047 − 27665)	31250 (16661 − 46503)	44 (18 − 87)	1045 (629 − 1610)
South Sudan	2012	3023214 (2417496 − 3619840)	87905 (53505 − 131378)	26429 (17806 − 34542)	57003 (27111 − 80719)	74 (31 − 138)	965 (563 − 1504)
South Sudan	2021	3086861 (2314956 − 3998677)	72675 (44274 − 113244)	22148 (12836 − 31690)	48002 (22086 − 71825)	93 (39 − 181)	938 (546 − 1448)
Uganda	2012	2039259 (1777836 − 2318454)	16879 (10159 − 29785)	12322 (6810 − 20356)	13384 (7833 − 20439)	62 (27 − 122)	895 (578 − 1381)
Uganda	2021	1594884 (1297431 − 1962321)	7408 (3659 − 13894)	6490 (1236 − 18546)	5884 (3088 − 10073)	68 (28 − 126)	718 (444 − 1134)

Table S1: Global, super−regional, regional, and country/territory nutrition indicator attributable disability−adjusted life years, 2012 and 2021

location_id	Year	Low birthweight, both sexes, <28 days [DALYs per 100,000]	Exclusive breastfeeding, both sexes, <6 months [DALYs per 100,000]	Stunting, both sexes, <5 years [DALYs per 100,000]	Wasting, both sexes, <5 years [DALYs per 100,000]	Overweight, both sexes, 2−4 years [DALYs per 100,000]	Anaemia, females, 15−49 years [DALYs per 100,000]
Tanzania	2012	2094576 (1756034 − 2470946)	29929 (19141 − 43675)	13985 (10340 − 17664)	16762 (9675 − 23416)	63 (28 − 120)	1372 (904 − 2037)
Tanzania	2021	1635518 (1245871 − 2111562)	13159 (7743 − 20478)	6610 (4316 − 10012)	7178 (4225 − 10910)	69 (29 − 135)	1318 (812 − 2034)
Zambia	2012	1917046 (1584341 − 2272505)	24426 (16187 − 34834)	16482 (12039 − 20512)	19077 (10607 − 26451)	68 (32 − 116)	1790 (1185 − 2500)
Zambia	2021	1386057 (1039020 − 1802547)	10068 (5755 − 16016)	6484 (3844 − 10095)	6537 (3583 − 10571)	46 (23 − 83)	1231 (783 − 1856)
Southern sub−Saharan Africa	2012	1648050 (1478902 − 1820645)	56833 (45043 − 68744)	9656 (6998 − 12862)	16286 (8680 − 21910)	18 (9 − 32)	1124 (735 − 1616)
Southern sub−Saharan Africa	2021	1612307 (1325290 − 1949950)	31957 (23959 − 41092)	5066 (3563 − 7130)	9626 (5334 − 13946)	21 (10 − 36)	1053 (680 − 1530)
Botswana	2012	1819613 (1438214 − 2284961)	36822 (25652 − 50345)	9364 (6515 − 12783)	14426 (6776 − 21220)	36 (14 − 66)	840 (493 − 1282)
Botswana	2021	1600373 (1213379 − 2083989)	25474 (16210 − 36301)	5835 (3820 − 8509)	10420 (4574 − 17038)	44 (18 − 82)	781 (467 − 1170)
Eswatini	2012	1307435 (1096239 − 1527027)	77895 (53624 − 102530)	13472 (8551 − 18481)	15775 (6575 − 27261)	37 (17 − 70)	877 (538 − 1317)
Eswatini	2021	1115537 (859818 − 1427232)	36367 (23971 − 51527)	6320 (4033 − 9564)	8121 (3837 − 14211)	48 (22 − 86)	820 (500 − 1226)
Lesotho	2012	2610197 (2115532 − 3187718)	82448 (58402 − 111846)	17415 (11566 − 22899)	25122 (9071 − 39073)	11 (5 − 20)	993 (647 − 1430)
Lesotho	2021	2355742 (1868033 − 2899826)	63425 (42164 − 91005)	13252 (8313 − 18950)	18724 (7199 − 30540)	11 (4 − 20)	906 (573 − 1378)

Table S1: Global, super−regional, regional, and country/territory nutrition indicator attributable disability−adjusted life years, 2012 and 2021

location_id	Year	Low birthweight, both sexes, <28 days [DALYs per 100,000]	Exclusive breastfeeding, both sexes, <6 months [DALYs per 100,000]	Stunting, both sexes, <5 years [DALYs per 100,000]	Wasting, both sexes, <5 years [DALYs per 100,000]	Overweight, both sexes, 2−4 years [DALYs per 100,000]	Anaemia, females, 15−49 years [DALYs per 100,000]
Namibia	2012	1559367 (1297245 − 1843674)	45686 (30220 − 61531)	6937 (4587 − 9626)	15223 (6010 − 23280)	13 (5 − 25)	601 (366 − 906)
Namibia	2021	1241129 (963658 − 1564247)	25475 (16272 − 35022)	3470 (2225 − 5075)	9104 (3879 − 15188)	15 (6 − 28)	540 (317 − 845)
South Africa	2012	1435721 (1246775 − 1626204)	59501 (47743 − 72592)	8873 (5937 − 11917)	15308 (7772 − 20784)	21 (10 − 36)	1182 (773 − 1700)
South Africa	2021	1508871 (1229918 − 1845752)	32661 (24304 − 42664)	4069 (2740 − 5706)	7793 (3614 − 11604)	24 (12 − 41)	1109 (717 − 1627)
Zimbabwe	2012	2069766 (1824810 − 2309624)	50008 (34747 − 66600)	10909 (8216 − 13966)	18162 (11225 − 23660)	10 (4 − 19)	1056 (697 − 1568)
Zimbabwe	2021	1841786 (1530789 − 2225856)	28836 (18112 − 39703)	6590 (4725 − 9321)	12982 (8978 − 17461)	11 (5 − 21)	1004 (622 − 1510)
Western sub−Saharan Africa	2012	2994004 (2736370 − 3236715)	77155 (57185 − 99238)	31838 (19497 − 44572)	35420 (14705 − 49407)	30 (14 − 55)	1796 (1199 − 2594)
Western sub−Saharan Africa	2021	2454545 (2115963 − 2831925)	38534 (26439 − 51869)	15806 (8156 − 24966)	17231 (7106 − 26409)	28 (13 − 51)	1705 (1117 − 2477)
Benin	2012	2858029 (2458682 − 3270190)	33785 (19854 − 54035)	26381 (13585 − 41353)	21848 (11834 − 34945)	35 (14 − 63)	1863 (1208 − 2752)
Benin	2021	2315818 (1960176 − 2659450)	15149 (8212 − 25420)	11282 (3487 − 22803)	8282 (4165 − 14990)	30 (13 − 54)	1787 (1102 − 2677)
Burkina Faso	2012	2466401 (2101610 − 2818926)	70972 (44975 − 109207)	28733 (13505 − 51433)	38510 (19256 − 54612)	51 (22 − 96)	1571 (986 − 2332)
Burkina Faso	2021	2048065 (1695095 − 2457679)	37425 (22590 − 56207)	15077 (7189 − 29524)	20100 (10941 − 29161)	47 (19 − 93)	1460 (896 − 2203)

Table S1: Global, super−regional, regional, and country/territory nutrition indicator attributable disability−adjusted life years, 2012 and 2021

location_id	Year	Low birthweight, both sexes, <28 days [DALYs per 100,000]	Exclusive breastfeeding, both sexes, <6 months [DALYs per 100,000]	Stunting, both sexes, <5 years [DALYs per 100,000]	Wasting, both sexes, <5 years [DALYs per 100,000]	Overweight, both sexes, 2−4 years [DALYs per 100,000]	Anaemia, females, 15−49 years [DALYs per 100,000]
Cabo Verde	2012	969702<br>(803367 − 1158510)	10164<br>(6594 − 15090)	1509<br>(993 − 2119)	2544<br>(1191 − 4034)	12<br>(4 − 25)	1000<br>(599 − 1552)
Cabo Verde	2021	590315<br>(429959 − 795637)	5137<br>(2728 − 8639)	534<br>(314 − 811)	1142<br>(481 − 1996)	14<br>(6 − 31)	961<br>(565 − 1464)
Cameroon	2012	1938796<br>(1649856 − 2248210)	47831<br>(29866 − 78321)	19827<br>(12066 − 29059)	17573<br>(8200 − 29831)	28<br>(13 − 52)	1228<br>(787 − 1790)
Cameroon	2021	1600465<br>(1311209 − 1943706)	21346<br>(11799 − 36932)	8363<br>(3939 − 15939)	6508<br>(2973 − 11454)	23<br>(10 − 43)	1124<br>(722 − 1715)
Chad	2012	2658189<br>(2251537 − 3102262)	142399<br>(92967 − 209651)	42412<br>(33208 − 52578)	67499<br>(25415 − 105619)	23<br>(10 − 44)	1764<br>(1054 − 2714)
Chad	2021	2461251<br>(2003896 − 3055986)	104787<br>(63618 − 162461)	26251<br>(19098 − 34249)	44927<br>(14637 − 74811)	22<br>(9 − 40)	1708<br>(1068 − 2615)
Côte d'Ivoire	2012	3006068<br>(2514448 − 3493319)	56215<br>(32895 − 89914)	17296<br>(8611 − 31408)	17245<br>(8710 − 28227)	15<br>(7 − 27)	1749<br>(1138 − 2516)
Côte d'Ivoire	2021	2362701<br>(1966639 − 2850139)	24016<br>(13796 − 37777)	7359<br>(3633 − 14287)	7129<br>(3581 − 11384)	14<br>(7 − 28)	1589<br>(959 − 2356)
Ghana	2012	2098257<br>(1817950 − 2372647)	15376<br>(10033 − 23457)	7315<br>(3097 − 15868)	9206<br>(5662 − 12665)	8<br>(4 − 15)	1748<br>(1130 − 2610)
Ghana	2021	1506279<br>(1147568 − 1978018)	7349<br>(4150 − 11847)	2589<br>(1192 − 5752)	3911<br>(2275 − 5842)	9<br>(3 − 16)	1520<br>(929 − 2270)
Guinea	2012	2751166<br>(2331964 − 3182725)	57100<br>(33688 − 88952)	28067<br>(15509 − 46370)	28184<br>(18088 − 40792)	26<br>(11 − 47)	1564<br>(1016 − 2260)
Guinea	2021	2192709<br>(1789869 − 2662328)	26776<br>(13993 − 43454)	13367<br>(6473 − 24636)	12523<br>(7980 − 18751)	22<br>(10 − 40)	1445<br>(938 − 2113)

Table S1: Global, super−regional, regional, and country/territory nutrition indicator attributable disability−adjusted life years, 2012 and 2021

location_id	Year	Low birthweight, both sexes, <28 days [DALYs per 100,000]	Exclusive breastfeeding, both sexes, <6 months [DALYs per 100,000]	Stunting, both sexes, <5 years [DALYs per 100,000]	Wasting, both sexes, <5 years [DALYs per 100,000]	Overweight, both sexes, 2−4 years [DALYs per 100,000]	Anaemia, females, 15−49 years [DALYs per 100,000]
Guinea−Bissau	2012	3031836 (2538499 − 3560479)	31053 (19033 − 49528)	18304 (12692 − 25001)	15514 (7759 − 23077)	19 (8 − 35)	1715 (1075 − 2634)
Guinea−Bissau	2021	2257302 (1804755 − 2765594)	12679 (6978 − 20559)	7500 (4666 − 11181)	6003 (3123 − 9690)	14 (6 − 27)	1616 (978 − 2567)
Liberia	2012	2400465 (1985233 − 2822757)	48936 (28138 − 85772)	14799 (8191 − 22837)	21655 (6878 − 39672)	22 (9 − 41)	3279 (2228 − 4638)
Liberia	2021	1976441 (1537736 − 2524145)	20305 (10290 − 36118)	6863 (2449 − 14015)	8477 (3211 − 16763)	18 (8 − 32)	3393 (2179 − 4885)
Mali	2012	4012214 (3606296 − 4429317)	42802 (27531 − 63323)	23037 (10860 − 42196)	34828 (22346 − 48100)	14 (6 − 33)	1999 (1254 − 2877)
Mali	2021	3290139 (2768888 − 3849707)	19512 (11801 − 30028)	11283 (6001 − 20790)	21317 (13942 − 29456)	10 (4 − 22)	2091 (1334 − 3130)
Mauritania	2012	2361609 (2014989 − 2748195)	25173 (13606 − 43370)	6439 (4737 − 8399)	11580 (5471 − 19073)	13 (6 − 28)	1369 (854 − 2105)
Mauritania	2021	1655798 (1395021 − 1978368)	10039 (5343 − 17508)	2409 (1748 − 3305)	4351 (2130 − 7388)	15 (6 − 32)	1232 (757 − 1922)
Niger	2012	2387318 (1869272 − 2982841)	71042 (46662 − 107182)	39486 (19355 − 60788)	40384 (18404 − 61846)	19 (8 − 38)	1407 (882 − 2122)
Niger	2021	2070832 (1747197 − 2505081)	38353 (24314 − 58192)	20809 (8766 − 37968)	20017 (8704 − 32826)	16 (7 − 31)	1391 (880 − 2132)
Nigeria	2012	3393049 (3054757 − 3793970)	101733 (74730 − 129580)	41772 (25808 − 55030)	43684 (14817 − 60068)	39 (17 − 74)	1907 (1270 − 2754)
Nigeria	2021	2802079 (2348501 − 3351092)	48281 (33120 − 65132)	20714 (10397 − 31334)	19914 (6549 − 31694)	38 (16 − 72)	1821 (1193 − 2687)

Table S1: Global, super−regional, regional, and country/territory nutrition indicator attributable disability−adjusted life years, 2012 and 2021

location_id	Year	Low birthweight, both sexes, <28 days [DALYs per 100,000]	Exclusive breastfeeding, both sexes, <6 months [DALYs per 100,000]	Stunting, both sexes, <5 years [DALYs per 100,000]	Wasting, both sexes, <5 years [DALYs per 100,000]	Overweight, both sexes, 2−4 years [DALYs per 100,000]	Anaemia, females, 15−49 years [DALYs per 100,000]
São Tomé and Príncipe	2012	1161450 (987855 − 1375079)	11081 (6109 − 19160)	5350 (2478 − 10333)	5404 (3393 − 8013)	13 (5 − 27)	1264 (802 − 1880)
São Tomé and Príncipe	2021	644749 (458148 − 854178)	4341 (2350 − 7359)	1167 (695 − 1810)	1957 (1215 − 2788)	13 (5 − 27)	1160 (724 − 1752)
Senegal	2012	2385962 (2124679 − 2689889)	33426 (20502 − 49594)	8524 (6341 − 11234)	11358 (5891 − 16757)	9 (4 − 18)	2078 (1376 − 3036)
Senegal	2021	1699190 (1381933 − 2056988)	13203 (7063 − 20891)	2645 (1806 − 3705)	3693 (1889 − 5429)	8 (3 − 17)	1941 (1218 − 2912)
Sierra Leone	2012	3054629 (2661107 − 3411757)	53196 (34916 − 77270)	28367 (13389 − 49979)	53994 (36056 − 79184)	45 (20 − 83)	1448 (939 − 2164)
Sierra Leone	2021	2383961 (1908902 − 2860021)	18498 (10615 − 29020)	10775 (3557 − 27238)	25263 (16401 − 37430)	40 (18 − 74)	1371 (850 − 2029)
The Gambia	2012	2450742 (2087831 − 2823156)	15661 (9940 − 23320)	7156 (4189 − 11299)	10522 (5148 − 15570)	7 (3 − 14)	2078 (1355 − 3047)
The Gambia	2021	1860854 (1469929 − 2329564)	7863 (4846 − 12556)	3132 (2206 − 4271)	5545 (2990 − 8207)	6 (2 − 13)	1957 (1272 − 2807)
Togo	2012	2346474 (2042606 − 2683945)	39792 (23918 − 62703)	15568 (9328 − 22752)	19055 (5808 − 33357)	16 (7 − 31)	1645 (1038 − 2426)
Togo	2021	1817823 (1466541 − 2221334)	17994 (10128 − 28743)	6531 (3394 − 10969)	7589 (2351 − 13930)	16 (6 − 31)	1659 (1012 − 2528)

Table S2: Spearman's rank correlation coefficients, by sex, standard GBD locations, 1990 − 2021

Indicator	Sex	Spearman's rank rho
LBW	Male	−0.606
LBW	Female	−0.4563
EBF	Male	0.01902
EBF	Female	0.01899
Stunting	Male	−0.8608
Stunting	Female	−0.8535
Wasting	Male	−0.7425
Wasting	Female	−0.7433
Overweight	Male	0.5913
Overweight	Female	0.5109
Anaemia	Female	−0.7512

Table S3. Ratio of projected 2030 to estimated 2012: livebirths, population of children under 5 years, population of females 15 to 49 years, and general fertility rate in 28 countries projected to meet the stunting Global Nutrition Target in 2030

Country	$\frac{births_{2030}}{births_{2012}}$	$\frac{pop_{\text{child 2030}}}{pop_{\text{child 2012}}}$	$\frac{pop_{15-49\text{ female 2030}}}{pop_{15-49\text{ female 2012}}}$	$\frac{GFR_{2030}}{GFR_{2012}}$
American Samoa	0.81	0.73	0.99	0.82
Armenia	0.51	0.56	0.78	0.66
Bangladesh	0.66	0.69	1.21	0.54
Bhutan	0.76	0.82	1.18	0.64
Cabo Verde	0.65	0.68	1.20	0.54
China	0.63	0.62	0.80	0.79
Cook Islands	0.84	0.77	0.96	0.88
Costa Rica	0.68	0.71	1.06	0.64
Democratic People's Republic of Korea	0.67	0.72	0.92	0.73
El Salvador	0.69	0.74	1.00	0.69
Georgia	0.70	0.74	0.79	0.89
Guyana	0.68	0.75	0.87	0.78
Iran (Islamic Republic of)	0.62	0.69	1.05	0.59
Latvia	0.60	0.63	0.69	0.86
Lithuania	0.57	0.61	0.68	0.84
Morocco	0.67	0.76	1.09	0.62
Northern Mariana Islands	0.87	0.68	0.83	1.05
Palau	0.70	0.68	0.77	0.92
Paraguay	0.87	0.88	1.24	0.70
Puerto Rico	0.42	0.39	0.72	0.58
Republic of Korea	0.57	0.59	0.79	0.72
Republic of Moldova	0.44	0.47	0.69	0.64
Sao Tome and Principe	0.79	0.8	1.43	0.55
Syrian Arab Republic	0.51	0.46	0.73	0.70
Tonga	0.79	0.85	1.02	0.77
Türkiye	0.70	0.75	1.06	0.66
Ukraine	0.45	0.47	0.79	0.57
United States Virgin Islands	0.49	0.5	0.63	0.77

Table S4. Annualised rates of change (ARC) 2012 to 2021: observed prevalence, expected prevalence, difference, attributable burden

	Low birthweight both sexes, birth	Exclusive breastfeeding, both sexes, age <6 months	Child stunting, both sexes, age <5 years	Child wasting, both sexes, age <5 years	Child overweight, both sexes, age 2–4 years	Anaemia, females, age 15–49 years
**Global**						
**Observed prevalence ARC, mean (95% UI)**	**-0·39% (-0·50, -0·28)***	**1·11% (0·93, 1·29)***	**-1·69% (-1·78, -1·59)***	**-2·86% (-2·93, -2·78)***	**2·32% (1·94, 2·73)***	**0·47% (0·29, 0·67)***
**Expected prevalence ARC**	**-0·97%**	**0·11%**	**-2·69%**	**-2·76%**	**1·21%**	**-1·48%**
**Prevalence ARC difference**	**0·58%**	**1·00%**	**1·00%**	**-0·09%**	**1·11%**	**1·94%**
**Attributable burden ARC**	**-2·35%**	**-7·49%**	**-8·23%**	**-8·65%**	**-0·11%**	**-0·12%**
**Central Europe, eastern Europe, and central Asia**						
**Observed prevalence ARC, mean (95% UI)**	**-0·09% (-0·29, 0·12)***	**0·74% (0·19, 1·36)***	**-1·58% (-1·97, -1·18)***	**-2·06% (-2·31, -1·80)***	**1·08% (0·16, 1·99)***	**0·05% (-0·51, 0·60)***
**Expected prevalence ARC**	**-0·40%**	**0·00%**	**-2·01%**	**-2·01%**	**0·51%**	**-1·37%**
**Prevalence ARC difference**	**0·31%**	**0·74%**	**0·43%**	**-0·04%**	**0·57%**	**1·42%**
**Attributable burden ARC**	**-3·07%**	**-3·96%**	**-8·91%**	**-7·26%**	**1·32%**	**-0·20%**
Central Asia						
Observed prevalence ARC, mean (95% UI)	-0·40% (-0·88, 0·09)*	0·57% (-0·11, 1·27)*	-2·95% (-3·39, -2·51)*	-2·96% (-3·27, -2·66)*	0·89% (-0·14, 1·91)*	-0·12% (-0·84, 0·58)
Expected prevalence ARC	-0·57%	0·00%	-2·42%	-2·53%	0·73%	-1·48%
Prevalence ARC difference	0·18%	0·56%	-0·53%	-0·43%	0·16%	1·36%
Attributable burden ARC	-2·96%	-5·76%	-10·69%	-9·34%	0·26%	-0·52%
Armenia						
Observed prevalence ARC, mean (95% UI)	0·45% (-0·38, 1·32)*	0·75% (-0·27, 1·74)*	-2·20% (-3·19, -1·24)*	-0·87% (-1·61, -0·18)*	0·82% (-1·19, 2·60)*	0·51% (-0·76, 2·21)*
Expected prevalence ARC	-0·27%	0·00%	-2·97%	-3·27%	0·78%	-2·17%
Prevalence ARC difference	0·72%	0·75%	0·77%	2·40%	0·04%	2·68%
Attributable burden ARC	-3·48%	-4·22%	-6·63%	-7·12%	0·15%	0·01%
Azerbaijan						
Observed prevalence ARC, mean (95% UI)	-0·32% (-1·42, 0·80)	1·00% (-1·45, 3·45)*	-1·28% (-2·12, -0·47)*	-1·89% (-2·50, -1·32)*	0·48% (-1·58, 2·62)*	-0·28% (-1·28, 1·05)
Expected prevalence ARC	-0·25%	0·00%	-2·62%	-2·93%	0·68%	-1·91%
Prevalence ARC difference	-0·07%	1·00%	1·34%	1·04%	-0·20%	1·63%
Attributable burden ARC	-3·12%	-5·04%	-9·56%	-8·81%	-0·35%	-0·39%
Georgia						
Observed prevalence ARC, mean (95% UI)	0·27% (-0·60, 1·13)*	0·29% (-0·98, 1·38)*	-3·44% (-4·58, -2·32)*	-4·62% (-5·30, -3·94)*	-0·54% (-2·64, 1·75)	-0·13% (-1·05, 0·97)
Expected prevalence ARC	-0·25%	0·00%	-4·04%	-4·08%	0·98%	-2·92%
Prevalence ARC difference	0·51%	0·29%	0·60%	-0·54%	-1·52%	2·79%
Attributable burden ARC	-6·54%	-7·60%	-12·41%	-12·25%	-1·27%	-0·22%
Kazakhstan						
Observed prevalence ARC, mean (95% UI)	-0·30% (-1·58, 0·95)	1·41% (0·11, 2·59)*	-3·04% (-4·10, -1·99)*	-3·07% (-3·61, -2·52)*	0·76% (-1·18, 2·66)*	-0·56% (-2·00, 0·87)
Expected prevalence ARC	-0·15%	0·00%	-1·94%	-2·00%	0·63%	-1·43%
Prevalence ARC difference	-0·15%	1·41%	-1·09%	-1·07%	0·13%	0·86%
Attributable burden ARC	-7·22%	-7·85%	-16·19%	-13·46%	-0·16%	-1·10%
Kyrgyzstan						
Observed prevalence ARC, mean (95% UI)	-0·23% (-1·44, 0·93)	0·22% (-0·69, 0·97)*	-1·96% (-3·01, -1·03)*	-2·99% (-3·64, -2·38)*	0·91% (-1·34, 3·14)*	-0·41% (-1·35, 0·74)
Expected prevalence ARC	-1·17%	0·00%	-3·22%	-3·08%	0·94%	-1·17%
Prevalence ARC difference	0·94%	0·22%	1·27%	0·10%	-0·03%	0·76%
Attributable burden ARC	-1·45%	-8·05%	-11·30%	-12·32%	0·47%	-0·66%

ARC= annualised rate of change. *denotes substantial increase or decrease, based upon consistent direction of mean and >80% of model posteriors.
Prevalence ARC difference= Observed mean ARC - Expected ARC.
Low birthweight prevalence ARC is at birth. Low birthweight attributable burden ARC is in neonatal age group (0 – 27 days).
ARC in attributable burden rate [disability adjusted life-years per 100,000], except for anaemia.
ARC in burden rate for anaemia [years lived with disability per 100,000]

Table S4. Annualised rates of change (ARC) 2012 to 2021: observed prevalence, expected prevalence, difference, attributable burden

	Low birthweight both sexes, birth	Exclusive breastfeeding, both sexes, age <6 months	Child stunting, both sexes, age <5 years	Child wasting, both sexes, age <5 years	Child overweight, both sexes, age 2–4 years	Anaemia, females, age 15–49 years
Mongolia						
Observed prevalence ARC, mean (95% UI)	-0·07% (-1·39, 1·27)	0·04% (-0·53, 0·61)*	-3·00% (-4·07, -1·98)*	-4·56% (-5·19, -3·92)*	-0·12% (-2·30, 2·07)	-0·39% (-1·13, 0·37)*
Expected prevalence ARC	-0·80%	0·00%	-2·48%	-2·54%	0·76%	-1·09%
Prevalence ARC difference	0·73%	0·04%	-0·52%	-2·02%	-0·88%	0·71%
Attributable burden ARC	-4·33%	-8·31%	-14·00%	-13·13%	-1·26%	-0·74%
Tajikistan						
Observed prevalence ARC, mean (95% UI)	-0·57% (-1·68, 0·55)*	0·55% (-0·69, 1·71)*	-2·56% (-3·35, -1·82)*	-2·85% (-3·34, -2·32)*	1·11% (-1·28, 3·39)*	1·54% (0·52, 2·92)*
Expected prevalence ARC	-1·14%	0·01%	-1·78%	-1·50%	0·83%	-0·42%
Prevalence ARC difference	0·57%	0·54%	-0·78%	-1·35%	0·28%	1·96%
Attributable burden ARC	-2·25%	-4·47%	-7·04%	-6·11%	0·28%	-0·62%
Turkmenistan						
Observed prevalence ARC, mean (95% UI)	-0·24% (-1·56, 1·20)	2·12% (1·08, 3·17)*	-4·35% (-5·38, -3·29)*	-2·28% (-2·81, -1·71)*	0·92% (-1·49, 3·35)*	-0·30% (-1·55, 0·98)
Expected prevalence ARC	-0·50%	0·00%	-3·53%	-4·04%	1·01%	-2·44%
Prevalence ARC difference	0·26%	2·12%	-0·82%	1·76%	-0·09%	2·14%
Attributable burden ARC	-2·63%	-4·35%	-9·28%	-8·05%	0·11%	-0·81%
Uzbekistan						
Observed prevalence ARC, mean (95% UI)	-0·10% (-1·31, 1·15)	-0·37% (-2·43, 1·55)	-3·90% (-5·02, -2·81)*	-3·47% (-4·14, -2·82)*	1·09% (-0·95, 3·08)*	-0·40% (-1·73, 0·94)
Expected prevalence ARC	-0·47%	0·00%	-2·87%	-3·30%	0·79%	-1·88%
Prevalence ARC difference	0·37%	-0·37%	-1·03%	-0·17%	0·29%	1·48%
Attributable burden ARC	-1·94%	-6·73%	-14·16%	-13·80%	0·55%	-0·57%
Central Europe						
Observed prevalence ARC, mean (95% UI)	0·05% (-0·23, 0·34)*	0·46% (-0·03, 0·99)*	-0·76% (-1·23, -0·30)*	-1·49% (-1·78, -1·23)*	1·17% (0·27, 2·07)*	0·09% (-0·67, 0·83)*
Expected prevalence ARC	-0·15%	0·00%	-2·89%	-2·26%	0·58%	-1·74%
Prevalence ARC difference	0·20%	0·46%	2·12%	0·77%	0·59%	1·83%
Attributable burden ARC	-4·16%	-3·30%	-8·45%	-5·47%	3·06%	-0·53%
Albania						
Observed prevalence ARC, mean (95% UI)	-0·17% (-1·41, 1·12)	-1·33% (-3·63, 0·97)*	-2·57% (-3·27, -1·85)*	-4·72% (-5·31, -4·11)*	-0·09% (-2·03, 1·81)	0·53% (-1·30, 2·39)*
Expected prevalence ARC	-0·31%	0·00%	-3·43%	-3·76%	0·92%	-2·51%
Prevalence ARC difference	0·13%	-1·33%	0·86%	-0·96%	-1·01%	3·04%
Attributable burden ARC	-2·75%	-8·35%	-9·13%	-9·52%	-0·43%	-0·24%
Bosnia and Herzegovina						
Observed prevalence ARC, mean (95% UI)	-0·10% (-1·50, 1·24)	0·55% (-1·68, 2·78)*	-0·29% (-1·30, 0·71)	-0·52% (-1·18, 0·15)*	0·92% (-0·87, 2·76)*	0·22% (-1·23, 1·78)*
Expected prevalence ARC	-0·18%	0·00%	-2·80%	-2·88%	0·71%	-2·05%
Prevalence ARC difference	0·08%	0·55%	2·51%	2·36%	0·21%	2·27%
Attributable burden ARC	-0·96%	-2·36%	-4·81%	-3·34%	0·64%	-0·35%
Bulgaria						
Observed prevalence ARC, mean (95% UI)	0·02% (-0·86, 0·87)*	0·48% (-1·28, 2·20)*	-0·60% (-1·99, 0·80)	-0·92% (-1·76, -0·10)*	0·84% (-1·19, 3·00)*	0·11% (-1·44, 1·65)*
Expected prevalence ARC	-0·13%	0·00%	-2·50%	-1·99%	0·60%	-1·58%
Prevalence ARC difference	0·15%	0·48%	1·90%	1·07%	0·24%	1·69%
Attributable burden ARC	-5·12%	-4·62%	-8·74%	-6·39%	0·63%	-0·52%

ARC= annualised rate of change. *denotes substantial increase or decrease, based upon consistent direction of mean and >80% of model posteriors.
Prevalence ARC difference= Observed mean ARC - Expected ARC.
Low birthweight prevalence ARC is at birth. Low birthweight attributable burden ARC is in neonatal age group (0 – 27 days).
ARC in attributable burden rate [disability adjusted life-years per 100,000], except for anaemia.
ARC in burden rate for anaemia [years lived with disability per 100,000]

Table S4. Annualised rates of change (ARC) 2012 to 2021: observed prevalence, expected prevalence, difference, attributable burden

	**Low birthweight both sexes, birth**	**Exclusive breastfeeding, both sexes, age <6 months**	**Child stunting, both sexes, age <5 years**	**Child wasting, both sexes, age <5 years**	**Child overweight, both sexes, age 2–4 years**	**Anaemia, females, age 15–49 years**
Croatia						
Observed prevalence ARC, mean (95% UI)	0·22% (-0·85, 1·29)*	0·74% (-1·16, 2·65)*	-0·34% (-1·43, 0·79)	-1·08% (-1·80, -0·34)*	1·36% (-0·72, 3·40)*	-0·10% (-1·53, 1·46)
Expected prevalence ARC	-0·16%	0·00%	-3·27%	-2·19%	0·63%	-1·82%
Prevalence ARC difference	0·37%	0·74%	2·93%	1·11%	0·73%	1·72%
Attributable burden ARC	-2·21%	1·21%	-3·54%	-1·26%	1·25%	-0·75%
Czechia						
Observed prevalence ARC, mean (95% UI)	-0·06% (-0·98, 0·84)	0·17% (-0·79, 1·10)*	0·03% (-1·11, 1·14)*	-0·68% (-1·49, 0·04)*	1·02% (-1·23, 3·16)*	0·26% (-1·25, 2·01)*
Expected prevalence ARC	-0·08%	0·00%	-1·40%	-0·80%	0·28%	-0·70%
Prevalence ARC difference	0·02%	0·17%	1·43%	0·12%	0·74%	0·96%
Attributable burden ARC	-2·61%	2·99%	-2·33%	-0·13%	0·49%	-0·28%
Hungary						
Observed prevalence ARC, mean (95% UI)	-0·04% (-1·00, 0·87)	0·56% (-1·47, 2·70)*	-0·64% (-1·77, 0·56)*	-1·48% (-2·15, -0·80)*	1·21% (-1·03, 3·53)*	-0·10% (-1·66, 1·56)
Expected prevalence ARC	-0·10%	0·00%	-2·10%	-1·42%	0·41%	-1·18%
Prevalence ARC difference	0·06%	0·56%	1·46%	-0·06%	0·80%	1·08%
Attributable burden ARC	-5·94%	2·77%	-3·70%	-0·37%	0·93%	-0·63%
Montenegro						
Observed prevalence ARC, mean (95% UI)	0·00% (-1·09, 1·08)	1·28% (-1·02, 3·59)*	0·06% (-0·98, 1·09)*	-0·71% (-1·34, -0·09)*	1·43% (-0·44, 3·19)*	0·02% (-1·44, 1·63)*
Expected prevalence ARC	-0·20%	0·00%	-4·22%	-2·94%	0·82%	-2·41%
Prevalence ARC difference	0·20%	1·28%	4·28%	2·22%	0·61%	2·43%
Attributable burden ARC	-7·96%	-11·38%	-16·10%	-12·74%	1·13%	-0·59%
North Macedonia						
Observed prevalence ARC, mean (95% UI)	0·03% (-0·99, 1·08)*	0·78% (-1·42, 2·82)*	-1·10% (-2·29, -0·02)*	-1·55% (-2·17, -0·89)*	1·05% (-0·81, 2·85)*	-0·02% (-1·00, 1·01)
Expected prevalence ARC	-0·16%	0·00%	-3·28%	-2·94%	0·74%	-2·24%
Prevalence ARC difference	0·19%	0·78%	2·19%	1·40%	0·31%	2·23%
Attributable burden ARC	-7·80%	-5·66%	-11·41%	-9·29%	0·66%	-0·07%
Poland						
Observed prevalence ARC, mean (95% UI)	0·08% (-0·27, 0·44)*	0·29% (-0·54, 1·16)*	-0·55% (-1·65, 0·57)*	-1·53% (-2·27, -0·80)*	1·40% (-0·82, 3·75)*	0·18% (-1·55, 1·93)*
Expected prevalence ARC	-0·20%	0·00%	-3·86%	-2·47%	0·76%	-2·08%
Prevalence ARC difference	0·28%	0·29%	3·30%	0·94%	0·65%	2·26%
Attributable burden ARC	-3·92%	3·85%	-4·07%	-0·42%	5·09%	-0·41%
Romania						
Observed prevalence ARC, mean (95% UI)	-0·11% (-1·00, 0·71)	0·77% (-1·20, 2·82)*	-1·10% (-2·09, -0·04)*	-1·20% (-2·02, -0·41)*	1·42% (-1·03, 3·74)*	-0·03% (-1·61, 1·69)
Expected prevalence ARC	-0·16%	0·00%	-3·17%	-2·56%	0·71%	-2·01%
Prevalence ARC difference	0·04%	0·77%	2·07%	1·35%	0·71%	1·98%
Attributable burden ARC	-4·56%	-7·56%	-10·41%	-8·48%	0·78%	-0·78%
Serbia						
Observed prevalence ARC, mean (95% UI)	0·20% (-0·78, 1·28)*	0·96% (-1·12, 3·08)*	0·85% (-0·21, 1·85)*	-1·55% (-2·21, -0·88)*	1·06% (-1·09, 2·99)*	-0·05% (-1·43, 1·49)
Expected prevalence ARC	-0·18%	0·00%	-3·61%	-2·53%	0·72%	-2·08%
Prevalence ARC difference	0·38%	0·96%	4·46%	0·98%	0·34%	2·03%
Attributable burden ARC	-3·16%	-2·72%	-4·80%	-3·51%	0·39%	-0·70%

ARC= annualised rate of change. *denotes substantial increase or decrease, based upon consistent direction of mean and >80% of model posteriors.
Prevalence ARC difference= Observed mean ARC - Expected ARC.
Low birthweight prevalence ARC is at birth. Low birthweight attributable burden ARC is in neonatal age group (0 – 27 days).
ARC in attributable burden rate [disability adjusted life-years per 100,000], except for anaemia.
ARC in burden rate for anaemia [years lived with disability per 100,000]

Table S4. Annualised rates of change (ARC) 2012 to 2021: observed prevalence, expected prevalence, difference, attributable burden

	**Low birthweight both sexes, birth**	**Exclusive breastfeeding, both sexes, age <6 months**	**Child stunting, both sexes, age <5 years**	**Child wasting, both sexes, age <5 years**	**Child overweight, both sexes, age 2–4 years**	**Anaemia, females, age 15–49 years**
Slovakia						
Observed prevalence ARC, mean (95% UI)	0·07% (-0·86, 1·05)*	0·14% (-0·35, 0·63)*	-0·41% (-1·42, 0·65)	-1·29% (-1·98, -0·57)*	1·02% (-1·40, 3·31)*	0·16% (-1·41, 1·72)*
Expected prevalence ARC	-0·08%	0·00%	-1·32%	-0·81%	0·31%	-0·69%
Prevalence ARC difference	0·15%	0·14%	0·91%	-0·48%	0·72%	0·85%
Attributable burden ARC	-2·62%	-4·29%	-6·89%	-5·44%	0·76%	-0·36%
Slovenia						
Observed prevalence ARC, mean (95% UI)	-0·03% (-1·09, 0·99)	0·60% (-0·85, 2·00)*	-0·28% (-1·37, 0·78)	-1·13% (-1·88, -0·36)*	1·19% (-0·93, 3·35)*	-0·08% (-1·53, 1·40)
Expected prevalence ARC	-0·11%	0·00%	-1·70%	-0·93%	0·36%	-0·83%
Prevalence ARC difference	0·09%	0·60%	1·43%	-0·19%	0·83%	0·74%
Attributable burden ARC	-5·31%	-0·68%	-6·13%	-4·46%	1·03%	-0·64%
Eastern Europe						
Observed prevalence ARC, mean (95% UI)	0·02% (-0·18, 0·21)*	0·79% (-0·52, 2·18)*	-0·89% (-1·62, -0·16)*	-1·61% (-2·12, -1·11)*	1·17% (-0·46, 2·84)*	-0·36% (-1·50, 0·71)
Expected prevalence ARC	-0·23%	0·00%	-4·20%	-3·27%	0·88%	-2·42%
Prevalence ARC difference	0·25%	0·79%	3·31%	1·66%	0·29%	2·06%
Attributable burden ARC	-7·72%	-6·47%	-11·69%	-8·82%	0·83%	-0·44%
Belarus						
Observed prevalence ARC, mean (95% UI)	0·01% (-1·24, 1·37)*	0·63% (-1·70, 2·99)*	-1·40% (-2·56, -0·24)*	-1·93% (-2·69, -1·15)*	1·92% (-0·34, 4·12)*	-0·52% (-1·85, 0·83)*
Expected prevalence ARC	-0·20%	0·00%	-4·10%	-3·05%	0·84%	-2·46%
Prevalence ARC difference	0·21%	0·63%	2·70%	1·12%	1·08%	1·94%
Attributable burden ARC	-7·33%	-2·97%	-8·81%	-5·34%	2·16%	-0·65%
Estonia						
Observed prevalence ARC, mean (95% UI)	0·14% (-1·01, 1·38)*	0·67% (-0·96, 2·34)*	-0·49% (-1·72, 0·87)	-0·64% (-1·42, 0·20)*	1·26% (-1·04, 3·66)*	-0·25% (-1·47, 1·04)
Expected prevalence ARC	-0·21%	0·00%	-3·30%	-1·84%	0·67%	-1·62%
Prevalence ARC difference	0·35%	0·67%	2·81%	1·20%	0·59%	1·37%
Attributable burden ARC	-7·36%	-8·97%	-14·29%	-9·73%	0·77%	-0·52%
Latvia						
Observed prevalence ARC, mean (95% UI)	0·01% (-1·17, 1·19)*	0·58% (-0·82, 2·02)*	-0·97% (-2·16, 0·30)*	-1·43% (-2·26, -0·61)*	1·20% (-1·17, 3·66)*	-0·47% (-1·73, 0·82)
Expected prevalence ARC	-0·18%	0·00%	-2·92%	-1·70%	0·60%	-1·49%
Prevalence ARC difference	0·19%	0·58%	1·95%	0·27%	0·60%	1·03%
Attributable burden ARC	-6·55%	-10·17%	-15·47%	-10·85%	0·73%	-0·77%
Lithuania						
Observed prevalence ARC, mean (95% UI)	-0·09% (-1·25, 1·12)	0·61% (-1·38, 2·68)*	-0·93% (-2·17, 0·31)*	-1·42% (-2·23, -0·59)*	1·57% (-0·97, 4·10)*	-0·56% (-1·89, 0·71)*
Expected prevalence ARC	-0·30%	0·00%	-4·55%	-2·51%	0·96%	-2·22%
Prevalence ARC difference	0·22%	0·61%	3·62%	1·08%	0·61%	1·66%
Attributable burden ARC	-4·60%	-6·60%	-13·24%	-9·29%	1·26%	-1·00%
Moldova						
Observed prevalence ARC, mean (95% UI)	-0·09% (-1·13, 0·93)	0·56% (-1·20, 2·34)*	-1·87% (-2·99, -0·61)*	-2·37% (-3·13, -1·61)*	2·21% (-0·44, 4·84)*	-0·51% (-1·57, 0·55)*
Expected prevalence ARC	-0·26%	0·00%	-4·16%	-4·23%	1·04%	-3·02%
Prevalence ARC difference	0·17%	0·56%	2·30%	1·86%	1·17%	2·51%
Attributable burden ARC	-5·50%	-3·07%	-8·18%	-6·15%	1·75%	-0·69%

ARC= annualised rate of change. *denotes substantial increase or decrease, based upon consistent direction of mean and >80% of model posteriors.
Prevalence ARC difference= Observed mean ARC - Expected ARC.
Low birthweight prevalence ARC is at birth. Low birthweight attributable burden ARC is in neonatal age group (0 – 27 days).
ARC in attributable burden rate [disability adjusted life-years per 100,000], except for anaemia.
ARC in burden rate for anaemia [years lived with disability per 100,000]

Table S4. Annualised rates of change (ARC) 2012 to 2021: observed prevalence, expected prevalence, difference, attributable burden

	**Low birthweight both sexes, birth**	**Exclusive breastfeeding, both sexes, age <6 months**	**Child stunting, both sexes, age <5 years**	**Child wasting, both sexes, age <5 years**	**Child overweight, both sexes, age 2–4 years**	**Anaemia, females, age 15–49 years**
Russia						
Observed prevalence ARC, mean (95% UI)	-0·09% (-0·25, 0·07)*	0·63% (-0·98, 2·37)*	-0·86% (-1·87, 0·17)*	-1·57% (-2·37, -0·71)*	0·83% (-1·17, 2·86)*	-0·52% (-2·05, 0·95)
Expected prevalence ARC	-0·19%	0·00%	-3·84%	-2·49%	0·74%	-2·09%
Prevalence ARC difference	0·11%	0·63%	2·98%	0·92%	0·08%	1·57%
Attributable burden ARC	-8·55%	-7·76%	-13·39%	-11·60%	0·60%	-0·63%
Ukraine						
Observed prevalence ARC, mean (95% UI)	-0·10% (-1·26, 1·00)	0·72% (-1·66, 2·86)*	-0·20% (-0·91, 0·56)	-0·47% (-1·09, 0·20)*	1·62% (-0·85, 4·13)*	0·27% (-0·72, 1·33)*
Expected prevalence ARC	-0·14%	0·00%	-3·09%	-2·58%	0·66%	-2·01%
Prevalence ARC difference	0·04%	0·72%	2·89%	2·11%	0·96%	2·28%
Attributable burden ARC	-4·45%	-0·40%	-3·54%	-2·89%	0·92%	0·21%
**High income**						
**Observed prevalence ARC, mean (95% UI)**	**-0·01% (-0·16, 0·13)**	**0·30% (0·00, 0·61)***	**-0·99% (-1·44, -0·50)***	**-0·83% (-1·02, -0·64)***	**1·47% (0·71, 2·24)***	**0·06% (-1·07, 1·25)***
**Expected prevalence ARC**	**-0·21%**	**0·00%**	**-3·27%**	**-2·19%**	**0·61%**	**-1·46%**
**Prevalence ARC difference**	**0·20%**	**0·30%**	**2·28%**	**1·36%**	**0·87%**	**1·52%**
**Attributable burden ARC**	**-2·35%**	**-6·51%**	**-9·68%**	**-6·98%**	**1·17%**	**0·06%**
Australasia						
Observed prevalence ARC, mean (95% UI)	-0·16% (-1·14, 0·80)	0·40% (-0·30, 1·03)*	-0·70% (-1·81, 0·42)*	-0·87% (-1·66, -0·04)*	1·47% (-0·31, 3·17)*	-0·19% (-1·39, 1·02)
Expected prevalence ARC	-0·20%	0·00%	-3·10%	-1·73%	0·65%	-1·53%
Prevalence ARC difference	0·04%	0·40%	2·40%	0·86%	0·82%	1·34%
Attributable burden ARC	-2·87%	-8·77%	-12·00%	-9·53%	0·75%	-0·17%
Australia						
Observed prevalence ARC, mean (95% UI)	-0·17% (-1·26, 0·99)	0·46% (-0·37, 1·25)*	-0·57% (-1·99, 0·80)	-0·84% (-1·75, 0·13)*	1·63% (-0·46, 3·66)*	-0·11% (-1·52, 1·32)
Expected prevalence ARC	-0·20%	0·00%	-3·10%	-1·73%	0·65%	-1·53%
Prevalence ARC difference	0·03%	0·46%	2·53%	0·89%	0·98%	1·41%
Attributable burden ARC	-3·12%	-8·91%	-11·92%	-9·25%	0·79%	-0·05%
New Zealand						
Observed prevalence ARC, mean (95% UI)	-0·21% (-1·10, 0·73)	0·06% (-0·74, 0·80)*	-0·90% (-2·02, 0·28)*	-0·99% (-1·91, -0·06)*	0·96% (-1·03, 2·87)*	-0·55% (-1·81, 0·76)
Expected prevalence ARC	-0·21%	0·00%	-2·81%	-1·53%	0·68%	-1·36%
Prevalence ARC difference	0·01%	0·06%	1·91%	0·54%	0·27%	0·81%
Attributable burden ARC	-2·19%	-8·78%	-12·17%	-10·13%	0·59%	-0·71%
High-income Asia Pacific						
Observed prevalence ARC, mean (95% UI)	-0·11% (-0·45, 0·21)	0·45% (-0·35, 1·30)*	-0·98% (-1·23, -0·71)*	-1·00% (-1·20, -0·81)*	2·21% (0·35, 4·00)*	0·06% (-2·15, 2·44)*
Expected prevalence ARC	-0·22%	0·00%	-2·30%	-1·15%	0·62%	-1·10%
Prevalence ARC difference	0·12%	0·45%	1·32%	0·15%	1·59%	1·16%
Attributable burden ARC	-5·45%	-7·02%	-10·53%	-8·30%	2·40%	0·19%
Brunei						
Observed prevalence ARC, mean (95% UI)	-0·16% (-0·94, 0·60)	0·54% (-0·30, 1·38)*	-0·47% (-1·21, 0·25)*	-0·89% (-1·64, -0·11)*	2·86% (0·12, 5·62)*	0·28% (-1·99, 3·40)*
Expected prevalence ARC	-0·16%	0·00%	-3·08%	-1·95%	0·61%	-1·66%
Prevalence ARC difference	0·00%	0·54%	2·61%	1·06%	2·25%	1·93%
Attributable burden ARC	-0·22%	-5·37%	-7·12%	-5·93%	1·59%	0·43%

ARC= annualised rate of change. *denotes substantial increase or decrease, based upon consistent direction of mean and >80% of model posteriors.
Prevalence ARC difference= Observed mean ARC - Expected ARC.
Low birthweight prevalence ARC is at birth. Low birthweight attributable burden ARC is in neonatal age group (0 – 27 days).
ARC in attributable burden rate [disability adjusted life-years per 100,000], except for anaemia.
ARC in burden rate for anaemia [years lived with disability per 100,000]

Table S4. Annualised rates of change (ARC) 2012 to 2021: observed prevalence, expected prevalence, difference, attributable burden

	Low birthweight both sexes, birth	Exclusive breastfeeding, both sexes, age <6 months	Child stunting, both sexes, age <5 years	Child wasting, both sexes, age <5 years	Child overweight, both sexes, age 2–4 years	Anaemia, females, age 15–49 years
Japan						
Observed prevalence ARC, mean (95% UI)	-0·12% (-0·38, 0·12)*	0·39% (-0·70, 1·53)*	-0·46% (-0·71, -0·22)*	-0·81% (-0·98, -0·66)*	1·96% (-0·76, 4·52)*	0·19% (-2·64, 3·32)*
Expected prevalence ARC	-0·15%	0·00%	-1·74%	-0·88%	0·43%	-0·80%
Prevalence ARC difference	0·03%	0·39%	1·27%	0·07%	1·53%	0·99%
Attributable burden ARC	-4·86%	-8·29%	-10·90%	-8·82%	2·95%	0·56%
Singapore						
Observed prevalence ARC, mean (95% UI)	-0·14% (-1·05, 0·74)	0·75% (-0·62, 2·14)*	-0·63% (-1·88, 0·59)*	-0·35% (-1·34, 0·61)	3·22% (0·56, 6·10)*	-0·56% (-9·17, 7·66)
Expected prevalence ARC	-0·12%	0·00%	-1·57%	-0·82%	0·40%	-0·77%
Prevalence ARC difference	-0·02%	0·75%	0·94%	0·48%	2·82%	0·21%
Attributable burden ARC	-2·84%	-8·85%	-14·59%	-13·00%	2·68%	-0·43%
South Korea						
Observed prevalence ARC, mean (95% UI)	-0·01% (-1·17, 1·03)	0·39% (-0·65, 1·52)*	-3·37% (-4·53, -2·13)*	-1·43% (-2·25, -0·61)*	2·57% (0·01, 5·01)*	-0·35% (-1·89, 1·13)
Expected prevalence ARC	-0·26%	0·00%	-2·56%	-1·26%	0·68%	-1·15%
Prevalence ARC difference	0·25%	0·39%	-0·81%	-0·17%	1·89%	0·80%
Attributable burden ARC	-5·56%	-3·52%	-11·67%	-6·69%	1·38%	-0·65%
High-income North America						
Observed prevalence ARC, mean (95% UI)	0·23% (0·04, 0·42)*	0·23% (-0·37, 0·82)*	-1·21% (-2·44, 0·10)*	-0·39% (-0·94, 0·18)*	1·15% (-1·02, 3·08)*	-0·02% (-1·71, 1·71)
Expected prevalence ARC	-0·18%	0·00%	-2·56%	-1·36%	0·55%	-1·16%
Prevalence ARC difference	0·42%	0·23%	1·35%	0·96%	0·60%	1·14%
Attributable burden ARC	-2·36%	-5·62%	-6·65%	-2·87%	0·57%	-0·15%
Canada						
Observed prevalence ARC, mean (95% UI)	0·00% (-1·16, 1·18)*	0·36% (-1·22, 1·82)*	-1·20% (-2·49, -0·03)*	0·10% (-0·91, 1·10)*	0·82% (-1·24, 2·92)*	0·00% (-6·80, 7·33)*
Expected prevalence ARC	-0·16%	0·00%	-1·69%	-0·85%	0·43%	-0·78%
Prevalence ARC difference	0·16%	0·36%	0·50%	0·95%	0·39%	0·78%
Attributable burden ARC	-2·78%	-7·56%	-10·76%	-7·20%	-0·12%	0·23%
Greenland						
Observed prevalence ARC, mean (95% UI)	-0·14% (-1·40, 1·08)	0·23% (-0·37, 0·87)*	-1·71% (-3·09, -0·44)*	-0·98% (-1·76, -0·22)*	0·90% (-1·40, 3·15)*	-0·74% (-2·75, 1·08)
Expected prevalence ARC	-0·09%	0·00%	-2·35%	-1·33%	0·28%	-1·15%
Prevalence ARC difference	-0·05%	0·23%	0·63%	0·35%	0·63%	0·41%
Attributable burden ARC	-3·46%	-6·65%	-10·13%	-8·04%	-0·17%	-0·92%
USA						
Observed prevalence ARC, mean (95% UI)	0·26% (0·07, 0·44)*	0·22% (-0·39, 0·82)*	-1·17% (-2·43, 0·19)*	-0·69% (-1·34, -0·05)*	1·16% (-1·34, 3·39)*	-0·03% (-1·52, 1·74)
Expected prevalence ARC	-0·19%	0·00%	-2·56%	-1·35%	0·56%	-1·19%
Prevalence ARC difference	0·44%	0·22%	1·40%	0·66%	0·60%	1·16%
Attributable burden ARC	-2·32%	-5·34%	-6·43%	-2·35%	0·63%	-0·17%
Southern Latin America						
Observed prevalence ARC, mean (95% UI)	0·13% (-0·50, 0·78)*	0·49% (-0·33, 1·19)*	-0·77% (-1·76, 0·25)*	-1·37% (-2·20, -0·56)*	1·60% (0·02, 3·10)*	-0·25% (-7·22, 6·51)
Expected prevalence ARC	-0·26%	0·00%	-4·73%	-4·65%	1·01%	-3·32%
Prevalence ARC difference	0·39%	0·49%	3·96%	3·28%	0·60%	3·07%
Attributable burden ARC	-2·89%	-9·04%	-11·29%	-9·99%	1·31%	-0·15%

ARC= annualised rate of change. *denotes substantial increase or decrease, based upon consistent direction of mean and >80% of model posteriors.
Prevalence ARC difference= Observed mean ARC - Expected ARC.
Low birthweight prevalence ARC is at birth. Low birthweight attributable burden ARC is in neonatal age group (0 – 27 days).
ARC in attributable burden rate [disability adjusted life-years per 100,000], except for anaemia.
ARC in burden rate for anaemia [years lived with disability per 100,000]

Table S4. Annualised rates of change (ARC) 2012 to 2021: observed prevalence, expected prevalence, difference, attributable burden

	Low birthweight both sexes, birth	Exclusive breastfeeding, both sexes, age <6 months	Child stunting, both sexes, age <5 years	Child wasting, both sexes, age <5 years	Child overweight, both sexes, age 2–4 years	Anaemia, females, age 15–49 years
Argentina						
Observed prevalence ARC, mean (95% UI)	0·13% (-0·67, 0·96)*	0·57% (-0·68, 1·62)*	-0·74% (-1·89, 0·46)*	-1·11% (-2·10, -0·14)*	1·65% (-0·55, 3·94)*	-0·22% (-9·41, 8·57)
Expected prevalence ARC	-0·25%	0·00%	-4·57%	-4·67%	0·93%	-3·31%
Prevalence ARC difference	0·38%	0·57%	3·83%	3·55%	0·71%	3·10%
Attributable burden ARC	-2·56%	-9·01%	-11·16%	-10·11%	1·31%	0·15%
Chile						
Observed prevalence ARC, mean (95% UI)	0·06% (-1·01, 1·15)*	0·40% (-0·17, 0·97)*	-1·32% (-2·59, -0·04)*	-3·53% (-4·39, -2·64)*	1·61% (-0·23, 3·42)*	-0·96% (-2·61, 0·64)*
Expected prevalence ARC	-0·25%	0·00%	-5·27%	-4·45%	1·18%	-3·45%
Prevalence ARC difference	0·31%	0·40%	3·95%	0·93%	0·43%	2·49%
Attributable burden ARC	-3·33%	-8·08%	-11·83%	-9·20%	1·33%	-1·08%
Uruguay						
Observed prevalence ARC, mean (95% UI)	-0·17% (-1·14, 0·73)	0·32% (-0·35, 0·94)*	-0·94% (-1·96, 0·09)*	-1·44% (-2·27, -0·56)*	1·41% (-0·88, 3·64)*	-0·81% (-8·09, 6·09)
Expected prevalence ARC	-0·34%	0·00%	-4·47%	-4·77%	1·14%	-3·27%
Prevalence ARC difference	0·17%	0·32%	3·53%	3·33%	0·28%	2·46%
Attributable burden ARC	-4·99%	-7·69%	-10·73%	-10·05%	0·80%	-0·79%
Western Europe						
Observed prevalence ARC, mean (95% UI)	-0·10% (-0·40, 0·17)	0·27% (-0·09, 0·63)*	-0·45% (-0·89, 0·01)*	-0·73% (-0·99, -0·47)*	1·41% (0·77, 2·07)*	0·12% (-1·74, 2·28)*
Expected prevalence ARC	-0·19%	0·00%	-2·75%	-1·66%	0·60%	-1·18%
Prevalence ARC difference	0·09%	0·27%	2·30%	0·93%	0·81%	1·30%
Attributable burden ARC	-1·61%	-3·99%	-7·48%	-4·72%	1·75%	0·06%
Andorra						
Observed prevalence ARC, mean (95% UI)	0·00% (-1·12, 1·05)*	0·25% (-0·44, 0·93)*	0·14% (-1·10, 1·30)*	0·38% (-0·36, 1·13)*	1·44% (-0·71, 3·50)*	-0·15% (-1·81, 1·50)
Expected prevalence ARC	-0·16%	0·00%	-1·86%	-0·94%	0·49%	-0·88%
Prevalence ARC difference	0·17%	0·25%	2·00%	1·32%	0·96%	0·73%
Attributable burden ARC	-10·22%	-9·91%	-11·96%	-10·46%	0·99%	-0·01%
Austria						
Observed prevalence ARC, mean (95% UI)	-0·07% (-1·05, 0·98)	0·32% (-1·25, 1·80)*	-0·35% (-1·42, 0·74)	-0·52% (-1·20, 0·20)*	1·09% (-1·17, 3·51)*	-0·43% (-2·18, 1·27)
Expected prevalence ARC	-0·15%	0·00%	-2·07%	-1·10%	0·45%	-0·99%
Prevalence ARC difference	0·07%	0·32%	1·72%	0·58%	0·64%	0·55%
Attributable burden ARC	-1·61%	-5·92%	-8·49%	-8·12%	0·58%	-0·47%
Belgium						
Observed prevalence ARC, mean (95% UI)	-0·02% (-0·95, 0·89)	0·36% (-0·83, 1·51)*	-0·47% (-1·62, 0·75)	-0·70% (-1·46, -0·01)*	1·66% (-0·85, 4·13)*	-0·38% (-1·67, 1·04)
Expected prevalence ARC	-0·19%	0·00%	-2·66%	-1·43%	0·58%	-1·28%
Prevalence ARC difference	0·16%	0·36%	2·19%	0·73%	1·08%	0·89%
Attributable burden ARC	-0·84%	-2·03%	-6·52%	-4·43%	1·00%	-0·47%
Cyprus						
Observed prevalence ARC, mean (95% UI)	-0·15% (-0·95, 0·61)	0·32% (-0·47, 1·06)*	-0·44% (-1·53, 0·70)	-0·61% (-1·31, 0·04)*	1·87% (-0·37, 4·11)*	-0·19% (-1·90, 1·43)
Expected prevalence ARC	-0·11%	0·00%	-1·79%	-1·01%	0·37%	-0·89%
Prevalence ARC difference	-0·04%	0·32%	1·35%	0·39%	1·50%	0·70%
Attributable burden ARC	-4·02%	-5·00%	-7·44%	-5·51%	2·12%	0·05%

ARC= annualised rate of change. *denotes substantial increase or decrease, based upon consistent direction of mean and >80% of model posteriors.
Prevalence ARC difference= Observed mean ARC - Expected ARC.
Low birthweight prevalence ARC is at birth. Low birthweight attributable burden ARC is in neonatal age group (0 – 27 days).
ARC in attributable burden rate [disability adjusted life-years per 100,000], except for anaemia.
ARC in burden rate for anaemia [years lived with disability per 100,000]

Table S4. Annualised rates of change (ARC) 2012 to 2021: observed prevalence, expected prevalence, difference, attributable burden

	Low birthweight both sexes, birth	Exclusive breastfeeding, both sexes, age <6 months	Child stunting, both sexes, age <5 years	Child wasting, both sexes, age <5 years	Child overweight, both sexes, age 2–4 years	Anaemia, females, age 15–49 years
Denmark						
Observed prevalence ARC, mean (95% UI)	-0·07% (-1·19, 1·13)	0·15% (-0·53, 0·79)*	-0·26% (-1·34, 0·84)	-0·43% (-1·18, 0·39)*	1·09% (-1·09, 3·37)*	-0·44% (-2·71, 2·69)
Expected prevalence ARC	-0·19%	0·00%	-1·41%	-0·67%	0·46%	-0·62%
Prevalence ARC difference	0·11%	0·15%	1·15%	0·24%	0·63%	0·18%
Attributable burden ARC	-1·50%	-2·76%	-4·73%	-2·58%	-0·65%	-0·62%
Finland						
Observed prevalence ARC, mean (95% UI)	-0·10% (-1·25, 1·11)	0·31% (-0·53, 1·13)*	-0·39% (-1·58, 0·74)	-0·24% (-0·89, 0·49)	1·27% (-0·99, 3·48)*	-0·23% (-1·80, 1·20)
Expected prevalence ARC	-0·19%	0·00%	-2·50%	-1·31%	0·56%	-1·18%
Prevalence ARC difference	0·09%	0·31%	2·11%	1·08%	0·71%	0·95%
Attributable burden ARC	-2·01%	-5·21%	-8·08%	-6·30%	0·90%	-0·29%
France						
Observed prevalence ARC, mean (95% UI)	-0·02% (-1·15, 1·09)	0·37% (-1·25, 1·88)*	-0·54% (-1·62, 0·56)*	-0·70% (-1·42, 0·02)*	1·65% (-0·61, 4·08)*	0·30% (-0·99, 1·61)*
Expected prevalence ARC	-0·17%	0·00%	-2·74%	-1·55%	0·57%	-1·36%
Prevalence ARC difference	0·15%	0·37%	2·20%	0·85%	1·08%	1·66%
Attributable burden ARC	0·12%	-3·42%	-4·52%	-2·56%	3·04%	-0·01%
Germany						
Observed prevalence ARC, mean (95% UI)	0·03% (-0·96, 0·99)*	0·10% (-0·98, 1·18)*	-0·46% (-1·51, 0·55)	-0·61% (-1·29, 0·04)*	0·90% (-1·14, 2·99)*	0·47% (-6·12, 7·64)*
Expected prevalence ARC	-0·15%	0·00%	-0·96%	-0·45%	0·37%	-0·42%
Prevalence ARC difference	0·19%	0·10%	0·50%	-0·16%	0·54%	0·88%
Attributable burden ARC	-1·26%	-4·95%	-9·28%	-6·59%	3·82%	0·59%
Greece						
Observed prevalence ARC, mean (95% UI)	0·04% (-0·84, 0·92)*	0·26% (-0·73, 1·27)*	0·12% (-1·24, 1·50)*	0·09% (-0·84, 0·99)*	1·82% (-0·22, 4·01)*	0·24% (-1·56, 2·24)*
Expected prevalence ARC	-0·06%	0·00%	-1·15%	-0·76%	0·23%	-0·64%
Prevalence ARC difference	0·10%	0·26%	1·27%	0·85%	1·60%	0·88%
Attributable burden ARC	1·17%	-4·19%	-5·75%	-4·77%	2·18%	0·51%
Iceland						
Observed prevalence ARC, mean (95% UI)	0·01% (-1·18, 1·15)*	0·15% (-0·68, 0·94)*	-0·40% (-1·50, 0·72)	-0·30% (-1·05, 0·42)	0·74% (-1·44, 2·81)*	-0·37% (-1·81, 1·13)
Expected prevalence ARC	-0·21%	0·00%	-2·12%	-1·07%	0·56%	-0·97%
Prevalence ARC difference	0·22%	0·15%	1·72%	0·77%	0·18%	0·61%
Attributable burden ARC	-3·07%	-4·66%	-4·22%	-3·77%	0·36%	-0·34%
Ireland						
Observed prevalence ARC, mean (95% UI)	1·84% (0·67, 3·04)*	0·49% (-1·11, 2·12)*	-0·86% (-1·93, 0·26)*	-1·05% (-1·76, -0·27)*	1·65% (-0·37, 3·74)*	-0·50% (-2·32, 1·37)
Expected prevalence ARC	-0·26%	0·00%	-3·13%	-1·61%	0·75%	-1·46%
Prevalence ARC difference	2·10%	0·49%	2·27%	0·55%	0·90%	0·96%
Attributable burden ARC	-1·95%	-5·48%	-9·50%	-8·14%	0·72%	-0·28%
Israel						
Observed prevalence ARC, mean (95% UI)	-0·06% (-1·18, 1·05)	0·28% (-0·46, 1·03)*	-0·53% (-1·59, 0·51)*	-0·78% (-1·50, -0·09)*	1·20% (-0·97, 3·40)*	-0·38% (-2·89, 1·56)
Expected prevalence ARC	-0·15%	0·00%	-2·95%	-1·87%	0·58%	-1·59%
Prevalence ARC difference	0·09%	0·28%	2·43%	1·10%	0·62%	1·20%
Attributable burden ARC	-8·51%	-6·40%	-11·45%	-8·71%	0·45%	-0·17%

ARC= annualised rate of change. *denotes substantial increase or decrease, based upon consistent direction of mean and >80% of model posteriors.
Prevalence ARC difference= Observed mean ARC - Expected ARC.
Low birthweight prevalence ARC is at birth. Low birthweight attributable burden ARC is in neonatal age group (0 – 27 days).
ARC in attributable burden rate [disability adjusted life-years per 100,000], except for anaemia.
ARC in burden rate for anaemia [years lived with disability per 100,000]

Table S4. Annualised rates of change (ARC) 2012 to 2021: observed prevalence, expected prevalence, difference, attributable burden

	Low birthweight both sexes, birth	Exclusive breastfeeding, both sexes, age <6 months	Child stunting, both sexes, age <5 years	Child wasting, both sexes, age <5 years	Child overweight, both sexes, age 2–4 years	Anaemia, females, age 15–49 years
Italy						
Observed prevalence ARC, mean (95% UI)	-0·27% (-0·62, 0·08)*	0·23% (-0·63, 1·09)*	-0·32% (-1·39, 0·80)	-0·46% (-1·19, 0·21)*	1·76% (-0·56, 3·98)*	-0·24% (-3·08, 2·49)
Expected prevalence ARC	-0·12%	0·00%	-2·32%	-1·48%	0·46%	-1·25%
Prevalence ARC difference	-0·14%	0·23%	2·00%	1·01%	1·30%	1·00%
Attributable burden ARC	-3·88%	0·21%	-6·00%	-1·82%	2·48%	-0·15%
Luxembourg						
Observed prevalence ARC, mean (95% UI)	0·01% (-1·10, 1·10)*	0·30% (-0·51, 1·11)*	-0·63% (-1·80, 0·54)*	-0·74% (-1·41, -0·05)*	1·33% (-0·92, 3·52)*	-0·45% (-2·21, 1·20)
Expected prevalence ARC	-0·18%	0·00%	-1·70%	-0·83%	0·48%	-0·76%
Prevalence ARC difference	0·19%	0·30%	1·07%	0·09%	0·86%	0·32%
Attributable burden ARC	2·66%	-0·40%	-2·72%	0·08%	0·77%	-0·31%
Malta						
Observed prevalence ARC, mean (95% UI)	-0·48% (-1·51, 0·51)*	0·40% (-0·36, 1·20)*	-0·97% (-2·02, 0·15)*	-1·19% (-1·89, -0·52)*	2·09% (-0·32, 4·48)*	-0·52% (-2·28, 1·39)
Expected prevalence ARC	-0·19%	0·00%	-3·86%	-2·59%	0·75%	-2·15%
Prevalence ARC difference	-0·29%	0·40%	2·89%	1·40%	1·34%	1·63%
Attributable burden ARC	-2·87%	-5·43%	-8·31%	-7·04%	1·68%	-0·33%
Monaco						
Observed prevalence ARC, mean (95% UI)	-0·02% (-1·10, 1·10)	0·23% (-0·44, 1·02)*	-0·67% (-1·76, 0·48)*	-0·72% (-1·46, 0·02)*	0·96% (-0·95, 2·97)*	-0·48% (-2·12, 1·16)
Expected prevalence ARC	-0·13%	0·00%	-0·61%	-0·28%	0·27%	-0·25%
Prevalence ARC difference	0·11%	0·23%	-0·06%	-0·44%	0·69%	-0·23%
Attributable burden ARC	-1·87%	-1·53%	1·94%	3·02%	0·47%	-0·48%
Netherlands						
Observed prevalence ARC, mean (95% UI)	-0·44% (-1·57, 0·76)	0·59% (-0·53, 1·66)*	-0·03% (-1·14, 1·07)	-0·09% (-0·88, 0·61)	1·45% (-0·93, 3·86)*	-0·45% (-2·05, 1·23)
Expected prevalence ARC	-0·16%	0·00%	-1·37%	-0·66%	0·41%	-0·61%
Prevalence ARC difference	-0·28%	0·59%	1·34%	0·56%	1·04%	0·16%
Attributable burden ARC	-0·90%	-3·63%	-5·61%	-2·91%	2·17%	-0·44%
Norway						
Observed prevalence ARC, mean (95% UI)	-0·33% (-0·76, 0·08)*	0·23% (-0·29, 0·72)*	-0·52% (-1·65, 0·52)*	-0·55% (-1·24, 0·20)*	1·02% (-1·07, 3·20)*	-0·24% (-2·13, 2·02)
Expected prevalence ARC	-0·23%	0·00%	-1·11%	-0·50%	0·53%	-0·47%
Prevalence ARC difference	-0·10%	0·23%	0·59%	-0·04%	0·49%	0·24%
Attributable burden ARC	-3·90%	-1·14%	-6·90%	-5·85%	-0·17%	-0·33%
Portugal						
Observed prevalence ARC, mean (95% UI)	1·21% (0·29, 2·15)*	0·32% (-0·70, 1·35)*	-0·53% (-1·65, 0·59)*	-0·72% (-1·41, -0·02)*	2·26% (0·08, 4·54)*	-0·48% (-2·48, 1·52)
Expected prevalence ARC	-0·12%	0·00%	-2·70%	-2·46%	0·59%	-1·86%
Prevalence ARC difference	1·33%	0·32%	2·17%	1·73%	1·67%	1·38%
Attributable burden ARC	-4·28%	-3·22%	-5·92%	-4·17%	2·77%	-0·01%
San Marino						
Observed prevalence ARC, mean (95% UI)	-0·01% (-1·22, 1·15)	0·22% (-0·48, 0·98)*	0·15% (-0·90, 1·25)*	0·12% (-0·61, 0·88)*	1·16% (-0·87, 3·12)*	-0·04% (-1·82, 1·67)
Expected prevalence ARC	-0·04%	0·00%	-0·28%	-0·13%	0·10%	-0·12%
Prevalence ARC difference	0·03%	0·22%	0·42%	0·25%	1·06%	0·09%
Attributable burden ARC	-7·06%	-7·32%	-9·09%	-7·66%	0·76%	0·20%

ARC= annualised rate of change. *denotes substantial increase or decrease, based upon consistent direction of mean and >80% of model posteriors.
Prevalence ARC difference= Observed mean ARC - Expected ARC.
Low birthweight prevalence ARC is at birth. Low birthweight attributable burden ARC is in neonatal age group (0 – 27 days).
ARC in attributable burden rate [disability adjusted life-years per 100,000], except for anaemia.
ARC in burden rate for anaemia [years lived with disability per 100,000]

Table S4. Annualised rates of change (ARC) 2012 to 2021: observed prevalence, expected prevalence, difference, attributable burden

	Low birthweight both sexes, birth	Exclusive breastfeeding, both sexes, age <6 months	Child stunting, both sexes, age <5 years	Child wasting, both sexes, age <5 years	Child overweight, both sexes, age 2–4 years	Anaemia, females, age 15–49 years
Spain						
Observed prevalence ARC, mean (95% UI)	-0·16% (-0·98, 0·69)	0·33% (-0·57, 1·24)*	-0·33% (-1·49, 0·80)	-0·51% (-1·26, 0·28)*	1·85% (-0·39, 4·02)*	-0·17% (-1·92, 1·64)
Expected prevalence ARC	-0·11%	0·00%	-2·43%	-1·87%	0·49%	-1·49%
Prevalence ARC difference	-0·05%	0·33%	2·10%	1·36%	1·35%	1·32%
Attributable burden ARC	-3·18%	-3·33%	-6·23%	-3·93%	1·00%	0·22%
Sweden						
Observed prevalence ARC, mean (95% UI)	0·08% (-0·85, 1·03)*	0·06% (-0·53, 0·65)*	-0·35% (-1·54, 0·68)	-0·52% (-1·20, 0·19)*	1·06% (-1·15, 3·19)*	-0·35% (-3·53, 2·72)
Expected prevalence ARC	-0·16%	0·00%	-1·48%	-0·71%	0·43%	-0·66%
Prevalence ARC difference	0·25%	0·06%	1·12%	0·19%	0·64%	0·31%
Attributable burden ARC	-3·95%	-3·53%	-7·28%	-5·14%	3·37%	-0·39%
Switzerland						
Observed prevalence ARC, mean (95% UI)	-0·06% (-1·09, 0·94)	0·37% (-0·53, 1·27)*	-0·38% (-1·47, 0·70)	-0·47% (-1·23, 0·20)*	0·71% (-1·47, 3·07)*	0·99% (-6·30, 8·57)*
Expected prevalence ARC	-0·15%	0·00%	-0·22%	-0·09%	0·32%	-0·09%
Prevalence ARC difference	0·09%	0·37%	-0·17%	-0·37%	0·38%	1·07%
Attributable burden ARC	-2·57%	-3·51%	-6·10%	-4·33%	0·22%	1·76%
UK						
Observed prevalence ARC, mean (95% UI)	0·01% (-0·13, 0·14)*	0·23% (-1·54, 1·97)*	-0·55% (-2·17, 1·03)	-0·70% (-1·57, 0·13)*	0·92% (0·31, 1·55)*	0·21% (-1·53, 2·46)*
Expected prevalence ARC	-0·21%	0·00%	-2·83%	-1·51%	0·64%	-1·35%
Prevalence ARC difference	0·22%	0·23%	2·28%	0·80%	0·28%	1·56%
Attributable burden ARC	-0·77%	-5·86%	-10·90%	-8·79%	0·39%	-0·12%
**Latin America and Caribbean**						
**Observed prevalence ARC, mean (95% UI)**	**0·02% (-0·16, 0·20)***	**0·53% (0·12, 0·94)***	**-1·32% (-1·51, -1·14)***	**-1·52% (-1·65, -1·39)***	**2·32% (1·43, 3·24)***	**-0·23% (-0·94, 0·48)**
**Expected prevalence ARC**	**-0·78%**	**0·02%**	**-2·23%**	**-2·27%**	**0·86%**	**-1·25%**
**Prevalence ARC difference**	**0·80%**	**0·51%**	**0·91%**	**0·75%**	**1·46%**	**1·02%**
**Attributable burden ARC**	**-3·50%**	**-7·12%**	**-8·38%**	**-6·63%**	**-0·07%**	**-0·72%**
Andean Latin America						
Observed prevalence ARC, mean (95% UI)	-0·10% (-0·73, 0·53)	0·42% (0·12, 0·69)*	-2·47% (-3·03, -1·92)*	-2·26% (-2·65, -1·87)*	1·96% (0·81, 3·06)*	1·04% (-0·14, 2·40)*
Expected prevalence ARC	-0·88%	0·00%	-3·49%	-3·77%	1·06%	-1·93%
Prevalence ARC difference	0·78%	0·42%	1·01%	1·51%	0·90%	2·97%
Attributable burden ARC	-4·73%	-8·85%	-10·84%	-8·65%	0·23%	-1·28%
Bolivia						
Observed prevalence ARC, mean (95% UI)	-0·35% (-1·63, 0·81)	0·45% (-0·10, 0·91)*	-2·28% (-3·28, -1·33)*	-2·60% (-3·22, -1·95)*	2·27% (0·55, 4·14)*	-1·80% (-2·73, -0·94)*
Expected prevalence ARC	-1·47%	0·00%	-3·38%	-3·15%	1·12%	-1·13%
Prevalence ARC difference	1·12%	0·45%	1·10%	0·55%	1·15%	-0·67%
Attributable burden ARC	-3·71%	-8·88%	-11·30%	-9·07%	-1·65%	-2·01%
Ecuador						
Observed prevalence ARC, mean (95% UI)	0·16% (-0·78, 1·14)*	0·46% (-0·47, 1·30)*	-1·12% (-2·01, -0·26)*	-1·63% (-2·28, -1·04)*	2·96% (0·80, 5·15)*	-0·47% (-1·72, 0·82)
Expected prevalence ARC	-0·86%	0·00%	-4·25%	-4·80%	1·21%	-2·60%
Prevalence ARC difference	1·02%	0·46%	3·13%	3·17%	1·75%	2·13%
Attributable burden ARC	-4·61%	-9·89%	-11·63%	-8·66%	0·92%	-0·62%

ARC= annualised rate of change. *denotes substantial increase or decrease, based upon consistent direction of mean and >80% of model posteriors.
Prevalence ARC difference= Observed mean ARC - Expected ARC.
Low birthweight prevalence ARC is at birth. Low birthweight attributable burden ARC is in neonatal age group (0 – 27 days).
ARC in attributable burden rate [disability adjusted life-years per 100,000], except for anaemia.
ARC in burden rate for anaemia [years lived with disability per 100,000]

Table S4. Annualised rates of change (ARC) 2012 to 2021: observed prevalence, expected prevalence, difference, attributable burden

	Low birthweight both sexes, birth	Exclusive breastfeeding, both sexes, age <6 months	Child stunting, both sexes, age <5 years	Child wasting, both sexes, age <5 years	Child overweight, both sexes, age 2–4 years	Anaemia, females, age 15–49 years
Peru						
Observed prevalence ARC, mean (95% UI)	-0·16% (-1·19, 0·86)	0·39% (0·05, 0·71)*	-3·53% (-4·54, -2·61)*	-2·89% (-3·56, -2·19)*	1·38% (-0·33, 3·02)*	2·43% (0·61, 4·52)*
Expected prevalence ARC	-0·59%	0·00%	-2·98%	-3·40%	0·92%	-1·90%
Prevalence ARC difference	0·43%	0·39%	-0·55%	0·52%	0·46%	4·33%
Attributable burden ARC	-5·25%	-7·55%	-10·02%	-7·12%	0·30%	-1·01%
Caribbean						
Observed prevalence ARC, mean (95% UI)	0·11% (-0·44, 0·72)*	0·12% (-0·63, 0·86)*	-0·56% (-1·08, -0·08)*	-0·97% (-1·37, -0·59)*	1·43% (0·42, 2·48)*	0·79% (-0·06, 1·69)*
Expected prevalence ARC	-0·28%	0·07%	-0·74%	-0·94%	0·37%	-0·86%
Prevalence ARC difference	0·40%	0·06%	0·18%	-0·03%	1·06%	1·65%
Attributable burden ARC	-1·08%	-2·83%	-4·38%	-3·37%	0·16%	0·19%
Antigua and Barbuda						
Observed prevalence ARC, mean (95% UI)	0·05% (-1·08, 1·20)*	1·03% (-0·94, 2·91)*	-0·72% (-1·93, 0·45)*	-0·78% (-1·54, 0·02)*	2·00% (-0·32, 4·34)*	-0·25% (-1·90, 1·34)
Expected prevalence ARC	-0·13%	0·00%	-2·81%	-2·50%	0·63%	-1·91%
Prevalence ARC difference	0·18%	1·03%	2·08%	1·72%	1·37%	1·65%
Attributable burden ARC	-2·47%	-3·78%	-4·41%	0·75%	-0·56%	-0·55%
Barbados						
Observed prevalence ARC, mean (95% UI)	-0·14% (-1·28, 0·99)	0·83% (-1·18, 2·93)*	-0·85% (-1·92, 0·20)*	-0·76% (-1·34, -0·14)*	2·26% (0·00, 4·68)*	-0·13% (-1·37, 1·22)
Expected prevalence ARC	-0·10%	0·00%	-2·10%	-1·86%	0·47%	-1·42%
Prevalence ARC difference	-0·05%	0·83%	1·25%	1·10%	1·79%	1·30%
Attributable burden ARC	-0·77%	-4·20%	-5·58%	-3·59%	0·70%	-0·21%
Belize						
Observed prevalence ARC, mean (95% UI)	-0·08% (-1·06, 0·87)	2·03% (0·13, 3·83)*	-1·64% (-2·63, -0·69)*	-2·13% (-2·91, -1·38)*	1·79% (-0·21, 3·96)*	-0·09% (-1·67, 1·44)
Expected prevalence ARC	-1·09%	0·00%	-2·90%	-2·82%	0·92%	-1·10%
Prevalence ARC difference	1·01%	2·03%	1·26%	0·69%	0·86%	1·01%
Attributable burden ARC	-1·78%	-5·26%	-7·71%	-1·99%	-0·72%	-0·37%
Bermuda						
Observed prevalence ARC, mean (95% UI)	0·02% (-1·20, 1·24)*	0·80% (-0·93, 2·71)*	-0·72% (-1·88, 0·56)*	-0·72% (-1·55, 0·06)*	1·79% (-0·29, 3·83)*	-0·41% (-2·22, 1·38)
Expected prevalence ARC	-0·13%	0·00%	-2·50%	-1·50%	0·48%	-1·29%
Prevalence ARC difference	0·15%	0·80%	1·79%	0·77%	1·31%	0·88%
Attributable burden ARC	-4·03%	-7·01%	-5·96%	-3·44%	0·20%	-0·54%
Cuba						
Observed prevalence ARC, mean (95% UI)	0·08% (-0·97, 1·16)*	0·71% (-0·47, 1·85)*	-0·58% (-1·85, 0·70)*	-0·29% (-1·12, 0·48)	2·16% (-0·08, 4·22)*	-0·40% (-2·10, 1·08)
Expected prevalence ARC	-0·53%	0·00%	-3·13%	-3·59%	0·92%	-2·06%
Prevalence ARC difference	0·61%	0·71%	2·55%	3·29%	1·24%	1·67%
Attributable burden ARC	-3·37%	-3·27%	-7·04%	-3·16%	0·95%	-0·69%
Dominica						
Observed prevalence ARC, mean (95% UI)	0·15% (-0·94, 1·19)*	0·77% (-0·85, 2·54)*	-0·18% (-1·49, 1·06)	-0·14% (-0·99, 0·68)	1·57% (-0·09, 3·47)*	0·12% (-0·84, 1·32)*
Expected prevalence ARC	-0·18%	0·00%	-3·54%	-3·27%	0·84%	-2·46%
Prevalence ARC difference	0·32%	0·77%	3·36%	3·13%	0·73%	2·58%
Attributable burden ARC	0·41%	-1·54%	-2·33%	-0·32%	-0·54%	-0·15%

ARC= annualised rate of change. *denotes substantial increase or decrease, based upon consistent direction of mean and >80% of model posteriors.
Prevalence ARC difference= Observed mean ARC - Expected ARC.
Low birthweight prevalence ARC is at birth. Low birthweight attributable burden ARC is in neonatal age group (0 – 27 days).
ARC in attributable burden rate [disability adjusted life-years per 100,000], except for anaemia.
ARC in burden rate for anaemia [years lived with disability per 100,000]

Table S4. Annualised rates of change (ARC) 2012 to 2021: observed prevalence, expected prevalence, difference, attributable burden

	Low birthweight both sexes, birth	Exclusive breastfeeding, both sexes, age <6 months	Child stunting, both sexes, age <5 years	Child wasting, both sexes, age <5 years	Child overweight, both sexes, age 2–4 years	Anaemia, females, age 15–49 years
Dominican Republic						
Observed prevalence ARC, mean (95% UI)	-0·12% (-1·10, 0·86)	-0·09% (-2·80, 2·41)	-2·08% (-3·10, -1·03)*	-1·83% (-2·48, -1·19)*	2·21% (-0·06, 4·44)*	-0·07% (-1·09, 1·26)
Expected prevalence ARC	-0·95%	0·00%	-2·85%	-2·89%	0·89%	-1·22%
Prevalence ARC difference	0·83%	-0·09%	0·78%	1·06%	1·32%	1·15%
Attributable burden ARC	-3·10%	-7·42%	-10·56%	-7·23%	-1·19%	-0·52%
Grenada						
Observed prevalence ARC, mean (95% UI)	0·04% (-0·97, 0·96)*	1·30% (-1·09, 3·51)*	-1·08% (-2·25, 0·16)*	-1·42% (-2·22, -0·60)*	2·41% (0·05, 5·00)*	-0·34% (-1·54, 0·95)
Expected prevalence ARC	-0·55%	0·00%	-3·20%	-3·67%	0·95%	-2·11%
Prevalence ARC difference	0·59%	1·30%	2·12%	2·25%	1·46%	1·77%
Attributable burden ARC	-1·56%	-7·03%	-9·05%	-1·23%	2·30%	-0·72%
Guyana						
Observed prevalence ARC, mean (95% UI)	-0·35% (-1·28, 0·55)	0·79% (-0·96, 2·35)*	-3·37% (-4·32, -2·45)*	-2·27% (-2·92, -1·59)*	1·89% (-0·45, 4·25)*	-0·34% (-1·52, 1·16)
Expected prevalence ARC	-1·08%	0·00%	-4·13%	-4·53%	1·30%	-2·27%
Prevalence ARC difference	0·73%	0·79%	0·76%	2·26%	0·59%	1·93%
Attributable burden ARC	-2·63%	-6·20%	-9·56%	-5·91%	-0·34%	-0·76%
Haiti						
Observed prevalence ARC, mean (95% UI)	-0·32% (-1·22, 0·66)	-0·26% (-1·38, 0·89)	-0·88% (-1·57, -0·25)*	-1·47% (-2·08, -0·88)*	2·61% (0·27, 4·91)*	1·34% (-0·52, 3·28)*
Expected prevalence ARC	-0·39%	0·19%	-0·55%	-0·75%	0·40%	-0·47%
Prevalence ARC difference	0·07%	-0·45%	-0·33%	-0·72%	2·20%	1·81%
Attributable burden ARC	-1·20%	-3·55%	-5·32%	-4·42%	0·95%	-0·13%
Jamaica						
Observed prevalence ARC, mean (95% UI)	-0·25% (-1·22, 0·69)	1·06% (-0·74, 2·67)*	-1·43% (-2·46, -0·32)*	-1·02% (-1·74, -0·30)*	1·93% (-0·45, 4·25)*	0·02% (-1·61, 1·67)*
Expected prevalence ARC	-0·30%	0·00%	-2·46%	-2·81%	0·70%	-1·75%
Prevalence ARC difference	0·05%	1·06%	1·03%	1·78%	1·23%	1·77%
Attributable burden ARC	-2·43%	-2·62%	-6·18%	-4·66%	-0·66%	-0·16%
Puerto Rico						
Observed prevalence ARC, mean (95% UI)	0·03% (-1·04, 1·01)*	0·97% (-0·67, 2·69)*	-0·70% (-2·01, 0·61)*	-0·80% (-1·65, 0·04)*	1·19% (-0·46, 3·04)*	-0·22% (-1·90, 1·36)
Expected prevalence ARC	-0·29%	0·00%	-5·34%	-3·35%	1·07%	-2·84%
Prevalence ARC difference	0·32%	0·97%	4·64%	2·55%	0·12%	2·62%
Attributable burden ARC	-6·02%	-5·03%	-9·34%	-0·32%	-0·22%	-0·28%
Saint Kitts and Nevis						
Observed prevalence ARC, mean (95% UI)	-0·02% (-1·13, 0·98)	0·97% (-0·63, 2·82)*	-0·79% (-2·05, 0·51)*	-1·03% (-1·79, -0·20)*	2·09% (-0·24, 4·36)*	-0·04% (-1·69, 1·65)
Expected prevalence ARC	-0·14%	0·00%	-2·99%	-2·56%	0·66%	-1·98%
Prevalence ARC difference	0·11%	0·97%	2·20%	1·54%	1·44%	1·93%
Attributable burden ARC	-1·06%	-3·30%	-5·14%	0·46%	-0·03%	-0·37%
Saint Lucia						
Observed prevalence ARC, mean (95% UI)	0·03% (-0·97, 1·00)*	1·54% (-1·83, 4·98)*	-0·41% (-1·69, 0·82)	-0·71% (-1·60, 0·19)*	2·64% (-0·09, 5·25)*	0·01% (-1·70, 1·55)*
Expected prevalence ARC	-0·33%	0·00%	-2·31%	-2·66%	0·67%	-1·59%
Prevalence ARC difference	0·36%	1·54%	1·90%	1·95%	1·97%	1·60%
Attributable burden ARC	-1·84%	-3·51%	-5·00%	-1·09%	-0·14%	-0·24%

ARC= annualised rate of change. *denotes substantial increase or decrease, based upon consistent direction of mean and >80% of model posteriors.
Prevalence ARC difference= Observed mean ARC - Expected ARC.
Low birthweight prevalence ARC is at birth. Low birthweight attributable burden ARC is in neonatal age group (0 – 27 days).
ARC in attributable burden rate [disability adjusted life-years per 100,000], except for anaemia.
ARC in burden rate for anaemia [years lived with disability per 100,000]

Table S4. Annualised rates of change (ARC) 2012 to 2021: observed prevalence, expected prevalence, difference, attributable burden

	Low birthweight both sexes, birth	Exclusive breastfeeding, both sexes, age <6 months	Child stunting, both sexes, age <5 years	Child wasting, both sexes, age <5 years	Child overweight, both sexes, age 2–4 years	Anaemia, females, age 15–49 years
Saint Vincent and the Grenadines						
Observed prevalence ARC, mean (95% UI)	0·08% (-1·10, 1·24)*	1·22% (-1·05, 3·28)*	-0·82% (-2·07, 0·44)*	-1·05% (-1·83, -0·26)*	2·54% (-0·14, 5·06)*	-0·04% (-1·60, 1·54)
Expected prevalence ARC	-0·88%	0·00%	-3·40%	-3·69%	0·99%	-1·78%
Prevalence ARC difference	0·96%	1·22%	2·57%	2·64%	1·55%	1·74%
Attributable burden ARC	-4·16%	-4·82%	-7·56%	-0·33%	0·11%	-0·32%
Suriname						
Observed prevalence ARC, mean (95% UI)	-0·19% (-1·16, 0·78)	1·09% (-2·06, 4·11)*	-1·45% (-2·68, -0·28)*	-1·01% (-1·75, -0·33)*	2·23% (-0·34, 4·87)*	-0·09% (-1·70, 1·46)
Expected prevalence ARC	-0·53%	0·00%	-2·22%	-2·48%	0·63%	-1·27%
Prevalence ARC difference	0·34%	1·09%	0·77%	1·47%	1·60%	1·17%
Attributable burden ARC	-1·23%	-3·54%	-5·79%	-3·56%	0·08%	-0·29%
The Bahamas						
Observed prevalence ARC, mean (95% UI)	0·00% (-0·97, 1·00)	0·99% (-0·84, 2·78)*	-0·44% (-1·66, 0·87)	-0·32% (-1·17, 0·46)	1·78% (-0·20, 3·90)*	-0·04% (-1·70, 1·57)
Expected prevalence ARC	-0·12%	0·00%	-2·41%	-1·54%	0·47%	-1·30%
Prevalence ARC difference	0·12%	0·99%	1·97%	1·22%	1·31%	1·26%
Attributable burden ARC	-3·25%	-5·61%	-6·51%	-1·90%	-0·86%	-0·10%
Trinidad and Tobago						
Observed prevalence ARC, mean (95% UI)	-0·06% (-1·02, 0·84)	0·29% (-2·02, 2·69)*	-1·13% (-2·39, 0·12)*	-1·18% (-1·97, -0·43)*	2·26% (-0·28, 4·69)*	-0·18% (-1·82, 1·58)
Expected prevalence ARC	-0·13%	0·00%	-2·80%	-2·19%	0·60%	-1·74%
Prevalence ARC difference	0·07%	0·29%	1·67%	1·01%	1·67%	1·56%
Attributable burden ARC	-2·40%	-5·20%	-8·49%	-5·49%	-3·03%	-0·28%
Virgin Islands						
Observed prevalence ARC, mean (95% UI)	0·08% (-1·00, 1·15)*	0·78% (-0·80, 2·37)*	-0·21% (-1·54, 1·14)	-0·14% (-0·96, 0·66)	0·97% (-0·62, 2·71)*	0·01% (-1·69, 1·62)*
Expected prevalence ARC	-0·12%	0·00%	-1·95%	-1·15%	0·43%	-1·01%
Prevalence ARC difference	0·20%	0·78%	1·75%	1·01%	0·54%	1·02%
Attributable burden ARC	-4·72%	-5·34%	-7·42%	-4·64%	-0·44%	0·27%
Central Latin America						
Observed prevalence ARC, mean (95% UI)	0·02% (-0·26, 0·31)*	0·82% (0·05, 1·57)*	-1·49% (-1·72, -1·25)*	-1·58% (-1·76, -1·39)*	2·32% (1·08, 3·57)*	-0·48% (-1·17, 0·20)*
Expected prevalence ARC	-0·74%	0·02%	-2·00%	-1·99%	0·79%	-1·10%
Prevalence ARC difference	0·76%	0·81%	0·51%	0·41%	1·53%	0·62%
Attributable burden ARC	-3·04%	-7·89%	-7·73%	-6·50%	-0·04%	-1·01%
Colombia						
Observed prevalence ARC, mean (95% UI)	0·32% (-0·77, 1·38)*	-0·53% (-1·65, 0·48)*	-3·16% (-4·36, -1·95)*	-1·95% (-2·63, -1·30)*	3·20% (0·69, 5·70)*	-0·80% (-2·09, 0·55)*
Expected prevalence ARC	-0·92%	0·00%	-3·95%	-4·41%	1·20%	-2·30%
Prevalence ARC difference	1·23%	-0·53%	0·79%	2·46%	2·00%	1·50%
Attributable burden ARC	-6·97%	-5·78%	-11·92%	-4·67%	1·75%	-0·90%
Costa Rica						
Observed prevalence ARC, mean (95% UI)	-0·10% (-1·23, 1·06)	0·58% (-0·65, 1·70)*	-3·09% (-4·60, -1·56)*	-0·24% (-1·20, 0·80)	1·81% (-0·13, 3·80)*	-0·45% (-4·13, 3·46)
Expected prevalence ARC	-0·46%	0·00%	-4·44%	-4·96%	1·15%	-3·19%
Prevalence ARC difference	0·35%	0·58%	1·36%	4·73%	0·66%	2·75%
Attributable burden ARC	-1·56%	-3·37%	-5·19%	-3·84%	0·82%	-0·44%

ARC= annualised rate of change. *denotes substantial increase or decrease, based upon consistent direction of mean and >80% of model posteriors.
Prevalence ARC difference= Observed mean ARC - Expected ARC.
Low birthweight prevalence ARC is at birth. Low birthweight attributable burden ARC is in neonatal age group (0 – 27 days).
ARC in attributable burden rate [disability adjusted life-years per 100,000], except for anaemia.
ARC in burden rate for anaemia [years lived with disability per 100,000]

Table S4. Annualised rates of change (ARC) 2012 to 2021: observed prevalence, expected prevalence, difference, attributable burden

	Low birthweight both sexes, birth	Exclusive breastfeeding, both sexes, age <6 months	Child stunting, both sexes, age <5 years	Child wasting, both sexes, age <5 years	Child overweight, both sexes, age 2–4 years	Anaemia, females, age 15–49 years
El Salvador						
Observed prevalence ARC, mean (95% UI)	-0·07% (-1·08, 0·97)	0·47% (-0·45, 1·30)*	-1·89% (-2·95, -0·80)*	-1·72% (-2·49, -1·01)*	2·52% (-0·08, 5·09)*	-0·79% (-2·02, 0·49)*
Expected prevalence ARC	-1·59%	0·01%	-2·88%	-2·42%	1·13%	-0·67%
Prevalence ARC difference	1·51%	0·47%	0·99%	0·70%	1·39%	-0·12%
Attributable burden ARC	-6·10%	-8·05%	-11·67%	-8·92%	0·78%	-0·80%
Guatemala						
Observed prevalence ARC, mean (95% UI)	-0·22% (-1·23, 0·91)	0·70% (-0·04, 1·35)*	-0·96% (-1·57, -0·34)*	-3·05% (-3·87, -2·35)*	2·39% (0·30, 4·52)*	-0·94% (-2·05, 0·35)*
Expected prevalence ARC	-1·62%	0·05%	-2·64%	-2·28%	1·25%	-0·71%
Prevalence ARC difference	1·40%	0·65%	1·68%	-0·77%	1·13%	-0·23%
Attributable burden ARC	-2·30%	-7·11%	-6·96%	-6·70%	0·50%	-3·29%
Honduras						
Observed prevalence ARC, mean (95% UI)	0·03% (-0·96, 1·15)*	0·86% (-0·55, 2·09)*	-1·94% (-2·84, -1·13)*	-0·29% (-0·92, 0·37)*	2·16% (-0·40, 4·66)*	-0·32% (-1·43, 0·78)
Expected prevalence ARC	-1·07%	0·09%	-1·64%	-1·58%	0·98%	-0·65%
Prevalence ARC difference	1·10%	0·77%	-0·31%	1·30%	1·18%	0·33%
Attributable burden ARC	-4·48%	-7·26%	-8·95%	-7·20%	-1·95%	-0·69%
Mexico						
Observed prevalence ARC, mean (95% UI)	0·07% (-0·20, 0·35)*	1·31% (-0·30, 2·98)*	-1·52% (-1·77, -1·28)*	-1·49% (-1·67, -1·30)*	2·40% (0·10, 4·54)*	-0·56% (-0·82, -0·30)*
Expected prevalence ARC	-0·81%	0·00%	-4·18%	-4·71%	1·21%	-2·55%
Prevalence ARC difference	0·88%	1·31%	2·66%	3·23%	1·19%	1·99%
Attributable burden ARC	-3·20%	-10·42%	-9·29%	-9·17%	-1·08%	-0·84%
Nicaragua						
Observed prevalence ARC, mean (95% UI)	-0·24% (-1·28, 0·87)	0·52% (-0·77, 1·65)*	-1·83% (-2·79, -0·93)*	-2·44% (-3·08, -1·80)*	2·34% (0·27, 4·42)*	-0·30% (-1·46, 0·88)
Expected prevalence ARC	-1·13%	0·05%	-1·80%	-1·61%	0·91%	-0·54%
Prevalence ARC difference	0·88%	0·47%	-0·03%	-0·83%	1·44%	0·24%
Attributable burden ARC	-4·01%	-8·27%	-12·06%	-8·59%	-0·68%	-0·46%
Panama						
Observed prevalence ARC, mean (95% UI)	-0·41% (-1·44, 0·71)	1·25% (-0·77, 3·12)*	-2·63% (-3·59, -1·76)*	-3·25% (-3·98, -2·53)*	2·12% (-0·12, 4·36)*	-1·36% (-4·56, 1·85)*
Expected prevalence ARC	-0·67%	0·00%	-5·54%	-6·20%	1·60%	-3·91%
Prevalence ARC difference	0·26%	1·25%	2·91%	2·95%	0·52%	2·55%
Attributable burden ARC	-2·24%	-6·09%	-8·55%	-5·22%	0·22%	-1·23%
Venezuela						
Observed prevalence ARC, mean (95% UI)	0·00% (-1·05, 1·07)*	1·13% (-1·60, 4·05)*	-0·11% (-1·03, 0·83)	-0·17% (-0·85, 0·48)	2·11% (0·10, 4·16)*	0·22% (-3·65, 4·06)*
Expected prevalence ARC	0·31%	0·00%	0·91%	0·95%	-0·31%	0·42%
Prevalence ARC difference	-0·31%	1·13%	-1·02%	-1·12%	2·42%	-0·20%
Attributable burden ARC	1·44%	-4·04%	-2·45%	-0·01%	0·81%	0·51%
Tropical Latin America						
Observed prevalence ARC, mean (95% UI)	-0·05% (-0·27, 0·17)	0·18% (-0·71, 1·04)*	-0·82% (-1·09, -0·57)*	-1·41% (-1·62, -1·21)*	2·48% (0·55, 4·54)*	-0·44% (-1·70, 0·85)
Expected prevalence ARC	-0·86%	0·01%	-2·31%	-2·27%	0·88%	-1·23%
Prevalence ARC difference	0·81%	0·17%	1·48%	0·86%	1·60%	0·79%
Attributable burden ARC	-5·23%	-13·25%	-14·30%	-13·33%	-1·06%	-0·57%

ARC= annualised rate of change. *denotes substantial increase or decrease, based upon consistent direction of mean and >80% of model posteriors.
Prevalence ARC difference= Observed mean ARC - Expected ARC.
Low birthweight prevalence ARC is at birth. Low birthweight attributable burden ARC is in neonatal age group (0 – 27 days).
ARC in attributable burden rate [disability adjusted life-years per 100,000], except for anaemia.
ARC in burden rate for anaemia [years lived with disability per 100,000]

Table S4. Annualised rates of change (ARC) 2012 to 2021: observed prevalence, expected prevalence, difference, attributable burden

	**Low birthweight both sexes, birth**	**Exclusive breastfeeding, both sexes, age <6 months**	**Child stunting, both sexes, age <5 years**	**Child wasting, both sexes, age <5 years**	**Child overweight, both sexes, age 2–4 years**	**Anaemia, females, age 15–49 years**
Brazil						
Observed prevalence ARC, mean (95% UI)	-0·04% (-0·27, 0·19)	0·19% (-0·73, 1·09)*	-0·71% (-0·99, -0·46)*	-1·30% (-1·51, -1·10)*	2·48% (0·47, 4·56)*	-0·44% (-1·71, 0·90)
Expected prevalence ARC	-0·74%	0·01%	-2·39%	-2·51%	0·87%	-1·36%
Prevalence ARC difference	0·69%	0·19%	1·68%	1·21%	1·61%	0·93%
Attributable burden ARC	-5·32%	-13·67%	-14·44%	-13·71%	-1·16%	-0·55%
Paraguay						
Observed prevalence ARC, mean (95% UI)	-0·16% (-1·15, 0·89)	0·07% (-1·29, 1·38)*	-3·79% (-4·98, -2·69)*	-4·55% (-5·21, -3·93)*	2·36% (0·02, 4·66)*	-0·55% (-2·10, 0·94)
Expected prevalence ARC	-1·17%	0·00%	-3·67%	-4·06%	1·21%	-2·07%
Prevalence ARC difference	1·01%	0·07%	-0·13%	-0·49%	1·15%	1·51%
Attributable burden ARC	-3·00%	-5·08%	-11·39%	-7·48%	1·30%	-1·00%
**North Africa and Middle East**						
**Observed prevalence ARC, mean (95% UI)**	**-0·21% (-0·58, 0·13)***	**1·09% (0·76, 1·40)***	**-1·77% (-2·03, -1·50)***	**-2·57% (-2·77, -2·37)***	**2·93% (2·34, 3·54)***	**-0·37% (-0·84, 0·05)***
**Expected prevalence ARC**	**-1·32%**	**0·05%**	**-3·30%**	**-3·21%**	**1·39%**	**-1·96%**
**Prevalence ARC difference**	**1·11%**	**1·04%**	**1·53%**	**0·64%**	**1·54%**	**1·58%**
**Attributable burden ARC**	**-5·03%**	**-7·23%**	**-9·98%**	**-9·37%**	**0·27%**	**-0·34%**
Afghanistan						
Observed prevalence ARC, mean (95% UI)	-0·95% (-1·96, 0·12)*	-0·25% (-1·06, 0·48)	-1·90% (-2·79, -1·11)*	-2·86% (-3·40, -2·32)*	2·90% (-0·01, 5·76)*	-0·50% (-1·65, 0·63)
Expected prevalence ARC	-0·96%	0·54%	-1·22%	-1·81%	0·98%	-0·99%
Prevalence ARC difference	0·01%	-0·79%	-0·68%	-1·05%	1·92%	0·49%
Attributable burden ARC	-3·44%	-8·82%	-9·59%	-9·30%	-0·91%	-0·99%
Algeria						
Observed prevalence ARC, mean (95% UI)	-0·19% (-1·38, 1·06)	1·41% (-0·16, 2·73)*	-2·17% (-3·33, -1·21)*	-3·06% (-3·65, -2·47)*	3·93% (1·49, 6·38)*	-0·39% (-2·34, 1·59)
Expected prevalence ARC	-0·69%	0·00%	-3·36%	-3·81%	1·02%	-2·07%
Prevalence ARC difference	0·50%	1·41%	1·18%	0·74%	2·91%	1·68%
Attributable burden ARC	-4·64%	-8·18%	-13·33%	-11·23%	2·25%	-0·26%
Bahrain						
Observed prevalence ARC, mean (95% UI)	0·32% (-0·63, 1·26)*	1·07% (-0·14, 2·21)*	-1·05% (-2·19, 0·18)*	-1·94% (-2·69, -1·12)*	3·61% (1·59, 5·87)*	-0·72% (-2·53, 1·22)
Expected prevalence ARC	-0·19%	0·00%	-3·75%	-3·39%	0·89%	-2·57%
Prevalence ARC difference	0·51%	1·07%	2·70%	1·45%	2·72%	1·85%
Attributable burden ARC	-4·02%	-9·65%	-12·68%	-9·74%	1·73%	-0·45%
Egypt						
Observed prevalence ARC, mean (95% UI)	-0·25% (-1·18, 0·65)	0·44% (-0·57, 1·33)*	-2·56% (-3·39, -1·78)*	-3·83% (-4·35, -3·27)*	2·93% (1·33, 4·48)*	-1·34% (-2·59, 0·03)*
Expected prevalence ARC	-2·87%	0·01%	-5·96%	-5·30%	2·17%	-1·71%
Prevalence ARC difference	2·62%	0·43%	3·40%	1·47%	0·76%	0·37%
Attributable burden ARC	-10·08%	-6·71%	-11·59%	-10·65%	0·10%	-1·93%
Iran						
Observed prevalence ARC, mean (95% UI)	0·00% (-0·30, 0·31)*	0·67% (-0·13, 1·39)*	-1·69% (-2·75, -0·58)*	-2·49% (-3·19, -1·78)*	4·06% (1·99, 6·33)*	-0·17% (-1·48, 1·25)
Expected prevalence ARC	-0·38%	0·00%	-3·51%	-3·95%	0·98%	-2·53%
Prevalence ARC difference	0·38%	0·67%	1·82%	1·46%	3·08%	2·36%
Attributable burden ARC	-16·59%	-22·31%	-26·69%	-23·01%	1·64%	-0·29%

ARC= annualised rate of change. *denotes substantial increase or decrease, based upon consistent direction of mean and >80% of model posteriors.
Prevalence ARC difference= Observed mean ARC - Expected ARC.
Low birthweight prevalence ARC is at birth. Low birthweight attributable burden ARC is in neonatal age group (0 – 27 days).
ARC in attributable burden rate [disability adjusted life-years per 100,000], except for anaemia.
ARC in burden rate for anaemia [years lived with disability per 100,000]

Table S4. Annualised rates of change (ARC) 2012 to 2021: observed prevalence, expected prevalence, difference, attributable burden

	Low birthweight both sexes, birth	Exclusive breastfeeding, both sexes, age <6 months	Child stunting, both sexes, age <5 years	Child wasting, both sexes, age <5 years	Child overweight, both sexes, age 2–4 years	Anaemia, females, age 15–49 years
Iraq						
Observed prevalence ARC, mean (95% UI)	-0·07% (-1·12, 0·98)	3·08% (2·00, 4·13)*	-3·25% (-4·03, -2·53)*	-3·93% (-4·50, -3·37)*	2·89% (0·93, 4·81)*	-0·83% (-2·69, 0·90)*
Expected prevalence ARC	-1·98%	0·00%	-7·07%	-7·43%	2·15%	-3·52%
Prevalence ARC difference	1·91%	3·08%	3·83%	3·50%	0·74%	2·69%
Attributable burden ARC	-4·31%	-6·22%	-9·68%	-9·03%	1·02%	-1·01%
Jordan						
Observed prevalence ARC, mean (95% UI)	0·46% (-0·45, 1·37)*	1·50% (0·11, 2·72)*	-1·04% (-2·20, 0·09)*	-1·42% (-2·14, -0·69)*	3·52% (1·36, 5·77)*	1·89% (0·75, 3·28)*
Expected prevalence ARC	-0·30%	0·00%	-4·22%	-4·42%	1·07%	-3·09%
Prevalence ARC difference	0·76%	1·50%	3·19%	3·01%	2·45%	4·97%
Attributable burden ARC	-4·42%	-6·43%	-8·89%	-7·89%	1·80%	0·70%
Kuwait						
Observed prevalence ARC, mean (95% UI)	0·27% (-0·78, 1·31)*	1·38% (0·03, 2·77)*	-1·37% (-2·50, -0·26)*	-1·00% (-1·67, -0·29)*	2·63% (1·08, 4·39)*	0·53% (-1·19, 2·51)*
Expected prevalence ARC	-0·29%	0·00%	-4·67%	-2·66%	0·96%	-2·33%
Prevalence ARC difference	0·56%	1·38%	3·30%	1·66%	1·67%	2·86%
Attributable burden ARC	-2·49%	-3·69%	-6·19%	-5·86%	1·08%	0·75%
Lebanon						
Observed prevalence ARC, mean (95% UI)	-0·09% (-1·11, 0·89)	1·56% (-0·32, 3·14)*	-1·35% (-2·22, -0·45)*	-1·59% (-2·34, -0·88)*	3·81% (1·80, 6·01)*	0·07% (-0·86, 0·94)*
Expected prevalence ARC	-0·24%	0·00%	-4·51%	-4·43%	1·07%	-3·23%
Prevalence ARC difference	0·16%	1·56%	3·16%	2·83%	2·75%	3·30%
Attributable burden ARC	-5·01%	-5·21%	-7·64%	-6·21%	2·55%	-0·07%
Libya						
Observed prevalence ARC, mean (95% UI)	0·05% (-1·13, 1·14)*	0·92% (-0·26, 2·13)*	-0·10% (-0·87, 0·67)	0·12% (-0·55, 0·81)*	3·62% (1·65, 5·74)*	0·43% (-1·22, 2·09)*
Expected prevalence ARC	-0·04%	0·00%	-0·99%	-0·91%	0·19%	-0·69%
Prevalence ARC difference	0·09%	0·92%	0·90%	1·03%	3·43%	1·11%
Attributable burden ARC	1·55%	-2·90%	-5·18%	-2·87%	1·41%	0·85%
Morocco						
Observed prevalence ARC, mean (95% UI)	-0·73% (-1·75, 0·20)*	1·79% (0·45, 3·01)*	-2·33% (-3·28, -1·39)*	-4·31% (-4·95, -3·67)*	2·63% (0·75, 4·47)*	-0·73% (-2·52, 0·85)*
Expected prevalence ARC	-2·13%	0·03%	-3·72%	-3·17%	1·57%	-0·92%
Prevalence ARC difference	1·40%	1·76%	1·40%	-1·14%	1·06%	0·18%
Attributable burden ARC	-5·95%	-9·01%	-15·21%	-12·94%	0·63%	-0·81%
Oman						
Observed prevalence ARC, mean (95% UI)	0·02% (-1·00, 0·98)*	0·70% (0·15, 1·23)*	-0·12% (-1·10, 0·85)	-0·32% (-0·92, 0·24)*	3·94% (1·85, 6·14)*	-0·56% (-2·66, 1·74)
Expected prevalence ARC	-0·21%	0·00%	-4·61%	-3·72%	0·97%	-2·93%
Prevalence ARC difference	0·23%	0·70%	4·49%	3·41%	2·97%	2·37%
Attributable burden ARC	-3·63%	-4·20%	-7·03%	-4·91%	3·19%	0·07%
Palestine						
Observed prevalence ARC, mean (95% UI)	-0·25% (-1·28, 0·89)	1·41% (-0·07, 2·82)*	-2·84% (-3·86, -1·76)*	-5·19% (-5·82, -4·56)*	3·67% (1·61, 5·76)*	-0·44% (-1·78, 1·19)
Expected prevalence ARC	-2·10%	0·00%	-5·59%	-5·44%	1·83%	-2·15%
Prevalence ARC difference	1·85%	1·41%	2·75%	0·25%	1·84%	1·71%
Attributable burden ARC	-6·25%	-7·38%	-11·61%	-12·05%	2·24%	-0·76%

ARC= annualised rate of change. *denotes substantial increase or decrease, based upon consistent direction of mean and >80% of model posteriors.
Prevalence ARC difference= Observed mean ARC - Expected ARC.
Low birthweight prevalence ARC is at birth. Low birthweight attributable burden ARC is in neonatal age group (0 – 27 days).
ARC in attributable burden rate [disability adjusted life-years per 100,000], except for anaemia.
ARC in burden rate for anaemia [years lived with disability per 100,000]

Table S4. Annualised rates of change (ARC) 2012 to 2021: observed prevalence, expected prevalence, difference, attributable burden

	**Low birthweight both sexes, birth**	**Exclusive breastfeeding, both sexes, age <6 months**	**Child stunting, both sexes, age <5 years**	**Child wasting, both sexes, age <5 years**	**Child overweight, both sexes, age 2–4 years**	**Anaemia, females, age 15–49 years**
Qatar						
Observed prevalence ARC, mean (95% UI)	-0·23% (-1·26, 0·73)	1·43% (0·05, 2·94)*	-1·66% (-2·75, -0·55)*	-2·47% (-3·17, -1·78)*	2·89% (1·17, 4·71)*	-0·55% (-2·88, 1·73)
Expected prevalence ARC	-0·32%	0·00%	-5·21%	-3·00%	1·06%	-2·61%
Prevalence ARC difference	0·08%	1·43%	3·55%	0·53%	1·83%	2·06%
Attributable burden ARC	-5·28%	-8·51%	-13·31%	-10·51%	2·18%	0·04%
Saudi Arabia						
Observed prevalence ARC, mean (95% UI)	-0·16% (-1·40, 1·06)	1·24% (0·12, 2·46)*	-1·48% (-2·64, -0·25)*	-2·80% (-3·53, -2·10)*	3·25% (1·54, 5·09)*	-0·38% (-2·24, 1·47)
Expected prevalence ARC	-0·29%	0·00%	-5·74%	-3·80%	1·14%	-3·17%
Prevalence ARC difference	0·14%	1·24%	4·26%	1·01%	2·11%	2·80%
Attributable burden ARC	-8·85%	-10·10%	-14·09%	-9·90%	1·99%	-0·54%
Sudan						
Observed prevalence ARC, mean (95% UI)	-0·65% (-1·68, 0·33)*	1·91% (1·30, 2·51)*	-1·68% (-2·22, -1·13)*	-2·90% (-3·46, -2·31)*	3·88% (1·32, 6·37)*	-0·59% (-2·41, 1·09)
Expected prevalence ARC	-2·09%	0·13%	-3·37%	-3·12%	1·77%	-1·14%
Prevalence ARC difference	1·44%	1·77%	1·70%	0·22%	2·11%	0·55%
Attributable burden ARC	-4·39%	-11·45%	-13·41%	-12·85%	-2·83%	-0·97%
Syria						
Observed prevalence ARC, mean (95% UI)	-0·04% (-1·04, 0·97)	1·38% (-0·06, 2·61)*	-0·81% (-1·35, -0·27)*	-0·97% (-1·59, -0·40)*	3·11% (1·86, 4·36)*	-0·39% (-2·18, 1·40)
Expected prevalence ARC	-0·40%	0·00%	-1·44%	-1·55%	0·44%	-0·73%
Prevalence ARC difference	0·36%	1·38%	0·63%	0·58%	2·67%	0·34%
Attributable burden ARC	-5·64%	-10·03%	-13·40%	-8·11%	1·45%	-0·23%
Tunisia						
Observed prevalence ARC, mean (95% UI)	0·13% (-1·02, 1·28)*	1·81% (-0·89, 4·42)*	-1·37% (-2·35, -0·44)*	-2·57% (-3·16, -1·99)*	3·38% (1·04, 5·84)*	-0·36% (-1·54, 0·79)
Expected prevalence ARC	-0·47%	0·00%	-3·32%	-3·80%	0·97%	-2·30%
Prevalence ARC difference	0·59%	1·81%	1·95%	1·22%	2·42%	1·94%
Attributable burden ARC	-5·92%	-7·77%	-11·52%	-10·58%	1·73%	-0·23%
Türkiye						
Observed prevalence ARC, mean (95% UI)	-0·06% (-1·10, 0·98)	1·17% (-0·35, 2·75)*	-3·52% (-4·69, -2·36)*	-0·85% (-1·51, -0·18)*	3·62% (1·39, 5·63)*	-0·43% (-1·75, 1·15)
Expected prevalence ARC	-0·69%	0·00%	-6·15%	-6·82%	1·68%	-4·35%
Prevalence ARC difference	0·63%	1·17%	2·63%	5·97%	1·94%	3·93%
Attributable burden ARC	-7·20%	-6·93%	-15·15%	-9·84%	1·72%	-0·60%
United Arab Emirates						
Observed prevalence ARC, mean (95% UI)	-0·04% (-1·09, 1·13)	0·52% (-0·25, 1·28)*	-1·03% (-2·08, -0·05)*	-2·47% (-3·23, -1·68)*	2·48% (1·07, 4·02)*	-0·85% (-4·06, 2·26)
Expected prevalence ARC	-0·10%	0·00%	-1·53%	-0·82%	0·31%	-0·73%
Prevalence ARC difference	0·06%	0·52%	0·51%	-1·65%	2·17%	-0·11%
Attributable burden ARC	-6·56%	-8·99%	-12·01%	-9·93%	-0·27%	-0·42%
Yemen						
Observed prevalence ARC, mean (95% UI)	-0·58% (-1·62, 0·45)*	2·40% (-0·07, 4·69)*	-1·00% (-1·61, -0·34)*	-0·42% (-0·92, 0·07)*	3·08% (0·79, 5·38)*	-0·02% (-0·76, 0·78)
Expected prevalence ARC	-0·57%	0·28%	-0·80%	-1·09%	0·58%	-0·69%
Prevalence ARC difference	-0·01%	2·13%	-0·19%	0·67%	2·50%	0·66%
Attributable burden ARC	-3·18%	-7·48%	-9·61%	-7·92%	-0·43%	-0·08%

ARC= annualised rate of change. *denotes substantial increase or decrease, based upon consistent direction of mean and >80% of model posteriors.
Prevalence ARC difference= Observed mean ARC - Expected ARC.
Low birthweight prevalence ARC is at birth. Low birthweight attributable burden ARC is in neonatal age group (0 – 27 days).
ARC in attributable burden rate [disability adjusted life-years per 100,000], except for anaemia.
ARC in burden rate for anaemia [years lived with disability per 100,000]

Table S4. Annualised rates of change (ARC) 2012 to 2021: observed prevalence, expected prevalence, difference, attributable burden

	Low birthweight both sexes, birth	Exclusive breastfeeding, both sexes, age <6 months	Child stunting, both sexes, age <5 years	Child wasting, both sexes, age <5 years	Child overweight, both sexes, age 2–4 years	Anaemia, females, age 15–49 years
**South Asia**						
**Observed prevalence ARC, mean (95% UI)**	**-0·79% (-0·99, -0·57)***	**1·23% (0·79, 1·66)***	**-2·14% (-2·28, -2·01)***	**-3·19% (-3·31, -3·06)***	**2·59% (0·35, 4·77)***	**-0·14% (-0·46, 0·20)**
**Expected prevalence ARC**	**-1·52%**	**0·34%**	**-2·93%**	**-3·33%**	**1·68%**	**-1·91%**
**Prevalence ARC difference**	**0·73%**	**0·90%**	**0·78%**	**0·15%**	**0·91%**	**1·77%**
**Attributable burden ARC**	**-2·65%**	**-9·94%**	**-11·34%**	**-10·95%**	**-3·52%**	**-1·09%**
Bangladesh						
Observed prevalence ARC, mean (95% UI)	-0·99% (-1·85, -0·17)*	1·54% (1·03, 2·02)*	-2·36% (-2·90, -1·83)*	-3·65% (-4·18, -3·11)*	2·99% (0·02, 5·69)*	-0·12% (-2·11, 2·13)
Expected prevalence ARC	-1·56%	0·55%	-2·24%	-2·83%	1·63%	-1·65%
Prevalence ARC difference	0·57%	0·99%	-0·12%	-0·82%	1·36%	1·54%
Attributable burden ARC	-5·32%	-9·97%	-12·14%	-11·79%	0·62%	-0·83%
Bhutan						
Observed prevalence ARC, mean (95% UI)	-0·92% (-2·08, 0·25)*	0·59% (-0·36, 1·43)*	-3·10% (-4·01, -2·24)*	-3·85% (-4·45, -3·24)*	2·26% (-0·20, 4·67)*	-0·33% (-1·96, 1·30)
Expected prevalence ARC	-0·76%	0·30%	-1·15%	-1·52%	0·85%	-0·95%
Prevalence ARC difference	-0·16%	0·30%	-1·94%	-2·32%	1·41%	0·62%
Attributable burden ARC	-3·54%	-7·72%	-10·08%	-9·57%	-0·95%	-1·02%
India						
Observed prevalence ARC, mean (95% UI)	-0·64% (-0·86, -0·43)*	0·93% (0·34, 1·49)*	-2·24% (-2·37, -2·11)*	-3·21% (-3·32, -3·10)*	2·89% (0·22, 5·62)*	-0·24% (-0·56, 0·11)*
Expected prevalence ARC	-2·05%	0·17%	-3·96%	-3·84%	1·82%	-1·62%
Prevalence ARC difference	1·41%	0·76%	1·72%	0·63%	1·07%	1·38%
Attributable burden ARC	-3·22%	-10·18%	-12·03%	-11·38%	-4·00%	-1·25%
Nepal						
Observed prevalence ARC, mean (95% UI)	-1·35% (-2·28, -0·45)*	0·42% (-0·05, 0·84)*	-2·15% (-2·95, -1·38)*	-1·86% (-2·46, -1·30)*	3·00% (0·44, 5·72)*	0·61% (-1·37, 2·86)*
Expected prevalence ARC	-0·84%	0·45%	-1·16%	-1·61%	0·83%	-0·98%
Prevalence ARC difference	-0·51%	-0·02%	-0·99%	-0·25%	2·17%	1·59%
Attributable burden ARC	-4·20%	-10·57%	-12·80%	-11·45%	-1·51%	0·00%
Pakistan						
Observed prevalence ARC, mean (95% UI)	-0·85% (-1·54, -0·18)*	4·15% (3·22, 5·05)*	-1·69% (-2·18, -1·22)*	-2·80% (-3·27, -2·28)*	0·92% (-1·85, 3·44)*	0·35% (-0·87, 1·71)*
Expected prevalence ARC	-1·07%	0·17%	-1·63%	-1·78%	1·08%	-0·90%
Prevalence ARC difference	0·23%	3·99%	-0·06%	-1·01%	-0·16%	1·25%
Attributable burden ARC	-0·75%	-10·18%	-9·60%	-10·02%	-2·05%	-0·42%
**Southeast Asia, east Asia, and Oceania**						
**Observed prevalence ARC, mean (95% UI)**	**0·05% (-0·10, 0·21)***	**0·95% (0·54, 1·38)***	**-1·74% (-2·12, -1·35)***	**-2·46% (-2·66, -2·26)***	**3·02% (2·55, 3·49)***	**-0·02% (-0·35, 0·36)**
**Expected prevalence ARC**	**-0·67%**	**0·01%**	**-3·87%**	**-4·05%**	**1·37%**	**-2·59%**
**Prevalence ARC difference**	**0·72%**	**0·94%**	**2·13%**	**1·59%**	**1·65%**	**2·56%**
**Attributable burden ARC**	**-3·43%**	**-6·15%**	**-9·36%**	**-8·23%**	**0·11%**	**-0·65%**
East Asia						
Observed prevalence ARC, mean (95% UI)	-0·32% (-0·58, -0·06)*	0·59% (-0·03, 1·20)*	-1·89% (-3·10, -0·62)*	-3·19% (-3·88, -2·53)*	3·31% (2·77, 3·87)*	-0·98% (-1·22, -0·75)*
Expected prevalence ARC	-0·76%	0·00%	-4·87%	-5·11%	1·44%	-3·08%
Prevalence ARC difference	0·44%	0·59%	2·98%	1·92%	1·87%	2·10%
Attributable burden ARC	-7·84%	-5·02%	-11·07%	-9·68%	1·23%	-1·40%

ARC= annualised rate of change. *denotes substantial increase or decrease, based upon consistent direction of mean and >80% of model posteriors.
Prevalence ARC difference= Observed mean ARC - Expected ARC.
Low birthweight prevalence ARC is at birth. Low birthweight attributable burden ARC is in neonatal age group (0 – 27 days).
ARC in attributable burden rate [disability adjusted life-years per 100,000], except for anaemia.
ARC in burden rate for anaemia [years lived with disability per 100,000]

Table S4. Annualised rates of change (ARC) 2012 to 2021: observed prevalence, expected prevalence, difference, attributable burden

	Low birthweight both sexes, birth	Exclusive breastfeeding, both sexes, age <6 months	Child stunting, both sexes, age <5 years	Child wasting, both sexes, age <5 years	Child overweight, both sexes, age 2–4 years	Anaemia, females, age 15–49 years
China						
Observed prevalence ARC, mean (95% UI)	-0·32% (-0·59, -0·06)*	0·61% (-0·04, 1·24)*	-1·82% (-3·11, -0·46)*	-3·21% (-3·94, -2·49)*	3·36% (2·81, 3·93)*	-1·04% (-1·29, -0·78)*
Expected prevalence ARC	-0·61%	0·00%	-4·89%	-5·22%	1·41%	-3·34%
Prevalence ARC difference	0·29%	0·61%	3·08%	2·01%	1·95%	2·30%
Attributable burden ARC	-7·95%	-4·91%	-10·94%	-9·54%	1·24%	-1·45%
North Korea						
Observed prevalence ARC, mean (95% UI)	-0·59% (-1·68, 0·58)*	0·27% (-0·04, 0·59)*	-2·55% (-3·26, -1·81)*	-3·28% (-4·03, -2·52)*	2·87% (0·74, 5·09)*	-0·22% (-1·49, 1·28)
Expected prevalence ARC	-0·78%	0·00%	-1·59%	-1·36%	0·55%	-0·39%
Prevalence ARC difference	0·19%	0·27%	-0·96%	-1·92%	2·32%	0·18%
Attributable burden ARC	-7·05%	-9·46%	-11·88%	-11·79%	1·28%	-0·57%
Taiwan (province of China)						
Observed prevalence ARC, mean (95% UI)	-0·13% (-1·26, 0·96)	0·27% (-0·14, 0·68)*	-0·75% (-2·03, 0·66)*	-0·96% (-1·80, -0·15)*	2·65% (0·67, 4·59)*	-0·11% (-1·64, 1·30)
Expected prevalence ARC	-0·26%	0·00%	-3·10%	-1·59%	0·74%	-1·43%
Prevalence ARC difference	0·13%	0·27%	2·35%	0·63%	1·91%	1·32%
Attributable burden ARC	-1·10%	-4·62%	-8·66%	-5·45%	4·12%	-0·05%
Oceania						
Observed prevalence ARC, mean (95% UI)	0·08% (-0·66, 0·82)*	0·11% (-0·29, 0·48)*	-0·22% (-0·65, 0·25)*	-1·46% (-1·94, -0·99)*	1·02% (-0·69, 2·63)*	0·12% (-1·31, 1·63)*
Expected prevalence ARC	-0·32%	0·19%	-0·48%	-0·69%	0·33%	-0·46%
Prevalence ARC difference	0·41%	-0·08%	0·26%	-0·76%	0·69%	0·58%
Attributable burden ARC	-0·07%	-4·08%	-6·19%	-6·03%	-1·03%	0·13%
American Samoa						
Observed prevalence ARC, mean (95% UI)	-0·17% (-1·22, 0·93)	0·45% (-0·16, 1·12)*	-3·29% (-4·65, -2·01)*	-0·72% (-1·55, 0·01)*	1·03% (-0·15, 2·35)*	-0·09% (-1·71, 1·45)
Expected prevalence ARC	-0·24%	0·00%	-4·05%	-4·21%	0·90%	-2·95%
Prevalence ARC difference	0·07%	0·45%	0·76%	3·49%	0·13%	2·86%
Attributable burden ARC	0·42%	-0·83%	-3·12%	-0·77%	-2·44%	0·07%
Cook Islands						
Observed prevalence ARC, mean (95% UI)	-0·08% (-1·14, 0·92)	0·37% (-0·17, 0·92)*	-3·62% (-5·07, -2·28)*	-0·92% (-1·67, -0·14)*	0·80% (-0·09, 1·79)*	-0·58% (-2·34, 1·04)
Expected prevalence ARC	-0·24%	0·00%	-5·12%	-4·09%	1·08%	-3·23%
Prevalence ARC difference	0·16%	0·37%	1·50%	3·18%	-0·27%	2·65%
Attributable burden ARC	-0·39%	1·12%	0·53%	2·37%	-1·16%	-0·72%
Federated States of Micronesia						
Observed prevalence ARC, mean (95% UI)	-0·25% (-1·39, 0·77)	0·61% (-0·16, 1·46)*	-3·10% (-4·35, -1·96)*	-0·85% (-1·58, -0·10)*	1·63% (-0·01, 3·37)*	-0·27% (-1·87, 1·29)
Expected prevalence ARC	-0·88%	0·00%	-2·00%	-1·80%	0·66%	-0·60%
Prevalence ARC difference	0·63%	0·61%	-1·10%	0·95%	0·97%	0·33%
Attributable burden ARC	-1·73%	-4·60%	-6·85%	-5·04%	-2·48%	-0·26%
Fiji						
Observed prevalence ARC, mean (95% UI)	-0·20% (-1·17, 0·78)	0·44% (-0·17, 1·16)*	-0·46% (-2·03, 0·92)	-2·86% (-3·43, -2·29)*	1·99% (0·15, 3·96)*	-0·37% (-1·58, 1·01)
Expected prevalence ARC	-0·53%	0·00%	-2·90%	-3·33%	0·98%	-1·93%
Prevalence ARC difference	0·32%	0·44%	2·43%	0·47%	1·01%	1·56%
Attributable burden ARC	0·22%	-7·24%	-10·78%	-7·61%	-0·15%	-0·26%

ARC= annualised rate of change. *denotes substantial increase or decrease, based upon consistent direction of mean and >80% of model posteriors.
Prevalence ARC difference= Observed mean ARC - Expected ARC.
Low birthweight prevalence ARC is at birth. Low birthweight attributable burden ARC is in neonatal age group (0 – 27 days).
ARC in attributable burden rate [disability adjusted life-years per 100,000], except for anaemia.
ARC in burden rate for anaemia [years lived with disability per 100,000]

Table S4. Annualised rates of change (ARC) 2012 to 2021: observed prevalence, expected prevalence, difference, attributable burden

	Low birthweight both sexes, birth	Exclusive breastfeeding, both sexes, age <6 months	Child stunting, both sexes, age <5 years	Child wasting, both sexes, age <5 years	Child overweight, both sexes, age 2–4 years	Anaemia, females, age 15–49 years
Guam						
Observed prevalence ARC, mean (95% UI)	-0·06% (-1·04, 0·96)	0·32% (-0·22, 0·92)*	-2·83% (-4·23, -1·36)*	-0·02% (-0·80, 0·78)	0·65% (-0·64, 1·90)*	0·12% (-1·66, 1·89)*
Expected prevalence ARC	-0·20%	0·00%	-3·64%	-2·43%	0·76%	-2·03%
Prevalence ARC difference	0·14%	0·32%	0·81%	2·40%	-0·11%	2·15%
Attributable burden ARC	-0·93%	-5·12%	-7·55%	-4·14%	-2·51%	0·22%
Kiribati						
Observed prevalence ARC, mean (95% UI)	-0·24% (-1·27, 0·75)	0·54% (-0·29, 1·41)*	-0·57% (-1·84, 0·67)*	-1·10% (-1·71, -0·46)*	1·76% (0·14, 3·37)*	-0·21% (-1·90, 1·47)
Expected prevalence ARC	-1·08%	0·03%	-1·69%	-1·48%	0·84%	-0·47%
Prevalence ARC difference	0·84%	0·51%	1·12%	0·38%	0·92%	0·26%
Attributable burden ARC	-0·24%	-5·55%	-6·60%	-5·22%	-2·31%	-0·21%
Marshall Islands						
Observed prevalence ARC, mean (95% UI)	-0·35% (-1·30, 0·56)	0·45% (-0·60, 1·46)*	-1·55% (-2·73, -0·31)*	0·06% (-0·85, 0·94)*	1·58% (-0·12, 3·33)*	-0·30% (-1·59, 0·95)
Expected prevalence ARC	-1·52%	0·00%	-2·98%	-2·53%	1·08%	-0·72%
Prevalence ARC difference	1·18%	0·45%	1·43%	2·58%	0·50%	0·42%
Attributable burden ARC	-1·70%	-5·29%	-8·19%	-5·81%	-2·53%	-0·25%
Nauru						
Observed prevalence ARC, mean (95% UI)	-0·45% (-1·60, 0·67)*	0·89% (0·07, 1·75)*	-2·71% (-3·56, -1·82)*	-3·88% (-4·78, -3·05)*	1·22% (-0·01, 2·39)*	-0·88% (-2·60, 0·80)*
Expected prevalence ARC	-1·96%	0·00%	-5·05%	-4·96%	1·67%	-1·99%
Prevalence ARC difference	1·51%	0·89%	2·34%	1·08%	-0·45%	1·11%
Attributable burden ARC	-1·98%	-7·40%	-9·75%	-8·91%	-4·63%	-1·31%
Niue						
Observed prevalence ARC, mean (95% UI)	-0·03% (-1·14, 0·93)	0·37% (-0·15, 0·96)*	-3·20% (-4·57, -1·85)*	-0·65% (-1·46, 0·07)*	1·33% (-0·07, 2·66)*	-0·30% (-1·93, 1·37)
Expected prevalence ARC	-0·16%	0·00%	-2·63%	-2·66%	0·69%	-1·93%
Prevalence ARC difference	0·13%	0·37%	-0·57%	2·01%	0·64%	1·62%
Attributable burden ARC	7·95%	10·08%	7·90%	8·65%	6·34%	-0·23%
Northern Mariana Islands						
Observed prevalence ARC, mean (95% UI)	-0·08% (-1·23, 0·98)	0·35% (-0·23, 0·89)*	-3·90% (-5·27, -2·53)*	-0·65% (-1·50, 0·14)*	1·05% (-0·06, 2·28)*	-0·58% (-2·82, 1·35)
Expected prevalence ARC	-0·13%	0·00%	-2·79%	-2·07%	0·60%	-1·68%
Prevalence ARC difference	0·05%	0·35%	-1·11%	1·42%	0·44%	1·11%
Attributable burden ARC	-1·92%	-5·20%	-8·11%	-3·06%	-2·68%	-0·89%
Palau						
Observed prevalence ARC, mean (95% UI)	-0·09% (-1·27, 0·97)	0·27% (-0·32, 0·85)*	-3·27% (-4·70, -1·94)*	-0·96% (-1·74, -0·12)*	1·07% (-0·10, 2·40)*	-0·20% (-1·99, 1·50)
Expected prevalence ARC	-0·11%	0·00%	-2·43%	-2·07%	0·51%	-1·61%
Prevalence ARC difference	0·01%	0·27%	-0·84%	1·11%	0·57%	1·41%
Attributable burden ARC	-1·15%	-3·44%	-6·50%	-3·89%	-2·07%	-0·03%
Papua New Guinea						
Observed prevalence ARC, mean (95% UI)	-0·02% (-0·89, 0·84)	0·00% (-0·47, 0·46)*	-0·51% (-0·97, -0·01)*	-1·82% (-2·36, -1·31)*	1·67% (-0·96, 4·22)*	0·08% (-1·68, 1·88)*
Expected prevalence ARC	-0·43%	0·23%	-0·59%	-0·82%	0·43%	-0·50%
Prevalence ARC difference	0·42%	-0·23%	0·08%	-1·00%	1·24%	0·58%
Attributable burden ARC	-0·25%	-4·44%	-6·68%	-6·51%	-0·89%	0·09%

ARC= annualised rate of change. *denotes substantial increase or decrease, based upon consistent direction of mean and >80% of model posteriors.
Prevalence ARC difference= Observed mean ARC - Expected ARC.
Low birthweight prevalence ARC is at birth. Low birthweight attributable burden ARC is in neonatal age group (0 – 27 days).
ARC in attributable burden rate [disability adjusted life-years per 100,000], except for anaemia.
ARC in burden rate for anaemia [years lived with disability per 100,000]

Table S4. Annualised rates of change (ARC) 2012 to 2021: observed prevalence, expected prevalence, difference, attributable burden

	Low birthweight both sexes, birth	Exclusive breastfeeding, both sexes, age <6 months	Child stunting, both sexes, age <5 years	Child wasting, both sexes, age <5 years	Child overweight, both sexes, age 2–4 years	Anaemia, females, age 15–49 years
Samoa						
Observed prevalence ARC, mean (95% UI)	-0·46% (-1·69, 0·59)	0·23% (-0·31, 0·80)*	-1·01% (-2·48, 0·62)*	-0·87% (-1·55, -0·25)*	1·18% (-0·08, 2·51)*	-0·11% (-0·98, 0·83)
Expected prevalence ARC	-0·80%	0·00%	-2·00%	-1·84%	0·63%	-0·64%
Prevalence ARC difference	0·34%	0·23%	0·99%	0·96%	0·56%	0·53%
Attributable burden ARC	-1·80%	-4·11%	-6·40%	-5·43%	-1·19%	-0·24%
Solomon Islands						
Observed prevalence ARC, mean (95% UI)	-0·44% (-1·50, 0·62)	0·13% (-0·19, 0·43)*	-1·18% (-1·68, -0·66)*	-0·02% (-0·67, 0·65)	2·09% (-0·37, 4·52)*	-0·38% (-2·03, 1·30)
Expected prevalence ARC	-0·51%	0·27%	-0·70%	-0·97%	0·50%	-0·59%
Prevalence ARC difference	0·06%	-0·14%	-0·47%	0·95%	1·59%	0·22%
Attributable burden ARC	-1·99%	-4·64%	-6·48%	-5·20%	-1·02%	0·18%
Tokelau						
Observed prevalence ARC, mean (95% UI)	-0·13% (-1·20, 0·94)	0·46% (-0·19, 1·10)*	-3·80% (-5·10, -2·54)*	-2·20% (-2·98, -1·40)*	1·38% (0·06, 2·79)*	-0·52% (-2·31, 1·24)
Expected prevalence ARC	-0·44%	0·00%	-3·38%	-3·85%	0·97%	-2·39%
Prevalence ARC difference	0·31%	0·46%	-0·42%	1·65%	0·41%	1·86%
Attributable burden ARC	17·54%	20·79%	16·03%	17·75%	7·33%	-0·56%
Tonga						
Observed prevalence ARC, mean (95% UI)	-0·21% (-1·53, 1·23)	0·54% (-0·13, 1·30)*	-6·63% (-8·18, -5·00)*	-5·17% (-6·18, -4·22)*	1·05% (0·12, 2·03)*	-0·32% (-1·94, 1·43)
Expected prevalence ARC	-0·91%	0·00%	-2·91%	-3·08%	0·92%	-1·40%
Prevalence ARC difference	0·71%	0·54%	-3·72%	-2·09%	0·13%	1·08%
Attributable burden ARC	-2·39%	-5·23%	-7·20%	-4·94%	-1·00%	-0·40%
Tuvalu						
Observed prevalence ARC, mean (95% UI)	-0·09% (-1·22, 1·08)	0·61% (-0·16, 1·44)*	-2·75% (-4·04, -1·48)*	0·47% (-0·33, 1·28)*	1·83% (0·23, 3·61)*	-0·40% (-2·06, 1·28)
Expected prevalence ARC	-1·44%	0·00%	-2·90%	-2·50%	1·02%	-0·73%
Prevalence ARC difference	1·35%	0·61%	0·15%	2·97%	0·82%	0·33%
Attributable burden ARC	-2·05%	-5·44%	-7·20%	-4·14%	-1·34%	-0·58%
Vanuatu						
Observed prevalence ARC, mean (95% UI)	-0·43% (-1·39, 0·54)	0·86% (-0·32, 1·92)*	-1·24% (-1·90, -0·59)*	-1·50% (-2·22, -0·77)*	2·48% (-0·06, 4·99)*	-0·28% (-1·90, 1·44)
Expected prevalence ARC	-0·49%	0·16%	-0·71%	-0·95%	0·58%	-0·59%
Prevalence ARC difference	0·07%	0·70%	-0·53%	-0·56%	1·90%	0·31%
Attributable burden ARC	-1·19%	-4·72%	-6·29%	-5·52%	-1·55%	-0·01%
Southeast Asia						
Observed prevalence ARC, mean (95% UI)	-0·46% (-0·66, -0·27)*	1·67% (1·29, 2·05)*	-1·71% (-1·90, -1·54)*	-2·28% (-2·44, -2·12)*	1·85% (0·72, 2·98)*	0·10% (-0·54, 0·80)*
Expected prevalence ARC	-0·89%	0·04%	-2·27%	-2·33%	0·97%	-1·46%
Prevalence ARC difference	0·43%	1·63%	0·56%	0·05%	0·87%	1·56%
Attributable burden ARC	-2·97%	-7·40%	-9·32%	-8·02%	-0·99%	-0·63%
Cambodia						
Observed prevalence ARC, mean (95% UI)	-0·42% (-1·60, 0·70)	0·29% (-0·12, 0·67)*	-2·10% (-2·65, -1·58)*	-2·63% (-3·21, -2·04)*	2·19% (-0·35, 4·94)*	-0·35% (-1·75, 1·41)
Expected prevalence ARC	-0·74%	0·28%	-1·05%	-1·40%	0·83%	-0·88%
Prevalence ARC difference	0·32%	0·01%	-1·05%	-1·23%	1·36%	0·53%
Attributable burden ARC	-2·99%	-8·87%	-10·32%	-9·90%	-1·26%	-1·23%

ARC= annualised rate of change. *denotes substantial increase or decrease, based upon consistent direction of mean and >80% of model posteriors.
Prevalence ARC difference= Observed mean ARC - Expected ARC.
Low birthweight prevalence ARC is at birth. Low birthweight attributable burden ARC is in neonatal age group (0 – 27 days).
ARC in attributable burden rate [disability adjusted life-years per 100,000], except for anaemia.
ARC in burden rate for anaemia [years lived with disability per 100,000]

Table S4. Annualised rates of change (ARC) 2012 to 2021: observed prevalence, expected prevalence, difference, attributable burden

	Low birthweight both sexes, birth	Exclusive breastfeeding, both sexes, age <6 months	Child stunting, both sexes, age <5 years	Child wasting, both sexes, age <5 years	Child overweight, both sexes, age 2–4 years	Anaemia, females, age 15–49 years
Indonesia						
Observed prevalence ARC, mean (95% UI)	-0·30% (-0·61, -0·01)*	1·85% (1·22, 2·43)*	-1·40% (-1·53, -1·26)*	-2·25% (-2·38, -2·11)*	1·68% (-0·75, 4·05)*	0·82% (-0·70, 2·55)*
Expected prevalence ARC	-0·91%	0·00%	-3·96%	-4·43%	1·21%	-2·33%
Prevalence ARC difference	0·61%	1·85%	2·56%	2·18%	0·47%	3·14%
Attributable burden ARC	-3·01%	-7·11%	-8·37%	-7·38%	-1·96%	-0·18%
Laos						
Observed prevalence ARC, mean (95% UI)	-0·96% (-2·11, 0·09)*	1·55% (0·78, 2·34)*	-2·66% (-3·38, -1·98)*	-1·82% (-2·47, -1·20)*	2·18% (-0·56, 4·91)*	0·99% (-0·49, 2·76)*
Expected prevalence ARC	-0·92%	0·25%	-1·32%	-1·61%	1·01%	-0·94%
Prevalence ARC difference	-0·04%	1·30%	-1·34%	-0·21%	1·17%	1·93%
Attributable burden ARC	-3·57%	-9·93%	-11·48%	-10·35%	-2·67%	-1·00%
Malaysia						
Observed prevalence ARC, mean (95% UI)	-0·09% (-0·91, 0·73)	0·71% (-0·43, 1·82)*	-0·70% (-1·58, 0·15)*	-1·24% (-1·90, -0·58)*	2·38% (-0·25, 4·85)*	-2·09% (-4·09, -0·05)*
Expected prevalence ARC	-0·20%	0·00%	-3·78%	-3·61%	0·90%	-2·67%
Prevalence ARC difference	0·11%	0·71%	3·07%	2·37%	1·48%	0·58%
Attributable burden ARC	-2·64%	-3·89%	-6·45%	-4·24%	0·76%	-1·41%
Maldives						
Observed prevalence ARC, mean (95% UI)	0·25% (-0·68, 1·20)*	0·68% (-0·22, 1·46)*	-1·79% (-2·69, -0·95)*	-1·85% (-2·52, -1·12)*	3·16% (0·42, 5·92)*	0·97% (-0·60, 2·50)*
Expected prevalence ARC	-0·94%	0·00%	-4·13%	-4·60%	1·18%	-2·40%
Prevalence ARC difference	1·20%	0·68%	2·33%	2·75%	1·99%	3·37%
Attributable burden ARC	-4·27%	-5·99%	-8·37%	-7·41%	-1·18%	0·28%
Mauritius						
Observed prevalence ARC, mean (95% UI)	-0·22% (-1·04, 0·61)	0·80% (-0·26, 1·86)*	-0·93% (-1·80, -0·04)*	-1·20% (-1·94, -0·46)*	2·48% (-0·16, 4·98)*	-0·04% (-1·75, 1·63)
Expected prevalence ARC	-0·27%	0·00%	-3·63%	-3·86%	0·96%	-2·66%
Prevalence ARC difference	0·06%	0·80%	2·69%	2·66%	1·52%	2·62%
Attributable burden ARC	-1·28%	-4·57%	-5·69%	-4·07%	0·26%	-0·54%
Myanmar						
Observed prevalence ARC, mean (95% UI)	-0·89% (-2·01, 0·20)*	3·70% (2·65, 4·73)*	-2·83% (-3·54, -2·19)*	-3·24% (-3·90, -2·52)*	2·19% (-0·64, 4·96)*	0·62% (-0·96, 2·23)*
Expected prevalence ARC	-1·55%	0·06%	-2·22%	-1·99%	1·25%	-0·69%
Prevalence ARC difference	0·66%	3·63%	-0·61%	-1·24%	0·94%	1·31%
Attributable burden ARC	-3·52%	-9·88%	-12·51%	-10·52%	-1·69%	-0·81%
Philippines						
Observed prevalence ARC, mean (95% UI)	-0·76% (-0·92, -0·60)*	1·06% (0·04, 2·02)*	-1·79% (-2·37, -1·26)*	-2·08% (-2·64, -1·49)*	2·51% (-0·40, 5·48)*	-0·58% (-1·48, 0·34)*
Expected prevalence ARC	-1·29%	0·00%	-4·87%	-5·25%	1·48%	-2·56%
Prevalence ARC difference	0·54%	1·06%	3·08%	3·17%	1·03%	1·98%
Attributable burden ARC	-2·60%	-6·16%	-9·05%	-7·88%	-0·32%	-0·57%
Seychelles						
Observed prevalence ARC, mean (95% UI)	-0·04% (-1·10, 1·04)	0·54% (-0·36, 1·49)*	-0·89% (-2·19, 0·44)*	-1·21% (-2·02, -0·38)*	2·46% (0·30, 4·76)*	-0·13% (-1·83, 1·55)
Expected prevalence ARC	-0·22%	0·00%	-3·38%	-3·45%	0·90%	-2·46%
Prevalence ARC difference	0·18%	0·54%	2·48%	2·24%	1·56%	2·33%
Attributable burden ARC	-0·66%	-3·66%	-5·60%	-4·19%	0·21%	-0·45%

ARC= annualised rate of change. *denotes substantial increase or decrease, based upon consistent direction of mean and >80% of model posteriors.
Prevalence ARC difference= Observed mean ARC - Expected ARC.
Low birthweight prevalence ARC is at birth. Low birthweight attributable burden ARC is in neonatal age group (0 – 27 days).
ARC in attributable burden rate [disability adjusted life-years per 100,000], except for anaemia.
ARC in burden rate for anaemia [years lived with disability per 100,000]

Table S4. Annualised rates of change (ARC) 2012 to 2021: observed prevalence, expected prevalence, difference, attributable burden

	Low birthweight both sexes, birth	Exclusive breastfeeding, both sexes, age <6 months	Child stunting, both sexes, age <5 years	Child wasting, both sexes, age <5 years	Child overweight, both sexes, age 2–4 years	Anaemia, females, age 15–49 years
Sri Lanka						
Observed prevalence ARC, mean (95% UI)	-0·60% (-1·26, 0·07)*	0·08% (-0·20, 0·34)*	-0·99% (-1·89, -0·13)*	-1·38% (-1·98, -0·83)*	2·62% (0·05, 5·16)*	0·01% (-1·51, 1·84)*
Expected prevalence ARC	-0·45%	0·00%	-4·10%	-4·60%	1·15%	-2·95%
Prevalence ARC difference	-0·16%	0·08%	3·11%	3·21%	1·48%	2·96%
Attributable burden ARC	-5·97%	-8·41%	-11·47%	-6·31%	-0·71%	-0·85%
Thailand						
Observed prevalence ARC, mean (95% UI)	0·06% (-1·00, 1·06)*	2·77% (0·51, 4·98)*	-1·40% (-2·30, -0·50)*	-0·60% (-1·24, 0·01)*	2·04% (-0·42, 4·27)*	-0·34% (-1·39, 0·72)
Expected prevalence ARC	-0·45%	0·00%	-3·30%	-3·77%	0·95%	-2·29%
Prevalence ARC difference	0·52%	2·77%	1·90%	3·17%	1·09%	1·96%
Attributable burden ARC	-3·90%	-4·38%	-5·77%	-4·37%	1·55%	-0·52%
Timor-Leste						
Observed prevalence ARC, mean (95% UI)	-0·61% (-1·62, 0·31)*	0·55% (-0·09, 1·12)*	-0·75% (-1·24, -0·25)*	-0·03% (-0·55, 0·47)	2·20% (-0·33, 4·63)*	0·63% (-0·98, 2·41)*
Expected prevalence ARC	-0·50%	0·25%	-0·77%	-1·05%	0·50%	-0·66%
Prevalence ARC difference	-0·11%	0·30%	0·02%	1·02%	1·69%	1·29%
Attributable burden ARC	-2·00%	-5·44%	-5·27%	-4·52%	-0·49%	-0·66%
Viet Nam						
Observed prevalence ARC, mean (95% UI)	-0·44% (-1·55, 0·70)	1·64% (-0·28, 3·42)*	-2·09% (-2·75, -1·42)*	-2·75% (-3·31, -2·14)*	1·75% (-0·80, 4·33)*	-0·37% (-1·27, 0·60)*
Expected prevalence ARC	-1·22%	0·00%	-3·31%	-3·34%	1·17%	-1·40%
Prevalence ARC difference	0·78%	1·64%	1·21%	0·59%	0·58%	1·03%
Attributable burden ARC	-4·19%	-7·77%	-10·73%	-10·60%	0·12%	-0·77%
**Sub-Saharan Africa**						
**Observed prevalence ARC, mean (95% UI)**	**-0·58% (-0·74, -0·42)***	**1·79% (1·59, 1·99)***	**-1·59% (-1·72, -1·45)***	**-2·74% (-2·86, -2·61)***	**1·82% (1·19, 2·45)***	**0·13% (-0·21, 0·48)***
**Expected prevalence ARC**	**-0·78%**	**0·37%**	**-1·14%**	**-1·48%**	**0·89%**	**-0·94%**
**Prevalence ARC difference**	**0·20%**	**1·42%**	**-0·45%**	**-1·26%**	**0·93%**	**1·07%**
**Attributable burden ARC**	**-2·32%**	**-7·81%**	**-7·92%**	**-8·65%**	**-1·06%**	**-0·59%**
Central sub-Saharan Africa						
Observed prevalence ARC, mean (95% UI)	-0·82% (-1·46, -0·12)*	1·97% (1·30, 2·57)*	-1·29% (-1·64, -0·94)*	-3·83% (-4·23, -3·40)*	2·48% (1·05, 3·94)*	-0·59% (-1·67, 0·55)*
Expected prevalence ARC	-1·07%	0·53%	-1·61%	-2·21%	1·07%	-1·27%
Prevalence ARC difference	0·24%	1·44%	0·32%	-1·62%	1·41%	0·68%
Attributable burden ARC	-3·48%	-12·79%	-10·98%	-13·82%	-3·70%	-1·53%
Angola						
Observed prevalence ARC, mean (95% UI)	-0·72% (-1·67, 0·27)*	2·17% (1·03, 3·34)*	-1·80% (-2·47, -1·20)*	-3·41% (-4·09, -2·78)*	2·95% (0·72, 5·17)*	-0·59% (-2·41, 1·26)
Expected prevalence ARC	-0·92%	0·45%	-1·60%	-2·08%	0·93%	-1·26%
Prevalence ARC difference	0·20%	1·71%	-0·20%	-1·33%	2·02%	0·68%
Attributable burden ARC	-3·42%	-12·63%	-11·23%	-13·24%	-4·30%	-0·98%
Central African Republic						
Observed prevalence ARC, mean (95% UI)	-0·18% (-1·10, 0·76)	1·06% (-0·09, 2·04)*	-0·47% (-0·95, 0·04)*	-1·88% (-2·47, -1·26)*	2·60% (0·26, 4·77)*	0·01% (-1·13, 1·45)*
Expected prevalence ARC	-0·25%	0·14%	-0·38%	-0·56%	0·26%	-0·31%
Prevalence ARC difference	0·07%	0·92%	-0·09%	-1·32%	2·34%	0·32%
Attributable burden ARC	-1·48%	-5·08%	-5·99%	-5·38%	-0·40%	-0·22%

ARC= annualised rate of change. *denotes substantial increase or decrease, based upon consistent direction of mean and >80% of model posteriors.
Prevalence ARC difference= Observed mean ARC - Expected ARC.
Low birthweight prevalence ARC is at birth. Low birthweight attributable burden ARC is in neonatal age group (0 – 27 days).
ARC in attributable burden rate [disability adjusted life-years per 100,000], except for anaemia.
ARC in burden rate for anaemia [years lived with disability per 100,000]

Table S4. Annualised rates of change (ARC) 2012 to 2021: observed prevalence, expected prevalence, difference, attributable burden

	Low birthweight both sexes, birth	Exclusive breastfeeding, both sexes, age <6 months	Child stunting, both sexes, age <5 years	Child wasting, both sexes, age <5 years	Child overweight, both sexes, age 2–4 years	Anaemia, females, age 15–49 years
Congo (Brazzaville)						
Observed prevalence ARC, mean (95% UI)	-0·50% (-1·52, 0·51)*	2·71% (1·16, 4·19)*	-1·87% (-2·65, -1·09)*	-1·86% (-2·47, -1·25)*	2·94% (0·57, 5·37)*	-0·23% (-1·53, 1·13)
Expected prevalence ARC	-1·87%	0·00%	-3·94%	-3·41%	1·34%	-1·03%
Prevalence ARC difference	1·37%	2·71%	2·08%	1·55%	1·59%	0·79%
Attributable burden ARC	-3·06%	-10·27%	-9·61%	-10·52%	-1·66%	-1·11%
DR Congo						
Observed prevalence ARC, mean (95% UI)	-1·09% (-2·16, -0·08)*	1·93% (1·12, 2·66)*	-1·16% (-1·60, -0·69)*	-4·07% (-4·58, -3·52)*	2·12% (-0·19, 4·33)*	-0·69% (-2·18, 0·88)*
Expected prevalence ARC	-1·14%	0·62%	-1·58%	-2·27%	1·14%	-1·31%
Prevalence ARC difference	0·04%	1·31%	0·42%	-1·80%	0·98%	0·61%
Attributable burden ARC	-3·77%	-14·71%	-11·52%	-15·78%	-4·10%	-1·85%
Equatorial Guinea						
Observed prevalence ARC, mean (95% UI)	-0·69% (-1·77, 0·35)*	1·91% (-0·38, 4·18)*	-1·85% (-2·59, -1·16)*	-2·58% (-3·22, -1·93)*	2·42% (1·04, 4·00)*	-0·60% (-2·54, 1·19)
Expected prevalence ARC	-1·42%	0·00%	-5·56%	-6·09%	1·66%	-3·11%
Prevalence ARC difference	0·72%	1·91%	3·71%	3·51%	0·77%	2·51%
Attributable burden ARC	-2·23%	-7·16%	-7·49%	-8·30%	-1·41%	-0·03%
Gabon						
Observed prevalence ARC, mean (95% UI)	-0·29% (-1·18, 0·60)	2·67% (-0·29, 5·67)*	-1·95% (-2·83, -1·02)*	-2·99% (-3·74, -2·23)*	3·13% (0·71, 5·39)*	-0·28% (-1·54, 1·13)
Expected prevalence ARC	-1·57%	0·00%	-4·88%	-5·03%	1·50%	-2·23%
Prevalence ARC difference	1·28%	2·67%	2·93%	2·04%	1·63%	1·95%
Attributable burden ARC	-3·96%	-9·20%	-10·45%	-10·39%	0·16%	-0·94%
Eastern sub-Saharan Africa						
Observed prevalence ARC, mean (95% UI)	-0·64% (-0·91, -0·37)*	0·86% (0·65, 1·06)*	-1·65% (-1·82, -1·48)*	-2·70% (-2·84, -2·55)*	2·18% (1·39, 2·95)*	0·10% (-0·39, 0·64)*
Expected prevalence ARC	-0·75%	0·41%	-1·05%	-1·37%	0·88%	-0·96%
Prevalence ARC difference	0·10%	0·45%	-0·60%	-1·33%	1·29%	1·06%
Attributable burden ARC	-2·70%	-7·34%	-8·08%	-9·29%	-0·60%	-0·46%
Burundi						
Observed prevalence ARC, mean (95% UI)	-0·06% (-1·21, 1·00)	0·80% (0·56, 1·03)*	-0·25% (-0·76, 0·35)*	-2·20% (-2·74, -1·61)*	2·49% (-0·40, 5·11)*	1·56% (0·22, 3·16)*
Expected prevalence ARC	-0·51%	0·29%	-0·66%	-0·99%	0·52%	-0·53%
Prevalence ARC difference	0·45%	0·50%	0·41%	-1·21%	1·96%	2·09%
Attributable burden ARC	-3·07%	-10·75%	-6·02%	-10·92%	-3·26%	0·57%
Comoros						
Observed prevalence ARC, mean (95% UI)	-0·56% (-1·46, 0·29)*	1·23% (-1·14, 3·60)*	-1·31% (-1·91, -0·76)*	-1·80% (-2·38, -1·25)*	1·88% (0·17, 3·58)*	-0·08% (-1·90, 1·75)
Expected prevalence ARC	-0·71%	0·25%	-1·02%	-1·34%	0·80%	-0·83%
Prevalence ARC difference	0·15%	0·98%	-0·29%	-0·46%	1·07%	0·75%
Attributable burden ARC	-3·18%	-6·67%	-7·64%	-6·35%	-0·69%	-0·77%
Djibouti						
Observed prevalence ARC, mean (95% UI)	-0·27% (-1·22, 0·64)	1·04% (-1·22, 3·20)*	-1·39% (-1·90, -0·85)*	-2·24% (-2·74, -1·74)*	1·63% (-0·52, 3·93)*	-0·50% (-2·32, 1·28)
Expected prevalence ARC	-0·94%	0·27%	-1·37%	-1·69%	1·03%	-0·98%
Prevalence ARC difference	0·67%	0·77%	-0·02%	-0·55%	0·60%	0·48%
Attributable burden ARC	-3·76%	-9·97%	-10·79%	-10·39%	-1·98%	-0·36%

ARC= annualised rate of change. *denotes substantial increase or decrease, based upon consistent direction of mean and >80% of model posteriors.
Prevalence ARC difference= Observed mean ARC - Expected ARC.
Low birthweight prevalence ARC is at birth. Low birthweight attributable burden ARC is in neonatal age group (0 – 27 days).
ARC in attributable burden rate [disability adjusted life-years per 100,000], except for anaemia.
ARC in burden rate for anaemia [years lived with disability per 100,000]

Table S4. Annualised rates of change (ARC) 2012 to 2021: observed prevalence, expected prevalence, difference, attributable burden

	Low birthweight both sexes, birth	Exclusive breastfeeding, both sexes, age <6 months	Child stunting, both sexes, age <5 years	Child wasting, both sexes, age <5 years	Child overweight, both sexes, age 2–4 years	Anaemia, females, age 15–49 years
Eritrea						
Observed prevalence ARC, mean (95% UI)	-1·09% (-2·10, -0·06)*	0·70% (0·12, 1·28)*	-1·37% (-2·08, -0·68)*	-2·39% (-2·89, -1·86)*	3·73% (1·20, 6·37)*	-0·24% (-2·16, 1·69)
Expected prevalence ARC	-0·61%	0·32%	-0·82%	-1·16%	0·60%	-0·69%
Prevalence ARC difference	-0·48%	0·38%	-0·55%	-1·23%	3·12%	0·45%
Attributable burden ARC	-2·69%	-7·99%	-9·27%	-7·93%	0·77%	-0·43%
Ethiopia						
Observed prevalence ARC, mean (95% UI)	-1·48% (-1·98, -0·96)*	1·01% (0·48, 1·52)*	-1·95% (-2·25, -1·66)*	-2·60% (-2·89, -2·33)*	1·76% (-0·86, 4·27)*	-0·61% (-1·08, -0·13)*
Expected prevalence ARC	-1·09%	0·60%	-1·41%	-2·06%	1·10%	-1·15%
Prevalence ARC difference	-0·39%	0·41%	-0·54%	-0·54%	0·66%	0·54%
Attributable burden ARC	-3·21%	-8·87%	-10·80%	-8·59%	-1·91%	-1·31%
Kenya						
Observed prevalence ARC, mean (95% UI)	-0·50% (-0·68, -0·32)*	2·37% (1·59, 3·12)*	-1·22% (-1·57, -0·89)*	-1·65% (-1·95, -1·35)*	1·99% (-0·45, 4·35)*	0·20% (-0·29, 0·72)*
Expected prevalence ARC	-1·62%	0·17%	-2·53%	-2·50%	1·49%	-1·07%
Prevalence ARC difference	1·12%	2·20%	1·31%	0·85%	0·49%	1·27%
Attributable burden ARC	-3·25%	-11·56%	-9·46%	-9·10%	-1·49%	-1·27%
Madagascar						
Observed prevalence ARC, mean (95% UI)	-0·90% (-1·75, 0·01)*	0·80% (-0·01, 1·49)*	-1·22% (-1·89, -0·49)*	-4·56% (-5·18, -3·94)*	2·74% (0·30, 5·17)*	0·00% (-1·22, 1·54)*
Expected prevalence ARC	-0·97%	0·52%	-1·31%	-1·86%	0·97%	-1·09%
Prevalence ARC difference	0·07%	0·28%	0·10%	-2·70%	1·77%	1·10%
Attributable burden ARC	-2·88%	-7·17%	-9·34%	-9·06%	-1·26%	-0·44%
Malawi						
Observed prevalence ARC, mean (95% UI)	-0·22% (-1·24, 0·72)	0·25% (-0·13, 0·61)*	-2·26% (-2·87, -1·69)*	-2·68% (-3·33, -2·02)*	2·21% (0·31, 4·04)*	-0·21% (-2·41, 2·25)
Expected prevalence ARC	-1·04%	0·56%	-1·32%	-1·89%	1·03%	-1·09%
Prevalence ARC difference	0·81%	-0·31%	-0·94%	-0·78%	1·18%	0·88%
Attributable burden ARC	-3·11%	-8·22%	-9·24%	-9·50%	-1·55%	-0·78%
Mozambique						
Observed prevalence ARC, mean (95% UI)	-0·58% (-1·57, 0·45)*	1·50% (0·36, 2·50)*	-1·79% (-2·43, -1·20)*	-3·05% (-3·64, -2·42)*	2·18% (0·07, 4·11)*	0·68% (-0·01, 1·35)*
Expected prevalence ARC	-0·93%	0·52%	-1·18%	-1·76%	0·94%	-0·96%
Prevalence ARC difference	0·34%	0·98%	-0·61%	-1·29%	1·24%	1·63%
Attributable burden ARC	-2·94%	-10·26%	-9·74%	-9·67%	-1·09%	1·64%
Rwanda						
Observed prevalence ARC, mean (95% UI)	-0·25% (-1·33, 0·85)	0·20% (0·04, 0·34)*	-1·59% (-2·21, -0·99)*	-4·20% (-4·83, -3·62)*	2·01% (-0·11, 4·12)*	-0·54% (-1·65, 0·65)*
Expected prevalence ARC	-0·96%	0·51%	-1·33%	-1·85%	0·95%	-1·13%
Prevalence ARC difference	0·71%	-0·30%	-0·25%	-2·36%	1·06%	0·58%
Attributable burden ARC	-4·04%	-8·92%	-10·41%	-9·76%	-0·80%	-1·62%
Somalia						
Observed prevalence ARC, mean (95% UI)	-0·18% (-1·11, 0·78)	0·72% (-1·83, 3·30)*	-0·82% (-1·61, -0·12)*	-1·82% (-2·23, -1·45)*	2·42% (-0·33, 5·13)*	-0·12% (-2·06, 1·74)
Expected prevalence ARC	-0·15%	0·09%	-0·15%	-0·26%	0·16%	-0·11%
Prevalence ARC difference	-0·03%	0·63%	-0·68%	-1·56%	2·26%	-0·01%
Attributable burden ARC	-1·98%	-3·79%	-5·49%	-14·05%	-2·76%	0·22%

ARC= annualised rate of change. *denotes substantial increase or decrease, based upon consistent direction of mean and >80% of model posteriors.
Prevalence ARC difference= Observed mean ARC - Expected ARC.
Low birthweight prevalence ARC is at birth. Low birthweight attributable burden ARC is in neonatal age group (0 – 27 days).
ARC in attributable burden rate [disability adjusted life-years per 100,000], except for anaemia.
ARC in burden rate for anaemia [years lived with disability per 100,000]

Table S4. Annualised rates of change (ARC) 2012 to 2021: observed prevalence, expected prevalence, difference, attributable burden

	Low birthweight both sexes, birth	Exclusive breastfeeding, both sexes, age <6 months	Child stunting, both sexes, age <5 years	Child wasting, both sexes, age <5 years	Child overweight, both sexes, age 2–4 years	Anaemia, females, age 15–49 years
South Sudan						
Observed prevalence ARC, mean (95% UI)	0·12% (-0·92, 1·15)*	-0·46% (-1·66, 0·56)	-0·56% (-1·09, -0·09)*	-1·25% (-1·68, -0·84)*	1·57% (-0·86, 4·03)*	-0·04% (-2·08, 2·07)
Expected prevalence ARC	-0·05%	0·03%	-0·07%	-0·10%	0·05%	-0·05%
Prevalence ARC difference	0·17%	-0·48%	-0·50%	-1·14%	1·53%	0·02%
Attributable burden ARC	0·23%	-2·11%	-1·96%	-1·91%	2·54%	-0·32%
Uganda						
Observed prevalence ARC, mean (95% UI)	-0·66% (-1·71, 0·40)*	0·79% (0·42, 1·15)*	-1·93% (-2·49, -1·40)*	-2·83% (-3·38, -2·31)*	2·82% (0·48, 5·04)*	0·28% (-1·17, 2·13)*
Expected prevalence ARC	-1·08%	0·58%	-1·48%	-2·07%	1·07%	-1·24%
Prevalence ARC difference	0·42%	0·21%	-0·45%	-0·76%	1·75%	1·52%
Attributable burden ARC	-2·73%	-9·15%	-7·12%	-9·13%	0·92%	-2·44%
Tanzania						
Observed prevalence ARC, mean (95% UI)	-0·59% (-1·50, 0·33)*	0·95% (0·34, 1·48)*	-1·73% (-2·32, -1·21)*	-2·97% (-3·52, -2·41)*	2·83% (0·41, 5·13)*	0·30% (-1·74, 2·62)*
Expected prevalence ARC	-1·06%	0·55%	-1·49%	-2·05%	1·05%	-1·26%
Prevalence ARC difference	0·47%	0·40%	-0·24%	-0·92%	1·78%	1·56%
Attributable burden ARC	-2·75%	-9·13%	-8·33%	-9·42%	1·02%	-0·44%
Zambia						
Observed prevalence ARC, mean (95% UI)	-1·09% (-2·12, -0·10)*	0·44% (0·06, 0·79)*	-3·19% (-3·74, -2·66)*	-4·48% (-5·07, -3·95)*	1·27% (-0·61, 3·07)*	-0·47% (-1·51, 0·66)*
Expected prevalence ARC	-1·57%	0·39%	-2·46%	-2·77%	1·59%	-1·44%
Prevalence ARC difference	0·48%	0·04%	-0·73%	-1·71%	-0·31%	0·97%
Attributable burden ARC	-3·60%	-9·85%	-10·37%	-11·90%	-4·22%	-4·16%
Southern sub-Saharan Africa						
Observed prevalence ARC, mean (95% UI)	-0·30% (-0·68, 0·05)*	2·45% (1·53, 3·37)*	-1·68% (-1·94, -1·43)*	-1·99% (-2·20, -1·78)*	1·49% (0·07, 2·94)*	-0·08% (-0·91, 0·81)
Expected prevalence ARC	-0·78%	0·19%	-1·31%	-1·58%	0·83%	-0·94%
Prevalence ARC difference	0·48%	2·26%	-0·37%	-0·42%	0·65%	0·85%
Attributable burden ARC	-0·24%	-6·40%	-7·17%	-5·84%	1·49%	-0·73%
Botswana						
Observed prevalence ARC, mean (95% UI)	-0·21% (-1·26, 0·86)	1·65% (0·29, 3·02)*	-1·29% (-1·95, -0·69)*	-2·17% (-2·75, -1·61)*	2·58% (0·39, 4·96)*	-0·57% (-2·15, 1·15)
Expected prevalence ARC	-0·82%	0·00%	-3·14%	-3·45%	0·98%	-1·72%
Prevalence ARC difference	0·61%	1·65%	1·85%	1·28%	1·60%	1·15%
Attributable burden ARC	-1·43%	-4·09%	-5·26%	-3·61%	2·19%	-0·81%
Eswatini						
Observed prevalence ARC, mean (95% UI)	-0·45% (-1·56, 0·65)	1·78% (0·71, 2·76)*	-2·08% (-2·81, -1·35)*	-2·54% (-3·19, -1·88)*	2·00% (0·06, 4·02)*	-0·40% (-1·45, 0·86)
Expected prevalence ARC	-1·47%	0·00%	-3·08%	-2·69%	1·07%	-0·83%
Prevalence ARC difference	1·02%	1·78%	1·00%	0·15%	0·94%	0·43%
Attributable burden ARC	-1·76%	-8·46%	-8·41%	-7·38%	2·73%	-0·76%
Lesotho						
Observed prevalence ARC, mean (95% UI)	-0·38% (-1·39, 0·62)	1·26% (0·84, 1·70)*	-1·49% (-2·19, -0·84)*	-3·21% (-3·81, -2·66)*	1·18% (-1·15, 3·40)*	-0·42% (-1·28, 0·47)*
Expected prevalence ARC	-1·06%	0·11%	-1·64%	-1·63%	1·00%	-0·71%
Prevalence ARC difference	0·68%	1·15%	0·15%	-1·58%	0·18%	0·29%
Attributable burden ARC	-1·14%	-2·91%	-3·04%	-3·27%	-0·04%	-1·01%

ARC= annualised rate of change. *denotes substantial increase or decrease, based upon consistent direction of mean and >80% of model posteriors.
Prevalence ARC difference= Observed mean ARC - Expected ARC.
Low birthweight prevalence ARC is at birth. Low birthweight attributable burden ARC is in neonatal age group (0 – 27 days).
ARC in attributable burden rate [disability adjusted life-years per 100,000], except for anaemia.
ARC in burden rate for anaemia [years lived with disability per 100,000]

Table S4. Annualised rates of change (ARC) 2012 to 2021: observed prevalence, expected prevalence, difference, attributable burden

	Low birthweight both sexes, birth	Exclusive breastfeeding, both sexes, age <6 months	Child stunting, both sexes, age <5 years	Child wasting, both sexes, age <5 years	Child overweight, both sexes, age 2–4 years	Anaemia, females, age 15–49 years
Namibia						
Observed prevalence ARC, mean (95% UI)	-0·42% (-1·44, 0·70)	1·15% (0·24, 2·02)*	-2·18% (-2·96, -1·47)*	-2·78% (-3·39, -2·12)*	2·58% (0·01, 5·17)*	-1·06% (-3·01, 0·81)*
Expected prevalence ARC	-1·28%	0·00%	-3·53%	-3·48%	1·12%	-1·40%
Prevalence ARC difference	0·86%	1·15%	1·35%	0·70%	1·46%	0·34%
Attributable burden ARC	-2·54%	-6·49%	-7·70%	-5·71%	1·89%	-1·19%
South Africa						
Observed prevalence ARC, mean (95% UI)	-0·16% (-0·56, 0·26)	2·98% (1·05, 4·75)*	-1·53% (-1·84, -1·24)*	-1·58% (-1·82, -1·35)*	1·65% (-0·12, 3·47)*	0·07% (-1·06, 1·24)*
Expected prevalence ARC	-0·35%	0·00%	-2·63%	-3·01%	0·75%	-1·85%
Prevalence ARC difference	0·19%	2·98%	1·10%	1·43%	0·90%	1·92%
Attributable burden ARC	0·55%	-6·66%	-8·66%	-7·50%	1·52%	-0·71%
Zimbabwe						
Observed prevalence ARC, mean (95% UI)	-0·56% (-1·64, 0·44)*	1·74% (0·96, 2·51)*	-2·02% (-2·76, -1·39)*	-2·91% (-3·49, -2·35)*	0·74% (-1·53, 2·88)*	-0·40% (-1·67, 1·13)
Expected prevalence ARC	-0·69%	0·25%	-1·01%	-1·34%	0·78%	-0·84%
Prevalence ARC difference	0·13%	1·48%	-1·01%	-1·57%	-0·03%	0·44%
Attributable burden ARC	-1·30%	-6·12%	-5·60%	-3·73%	1·54%	-0·56%
Western sub-Saharan Africa						
Observed prevalence ARC, mean (95% UI)	-0·52% (-0·71, -0·33)*	3·45% (2·99, 3·88)*	-1·65% (-1·90, -1·40)*	-2·71% (-2·91, -2·48)*	1·43% (0·17, 2·63)*	0·23% (-0·30, 0·79)*
Expected prevalence ARC	-0·72%	0·30%	-1·04%	-1·33%	0·84%	-0·79%
Prevalence ARC difference	0·20%	3·16%	-0·61%	-1·38%	0·59%	1·01%
Attributable burden ARC	-2·21%	-7·71%	-7·78%	-8·01%	-0·82%	-0·57%
Benin						
Observed prevalence ARC, mean (95% UI)	-0·32% (-1·31, 0·64)	0·95% (0·21, 1·68)*	-2·03% (-2·51, -1·58)*	-4·12% (-4·70, -3·56)*	1·70% (-0·62, 4·06)*	1·24% (-0·53, 3·16)*
Expected prevalence ARC	-0·83%	0·45%	-1·10%	-1·58%	0·83%	-0·91%
Prevalence ARC difference	0·50%	0·50%	-0·94%	-2·54%	0·87%	2·15%
Attributable burden ARC	-2·34%	-8·91%	-9·44%	-10·78%	-1·87%	-0·46%
Burkina Faso						
Observed prevalence ARC, mean (95% UI)	-0·42% (-1·21, 0·38)*	2·62% (1·90, 3·38)*	-1·81% (-2·38, -1·28)*	-3·32% (-3·79, -2·86)*	1·03% (-1·17, 3·34)*	-0·02% (-1·20, 1·33)
Expected prevalence ARC	-0·71%	0·41%	-0·87%	-1·32%	0·73%	-0·69%
Prevalence ARC difference	0·29%	2·21%	-0·95%	-2·00%	0·30%	0·67%
Attributable burden ARC	-2·07%	-7·11%	-7·17%	-7·22%	-1·10%	-0·81%
Cabo Verde						
Observed prevalence ARC, mean (95% UI)	-0·45% (-1·46, 0·57)	0·57% (-0·16, 1·33)*	-1·72% (-2·75, -0·74)*	-2·40% (-3·07, -1·68)*	3·14% (0·46, 5·77)*	-0·04% (-1·85, 1·71)
Expected prevalence ARC	-1·58%	0·07%	-2·54%	-2·28%	1·29%	-0·78%
Prevalence ARC difference	1·13%	0·50%	0·82%	-0·12%	1·86%	0·74%
Attributable burden ARC	-5·51%	-7·58%	-11·54%	-8·89%	2·20%	-0·43%
Cameroon						
Observed prevalence ARC, mean (95% UI)	-0·92% (-1·93, 0·19)*	2·21% (0·60, 3·63)*	-1·97% (-2·58, -1·43)*	-3·29% (-3·91, -2·66)*	2·57% (0·44, 4·67)*	-0·35% (-1·31, 0·88)
Expected prevalence ARC	-1·03%	0·39%	-1·48%	-1·94%	1·13%	-1·19%
Prevalence ARC difference	0·11%	1·81%	-0·50%	-1·36%	1·45%	0·84%
Attributable burden ARC	-2·13%	-8·96%	-9·59%	-11·04%	-2·10%	-0·99%

ARC= annualised rate of change. *denotes substantial increase or decrease, based upon consistent direction of mean and >80% of model posteriors.
Prevalence ARC difference= Observed mean ARC - Expected ARC.
Low birthweight prevalence ARC is at birth. Low birthweight attributable burden ARC is in neonatal age group (0 – 27 days).
ARC in attributable burden rate [disability adjusted life-years per 100,000], except for anaemia.
ARC in burden rate for anaemia [years lived with disability per 100,000]

Table S4. Annualised rates of change (ARC) 2012 to 2021: observed prevalence, expected prevalence, difference, attributable burden

	Low birthweight both sexes, birth	Exclusive breastfeeding, both sexes, age <6 months	Child stunting, both sexes, age <5 years	Child wasting, both sexes, age <5 years	Child overweight, both sexes, age 2–4 years	Anaemia, females, age 15–49 years
Chad						
Observed prevalence ARC, mean (95% UI)	-0·49% (-1·60, 0·56)*	-2·44% (-5·27, 0·51)*	-1·32% (-2·05, -0·56)*	-1·09% (-1·57, -0·63)*	1·63% (-0·67, 4·21)*	0·19% (-1·39, 1·78)*
Expected prevalence ARC	-0·60%	0·35%	-0·76%	-1·20%	0·63%	-0·60%
Prevalence ARC difference	0·12%	-2·80%	-0·56%	0·11%	1·00%	0·79%
Attributable burden ARC	-0·86%	-3·41%	-5·33%	-4·52%	-0·51%	-0·36%
Côte d'Ivoire						
Observed prevalence ARC, mean (95% UI)	-0·97% (-1·88, 0·01)*	4·68% (2·77, 6·55)*	-3·02% (-3·75, -2·37)*	-3·92% (-4·46, -3·39)*	1·83% (-0·69, 4·27)*	-0·15% (-1·19, 1·25)
Expected prevalence ARC	-0·81%	0·43%	-1·09%	-1·51%	0·80%	-0·91%
Prevalence ARC difference	-0·16%	4·25%	-1·93%	-2·41%	1·04%	0·77%
Attributable burden ARC	-2·68%	-9·45%	-9·49%	-9·81%	-0·11%	-1·07%
Ghana						
Observed prevalence ARC, mean (95% UI)	-0·97% (-1·99, 0·08)*	-0·04% (-0·85, 0·70)	-3·30% (-4·10, -2·50)*	-3·98% (-4·51, -3·43)*	2·84% (0·22, 5·35)*	-0·16% (-1·83, 1·65)
Expected prevalence ARC	-2·06%	0·02%	-3·68%	-3·12%	1·49%	-0·89%
Prevalence ARC difference	1·09%	-0·06%	0·38%	-0·87%	1·35%	0·73%
Attributable burden ARC	-3·68%	-8·20%	-11·54%	-9·51%	0·97%	-1·56%
Guinea						
Observed prevalence ARC, mean (95% UI)	-0·24% (-1·13, 0·66)	-0·02% (-2·00, 1·75)	-1·57% (-2·16, -1·00)*	-1·84% (-2·41, -1·25)*	1·26% (-1·04, 3·86)*	-0·39% (-1·40, 0·80)
Expected prevalence ARC	-0·86%	0·48%	-1·10%	-1·62%	0·87%	-0·90%
Prevalence ARC difference	0·62%	-0·50%	-0·47%	-0·22%	0·39%	0·51%
Attributable burden ARC	-2·52%	-8·41%	-8·24%	-9·01%	-1·93%	-0·88%
Guinea-Bissau						
Observed prevalence ARC, mean (95% UI)	-0·45% (-1·44, 0·52)*	2·17% (1·26, 3·02)*	-1·77% (-2·39, -1·16)*	-2·75% (-3·42, -2·07)*	2·24% (-0·60, 4·94)*	0·03% (-1·68, 1·83)*
Expected prevalence ARC	-0·71%	0·39%	-0·94%	-1·36%	0·71%	-0·77%
Prevalence ARC difference	0·25%	1·79%	-0·84%	-1·39%	1·53%	0·80%
Attributable burden ARC	-3·28%	-9·95%	-9·91%	-10·55%	-3·45%	-0·66%
Liberia						
Observed prevalence ARC, mean (95% UI)	-0·58% (-1·50, 0·30)*	1·25% (0·52, 1·89)*	-2·39% (-3·22, -1·70)*	-3·44% (-4·10, -2·83)*	2·34% (0·16, 4·66)*	0·93% (-0·42, 2·40)*
Expected prevalence ARC	-0·79%	0·44%	-1·04%	-1·52%	0·79%	-0·86%
Prevalence ARC difference	0·21%	0·82%	-1·35%	-1·92%	1·55%	1·79%
Attributable burden ARC	-2·16%	-9·77%	-8·54%	-10·42%	-2·15%	0·38%
Mali						
Observed prevalence ARC, mean (95% UI)	-1·91% (-2·69, -1·05)*	0·71% (-0·44, 1·77)*	-1·91% (-2·51, -1·37)*	-2·92% (-3·48, -2·36)*	1·01% (-1·74, 3·88)*	0·11% (-1·57, 1·84)*
Expected prevalence ARC	-0·76%	0·44%	-0·93%	-1·44%	0·78%	-0·74%
Prevalence ARC difference	-1·15%	0·27%	-0·98%	-1·48%	0·23%	0·85%
Attributable burden ARC	-2·20%	-8·73%	-7·93%	-5·45%	-3·58%	0·50%
Mauritania						
Observed prevalence ARC, mean (95% UI)	-0·82% (-1·63, 0·04)*	4·09% (3·03, 5·15)*	-2·46% (-3·18, -1·70)*	-3·32% (-3·87, -2·78)*	3·21% (0·51, 5·75)*	-0·32% (-2·13, 1·59)
Expected prevalence ARC	-1·11%	0·24%	-1·58%	-1·88%	1·16%	-1·05%
Prevalence ARC difference	0·29%	3·85%	-0·88%	-1·44%	2·05%	0·73%
Attributable burden ARC	-3·95%	-10·21%	-10·92%	-10·88%	1·47%	-1·17%

ARC= annualised rate of change. *denotes substantial increase or decrease, based upon consistent direction of mean and >80% of model posteriors.
Prevalence ARC difference= Observed mean ARC - Expected ARC.
Low birthweight prevalence ARC is at birth. Low birthweight attributable burden ARC is in neonatal age group (0 – 27 days).
ARC in attributable burden rate [disability adjusted life-years per 100,000], except for anaemia.
ARC in burden rate for anaemia [years lived with disability per 100,000]

Table S4. Annualised rates of change (ARC) 2012 to 2021: observed prevalence, expected prevalence, difference, attributable burden

	**Low birthweight both sexes, birth**	**Exclusive breastfeeding, both sexes, age <6 months**	**Child stunting, both sexes, age <5 years**	**Child wasting, both sexes, age <5 years**	**Child overweight, both sexes, age 2–4 years**	**Anaemia, females, age 15–49 years**
Niger						
Observed prevalence ARC, mean (95% UI)	-0·37% (-1·41, 0·62)	0·66% (-1·27, 2·51)*	-0·70% (-1·28, -0·11)*	-2·03% (-2·50, -1·56)*	0·79% (-1·94, 3·45)*	0·00% (-1·65, 1·71)*
Expected prevalence ARC	-0·44%	0·27%	-0·51%	-0·84%	0·47%	-0·39%
Prevalence ARC difference	0·07%	0·39%	-0·20%	-1·19%	0·32%	0·39%
Attributable burden ARC	-1·58%	-6·85%	-7·12%	-7·80%	-2·02%	-0·12%
Nigeria						
Observed prevalence ARC, mean (95% UI)	-0·24% (-0·43, -0·04)*	6·93% (5·91, 7·88)*	-1·53% (-1·99, -1·07)*	-2·93% (-3·33, -2·51)*	1·23% (-0·95, 3·36)*	0·32% (-0·69, 1·30)*
Expected prevalence ARC	-1·27%	0·27%	-1·88%	-2·12%	1·30%	-1·12%
Prevalence ARC difference	1·02%	6·66%	0·34%	-0·81%	-0·07%	1·44%
Attributable burden ARC	-2·13%	-8·28%	-7·79%	-8·73%	-0·41%	-0·51%
São Tomé and Príncipe						
Observed prevalence ARC, mean (95% UI)	-0·51% (-1·54, 0·47)*	0·91% (0·47, 1·34)*	-3·29% (-4·00, -2·63)*	-4·55% (-5·19, -3·89)*	2·34% (-0·27, 4·89)*	-0·18% (-1·56, 1·42)
Expected prevalence ARC	-1·40%	0·31%	-2·06%	-2·39%	1·43%	-1·29%
Prevalence ARC difference	0·89%	0·60%	-1·22%	-2·16%	0·91%	1·11%
Attributable burden ARC	-6·54%	-10·41%	-16·92%	-11·29%	-0·52%	-0·95%
Senegal						
Observed prevalence ARC, mean (95% UI)	-1·16% (-2·09, -0·17)*	1·06% (0·18, 1·89)*	-3·04% (-3·87, -2·24)*	-2·97% (-3·64, -2·31)*	2·62% (0·05, 5·17)*	0·94% (-0·71, 2·62)*
Expected prevalence ARC	-0·90%	0·48%	-1·24%	-1·75%	0·89%	-1·03%
Prevalence ARC difference	-0·26%	0·58%	-1·80%	-1·22%	1·72%	1·97%
Attributable burden ARC	-3·77%	-10·32%	-13·00%	-12·48%	-1·37%	-0·76%
Sierra Leone						
Observed prevalence ARC, mean (95% UI)	-1·43% (-2·45, -0·36)*	4·62% (3·77, 5·39)*	-2·96% (-3·52, -2·41)*	-4·49% (-5·02, -3·96)*	1·74% (0·01, 3·49)*	-0·12% (-1·28, 1·19)
Expected prevalence ARC	-0·94%	0·52%	-1·22%	-1·78%	0·94%	-1·00%
Prevalence ARC difference	-0·50%	4·10%	-1·74%	-2·72%	0·80%	0·89%
Attributable burden ARC	-2·75%	-11·74%	-10·76%	-8·44%	-1·28%	-0·61%
The Gambia						
Observed prevalence ARC, mean (95% UI)	-1·10% (-2·14, -0·14)*	1·62% (0·93, 2·27)*	-2·75% (-3·58, -1·94)*	-4·10% (-4·74, -3·41)*	1·07% (-1·78, 3·67)*	0·04% (-1·25, 1·58)*
Expected prevalence ARC	-0·71%	0·38%	-0·97%	-1·36%	0·70%	-0·82%
Prevalence ARC difference	-0·38%	1·24%	-1·78%	-2·73%	0·37%	0·85%
Attributable burden ARC	-3·06%	-7·65%	-9·18%	-7·12%	-1·63%	-0·67%
Togo						
Observed prevalence ARC, mean (95% UI)	-0·61% (-1·71, 0·52)*	0·74% (0·02, 1·41)*	-2·05% (-2·84, -1·36)*	-3·75% (-4·32, -3·17)*	2·62% (0·27, 4·95)*	0·76% (-1·00, 2·49)*
Expected prevalence ARC	-0·86%	0·46%	-1·18%	-1·66%	0·85%	-0·99%
Prevalence ARC difference	0·25%	0·28%	-0·87%	-2·08%	1·77%	1·74%
Attributable burden ARC	-2·84%	-8·82%	-9·65%	-10·23%	-0·15%	0·09%[1]

ARC= annualised rate of change. *denotes substantial increase or decrease, based upon consistent direction of mean and >80% of model posteriors.
Prevalence ARC difference= Observed mean ARC - Expected ARC.
Low birthweight prevalence ARC is at birth. Low birthweight attributable burden ARC is in neonatal age group (0 – 27 days).
ARC in attributable burden rate [disability adjusted life-years per 100,000], except for anaemia.
ARC in burden rate for anaemia [years lived with disability per 100,000]

Table S5. Global, regional, and country number of children with stunting, 2012, 2021, and projected for 2030 and 2050

	Stunting, 2012, both sexes, < 5 years number (95% UI)	Stunting, 2021, both sexes, < 5 years number (95% UI)	Stunting, 2030, both sexes, < 5 years number (95% UI)	Stunting, 2050, both sexes, < 5 years number (95% UI)
**Global**	**182783800·2 (181484926·3 - 183957344·0)**	**155708487·1 (154438801·9 - 157009528·8)**	**130264781·9 (88117771·7 - 150593686·7)**	**82511460·3* (53107886·8 - 97954159·5)**
**Central Europe, eastern Europe, and central Asia**	**3954939·0 (3826315·2 - 4089369·1)**	**3206690·6 (3081112·1 - 3341425·0)**	**2269792·2 (1334914·7 - 2890071·9)**	**1506207·2* (823057·9 - 2113131·5)**
Central Asia	1513133·5 (1463955·0 - 1561246·5)	1331482·6 (1270751·2 - 1393128·2)	968369·7 (551686·0 - 1263297·7)	659393·6* (355750·1 - 941410·0)
Armenia	33040·7 (30530·3 - 35458·5)	23200·8 (21006·8 - 25700·7)	12776·0* (7172·0 - 17450·1)	6616·1* (3315·7 - 11015·1)
Azerbaijan	144044·3 (135628·0 - 152554·1)	115245·4 (104850·8 - 125334·9)	81309·6 (45967·5 - 110651·4)	40267·3* (18549·2 - 66408·8)
Georgia	28715·8 (25989·5 - 31565·9)	19427·3 (17214·0 - 22105·3)	13227·8* (7208·5 - 17713·0)	9177·3* (4789·2 - 12521·5)
Kazakhstan	216476·6 (201524·2 - 230951·0)	190887·4 (170890·1 - 210799·1)	145787·6 (73876·9 - 208571·5)	90808·1* (42462·0 - 153819·5)
Kyrgyzstan	124422·9 (117932·1 - 130965·0)	113984·4 (103465·8 - 124774·7)	81893·1 (46885·8 - 107035·9)	51264·3* (27393·1 - 76041·2)
Mongolia	52539·1 (48860·7 - 55999·0)	46911·0 (41913·3 - 51765·3)	34830·3 (17711·3 - 48973·0)	23077·0* (10350·3 - 38563·8)
Tajikistan	315258·5 (300664·2 - 329974·2)	300942·3 (278670·6 - 323135·6)	233917·8 (139865·7 - 305649·2)	188102·2 (107551·9 - 259574·9)
Turkmenistan	72347·1 (68586·1 - 76266·5)	50951·2 (46698·1 - 55774·6)	36486·4 (18995·1 - 52334·8)	20226·7* (9399·5 - 36666·8)
Uzbekistan	529748·9 (487079·9 - 569849·6)	469556·0 (416689·9 - 524212·8)	328141·1 (194855·9 - 430330·2)	229854·5* (115098·6 - 361776·2)
Central Europe	596064·2 (570695·9 - 624108·3)	503334·8 (479207·8 - 528729·9)	353889·7 (192701·8 - 470514·2)	209147·8* (112717·1 - 293780·4)
Albania	41063·8 (38862·8 - 43115·6)	27848·7 (25796·7 - 29869·2)	20814·9 (14328·3 - 24853·4)	9780·0* (5739·6 - 13205·9)
Bosnia and Herzegovina	18423·9 (16921·4 - 20097·0)	15221·1 (13787·2 - 16866·9)	9321·8 (5614·3 - 12291·0)	4227·4* (2325·3 - 5940·4)
Bulgaria	19368·6 (16828·7 - 22139·1)	15781·6 (13693·1 - 17918·2)	11891·4 (6795·8 - 15808·8)	7831·9* (4279·3 - 10839·0)
Croatia	20681·1 (18549·5 - 22960·4)	16863·0 (15079·0 - 18871·5)	11932·3 (5867·9 - 16016·5)	5630·8* (2835·6 - 7963·1)
Czechia	15587·8 (13865·8 - 17422·3)	15181·5 (13670·2 - 17035·9)	11680·9 (6162·5 - 17383·9)	10289·4 (5623·3 - 15263·9)
Hungary	45383·9 (40718·9 - 50396·5)	41658·6 (37256·9 - 46785·3)	34119·0 (17429·8 - 46539·4)	24341·5 (12080·6 - 37038·1)
Montenegro	3939·9 (3644·0 - 4300·8)	3720·8 (3358·2 - 4145·3)	3088·0 (1704·1 - 4164·2)	2136·8 (1174·2 - 2930·1)
North Macedonia	9014·4 (8205·8 - 9905·4)	7295·4 (6465·9 - 8182·2)	5067·9 (2437·3 - 7329·2)	2365·5* (1131·3 - 3586·0)

*mean < the stunting global nutrition target (50% reduction from 2012)

Table S5. Global, regional, and country number of children with stunting, 2012, 2021, and projected for 2030 and 2050

	Stunting, 2012, both sexes, < 5 years number (95% UI)	Stunting, 2021, both sexes, < 5 years number (95% UI)	Stunting, 2030, both sexes, < 5 years number (95% UI)	Stunting, 2050, both sexes, < 5 years number (95% UI)
Poland	183520·5 (163876·6 - 204770·0)	161803·5 (144616·7 - 181062·2)	104342·9 (52374·8 - 145331·8)	59884·2* (28758·0 - 95709·9)
Romania	147938·5 (133599·0 - 162436·7)	118410·7 (104983·6 - 131390·9)	90189·0 (48842·4 - 119299·7)	51820·9* (26799·1 - 72629·7)
Serbia	42818·4 (39356·0 - 46416·8)	35989·3 (32574·8 - 39043·2)	23738·2 (12931·5 - 32055·3)	11171·5* (6005·4 - 16494·1)
Slovakia	29748·5 (26790·1 - 33045·7)	28022·9 (25110·5 - 31068·1)	21247·3 (11067·8 - 29626·4)	14380·9* (7042·1 - 22757·6)
Slovenia	9238·1 (8263·0 - 10227·0)	8087·4 (7224·2 - 9016·9)	6456·2 (3310·2 - 8764·9)	5286·9 (2593·0 - 7695·5)
Eastern Europe	1832106·8 (1718479·3 - 1953287·3)	1355753·3 (1262438·4 - 1458378·3)	947532·9 (583569·1 - 1203408·3)	637665·8* (354937·1 - 876530·0)
Belarus	25575·9 (23018·0 - 28353·7)	19184·6 (16984·8 - 21570·2)	13541·4 (6793·2 - 20204·4)	9960·7* (5220·6 - 15415·0)
Estonia	5470·1 (4799·1 - 6194·8)	4699·2 (4185·6 - 5277·5)	3185·4 (1534·7 - 4658·4)	1940·6* (800·5 - 3340·9)
Latvia	8501·8 (7545·3 - 9640·2)	7106·9 (6247·4 - 8039·2)	3964·8* (1978·7 - 5660·3)	2124·0* (878·7 - 3735·7)
Lithuania	11450·4 (10102·5 - 12911·6)	9077·5 (7889·0 - 10293·1)	5401·0* (2569·3 - 7722·4)	2709·9* (1175·0 - 4628·1)
Moldova	15220·7 (13594·0 - 16787·9)	10116·4 (8876·2 - 11509·3)	5018·9* (2311·3 - 7218·1)	2020·3* (951·2 - 3344·8)
Russia	1192602·0 (1082804·6 - 1312427·1)	932280·2 (842157·4 - 1028402·8)	659807·3 (380164·6 - 888000·6)	466153·4* (227251·5 - 707678·1)
Ukraine	580094·7 (543789·0 - 616945·7)	359159·0 (334135·0 - 385568·5)	256614·1* (175194·6 - 308011·2)	152756·9* (96685·6 - 193675·1)
**High income**	**1786977·4 (1718646·8 - 1865860·3)**	**1490090·5 (1425769·9 - 1562227·1)**	**1314924·4 (676870·3 - 1860494·5)**	**1061929·9 (552978·1 - 1531654·2)**
Australasia	35696·6 (32133·4 - 39796·3)	34073·3 (30669·2 - 37931·6)	33378·8 (15312·8 - 51514·3)	32538·4 (15632·5 - 49895·6)
Australia	27886·0 (24249·7 - 31672·5)	26879·9 (23563·9 - 30600·2)	26437·1 (12322·5 - 40744·8)	26148·2 (12549·6 - 40297·6)
New Zealand	7703·3 (6837·3 - 8704·7)	7289·1 (6378·3 - 8160·0)	6941·7 (3078·0 - 10658·3)	6390·2 (2970·5 - 9962·8)
High-income Asia Pacific	503835·1 (495890·6 - 511547·4)	371976·6 (362592·0 - 381545·6)	342027·2 (201370·1 - 428060·0)	267650·8 (150858·5 - 340361·8)
Brunei	5821·5 (5465·0 - 6177·4)	5542·5 (5123·6 - 5995·8)	4861·0 (3481·5 - 5951·2)	2708·0* (1917·9 - 3332·9)
Japan	427320·8 (420379·4 - 433582·0)	347931·5 (339011·7 - 356601·0)	302997·5 (181322·2 - 372698·7)	239016·5 (137545·1 - 298245·7)
Singapore	9236·5 (8168·2 - 10427·3)	9941·7 (8689·7 - 11415·6)	8568·8 (3647·8 - 13347·6)	8688·1 (3833·0 - 13664·7)

*mean < the stunting global nutrition target (50% reduction from 2012)

Table S5. Global, regional, and country number of children with stunting, 2012, 2021, and projected for 2030 and 2050

	Stunting, 2012, both sexes, < 5 years number (95% UI)	Stunting, 2021, both sexes, < 5 years number (95% UI)	Stunting, 2030, both sexes, < 5 years number (95% UI)	Stunting, 2050, both sexes, < 5 years number (95% UI)
South Korea	60763·7 (56673·5 - 65204·6)	30041·4* (26611·6 - 33824·2)	25599·8* (10459·3 - 38237·9)	17238·2* (6794·6 - 26240·9)
High-income North America	562605·3 (505365·6 - 626281·8)	473121·8 (413851·0 - 540507·7)	452744·2 (206661·1 - 702685·0)	388899·6 (182169·7 - 610929·4)
Canada	16093·0 (14209·5 - 18094·5)	14201·0 (12563·5 - 15934·1)	14497·2 (6640·2 - 22550·6)	15328·6 (7312·6 - 24394·1)
Greenland	126·1 (110·8 - 144·8)	105·8 (92·0 - 120·5)	86·6 (38·5 - 134·0)	64·1 (30·9 - 97·7)
USA	546546·4 (489794·9 - 610675·3)	459234·4 (399532·6 - 526416·4)	438153·3 (199836·1 - 681428·8)	373500·7 (174731·6 - 590227·8)
Southern Latin America	345082·2 (313743·0 - 377965·0)	276459·7 (248222·1 - 306645·3)	206886·7 (104308·4 - 290519·2)	121454·0* (61799·0 - 174388·2)
Argentina	290709·1 (259573·0 - 323916·4)	230303·5 (203115·0 - 260734·8)	173040·7 (85099·0 - 244955·9)	99107·8* (49463·7 - 144397·4)
Chile	27961·0 (25091·2 - 31185·8)	22192·4 (19610·5 - 25197·5)	18321·0 (10178·2 - 25896·9)	13016·6* (7662·5 - 19330·2)
Uruguay	25125·0 (23097·7 - 27485·8)	19929·9 (17851·1 - 22087·8)	15514·7 (7977·6 - 20798·7)	9323·5* (4655·7 - 12806·1)
Western Europe	340958·0 (325032·0 - 356777·7)	303516·6 (289514·4 - 317494·4)	279690·3 (153080·8 - 408210·5)	251237·4 (142670·5 - 363804·9)
Andorra	46·7 (41·4 - 52·8)	32·4 (28·9 - 36·3)	30·7 (16·2 - 46·4)	20·2* (11·0 - 30·2)
Austria	5877·9 (5273·2 - 6507·4)	6217·9 (5602·3 - 6886·6)	5971·8 (3193·8 - 8893·8)	5305·9 (2946·7 - 7920·8)
Belgium	9368·0 (8365·9 - 10429·9)	8226·3 (7342·9 - 9253·8)	7679·9 (4092·6 - 11578·0)	7364·0 (4094·5 - 10928·9)
Cyprus	1200·1 (1072·4 - 1346·0)	1303·6 (1174·7 - 1459·7)	1057·7 (597·6 - 1537·5)	860·3 (505·6 - 1270·3)
Denmark	4953·8 (4406·5 - 5545·7)	4754·1 (4261·6 - 5287·0)	4868·3 (2648·2 - 7345·4)	4334·4 (2436·2 - 6484·8)
Finland	4847·5 (4358·7 - 5458·6)	3746·1 (3358·1 - 4175·4)	3584·9 (1921·5 - 5393·0)	3124·6 (1717·5 - 4641·1)
France	59421·8 (53150·3 - 66644·6)	50567·0 (45162·1 - 56327·5)	45792·4 (24079·3 - 66579·4)	37501·3 (20601·4 - 55150·4)
Germany	45607·7 (41408·2 - 50404·7)	50991·6 (46177·0 - 56157·1)	48167·5 (26077·9 - 72089·7)	45252·8 (24808·4 - 68448·7)
Greece	11101·5 (9769·7 - 12582·0)	8872·5 (7793·7 - 10213·8)	7243·8 (3963·5 - 10535·5)	5172·5* (2913·2 - 7568·0)
Iceland	354·9 (319·1 - 396·4)	320·6 (287·8 - 357·2)	358·5 (187·6 - 540·5)	374·5 (199·8 - 564·4)
Ireland	5257·4 (4711·8 - 5872·3)	3973·2 (3565·4 - 4423·3)	3626·7 (1986·8 - 5293·6)	3848·4 (2214·7 - 5613·2)

*mean < the stunting global nutrition target (50% reduction from 2012)

Table S5. Global, regional, and country number of children with stunting, 2012, 2021, and projected for 2030 and 2050

	**Stunting, 2012, both sexes, < 5 years number (95% UI)**	**Stunting, 2021, both sexes, < 5 years number (95% UI)**	**Stunting, 2030, both sexes, < 5 years number (95% UI)**	**Stunting, 2050, both sexes, < 5 years number (95% UI)**
Israel	12143·6 (10892·6 - 13628·9)	12833·5 (11443·5 - 14324·7)	12082·4 (6433·8 - 18011·2)	12858·8 (7151·0 - 19057·4)
Italy	40122·5 (36052·8 - 44789·1)	30077·1 (27106·2 - 33299·1)	25446·3 (13846·1 - 38039·9)	18615·2* (10647·7 - 28226·3)
Luxembourg	485·0 (435·6 - 541·6)	510·4 (456·7 - 568·4)	561·9 (299·2 - 836·6)	639·5 (347·7 - 952·6)
Malta	351·6 (313·0 - 391·0)	341·7 (306·0 - 381·3)	336·1 (185·8 - 492·1)	327·8 (190·4 - 471·0)
Monaco	19·2 (17·2 - 21·4)	19·3 (17·2 - 21·5)	15·8 (8·4 - 23·9)	12·8 (7·0 - 19·5)
Netherlands	13299·7 (11871·9 - 14919·1)	12468·0 (11192·6 - 14063·6)	12936·9 (6756·2 - 19294·0)	11233·5 (6061·5 - 16607·5)
Norway	4537·5 (4059·9 - 5051·8)	3924·2 (3522·6 - 4416·8)	3851·7 (2034·6 - 5778·7)	3706·2 (2011·9 - 5614·9)
Portugal	8263·5 (7398·1 - 9259·2)	6930·7 (6237·9 - 7732·7)	6416·4 (3547·9 - 9414·1)	4988·9 (2958·5 - 7360·1)
San Marino	22·1 (19·7 - 24·7)	17·2 (15·4 - 19·3)	16·6 (8·2 - 25·0)	11·8 (6·0 - 17·9)
Spain	29001·6 (25884·1 - 32253·8)	21247·7 (18954·0 - 23694·1)	20644·8 (12049·6 - 28584·7)	20472·6 (12641·9 - 28592·3)
Sweden	8348·4 (7482·7 - 9290·6)	8201·7 (7375·0 - 9202·8)	8049·6 (4207·8 - 12065·1)	9013·4 (4831·5 - 13540·5)
Switzerland	5717·0 (5127·3 - 6369·3)	5988·3 (5389·8 - 6655·2)	5638·2 (2939·3 - 8520·4)	5408·0 (2886·1 - 8130·5)
UK	70233·3 (59395·7 - 82612·4)	61381·9 (52256·3 - 71606·5)	55067·6 (32494·0 - 78040·5)	50571·0 (30744·9 - 72099·2)
**Latin America and Caribbean**	**7206252·4 (7119858·7 - 7303396·0)**	**6111967·8 (6009996·4 - 6217150·4)**	**4831328·8 (2696878·6 - 6286092·2)**	**2682500·6* (1400833·6 - 3662486·4)**
Andean Latin America	1201058·2 (1165047·0 - 1237095·9)	1027673·4 (975640·4 - 1078899·5)	773901·9 (408657·1 - 1052468·9)	404328·4* (189666·2 - 627159·5)
Bolivia	262364·8 (247090·2 - 278261·3)	215653·7 (194340·9 - 236271·4)	169425·8 (87239·0 - 241841·1)	91704·8* (37008·2 - 160264·9)
Ecuador	382020·1 (357118·3 - 407157·9)	337087·9 (307661·3 - 366169·6)	274114·1 (163064·5 - 354242·1)	168289·7* (91055·8 - 252160·8)
Peru	556478·8 (534155·5 - 578430·3)	465548·1 (423387·7 - 507594·2)	330362·1 (153696·3 - 491097·5)	144333·9* (56333·2 - 280436·5)
Caribbean	544859·4 (526872·1 - 564064·2)	516702·7 (492205·3 - 540843·1)	421536·6 (257250·6 - 543517·9)	221507·8* (119441·7 - 301771·0)
Antigua and Barbuda	468·0 (411·5 - 530·0)	376·7 (333·2 - 424·7)	319·4 (146·1 - 465·7)	188·1* (87·1 - 282·2)
Barbados	2011·1 (1825·1 - 2214·6)	1486·6 (1347·6 - 1639·2)	1234·3 (720·4 - 1650·6)	743·2* (435·6 - 998·7)

*mean < the stunting global nutrition target (50% reduction from 2012)

Table S5. Global, regional, and country number of children with stunting, 2012, 2021, and projected for 2030 and 2050

	Stunting, 2012, both sexes, < 5 years number (95% UI)	Stunting, 2021, both sexes, < 5 years number (95% UI)	Stunting, 2030, both sexes, < 5 years number (95% UI)	Stunting, 2050, both sexes, < 5 years number (95% UI)
Belize	6419·3 (5977·3 - 6877·1)	5588·9 (5049·2 - 6111·8)	5320·1 (3167·5 - 6955·6)	3775·2 (1917·2 - 5774·2)
Bermuda	193·7 (168·9 - 218·2)	133·9 (117·8 - 151·3)	103·9 (41·7 - 156·6)	68·2* (27·2 - 106·2)
Cuba	38900·0 (35094·8 - 42839·4)	31774·7 (28509·8 - 35532·3)	25639·9 (13288·5 - 38056·1)	16759·0* (8694·5 - 26535·6)
Dominica	373·4 (330·2 - 421·7)	259·4 (228·0 - 293·8)	221·3 (108·2 - 318·0)	137·9* (65·6 - 204·8)
Dominican Republic	86736·5 (80458·5 - 93418·5)	77778·3 (70210·0 - 85579·3)	60561·5 (28805·9 - 87359·3)	33836·3* (14487·7 - 61391·9)
Grenada	777·2 (682·3 - 874·4)	556·0 (491·4 - 627·1)	479·9 (234·3 - 678·6)	254·5* (116·5 - 386·8)
Guyana	11251·7 (10538·2 - 11964·0)	8339·4 (7539·1 - 9189·1)	5087·1* (2688·8 - 6668·9)	2785·8* (1302·1 - 4242·6)
Haiti	337160·8 (321407·1 - 352738·1)	350852·4 (328585·1 - 372928·5)	286156·6 (177320·1 - 361460·3)	144459·5* (77571·9 - 202422·1)
Jamaica	15193·4 (13787·9 - 16748·6)	10943·4 (9725·0 - 12315·7)	8285·8 (4151·6 - 11850·0)	3782·1* (1857·0 - 5823·0)
Puerto Rico	11165·5 (9791·4 - 12730·8)	5366·3* (4670·6 - 6130·7)	3841·5* (1583·7 - 5788·7)	1538·3* (617·2 - 2420·0)
Saint Kitts and Nevis	253·2 (223·9 - 287·3)	216·5 (190·5 - 246·0)	164·1 (74·7 - 241·4)	85·0* (39·0 - 128·6)
Saint Lucia	368·0 (326·3 - 414·6)	294·8 (261·7 - 332·0)	232·6 (133·3 - 334·4)	127·2* (78·4 - 178·5)
Saint Vincent and the Grenadines	748·0 (654·4 - 847·4)	560·2 (494·3 - 635·9)	422·3 (197·0 - 610·7)	258·4* (119·8 - 403·5)
Suriname	4471·9 (4068·1 - 4912·3)	3695·0 (3274·8 - 4162·0)	3260·7 (1614·5 - 4784·8)	1962·2* (921·3 - 3243·5)
The Bahamas	2014·6 (1766·0 - 2282·8)	1548·1 (1363·5 - 1755·8)	1457·3 (726·8 - 2075·9)	1074·3 (523·0 - 1574·1)
Trinidad and Tobago	8453·6 (7663·6 - 9309·1)	6464·9 (5736·8 - 7272·3)	4265·5 (2137·5 - 6482·2)	2035·4* (1102·4 - 3049·5)
Virgin Islands	432·3 (376·4 - 491·0)	249·9 (219·5 - 284·7)	203·1* (99·0 - 294·0)	133·7* (62·7 - 201·1)
Central Latin America	3793348·7 (3722809·2 - 3867594·8)	2888007·6 (2819892·1 - 2965359·6)	2301064·4 (1343590·3 - 2948363·3)	1199707·7* (680883·1 - 1592209·9)
Colombia	425373·6 (394350·6 - 457794·5)	296558·1 (263977·3 - 332644·5)	232130·3 (95525·6 - 340646·1)	121382·1* (44159·4 - 209653·7)
Costa Rica	16810·4 (14669·3 - 19066·5)	10979·9 (9522·4 - 12736·1)	7835·1* (3892·8 - 11263·7)	4147·7* (1900·8 - 6230·9)
El Salvador	108626·7 (101272·8 - 115591·5)	85609·3 (76409·1 - 94986·0)	52724·9* (26818·8 - 73358·1)	17721·4* (7841·8 - 27539·6)

*mean < the stunting global nutrition target (50% reduction from 2012)

Table S5. Global, regional, and country number of children with stunting, 2012, 2021, and projected for 2030 and 2050

	**Stunting, 2012, both sexes, < 5 years number (95% UI)**	**Stunting, 2021, both sexes, < 5 years number (95% UI)**	**Stunting, 2030, both sexes, < 5 years number (95% UI)**	**Stunting, 2050, both sexes, < 5 years number (95% UI)**
Guatemala	737456·4 (697738·2 - 785132·1)	617721·7 (586406·3 - 657808·1)	567645·9 (401732·6 - 664407·5)	318339·9* (205675·7 - 390500·6)
Honduras	251085·5 (237324·6 - 265775·4)	224729·4 (207630·5 - 243581·7)	172170·5 (103879·6 - 221862·7)	77229·6* (42483·4 - 107081·7)
Mexico	1680306·3 (1646882·0 - 1711598·2)	1221241·2 (1191792·8 - 1250276·7)	925478·7 (476685·0 - 1254680·9)	469487·6* (230694·4 - 723122·7)
Nicaragua	114467·2 (108701·3 - 120451·7)	95755·1 (86899·1 - 104435·4)	62663·4 (32559·9 - 85300·0)	25156·7* (12430·1 - 35741·9)
Panama	65164·8 (61100·0 - 69909·7)	53610·6 (48426·6 - 59059·0)	46628·0 (23881·2 - 63622·9)	34898·8 (15826·6 - 57160·7)
Venezuela	394403·6 (363581·6 - 426637·1)	309840·2 (281683·4 - 341635·4)	233787·6 (141113·0 - 299973·7)	131343·9* (75201·2 - 178016·2)
Tropical Latin America	1682915·1 (1640418·4 - 1724192·9)	1598999·0 (1555687·3 - 1643883·6)	1335699·8 (675064·1 - 1819536·5)	857001·6 (393568·9 - 1267664·9)
Brazil	1615677·3 (1574662·3 - 1657042·3)	1553415·3 (1511843·5 - 1596087·9)	1302937·4 (658866·3 - 1768671·0)	842289·3 (386184·0 - 1241600·8)
Paraguay	67255·6 (61356·7 - 73831·6)	46472·4 (41207·1 - 52169·6)	32762·4* (15212·9 - 50672·1)	14712·3* (6945·6 - 28434·3)
**North Africa and Middle East**	**14593406·6 (14319635·3 - 14874672·7)**	**12118686·8 (11818441·5 - 12441399·6)**	**10164972·8 (6554738·4 - 12119710·1)**	**6387375·8* (3710849·6 - 8423252·9)**
Afghanistan	1800925·0 (1666201·2 - 1963920·5)	1963046·7 (1838326·0 - 2125354·4)	2174284·2 (1211419·3 - 2699825·8)	1723981·4 (906415·7 - 2475786·7)
Algeria	631847·4 (574302·8 - 684834·8)	578047·5 (516891·4 - 636874·4)	338160·8 (174005·3 - 468701·3)	136474·8* (65553·0 - 240509·3)
Bahrain	10222·9 (9153·7 - 11387·2)	9068·0 (8132·3 - 10180·9)	6048·4 (3026·1 - 9027·5)	3508·8* (2062·8 - 5175·2)
Egypt	2931213·3 (2789138·7 - 3072252·8)	2385631·5 (2197489·6 - 2569607·6)	1790367·3 (1015915·3 - 2348023·2)	1015691·8* (488330·5 - 1761026·7)
Iran	712489·7 (644211·0 - 783763·8)	563105·9 (499395·4 - 634265·7)	330911·7* (155829·6 - 466394·7)	179481·8* (80930·1 - 286575·8)
Iraq	1090385·6 (1039233·9 - 1137998·6)	769949·6 (711396·2 - 828456·5)	711154·4 (413627·4 - 954778·7)	437719·2* (171749·4 - 713334·9)
Jordan	95474·0 (86428·2 - 104915·3)	93135·8 (82637·1 - 104804·6)	90338·2 (43456·1 - 131416·0)	75130·0 (34176·9 - 123857·7)
Kuwait	13592·0 (12193·8 - 14984·0)	11564·2 (10250·0 - 12908·8)	9129·9 (4146·6 - 14103·2)	8376·7 (3868·2 - 13827·8)
Lebanon	63996·7 (58925·0 - 68716·4)	57584·5 (52283·8 - 63042·4)	38064·2 (18884·8 - 52081·7)	20103·6* (9217·2 - 34027·6)
Libya	110519·1 (102879·1 - 118280·3)	82790·0 (76183·3 - 89416·4)	64176·6 (36821·1 - 81126·1)	37064·1* (20675·2 - 49847·4)
Morocco	627155·1 (583519·5 - 667850·4)	442265·6 (400103·8 - 482678·6)	280928·8* (153936·9 - 373710·1)	88717·3* (46167·8 - 123324·0)

*mean < the stunting global nutrition target (50% reduction from 2012)

Table S5. Global, regional, and country number of children with stunting, 2012, 2021, and projected for 2030 and 2050

	Stunting, 2012, both sexes, < 5 years number (95% UI)	Stunting, 2021, both sexes, < 5 years number (95% UI)	Stunting, 2030, both sexes, < 5 years number (95% UI)	Stunting, 2050, both sexes, < 5 years number (95% UI)
Oman	42790·1 (40907·0 - 44715·7)	54843·7 (50616·0 - 59467·2)	41353·5 (23333·9 - 53580·2)	36813·8 (19050·2 - 50180·4)
Palestine	71295·3 (65808·9 - 76892·9)	52310·3 (47102·6 - 58364·6)	41471·3 (18128·1 - 62624·7)	24985·1* (9588·4 - 49924·9)
Qatar	12519·4 (11145·1 - 13860·4)	15397·9 (13612·0 - 17240·1)	14364·4 (6769·3 - 21042·9)	17073·8 (6995·4 - 28029·4)
Saudi Arabia	240890·5 (217195·8 - 266316·1)	201968·7 (178711·0 - 227897·8)	136363·6 (62751·5 - 195013·9)	71086·2* (26038·6 - 124164·1)
Sudan	1951248·9 (1866447·8 - 2044881·3)	1684818·9 (1597651·9 - 1774945·6)	1447777·8 (988130·8 - 1670117·6)	899283·9* (585802·7 - 1138986·5)
Syria	765665·8 (732104·8 - 798323·7)	282628·7* (266798·1 - 298810·7)	301165·5* (205034·0 - 365740·9)	196810·9* (124424·9 - 278552·6)
Tunisia	101196·8 (95064·4 - 108233·9)	83286·8 (75806·6 - 91191·1)	51031·8 (27214·2 - 71782·1)	22098·8* (10541·6 - 34715·2)
Türkiye	655486·8 (596709·1 - 717837·9)	428383·6 (378456·6 - 484663·0)	268704·2* (125289·4 - 389855·2)	107468·6* (46365·8 - 182159·6)
United Arab Emirates	62698·2 (56471·8 - 68943·2)	56965·6 (50906·8 - 62989·3)	62977·8 (31388·6 - 84229·5)	77743·2 (34928·6 - 120221·5)
Yemen	2305159·2 (2205198·3 - 2413789·4)	2054237·5 (1939696·5 - 2191149·1)	1955870·9 (1451773·1 - 2176786·1)	1201272·5 (687792·0 - 1601644·1)
**South Asia**	**74216048·6 (73625772·2 - 74834416·5)**	**56016970·9 (55388769·7 - 56690677·7)**	**42270839·2 (27450702·7 - 48101811·8)**	**17764802·2* (11183347·7 - 22023447·3)**
Bangladesh	6645517·9 (6441773·7 - 6894857·1)	4357956·6 (4142640·9 - 4595881·9)	2862217·4* (1763575·3 - 3627535·4)	924391·4* (573469·8 - 1181590·7)
Bhutan	19702·3 (18431·0 - 21103·9)	13162·3 (11918·3 - 14257·8)	8430·9* (4789·4 - 11215·1)	2095·7* (1039·0 - 2956·6)
India	54980962·7 (54598692·0 - 55378954·0)	39801668·5 (39409350·1 - 40246225·8)	30392937·8 (19073466·5 - 34660599·7)	12250034·7* (7620716·7 - 16459918·6)
Nepal	1225767·0 (1146985·9 - 1315387·8)	1014768·2 (951212·6 - 1090397·6)	634725·2 (387225·9 - 832784·4)	146904·1* (68945·5 - 221808·8)
Pakistan	11236197·4 (10872008·2 - 11589715·6)	10753664·7 (10314052·4 - 11237886·6)	8372527·9 (5269913·5 - 9810476·4)	4441376·2* (2556793·6 - 5841150·9)
**Southeast Asia, east Asia, and Oceania**	**25908519·1 (25017205·3 - 26825623·6)**	**22302746·5 (21486085·4 - 23156778·5)**	**15803171·1 (10169156·2 - 19254552·3)**	**9053880·5* (5388899·7 - 12373150·1)**
East Asia	7470584·5 (6613861·4 - 8358456·9)	6590059·6 (5797804·2 - 7395759·7)	3137995·9* (1619135·1 - 4343549·8)	1538822·5* (622895·1 - 2505494·3)
China	6969581·9 (6113875·7 - 7852229·6)	6231335·7 (5435540·6 - 7036614·0)	2891749·9* (1458041·0 - 4055941·8)	1420752·9* (557988·4 - 2358982·3)
North Korea	473369·6 (455602·2 - 491410·0)	314341·5 (295143·2 - 334478·7)	229919·9* (140700·0 - 284209·6)	107921·7* (64291·3 - 150955·4)
Taiwan (province of China)	25790·4 (23194·6 - 28485·0)	21998·0 (19642·6 - 24612·3)	16326·1 (7543·0 - 23947·5)	10147·9* (4822·1 - 15132·4)

*mean < the stunting global nutrition target (50% reduction from 2012)

Table S5. Global, regional, and country number of children with stunting, 2012, 2021, and projected for 2030 and 2050

	Stunting, 2012, both sexes, < 5 years number (95% UI)	Stunting, 2021, both sexes, < 5 years number (95% UI)	Stunting, 2030, both sexes, < 5 years number (95% UI)	Stunting, 2050, both sexes, < 5 years number (95% UI)
Oceania	613802·5 (595625·9 - 634881·2)	735539·6 (705398·2 - 772714·5)	827126·0 (618697·9 - 930762·4)	897867·0 (602172·9 - 1079264·5)
American Samoa	688·7 (604·3 - 771·8)	310·2* (270·7 - 356·6)	343·1* (174·6 - 468·0)	265·1* (130·8 - 376·8)
Cook Islands	115·8 (101·1 - 132·4)	67·0 (57·6 - 77·5)	57·3* (22·1 - 83·5)	32·7* (12·3 - 50·9)
Federated States of Micronesia	1782·2 (1592·7 - 1986·2)	1153·9 (1000·5 - 1302·2)	937·4 (569·2 - 1234·7)	613·0* (340·5 - 859·7)
Fiji	6661·6 (5836·0 - 7650·7)	6189·1 (5574·8 - 6871·2)	4324·4 (2236·7 - 6206·9)	3292·7* (1642·2 - 4990·9)
Guam	1192·3 (1039·5 - 1357·5)	782·1 (679·5 - 899·7)	624·2 (257·5 - 912·0)	473·7* (188·5 - 727·6)
Kiribati	2033·0 (1802·5 - 2272·8)	1973·6 (1767·2 - 2195·0)	1597·6 (1016·9 - 2029·7)	1061·8 (617·6 - 1391·8)
Marshall Islands	897·6 (796·1 - 1004·0)	661·9 (577·2 - 741·6)	581·4 (342·1 - 766·8)	402·9* (223·5 - 561·7)
Nauru	289·8 (268·4 - 309·0)	211·6 (192·7 - 231·3)	194·0 (92·2 - 271·6)	155·4 (58·9 - 253·1)
Niue	13·1 (11·6 - 14·9)	9·0 (7·9 - 10·4)	7·6 (3·2 - 11·1)	5·2* (2·1 - 8·0)
Northern Mariana Islands	384·2 (333·9 - 435·9)	198·3 (171·3 - 227·3)	173·5* (73·2 - 255·6)	133·1* (53·1 - 200·8)
Palau	137·9 (121·3 - 156·2)	73·7 (63·7 - 84·7)	63·6* (29·4 - 92·2)	45·6* (19·6 - 69·0)
Papua New Guinea	527278·3 (510173·4 - 546656·9)	651208·2 (622946·6 - 686492·3)	746063·5 (558596·2 - 837648·2)	824801·0 (554447·1 - 991544·9)
Samoa	1285·2 (1137·4 - 1444·0)	1279·3 (1110·6 - 1457·1)	1198·4 (563·7 - 1714·8)	1391·7 (633·1 - 2255·7)
Solomon Islands	24919·1 (24138·2 - 25717·8)	24122·6 (22929·2 - 25312·4)	22452·8 (15036·8 - 27602·7)	14201·9 (7688·6 - 20163·3)
Tokelau	12·7 (11·2 - 14·3)	8·2 (7·2 - 9·4)	6·7 (3·3 - 9·7)	3·9* (1·8 - 6·4)
Tonga	864·9 (751·2 - 986·3)	460·7 (396·7 - 540·0)	360·6* (178·3 - 508·5)	353·6* (165·8 - 557·2)
Tuvalu	103·0 (91·1 - 116·4)	86·1 (74·9 - 98·6)	82·9 (47·2 - 108·4)	64·1 (33·6 - 90·1)
Vanuatu	9652·8 (9204·8 - 10079·5)	9281·9 (8659·7 - 9882·4)	8990·8 (5969·1 - 10836·7)	8162·5 (5287·4 - 10612·0)
Southeast Asia	18086746·3 (17898716·8 - 18268929·7)	14758611·6 (14518180·2 - 15013990·1)	11865786·1 (7900175·4 - 14255473·4)	6647708·6* (3940621·3 - 8999262·7)
Cambodia	649323·2 (624623·4 - 677766·2)	547985·2 (518674·6 - 579454·8)	404497·2 (251714·7 - 500996·2)	158137·1* (80858·9 - 225201·0)

*mean < the stunting global nutrition target (50% reduction from 2012)

Table S5. Global, regional, and country number of children with stunting, 2012, 2021, and projected for 2030 and 2050

	Stunting, 2012, both sexes, < 5 years number (95% UI)	Stunting, 2021, both sexes, < 5 years number (95% UI)	Stunting, 2030, both sexes, < 5 years number (95% UI)	Stunting, 2050, both sexes, < 5 years number (95% UI)
Indonesia	8496080·8 (8426130·3 - 8561261·2)	6985312·4 (6895612·7 - 7080274·1)	5739255·4 (3767710·9 - 6794345·6)	3221695·9* (1807559·9 - 4463441·9)
Laos	325856·5 (307418·1 - 348634·0)	272564·2 (255916·2 - 291617·8)	218802·6 (147428·5 - 263050·7)	109915·1* (67982·1 - 145951·9)
Malaysia	484458·5 (459409·2 - 507437·6)	443279·1 (416179·4 - 472668·5)	357153·2 (230013·1 - 438715·6)	200566·6* (115652·1 - 261218·5)
Maldives	6954·7 (6491·3 - 7463·5)	5423·6 (4907·6 - 5922·8)	4050·4 (2397·9 - 5355·7)	2159·6* (999·9 - 3548·3)
Mauritius	13603·8 (12454·2 - 14807·0)	10684·8 (9767·4 - 11663·3)	9019·8 (4897·3 - 11912·1)	4359·2* (2204·9 - 6036·5)
Myanmar	1586501·2 (1522897·0 - 1666532·3)	1281180·8 (1208120·4 - 1357679·1)	970220·1 (599772·4 - 1204229·5)	400244·0* (224484·3 - 544680·6)
Philippines	3556292·9 (3447802·1 - 3678418·7)	2879018·2 (2735787·6 - 3031790·2)	2505105·5 (1596238·8 - 3145454·1)	1752923·9* (978027·5 - 2468081·6)
Seychelles	800·6 (703·2 - 895·9)	734·8 (635·1 - 839·1)	688·7 (349·5 - 970·2)	590·3 (296·8 - 864·4)
Sri Lanka	300659·0 (284515·9 - 316764·9)	236809·5 (218755·9 - 255594·3)	162860·1 (80593·5 - 235199·0)	64180·8* (26521·9 - 120080·2)
Thailand	515571·2 (484198·5 - 549077·1)	356858·2 (328849·6 - 387170·7)	281107·1 (159694·0 - 368462·7)	121184·0* (62994·0 - 173273·8)
Timor-Leste	78228·8 (75391·1 - 81567·2)	83166·3 (79864·8 - 87245·7)	83157·0 (60060·0 - 93739·2)	54401·1 (31548·2 - 67207·2)
Viet Nam	2097849·2 (2028102·1 - 2170662·5)	1760528·1 (1651251·1 - 1874378·5)	1114323·6 (680207·4 - 1463930·5)	548641·9* (264972·4 - 1009233·8)
**Sub-Saharan Africa**	**55400002·1 (54893222·3 - 55938086·9)**	**53983794·9 (53332842·2 - 54637315·6)**	**53586050·5 (37024578·2 - 60783231·3)**	**44039952·7 (28561283·2 - 50956345·3)**
Central sub-Saharan Africa	7347950·6 (7142514·1 - 7595131·2)	7227399·2 (6988818·8 - 7467615·3)	6883910·2 (4866414·6 - 7710702·3)	5396028·8 (3344078·5 - 6660031·5)
Angola	1789714·0 (1686380·5 - 1921455·7)	1848677·9 (1752725·1 - 1953953·8)	1667147·8 (1110804·6 - 1981688·2)	1098601·9 (583070·2 - 1561352·9)
Central African Republic	320120·1 (307498·3 - 336038·2)	312241·2 (298471·2 - 329269·4)	310414·7 (236458·2 - 337025·5)	224544·1 (157365·9 - 258497·0)
Congo (Brazzaville)	157868·7 (150983·9 - 165323·9)	123629·3 (114236·2 - 132812·3)	106958·0 (67373·5 - 134172·1)	74322·3* (40998·1 - 106534·7)
DR Congo	4988785·8 (4825295·9 - 5192700·4)	4865639·7 (4670866·1 - 5085030·5)	4733028·3 (3423146·0 - 5267662·4)	3953080·0 (2472706·4 - 5163536·5)
Equatorial Guinea	46419·9 (44922·5 - 48019·8)	40860·6 (37838·6 - 43707·9)	38082·1 (23962·9 - 51183·0)	28129·9 (13434·9 - 45136·7)
Gabon	40242·7 (37433·7 - 43168·3)	32689·6 (29724·5 - 35888·6)	28279·4 (16265·3 - 37675·8)	17350·6* (8193·5 - 28073·3)
Eastern sub-Saharan Africa	21799079·7 (21508727·2 - 22090363·3)	20398484·1 (20075405·0 - 20697082·4)	19903053·3 (13828659·2 - 22723096·5)	14180556·9 (9431689·5 - 17362063·5)

*mean < the stunting global nutrition target (50% reduction from 2012)

Table S5. Global, regional, and country number of children with stunting, 2012, 2021, and projected for 2030 and 2050

	Stunting, 2012, both sexes, < 5 years number (95% UI)	Stunting, 2021, both sexes, < 5 years number (95% UI)	Stunting, 2030, both sexes, < 5 years number (95% UI)	Stunting, 2050, both sexes, < 5 years number (95% UI)
Burundi	881140·9 (846390·2 - 927978·1)	1022455·5 (977190·5 - 1087570·4)	1140477·5 (996718·8 - 1193235·0)	1189960·0 (970857·5 - 1275450·4)
Comoros	29087·4 (27688·0 - 30590·2)	24470·3 (23110·0 - 25832·8)	19317·1 (12341·2 - 23303·2)	8837·4* (5403·7 - 11745·3)
Djibouti	42090·5 (40640·4 - 43562·4)	41653·3 (39481·8 - 43867·3)	30641·2 (18687·1 - 37266·7)	22532·2 (13488·7 - 29712·8)
Eritrea	355716·6 (333429·3 - 381506·0)	346314·5 (324081·4 - 373164·9)	334859·2 (243544·3 - 373745·5)	228403·0 (133285·1 - 280943·0)
Ethiopia	5743229·6 (5591865·9 - 5908232·0)	5324865·3 (5176182·3 - 5466389·7)	5419721·9 (3539932·0 - 6214506·0)	3236688·0 (1875170·3 - 4560702·3)
Kenya	1966121·8 (1906099·9 - 2029440·6)	1633293·8 (1581359·1 - 1685616·0)	1438663·0 (954950·6 - 1712806·6)	928193·9* (610585·1 - 1160369·2)
Madagascar	1822953·9 (1721203·4 - 1940467·4)	1723442·0 (1602719·3 - 1852219·8)	1804487·5 (1415650·6 - 1948483·3)	1643538·5 (1195283·5 - 1830742·3)
Malawi	954010·2 (917145·1 - 998737·7)	779037·1 (737258·7 - 817271·8)	690326·9 (443896·1 - 817208·4)	363438·4* (214531·5 - 497492·7)
Mozambique	1735024·0 (1650004·6 - 1833994·4)	1692581·4 (1604382·9 - 1793244·0)	1475675·2 (978183·1 - 1828062·2)	850874·8* (483963·1 - 1137446·1)
Rwanda	633228·3 (603563·1 - 669759·9)	579295·0 (549647·2 - 613740·1)	541772·4 (355838·4 - 650781·8)	321448·2 (175805·8 - 473003·0)
Somalia	839290·6 (796755·3 - 883662·0)	1082260·7 (1004049·7 - 1162975·0)	1236879·1 (844976·5 - 1451833·5)	1412748·6 (941749·5 - 1750798·3)
South Sudan	540463·9 (521067·9 - 558908·8)	445589·2 (420626·5 - 469378·7)	537155·7 (307636·4 - 695437·8)	473483·2 (86383·2 - 779620·6)
Uganda	2110104·5 (2025458·3 - 2204280·8)	1963894·7 (1859358·9 - 2070249·9)	1813345·6 (1125263·7 - 2286109·0)	1123347·5 (614848·8 - 1549586·0)
Tanzania	2999488·3 (2884014·4 - 3148066·4)	2793017·9 (2661060·7 - 2924980·4)	2582198·1 (1675563·5 - 3131944·0)	1756458·2 (990194·9 - 2534866·3)
Zambia	1128884·4 (1091243·8 - 1174561·6)	921303·3 (875383·8 - 963074·6)	821621·5 (513839·0 - 995902·7)	609268·8 (357836·1 - 898171·2)
Southern sub-Saharan Africa	2212603·5 (2172058·2 - 2254911·4)	1823226·1 (1777817·1 - 1867824·9)	1497456·4 (955405·9 - 1876471·2)	954061·4* (529153·5 - 1442082·1)
Botswana	68600·2 (65448·8 - 72003·8)	61905·5 (57742·4 - 66031·6)	53097·5 (34365·3 - 67009·5)	35855·7 (21913·2 - 51139·3)
Eswatini	45941·4 (43706·4 - 48533·5)	35009·8 (32589·7 - 37486·9)	28081·5 (17788·4 - 34649·6)	16933·0* (9756·5 - 22949·2)
Lesotho	84410·7 (79651·3 - 89822·7)	66759·9 (63246·9 - 70549·7)	56680·2 (37457·9 - 67106·0)	29854·3* (18320·9 - 42073·5)
Namibia	71823·0 (68372·6 - 75358·6)	56719·2 (52586·7 - 60901·8)	50084·1 (30688·1 - 65685·8)	34831·7* (16510·8 - 52397·1)
South Africa	1287153·2 (1258154·0 - 1317751·5)	1045734·3 (1012379·5 - 1077207·0)	814895·7 (525187·3 - 1015767·7)	513957·9* (282455·9 - 758293·9)

*mean < the stunting global nutrition target (50% reduction from 2012)

Table S5. Global, regional, and country number of children with stunting, 2012, 2021, and projected for 2030 and 2050

	Stunting, 2012, both sexes, < 5 years number (95% UI)	Stunting, 2021, both sexes, < 5 years number (95% UI)	Stunting, 2030, both sexes, < 5 years number (95% UI)	Stunting, 2050, both sexes, < 5 years number (95% UI)
Zimbabwe	654873·2 (626117·7 - 686138·6)	560524·8 (526789·5 - 594815·5)	494617·4 (319393·9 - 631108·4)	322628·8* (162245·5 - 525066·5)
Western sub-Saharan Africa	24000328·1 (23662812·4 - 24372980·2)	24494513·3 (23938870·6 - 25069571·0)	25301632·8 (17431511·6 - 28649474·0)	23508363·8 (15003497·5 - 27458272·2)
Benin	758180·9 (733975·1 - 784063·2)	788377·8 (751800·0 - 827231·5)	913985·8 (640586·3 - 1020901·3)	931494·6 (594370·2 - 1162138·3)
Burkina Faso	1068610·2 (1038506·7 - 1102427·4)	1118364·9 (1061256·2 - 1174862·6)	1111718·4 (707189·8 - 1343759·2)	867418·5 (433672·3 - 1223974·3)
Cabo Verde	7953·7 (7196·7 - 8776·9)	5529·7 (4900·5 - 6142·9)	3221·5* (1501·5 - 4708·9)	909·4* (452·4 - 1363·5)
Cameroon	1365907·8 (1307934·3 - 1429274·5)	1336282·8 (1256084·8 - 1408054·8)	1129203·8 (735170·9 - 1400463·1)	731053·8 (407437·5 - 1050972·5)
Chad	982227·6 (936804·2 - 1040238·6)	1186717·5 (1109833·6 - 1287955·2)	1533675·1 (1124594·1 - 1664563·7)	2113053·6 (1380457·8 - 2467128·6)
Côte d'Ivoire	1096887·8 (1051793·5 - 1147397·2)	965103·3 (904750·0 - 1030188·4)	878136·2 (552410·2 - 1140876·4)	569467·1 (227026·9 - 1008572·0)
Ghana	937146·0 (894939·5 - 978849·6)	812261·5 (747595·1 - 882166·7)	591481·1 (323431·5 - 756958·2)	299304·0* (133712·8 - 496489·4)
Guinea	650903·5 (624148·1 - 686458·2)	667412·0 (635807·9 - 707400·6)	686624·5 (455625·1 - 804840·7)	552606·1 (295055·3 - 759221·6)
Guinea-Bissau	94204·6 (90690·7 - 97694·8)	90832·9 (85723·3 - 96275·1)	83413·0 (55843·3 - 103974·4)	49035·3 (21730·2 - 74486·2)
Liberia	249259·3 (235418·0 - 265626·5)	204460·0 (191355·0 - 219941·3)	173370·4 (110668·2 - 209761·5)	95989·2* (53725·0 - 134621·9)
Mali	996931·1 (964200·6 - 1031617·5)	1127551·2 (1069412·8 - 1190774·4)	1210372·0 (813993·8 - 1467791·4)	1126572·4 (634558·3 - 1513990·0)
Mauritania	145849·4 (141594·0 - 150279·4)	125901·6 (117034·8 - 134648·4)	95279·0 (55373·6 - 120908·6)	44500·8* (18837·3 - 63314·5)
Niger	1704659·1 (1610908·7 - 1807288·0)	2157271·4 (2033399·1 - 2281924·0)	2736305·6 (1941632·6 - 3094295·4)	3958451·0 (2498852·4 - 4814599·6)
Nigeria	12667237·8 (12359753·1 - 12990306·9)	12743857·4 (12220740·9 - 13259630·4)	13220234·2 (9100129·9 - 14817690·8)	11658956·4 (7361767·4 - 14405692·2)
São Tomé and Príncipe	7576·3 (7207·6 - 7921·4)	4938·3 (4605·7 - 5286·0)	3406·7* (2037·3 - 4406·9)	1248·6* (694·8 - 1814·9)
Senegal	464122·2 (443540·4 - 484559·2)	364194·8 (337854·2 - 391161·4)	300311·6 (177345·1 - 398683·6)	138813·7* (54097·1 - 218318·4)
Sierra Leone	416274·3 (401819·7 - 433486·1)	380460·7 (360629·6 - 399231·9)	386325·2 (251964·8 - 442722·1)	256550·5 (154018·6 - 360120·9)
The Gambia	75008·3 (71627·6 - 78450·5)	63648·1 (59209·9 - 68658·8)	56794·5 (37098·8 - 70053·3)	29003·5* (14220·6 - 41753·7)
Togo	291062·2 (281048·6 - 301243·8)	255235·1 (237712·4 - 271500·8)	187420·5 (105254·9 - 250576·4)	83606·6* (31981·5 - 146511·9)

*mean < the stunting global nutrition target (50% reduction from 2012)
