# Supplementary material for: Global, regional, and national progress towards the 2030 global nutrition targets and forecasts to 2050: a systematic analysis for the Global Burden of Disease Study 2021
Source: Lancet. 2024 Dec 21;404(10471):2543–83. doi: 10.1016/S0140-6736(24)01821-X (PMC11703702; doi:10.1016/S0140-6736(24)01821-X)
Supplement: Supplementary appendix 3 [file mmc3.pdf]

THE LANCET

Supplementary appendix 3

This appendix formed part of the original submission and has been peer reviewed. We post it as supplied by the authors.

Supplement to: Global Nutrition Target Collaborators. Global, regional, and national progress towards the 2030 global nutrition targets and forecasts to 2050: a systematic analysis for the Global Burden of Disease Study 2021. *Lancet* 2024; published online Dec 9. https://doi.org/10.1016/S0140-6736(24)01821-X.

Appendix 3: Authorship appendix to "Global, regional, and national progress towards the 2030 global nutrition targets and forecasts to 2050: a systematic analysis for the Global Burden of Disease Study 2021"

This appendix provides further authorship detail for "Global, regional, and national progress towards the 2030 global nutrition targets and forecasts to 2050: a systematic analysis for the Global Burden of Disease Study 2021"

Table of Contents

GBD 2021 Global Nutrition Targets Collaborators.......... 2

Affiliations 5

Authors' Contributions.......... 23

- Managing the overall research enterprise.......... 23
- Writing the first draft of the manuscript 23
- Primary responsibility for applying analytical methods to produce estimates 24
- Primary responsibility for seeking, cataloguing, extracting, or cleaning data; designing or coding figures and tables.......... 24
- Providing data or critical feedback on data sources.......... 24
- Developing methods or computational machinery 25
- Providing critical feedback on methods or results 26
- Drafting the work or revising it critically for important intellectual content 29
- Managing the estimation or publications process.......... 31

GBD 2021 Global Nutrition Targets Collaborators

Michael Benjamin Arndt, Yohannes Habtegiorgis Abate, Mohsen Abbasi-Kangevari, Samar Abd ElHafeez, Michael Abdelmasseh, Sherief Abd-Elsalam, Deldar Morad Abdulah, Rizwan Suliankatchi Abdulkader, Hassan Abidi, Olumide Abiodun, Richard Gyan Aboagye, Hassan Abolhassani, Yonas Derso Abtew, Eman Abu-Gharbieh, Niveen ME Abu-Rmeileh, Juan Manuel Acuna, Kidist Adamu, Denberu Eshetie Adane, Isaac Yeboah Addo, Daniel Adedayo Adeyinka, Qorinah Estiningtyas Sakilah Adnani, Aanuoluwapo Adeyimika Afolabi, Fatemeh Afrashteh, Saira Afzal, Antonella Agodi, Bright Opoku Ahinkorah, Aqeel Ahmad, Sajjad Ahmad, Tauseef Ahmad, Ali Ahmadi, Ali Ahmed, Luai A A Ahmed, Marjan Ajami, Budi Aji, Hossein Akbarialiabad, Maxwell Akonde, Hanadi Al Hamad, Yazan Al Thaher, Ziyad Al-Aly, Khalid F Alhabib, Robert Kaba Alhassan, Beriwan Abdulqadir Ali, Syed Shujait Ali, Yousef Alimohamadi, Syed Mohamed Aljunid, Hesham M Al-Mekhlafi, Sami Almustanyir, Mahmoud A Alomari, Alaa B Al-Tammemi, Khalid A Altirkawi, Nelson Alvis-Guzman, Nelson J Alvis-Zakzuk, Edward Kwabena Ameyaw, Tarek Tawfik Amin, Sohrab Amiri, Hubert Amu, Dickson A Amugsi, Tadele Fentabel Fentabil Anagaw, Robert Ancuceanu, Dhanalakshmi Angappan, Alireza Ansari-Moghaddam, Ernoiz Antriyandarti, Davood Anvari, Anayochukwu Edward Anyasodor, Jalal Arabloo, Aleksandr Y Aravkin, Hany Ariffin, Timur Aripov, Mesay Arkew, Benedetta Armocida, Ashokan Arumugam, Ni Ketut Aryastami, Malke Asaad, Zatollah Asemi, Mulu Tiruneh Asemu, Mohammad Asghari-Jafarabadi, Thomas Astell-Burt, Seyyed Shamsadin Athari, Gamechu Hunde Atomsa, Prince Atorkey, Maha Moh'd Wahbi Atout, Avinash Aujayeb, Mamaru Ayenew Awoke, Sina Azadnajafabad, Rui M S Azevedo, Darshan B B, Ashish D Badiye, Nayereh Baghcheghi, Nasser Bagheri, Sara Bagherieh, Atif Amin Baig, Jennifer L Baker, Madhan Balasubramanian, Ovidiu Constantin Baltatu, Maciej Banach, Palash Chandra Banik, Martina Barchitta, Till Winfried Bärnighausen, Ronald D Barr, Amadou Barrow, Lingkan Barua, Azadeh Bashiri, Pritish Baskaran, Saurav Basu, Alehegn Bekele, Sefealem Assefa Belay, Uzma Iqbal Belgaumi, Shelly L Bell, Luis Belo, Derrick A Bennett, Isabela M Bensenor, Girma Beressa, Amiel Nazer C Bermudez, Habtamu B Beyene, Akshaya Srikanth Bhagavathula, Nikha Bhardwaj, Pankaj Bhardwaj, Sonu Bhaskar, Natalia V Bhattacharjee, Zulfiqar A Bhutta, Saeid Bitaraf, Virginia Bodolica, Milad Bonakdar Hashemi, Dejana Braithwaite, Muhammad Hammad Butt, Zahid A Butt, Daniela Calina, Luis Alberto Cámera, Luciana Aparecida Campos, Chao Cao, Rosario Cárdenas, Márcia Carvalho, Carlos A Castañeda-Orjuela, Alberico L Catapano, Maria Sofia Cattaruzza, Francieli Cembranel, Ester Cerin, Joshua Chadwick, Julian Chalek, Eeshwar K Chandrasekar, Jaykaran Charan, Vijay Kumar Chattu, Kirti Chauhan, Ju-Huei Chien, Abdulaal Chitheer, Sonali Gajanan Choudhari, Enayet Karim Chowdhury, Dinh-Toi Chu, Isaac Sunday Chukwu, Sheng-Chia Chung, Rafael M Claro, Alyssa Columbus, Samuele Cortese, Natalia Cruz-Martins, Bashir Dabo, Omid Dadras, Xiaochen Dai, Emanuele D'Amico, Lalit Dandona, Rakhi Dandona, Isaac Darban, Gary L Darmstadt, Aso Mohammad Darwesh, Amira Hamed Darwish, Jai K Das, Saswati Das, Kairat Davletov, Fernando Pio De la Hoz, Aklilu Tamire Debele, Dessalegn Demeke, Solomon Demissie, Edgar Denova-Gutiérrez, Hardik Dineshbhai Desai, Abebaw Alemayehu Desta, Samath Dhamminda Dharmaratne, Meghnath Dhimal, Diana Dias da Silva, Daniel Diaz, Mengistie Diress, Shirin Djalalinia, Saeid Doaei, Deepa Dongarwar, Haneil Larson Dsouza, Sareh Edalati, Hisham Atan Edinur, Michael Ekholuenetale, Temitope Cyrus Ekundayo, Iffat Elbarazi, Islam Y Elgendy, Muhammed Elhadi, Omar Abdelsadek Abdou Elmeligy, Habitu Birhan Eshetu, Juan Espinosa-Montero, Habtamu Esubalew, Farshid Etaee, Werku Etafa, Adeniyi Francis Fagbamigbe, Ildar Ravisovich Fakhradiyev, Luca Falzone, Carla Sofia e Sá Farinha, Sam Farmer, Abidemi Omolara Fasanmi, Ali Fatehizadeh, Valery L Feigin, Alireza Feizkhah, Xiaoqi Feng, Pietro Ferrara, Getahun Fetensa, Florian Fischer, Ryan Fitzgerald, David Flood, Nataliya A Foigt, Morenike Oluwatoyin Folayan, Kayode Raphael Fowobaje, Richard Charles Franklin, Takeshi Fukumoto, Muktar A Gadanya, Abhay

Motiramji Gaidhane, Santosh Gaihre, Emmanuela Gakidou, Yaseen Galali, Nasrin Galehdar, William M Gardner, Priyanka Garg, Teferi Gebru Gebremeskel, Urge Gerema, Lemma Getacher, Motuma Erena Getachew, Solomon Getawa, Kazem Ghaffari, Seyyed-Hadi Ghamari, Mohammad Ghasemi Nour, Fariba Ghassemi, Nermin Ghith, Maryam Gholamalizadeh, Ali Gholami, Ali Gholamrezanezhad, Sherief Ghozy, Paramjit Singh Gill, Tiffany K Gill, James C Glasbey, Mahaveer Golechha, Pouya Goleij, Davide Golinelli, Houman Goudarzi, Michal Grivna, Habtamu Alganeh Guadie, Mohammed Ibrahim Mohialdeen Gubari, Temesgen Worku Gudayu, Avirup Guha, Damitha Asanga Gunawardane, Anish Kumar Gupta, Bhawna Gupta, Rahul Gupta, Sapna Gupta, Veer Bala Gupta, Vivek Kumar Gupta, Hailey Hagins, Arvin Haj-Mirzaian, Alexis J Handal, Asif Hanif, Graeme J Hankey, Harapan Harapan, Arief Hargono, Josep Maria Haro, Ahmed I Hasaballah, Md Mehedi Hasan, Hamidreza Hasani, Abdiwahab Hashi, Soheil Hassanipour, Rasmus J Havmoeller, Simon I Hay, Khezar Hayat, Jiawei He, Mahsa Heidari-Foroozan, Claudiu Herteliu, Kamran Hessami, Demisu Zenbaba Heyi, Kamal Hezam, Yuta Hiraike, Ramesh Holla, Praveen Hoogar, Sheikh Jamal Hossain, Mehdi Hosseinzadeh, Mihaela Hostiuc, Sorin Hostiuc, Soodabeh Hoveidamanesh, Junjie Huang, Kyle Matthew Humphrey, Salman Hussain, Foziya Mohammed Hussien, Bing-Fang Hwang, Licia Iacoviello, Pulwasha Maria Iftikhar, Olayinka Stephen Ilesanmi, Irena M Ilic, Milena D Ilic, Mustapha Immurana, Leeberk Raja Inbaraj, Farideh Iravanpour, Sheikh Mohammed Shariful Islam, Farhad Islami, Nahlah Elkudssiah Ismail, Hiroyasu Iso, Gaetano Isola, Masao Iwagami, Chidozie Declan Iwu, Linda Merin J, Louis Jacob, Haitham Jahrami, Mihajlo Jakovljevic, Elham Jamshidi, Manthan Dilipkumar Janodia, Krishnamurthy Jayanna, Sathish Kumar Jayapal, Shubha Jayaram, Rime Jebai, Alelign Tasew Jema, Bijay Mukesh Jeswani, Jost B Jonas, Abel Joseph, Nitin Joseph, Charity Ehimwenma Joshua, Jacek Jerzy Jozwiak, Mikk Jürisson, Billingsley Kaambwa, Ali Kabir, Zubair Kabir, Vidya Kadashetti, Vineet Kumar Kamal, Bhushan Dattatray Kamble, Himal Kandel, Neeti Kapoor, Ibraheem M Karaye, Patrick DMC Katoto, Joonas H Kauppila, Harkiran Kaur, Gbenga A Kayode, Worku Misganaw Kebede, Jemal Yusuf Kebira, Tibebeselassie S Keflie, Jessica A Kerr, Mohammad Keykhaei, Yousef Saleh Khader, Himanshu Khajuria, Nauman Khalid, Mohammad Khammarnia, M Nuruzzaman Khan, Moien AB Khan, Taimoor Khan, Yusra H Khan, Javad Khanali, Shaghayegh Khanmohammadi, Khaled Khatab, Moawiah Mohammad Khatatbeh, Sorour Khateri, Mahalaqua Nazli Khatib, Hamid Reza Khayat Kashani, Jagdish Khubchandani, Zemene Demelash Kifle, Gyu Ri Kim, Ruth W Kimokoti, Adnan Kisa, Sezer Kisa, Farzad Kompani, Shivakumar KM Marulasiddaiah Kondlahalli, Hamid Reza Koohestani, Oleksii Korzh, Sindhura Lakshmi Koulmane Laxminarayana, Ai Koyanagi, Kewal Krishan, Vijay Krishnamoorthy, Barthelemy Kuate Defo, Burcu Kucuk Bicer, Mohammed Kuddus, G Anil Kumar, Manasi Kumar, Nithin Kumar, Almagul Kurmanova, Om P Kurmi, Dian Kusuma, Carlo La Vecchia, Ben Lacey, Dharmesh Kumar Lal, Anders O Larsson, Kamaluddin Latief, Caterina Ledda, Paul H Lee, Sang-woong Lee, Wei-Chen Lee, Yo Han Lee, Jacopo Lenzi, Ming-Chieh Li, Wei Li, Virendra S Ligade, Stephen S Lim, Paulina A Lindstedt, Chun-Han Lo, Justin Lo, Rakesh Lodha, Arianna Maever Loreche, László Lorenzovici, Stefan Lorkowski, Farzan Madadizadeh, Áurea M Madureira-Carvalho, Preetam Bhalchandra Mahajan, Konstantinos Christos Makris, Elaheh Malakan Rad, Ahmad Azam Malik, Tauqeer Hussain Mallhi, Deborah Carvalho Malta, Helena Manguerra, Abdoljalal Marjani, Santi Martini, Miquel Martorell, Awoke Masrie, Elezebeth Mathews, Andrea Maugeri, Maryam Mazaheri, Rishi P Mediratta, Man Mohan Mehndiratta, Yohannes Adama Melaku, Walter Mendoza, Ritesh G Menezes, George A Mensah, Alexios-Fotios A Mentis, Tuomo J Meretoja, Tomislav Mestrovic, Tomasz Miazgowski, Ted R Miller, GK Mini, Mojgan Mirghafourvand, Andreea Mirica, Erkin M Mirrakhimov, Moonis Mirza, Sanjeev Misra, Prasanna Mithra, Karzan Abdulmuhsin Mohammad, Abdollah Mohammadian-Hafshejani, Shafiu Mohammed, Mohammad Mohseni, Ali H Mokdad, Lorenzo Monasta, Mohammad Ali Moni, Maryam Moradi, Yousef Moradi,

Shane Douglas Morrison, Vincent Mougin, Sumaira Mubarik, Ulrich Otto Mueller, Francesk Mulita, Daniel Munblit, Efren Murillo-Zamora, Christopher J L Murray, Ghulam Mustafa, Ahamarshan Jayaraman Nagarajan, Vinay Nangia, Sreenivas Narasimha Swamy, Zuhair S Natto, Muhammad Naveed, Biswa Prakash Nayak, Seyed Aria Nejadghaderi, Georges Nguefack-Tsague, Josephine W Ngunjiri, Phuong The Nguyen, QuynhAnh P Nguyen, Robina Khan Niazi, Chukwudi A Nnaji, Nurulamin M Noor, Jean Jacques Noubiap, Chisom Adaobi Nri-Ezedi, Dieta Nurrika, Vincent Ebuka Nwatah, Bogdan Oancea, Kehinde O Obamiro, Onome Bright Oghenetega, Ropo Ebenezer Ogunsakin, Hassan Okati-Aliabad, Akinkunmi Paul Okekunle, Daniel Micheal Okello, Osaretin Christabel Okonji, Andrew T Olagunju, Diriba Dereje Olana, Gláucia Maria Moraes Oliveira, Bolajoko Olubukunola Olusanya, Jacob Olusegun Olusanya, Sok King Ong, Doris V Ortega-Altamirano, Alberto Ortiz, Sergej M Ostojic, Adrian Otoiu, Abdu Oumer, Alicia Padron-Monedero, Jagadish Rao Padubidri, Adrian Pana, Songhomitra Panda-Jonas, Anamika Pandey, Seithikurippu R Pandi-Perumal, Paraskevi Papadopoulou, Shahina Pardhan, Maja Pasovic, Jay Patel, Aslam Ramjan Pathan, Deepak Paudel, Shrikant Pawar, Veincent Christian Filipino Pepito, Gavin Pereira, Marcos Pereira, Norberto Perico, Simone Perna, Ionela-Roxana Petcu, Fanny Emily Petermann-Rocha, Zahra Zahid Piracha, Nishad Plakkal, Naeimeh Pourtaheri, Amir Radfar, Venkatraman Radhakrishnan, Catalina Raggi, Pankaja Raghav, Fakher Rahim, Vafa Rahimi-Movaghar, Azizur Rahman, Md Mosfequr Rahman, Md Obaidur Rahman, Mosiur Rahman, Muhammad Aziz Rahman, Amir Masoud Rahmani, Vahid Rahmanian, Setyaningrum Rahmawaty, Rajesh Kumar Rai, Ivano Raimondo, Sathish Rajaa, Prashant Rajput, Pradhum Ram, Shakthi Kumaran Ramasamy, Sheena Ramazanu, Chythra R Rao, Indu Ramachandra Rao, Sowmya J Rao, Drona Prakash Rasali, Ahmed Mustafa Rashid, Mohammad-Mahdi Rashidi, Zubair Ahmed Ratan, Salman Rawaf, Lal Rawal, Elrashdy M Moustafa Mohamed Redwan, Giuseppe Remuzzi, Kannan RR Rengasamy, Andre M N Renzaho, Malihe Rezaee, Nazila Rezaei, Mohsen Rezaeian, Abanoub Riad, Jennifer Rickard, Alina Rodriguez, Jefferson Antonio Buendia Rodriguez, Leonardo Roever, Peter Rohloff, Bedanta Roy, Godfrey M Rwegerera, Chandan S N, Aly M A Saad, Maha Mohamed Saber-Ayad, Siamak Sabour, Mamta Sachdeva Dhingra, Basema Ahmad Saddik, Erfan Sadeghi, Malihe Sadeghi, Saeid Sadeghian, Umar Saeed, Sahar Saeedi Moghaddam, Sher Zaman Safi, Fatemeh Saheb Sharif-Askari, Amirhossein Sahebkar, Harihar Sahoo, Soumya Swaroop Sahoo, Mirza Rizwan Sajid, Marwa Rashad Salem, Abdallah M Samy, Juan Sanabria, Rama Krishna Sanjeev, Senthilkumar Sankararaman, Itamar S Santos, Milena M Santric-Milicevic, Sivan Yegnanarayana Iyer Saraswathy, Saman Sargazi, Yaser Sarikhani, Maheswar Satpathy, Monika Sawhney, Ganesh Kumar Saya, Abu Sayeed, Nikolaos Scarmeas, Markus P Schlaich, Rachel D Schneider, Aletta Elisabeth Schutte, Subramanian Senthilkumaran, Sadaf G Sepanlou, Dragos Serban, Allen Seylani, Mahan Shafie, Pritik A Shah, Ataollah Shahbandi, Masood Ali Shaikh, Adisu Tafari T Shama, Mehran Shams-Beyranvand, Mohd Shanawaz, Mequannent Melaku Sharew, Pavanchand H Shetty, Rahman Shiri, Velizar Shivarov, Seyed Afshin Shorofi, Kerem Shuval, Migbar Mekonnen Sibhat, Luís Manuel Lopes Rodrigues Silva, Jasvinder A Singh, Narinder Pal Singh, Paramdeep Singh, Surjit Singh, Anna Aleksandrovna Skryabina, Amanda E Smith, Yonatan Solomon, Yi Song, Reed J D Sorensen, Jeffrey D Stanaway, Mu'awiyyah Babale Sufiyan, Muhammad Suleman, Jing Sun, Dev Ram Sunuwar, Mindy D Szeto, Rafael Tabarés-Seisdedos, Seyed-Amir Tabatabaeizadeh, Shima Tabatabai, Moslem Taheri Soodejani, Jacques Lukenze JL Tamuzi, Ker-Kan Tan, Ingan Ukur Tarigan, Zerihun Tariku, Md Tariqujjaman, Elvis Enowbeyang Tarkang, Nathan Y Tat, Birhan Tsegaw Taye, Heather Jean Taylor, Yibekal Manaye Tefera, Arash Tehrani-Banihashemi, Mohamad-Hani Temsah, Masayuki Teramoto, Pugazhenthan Thangaraju, Rekha Thapar, Arulmani Thiyagarajan, Amanda G Thrift, Ales Tichopad, Jansje Henny Vera Ticoalu, Tala Tillawi, Tenaw Yimer Tiruye, Marcello Tonelli, Roman Topor-Madry, Mathilde Touvier, Marcos Roberto Tovani-Palone, Mai Thi

Ngoc Tran, Sana Ullah, Eduardo A Undurraga, Bhaskaran Unnikrishnan, Tolassa Wakayo Ushula, Seyed Mohammad Vahabi, Alireza Vakilian, Sahel Valadan Tahbaz, Rohollah Valizadeh, Jef Van den Eynde, Shoban Babu Varthya, Tommi Juhani Vasankari, Narayanaswamy Venketasubramanian, Madhur Verma, Massimiliano Veroux, Dominique Vervoort, Vasily Vlassov, Stein Emil Vollset, Rade Vukovic, Yasir Waheed, Cong Wang, Fang Wang, Molla Mesele Wassie, Kosala Gayan Weerakoon, Melissa Y Wei, Andrea Werdecker, Nuwan Darshana Wickramasinghe, Asrat Arja Wolde, Gedif Ashebir Wubetie, Ratna Dwi Wulandari, Rongbin Xu, Suowen Xu, Xiaoyue Xu, Lalit Yadav, Kazumasa Yamagishi, Lin Yang, Yuichiro Yano, Sanni Yaya, Fereshteh Yazdanpanah, Sisay Shewasinad Yehualashet, Arzu Yiğit, Vahit Yiğit, Dong Keon Yon, Chuanhua Yu, Chun-Wei Yuan, Giulia Zamagni, Sojib Bin Zaman, Aurora Zanghì, Moein Zangiabadian, Iman Zare, Michael Zastrozhin, Bethany Zigler, Mohammad Zoladl, Zhiyong Zou, Nicholas J Kassebaum, and Robert C Reiner Jr.

Affiliations

Institute for Health Metrics and Evaluation (M B Arndt PhD, A Y Aravkin PhD, N V Bhattacharjee PhD, J Chalek BS, X Dai PhD, Prof L Dandona MD, Prof R Dandona PhD, Prof S D Dharmaratne MD, S Farmer BA, Prof V L Feigin PhD, R Fitzgerald MPH, Prof E Gakidou PhD, W M Gardner MPH, H Hagins MSPH, Prof S I Hay FMedSci, J He MSc, K M Humphrey MS, Prof S S Lim PhD, P A Lindstedt MPH, J Lo BA, H Manguerra BS, T Mestrovic PhD, Prof A H Mokdad PhD, V Mougin BA, Prof C J L Murray DPhil, Q P Nguyen BS, M Pasovic MEd, C Raggi MS, R D Schneider MPPM, A E Smith MPA, R J D Sorensen PhD, J D Stanaway PhD, H J Taylor BA, Prof S E Vollset DrPH, A A Wolde MPH, C Yuan PhD, B Zigler MPH, N J Kassebaum MD, R C Reiner Jr PhD), Department of Global Health (M B Arndt PhD, R J D Sorensen PhD), Department of Applied Mathematics (A Y Aravkin PhD), Department of Health Metrics Sciences, School of Medicine (A Y Aravkin PhD, X Dai PhD, Prof R Dandona PhD, Prof S D Dharmaratne MD, Prof E Gakidou PhD, Prof S I Hay FMedSci, Prof S S Lim PhD, Prof A H Mokdad PhD, Prof C J L Murray DPhil, J D Stanaway PhD, Prof S E Vollset DrPH, N J Kassebaum MD, R C Reiner Jr PhD), School of Health Systems and Public Health (C Iwu MPH), Department of Anesthesiology & Pain Medicine (V Krishnamoorthy MD, N J Kassebaum MD), University of Washington, Seattle, WA, USA; Department of Clinical Governance and Quality Improvement (Y H Abate MSc), Aleta Wondo General Hospital, Aleta Wondo, Ethiopia; Non-communicable Diseases Research Center (M Abbasi-Kangevari MD), Department of Epidemiology (A Ahmadi PhD, Prof S Sabour PhD), National Nutrition and Food Technology Research Institute (M Ajami PhD), Department of Urology (M Bonakdar Hashemi MD), Department of Community Nutrition (S Doaei PhD), Social Determinants of Health Research Center (S Ghamari MD, J Khanali MD, M Rashidi MD), Cancer Research Center (M Gholamalizadeh PhD), Obesity Research Center (A Haj-Mirzaian MD), School of Medicine (M Heidari-Foroozan BSc, M Zangiabadian MD), Department of Neurosurgery (H Khayat Kashani MD), Department of Pharmacology (M Rezaee MD), Department of Medical Education (S Tabatabai PhD), Shahid Beheshti University of Medical Sciences, Tehran, Iran; Department of Epidemiology (S Abd ElHafeez DrPH), Pediatric Dentistry and Dental Public Health Department (Prof O A A Elmeligy PhD), Alexandria University, Alexandria, Egypt; Department of Surgery (M Abdelmasseh MD, Prof J Sanabria MD), Marshall University, Huntington, WV, USA; Department of Tropical Medicine and Infectious Diseases (S Abd-Elsalam PhD), Department of Pediatrics (A H Darwish MD), Tanta University, Tanta, Egypt; Community and Maternity Nursing Unit (D M Abdulah MPH), University of Duhok, Duhok, Iraq; National Institute of Epidemiology (R Abdulkader PhD), Indian Council of Medical Research, Chennai, India; Laboratory Technology Sciences Department (H Abidi PhD), Department of Nursing (M Zoladl PhD), Yasuj University of Medical Sciences, Yasuj, Iran; Department of Community Medicine (Prof O Abiodun MPH), Babcock University, Ilishan-Remo, Nigeria; Department of Family and Community

Health (R G Aboagye MPH), Department of Population and Behavioural Sciences (H Amu PhD, Prof E E Tarkang PhD), Institute of Health Research (M Immurana PhD), University of Health and Allied Sciences, Ho, Ghana (R K Alhassan PhD); Research Center for Immunodeficiencies (H Abolhassani PhD), Iranian Research Center for HIV/AIDS (IRCHA) (O Dadras PhD), Non-communicable Diseases Research Center (S Ghamari MD, M Keykhaei MD, M Rashidi MD, N Rezaei MD), Department of Ophthalmology (Prof F Ghassemi MD), Students' Scientific Research Center (SSRC) (M Keykhaei MD), Non-Communicable Diseases Research Center (J Khanali MD, S Saeedi Moghaddam MSc), School of Medicine (S Khanmohammadi MD, A Shahbandi MD), Children's Medical Center (Prof F Kompani MD), Department of Pediatric Cardiology (Prof E Malakan Rad MD), Sina Trauma and Surgery Research Center (Prof V Rahimi-Movaghar MD), Tehran Heart Center (M Rezaee MD), Digestive Diseases Research Institute (S G Sepanlou MD), Department of Neurology (M Shafie MD), Faculty of Medicine (S Vahabi MD), Department of Pediatric Allergy and Immunology (F Yazdanpanah MD), Tehran University of Medical Sciences, Tehran, Iran (K Ghaffari PhD); Department of Medical Biochemistry and Biophysics (H Abolhassani PhD), Department of Molecular Medicine and Surgery (Prof J H Kauppila MD), Karolinska Institute, Stockholm, Sweden; Department of Biomedical Science (Y D Abtew MSc), Department of Medical Anatomy (A Bekele MSc), Department of Anatomy (S Demissie MSc), Department of Public Health (H Esubalew MPH, G A Wubetie MPH), Arba Minch University, Arba Minch, Ethiopia; Department of Clinical Sciences (Prof E Abu-Gharbieh PhD), Department of Physiotherapy (A Arumugam PhD), Clinical Sciences Department (Prof M M Saber-Ayad PhD), College of Medicine (Prof B A Saddik PhD), Sharjah Institute of Medical Sciences (F Saheb Sharif-Askari PhD), University of Sharjah, Sharjah, United Arab Emirates (K A Altirkawi MD); Department of Biopharmaceutics and Clinical Pharmacy (Prof E Abu-Gharbieh PhD), University of Jordan, Amman, Jordan; Institute of Community and Public Health (Prof N M Abu-Rmeileh PhD), Birzeit University, Ramallah, Palestine; Department of Clinical Medicine (Prof J M Acuna MD), American University of Antigua, Coolidge, Antigua and Barbuda; FIU Robert Stempel College of Public Health & Social Work (Prof J M Acuna MD), Department of Epidemiology (R Jebai MPH), Florida International University, Miami, FL, USA; Department of Health System Management (K Adamu MPH), Department of Public Health (F M Hussien MPH, F M Hussien MPH), Wollo University, Dessie, Ethiopia; Department of Anesthesia and Critical Care (D E Adane MSc), Department of Public Health (M T Asemu MSc), Debre Tabor University, Debre Tabor, Ethiopia; School of Medicine (I Y Addo PhD), School of Architecture, Design, and Planning (Prof T Astell-Burt PhD), Menzies Centre for Health Policy and Economics (M Balasubramanian PhD), Sydney Medical School (S Islam PhD), Save Sight Institute (H Kandel PhD), Department of Public Health (M Khan PhD), University of Sydney, Sydney, NSW, Australia; Centre for Social Research in Health (I Y Addo PhD), St George and Sutherland Clinical School (H Akbarialiabad MD), School of Population Health (X Feng PhD, Prof B A Saddik PhD, Prof A E Schutte PhD, X Xu PhD), University of New South Wales, Sydney, NSW, Australia; Department of Community Health and Epidemiology (D A Adeyinka PhD), University of Saskatchewan, Saskatoon, SK, Canada; Department of Public Health (D A Adeyinka PhD), Federal Ministry of Health, Abuja, Nigeria; Department of Public Health (Q Adnani PhD), Universitas Padjadjaran (Padjadjaran University), Bandung, Indonesia; Technical Services Directorate (A A Afolabi MPH), MSI Nigeria Reproductive Choices, Abuja, Nigeria; School of Medicine (F Afrashteh MD), Pars Advanced and Minimally Invasive Medical Manners Research Center (Y Alimohamadi PhD), Health Management and Economics Research Center (J Arabloo PhD), Department of Ophthalmology (H Hasani MD), Minimally Invasive Surgery Research Center (A Kabir MD), Preventive Medicine and Public Health Research Center (A Tehrani-Banihashemi PhD), Department of Community and Family Medicine (A Tehrani-Banihashemi PhD), Iran University of Medical Sciences, Tehran, Iran (M

Moradi MD); Department of Community Medicine (Prof S Afzal PhD), King Edward Memorial Hospital, Lahore, Pakistan; Department of Public Health (Prof S Afzal PhD), Public Health Institute, Lahore, Pakistan; Department of Medical and Surgical Sciences and Advanced Technologies "GF Ingrassia" (Prof A Agodi PhD, M Barchitta PhD, Prof E D'Amico MD, A Maugeri PhD, Prof M Veroux PhD), Department of Biomedical and Biotechnological Sciences (L Falzone PhD), Department of General Surgery and Medical-Surgical Specialties (Prof G Isola PhD), Department of Clinical and Experimental Medicine (Prof C Ledda PhD), University of Catania, Catania, Italy; School of Public Health (B O Ahinkorah MPhil), University of Technology Sydney, Sydney, NSW, Australia; Department of Medical Biochemistry (A Ahmad PhD), College of Medicine (Prof G Mustafa MD), Department of Pharmacology (A R Pathan PhD), Shaqra University, Shaqra, Saudi Arabia; Department of Health and Biological Sciences (S Ahmad PhD), Abasyn University, Peshawar, Pakistan; Department of Natural Sciences (S Ahmad PhD), Gilbert and Rose-Marie Chagoury School of Medicine (Prof L Roever PhD), Lebanese American University, Beirut, Lebanon; School of Public Health (T Ahmad PhD), Zhejiang University, Hangzhou, China; Department of Epidemiology and Biostatistics (A Ahmadi PhD), Modeling in Health Research Center (A Mohammadian-Hafshejani PhD), Shahrekord University of Medical Sciences, Shahrekord, Iran; Department of Pharmacy Practice (A Ahmed PhD), Riphah Institute of Pharmaceutical Sciences, Islamabad, Pakistan; Division of Infectious Diseases and Global Public Health (IDGPH) (A Ahmed PhD), University of California San Diego, San Diego, CA, USA; Institute of Public Health (Prof L A Ahmed PhD, I Elbarazi DrPH, Prof M Grivna PhD), Family Medicine Department (M A Khan MSc), United Arab Emirates University, Al Ain, United Arab Emirates; Faculty of Medicine and Public Health (B Aji DrPH), Jenderal Soedirman University, Purwokerto, Indonesia; Department of Epidemiology and Biostatistics (M Akonde MLS), University of South Carolina, Columbia, SC, USA; Department of Geriatric and Long Term Care (H Al Hamad MD), Rumailah Hospital (H Al Hamad MD), Hamad Medical Corporation, Doha, Qatar; Faculty of Pharmacy (Y Al Thaher PhD), Faculty of Nursing (M M W Atout PhD), Philadelphia University, Amman, Jordan; School of Pharmacy (Y Al Thaher PhD), Cardiff University, Cardiff, UK; Department of Research and Development (Z Al-Aly MD), Department of Surgery (S Azadnajafabad MD, C Wang PhD), Washington University in St. Louis, St. Louis, MO, USA; Clinical Epidemiology Center (Z Al-Aly MD), US Department of Veterans Affairs (VA), St. Louis, MO, USA; Department of Cardiac Sciences (Prof K F Alhabib MD), Pediatric Intensive Care Unit (Prof M Temsah MD), King Saud University, Riyadh, Saudi Arabia; Erbil Technical Health College (B A Ali PhD), Erbil Polytechnic University, Erbil, Iraq; School of Pharmacy (B A Ali PhD), Tishk International University, Erbil, Iraq; Center for Biotechnology and Microbiology (S S Ali PhD, M Suleman PhD), University of Swat, Swat, Pakistan; Department of Public Health and Community Medicine (Prof S M Aljunid PhD), International Medical University, Kuala Lumpur, Malaysia; International Centre for Casemix and Clinical Coding (Prof S M Aljunid PhD), National University of Malaysia, Bandar Tun Razak, Malaysia; Department of Parasitology (Prof H M Al-Mekhlafi PhD), Department of Paediatrics (Prof H Ariffin PhD), University of Malaya Medical Centre (Prof H Ariffin PhD), University of Malaya, Kuala Lumpur, Malaysia; Department of Parasitology (Prof H M Al-Mekhlafi PhD), Sana'a University, Sana'a, Yemen; College of Medicine (S Almustanyir MD, Prof O Baltatu PhD), Alfaisal University, Riyadh, Saudi Arabia; Ministry of Health, Riyadh, Saudi Arabia (S Almustanyir MD); Department of Physical Therapy and Rehabilitation Sciences (Prof M A Alomari PhD), Department of Rehabilitation Sciences and Physical Therapy (Prof M A Alomari PhD), Department of Public Health (Prof Y S Khader PhD), Jordan University of Science and Technology, Irbid, Jordan; Research, Policy, and Training Directorate (A Al-Tammemi MPH), Jordan Center for Disease Control, Amman, Jordan; Applied Science Research Center (A Al-Tammemi MPH), Applied Science Private University, Amman, Jordan; Research Group in Health

Economics (Prof N Alvis-Guzman PhD), Universidad de Cartagena (University of Cartagena), Cartagena, Colombia; Research Group in Hospital Management and Health Policies (Prof N Alvis-Guzman PhD), Department of Economic Sciences (N J Alvis-Zakzuk MSc), Universidad de la Costa (University of the Coast), Barranquilla, Colombia; National Health Observatory (N J Alvis-Zakzuk MSc), National Institute of Health, Bogota, Colombia; School of Graduate Studies (E K Ameyaw MPhil), Lingnan University, Hong Kong, China; Public Health and Community Medicine Department (Prof T T Amin MD), Cairo University, Cairo, Egypt; Quran and Hadith Research Center (S Amiri PhD), Baqiyatallah University of Medical Sciences, Tehran, Iran; Department of Maternal and Child Wellbeing (D A Amugsi PhD), African Population and Health Research Center, Nairobi, Kenya; Department of Health Promotion (T F Anagaw MPH), Department of Biomedical Sciences (S A Belay MSc), Department of Physiology (D Demeke MSc), Department of Health Informatics (H A Guadie MPH), Department of Public Health Nutrition (Y Melaku PhD), Bahir Dar University, Bahir Dar, Ethiopia; Faculty of Pharmacy (Prof R Ancuceanu PhD), Department of Internal Medicine (M Hostiuc PhD), Department of Legal Medicine and Bioethics (Prof S Hostiuc PhD), Department of General Surgery (D Serban PhD), Carol Davila University of Medicine and Pharmacy, Bucharest, Romania; Department of Child Neurology (D Angappan MD), Oregon Health and Science University, Portland, OR, USA; Department of Epidemiology and Biostatistics (Prof A Ansari-Moghaddam PhD), Health Promotion Research Center (M Khammarnia PhD, H Okati-Aliabad PhD), Department of Biochemistry (S Sargazi PhD), Zahedan University of Medical Sciences, Zahedan, Iran; Agribusiness Study Program (E Antriyandarti DrAgrSc), Sebelas Maret University, Surakarta, Indonesia; Department of Parasitology (D Anvari PhD), Iranshahr University of Medical Sciences, Iranshahr, Iran; Rural Health Research Institute (A E Anyasodor PhD, Prof J Sun PhD), Charles Sturt University, Orange, NSW, Australia; Public Health and Healthcare Management (T Aripov PhD), Tashkent Institute of Postgraduate Medical Education, Tashkent, Uzbekistan; Boston Children's Hospital, Boston, MA, USA (T Aripov PhD); Department of Medical Laboratory Sciences (M Arkew MSc), Department of Health Policy and Management (A T Debele MSc), Department of Nutrition and Reproductive Health (J Y Kebira MPH), School of Public Health (A Masrie MPH, A Oumer PhD), Haramaya University, Harar, Ethiopia; Department of Cardiovascular, Endocrine-Metabolic Diseases and Aging (B Armocida MD), Istituto Superiore di Sanità (ISS), Rome, Italy; Department of Physiotherapy (A Arumugam PhD), Kasturba Medical College, Mangalore (D B B MD, R Holla MD, Prof B Unnikrishnan MD), Department of Pharmaceutical Regulatory Affairs and Management (V S Ligade PhD), Department of Community Medicine (C R Rao MD), Department of Nephrology (I Rao DM), Manipal Academy of Higher Education, Manipal, India (H L Dsouza MD); National Research and Innovation Agency, Jakarta, Indonesia (N K Aryastami PhD, I U Tarigan PhD); Department of Plastic Surgery (M Asaad MD), Health Science Center (D Dongarwar MS), University of Texas, Houston, TX, USA; Research Center for Biochemistry and Nutrition in Metabolic Diseases (Z Asemi PhD), Kashan University of Medical Sciences, Kashan, Iran; Cabrini Research (Prof M Asghari-Jafarabadi PhD), Cabrini Health, Malvern, VIC, Australia; School of Public Health and Preventative Medicine (Prof M Asghari-Jafarabadi PhD), Department of Epidemiology and Preventive Medicine (M A Awoke MPH), Department of Epidemiology and Preventative Medicine (E K Chowdhury PhD), Department of Medicine (Prof A G Thrift PhD), School of Public Health and Preventive Medicine (R Xu PhD), Monash University, Melbourne, VIC, Australia; Department of Immunology (S Athari PhD), Zanjan University of Medical Sciences, Zanjan, Iran; Pediatrics and Child Health Nursing (G H Atomsa MSc), Department of Public Health (U Gerema MSc, M E Getachew MPH), Department of Biomedical Sciences (D Olana MSc), Department of Dietetics and Nutrition (T W Ushula MSc), Jimma University, Jimma, Ethiopia; School of Medicine and Public Health (P Atorkey PhD), University of

Newcastle, Newcastle, NSW, Australia; Australian College of Applied Professions (P Atorkey PhD), Australian College of Applied Professions, Sydney, NSW, Australia; Northumbria HealthCare NHS Foundation Trust, Newcastle upon Tyne, UK (A Aujayeb MBBS); Leeds Institute of Rheumatic and Musculoskeletal Medicine (S Azadnajafabad MD), School of Dentistry (J Patel BSc), University of Leeds, Leeds, UK; Department of Sciences (Prof R M S Azevedo PhD), Toxicology Research Unit (TOXRUN) (Prof D Dias da Silva PhD, Á M Madureira-Carvalho PhD), Cooperativa de Ensino Superior Politécnico e Universitário (University Polytechnic Higher Education Cooperative) (CESPU), Gandra, Portugal; Department of Forensic Science (A D Badiye PhD, N Kapoor PhD), Government Institute of Forensic Science Nagpur, Nagpur, India; Rashtrasant Tukadoji Maharaj Nagpur University, Nagpur, India (A D Badiye PhD); Department of Nursing (N Baghcheghi PhD), Social Determinants of Health Research Center (H Koohestani PhD), Saveh University of Medical Sciences, Saveh, Iran; Health Research Institute (Prof N Bagheri PhD), University of Canberra, Canberra, ACT, Australia; School of Medicine (S Bagherieh BSc), Department of Health Services Management (M Mohseni PhD), Isfahan University of Medical Sciences, Isfahan, Iran; International Medical School (A A Baig PhD), Management and Science University, Alam, Malaysia; Center for Clinical Research and Prevention (J L Baker PhD), Bispebjerg University Hospital, Frederiksberg, Denmark; Health Care Management Department (M Balasubramanian PhD), College of Medicine and Public Health (T G Gebremeskel PhD, B Kaambwa PhD), Health Economics Unit (B Kaambwa PhD), Flinders Health and Medical Research Institute (FHMRI) (Y Melaku PhD), Department of Nursing and Health Sciences (S Shorofi PhD), Flinders University, Adelaide, SA, Australia; Center of Innovation, Technology and Education (CITE) (Prof O Baltatu PhD), Anhembi Morumbi University, São José dos Campos, Brazil; Department of Hypertension (Prof M Banach PhD), Medical University of Lodz, Lodz, Poland; Polish Mothers' Memorial Hospital Research Institute, Lodz, Poland (Prof M Banach PhD); Department of Non-communicable Diseases (P C Banik MPhil, L Barua MPH), Bangladesh University of Health Sciences, Dhaka, Bangladesh; Heidelberg Institute of Global Health (HIGH) (Prof T W Bärnighausen MD, Prof S Mohammed PhD), Department of Ophthalmology (S Panda-Jonas MD), Heidelberg University, Heidelberg, Germany; T.H. Chan School of Public Health (Prof T W Bärnighausen MD), Dana-Farber Cancer Institute (C Cao PhD), Division of Cardiology (I Y Elgendy MD), Maternal Fetal Care Center (K Hessami MD), Department of Health Policy and Oral Epidemiology (Z S Natto DrPH), Department of Global Health and Population (P Rohloff MD), Harvard University, Boston, MA, USA; Department of Pediatrics (Prof R D Barr MD), Department of Medicine (O P Kurmi PhD), Department of Psychiatry and Behavioural Neurosciences (A T Olagunju MD), McMaster University, Hamilton, ON, Canada; Department of Public and Environmental Health (A Barrow MPH), University of The Gambia, Banjul, The Gambia; Department of Epidemiology (A Barrow MPH, D Braithwaite PhD), University of Florida, Gainesville, FL, USA; Health Information Management (A Bashiri PhD), Maternal Fetal Medicine Research Center (K Hessami MD), Basic Science Laboratory (F Iravanpour PhD), Department of Biostatistics (E Sadeghi PhD), Health Policy Research Center (Y Sarikhani PhD), Non-communicable Disease Research Center (S G Sepanlou MD), Shiraz University of Medical Sciences, Shiraz, Iran; Department of Community Medicine (P Baskaran MD), Sri Manakula Vinayagar Medical College and Hospital, Puducherry, Puducherry, India; Department of Academics (S Basu MD), Indian Institute of Public Health, Gurgaon, India; Department of Oral Pathology and Microbiology (U I Belgaumi MD), Department of Oral and Maxillofacial Pathology (V Kadashetti MDS), Department of Public Health Dentistry (Prof S M Kondlahalli MD), Krishna Vishwa Vidyapeeth (Deemed to be University), Karad, India; School of the Environment (Prof S L Bell PhD), Department of Internal Medicine (F Etaee MD), Department of Psychiatry (W Li PhD), Department of Genetics (S Pawar PhD), Yale University, New

Haven, CT, USA; School of Health Policy and Management (Prof S L Bell PhD), Department of Preventive Medicine (Prof Y Lee PhD), Korea University, Seoul, South Korea; Department of Biological Sciences (Prof L Belo PhD), Research Unit on Applied Molecular Biosciences (UCIBIO) (Prof L Belo PhD, Prof D Dias da Silva PhD), Associated Laboratory for Green Chemistry (LAQV) (M Carvalho PhD), Institute for Research and Innovation in Health (i3S) (Prof N Cruz-Martins PhD), University of Porto, Porto, Portugal; Nuffield Department of Population Health (D A Bennett PhD, B Lacey DPhil), University of Oxford, Oxford, UK; Department of Internal Medicine (I M Bensenor PhD, I S Santos PhD), Center for Clinical and Epidemiological Research (I S Santos PhD), University of São Paulo, São Paulo, Brazil; Department of Public Health (G Beressa MSc), Madda Walabu University, Addis Ababa, Ethiopia; Department of Nutrition and Dietetics (G Beressa MSc), Jimma University, Addis Ababa, Ethiopia; Department of Epidemiology and Biostatistics (A C Bermudez MD), National Institutes of Health (A Loreche BS), University of the Philippines Manila, Manila, Philippines; Department of Epidemiology (A C Bermudez MD), Brown University, Providence, RI, USA; Metabolomics Laboratory (H B Beyene PhD), Hypertension and Kidney Disease Laboratory (Prof M P Schlaich MD), Baker Heart and Diabetes Institute, Melbourne, VIC, Australia; Department of Microbiology (H B Beyene PhD), Addis Ababa University, Addis Ababa, Ethiopia; Department of Public Health (A S Bhagavathula PhD), North Dakota State University, Fargo, ND, USA; Division of Gastroenterology and Hepatology (A S Bhagavathula PhD), Mayo Clinic, Jacksonville, FL, USA; Department of Anatomy (N Bhardwaj MD), Department of Community Medicine and Family Medicine (Prof P Bhardwaj MD, Prof P Raghav MD), School of Public Health (Prof P Bhardwaj MD), Department of Pharmacology (J Charan MD, S Singh MD, S B Varthya MD), Department of Surgical Oncology (Prof S Misra MCh), All India Institute of Medical Sciences, Jodhpur, India; Global Health Neurology Lab (S Bhaskar MD), NSW Brain Clot Bank, Sydney, NSW, Australia; Division of Cerebrovascular Medicine and Neurology (S Bhaskar MD), National Cerebral and Cardiovascular Center, Suita, Japan; Centre for Global Child Health (Prof Z A Bhutta PhD), Temerty Faculty of Medicine (V Chattu MD), University of Toronto, Toronto, ON, Canada; Centre of Excellence in Women & Child Health (Prof Z A Bhutta PhD), Division of Women and Child Health (J K Das MD), Aga Khan University, Karachi, Pakistan; Department of Community Medicine (Prof S Bitaraf PhD), Department of Pediatric Neurology (S Sadeghian MD), Ahvaz Jundishapur University of Medical Sciences, Ahvaz, Iran; School of Business Administration (Prof V Bodolica PhD), American University of Sharjah, Sharjah, United Arab Emirates; Cancer Population Sciences Program (D Braithwaite PhD), University of Florida Health Cancer Center, Gainesville, FL, USA; Faculty of Pharmacy (M Butt MS), Department of Biotechnology (M Naveed PhD), University of Central Punjab, Lahore, Pakistan; School of Public Health Sciences (Z A Butt PhD), University of Waterloo, Waterloo, ON, Canada; Al Shifa School of Public Health (Z A Butt PhD), Al Shifa Trust Eye Hospital, Rawalpindi, Pakistan; Department of Clinical Pharmacy (Prof D Calina PhD), University of Medicine and Pharmacy of Craiova, Romania, Craiova, Romania; Department of Internal Medicine (Prof L A Cámera MD), Hospital Italiano de Buenos Aires (Italian Hospital of Buenos Aires), Buenos Aires, Argentina; Board of Directors (Prof L A Cámera MD), Argentine Society of Medicine, Buenos Aires, Argentina; Institute of Biomedical Engineering (Prof L A Campos PhD), Anhembi Morumbi University, Sao Jose dos Campos, Brazil; Department of Biomedical Engineering (Prof L A Campos PhD), Center of Innovation, Technology and Education (CITE) at São José dos Campos Technology Park, Sao Jose dos Campos, Brazil; Department of Health Care (Prof R Cárdenas DSc), Metropolitan Autonomous University, Mexico City, Mexico; Faculty of Health Sciences (M Carvalho PhD), University Fernando Pessoa, Porto, Portugal; Colombian National Health Observatory (C A Castañeda-Orjuela PhD), Instituto Nacional de Salud (National Institute of Health), Bogota, Colombia; Epidemiology and Public Health

Evaluation Group (C A Castañeda-Orjuela PhD), Department of Public Health (Prof F P De la Hoz PhD), National University of Colombia, Bogota, Colombia; Department of Pharmacological and Biomolecular Sciences (Prof A L Catapano PhD), Department of Clinical Sciences and Community Health (Prof C La Vecchia MD), Department of Food, Environmental and Nutritional Sciences (Prof S Perna PhD), University of Milan, Milan, Italy; MultiMedica Sesto San Giovanni IRCCS, Sesto San Giovanni, Italy (Prof A L Catapano PhD); Department of Public Health and Infectious Diseases (M S Cattaruzza PhD), La Sapienza University, Rome, Italy; Department of Nutrition (Prof F Cembranel DSc), Federal University of Santa Catarina, Florianópolis, Brazil; Mary MacKillop Institute for Health Research (Prof E Cerin PhD), Australian Catholic University, Melbourne, VIC, Australia; School of Public Health (Prof E Cerin PhD), University of Hong Kong, Hong Kong, China; Non-communicable Diseases Division (J Chadwick MD), Division of Epidemiology and Biostatistics (V K Kamal PhD), National Institute of Epidemiology, Chennai, India; Department of Anesthesiology and Perioperative Medicine (E K Chandrasekar MD), School of Medicine (Prof S Xu PhD), University of Rochester, Rochester, NY, USA; Department of Community Medicine (V Chattu MD), Datta Meghe Institute of Medical Sciences, Sawangi, India; Department of Biostatistics and Epidemiology (K Chauhan PhD), Indian Institute of Public Health, Shillong, India; Department of Laboratory Medicine (J Chien PhD), Taichung Tzu-Chi Hospital Buddhist Tzu-Chi Medical Foundation, Tanzih, Taiwan; Department of Medical Laboratory Science and Biotechnology (J Chien PhD), Central Taiwan University of Science and Technology, Taiwan; Iraq Field Epidemiology Training Program (I-FETP) (A Chitheer MD), Ministry of Health, Baghdad, Iraq; Department of Community Medicine (Prof S G Choudhari MD), Jawaharlal Nehru Medical College, Wardha, India; School of Public Health (E K Chowdhury PhD, T R Miller PhD), Curtin University, Perth, WA, Australia; The Interdisciplinary Research Group on Biomedicine and Health (D Chu PhD), Faculty of Applied Sciences (D Chu PhD), VNU International School (VNUIS), Hanoi, Vietnam; Department of Paediatric Surgery (I S Chukwu BMedSc), Federal Medical Centre, Umuahia, Nigeria; Department of Health Informatics (S Chung PhD), Medical Research Council Clinical Trials Unit (N M Noor MRCP), University College London, London, UK; Health Data Research UK, London, UK (S Chung PhD); Department of Nutrition (Prof R M Claro PhD), Department of Maternal-Child Nursing and Public Health (Prof D C Malta PhD), Federal University of Minas Gerais, Belo Horizonte, Brazil; Department of Biostatistics (A Columbus MS), Department of Health Policy and Management (D Vervoort MD), Johns Hopkins University, Baltimore, MD, USA (E Jamshidi PharmD); School of Psychology (Prof S Cortese PhD), Southampton Clinical Trials Unit (P H Lee PhD), University of Southampton, Southampton, UK; Department of Child and Adolescent Psychiatry (Prof S Cortese PhD), Institute for Excellence in Health Equity (M Kumar PhD), New York University, New York, NY, USA; Department of Diagnostic and Therapeutic Technologies (Prof N Cruz-Martins PhD), Cooperativa de Ensino Superior Politécnico e Universitário (Polytechnic and University Higher Education Cooperative), Vila Nova de Famalicão, Portugal; Department of Epidemiology & Biostatistics (B Dabo MSPH), University of South Florida, Tampa, FL, USA; Department of Medical Microbiology and Parasitology (B Dabo MSPH), Bayero University, Kano, Nigeria; Department of Global Public Health and Primary Care (O Dadras PhD), University of Bergen, Bergen, Norway; Public Health Foundation of India, Gurugram, India (Prof L Dandona MD, Prof R Dandona PhD, G Kumar PhD, A Pandey PhD); Non-communicable Diseases Division (NCD) (A K Gupta PharmD), Department of Biostatistics (V K Kamal PhD), Indian Council of Medical Research, New Delhi, India (Prof L Dandona MD, D K Lal MD); Department of Medical Microbiology (I Darban BSc), University of Ghana, Accra, Ghana; Department of Pediatrics (Prof G L Darmstadt MD), Stanford University School of Medicine, Stanford, CA, USA; Department of Information Technology (A M Darwesh PhD), University of Human Development,

Sulaymaniyah, Iraq; Department of Biochemistry (S Das MD), Ministry of Health and Welfare, New Delhi, India; Health Research Institute (Prof K Davletov PhD), Director of the Scientific and Technological Park (I R Fakhradiyev PhD), Kazakh National Medical University, Almaty, Kazakhstan; Center for Nutrition and Health Research (E Denova-Gutiérrez DSc), Department of Obesity, Diabetes and Cardiovascular Risk (Prof J Espinosa-Montero PhD), Center for Health Systems Research (D V Ortega-Altamirano EdD), National Institute of Public Health, Cuernavaca, Mexico; Department of Research (H D Desai MD), Gujarat Adani Institute of Medical Sciences, Bhuj, India; Department of Surgical Nursing (A A Desta MSc), Department of Human Physiology (M Diress MSc), Department of Health Promotion and Health Behavior (H Eshetu MPH), Department of Hematology and Immunohematology (S Getawa MSc), Department of Clinical Midwifery (T W Gudayu MPH), Department of Pharmacology (Z D Kifle MSc), Institute of Public Health (M M Sharew MPH, M M Wassie MSc), University of Gondar, Gondar, Ethiopia; Department of Community Medicine (Prof S D Dharmaratne MD), University of Peradeniya, Peradeniya, Sri Lanka; Research Department (M Dhimal PhD), Nepal Health Research Council, Kathmandu, Nepal; Institute of Occupational, Social and Environmental Medicine (M Dhimal PhD), Goethe University Frankfurt, Frankfurt am Main, Germany; Faculty of Science (Prof D Diaz PhD), National Autonomous University of Mexico, Mexico City, Mexico; Development of Research and Technology Center (S Djalalinia PhD), Ministry of Health and Medical Education, Tehran, Iran; School of Health (S Doaei PhD), Department of Social Medicine and Epidemiology (A Feizkhah MD), Gastrointestinal and Liver Diseases Research Center (S Hassanipour PhD), Caspian Digestive Disease Research Center (S Hassanipour PhD), Guilan University of Medical Sciences, Rasht, Iran; Department of Forensic Medicine and Toxicology (H L Dsouza MD), Kasturba Medical College Mangalore, Mangalore, India; Department of Community Nutrition (S Edalati PhD), Qazvin University of Medical Sciences, Qazvin, Iran; School of Health Sciences (H A Edinur PhD), Universiti Sains Malaysia (University of Science Malaysia), Kubang Kerian, Malaysia; Faculty of Science and Health (M Ekholuenetale PhD), University of Portsmouth, Hampshire, UK; Department of Microbiology (T C Ekundayo PhD), University of Medical Sciences, Ondo, Ondo, Nigeria; Division of Cardiovascular Medicine (I Y Elgendy MD), University of Kentucky, Lexington, KY, USA; Faculty of Medicine (M Elhadi MD), University of Tripoli, Tripoli, Libya; Houston Methodist Hospital, Houston, TX, USA (M Elhadi MD); Department of Pediatric Dentistry (Prof O A A Elmeligy PhD), Rabigh Faculty of Medicine (Prof A Malik PhD), Department of Dental Public Health (Z S Natto DrPH), King Abdulaziz University, Jeddah, Saudi Arabia; Department of Pediatrics & Child Health Nursing (W Etafa MSc), Department of Nursing (G Fetensa MSc), Department of Public Health (M E Getachew MPH), Wollega University, Nekemte, Ethiopia; Department of Epidemiology and Medical Statistics (A F Fagbamigbe PhD, K R Fowobaje MSc), Department of Obstetrics and Gynecology (O B Oghenetega MSc), College of Medicine (A P Okekunle PhD), University of Ibadan, Ibadan, Nigeria; Research Centre for Healthcare and Community (A F Fagbamigbe PhD), Faculty of Health and Life Sciences (O P Kurmi PhD), Coventry University, Coventry, UK; Epidemiology and Biostatistics Unit (L Falzone PhD), IRCCS Pascale, Naples, Italy; Dissemination Division (C S e Farinha MSc), National Institute of Statistics, Lisbon, Portugal; Activity Planning and Control Unit (C S e Farinha MSc), Directorate-General of Health (DGS), Lisbon, Portugal; Satcher Health Leadership Institute (A O Fasanmi PhD), Morehouse School of Medicine, Atlanta, GA, USA; School of Medicine (A O Fasanmi PhD), Department of Cardiology (P Ram MD), Emory University, Atlanta, GA, USA; School of Engineering (A Fatehizadeh PhD), Edith Cowan University, Joondalup, WA, Australia; National Institute for Stroke and Applied Neurosciences (Prof V L Feigin PhD), Auckland University of Technology, Auckland, New Zealand; Research Center of Neurology, Moscow, Russia (Prof V L Feigin PhD); National Institute of Environmental Health (X Feng PhD), Chinese Center for Disease

Control and Prevention, Beijing, China; Center for Public Health Research (P Ferrara PhD), University of Milan Bicocca, Monza, Italy; Laboratory of Public Health (P Ferrara PhD), IRCCS Istituto Auxologico Italiano, Milan, Italy; Institute of Public Health (F Fischer PhD), Charité Universitätsmedizin Berlin (Charité Medical University Berlin), Berlin, Germany; Center for Research in Indigenous Health (D Flood MD), Maya Health Alliance, Tecpán, Guatemala; Department of Internal Medicine (D Flood MD), University of Michigan, Ann Arbor, MI, USA; Institute of Gerontology (N A Foigt PhD), National Academy of Medical Sciences of Ukraine, Kyiv, Ukraine; Department of Child Dental Health (Prof M O Folayan FWACS), Obafemi Awolowo University, Ile-Ife, Nigeria; Clinical Science Department (Prof M O Folayan FWACS), Nigerian Institute of Medical Research, Lagos, Nigeria; Child Survival Unit (K R Fowobaje MSc), Centre for African Newborn Health and Nutrition, Ibadan, Nigeria; College of Public Health, Medical, and Veterinary Sciences (Prof R C Franklin PhD), James Cook University, Townsville, QLD, Australia (K O Obamiro PhD); Department of Dermatology (T Fukumoto PhD), Kobe University, Kobe, Japan; Department of Community Medicine (Prof M A Gadanya MD), Bayero University Kano, Kano, Nigeria; Department of Community Medicine (Prof M A Gadanya MD), Aminu Kano Teaching Hospital, Kano, Nigeria; Department of Community Medicine (Prof A M Gaidhane MD), Datta Meghe Institute of Medical Sciences, Wardha, India; Institute of Applied Health Sciences (S Gaihre PhD), University of Aberdeen, Aberdeen, UK; Department of Food Technology (Y Galali ResM), Department of Biology (K A Mohammad PhD), Salahaddin University-Erbil, Erbil, Iraq; Department of Nutrition and Dietetics (Y Galali ResM), Cihan University-Erbil, Erbil, Iraq; Faculty of Paramedicine (N Galehdar PhD), Lorestan University of Medical Sciences, Khorramabad, Iran; Obstetrics and Gynaecology (P Garg MD), Department of Hospital Administration (M Mirza MD), Department of Community Medicine and Family Medicine (S S Sahoo MD, M Verma MD), Department of Radiodiagnosis (P Singh MD), All India Institute of Medical Sciences, Bathinda, India; Department of Reproductive and Family Health (T G Gebremeskel PhD), Axum College of Health Science, Axum, Ethiopia; Department of Public Health (L Getacher PhD), Department of Nursing (W M Kebede MSc), School of Nursing and Midwifery (B Taye MSc), Department of Pediatrics and Child Health Nursing (S S Yehualashet MSc), Debre Berhan University, Debre Berhan, Ethiopia; Department of Laboratory Sciences (K Ghaffari PhD), Khomein University of Medical Sciences, Khomein, Iran; E-Learning Center (M Ghasemi Nour MD), Biotechnology Research Center (Prof A Sahebkar PhD), Mashhad University of Medical Sciences, Mashhad, Iran; Research Group for Childhood Cancer (N Ghith PhD), Cancer Research Institute, Danish Cancer Society, Copenhagen, Denmark; Department of Epidemiology and Biostatistics (A Gholami PhD), Non-Communicable Diseases Research Center (A Gholami PhD), Neyshabur University of Medical Sciences, Neyshabur, Iran; Department of Radiology (A Gholamrezanezhad MD), University of Southern California, Los Angeles, CA, USA; Departments of Radiology and Neurosurgery (S Ghozy MD), Mayo Clinic, Rochester, MN, USA; Warwick Medical School (Prof P S Gill DM, Prof J A B Rodriguez PhD), University of Warwick, Coventry, UK; Adelaide Medical School (T K Gill PhD), University of Adelaide, Adelaide, SA, Australia; NIHR Global Health Research Unit on Global Surgery (J C Glasbey MSc), University of Birmingham, Birmingham, UK; Department of Health Systems and Policy Research (Prof M Golechha PhD), Indian Institute of Public Health, Gandhinagar, India; Department of Genetics (P Goleij MSc), Sana Institute of Higher Education, Sari, Iran; Universal Scientific Education and Research Network (USERN) (P Goleij MSc), Kermanshah University of Medical Sciences, Kermanshah, Iran; Department of Life Sciences, Health and Healthcare Professions (Prof D Golinelli MD), Link Campus University, Rome, Italy; Health Services Research, Evaluation and Policy Unit (Prof D Golinelli MD), AUSL della Romagna, Ravenna, Italy; Department of Respiratory Medicine (H Goudarzi PhD), Center for Environmental and Health Sciences (H Goudarzi PhD), Hokkaido University,

Sapporo, Japan; Department of Public Health and Preventive Medicine (Prof M Grivna PhD), Charles University, Prague, Czech Republic; Department of Clinical Science (M I M Gubari PhD), University Of Sulaimani, Sulaimani, Iraq; Harrington Heart and Vascular Institute (A Guha MD), Department of Nutrition and Preventive Medicine (Prof J Sanabria MD), Department of Pediatrics (S Sankararaman MD), Case Western Reserve University, Cleveland, OH, USA; Division of Cardiovascular Medicine (A Guha MD), Ohio State University, Columbus, OH, USA; Department of Community Medicine (D A Gunawardane MD), University of Peradeniya, Kandy, Sri Lanka; Department of Nephrology (A K Gupta PharmD), Max Super Speciality Hospital, New Delhi, India; Department of Public Health (B Gupta PhD), Torrens University Australia, Melbourne, VIC, Australia; Department of Cardiology (R Gupta MBBS), Lehigh Valley Health Network, Allentown, PA, USA; Department of Toxicology (S Gupta MSc), Shriram Institute for Industrial Research, Delhi, India; School of Medicine (V Gupta PhD), Deakin University, Geelong, VIC, Australia; Faculty of Medicine Health and Human Sciences (Prof V K Gupta PhD), Macquarie University, Sydney, NSW, Australia; Department of Radiology (A Haj-Mirzaian MD), Massachusetts General Hospital, Boston, MA, USA; Department of Epidemiology (A J Handal PhD), University of Michigan School of Public Health, Ann Arbor, MI, USA; Sakarya University, Turkey, Sakarya, Turkiye (A Hanif PhD); Centre for Neuromuscular and Neurological Disorders (Prof G J Hankey MD), Dobney Hypertension Centre (Prof M P Schlaich MD), The University of Western Australia, Perth, WA, Australia; Stroke Research Centre (Prof G J Hankey MD), Perron Institute for Neurological and Translational Science, Perth, WA, Australia; Medical Research Unit (H Harapan PhD), Universitas Syiah Kuala (Syiah Kuala University), Banda Aceh, Indonesia; Department of Epidemiology Population Biostatistics and Health Promotion (A Hargono PhD), Faculty of Public Health (Prof S Martini PhD), Department of Health Policy and Administration (R D Wulandari DrPH), Universitas Airlangga (Airlangga University), Surabaya, Indonesia; Research Unit (J M Haro MD), Parc Sanitari Sant Joan de Deu, Barcelona, Spain; Department of Mental Health (J M Haro MD), Carlos III Health Institute (Prof R Tabarés-Seisdedos PhD), Biomedical Research Networking Center for Mental Health Network (CiberSAM), Madrid, Spain; Department of Zoology and Entomology (A I Hasaballah PhD), Al-Azhar University, Cairo, Egypt; Institute for Social Science Research (M Hasan MPH), ARC Centre of Excellence for Children and Families over the Life Course (M Hasan MPH), The University of Queensland, Indooroopilly, QLD, Australia; Department of Public Health (A Hashi PhD), Jigjiga University, Jigjiga, Ethiopia; Skaane University Hospital (R J Havmoeller PhD), Skaane County Council, Malmö, Sweden; Institute of Pharmaceutical Sciences (K Hayat MS), University of Veterinary and Animal Sciences, Lahore, Pakistan; Department of Pharmacy Administration and Clinical Pharmacy (K Hayat MS), Xian Jiaotong University, Xian, China; Department of Epidemiology (M Heidari-Foroozan BSc, S Khanmohammadi MD, S Nejadghaderi MD), Non-Communicable Diseases Research Center (NCDRC), Tehran, Iran; Department of Statistics and Econometrics (Prof C Herteliu PhD, A Mirica PhD, A Otoiu PhD, I Petcu PhD), Bucharest University of Economic Studies, Bucharest, Romania; Babes-Bolyai University, Cluj-Napoca, Romania (Prof C Herteliu PhD); Department of Public Health (D Z Heyi MPH), Madda Walabu University, Robe, Ethiopia; Department of Microbiology (K Hezam PhD), Taiz University, Taiz, Yemen; School of Medicine (K Hezam PhD), Nankai University, Tianjin, China; Graduate School of Medicine (Y Hiraike PhD), University of Tokyo, Tokyo, Japan; School of Social Sciences (P Hoogar PhD), The Apollo University, Chittoor, India; Maternal and Child Health Division (MCHD) (S J Hossain MPH, A Sayeed MSc), Nutrition and Clinical Services Division (M Tariqujjaman MSc), International Centre for Diarrhoeal Disease Research, Bangladesh, Dhaka, Bangladesh; School of Computer Science (Prof M Hosseinzadeh PhD), Duy Tan University, Da Nang, Vietnam; Jadara University Research Center (Prof M Hosseinzadeh PhD), Jadara

University, Irbid, Jordan; Department of Clinical Legal Medicine (Prof S Hostiuc PhD), National Institute of Legal Medicine Mina Minovici, Bucharest, Romania; Burn Research Center (S Hoveidamanesh MD), Shahid Motahari Hospital, Tehran, Iran; Faculty of Medicine (J Huang MD), The Chinese University of Hong Kong, Hong Kong, China; Czech National Centre for Evidence-Based Healthcare and Knowledge Translation (S Hussain PhD), Institute of Biostatistics and Analyses (S Hussain PhD), Department of Public Health (A Riad PhD), Czech National Centre for Evidence-based Healthcare and Knowledge Translation (A Riad PhD), Masaryk University, Brno, Czech Republic; Department of Occupational Safety and Health (Prof B Hwang PhD), China Medical University, Taiwan, Taichung, Taiwan; Department of Occupational Therapy (Prof B Hwang PhD), Asia University, Taiwan, Taichung, Taiwan; Department of Epidemiology and Prevention (Prof L Iacoviello MD), IRCCS - Istituto di Ricerche Farmacologiche Mario Negri, Pozzilli, Italy; Department of Medicine and Surgery (Prof L Iacoviello MD), LUM University, Casamassima, Italy; Health Policy and Management Department (P M Iftikhar MD), City University of New York, New York, NY, USA; West Africa RCC (O S Ilesanmi PhD), Africa Centre for Disease Control and Prevention, Abuja, Nigeria; Department of Community Medicine (O S Ilesanmi PhD), University College Hospital, Ibadan, Ibadan, Nigeria; Faculty of Medicine (I M Ilic PhD, Prof M M Santric-Milicevic PhD), School of Public Health and Health Management (Prof M M Santric-Milicevic PhD), School of Medicine (R Vukovic PhD), University of Belgrade, Belgrade, Serbia; Faculty of Medical Sciences (Prof M D Ilic PhD), University of Kragujevac, Kragujevac, Serbia; Department of Health Research (L R Inbaraj MD), ICMR National Institute for Research in Tuberculosis, Chennai, India; Institute for Physical Activity and Nutrition (S Islam PhD), Deakin University, Burwood, VIC, Australia; Department of Surveillance and Health Equity Science (F Islami PhD), American Cancer Society, Atlanta, GA, USA; Department of Clinical Pharmacy & Pharmacy Practice (Prof N Ismail PhD), Asian Institute of Medicine, Science and Technology, Bedong, Malaysia; Malaysian Academy of Pharmacy, Puchong, Malaysia (Prof N Ismail PhD); Public Health Department of Social Medicine (Prof H Iso MD), Osaka University, Suita, Japan; Department of Health Services Research (M Iwagami PhD), Department of Public Health Medicine (Prof K Yamagishi MD), University of Tsukuba, Tsukuba, Japan; Department of Non-Communicable Disease Epidemiology (M Iwagami PhD), London School of Hygiene & Tropical Medicine, London, UK; Department of Orthodontics & Dentofacial Orthopedics (L J BDS), Dr. D. Y. Patil University, Pune, India; Department of Physical and Medicine (L Jacob MD), Université Paris Cité, Paris, France; Research and Development Unit (L Jacob MD), Biomedical Research Networking Center for Mental Health Network (CiberSAM), Barcelona, Spain; College of Medicine and Medical Sciences (H Jahrami PhD), Arabian Gulf University, Manama, Bahrain; Ministry of Health, Manama, Bahrain (H Jahrami PhD); The World Academy of Sciences UNESCO, Trieste, Italy (Prof M Jakovljevic PhD); Shaanxi University of Technology, Hanzhong, China (Prof M Jakovljevic PhD); School of Pharmaceutical Management (Prof M D Janodia PhD), IIHMR University, Jaipur, India; Department of Public Health (Prof K Jayanna MD), M S Ramaiah University of Applied Sciences, Bangalore, India; Institute for Global Public Health (Prof K Jayanna MD), University of Manitoba, Winnipeg, MB, Canada; Centre of Studies and Research (S Jayapal PhD), Ministry of Health, Muscat, Oman; Department of Biochemistry (Prof S Jayaram MD), Government Medical College, Mysuru, India; Department of Public Health (A Jema MPH), Madda Walabu University, Goba, Ethiopia; Department of Internal Medicine (B M Jeswani MBBS), GCS Medical College, Hospital & Research Centre, Ahmedabad, India; Rothschild Foundation Hospital (Prof J B Jonas MD), Institute of Molecular and Clinical Ophthalmology Basel, Paris, France; Singapore Eye Research Institute, Singapore, Singapore (Prof J B Jonas MD); Department of Gastroenterology and Hepatology (A Joseph MD), Department of Radiology (S Ramasamy MD), Stanford University, Stanford, CA, USA; Department of Community Medicine (N

Joseph MD, N Kumar MD, P Mithra MD, R Thapar MD), Department of Forensic Medicine and Toxicology (Prof J Padubidri MD, P H Shetty MD), Manipal Academy of Higher Education, Mangalore, India; Department of Economics (C E Joshua BSc), National Open University, Benin City, Nigeria; Department of Family Medicine and Public Health (J J Jozwiak PhD), University of Opole, Opole, Poland; Institute of Family Medicine and Public Health (M Jürisson PhD), University of Tartu, Tartu, Estonia; School of Public Health (Z Kabir PhD), University College Cork, Cork, Ireland; Department of Community Medicine and Family Medicine (B D Kamble MD), All India Institute of Medical Sciences, Hyderabad, India; Department of Community Medicine (B D Kamble MD), Banaras Hindu University, Varanasi, India; Sydney Eye Hospital (H Kandel PhD), South Eastern Sydney Local Health District, Sydney, NSW, Australia; School of Health Professions and Human Services (I M Karaye MD), Hofstra University, Hempstead, NY, USA; Department of Anesthesiology (I M Karaye MD), Montefiore Medical Center, Bronx, NY, USA; Centre for Tropical Diseases and Global Health (Prof P D Katoto PhD), Catholic University of Bukavu, Bukavu, Democratic Republic of the Congo; Department of Global Health (Prof P D Katoto PhD), Department of Epidemiology (J L J Tamuzi MSc), Stellenbosch University, Cape Town, South Africa; Surgery Research Unit (Prof J H Kauppila MD), University of Oulu, Oulu, Finland; Public Health Foundation of India, New Delhi, India (H Kaur MPH); International Research Center of Excellence (G A Kayode PhD), Institute of Human Virology Nigeria, Abuja, Nigeria; Julius Centre for Health Sciences and Primary Care (G A Kayode PhD), Utrecht University, Utrecht, Netherlands; Institute of Biological Chemistry and Nutrition (T S Keflie PhD), University Hohenheim, Stuttgart, Germany; Centre for Adolescent Health (J A Kerr PhD), Murdoch Childrens Research Institute, Parkville, VIC, Australia; Department of Psychological Medicine (J A Kerr PhD), University of Otago, Christchurch, New Zealand; Amity Institute of Forensic Sciences (H Khajuria PhD, B P Nayak PhD), Amity University, Noida, India; College of Health Sciences (N Khalid PhD), Abu Dhabi University, Adu Dhabi, United Arab Emirates; Population Science Department (M Khan PhD), Jatiya Kabi Kazi Nazrul Islam University, Mymensingh, Bangladesh; Primary Care Department (M A Khan MSc), NHS North West London, London, UK; Department of Radiation Oncology (T Khan PhD), Division of Cardiology (J Noubiap MD), Department of Epidemiology and Biostatistics (M Teramoto MD), Department of Bioengineering and Therapeutical Sciences (Prof M Zastrozhin PhD), University of California San Francisco, San Francisco, CA, USA; Department of Clinical Pharmacy (Y H Khan PhD, T Mallhi PhD), Jouf University, Sakaka, Saudi Arabia; College of Health, Wellbeing and Life Sciences (Prof K Khatab PhD), Sheffield Hallam University, Sheffield, UK; College of Arts and Sciences (Prof K Khatab PhD), Ohio University, Zanesville, OH, USA; Department of Basic Medical Sciences (Prof M M Khatatbeh PhD), Yarmouk University, Irbid, Jordan; School of Medicine (S Khateri MD), Department of Epidemiology and Biostatistics (Y Moradi PhD), Kurdistan University of Medical Sciences, Sanandaj, Iran; Global Consortium for Public Health Research (Prof M Khatib PhD), Datta Meghe Institute of Higher Education and Research, Wardha, India; Department of Public Health (J Khubchandani PhD), New Mexico State University, Las Cruces, NM, USA; Institute of Health Services Research (G Kim PhD), Yonsei University, Seoul, South Korea; Millennium Prevention, Inc., Westwood, MA, USA (R W Kimokoti MD); School of Health Sciences (Prof A Kisa PhD), Kristiania University College, Oslo, Norway; Department of International Health and Sustainable Development (Prof A Kisa PhD), Tulane University, New Orleans, LA, USA; Department of Nursing and Health Promotion (S Kisa PhD), Oslo Metropolitan University, Oslo, Norway; Department of General Practice and Family Medicine (Prof O Korzh DSc), Kharkiv National Medical University, Kharkiv, Ukraine; Kasturba Medical College, Manipal (S Koulmane Laxminarayana MD), Manipal Academy of Higher Education, Udupi, India; San Juan de Dios Sanitary Park, Barcelona, Spain (A Koyanagi MD); Department of Anthropology (Prof K Krishan PhD), Panjab University,

Chandigarh, India; Department of Anesthesiology (V Krishnamoorthy MD), Duke University, Durham, NC, USA; Department of Demography (Prof B Kuate Defo PhD), Department of Social and Preventive Medicine (Prof B Kuate Defo PhD), University of Montreal, Montreal, QC, Canada; Faculty of Medicine (B Kucuk Bicer PhD), Gazi University, Ankara, Turkiye; Department of Biochemistry (Prof M Kuddus PhD), University of Hail, Hail, Saudi Arabia; Department of Psychiatry (M Kumar PhD), University of Nairobi, Nairobi, Kenya; Department of Clinical Subjects (A Kurmanova MD), Al Farabi Kazakh National University, Almaty, Kazakhstan; Department of Health Services Research and Management (D Kusuma DSc), City University of London, London, UK; Faculty of Public Health (D Kusuma DSc), Centre for Family Welfare (K Latief PhD), University of Indonesia, Depok, Indonesia; National Institute for Health Research (NIHR) Oxford Biomedical Research Centre, Oxford, UK (B Lacey DPhil); Department of Medical Sciences (Prof A O Larsson PhD), Uppsala University, Uppsala, Sweden; Department of Clinical Chemistry and Pharmacology (Prof A O Larsson PhD), Uppsala University Hospital, Uppsala, Sweden; Department of Global Health and Health Security (K Latief PhD), Taipei Medical University, Taipei, Taiwan; Pattern Recognition and Machine Learning Lab (Prof S Lee PhD), Gachon University, Seongnam, South Korea; Department of Family Medicine (W Lee PhD), University of Texas Medical Branch, Galveston, TX, USA; Department of Biomedical and Neuromotor Sciences (J Lenzi PhD), University of Bologna, Bologna, Italy; Department of Health Promotion and Health Education (M Li PhD), National Taiwan Normal University, Taipei, Taiwan; Department of Internal Medicine (C Lo MD), Kirk Kerkorian School of Medicine at UNLV, Las Vegas, NV, USA; Department of Paediatrics (Prof R Lodha MD), All India Institute of Medical Sciences, New Delhi, India; School of Medicine and Public Health (A Loreche BS), Center for Research and Innovation (V F Pepito MSc), Ateneo De Manila University, Pasig City, Philippines; Department of Health Economics (L Lorenzovici MSc), Syreon Research Romania, Targu Mures, Romania; Department of Doctoral Studies (L Lorenzovici MSc), George Emil Palade University of Medicine, Pharmacy, Science, and Technology of Targu Mures, Targu Mures, Romania; Institute of Nutritional Sciences (Prof S Lorkowski PhD), Friedrich Schiller University Jena, Jena, Germany; Competence Cluster for Nutrition and Cardiovascular Health (nutriCARD), Jena, Germany (Prof S Lorkowski PhD); Department of Biostatistics and Epidemiology (F Madadizadeh PhD), Yazd University of Medical Sciences, Yazd, Iran; Laboratório de Farmacognosia (LAQV) (Associated Laboratory for Green Chemistry (Á M Madureira-Carvalho PhD), Universidade do Porto (University of Porto), Porto, Portugal; Department of Community Medicine (P B Mahajan MD), Jawaharlal Institute of Postgraduate Medical Education and Research, Karaikal, India; Cyprus International Institute for Environmental and Public Health (Prof K C Makris PhD), Cyprus University of Technology, Limassol, Cyprus; Department of Biochemistry (A Marjani PhD), Golestan University of Medical Sciences, Gorgan, Iran; Indonesian Public Health Association, Surabaya, Indonesia (Prof S Martini PhD); Department of Nutrition and Dietetics (M Martorell PhD), Centre for Healthy Living (M Martorell PhD), University of Concepción, Concepción, Chile; Department of Public Health and Community Medicine (E Mathews PhD), Central University of Kerala, Kasaragod, India; Department of Social Medicine and Family (M Mazaheri PhD), Dezful University of Medical Sciences, Dezful, Iran; Division of Pediatric Hospital Medicine (R P Mediratta MD), Stanford University, Palo Alto, CA, USA; Neurology Department (Prof M Mehndiratta MD), Janakpuri Super Specialty Hospital Society, New Delhi, India; Department of Neurology (Prof M Mehndiratta MD), Govind Ballabh Institute of Medical Education and Research, New Delhi, India; Universidad Nacional Mayor de San Marcos, Lima, Peru (W Mendoza MD); Division of Forensic Medicine (Prof R G Menezes MD), Imam Abdulrahman Bin Faisal University, Dammam, Saudi Arabia; Center for Translation Research and Implementation Science (G A Mensah MD), National Institutes of Health, Bethesda, MD, USA; Department of Medicine (G A Mensah

MD), Technical Department (C A Nnaji PhD), School of Public Health and Family Medicine (C A Nnaji PhD), University of Cape Town, Cape Town, South Africa; International Dx Department (A A Mentis MD), BGI Genomics, Copenhagen, Denmark; Comprehensive Cancer Center (T J Meretoja MD), Helsinki University Hospital, Helsinki, Finland; University of Helsinki, Helsinki, Finland (T J Meretoja MD); University Centre Varazdin (T Mestrovic PhD), University North, Varazdin, Croatia; Department of Propedeutics of Internal Diseases & Arterial Hypertension (Prof T Miazgowski MD), Pomeranian Medical University, Szczecin, Poland; Pacific Institute for Research & Evaluation, Calverton, MD, USA (T R Miller PhD); Department of Public Health Dentistry (Prof G Mini PhD), Saveetha Institute of Medical and Technical Sciences (SIMATS), Chennai, India; Global Institute of Public Health (Prof G Mini PhD), Ananthapuri Hospitals and Research Institute, Trivandrum, India; Faculty of Nursing and Midwifery (Prof M Mirghafourvand PhD), Tabriz University of Medical Sciences, Tabriz, Iran; Internal Medicine Programme (Prof E M Mirrakhimov PhD), Kyrgyz State Medical Academy, Bishkek, Kyrgyzstan; Department of Atherosclerosis and Coronary Heart Disease (Prof E M Mirrakhimov PhD), National Center of Cardiology and Internal Disease, Bishkek, Kyrgyzstan; Department of Medical Microbiology (K A Mohammad PhD), Knowledge University, Erbil, Iraq; Health Systems and Policy Research Unit (Prof S Mohammed PhD), Department of Community Medicine (M Sufiyan MD), Ahmadu Bello University, Zaria, Nigeria; Department of Health Services Management (M Mohseni PhD), Iran University of Medical Sciences, Iran, Iran; Clinical Epidemiology and Public Health Research Unit (L Monasta DSc, G Zamagni MSc), Burlo Garofolo Institute for Maternal and Child Health, Trieste, Italy; AI & Cyber Futures Institute (M Moni PhD), Charles Sturt University, Bathurst, NSW, Australia; The University of Queensland, Brisbane, QLD, Australia (M Moni PhD); Division of Plastic and Reconstructive Surgery (S D Morrison MD), University of Washington Medical Center, Seattle, WA, USA; Unit of Pharmacotherapy, Epidemiology and Economics (Prof S Mubarik PhD), University of Groningen (Rijksuniversiteit Groningen), Groningen, Netherlands; Department of Epidemiology and Biostatistics (Prof S Mubarik PhD, Prof C Yu PhD), Wuhan University, Wuhan, China; Demographic Change and Aging Research Area (A Werdecker PhD), Federal Institute for Population Research, Wiesbaden, Germany (Prof U O Mueller MD); Center for Population and Health, Wiesbaden, Germany (Prof U O Mueller MD); Department of Surgery (F Mulita PhD), General University Hospital of Patras, Patras, Greece; Faculty of Medicine (F Mulita PhD), University of Thessaly, Larissa, Greece; Department of Paediatrics and Paediatric Infectious Diseases (Prof D Munblit PhD), I.M. Sechenov First Moscow State Medical University, Moscow, Russia; National Heart & Lung Institute (Prof D Munblit PhD), Department of Primary Care and Public Health (Prof S Rawaf MD), Department of Epidemiology and Biostatistics (Prof A Rodriguez PhD), The George Institute for Global Health (Prof S Yaya PhD), Imperial College London, London, UK; Clinical Epidemiology Research Unit (E Murillo-Zamora PhD), Mexican Institute of Social Security, Villa de Alvarez, Mexico; Postgraduate in Medical Sciences (E Murillo-Zamora PhD), Universidad de Colima, Colima, Mexico; Department of Pediatrics & Pediatric Pulmonology (Prof G Mustafa MD), Institute of Mother & Child Care, Multan, Pakistan; Research and Analytics Department (A J Nagarajan MTech), Initiative for Financing Health and Human Development, Chennai, India; Department of Research and Analytics (A J Nagarajan MTech), Bioinsilico Technologies, Chennai, India; Suraj Eye Institute, Nagpur, India (V Nangia MD); Mysore Medical College and Research Institute (Prof S Narasimha Swamy MD), Government Medical College, Mysore, India; HIV/STI Surveillance Research Center (S Nejadghaderi MD), Kerman University of Medical Sciences, Kerman, Iran; Department of Public Health (G Nguefack-Tsague PhD), University of Yaoundé I, Yaoundé, Cameroon; Department of Biological Sciences (J W Ngunjiri PhD), University of Embu, Embu, Kenya; Hitotsubashi Institute for Advanced Study (HIAS) (P T Nguyen DrPH),

Hitotsubashi University, Tokyo, Japan; Institute for Cancer Control (P T Nguyen DrPH), National Cancer Center, Chuo-ku, Japan; International Islamic University Islamabad, Islamabad, Pakistan (R K Niazi PhD); Department of Gastroenterology (N M Noor MRCP), Cambridge University Hospitals, Cambridge, UK; Department of Paediatrics (C A Nri-Ezedi PhD), Nnamdi Azikiwe University, Awka, Nigeria; Department of Public Health (D Nurrika PhD), Banten School of Health Science, South Tangerang, Indonesia; Ministry of Research, Technology and Higher Education (D Nurrika PhD), Higher Education Service Institutions (LL-DIKTI) Region IV, Bandung, Indonesia; Department of Pediatrics (V E Nwatah MD), National Hospital Abuja, Abuja, Nigeria; Department of International Public Health (V E Nwatah MD), University of Liverpool, Liverpool, UK; Department of Applied Economics and Quantitative Analysis (Prof B Oancea PhD), University of Bucharest, Bucharest, Romania; School of Health Systems & Public Health (R E Ogunsakin PhD), University of Pretoria, Pretoria, South Africa; Department of Food and Nutrition (A P Okekunle PhD), Seoul National University, Seoul, South Korea; Department of Agribusiness and Natural Resource Economics (D M Okello PhD), Kabale University, Kabale, Uganda; Department of Rural Development and Agribusiness (D M Okello PhD), Gulu University, Gulu, Uganda; School of Pharmacy (O C Okonji MSc), University of the Western Cape, Cape Town, South Africa; Department of Psychiatry (A T Olagunju MD), University of Lagos, Lagos, Nigeria; Cardiology Department (G M M Oliveira PhD), Federal University of Rio de Janeiro, Rio de Janeiro, Brazil; Department of Research Policy & Administration (J O Olusanya MBA), Centre for Healthy Start Initiative, Lagos, Nigeria (B O Olusanya PhD); Department of Public Health (S Ong FAMS), Ministry of Health, Bandar Seri Begawan, Brunei; Institute of Health Sciences (S Ong FAMS), Universiti Brunei Darussalam, Bandar Seri Begawan, Brunei; Department of Medicine (Prof A Ortiz MD), Universidad Autónoma de Madrid (Autonomous University of Madrid), Madrid, Spain; Department of Nephrology and Hypertension (Prof A Ortiz MD), The Institute for Health Research Foundation Jiménez Díaz University Hospital, Madrid, Spain; Department of Nutrition and Public Health (Prof S M Ostojic PhD), University of Agder, Kristiansand, Norway; Department of Biomedical Sciences (Prof S M Ostojic PhD), University of Novi Sad, Novi Sad, Serbia; Department of Public Health (A Oumer PhD, Y M Tefera MPH), Department of Nursing (Y Solomon MSc), Department of Nutrition (Z Tariku MPH), Dire Dawa University, Dire Dawa, Ethiopia; National School of Public Health (A Padron-Monedero PhD), Institute of Health Carlos III, Madrid, Spain; Department of Public Health (A Pana PhD), Babes Bolyai University, Cluj Napoca, Romania; Department of Health Metrics (A Pana PhD), Center for Health Outcomes & Evaluation, Bucharest, Romania; Centre for Research and Development (Prof S R Pandi-Perumal MSc), Chandigarh University, Punjab, India; Division of Research and Development (Prof S R Pandi-Perumal MSc), Lovely Professional University, Phagwara, India; Department of Science and Mathematics (Prof P Papadopoulou PhD), Deree-The American College of Greece, Athens, Greece; Department of Biophysics (Prof P Papadopoulou PhD), University of Athens, Athens, Greece; Vision and Eye Research Institute (Prof S Pardhan PhD), Anglia Ruskin University, Cambridge, UK; Global Health Governance Programme (J Patel BSc), University of Edinburgh, Edinburgh, UK; Research Consultancy (A R Pathan PhD), Author Gate Publications, Malegaon, India; Health, Nutrition, and HIV/AIDS Program (D Paudel PhD), Save the Children, Kathmandu, Nepal; Center for International Health (D Paudel PhD), Ludwig Maximilians University, Munich, Germany; School of Population Health (Prof G Pereira PhD), Curtin University, Bentley, WA, Australia; Centre for Fertility and Health (Prof G Pereira PhD), Norwegian Institute of Public Health, Oslo, Norway; Institute of Collective Health (Prof M Pereira PhD), Federal University of Bahia, Salvador, Brazil; Mario Negri Institute for Pharmacological Research, Bergamo, Italy (N Perico MD, Prof G Remuzzi MD); Facultad de Medicina (F E Petermann-Rocha PhD), Universidad Diego Portales (Diego Portales University), Santiago, Chile; School

of Cardiovascular and Metabolic Health (F E Petermann-Rocha PhD), University of Glasgow, Glasgow, UK; International Center of Medical Sciences Research, Islamabad, Pakistan (Z Z Piracha PhD); Department of Neonatology (N Plakkal MD), Department of Preventive and Social Medicine (G Saya MD), Jawaharlal Institute of Postgraduate Medical Education and Research, Puducherry, India; Non-communicable Diseases Research Center (N Pourtaheri PhD), Bam University of Medical Sciences, Bam, Iran; College of Medicine (A Radfar MD), University of Central Florida, Orlando, FL, USA; Avicenna Medical and Clinical Research Institute, Encino, CA, USA (A Radfar MD); Department of Medical Oncology (Prof V Radhakrishnan MD), Cancer Institute (W.I.A), Chennai, India; Department of Medical Laboratory Technologies (Prof F Rahim PhD), Al-Noor Center of Research and Innovation (Prof F Rahim PhD), Alnoor University, Mousl, Iraq; Data Mining Research Unit (DaMRA) (A Rahman PhD), Charles Sturt University, Wagga Wagga, NSW, Australia; Department of Population Science and Human Resource Development (Prof M Rahman PhD, Prof M Rahman DrPH), University of Rajshahi, Rajshahi, Bangladesh; National Institute of Infectious Diseases (M Rahman PhD), Center for Surveillance, Immunization, and Epidemiologic Research, Tokyo, Japan; Center for Evidence-Based Medicine and Clinical Research, Dhaka, Bangladesh (M Rahman PhD); Institute of Health and Wellbeing (Prof M Rahman PhD), Federation University Australia, Berwick, VIC, Australia; School of Nursing and Midwifery (Prof M Rahman PhD), La Trobe University, Melbourne, VIC, Australia; Future Technology Research Center (A Rahmani PhD), National Yunlin University of Science and Technology, Yunlin, Taiwan; Department of Public Health (V Rahmanian PhD), Torbat Jam Faculty of Medical Sciences, Torbat Jam, Iran; Department of Nutrition Science (S Rahmawaty PhD), Muhammadiyah University of Surakarta, Surakarta, Indonesia; Society for Health and Demographic Surveillance, Suri, India (R Rai PhD); Institute of Nutrition (R Rai PhD), Mahidol University, Salaya, Thailand; Department of Medical, Surgical and Experimental Sciences (I Raimondo MD), University of Sassari, Sassari, Italy; Gynecology and Breast Care Center (I Raimondo MD), Mater Olbia Hospital (Qatar Foundation Endowment and Policlinico Universitario Agostino Gemelli IRCCS Foundation), Olbia, Italy; Department of Community Medicine (S Rajaa MD), Employees' State Insurance Model Hospital, Chennai, India; Centre for Chronic Disease Control, New Delhi, India (P Rajput PhD); School of Nursing & Health Sciences (S Ramazanu PhD), Hong Kong Metropolitan University, Hong Kong, China; Saw Swee Hock School of Public Health (S Ramazanu PhD), Department of Surgery (K Tan PhD), Yong Loo Lin School of Medicine (Prof N Venketasubramanian MSc), National University of Singapore, Singapore, Singapore; Department of Oral Pathology, Microbiology and Forensic Odontology (S Rao MDS), Sharavathi Dental College and Hospital, Shimogga, India; Data Analytic Services (D P Rasali PhD), British Columbia Centre for Disease Control, Vancouver, BC, Canada; School of Population and Public Health (D P Rasali PhD), University of British Columbia, Vancouver, BC, Canada; Department of Medicine (A M Rashid MD), Jinnah Sindh Medical University, Karachi, Pakistan; Baylor University, Dallas, TX, USA (A M Rashid MD); Department of Biomedical Engineering (Z Ratan MSc), Khulna University of Engineering and Technology, Khulna, Bangladesh; School of Health and Society (Z Ratan MSc), University of Wollongong, Wollongong, NSW, Australia; Academic Public Health England (Prof S Rawaf MD), Public Health England, London, UK; School of Health, Medical and Applied Sciences (L Rawal PhD), CQ University, Sydney, NSW, Australia; Department of Biological Sciences (Prof E M M Redwan PhD), King Abdulaziz University, Jeddah, Egypt; Department of Protein Research (Prof E M M Redwan PhD), Research and Academic Institution, Alexandria, Egypt; Saveetha Dental College and Hospitals (K Rengasamy PhD, M Tovani-Palone PhD), Center for Global Health Research (Prof A Sahebkar PhD), Saveetha University, Chennai, India; Centre for Excellence in Pharmaceutical Sciences (K Rengasamy PhD), North-West University, Potchefstroom, South Africa; School of Medicine (Prof A M N Renzaho

PhD), Translational Health Research Institute (Prof A M N Renzaho PhD), Western Sydney University, Campbelltown, NSW, Australia; Department of Epidemiology and Biostatistics (Prof M Rezaeian PhD), Department of Neurology (A Vakilian MD), Non-communicable Diseases Research Center (A Vakilian MD), Rafsanjan University of Medical Sciences, Rafsanjan, Iran; Department of Surgery (J Rickard MD), University of Minnesota, Minneapolis, MN, USA; Department of Surgery (J Rickard MD), University Teaching Hospital of Kigali, Kigali, Rwanda; Wolfson Institute of Population Health (Prof A Rodriguez PhD), Queen Mary University of London, London, UK; Department of Pharmacology and Toxicology (Prof J A B Rodriguez PhD), University of Antioquia, Medellin, Colombia; Department of Clinical Research (Prof L Roever PhD), University of Sao Paulo, Ribeirão Preto, Brazil; Center for Indigenous Health Research (P Rohloff MD), Wuqu' Kawoq Maya Health Alliance, Tecpan, Guatemala; Faculty of Medicine (B Roy PhD), Quest International University Perak, Ipoh, Malaysia; Department of Internal Medicine (G M Rwegerera MD), University of Botswana, Gaborone, Botswana; Department of Oral and Maxillofacial Surgery (C S N PhD), Jagadguru Sri Shivarathreeswara University, Mysore, India; Cardiovascular Department (Prof A M A Saad MD), Zagazig University, Zagazig, Egypt; Department of Medical Pharmacology (Prof M M Saber-Ayad PhD), Public Health and Community Medicine Department (M R Salem MD), Cairo University, Giza, Egypt; Department of Pharmaceuticals (Prof M Sachdeva Dhingra PhD), Bihar College of Pharmacy, Patna, India; Health Information Management (M Sadeghi PhD), Semnan University of Medical Sciences, Semnan, Iran; Clinical and Biomedical Research Center (Prof U Saeed PhD), Foundation University Islamabad, Islamabad, Pakistan; International Center of Medical Sciences Research (ICMSR), Islamabad, Pakistan (Prof U Saeed PhD); Kiel Institute for the World Economy, Kiel, Germany (S Saeedi Moghaddam MSc); Faculty of Medicine, Bioscience and Nursing (S Safi PhD), MAHSA University, Selangor, Malaysia; Interdisciplinary Research Centre in Biomedical Materials (IRCBM) (S Safi PhD), COMSATS Institute of Information Technology, Lahore, Pakistan; Department of Family and Generations (H Sahoo PhD), International Institute for Population Sciences, Mumbai, India; Department of Statistics (M R Sajid PhD), University of Gujrat, Gujrat, Pakistan; Department of Entomology (A M Samy PhD), Medical Ain Shams Research Institute (MASRI) (A M Samy PhD), Ain Shams University, Cairo, Egypt; Department of Pediatrics (Prof R K Sanjeev MD), SRM University, Chennai, India; Department of Pediatrics (S Sankararaman MD), University Hospitals Rainbow Babies & Children's Hospital, Cleveland, OH, USA; Independent Consultant, Thiruvananthapuram, India (S Y Saraswathy PhD); Department of Public Health (Y Sarikhani PhD), Jahrom University of Medical Sciences, Jahrom, Iran; UGC Centre of Advanced Study in Psychology (M Satpathy PhD), Utkal University, Bhubaneswar, India; Udyam-Global Association for Sustainable Development, Bhubaneswar, India (M Satpathy PhD); Department of Public Health Sciences (M Sawhney PhD), University of North Carolina at Charlotte, Charlotte, NC, USA; Department of Post-Harvest Technology and Marketing (A Sayeed MSc), Patuakhali Science and Technology University, Patuakhali, Bangladesh; Department of Neurology (Prof N Scarmeas PhD), National and Kapodistrian University of Athens, Athens, Greece; Department of Neurology (Prof N Scarmeas PhD), Columbia University, New York, NY, USA; Cardiovascular Program (X Xu PhD), The George Institute for Global Health, Sydney, NSW, Australia (Prof A E Schutte PhD); Emergency Department (S Senthilkumaran PhD), Manian Medical Centre, Erode, India; Fourth Department of General Surgery (D Serban PhD), Emergency University Hospital Bucharest, Bucharest, Romania; National Heart, Lung, and Blood Institute (A Seylani BS), National Institutes of Health, Rockville, MD, USA; Department of Microbiology (P A Shah MBBS), Rajiv Gandhi University of Health Sciences, Bangalore, India; Independent Consultant, Karachi, Pakistan (M A Shaikh MD); Department of Public Health (A T T Shama MPH), Wollega University, Nekemt, Ethiopia; School of Medicine (M Shams-Beyranvand MSc), Alborz University of Medical Sciences, Karaj,

Iran; College of Nursing and Health Sciences (M Shanawaz MD), Jazan University, Jazan, Saudi Arabia; Finnish Institute of Occupational Health, Helsinki, Finland (R Shiri PhD); Department of Experimental Research (V Shivarov PhD), Medical University Pleven, Sofia, Bulgaria; Department of Genetics (V Shivarov PhD), Sofia University "St. Kliment Ohridiski", Sofia, Bulgaria; Department of Medical-Surgical Nursing (S Shorofi PhD), Mazandaran University of Medical Sciences, Sari, Iran; The Cooper Institute, Dallas, TX, USA (K Shuval PhD); Department of Pediatrics and Child Health Nursing (M M Sibhat MSc), Dilla University, Dilla, Ethiopia; Sport Physical Activity and Health Research & Innovation Center (SPRINT) (Prof L M R Silva PhD), Polytechnic Institute of Guarda, Guarda, Portugal; CICS-UBI Health Sciences Research Center (Prof L M R Silva PhD), University of Beira Interior, Covilhã, Portugal; School of Medicine (Prof J A Singh MD), Baylor College of Medicine, Houston, TX, USA; Department of Medicine Service (Prof J A Singh MD), US Department of Veterans Affairs (VA), Houston, TX, USA; Faculty of Medicine and Health Sciences (Prof N P Singh MD), Shree Guru Gobind Singh Tricentenary University, Gurugram, India; Department of Infectious Diseases and Epidemiology (A A Skryabina MD), Pirogov Russian National Research Medical University, Moscow, Russia; Institute of Child and Adolescent Health (Y Song PhD, Prof Z Zou MD), Peking University, Beijing, China; School of Life Sciences (M Suleman PhD), Xiamen University, Xiamen, China; Institute of Integrated Intelligence and Systems (Prof J Sun PhD), Griffith University, Brisbane, QLD, Australia; Department of Research and Development (D R Sunuwar MSc), Armed Police Force Hospital, Kathmandu, Nepal; Department of Public Health (D R Sunuwar MSc), Asian College for Advance Studies, Purbanchal University, Lalitpur, Nepal; Northwestern University, Chicago, IL, USA (M D Szeto MS); Department of Medicine (Prof R Tabarés-Seisdedos PhD), University of Valencia, Valencia, Spain; Department of Basic Medical Sciences (S Tabatabaeizadeh PhD), Department of Internal Medicine (S Tabatabaeizadeh PhD), Islamic Azad University, Mashhad, Iran; Department of Biostatistics and Epidemiology (M Taheri Soodejani PhD), Shahid Sadoughi University of Medical Sciences, Yazd, Iran; Department of Medicine (J L J Tamuzi MSc), Northlands Medical Group, Omuthiya, Namibia; School of Nursing and Public Health (Prof E E Tarkang PhD), University of KwaZulu-Natal, Durban, South Africa; Department of Economics (N Y Tat MS), Rice University, Houston, TX, USA; Department of Research and Innovation (N Y Tat MS), Enventure Medical Innovation, Houston, TX, USA; Department of Pharmacology (P Thangaraju MD), All India Institute of Medical Sciences, Raipur, India; Clinical Epidemiology (A Thiyagarajan MPH), Leibniz Institute for Prevention Research and Epidemiology, Bremen, Germany; Faculty of Biomedical Engineering (A Tichopad PhD), Czech Technical University, Prague, Czech Republic; Faculty of Public Health (J H V Ticoalu MPH), Universitas Sam Ratulangi (Sam Ratulangi University), Manado, Indonesia; Nuffield Department of Primary Care Health Sciences (T Tillawi MD), Oxford University, Oxford, UK; Department of Allied Health and Human Performance (T Y Tiruye PhD), University of South Australia, Adelaide, SA, Australia; Public Health Department (T Y Tiruye PhD), Debre Markos University, Debre Markos, Ethiopia; Department of Medicine (Prof M Tonelli MD), Department of Oncology (L Yang PhD), University of Calgary, Calgary, AB, Canada; Institute of Public Health (R Topor-Madry PhD), Jagiellonian University Medical College, Kraków, Poland; Agency for Health Technology Assessment and Tariff System, Warsaw, Poland (R Topor-Madry PhD); Nutritional Epidemiology Research Team (EREN) (M Touvier PhD), National Institute for Health and Medical Research (INSERM), Paris, France; Department of Health, Medicine and Human Biology (M Touvier PhD), Sorbonne Paris Nord University, Bobigny, France; School of Medicine and Dentistry (M T N Tran PhD), Queensland University of Technology, GoldCoast, QLD, Australia; Health Informatics Department (M T N Tran PhD), Hanoi Medical University, Ha Noi, Vietnam; Department of Zoology (S Ullah PhD), Division of Science and Technology (S Ullah PhD), University of Education Lahore, Lahore, Pakistan; School of Government (E A

Undurraga PhD), Pontificia Universidad Catolica de Chile (Pontifical Catholic University of Chile), Santiago, Chile; Clinical Cancer Research Center (S Valadan Tahbaz PhD), Milad General Hospital, Tehran, Iran; Department of Microbiology (S Valadan Tahbaz PhD), Islamic Azad University, Tehran, Iran; Urmia University of Medical Sciences, Urmia, Iran (R Valizadeh PhD); Department of Cardiovascular Sciences (J Van den Eynde BSc), Katholieke Universiteit Leuven, Leuven, Belgium; UKK Institute, Tampere, Finland (Prof T J Vasankari PhD); Faculty of Medicine and Health Technology (Prof T J Vasankari PhD), Tampere University, Tampere, Finland; Raffles Neuroscience Centre (Prof N Venketasubramanian MSc), Raffles Hospital, Singapore, Singapore; Department of Health Care Administration and Economics (Prof V Vlassov MD), National Research University Higher School of Economics, Moscow, Russia; GBD Collaborating Unit (Prof S E Vollset DrPH), Norwegian Institute of Public Health, Bergen, Norway; Department of Pediatric Endocrinology (R Vukovic PhD), Mother and Child Healthcare Institute of Serbia "Dr Vukan Cupic", Belgrade, Serbia; NUST School of Health Sciences (Prof Y Waheed PhD), National University of Sciences and Technology (NUST), Islamabad, Pakistan; Operational Research Center in Healthcare (Prof Y Waheed PhD), Near East University, Nicosia, Turkiye; Brigham and Women's Hospital, Boston, MA, USA (C Wang PhD); School of Public Health (F Wang PhD), Xuzhou Medical University, Xuzhou, China; Department of Parasitology (Prof K G Weerakoon PhD), Department of Community Medicine (N D Wickramasinghe MD), Rajarata University of Sri Lanka, Anuradhapura, Sri Lanka; General Internal Medicine and Health Services Research (M Y Wei MD), University of California Los Angeles, Los Angeles, CA, USA; Department of Medicine (M Y Wei MD), Greater Los Angeles VA Healthcare System, Los Angeles, CA, USA; National Data Management Center for Health (NDMC) (A A Wolde MPH), Ethiopian Public Health Institute, Addis Ababa, Ethiopia; Department of Endocrinology (Prof S Xu PhD), University of Science and Technology of China, Hefei, China; Centre for Health Systems and Safety Research (L Yadav PhD), Australian Institute of Health Innovation, Macquarie University, Macquarie Park, NSW, Australia; Adelaide Medical School (L Yadav PhD), The University of Adelaide, Adelaide, SA, Australia; Department of Public Health (Prof K Yamagishi MD), Faculty of Medicine (Y Yano MD), Juntendo University, Tokyo, Japan; Department of Cancer Epidemiology and Prevention Research (L Yang PhD), Alberta Health Services, Calgary, AB, Canada; Department of Radiology (F Yazdanpanah MD), University of Pennsylvania, Philadelphia, PA, USA; Department of Health Management (A Yiğit PhD, V Yiğit PhD), Süleyman Demirel Üniversitesi (Süleyman Demirel University), Isparta, Turkiye; Department of Pediatrics (Prof D Yon MD), Kyung Hee University, Seoul, South Korea; Department of Health Sciences (S Zaman PhD), James Madison University, Harrisonburg, VA, USA; Sant'Elia Hospital (A Zanghì MD), University of Catania, Caltanissetta, Italy; Research and Development Department (I Zare BSc), Sina Medical Biochemistry Technologies, Shiraz, Iran; Department of Administration (Prof M Zastrozhin PhD), PGxAI, San Francisco, CA, USA

Authors' Contributions

Managing the overall research enterprise

Emmanuela Gakidou, Nicholas J Kassebaum, Christopher J L Murray, Robert C Reiner Jr, Amanda E Smith, and Stein Emil Vollset.

Writing the first draft of the manuscript

Michael Benjamin Arndt

Primary responsibility for applying analytical methods to produce estimates

Michael Benjamin Arndt, Natalia V Bhattacharjee, Julian Chalek, Sam Farmer, Ryan Fitzgerald, William M Gardner, Kyle Matthew Humphrey, Helena Manguerra, Catalina Raggi, and Reed J D Sorensen.

Primary responsibility for seeking, cataloguing, extracting, or cleaning data; designing or coding figures and tables

Michael Benjamin Arndt**,** Ryan Fitzgerald, Justin Lo, Helena Manguerra, Heather Jean Taylor, and Bethany Zigler.

Providing data or critical feedback on data sources

Yohannes Habtegiorgis Abate, Samar Abd ElHafeez, Rizwan Suliankatchi Abdulkader, Hassan Abidi, Richard Gyan Aboagye, Hassan Abolhassani, Yonas Derso Abtew, Niveen ME Abu-Rmeileh, Juan Manuel Acuna, Denberu Eshetie Adane, Qorinah Estiningtyas Sakilah Adnani, Saira Afzal, Antonella Agodi, Bright Opoku Ahinkorah, Sajjad Ahmad, Tauseef Ahmad, Ali Ahmadi, Ali Ahmed, Budi Aji, Hossein Akbarialiabad, Hanadi Al Hamad, Khalid F Alhabib, Robert Kaba Alhassan, Beriwan Abdulqadir Ali, Syed Shujait Ali, Syed Mohamed Aljunid, Sami Almustanyir, Alaa B Al-Tammemi, Nelson Alvis-Guzman, Nelson J Alvis-Zakzuk, Edward Kwabena Ameyaw, Hubert Amu, Ernoiz Antriyandarti, Davood Anvari, Jalal Arabloo, Hany Ariffin, Benedetta Armocida, Zatollah Asemi, Seyyed Shamsadin Athari, Gamechu Hunde Atomsa, Prince Atorkey, Ashish D Badiye, Sara Bagherieh, Atif Amin Baig, Madhan Balasubramanian, Ovidiu Constantin Baltatu, Maciej Banach, Palash Chandra Banik, Martina Barchitta, Till Winfried Bärnighausen, Amadou Barrow, Alehegn Bekele, Sefealem Assefa Belay, Uzma Iqbal Belgaumi, Shelly L Bell, Derrick A Bennett, Habtamu B Beyene, Akshaya Srikanth Bhagavathula, Sonu Bhaskar, Zulfiqar A Bhutta, Milad Bonakdar Hashemi, Dejana Braithwaite, Muhammad Hammad Butt, Luis Alberto Cámera, Luciana Aparecida Campos, Chao Cao, Carlos A Castañeda-Orjuela, Francieli Cembranel, Joshua Chadwick, Vijay Kumar Chattu, Abdulaal Chitheer, Dinh-Toi Chu, Rafael M Claro, Alyssa Columbus, Samuele Cortese, Natalia Cruz-Martins, Xiaochen Dai, Lalit Dandona, Rakhi Dandona, Aso Mohammad Darwesh, Amira Hamed Darwish, Saswati Das, Fernando Pio De la Hoz, Aklilu Tamire Debele, Hardik Dineshbhai Desai, Meghnath Dhimal, Diana Dias da Silva, Haneil Larson Dsouza, Michael Ekholuenetale, Temitope Cyrus Ekundayo, Habtamu Esubalew, Adeniyi Francis Fagbamigbe, Ildar Ravisovich Fakhradiyev, Luca Falzone, Abidemi Omolara Fasanmi, Ali Fatehizadeh, Alireza Feizkhah, Ryan Fitzgerald, David Flood, Morenike Oluwatoyin Folayan, Takeshi Fukumoto, Muktar A Gadanya, Santosh Gaihre, Yaseen Galali, William M Gardner, Teferi Gebru Gebremeskel, Lemma Getacher, Solomon Getawa, Kazem Ghaffari, Sherief Ghozy, James C Glasbey, Mahaveer Golechha, Pouya Goleij, Habtamu Alganeh Guadie, Avirup Guha, Sapna Gupta, Veer Bala Gupta, Vivek Kumar Gupta, Arvin Haj-Mirzaian, Harapan Harapan, Josep Maria Haro, Soheil Hassanipour, Claudiu Herteliu, Demisu Zenbaba Heyi, Praveen Hoogar, Sheikh Jamal Hossain, Mehdi Hosseinzadeh, Salman Hussain, Licia Iacoviello, Farideh Iravanpour, Sheikh Mohammed Shariful Islam, Nahlah Elkudssiah Ismail, Gaetano Isola, Linda Merin J, Haitham Jahrami, Mihajlo Jakovljevic, Sathish Kumar Jayapal, Shubha Jayaram, Bijay Mukesh Jeswani, Jost B Jonas, Abel Joseph, Charity Ehimwenma Joshua, Jacek Jerzy Jozwiak, Mikk Jürisson, Billingsley Kaambwa, Zubair Kabir, Vidya Kadashetti, Himal Kandel, Neeti Kapoor, Nicholas J Kassebaum, Patrick DMC Katoto, Harkiran Kaur, Gbenga A Kayode, Worku Misganaw Kebede, Jemal Yusuf Kebira, Tibebeselassie S Keflie, Mohammad Keykhaei, Yousef Saleh Khader, Himanshu Khajuria, Nauman Khalid, Mohammad Khammarnia, M Nuruzzaman Khan, Moien AB Khan, Yusra H Khan, Khaled Khatab, Sorour Khateri, Mahalaqua Nazli Khatib, Jagdish Khubchandani, Zemene Demelash Kifle, Gyu Ri Kim, Adnan Kisa, Sezer Kisa, Shivakumar KM Marulasiddaiah Kondlahalli, Oleksii Korzh, Sindhura Lakshmi Koulmane

Laxminarayana, Kewal Krishan, Vijay Krishnamoorthy, Barthelemy Kuate Defo, Burcu Kucuk Bicer, G Anil Kumar, Manasi Kumar, Dian Kusuma, Dharmesh Kumar Lal, Kamaluddin Latief, Caterina Ledda, Sang-woong Lee, Virendra S Ligade, Stephen S Lim, László Lorenzovici, Stefan Lorkowski, Farzan Madadizadeh, Tauqeer Hussain Mallhi, Deborah Carvalho Malta, Abdoljalal Marjani, Awoke Masrie, Andrea Maugeri, Maryam Mazaheri, Rishi P Mediratta, Man Mohan Mehndiratta, Yohannes Adama Melaku, Walter Mendoza, Ritesh G Menezes, Tuomo J Meretoja, GK Mini, Erkin M Mirrakhimov, Abdollah Mohammadian-Hafshejani, Shafiu Mohammed, Ali H Mokdad, Mohammad Ali Moni, Maryam Moradi, Yousef Moradi, Vincent Mougin, Sumaira Mubarik, Francesk Mulita, Efren Murillo-Zamora, Christopher J L Murray, Ahamarshan Jayaraman Nagarajan, Sreenivas Narasimha Swamy, Zuhair S Natto, Biswa Prakash Nayak, Josephine W Ngunjiri, QuynhAnh P Nguyen, Robina Khan Niazi, Chukwudi A Nnaji, Jean Jacques Noubiap, Dieta Nurrika, Bogdan Oancea, Kehinde O Obamiro, Onome Bright Oghenetega, Osaretin Christabel Okonji, Andrew T Olagunju, Bolajoko Olubukunola Olusanya, Jacob Olusegun Olusanya, Sok King Ong, Alberto Ortiz, Sergej M Ostojic, Adrian Otoiu, Jagadish Rao Padubidri, Adrian Pana, Songhomitra Panda-Jonas, Anamika Pandey, Seithikurippu R Pandi-Perumal, Paraskevi Papadopoulou, Shahina Pardhan, Maja Pasovic, Jay Patel, Aslam Ramjan Pathan, Shrikant Pawar, Veincent Christian Filipino Pepito, Gavin Pereira, Simone Perna, Zahra Zahid Piracha, Naeimeh Pourtaheri, Catalina Raggi, Pankaja Raghav, Fakher Rahim, Vafa Rahimi-Movaghar, Amir Masoud Rahmani, Sathish Rajaa, Pradhum Ram, Shakthi Kumaran Ramasamy, Sheena Ramazanu, Chythra R Rao, Sowmya J Rao, Ahmed Mustafa Rashid, Zubair Ahmed Ratan, Salman Rawaf, Lal Rawal, Kannan RR Rengasamy, Andre M N Renzaho, Alina Rodriguez, Jefferson Antonio Buendia Rodriguez, Leonardo Roever, Peter Rohloff, Godfrey M Rwegerera, Aly M A Saad, Siamak Sabour, Basema Ahmad Saddik, Umar Saeed, Mirza Rizwan Sajid, Marwa Rashad Salem, Abdallah M Samy, Juan Sanabria, Itamar S Santos, Milena M Santric-Milicevic, Sivan Yegnanarayana Iyer Saraswathy, Maheswar Satpathy, Monika Sawhney, Markus P Schlaich, Subramanian Senthilkumaran, Dragos Serban, Pritik A Shah, Masood Ali Shaikh, Luís Manuel Lopes Rodrigues Silva, Jasvinder A Singh, Narinder Pal Singh, Paramdeep Singh, Anna Aleksandrovna Skryabina, Yonatan Solomon, Jeffrey D Stanaway, Muhammad Suleman, Mindy D Szeto, Rafael Tabarés-Seisdedos, Shima Tabatabai, Moslem Taheri Soodejani, Ker-Kan Tan, Zerihun Tariku, Elvis Enowbeyang Tarkang, Yibekal Manaye Tefera, Pugazhenthan Thangaraju, Tala Tillawi, Tenaw Yimer Tiruye, Roman Topor-Madry, Mathilde Touvier, Marcos Roberto Tovani-Palone, Mai Thi Ngoc Tran, Sana Ullah, Bhaskaran Unnikrishnan, Sahel Valadan Tahbaz, Jef Van den Eynde, Shoban Babu Varthya, Tommi Juhani Vasankari, Narayanaswamy Venketasubramanian, Vasily Vlassov, Yasir Waheed, Molla Mesele Wassie, Kosala Gayan Weerakoon, Gedif Ashebir Wubetie, Suowen Xu, Kazumasa Yamagishi, Yuichiro Yano, Sanni Yaya, Dong Keon Yon, Chuanhua Yu, Iman Zare, Michael Zastrozhin, and Mohammad Zoladl.

Developing methods or computational machinery

Qorinah Estiningtyas Sakilah Adnani, Saira Afzal, Ali Ahmadi, Ali Ahmed, Hubert Amu, Davood Anvari, Aleksandr Y Aravkin, Akshaya Srikanth Bhagavathula, Natalia V Bhattacharjee, Milad Bonakdar Hashemi, Muhammad Hammad Butt, Joshua Chadwick, Dinh-Toi Chu, Xiaochen Dai, Aso Mohammad Darwesh, Hardik Dineshbhai Desai, Michael Ekholuenetale, Adeniyi Francis Fagbamigbe, Ali Fatehizadeh, Ryan Fitzgerald, William M Gardner, Kazem Ghaffari, Fariba Ghassemi, Sherief Ghozy, Simon I Hay, Jiawei He, Kamran Hessami, Mehdi Hosseinzadeh, Olayinka Stephen Ilesanmi, Farideh Iravanpour, Gaetano Isola, Haitham Jahrami, Sathish Kumar Jayapal, Bijay Mukesh Jeswani, Charity Ehimwenma Joshua, Nicholas J Kassebaum, Jemal Yusuf Kebira, Tibebeselassie S Keflie, Mohammad Khammarnia, M Nuruzzaman Khan, Sorour Khateri, Mahalaqua Nazli Khatib, Adnan Kisa, Kamaluddin Latief, Sang-woong Lee, Paulina A

Lindstedt, Justin Lo, Awoke Masrie, Ali H Mokdad, Mohammad Ali Moni, Yousef Moradi, Vincent Mougin, Francesk Mulita, Christopher J L Murray, Ghulam Mustafa, Josephine W Ngunjiri, QuynhAnh P Nguyen, Seithikurippu R Pandi-Perumal, Maja Pasovic, Zahra Zahid Piracha, Catalina Raggi, Azizur Rahman, Amir Masoud Rahmani, Umar Saeed, Abdallah M Samy, Maheswar Satpathy, Ganesh Kumar Saya, Seyed Afshin Shorofi, Yi Song, Jeffrey D Stanaway, Muhammad Suleman, Zerihun Tariku, Yibekal Manaye Tefera, Tala Tillawi, Mai Thi Ngoc Tran, Shoban Babu Varthya, Stein Emil Vollset, and Chun-Wei Yuan.

Providing critical feedback on methods or results

Yohannes Habtegiorgis Abate, Samar Abd ElHafeez, Michael Abdelmasseh, Sherief Abd-Elsalam, Deldar Morad Abdulah, Rizwan Suliankatchi Abdulkader, Hassan Abidi, Richard Gyan Aboagye, Hassan Abolhassani, Yonas Derso Abtew, Eman Abu-Gharbieh, Juan Manuel Acuna, Kidist Adamu, Denberu Eshetie Adane, Isaac Yeboah Addo, Daniel Adedayo Adeyinka, Qorinah Estiningtyas Sakilah Adnani, Aanuoluwapo Adeyimika Afolabi, Fatemeh Afrashteh, Saira Afzal, Antonella Agodi, Bright Opoku Ahinkorah, Aqeel Ahmad, Sajjad Ahmad, Tauseef Ahmad, Ali Ahmadi, Ali Ahmed, Luai A A Ahmed, Budi Aji, Hossein Akbarialiabad, Maxwell Akonde, Hanadi Al Hamad, Yazan Al Thaher, Ziyad Al-Aly, Khalid F Alhabib, Robert Kaba Alhassan, Syed Shujait Ali, Yousef Alimohamadi, Syed Mohamed Aljunid, Hesham M Al-Mekhlafi, Sami Almustanyir, Mahmoud A Alomari, Alaa B Al-Tammemi, Khalid A Altirkawi, Nelson Alvis-Guzman, Nelson J Alvis-Zakzuk, Edward Kwabena Ameyaw, Tarek Tawfik Amin, Sohrab Amiri, Hubert Amu, Dickson A Amugsi, Tadele Fentabel Fentabil Anagaw, Robert Ancuceanu, Dhanalakshmi Angappan, Alireza Ansari-Moghaddam, Ernoiz Antriyandarti, Davood Anvari, Anayochukwu Edward Anyasodor, Jalal Arabloo, Hany Ariffin, Timur Aripov, Mesay Arkew, Benedetta Armocida, Ashokan Arumugam, Ni Ketut Aryastami, Mulu Tiruneh Asemu, Mohammad Asghari-Jafarabadi, Thomas Astell-Burt, Seyyed Shamsadin Athari, Gamechu Hunde Atomsa, Prince Atorkey, Maha Moh'd Wahbi Atout, Mamaru Ayenew Awoke, Sina Azadnajafabad, Rui M S Azevedo, Darshan B B, Nayereh Baghcheghi, Nasser Bagheri, Sara Bagherieh, Atif Amin Baig, Jennifer L Baker, Ovidiu Constantin Baltatu, Maciej Banach, Palash Chandra Banik, Martina Barchitta, Till Winfried Bärnighausen, Amadou Barrow, Lingkan Barua, Azadeh Bashiri, Pritish Baskaran, Saurav Basu, Alehegn Bekele, Sefealem Assefa Belay, Uzma Iqbal Belgaumi, Shelly L Bell, Derrick A Bennett, Isabela M Bensenor, Girma Beressa, Amiel Nazer C Bermudez, Habtamu B Beyene, Akshaya Srikanth Bhagavathula, Nikha Bhardwaj, Pankaj Bhardwaj, Sonu Bhaskar, Natalia V Bhattacharjee, Zulfiqar A Bhutta, Virginia Bodolica, Milad Bonakdar Hashemi, Dejana Braithwaite, Muhammad Hammad Butt, Zahid A Butt, Chao Cao, Rosario Cárdenas, Márcia Carvalho, Alberico L Catapano, Francieli Cembranel, Ester Cerin, Joshua Chadwick, Eeshwar K Chandrasekar, Jaykaran Charan, Vijay Kumar Chattu, Kirti Chauhan, Sonali Gajanan Choudhari, Enayet Karim Chowdhury, Dinh-Toi Chu, Isaac Sunday Chukwu, Sheng-Chia Chung, Alyssa Columbus, Samuele Cortese, Natalia Cruz-Martins, Bashir Dabo, Omid Dadras, Xiaochen Dai, Emanuele D'Amico, Lalit Dandona, Rakhi Dandona, Isaac Darban, Gary L Darmstadt, Aso Mohammad Darwesh, Amira Hamed Darwish, Jai K Das, Saswati Das, Kairat Davletov, Fernando Pio De la Hoz, Aklilu Tamire Debele, Dessalegn Demeke, Solomon Demissie, Hardik Dineshbhai Desai, Abebaw Alemayehu Desta, Samath Dhamminda Dharmaratne, Meghnath Dhimal, Diana Dias da Silva, Daniel Diaz, Mengistie Diress, Shirin Djalalinia, Saeid Doaei, Deepa Dongarwar, Haneil Larson Dsouza, Sareh Edalati, Hisham Atan Edinur, Michael Ekholuenetale, Temitope Cyrus Ekundayo, Islam Y Elgendy, Muhammed Elhadi, Omar Abdelsadek Abdou Elmeligy, Habitu Birhan Eshetu, Juan Espinosa-Montero, Habtamu Esubalew, Farshid Etaee, Adeniyi Francis Fagbamigbe, Ildar Ravisovich Fakhradiyev, Luca Falzone, Carla Sofia e Sá Farinha, Abidemi Omolara Fasanmi, Ali Fatehizadeh, Valery L Feigin, Alireza Feizkhah, Xiaoqi Feng, Getahun Fetensa, Florian

Fischer, Ryan Fitzgerald, David Flood, Nataliya A Foigt, Morenike Oluwatoyin Folayan, Kayode Raphael Fowobaje, Richard Charles Franklin, Takeshi Fukumoto, Muktar A Gadanya, Abhay Motiramji Gaidhane, Santosh Gaihre, Emmanuela Gakidou, Yaseen Galali, Nasrin Galehdar, William M Gardner, Priyanka Garg, Teferi Gebru Gebremeskel, Lemma Getacher, Motuma Erena Getachew, Solomon Getawa, Kazem Ghaffari, Mohammad Ghasemi Nour, Fariba Ghassemi, Nermin Ghith, Maryam Gholamalizadeh, Ali Gholami, Ali Gholamrezanezhad, Sherief Ghozy, Paramjit Singh Gill, Tiffany K Gill, James C Glasbey, Mahaveer Golechha, Davide Golinelli, Houman Goudarzi, Michal Grivna, Habtamu Alganeh Guadie, Mohammed Ibrahim Mohialdeen Gubari, Temesgen Worku Gudayu, Avirup Guha, Damitha Asanga Gunawardane, Anish Kumar Gupta, Bhawna Gupta, Rahul Gupta, Sapna Gupta, Veer Bala Gupta, Vivek Kumar Gupta, Arvin Haj-Mirzaian, Asif Hanif, Harapan Harapan, Ahmed I Hasaballah, Md Mehedi Hasan, Hamidreza Hasani, Abdiwahab Hashi, Soheil Hassanipour, Rasmus J Havmoeller, Simon I Hay, Khezar Hayat, Mahsa Heidari-Foroozan, Claudiu Herteliu, Kamran Hessami, Demisu Zenbaba Heyi, Kamal Hezam, Yuta Hiraike, Ramesh Holla, Praveen Hoogar, Sheikh Jamal Hossain, Mehdi Hosseinzadeh, Mihaela Hostiuc, Soodabeh Hoveidamanesh, Salman Hussain, Foziya Mohammed Hussien, Bing-Fang Hwang, Pulwasha Maria Iftikhar, Olayinka Stephen Ilesanmi, Irena M Ilic, Milena D Ilic, Mustapha Immurana, Leeberk Raja Inbaraj, Farideh Iravanpour, Sheikh Mohammed Shariful Islam, Farhad Islami, Nahlah Elkudssiah Ismail, Gaetano Isola, Masao Iwagami, Chidozie Declan Iwu, Louis Jacob, Haitham Jahrami, Mihajlo Jakovljevic, Elham Jamshidi, Sathish Kumar Jayapal, Shubha Jayaram, Rime Jebai, Alelign Tasew Jema, Bijay Mukesh Jeswani, Jost B Jonas, Abel Joseph, Nitin Joseph, Charity Ehimwenma Joshua, Jacek Jerzy Jozwiak, Mikk Jürisson, Billingsley Kaambwa, Ali Kabir, Zubair Kabir, Vidya Kadashetti, Vineet Kumar Kamal, Bhushan Dattatray Kamble, Himal Kandel, Ibraheem M Karaye, Nicholas J Kassebaum, Patrick DMC Katoto, Joonas H Kauppila, Harkiran Kaur, Gbenga A Kayode, Worku Misganaw Kebede, Jemal Yusuf Kebira, Tibebeselassie S Keflie, Jessica A Kerr, Yousef Saleh Khader, Himanshu Khajuria, Nauman Khalid, Mohammad Khammarnia, M Nuruzzaman Khan, Moien AB Khan, Taimoor Khan, Yusra H Khan, Javad Khanali, Shaghayegh Khanmohammadi, Khaled Khatab, Moawiah Mohammad Khatatbeh, Sorour Khateri, Mahalaqua Nazli Khatib, Zemene Demelash Kifle, Gyu Ri Kim, Ruth W Kimokoti, Adnan Kisa, Sezer Kisa, Farzad Kompani, Shivakumar KM Marulasiddaiah Kondlahalli, Hamid Reza Koohestani, Oleksii Korzh, Sindhura Lakshmi Koulmane Laxminarayana, Ai Koyanagi, Kewal Krishan, Vijay Krishnamoorthy, Barthelemy Kuate Defo, Burcu Kucuk Bicer, Mohammed Kuddus, G Anil Kumar, Manasi Kumar, Nithin Kumar, Om P Kurmi, Dian Kusuma, Carlo La Vecchia, Ben Lacey, Dharmesh Kumar Lal, Anders O Larsson, Kamaluddin Latief, Caterina Ledda, Sang-woong Lee, Wei-Chen Lee, Yo Han Lee, Jacopo Lenzi, Ming-Chieh Li, Wei Li, Virendra S Ligade, Stephen S Lim, Chun-Han Lo, Justin Lo, Rakesh Lodha, Arianna Maever Loreche, László Lorenzovici, Stefan Lorkowski, Farzan Madadizadeh, Áurea M Madureira-Carvalho, Preetam Bhalchandra Mahajan, Konstantinos Christos Makris, Elaheh Malakan Rad, Ahmad Azam Malik, Tauqeer Hussain Mallhi, Deborah Carvalho Malta, Santi Martini, Miquel Martorell, Awoke Masrie, Elezebeth Mathews, Andrea Maugeri, Rishi P Mediratta, Man Mohan Mehndiratta, Yohannes Adama Melaku, Walter Mendoza, Ritesh G Menezes, Alexios-Fotios A Mentis, Tomislav Mestrovic, Tomasz Miazgowski, Ted R Miller, GK Mini, Andreea Mirica, Erkin M Mirrakhimov, Moonis Mirza, Sanjeev Misra, Prasanna Mithra, Abdollah Mohammadian-Hafshejani, Shafiu Mohammed, Mohammad Mohseni, Ali H Mokdad, Mohammad Ali Moni, Maryam Moradi, Yousef Moradi, Shane Douglas Morrison, Sumaira Mubarik, Ulrich Otto Mueller, Francesk Mulita, Efren Murillo-Zamora, Christopher J L Murray, Ghulam Mustafa, Ahamarshan Jayaraman Nagarajan, Vinay Nangia, Sreenivas Narasimha Swamy, Zuhair S Natto, Muhammad Naveed, Biswa Prakash Nayak, Seyed Aria Nejadghaderi, Georges Nguefack-Tsague, Josephine W Ngunjiri, Phuong The Nguyen, Robina Khan Niazi, Chukwudi A

Nnaji, Jean Jacques Noubiap, Chisom Adaobi Nri-Ezedi, Vincent Ebuka Nwatah, Bogdan Oancea, Kehinde O Obamiro, Onome Bright Oghenetega, Ropo Ebenezer Ogunsakin, Hassan Okati-Aliabad, Akinkunmi Paul Okekunle, Daniel Micheal Okello, Osaretin Christabel Okonji, Andrew T Olagunju, Gláucia Maria Moraes Oliveira, Bolajoko Olubukunola Olusanya, Jacob Olusegun Olusanya, Doris V Ortega-Altamirano, Alberto Ortiz, Sergej M Ostojic, Adrian Otoiu, Abdu Oumer, Jagadish Rao Padubidri, Adrian Pana, Songhomitra Panda-Jonas, Anamika Pandey, Seithikurippu R Pandi-Perumal, Paraskevi Papadopoulou, Shahina Pardhan, Maja Pasovic, Jay Patel, Aslam Ramjan Pathan, Deepak Paudel, Shrikant Pawar, Veincent Christian Filipino Pepito, Gavin Pereira, Marcos Pereira, Simone Perna, Ionela-Roxana Petcu, Fanny Emily Petermann-Rocha, Zahra Zahid Piracha, Naeimeh Pourtaheri, Amir Radfar, Venkatraman Radhakrishnan, Pankaja Raghav, Fakher Rahim, Vafa Rahimi-Movaghar, Azizur Rahman, Md Mosfequr Rahman, Md Obaidur Rahman, Mosiur Rahman, Muhammad Aziz Rahman, Amir Masoud Rahmani, Vahid Rahmanian, Setyaningrum Rahmawaty, Rajesh Kumar Rai, Sathish Rajaa, Prashant Rajput, Pradhum Ram, Shakthi Kumaran Ramasamy, Chythra R Rao, Indu Ramachandra Rao, Sowmya J Rao, Drona Prakash Rasali, Ahmed Mustafa Rashid, Mohammad-Mahdi Rashidi, Zubair Ahmed Ratan, Salman Rawaf, Lal Rawal, Elrashdy M Moustafa Mohamed Redwan, Kannan RR Rengasamy, Andre M N Renzaho, Nazila Rezaei, Mohsen Rezaeian, Abanoub Riad, Jennifer Rickard, Alina Rodriguez, Jefferson Antonio Buendia Rodriguez, Leonardo Roever, Peter Rohloff, Godfrey M Rwegerera, Chandan S N, Aly M A Saad, Maha Mohamed Saber-Ayad, Siamak Sabour, Basema Ahmad Saddik, Erfan Sadeghi, Malihe Sadeghi, Saeid Sadeghian, Umar Saeed, Sahar Saeedi Moghaddam, Sher Zaman Safi, Fatemeh Saheb Sharif-Askari, Harihar Sahoo, Soumya Swaroop Sahoo, Mirza Rizwan Sajid, Marwa Rashad Salem, Abdallah M Samy, Juan Sanabria, Rama Krishna Sanjeev, Milena M Santric-Milicevic, Sivan Yegnanarayana Iyer Saraswathy, Saman Sargazi, Yaser Sarikhani, Maheswar Satpathy, Monika Sawhney, Ganesh Kumar Saya, Abu Sayeed, Nikolaos Scarmeas, Markus P Schlaich, Aletta Elisabeth Schutte, Subramanian Senthilkumaran, Sadaf G Sepanlou, Dragos Serban, Mahan Shafie, Pritik A Shah, Ataollah Shahbandi, Masood Ali Shaikh, Adisu Tafari T Shama, Mehran Shams-Beyranvand, Mohd Shanawaz, Mequannent Melaku Sharew, Rahman Shiri, Seyed Afshin Shorofi, Kerem Shuval, Migbar Mekonnen Sibhat, Luís Manuel Lopes Rodrigues Silva, Jasvinder A Singh, Narinder Pal Singh, Paramdeep Singh, Anna Aleksandrovna Skryabina, Amanda E Smith, Yonatan Solomon, Yi Song, Jeffrey D Stanaway, Muhammad Suleman, Jing Sun, Dev Ram Sunuwar, Mindy D Szeto, Rafael Tabarés-Seisdedos, Seyed-Amir Tabatabaeizadeh, Shima Tabatabai, Jacques Lukenze JL Tamuzi, Ker-Kan Tan, Ingan Ukur Tarigan, Zerihun Tariku, Md Tariqujjaman, Elvis Enowbeyang Tarkang, Birhan Tsegaw Taye, Yibekal Manaye Tefera, Mohamad-Hani Temsah, Masayuki Teramoto, Pugazhenthan Thangaraju, Rekha Thapar, Arulmani Thiyagarajan, Amanda G Thrift, Jansje Henny Vera Ticoalu, Tala Tillawi, Tenaw Yimer Tiruye, Marcello Tonelli, Roman Topor-Madry, Mathilde Touvier, Marcos Roberto Tovani-Palone, Mai Thi Ngoc Tran, Sana Ullah, Bhaskaran Unnikrishnan, Tolassa Wakayo Ushula, Seyed Mohammad Vahabi, Alireza Vakilian, Sahel Valadan Tahbaz, Rohollah Valizadeh, Jef Van den Eynde, Shoban Babu Varthya, Narayanaswamy Venketasubramanian, Madhur Verma, Massimiliano Veroux, Dominique Vervoort, Stein Emil Vollset, Rade Vukovic, Yasir Waheed, Cong Wang, Fang Wang, Molla Mesele Wassie, Kosala Gayan Weerakoon, Melissa Y Wei, Andrea Werdecker, Nuwan Darshana Wickramasinghe, Asrat Arja Wolde, Gedif Ashebir Wubetie, Ratna Dwi Wulandari, Rongbin Xu, Suowen Xu, Xiaoyue Xu, Sanni Yaya, Fereshteh Yazdanpanah, Sisay Shewasinad Yehualashet, Arzu Yiğit, Vahit Yiğit, Dong Keon Yon, Chuanhua Yu, Giulia Zamagni, Sojib Bin Zaman, Aurora Zanghì, Moein Zangiabadian, Michael Zastrozhin, Mohammad Zoladl, and Zhiyong Zou.

Drafting the work or revising it critically for important intellectual content

Yohannes Habtegiorgis Abate, Mohsen Abbasi-Kangevari, Samar Abd ElHafeez, Michael Abdelmasseh, Sherief Abd-Elsalam, Hassan Abidi, Olumide Abiodun, Hassan Abolhassani, Eman Abu-Gharbieh, Niveen ME Abu-Rmeileh, Juan Manuel Acuna, Denberu Eshetie Adane, Isaac Yeboah Addo, Daniel Adedayo Adeyinka, Qorinah Estiningtyas Sakilah Adnani, Aanuoluwapo Adeyimika Afolabi, Saira Afzal, Antonella Agodi, Bright Opoku Ahinkorah, Ali Ahmadi, Luai A A Ahmed, Marjan Ajami, Hossein Akbarialiabad, Yazan Al Thaher, Khalid F Alhabib, Robert Kaba Alhassan, Syed Shujait Ali, Sami Almustanyir, Mahmoud A Alomari, Alaa B Al-Tammemi, Nelson Alvis-Guzman, Nelson J Alvis-Zakzuk, Tarek Tawfik Amin, Sohrab Amiri, Hubert Amu, Dickson A Amugsi, Tadele Fentabel Fentabil Anagaw, Robert Ancuceanu, Dhanalakshmi Angappan, Anayochukwu Edward Anyasodor, Jalal Arabloo, Hany Ariffin, Timur Aripov, Mesay Arkew, Benedetta Armocida, Michael Benjamin Arndt, Ashokan Arumugam, Malke Asaad, Mulu Tiruneh Asemu, Seyyed Shamsadin Athari, Gamechu Hunde Atomsa, Prince Atorkey, Maha Moh'd Wahbi Atout, Avinash Aujayeb, Mamaru Ayenew Awoke, Sina Azadnajafabad, Rui M S Azevedo, Ashish D Badiye, Sara Bagherieh, Atif Amin Baig, Jennifer L Baker, Madhan Balasubramanian, Ovidiu Constantin Baltatu, Maciej Banach, Martina Barchitta, Till Winfried Bärnighausen, Amadou Barrow, Azadeh Bashiri, Pritish Baskaran, Alehegn Bekele, Uzma Iqbal Belgaumi, Shelly L Bell, Luis Belo, Isabela M Bensenor, Girma Beressa, Habtamu B Beyene, Akshaya Srikanth Bhagavathula, Sonu Bhaskar, Saeid Bitaraf, Virginia Bodolica, Milad Bonakdar Hashemi, Dejana Braithwaite, Muhammad Hammad Butt, Daniela Calina, Márcia Carvalho, Carlos A Castañeda-Orjuela, Alberico L Catapano, Maria Sofia Cattaruzza, Francieli Cembranel, Ester Cerin, Joshua Chadwick, Eeshwar K Chandrasekar, Vijay Kumar Chattu, Ju-Huei Chien, Dinh-Toi Chu, Rafael M Claro, Alyssa Columbus, Samuele Cortese, Natalia Cruz-Martins, Bashir Dabo, Emanuele D'Amico, Gary L Darmstadt, Aklilu Tamire Debele, Edgar Denova-Gutiérrez, Hardik Dineshbhai Desai, Meghnath Dhimal, Diana Dias da Silva, Daniel Diaz, Deepa Dongarwar, Haneil Larson Dsouza, Sareh Edalati, Michael Ekholuenetale, Iffat Elbarazi, Islam Y Elgendy, Muhammed Elhadi, Omar Abdelsadek Abdou Elmeligy, Juan Espinosa-Montero, Habtamu Esubalew, Farshid Etaee, Werku Etafa, Adeniyi Francis Fagbamigbe, Luca Falzone, Ali Fatehizadeh, Pietro Ferrara, Getahun Fetensa, Florian Fischer, Nataliya A Foigt, Morenike Oluwatoyin Folayan, Takeshi Fukumoto, Muktar A Gadanya, Santosh Gaihre, Yaseen Galali, Nasrin Galehdar, Priyanka Garg, Teferi Gebru Gebremeskel, Urge Gerema, Lemma Getacher, Kazem Ghaffari, Seyyed-Hadi Ghamari, Mohammad Ghasemi Nour, Fariba Ghassemi, Nermin Ghith, Sherief Ghozy, Paramjit Singh Gill, Tiffany K Gill, James C Glasbey, Davide Golinelli, Michal Grivna, Temesgen Worku Gudayu, Avirup Guha, Damitha Asanga Gunawardane, Bhawna Gupta, Rahul Gupta, Sapna Gupta, Veer Bala Gupta, Vivek Kumar Gupta, Alexis J Handal, Graeme J Hankey, Harapan Harapan, Arief Hargono, Josep Maria Haro, Ahmed I Hasaballah, Hamidreza Hasani, Abdiwahab Hashi, Rasmus J Havmoeller, Simon I Hay, Khezar Hayat, Claudiu Herteliu, Kamran Hessami, Demisu Zenbaba Heyi, Kamal Hezam, Yuta Hiraike, Ramesh Holla, Sorin Hostiuc, Junjie Huang, Salman Hussain, Foziya Mohammed Hussien, Pulwasha Maria Iftikhar, Olayinka Stephen Ilesanmi, Irena M Ilic, Milena D Ilic, Mustapha Immurana, Farideh Iravanpour, Sheikh Mohammed Shariful Islam, Farhad Islami, Nahlah Elkudssiah Ismail, Hiroyasu Iso, Gaetano Isola, Chidozie Declan Iwu, Linda Merin J, Louis Jacob, Haitham Jahrami, Mihajlo Jakovljevic, Manthan Dilipkumar Janodia, Krishnamurthy Jayanna, Sathish Kumar Jayapal, Shubha Jayaram, Rime Jebai, Alelign Tasew Jema, Bijay Mukesh Jeswani, Jost B Jonas, Abel Joseph, Nitin Joseph, Charity Ehimwenma Joshua, Jacek Jerzy Jozwiak, Mikk Jürisson, Billingsley Kaambwa, Ali Kabir, Vidya Kadashetti, Himal Kandel, Neeti Kapoor, Nicholas J Kassebaum, Patrick DMC Katoto, Joonas H Kauppila, Gbenga A Kayode, Worku Misganaw Kebede, Jemal Yusuf Kebira, Tibebeselassie S Keflie, Jessica A Kerr, Yousef Saleh Khader, Himanshu Khajuria, Nauman Khalid, Mohammad Khammarnia, M

Nuruzzaman Khan, Moien AB Khan, Taimoor Khan, Yusra H Khan, Javad Khanali, Shaghayegh Khanmohammadi, Khaled Khatab, Moawiah Mohammad Khatatbeh, Sorour Khateri, Mahalaqua Nazli Khatib, Hamid Reza Khayat Kashani, Jagdish Khubchandani, Gyu Ri Kim, Adnan Kisa, Sezer Kisa, Farzad Kompani, Shivakumar KM Marulasiddaiah Kondlahalli, Oleksii Korzh, Sindhura Lakshmi Koulmane Laxminarayana, Ai Koyanagi, Kewal Krishan, Barthelemy Kuate Defo, Burcu Kucuk Bicer, Mohammed Kuddus, Almagul Kurmanova, Om P Kurmi, Dian Kusuma, Carlo La Vecchia, Ben Lacey, Anders O Larsson, Kamaluddin Latief, Caterina Ledda, Paul H Lee, Jacopo Lenzi, Wei Li, Chun-Han Lo, László Lorenzovici, Stefan Lorkowski, Farzan Madadizadeh, Áurea M Madureira-Carvalho, Preetam Bhalchandra Mahajan, Elaheh Malakan Rad, Ahmad Azam Malik, Tauqeer Hussain Mallhi, Deborah Carvalho Malta, Abdoljalal Marjani, Miquel Martorell, Awoke Masrie, Elezebeth Mathews, Andrea Maugeri, Rishi P Mediratta, Walter Mendoza, Ritesh G Menezes, George A Mensah, Alexios-Fotios A Mentis, Tuomo J Meretoja, Tomislav Mestrovic, Tomasz Miazgowski, Ted R Miller, Mojgan Mirghafourvand, Moonis Mirza, Prasanna Mithra, Karzan Abdulmuhsin Mohammad, Abdollah Mohammadian-Hafshejani, Shafiu Mohammed, Ali H Mokdad, Lorenzo Monasta, Mohammad Ali Moni, Maryam Moradi, Yousef Moradi, Shane Douglas Morrison, Ulrich Otto Mueller, Francesk Mulita, Daniel Munblit, Efren Murillo-Zamora, Christopher J L Murray, Ghulam Mustafa, Ahamarshan Jayaraman Nagarajan, Sreenivas Narasimha Swamy, Zuhair S Natto, Muhammad Naveed, Biswa Prakash Nayak, Seyed Aria Nejadghaderi, Georges Nguefack-Tsague, Josephine W Ngunjiri, Robina Khan Niazi, Nurulamin M Noor, Chisom Adaobi Nri-Ezedi, Dieta Nurrika, Vincent Ebuka Nwatah, Bogdan Oancea, Kehinde O Obamiro, Onome Bright Oghenetega, Akinkunmi Paul Okekunle, Daniel Micheal Okello, Osaretin Christabel Okonji, Andrew T Olagunju, Diriba Dereje Olana, Bolajoko Olubukunola Olusanya, Jacob Olusegun Olusanya, Doris V Ortega-Altamirano, Alberto Ortiz, Sergej M Ostojic, Adrian Otoiu, Abdu Oumer, Alicia Padron-Monedero, Jagadish Rao Padubidri, Songhomitra Panda-Jonas, Seithikurippu R Pandi-Perumal, Shahina Pardhan, Jay Patel, Shrikant Pawar, Veincent Christian Filipino Pepito, Gavin Pereira, Marcos Pereira, Norberto Perico, Ionela-Roxana Petcu, Fanny Emily Petermann-Rocha, Zahra Zahid Piracha, Nishad Plakkal, Amir Radfar, Venkatraman Radhakrishnan, Pankaja Raghav, Fakher Rahim, Vafa Rahimi-Movaghar, Azizur Rahman, Ivano Raimondo, Sathish Rajaa, Prashant Rajput, Pradhum Ram, Shakthi Kumaran Ramasamy, Chythra R Rao, Sowmya J Rao, Ahmed Mustafa Rashid, Mohammad-Mahdi Rashidi, Lal Rawal, Elrashdy M Moustafa Mohamed Redwan, Giuseppe Remuzzi, Kannan RR Rengasamy, Andre M N Renzaho, Malihe Rezaee, Nazila Rezaei, Abanoub Riad, Jennifer Rickard, Jefferson Antonio Buendia Rodriguez, Leonardo Roever, Peter Rohloff, Bedanta Roy, Godfrey M Rwegerera, Chandan S N, Aly M A Saad, Maha Mohamed Saber-Ayad, Siamak Sabour, Mamta Sachdeva Dhingra, Basema Ahmad Saddik, Malihe Sadeghi, Umar Saeed, Sahar Saeedi Moghaddam, Sher Zaman Safi, Fatemeh Saheb Sharif-Askari, Amirhossein Sahebkar, Soumya Swaroop Sahoo, Mirza Rizwan Sajid, Marwa Rashad Salem, Abdallah M Samy, Juan Sanabria, Senthilkumar Sankararaman, Itamar S Santos, Milena M Santric-Milicevic, Yaser Sarikhani, Maheswar Satpathy, Ganesh Kumar Saya, Abu Sayeed, Nikolaos Scarmeas, Markus P Schlaich, Aletta Elisabeth Schutte, Sadaf G Sepanlou, Dragos Serban, Allen Seylani, Mahan Shafie, Pritik A Shah, Adisu Tafari T Shama, Mehran Shams-Beyranvand, Mohd Shanawaz, Mequannent Melaku Sharew, Pavanchand H Shetty, Velizar Shivarov, Seyed Afshin Shorofi, Kerem Shuval, Migbar Mekonnen Sibhat, Luís Manuel Lopes Rodrigues Silva, Jasvinder A Singh, Narinder Pal Singh, Paramdeep Singh, Surjit Singh, Anna Aleksandrovna Skryabina, Amanda E Smith, Yonatan Solomon, Yi Song, Mu'awiyyah Babale Sufiyan, Muhammad Suleman, Dev Ram Sunuwar, Seyed-Amir Tabatabaeizadeh, Shima Tabatabai, Jacques Lukenze JL Tamuzi, Ker-Kan Tan, Zerihun Tariku, Nathan Y Tat, Birhan Tsegaw Taye, Yibekal Manaye Tefera, Arash Tehrani-Banihashemi, Mohamad-Hani Temsah, Masayuki Teramoto, Pugazhenthan Thangaraju, Arulmani

Thiyagarajan, Ales Tichopad, Tala Tillawi, Tenaw Yimer Tiruye, Marcello Tonelli, Roman Topor-Madry, Mathilde Touvier, Marcos Roberto Tovani-Palone, Mai Thi Ngoc Tran, Sana Ullah, Eduardo A Undurraga, Bhaskaran Unnikrishnan, Tolassa Wakayo Ushula, Sahel Valadan Tahbaz, Jef Van den Eynde, Shoban Babu Varthya, Tommi Juhani Vasankari, Narayanaswamy Venketasubramanian, Madhur Verma, Massimiliano Veroux, Dominique Vervoort, Vasily Vlassov, Stein Emil Vollset, Rade Vukovic, Cong Wang, Fang Wang, Molla Mesele Wassie, Kosala Gayan Weerakoon, Melissa Y Wei, Andrea Werdecker, Nuwan Darshana Wickramasinghe, Gedif Ashebir Wubetie, Lalit Yadav, Kazumasa Yamagishi, Lin Yang, Sanni Yaya, Sisay Shewasinad Yehualashet, Arzu Yiğit, Vahit Yiğit, Sojib Bin Zaman, Aurora Zanghì, Moein Zangiabadian, Iman Zare, Michael Zastrozhin, and Mohammad Zoladl.

Managing the estimation or publications process

Hailey Hagins, Simon I Hay, Nicholas J Kassebaum, Paulina A Lindstedt, Ali H Mokdad, Maja Pasovic, Rachel D Schneider, Amanda E Smith, and Jeffrey D Stanaway.
